# Compact RNA sensors for increasingly complex functions of multiple inputs

**DOI:** 10.1038/s41557-025-01907-8

**Published:** 2025-11-12

**Authors:** Christian A. Choe, Johan O. L. Andreasson, Feriel Melaine, Wipapat Kladwang, Michelle J. Wu, Fernando Portela, Roger Wellington-Oguri, John J. Nicol, Hannah K. Wayment-Steele, Michael Gotrik, Eterna Participants, Purvesh Khatri, William J. Greenleaf, Rhiju Das

**Affiliations:** 1 Department of Bioengineering, Stanford University School of Medicine, Stanford, CA USA; 2 Department of Genetics, Stanford University School of Medicine, Stanford, CA USA; 3 Department of Biochemistry, Stanford University School of Medicine, Stanford, CA USA; 4 Program in Biomedical Informatics, Stanford University School of Medicine, Stanford, CA USA; 5 Eterna Massive Open Laboratory, Seattle, USA; 6 Department of Chemistry, Stanford University, Stanford, CA USA; 7 Stanford Center for Biomedical Informatics Research, Stanford University, Stanford, CA USA; 8 Stanford Institute for Immunity, Transplantation and Infection, Stanford University School of Medicine, Stanford, CA USA; 9 Howard Hughes Medical Institute, Stanford University, Stanford, CA USA; 10 Present Address: Advanced Energy Industries, Inc., Fort Collins, CO USA; 11 Present Address: Verily Life Sciences, San Francisco, CA USA; 12 Present Address: Protillion Biosciences, Burlingame, CA USA

**Keywords:** Synthetic biology, RNA nanotechnology, Biosensors

## Abstract

Designing single molecules that compute general functions of input molecular partners is a major unsolved challenge in molecular design. Here we demonstrate that high-throughput, iterative experimental testing of diverse RNA designs crowdsourced from the online game Eterna yields sensors of increasingly complex functions of input oligonucleotide concentrations. After designing single-input RNA sensors with activation ratios beyond our detection limits, we created logic gates, including challenging XOR and XNOR gates, and sensors that respond to the ratio of two inputs. Finally, we describe the OpenTB challenge, which elicited 85-nucleotide sensors that compute a score for diagnosing active tuberculosis based on the ratio of products of three gene segments. Building on OpenTB design strategies, we created an algorithm, Nucleologic, that produces similarly compact sensors for the three-gene score based on RNA and DNA. These results expand the possibilities for using compact, single-molecule sensors in a range of applications previously constrained by design complexity.

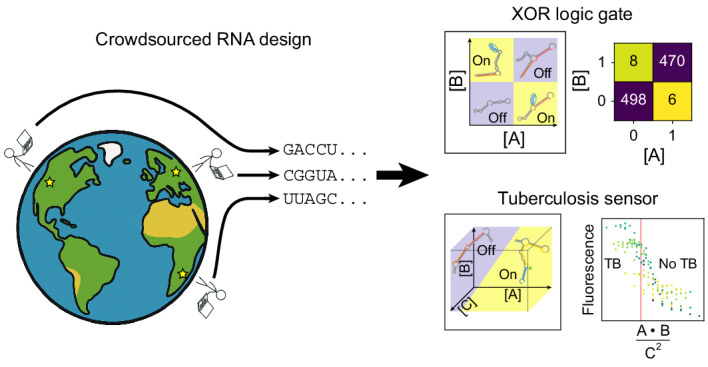

## Abstract (web summary)

Designing single molecules capable of complex sensing functions is challenging. Now, using crowdsourced RNA designs from the online game Eterna, compact single-molecule sensors have been demonstrated for a variety of tasks, including a complex three-input tuberculosis diagnostic. The development of a Monte Carlo Tree Search algorithm enabled automated design of similarly sophisticated nucleic-acid sensors.

## Main

Throughout biology, a large number of macromolecular systems carry out complex calculations essential for life, including cell-cycle regulation^1^, cell growth^2^ and tissue development^3^, but our ability to design comparably sophisticated biomolecular computers de novo remains primitive. Progress in the rational design of biomolecular computers would transform numerous fields; directed drug delivery, gene editors and biosensors are technologies that all would benefit from computations at a microscopic scale in complex cellular environments. Existing multicomponent molecular computers achieve complex functions^4–12^ but often require precise stoichiometry and numerous interacting parts, limiting their practicality in vivo. Computers that are instead based on single molecules might solve these issues and be capable of accurate complex computation in ambient cellular conditions or as ‘stand-alone’ computers outside cells^13,14^. Single-molecule computers may also offer superior thermodynamic efficiency, potentially creating new paradigms for low-energy computing^15,16^.

Is there a limit to the functional forms that a single molecule can approximate? The behaviours of macromolecules and their complexes have long been described through partition functions, which are ratios of two polynomials with non-negative coefficients. For example, expressions for haemoglobin behaviour involve terms up to the fourth order in the partial pressure of oxygen and the concentration of protons and small molecules^17,18^, and such expressions can compute complex logic^19^. Theoretically, there is no limit to the functional form a single molecule can approximate, but there may be a physical limit due to the kinetics and complexity of the molecule itself. Drawing motivation from this work and theorems derived for positive rational polynomials^20,21^, a single macromolecule complex at equilibrium that is capable of binding input molecules and output molecules should be able to approximate any bounded polynomial (Supplementary Appendix: Molecular rational function approximation). In this Article we more concretely explore the importance of these results by designing RNA-based approximators for functions involving polynomials with practical interest. The versatility of RNA as an allosteric biomolecular sensor is apparent in the diversity of natural ‘riboswitch’ molecules that change their structure to alter downstream regulation upon binding an input molecule, which could be a drug, a metabolite produced by a downstream pathway, or a protein binding partner^22^. Numerous examples of ‘tandem’ riboswitches exist that carry out computations involving multiple input ligands^23^. The versatility of RNA is also demonstrated by the diverse synthetic elements, including designed allosteric riboswitches^24,25^, therapeutics^26,27^ and diagnostics^28–30^, that have been implemented with RNA as a substrate.

As a driving application of a complex biomolecular calculation, we have taken inspiration from ratiometric gene signatures being discovered across diseases and host responses, including sepsis, cancers, malaria and pulmonary tuberculosis (TB)^31–34^. TB remains a major public health challenge worldwide, and the development of an accurate and accessible tool to discriminate active TB from latent TB and other diseases is critical. The World Health Organization has identified the need for a non-sputum-based triage test to identify individuals who require further testing^35^. In this context, the use of a three-gene transcriptional biomarker comprising concentrations of mRNA genes for guanylate binding protein 5 (*GBP5*), dual specificity phosphatase 3 (*DUSP3*) and Krüppel-like transcription factor 2 (*KLF2*) has emerged as a promising signature for TB diagnosis due to its specificity and sensitivity^36–38^. These genes collectively form a three-gene signature referred to as Sweeney3 or, in this Article, the ‘TB-score’. Sweeney and colleagues identified this combinatorial score based on blood messenger RNA (mRNA) expression levels, demonstrating its potential for discriminating active TB from other diseases. However, the complexity of the TB-score, which involves the quantity [*GBP5*][*DUSP3*]/[*KLF2*]^2^, currently requires expensive equipment involving quantitative polymerase chain reaction with reverse transcription (RT–qPCR). A single-strand nucleic-acid sensor that could carry out the TB-score computation in samples after or during cell-free RNA amplification could enable an alternative method for point-of-care diagnostics in low-resource settings^30,39,40^.

**Fig. 1:**
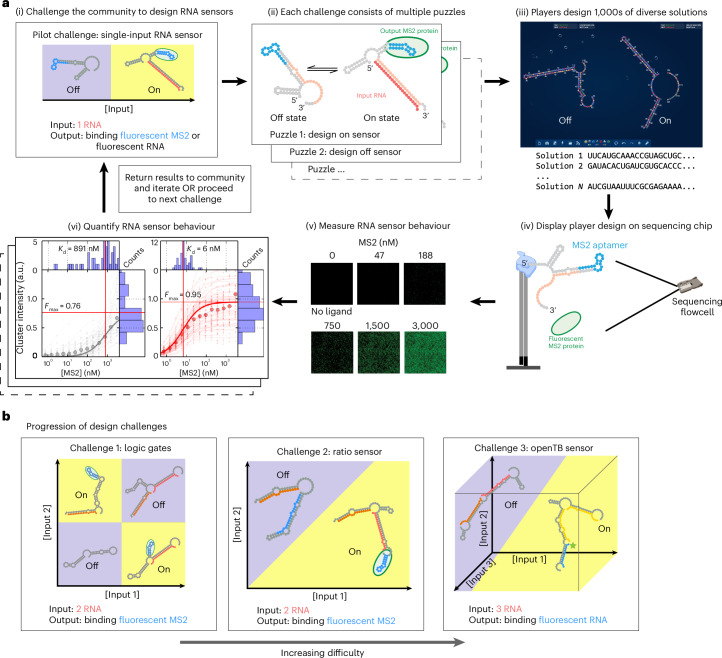
Pipeline for crowdsourced RNA sensor design and high-throughput testing. **a**, Workflow for crowdsourced RNA sensor design. (i) RNA sensor design challenges were presented to the Eterna community. (ii) Each challenge consisted of multiple puzzles such as designing an on or off sensor for the specified inputs and outputs. (iii) The Eterna interface enabled players to design RNAs with two or more states with feedback from RNA folding packages; players designed thousands of diverse solutions. (iv) Player designs were synthesized by DNA array synthesis and converted to libraries ready for RNA-MaP characterization. (v) Binding of a fluorescent output reporter was quantified across all clusters at increasing reporter concentrations in the background of input molecules at fixed concentrations to measure RNA sensor behaviour. Each cluster on the flow cell corresponded to a designed sequence. (vi) Binding data were quantified from multiple clusters for a single RNA sensor variant with the median fit shown. The data were then released to the Eterna community, and subsequent rounds of designs were solicited, or the next challenge was presented. **b**, The community was challenged with increasingly difficult design challenges, gradually building up to the complex TB sensor. In **a** and **b**, yellow and blue denote input conditions in which the sensor response (binding of the output ligand) is tighter or weaker than a specified threshold.

**Extended Data Fig. 1:**
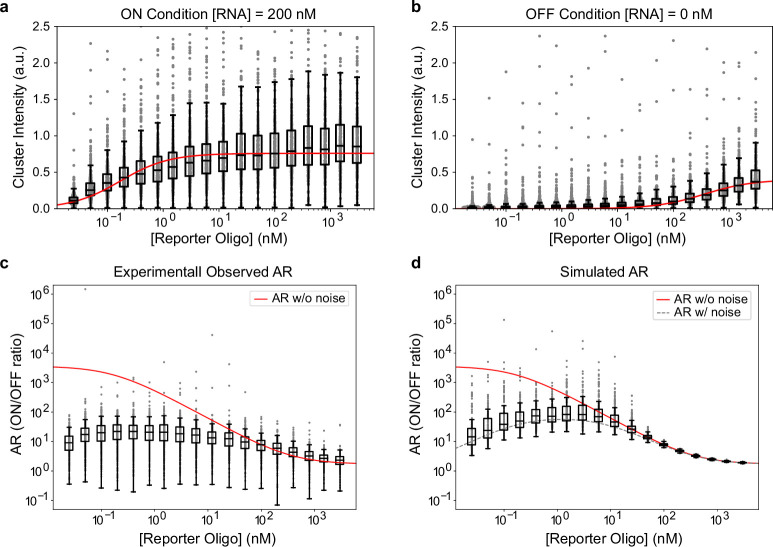
RNA-MaP measurements for the best OFF sensor (Omei In/Out C v16). The data correspond to the best OFF sensor from Fig. 1f. **a, b**) Cluster intensity of individual clusters measured on RNA-MaP for the RNA design in the ON and OFF conditions measured across different reporter oligo concentrations. The red curve represents the binding fits to the data. Data were collected from 308 clusters. **c**) Activation ratios (ON/OFF ratios) of the individual clusters at different reporter concentrations. The red curve represents the calculated qAR using the binding fits. **d**) Simulated data using the binding fits. 200 clusters were simulated by adding random Gaussian noise with variance 0.01. The red line corresponds to the qAR value with no noise (approaches 2050 as [Reporter Oligo] approaches 0). The grey line corresponds to the AR values measured if the noise is kept at a constant value of 0.01. The box and whisker plot plots the distribution of AR values measured with random Gaussian noise with variance 0.01. The centerline of the box plot indicates the median; the edges of the box correspond to the first and third quartiles; the whiskers extend to 1.5 times the interquartile range.

To tackle this problem, we developed a set of crowdsourcing challenges for citizen scientists engaging in the Eterna videogame^41^. Previous work demonstrated the ability of the Eterna community to solve RNA design tasks ranging from mRNA stabilization to the design of small-molecule-activated RNA sensors achieving thermodynamic optimality^13,14,42,43^. We presented increasingly difficult challenges on the Eterna platform to build up to the final goal of designing a complex, multi-input sensor (Fig. 1 and Supplementary Table 2). Within each challenge were a set of design puzzles, each representing a sub-problem of the overall challenge (Fig. 1a,ii). For example, in the pilot challenge of designing a single-input RNA sensor, one task was to design an ‘on’ sensor, and another task was to design an ‘off’ sensor. Both ‘on’ and ‘off’ sensors accomplish the same goal of distinguishing the presence of an input RNA. The Eterna interface was extended to allow players to design an RNA for more than one condition simultaneously (Fig. 1a,iii) and to provide estimates of the free energies of RNA folding in different conditions to give players rapid computational feedback^44–46^. Although imperfect, these free-energy estimates provided an approximation of the lowest-energy secondary structure for a given sequence to guide player designs. After player designs were collected, they were synthesized and displayed on an Illumina sequencing chip for RNA-MaP (RNA on a massively parallel array)^42^ characterization. These experiments quantified the behaviour of the player designs by measuring the affinity of the RNA sensor for a fluorescent output ligand across different input ligand conditions (Fig. 1a,iv–vi and Extended Data Fig. 1). The results were then returned to the community, who, with this experimental feedback, were tasked with improving on their previous results or tackling harder challenges (Fig. 1b).

The Eterna design challenges culminated with the OpenTB challenge, which asked players to design an RNA sensor that could detect if the three-gene TB-score exceeded the threshold that corresponds to active TB^36^. This biomarker signal takes as input the mRNA concentrations of the genes *GBP5*, *DUSP3* and *KLF2*. From each mRNA, we selected a short fragment to create simplified RNA inputs to the RNA sensor to be designed. The output fluorescence signal was generated by a fluorescently tagged RNA reporter engineered to bind to one of the possible states designed for the RNA sensor. Along with further testing with flow cytometry as an independent experimental readout, the results with the OpenTB sensor design provided a proof of concept for using RNA sensors to detect a complex diagnostic signature.

Through these progressively more difficult challenges, Eterna players developed and documented new and productive RNA design strategies. We incorporated player-derived strategies into a Monte Carlo Tree Search algorithm called ‘Nucleologic’ that allowed for automation of the RNA sensor design process. Using Nucleologic, we generated candidate designs to compute this complex TB-score biomarker output signal. After experimentally testing a few selected designs, we identified a successful RNA and DNA sensor for the TB-score with performance comparable to the top designs submitted by the players. Nucleologic, which harnesses the human-inspired heuristics used by Eterna players, thus shows promise in expediting the design of nucleic-acid sensors that can compute increasingly complex functions of multiple inputs.

## Results

### Pilot challenge—single-input RNA sensors

**Fig. 2:**
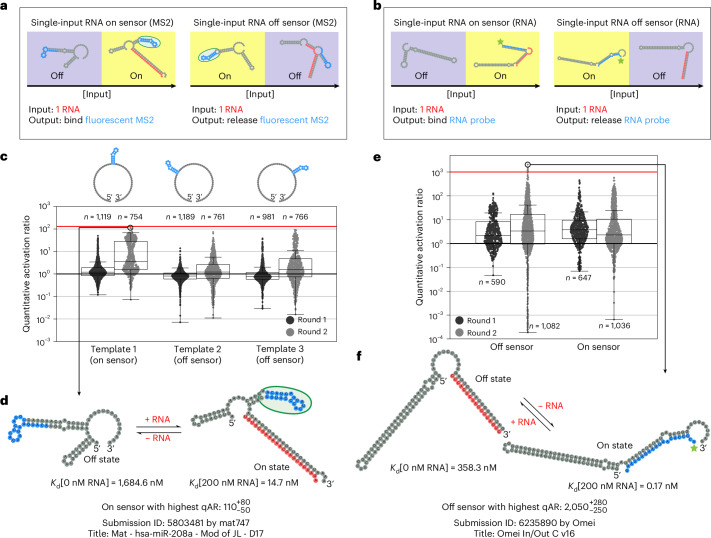
Pilot challenge—single-input RNA sensors. **a**,**b**, Players were tasked with designing the output to bind a fluorescent MS2 coat protein (**a**), as well as to bind or release a fluorescent RNA oligonucleotide reporter (**b**). **c**, Results from the puzzle in **a**. Top: players were constrained to three different templates for the design, with each template having a different MS2 hairpin location. Bottom: measured qARs across the architecture variants over two iterative rounds. **d**, Top player design of an RNA input/MS2 output on sensor puzzle. **e**, Puzzle **b** results. Measured qARs for the on and off sensors over two iterative rounds. **f**, Top player design of an RNA input/RNA output on sensor puzzle. In **c** and **e**, the red line is a soft upper limit for qARs that can be accurately measured. For the fluorescent MS2 sensor and fluorescent RNA sensor the values are 130 and 1,000, respectively. In **d** and **f**, RNA secondary structures were predicted using NUPACK. The centreline of the box plot indicates the median, the edges of the box correspond to the first and third quartiles, and the whiskers extend to 1.5 times the interquartile range.

As an initial step towards more complex RNA design challenges, Eterna players were presented with a pilot challenge to design single-input RNA sensors that responded to a separate RNA oligonucleotide. As a baseline, we sought to emulate or outperform earlier work that reported activation ratios as high as ~900 for sensors of RNA oligonucleotides (albeit in cellular contexts)^39,47,48^. To connect this pilot round with the eventual challenge of designing TB-score sensors, we explored two signalling mechanisms (that is, ‘outputs’). In the first mechanism, RNA sensors were turned on by having binding of input RNA lead to display of an MS2 virus stem loop RNA structure, which recruits a fluorescently labelled MS2 virus coat protein (Fig. 2a). This mechanism was chosen because Eterna players previously designed RNA sensors with MS2 protein binding as an output signal^42^. The input for these puzzles was a short RNA derived from hsa-miR-208a, a 22-nt microRNA (Supplementary Table 3) whose detection might aid in diagnosing cardiac hypertrophy^49^. Motivated by the final goal of developing sensors compatible with TB diagnosis, the second output mechanism involved hybridization of a fluorescent RNA reporter to a complementary sequence element in the sensor in the on state. This output mechanism allowed for incorporation in fluorescence-based or lateral flow-based diagnostic devices. Depending on the puzzle, players designed either an on sensor where the RNA sensor fluoresced when bound to the input, or an off sensor where the RNA sensor fluoresced when not bound to the input.

Based on our previous work demonstrating the importance of widely exploring the relative placement of functional elements to achieve success^14^, Eterna players were allowed to choose between three different templates that placed the MS2 aptamer sequence at different locations along the engineerable RNA molecule (Fig. 2c). For the designs using a RNA reporter output, the flexibility of the NUPACK prediction algorithm allowed the position of the RNA reporter binding site to be left unconstrained. All designs were limited to 85 nucleotides in length.

**Extended Data Fig. 2:**
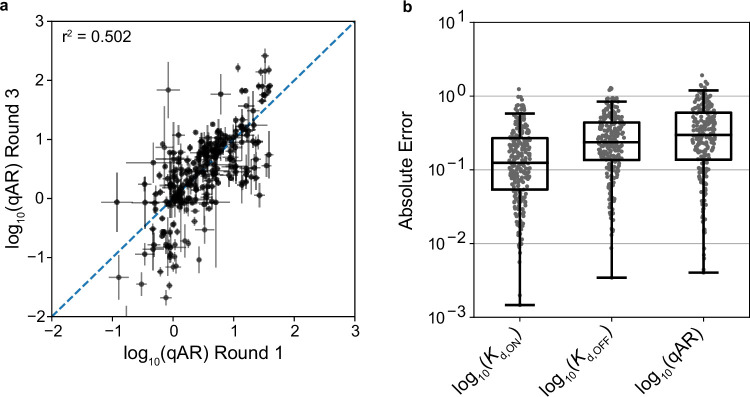
Reproducibility of quantitative activation ratio estimation with RNA-MaP experiments. In the Pilot Challenge (single-input RNA sensors), 245 designs for Template 1 Round 1 were experimentally re-evaluated in Round 3. **a**) Plot of the qAR measured in Round 1 against the qAR measured in Round 2. The centre of each point represents the reported log_10_(qAR) from the respective rounds. Error bars are from propagated standard errors of the mean for log_10_(qAR) used to fit the binding curves (n = 2). Pearson correlation coefficient is r^2^ = 0.502 and RMSD of log_10_(qAR) of 0.528. **b**) Box plot of the absolute error in the log_10_ values of *K*_d,ON_, *K*_d,OFF_, and qAR between Round 1 and Round 3. The median absolute errors are 1.33, 1.73, and 1.98 for *K*_d,ON_, *K*_d,OFF_, and qAR respectively. The centerline of the box plot indicates the median; the edges of the box correspond to the first and third quartiles; the whiskers extend to 1.5 times the interquartile range.

For sensor designs that used MS2 binding as the output signal, 3,369 and 2,319 player designs were characterized in rounds 1 and 2, respectively, split across three different template options (Fig. 2c). Round 1 presented players with only three templates, and round 2 introduced an additional template to increase the diversity of designs. The affinity of the RNA designs for the output molecules was then measured in the absence (0 nM) or presence (200 nM) of the input oligonucleotide. From the affinities for the output reporters, the quantitative activation ratio (qAR) was calculated for each design by dividing *K*_d_[0 nM input] by *K*_d_[200 nM input]. The qAR represented the fold change in the observed *K*_d_ of reporter binding between the off state (weak reporter affinity) and on state (strong reporter affinity). Thus, larger qAR values represented a sensor that better discriminated between the high and low input environments. Sensors selected by qAR retained their function in independent flow cytometry assays measured at a single output reported concentration^14^ (see Flow cytometer characterization of the best OpenTB sensor). We chose to use fold change in *K*_d_ to measure qAR as it gave an unbiased and high signal-to-noise measure of performance by taking into account overall RNA sensor behaviour across multiple output concentrations rather than at a single output concentration, where a direct measurement of activation ratio by RNA-MaP would have high noise. In the limit that the output ligand concentration approaches zero, the fold change in observed *K*_d_ is equal to the qAR (on signal/off signal ratio) values commonly reported in literature for switches (Methods)^50^; because we could measure *K*_d_ values between 1 nM and 1 μM, there was a soft upper qAR bound of 1,000 (Methods). RNA-MaP measurements of qAR up to this value were reproducible with a median error of less than twofold (Extended Data Fig. 2). Player designs improved dramatically between the two rounds (Fig. 2c). By refining previous submissions, players achieved designs with qAR values close to or above 100, with the top design achieving a qAR of ${110}_{-50}^{+80}$ (error values written in superscript and subscript correspond to one s.e., derived from fits to log *K*_d_, which gave a log_10_ qAR of 2.06 ± 0.22) (Fig. 2d). The functionality of the sensor relied on a mechanism similar to the approach for rationally designing a Spinach aptamer sensor reported in ref. ^51^. In the off state, the sensor was designed to have a misfolded MS2 hairpin structure as a result of base pairing with a complementary sequence. The on state was achieved when the binding energy of input RNA refolded the MS2 hairpin to its functional structure. A smaller set of designs were collected in a third round for template 3 (Supplementary Fig. 1) and the resulting qARs measured did not show an overall improvement from earlier rounds.

Motivated by the excellent performance in MS2-based output problems, 1,237 and 2,118 player designs were collected over two rounds for puzzles with RNA-based outputs more relevant for the TB-score sensors (Fig. 2e,f). Round 1 used an 18-nt input and a shorter 10-nt reporter oligonucleotide, and round 2 used a 17-nt input and a longer 20-nt reporter oligonucleotide. The reporter length was increased due to community feedback suggesting it was too difficult to design for a short output binding site (Supplementary Table 3). With a longer reporter oligonucleotide, players achieved qARs above 1,000, with a maximum observed qAR of $2,\!050_{-250}^{+280}$ (log_10_ qAR of 3.31 ± 0.06) (Fig. 2e,f) for off sensors. Based on the predicted NUPACK structure, the off sensor seemed to employ a toehold-mediated strand displacement reaction to displace the reporter RNA. The on sensors achieved slightly lower qAR values, with a maximum observed qAR of ${570}_{-49}^{+54}$ (log_10_ qAR of 2.76 ± 0.04). Although not as dramatic an improvement compared to the MS2-based output puzzles, players achieved better upper-quartile qAR values. Overall, throughout the pilot challenge, players achieved qARs at or beyond our experimental detection limits.

### Challenge 1—logic gates

**Fig. 3:**
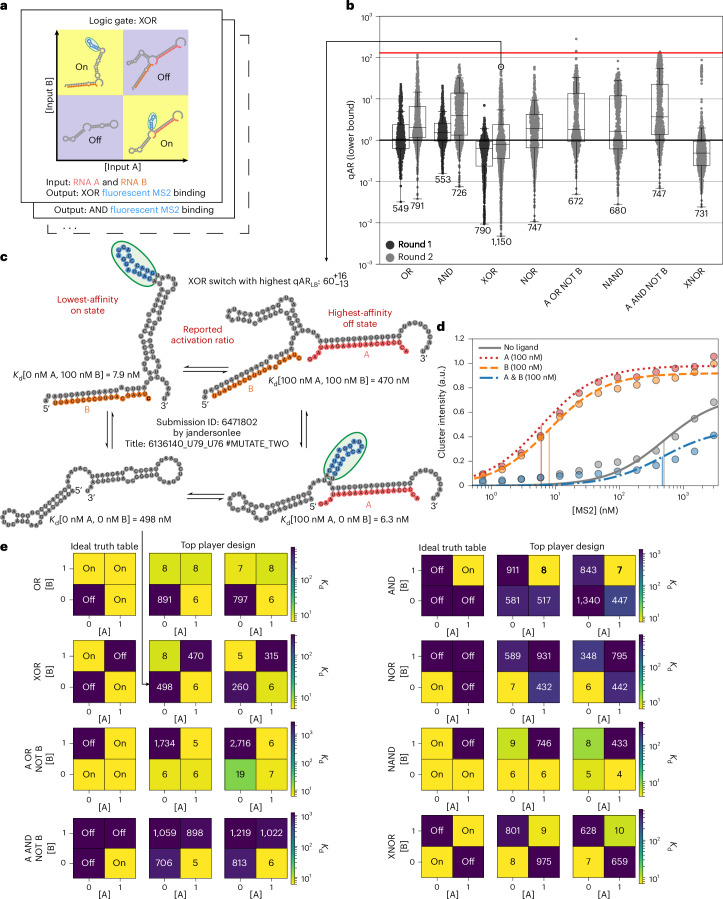
Challenge 1—logic gates. **a**, Each puzzle in this challenge corresponded to a different logic gate: AND, OR, NOR, XOR, NAND, XNOR, A OR NOT B and A AND NOT B. The two inputs are denoted RNA A and B, with the output binding a fluorescent MS2 coat protein. **b**, Measured qARs (lower bound) of the eight different logic gates over two iterative rounds. The red line is a soft upper limit for qARs that can be accurately measured (130). The centreline of the box plot indicates the median, the edges of the box correspond to the first and third quartiles, and the whiskers extend to 1.5 times the interquartile range. The number of samples in each box plot is shown. **c**, The top player design for the XOR puzzle. RNA secondary structures were predicted using NUPACK. **d**, RNA-MaP binding affinity measurements of the player design in **c**. Vertical lines correspond to *K*_d_ values. The points represent the median experimental fluorescence used to fit the binding curve. **e**, Ideal truth table and experimental results for each logic gate. The experimental data are from the top two designs from different Eterna players. *K*_d_ values are given in nM. 0 and 1 ‘binary’ values correspond to 0 and 100 nM concentrations of A and B.

We next challenged the Eterna community to generate designs of RNA Boolean logic gates (Fig. 3a). This first full-scale challenge (challenge 1) built on the pilot challenge by incorporating one additional input. The goal was to gradually provide the Eterna design community experience in designing more complex multi-input RNA sensors, as logic gates are, in their own right, useful tools in synthetic biology and nanotechnology and can, in principle, be chained together to execute complex computations.

All logic gates were designed to bind fluorescently tagged MS2 protein as the output signal. In response to player feedback, an MS2 ‘stamp’ tool was added to Eterna. This enabled players to easily place the MS2 hairpin RNA sequence anywhere they wanted within their design, giving players more flexibility in the design process compared to the pilot challenge. Each design was tested under four conditions corresponding to the four different binary inputs of the logic gate, where A and B represented the first and second bit, respectively. The binary input of 0 corresponded to 0 nM, a binary input of 1 corresponded to 100 nM, and the A and B sequences reused sequences of the input and output oligonucleotides used in the pilot challenge (Supplementary Table 3). With multiple on and off conditions, it was possible to calculate various qAR values depending on the on and off condition chosen. To evaluate the performance for each design, a conservative ‘quantitative activation ratio lower bound’ (qAR_LB_) was computed by calculating the ratio of the *K*_d_ values for the highest-affinity off state with the lowest-affinity on state, where the off and on states were the conditions in which an ideal logic gate would return a 0 or 1, respectively. Earlier work on single-molecule logic gates achieved ARs as high as 21 for OR, AND and NOR gates^9^ and above 100 when coupled to additional components such as DNA polymerases^52^. qAR_LB_ values for XOR and XNOR gates from RNA, protein and DNA systems have remained below 10 (refs. ^9,25,53–58^). We sought to determine whether similar or better values might be achievable with single-molecule RNA sensors designed on Eterna.

In round 1 of the logic gate challenge, Eterna players were tasked with designing OR, AND and XOR gates. These design tasks were expanded in the second round to include NOR, A OR NOT B, NAND, A AND NOT B and XNOR for a total of eight logic gates, which covered all possible truth tables (up to permutation of A and B; Fig. 3b). During the first round, the best of 1,892 player designs achieved qAR_LB_ values near 20, and in the second round, the best of 6,244 player designs achieved qAR_LB_ values greater than 100. Of all the logic gates, the XOR gate was the most difficult for Eterna players to design, with the majority of round 1 designs having qAR_LB_ < 1. Nevertheless, after round 2, the top player designs for XOR and XNOR achieved a qAR_LB_ of ${60}_{-13}^{+16}$ (log_10_ qAR_LB_ = 1.78 ± 0.10) and ${87}_{-21}^{+28}$ (log_10_ qAR_LB_ = 1.94 ± 0.12), respectively (Fig. 3c,d). Many successful XOR and XNOR gates in round 2 (notably the distinct high qAR_LB_ population of XNOR in Fig. 3b) were designed by modifying sequences for round 1 AND and OR gates that experimentally gave hints of XOR or XNOR activity (Supplementary Table 4). This result suggested that carrying out multiple simultaneous challenges on Eterna could lead to a productive cross-fertilization of solutions. For all eight logic gates, the top player designs successfully approximated the logic gate outputs (Fig. 3e).

### Challenge 2—ratio sensor

**Fig. 4:**
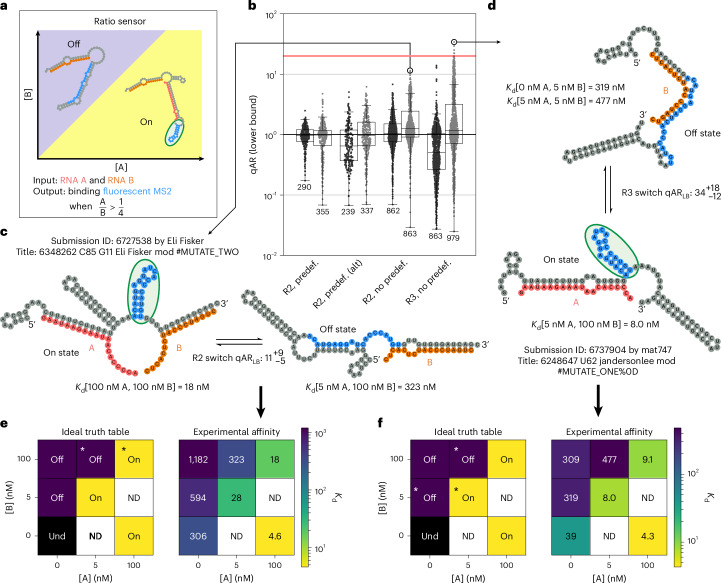
Challenge 2—ratio sensor. **a**, Players were tasked with designing an RNA sensor that turned on when [A]/[B] > ¼, with binding of a fluorescent MS2 coat protein as the output signal. **b**, Measured qARs (lower bound) across different puzzles and rounds. The red line is the theoretical maximum for a qAR_LB_ of 20. The centreline of the box plot indicates the median, the edges of the box correspond to the first and third quartiles, and the whiskers extend to 1.5 times the interquartile range. The number of samples in each box plot is shown. **c**, NUPACK structure of the top player design from the R2 puzzles. **d**, NUPACK structure of the top player design for the R3 puzzle. **e**, Ideal truth table and player design truth table for **d**. **f**, Ideal truth table and player design truth table for **c**. Asterisks in the ideal truth tables of **e** and **f** indicate the in silico conditions simulated in Eterna puzzles. Und, undefined; ND, not determined. Undefined conditions do not have a valid [A]/[B] value. Not determined conditions were not experimentally measured.

With the aim of building up to the final challenge of sensing the three-input TB-score, which involved multiplication and division of the concentrations of input RNAs, we challenged the Eterna community to design an RNA sensor capable of computing the ratio of the concentrations of two input molecules. Specifically, players were tasked with designing an RNA sensor to detect whether the ratio of two input RNAs A and B was greater than 1/4 (Fig. 4a). The key idea behind this challenge was driven by a mathematical form guaranteed by equilibrium thermodynamics: if a sensor could be designed to have two mutually exclusive states, one state binding A (but not B) and another state binding B (but not A), the relative population of the states would be proportional to [A]/[B] (Methods). If the relative energetics of the two states could be set to achieve equal populations at [A]/[B] = ¼ and to favour the A-binding state under high [A]/[B] conditions and the B binding state at low [A]/[B] conditions, the sensor would respond to the desired ratio.

Several different puzzles were created to explore which in silico constraints might yield the most performant ratio sensors (Fig. 4b). The ‘R2’ puzzles tasked players to design a sensor that exhibited the desired behaviour at two different simulated input concentrations, and an ‘R3’ puzzle tasked players to design a sensor that exhibited the desired behaviour at three different input concentration combinations. The simulated design conditions for the R2 puzzle were 5 nM A & 100 nM B and 100 nM A & B, corresponding to A/B of 1/20 and 1. Design conditions for the R3 puzzle only shared the condition 5 nM A & 100 nM B while also having 0 nM A & 5 nM B and 5 nm A and 5 nM B, corresponding to A/B of 0 and 1, respectively. The hypothesis for R3 was that by having an additional off condition constraining the puzzles, the submitted designs would be more robust across all A and B concentrations tested experimentally. The A/B ratios of 1/20 and 1 were chosen to bracket the 1/4 ratio relatively evenly. (Measuring the *K*_d_ of the designs exactly at the 1/4 ratio would not measure whether a sensor accurately distinguished conditions above or below the ratio.)

Furthermore, we sought to understand whether allowing players to each explore a wide set of binding sites for A and B might be better than focusing the Eterna community’s attention on specific sets of predefined binding sites. We deployed three sets of R2 puzzles to test this idea (‘predef.’, ‘predef. alt’ and ‘no predef.’; Fig. 4b). Finally, to test the generality of player design strategies, we used different A and B sequences here than in challenge 1, using input and output sequences from different rounds of the pilot challenge (Supplementary Table 3).

The designs were experimentally tested across a total of seven conditions, expanding the two or three conditions presented to Eterna players (Fig. 4f,g), over two rounds with 2,254 and 2,534 designs tested, respectively. qAR_LB_ was again computed as a worst-case ratio of the highest-affinity off state with the lowest-affinity on state with respect to all seven test conditions; the best possible theoretical qAR_LB_ value achievable was 20 (Methods). Across the two design rounds, many player designs achieved qAR_LB_ values greater than 10. The top player design from R2 puzzles gave a qAR_LB_ of ${11}_{-5}^{+9}$ (log_10_ qAR_LB_ = 1.06 ± 0.26; Fig. 4c). These top designs came from the R2 puzzle without predefined binding sites, which overall led to better qAR_LB_ than the two puzzles that constrained designs with predefined A and B binding sites. This result supported the principle that wide varieties of design patterns should be explored during the design process, as also supported by our earlier work on small-molecule sensors^14^ as well as the previous challenges in this study (Figs. 2 and 3). Across the two rounds the upper-quartile qAR increased between the rounds for both R2 and R3 puzzles; however, the best overall submissions were from the R3 puzzle, supporting our hypothesis that an additional simulated design condition would favour better solutions (Fig. 4b). The top R3 designs achieved experimental truth tables similar to the ideal truth table across all conditions and a top qAR_LB_ of ${34}_{-12}^{+18}$ (log_10_ qAR_LB_ = 1.53 ± 0.18; Fig. 4e,f). Interestingly, many designs from this round came from strategies developed by Eterna players in the previous challenges and developed further in the following challenge (Supplementary Table 4).

### Challenge 3—openTB sensors

**Fig. 5:**
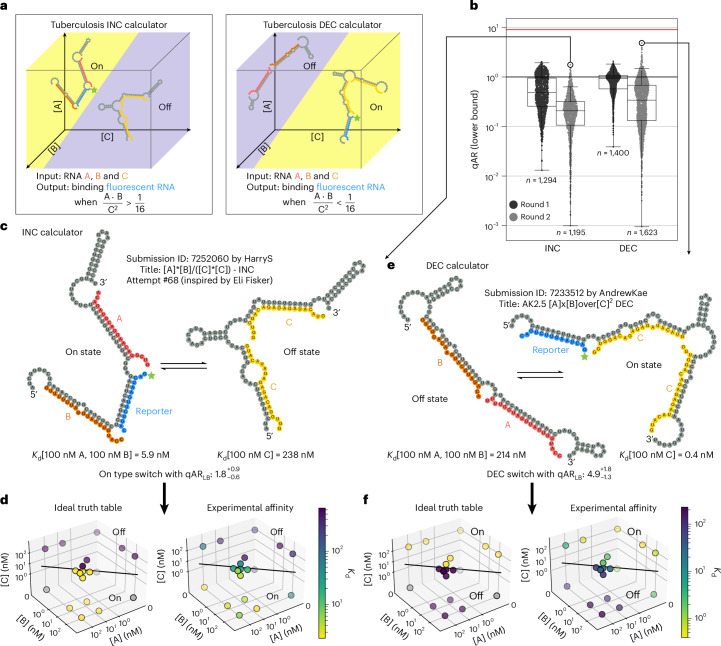
Challenge 3—openTB sensors. **a**, Players were tasked with designing an ‘INC’ TB-score sensor with fluorescent RNA reporter binding when [A][B]/[C]^2^ > 1/16 (left), or a ‘DEC’ sensor binding the fluorescent RNA reporter when [A][B]/[C]^2^ < 1/16 (right). **b**, Measured qARs (lower bound) for INC and DEC designs across two iterative rounds. The red line is the theoretical maximum for a qAR_LB_ of 9. The centreline of the box plot indicates the median, the edges of the box correspond to the first and third quartiles, and the whiskers extend to 1.5 times the interquartile range. **c**, NUPACK structure of the best INC player design from round 2. **d**, Ideal truth table and experimental truth table for the INC design in **c**. **e**, NUPACK structure of the best DEC design, ‘AK2.5’, from round 2. **f**, Ideal truth table and experimental truth table for the DEC design in **e**. In **d** and **f**, the separatrix plane for [A][B]/[C]^2^ = 1/16 appears as a line due to the chosen view of the three-dimensional (3D) plot, and grey points in the ideal truth tables are undefined with respect to the three-gene TB-score.

After successfully creating two input sensors for logic gates and a ratio function, we challenged Eterna players to design an RNA sensor to compute the three-gene TB-score for active TB. In clinical studies, active TB correlates with the expression of three gene mRNAs (*GBP5*, *DUSP3* and *KLF2*; hereafter A, B and C) in which [A][B]/[C]^2^ is greater than or equal to 1/16 (Methods). In the OpenTB challenge, we envisioned that both positive and negative sensors for this signature would be clinically useful either separately or in combination for a more robust diagnostic. We therefore challenged the Eterna community to design RNA sensors to address the full TB-score calculation: [A][B]/[C]^2^ > 1/16 (‘INC’ to detect when the TB-score increased above the threshold) or [A][B]/[C]^2^ < 1/16 (‘DEC’ to detect when the TB-score decreased below the threshold) (Fig. 5a). Additional puzzles were presented to let players experiment with designing simpler intermediate RNA sensors related to this final sensor (Supplementary Table 2 and Supplementary Fig. 2).

**Extended Data Fig. 3:**
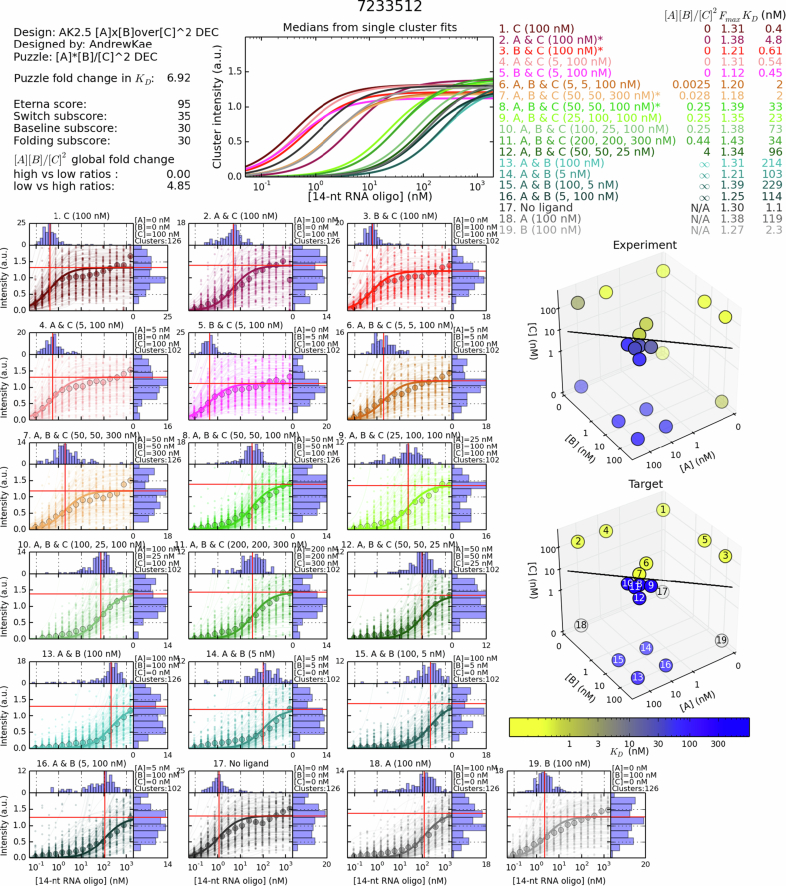
Example of RNA-MaP data summary plot made available to Eterna participants for OpenTB challenge. These data are for Round 2 of Challenge 3: OpenTB sensor, for design AK2.5 submitted by Andrew Kae. The affinity of the designed RNA to the reporter RNA is computed at various conditions.

Players were tasked with designing RNA sensors that exhibited two mutually exclusive states, one state binding a single copy of A and B but no C strands, and the other state binding two copies of C but no A or B. The puzzles involved four simulated design conditions: 100 nM A & 100 nM C, 100 nM B & 100 nM C, 50 nM A&B & 300 nM C, 50 nM A&B & 100 nM C. For all simulated conditions, 25 nM of the RNA output reporter R (initially chosen to be the same as previous challenge 2) was present. These four conditions corresponded to values of the TB-score ratio equal to 0, 0, 1/36 and 1/4, respectively. The first two conditions that kept A or B as 0 nM ensured player designs could properly bind A and B and treated them as functionally interchangeable, as they were both multiplied on the numerator of the TB-score function. This helped to avoid designing an [A]/[C] or [B]/[C] sensor. Having the sensor switch its favoured state between the last two conditions ensured that binding of C competed with the binding of A and B; requiring two copies of C bind cooperatively ensured that the sensor’s output involved [C]^2^ in the denominator. Altogether, these four simulated solution conditions established strong boundary conditions for player designs. The standard formula for chemical equilibrium then required that the ratio of the sensor’s two states be proportional to [A][B]/[C]^2^ and helped ensure that the sensor would sustain its desired behaviour at other concentrations of A, B and C (Methods), an assumption we also tested experimentally below. Throughout the OpenTB challenge, players also had access to simulation plots that provided in silico NUPACK predictions at a broad suite of input concentrations ranging from femtomolar to millimolar to help players refine sequences beyond the four conditions presented in Eterna (Supplementary Fig. 3). In addition, after each round of experiments, the Eterna community was given a detailed PDF summary of each design, showing the binding curves of the reporter RNA under all conditions tested in RNA-MaP (an example is shown in Extended Data Fig. 3).

For round 2, the 20-nt input sequences were changed after players selected new fragments from the TB-score genes *GBP5*, *DUSP3* and *KLF2* based on BLAST analysis (Supplementary Table 3). Also, based on player recommendation, the RNA output reporter was lengthened from 10-nt to 14-nt to create more binding potential. Finally, a ‘Freeze Mode’ was added to Eterna at player request which allowed players to modify their RNA sequence without triggering NUPACK computation, which required over a minute on most player computers.

A total of 2,694 round 1 designs were tested at 13 different combinations of A, B and C input concentrations, and 2,818 round 2 designs were tested against 19 different combinations to more fully explore the phase space of behaviours (Supplementary Table 5). Under each of these conditions, full binding curves were derived measuring the effective *K*_d_ of the output signalling reporter to the RNA molecule. For each design, qAR_LB_ was computed as a ‘worst-case’ metric, similarly to the previous challenges, as the ratio of the highest-affinity off state with the lowest-affinity on state with respect to all experimentally tested conditions. A perfect sensor would achieve a qAR_LB_ of (1/4)/(1/36) = 9. The best INC design achieved a qAR_LB_ of ${1.8}_{-0.6}^{+0.9}$ (log_10_ qAR_LB_ = 0.25 ± 0.18; Fig. 5c). Worse INC designs were observed in round 2 than in round 1. This decrease in qAR_LB_ may have been due to the increased number of conditions tested, which increased the likelihood of observing errors in the sensor that were sensitively captured by qAR_LB_ (Fig. 5b). The DEC design challenges were more successful, with player designs such as AK2.5 reaching a qAR_LB_ of ${4.9}_{-1.3}^{+1.8}$ (log_10_ qAR_LB_ = 0.69 ± 0.13; Fig. 5b,d). These values were affected by experimental uncertainties in some of the 19 test conditions, probably leading to artificial suppression of qAR_LB_. When focusing specifically on the four A, B and C conditions that were simulated in the Eterna puzzle, this same AK2.5 sensor gave a qAR_LB*_ of ${11.5}_{-2.5}^{+3.3}$ (log_10_ qAR_LB*_ = 1.06 ± 0.11; Supplementary Fig. 4a), agreeing with the maximum value of 9. The asterisk is used to denote that the metric is only computed using a subset of the experimental data. To test whether the improvements might be due to the updated input and output RNA sequences, round 3 repeated the challenges but reverted these sequences to the original round 1 sequences (Supplementary Table 2); indeed, this round led to a sensor performance as poor as in round 1 (Supplementary Fig. 4b). For further independent evaluation across a broader range of input conditions, the top INC and DEC designs were carried forward to flow cytometry measurements, as described next.

### Flow cytometer characterization of the best OpenTB sensor

**Fig. 6:**
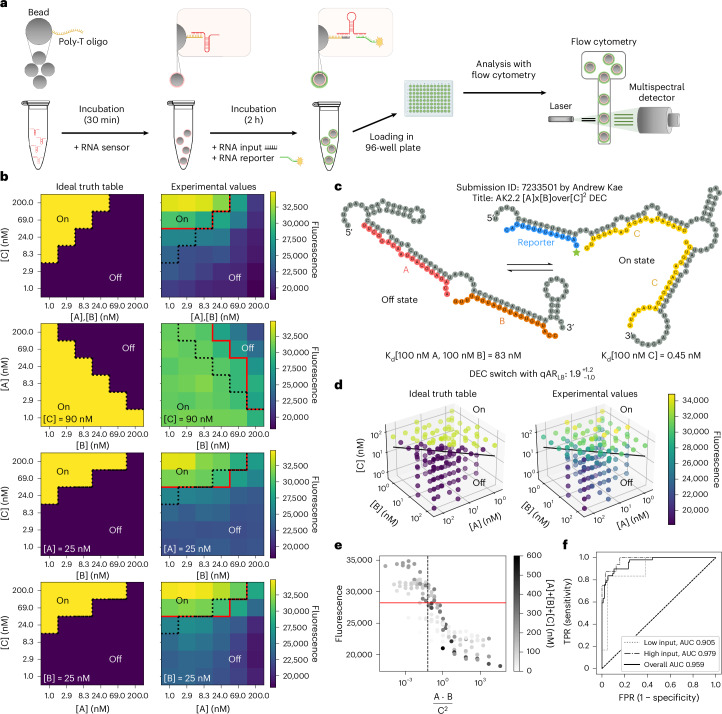
Flow cytometry evaluation of the top performing DEC TB sensor, AK2.2. **a**, Experimental pipeline for testing an RNA sensor with flow cytometry. Beads with 1-μm diameter, coated in (dT)_25_, were incubated with the RNA sensor and then washed to remove unbound RNA. The beads were then incubated at various conditions and fluorescence measured in the flow cytometer. **b**, Ideal and experimental truth tables across various conditions for design AK2.2. **c**, NUPACK structure of AK2.2. **d**, 3D scatter plot of the ideal truth table (left) and experimental values (right) with the separatrix plane at [A][B]/[C]^2^ = 1/16. **e**, Distribution of the measured fluorescence of design AK2.2 across different values of [A][B]/[C]^2^. **f**, ROC curve of design AK2.2. ‘Low input’ and ‘high input’ correspond to computing AUROC for data points where [A] + [B] + [C] values are <75 nM and >170 nM, respectively. TPR, true positive rate; FPR, false positive rate. In **b** and **e** the dotted black line is the TB diagnostic threshold at [A][B]/[C]^2^ = 1/16. The red line represents the optimal diagnostic threshold for the sensor.

**Extended Data Fig. 4:**
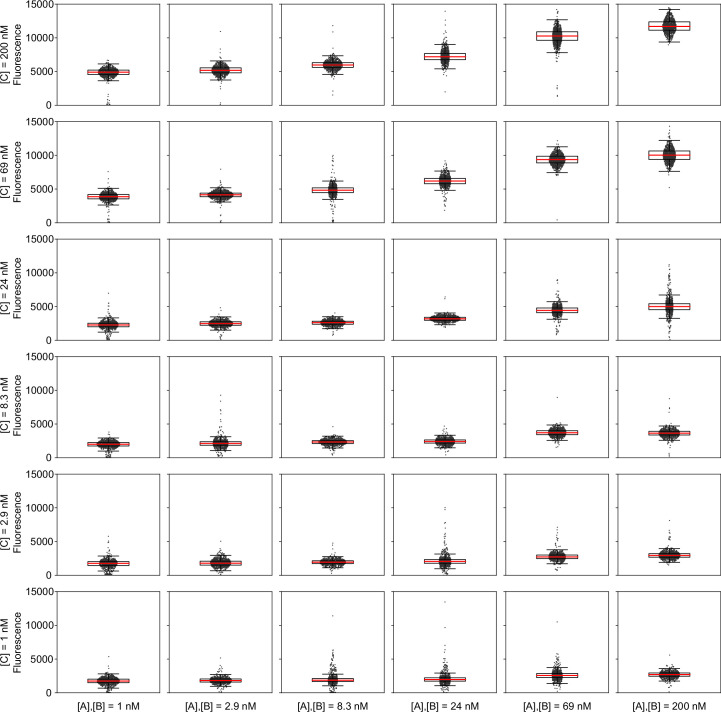
Raw flow cytometry data of MCTS_20 (INC). The data correspond to the raw data for Extended Data Fig. 5 for MCTS_20 (INC). Each row and column corresponds to a different [C] and [A],[B] concentration respectively. [R] was kept at 30 nM. Each tile shows the box plot with the red line indicating the median value. The scatter plot behind the box plot shows the distribution of the fluorescence measured. The distance of the scatter points from the centre indicates the density based on a Gaussian kernel density estimation of the fluorescence. A subset of only 1,000 events were plotted out of the 10,000 total events for visibility. The centerline of the box plot indicates the median; the edges of the box correspond to the first and third quartiles; the whiskers extend to 1.5 times the interquartile range.

**Extended Data Fig. 5:**
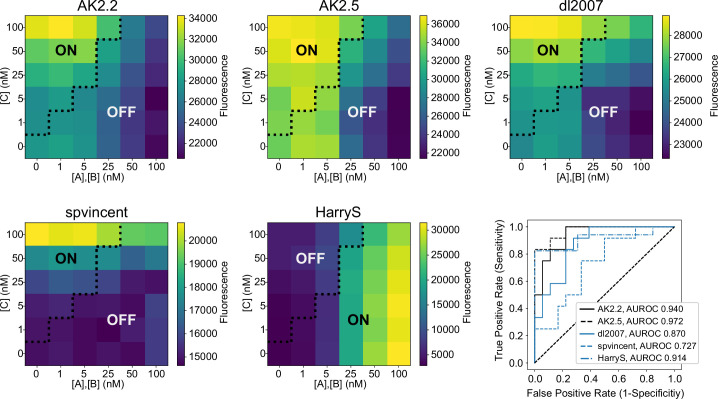
Flow cytometry characterization of top player designs from Round 2 of Challenge 3: OpenTB calculators. Five different player designs were tested in 36 different conditions as shown in the heatmap. For all conditions, [R] was kept at 30 nM. The titles of the heatmaps are the player names except AK2.2 and AK2.5 which are designs from Eterna player Andrew Kae. From left to right and top to bottom the 5 designs are from (1) player: AndrewKae design ID: 7233501 design name: ‘AK2.2 [A]x[B]over[C]^2 DEC’, (2) player: AndrewKae design ID: 7233512 design name: ‘AK2.5 [A]x[B]over[C]^2 DEC’, (3) player: dl2007 design ID: 7172797 design name: ‘A*B/C^2 DEC 8 9’, player: spvincent design ID: 7230841 design name: ‘A*B/C*C Dec Mod20’, player: HarryS design ID: 7252060 design name: ‘[A]*[B]/([C]*[C]) - INC Attempt #68 (Inspired by Eli Fisker)’. Designs 1-4 are DEC sensors and design 5 is an INC sensor. Receiver operating characteristic (ROC) curves of all five designs are shown on the bottom right.

As an independent and more thorough test of functional accuracy, the top-scoring player designs from the OpenTB challenge were selected for characterization across a wide range of input conditions using flow cytometry (Fig. 6a). To ensure equilibrium, all samples were incubated for 2 h before flow cytometry analysis. This orthogonal measurement of fluorescence response was achieved by first attaching the sensors to the surface of magnetic beads and then incubating with a fluorescent RNA reporter (30 nM) at different input concentrations of A, B and C input RNA molecules. For each condition, the fluorescent signal was taken as the median of 10,000 flow cytometry events (Extended Data Fig. 4 and Methods). The sequences of the input oligonucleotides A, B and C were the same from round 2 of the OpenTB challenge. Because some of the [A][B]/[C]^2^ conditions in the flow cytometry experiments approached closely to the separatrix 1/16, the best achievable qAR_LB_ metric would approach 1 even for perfect designs and not be useful for ranking. We therefore ranked designs by a metric more common in diagnostic characterization, the area under the receiver operating characteristic curve (AUROC), which varied the output fluorescence threshold and computed specificity and sensitivity. In agreement with RNA-MaP measurements, DEC sensors outperformed INC sensors in flow cytometry. A particularly notable DEC sensor with excellent performance at low concentrations of A, B and C input RNA molecules was a close homologue of AK2.5 named AK2.2 (Fig. 6b,c and Supplementary Fig. 5), which achieved an AUROC of 0.935 under these test conditions (Extended Data Fig. 5). AK2.2 was carried forward for more detailed testing across 144 different artificially prepared conditions (Fig. 6b). Overall, AK2.2 achieved an AUROC of 0.959 across these conditions (95% confidence interval (CI), 0.930–0.988; Fig. 6d). Across the entire input-space volume that was experimentally tested, AK2.2 was able to properly categorize points as positive or negative with a specificity of 89.6% and a sensitivity of 89.5% at a threshold chosen to maximize the sum of the specificity and sensitivity (Fig. 6e, red line). The sensor performance was expected to be best at the highest input oligonucleotide concentrations, where the assumption that either A and B or two copies of C bound would best hold, without states with fewer input oligonucleotides bound. Indeed, at concentrations of [A] + [B] + [C] > 170 nM, the sensor performance was visually clearer (Fig. 6d, black points) and AUROC increased to 0.979 (95% CI, 0.948–1.0) for A, B and C in this high input concentration range.

### Computational design of RNA and DNA sensors for the TB-score

Starting with early design rounds on two-input Boolean logic gates, Eterna players derived a sequence-independent heuristic for sensor design named the ‘domain matching secondary structure design’ (DMSSD; Supplementary Table 4). DMSSD deployed a constraint-driven method using predefined domains to design secondary structures. The method ‘chunked’ the RNA sequence into domains, where each domain was associated with another complementary or near-complementary domain in the sequence to facilitate intramolecular interaction via secondary structure. This method was itself an improvement on a common player strategy called kernel attractors (Supplementary Table 4), where a domain in the designed sequence was ‘attracted’ to the output and input oligonucleotides due to complementary sequences. Although the kernel attractor strategy required fine-tuning the interaction strength of one domain with two or more sequences, DMSSD simplified the process by focusing on designing interactions between a domain and its complementary domain and relying on mutual exclusion of interleaved stems in the RNA secondary structure.

**Fig. 7:**
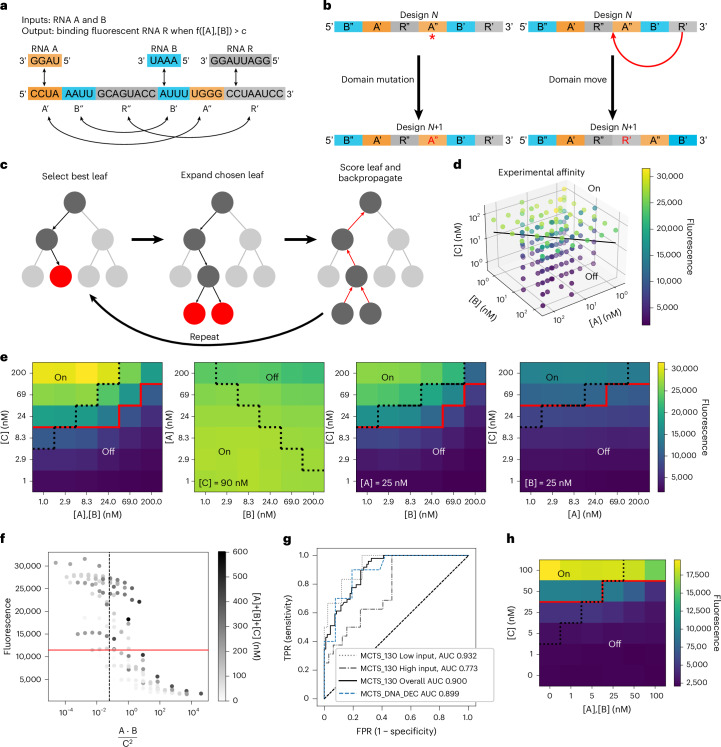
Nucleologic uses Monte Carlo Tree Search to automate the design of complex nucleic-acid sensors. **a**, Example demonstrating the use of DMSSD for a sensor with two input RNAs (A, B) and one output RNA reporter (R). Arrows indicate complementary domains. **b**, Nucleologic can either perform a ‘domain mutation’, which creates a random mutation, or a ‘domain move’, which moves a domain. **c**, The Nucleologic algorithm consists of three steps: selecting the best leaf node based on the UCT score, expanding the leaf node by generating children using the modifications in **b**, and scoring the leaf and backpropagating the value, updating the UCT score of all nodes in the path back to the root node. **d**, 3D scatter plot of the experimental values with the separatrix plane at [A][B]/[C]^2^ = 1/16 for the best Nucleologic design HP_MCTS_130. **e**, Experimental truth tables measured across various conditions. **f**, Distribution of the measured fluorescence of design HP_MCTS_130 across different values of [A][B]/[C]^2^. **g**, ROC curve of design HP_MCTS_130. ‘Low input’ and ‘high input’ correspond to computing AUROC for data points where [A] + [B] + [C] values are <75 nM and >170 nM, respectively. The ROC curve for MCTS_DNA_DEC (blue) is shown for comparison. **h**, MCTS_DNA_DEC experimental truth table for A,B (at equal concentrations) against C. In **e** and **f**, the dotted black line is the TB diagnostic threshold at [A][B]/[C]^2^ = 1/16. The red line represents the optimal diagnostic threshold for the sensor.

Figure 7a shows an example of employing DMSSD to design an RNA sensor to detect two input RNAs, A and B, along with an output RNA R (Methods). The RNA sensor sequence was first partitioned into several domains: A′, A′′, B′, B′′, R′ and R′′. Domains A′ and B′ were designated to harbour complementary sequences to inputs A and B, respectively. For the output, domain R′ was complementary to a fluorescently tagged RNA reporter. In addition to these domains, extra domains were added that were complementary to an existing domain, such as A′′, which was complementary to A′. This allowed for complex secondary structure rearrangement in the absence and presence of the input ligands due to the network of complementary regions in the RNA and interweaving of the domain locations. In particular, the R′–R′′ stem cannot occur simultaneously with the A′–A′′ or B′–B′′ pairing. By altering the complementarity between domains and the order of domains, it was possible to alter the RNA sensor’s response to fit the boundary conditions of a user-defined function f([A],[B]).

Inspired by these design principles from Eterna players (Supplementary Appendix: Eterna player resources, within Supplementary Data file), we created Nucleologic, a Monte Carlo Tree Search algorithm for automating the design of complex RNA sensors (Fig. 7b,c). The input to Nucleologic was the set of domains that made up the single-strand nucleic-acid sequence as well as the order of the domains. Typically, a domain with a sequence complementary to the input and output RNAs was included with extra domains that were filled with Ns. During sequence optimization, Nucleologic performed two types of move on the sequence: domain mutation, which mutated the sequence within the domain, or domain move, which moved the location of a domain (Methods). The choices of which intermediate solutions to carry forward were made based on Monte Carlo Tree Search, a classic automated game playing strategy^59–61^. By posing the problem as a game with DMSSD-inspired moves, Nucleologic optimized the simulated AR and fold change between the on and off states defined by the user calculated using NUPACK.

**Extended Data Fig. 6:**
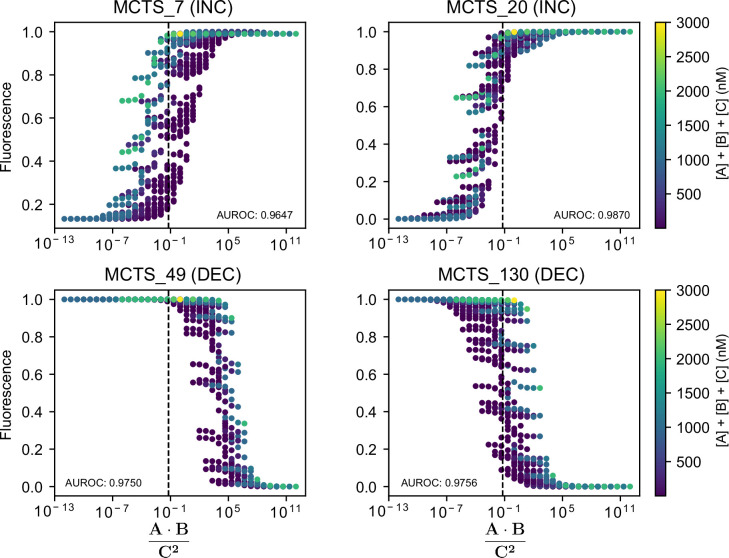
*In silico* screening of Nucleologic designs. The top two DEC and INC designs were chosen based on an *in silico* screen using NUPACK. The sequences were simulated at different concentrations of the inputs [A], [B], and [C] while keeping [R] at 30 nM. The concentrations consisted of 10 values evenly spaced on the log scale from 1 pM to 1 μM for a total of 1000 conditions. Using the NUPACK calculated values, a logistic regression model was fitted to compute the AUROC value. The dashed vertical line indicates where [A][B]/[C]^2^ = 1/16.

**Extended Data Fig. 7:**
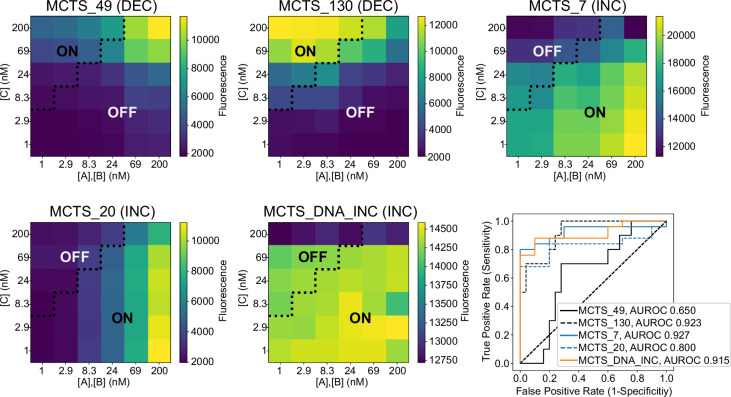
Flow cytometry characterization of other Nucleologic designs. The top two DEC and INC RNA designs as well as an INC DNA design from Nuclelogic were tested in 36 different conditions as shown in the heatmap. For all conditions [R] was kept at 30 nM. The title of the heatmap is the design name and the type of sensor, INC or DEC, is in parentheses. Receiver operating characteristic (ROC) curves of all designs are shown on the bottom right.

**Extended Data Fig. 8:**
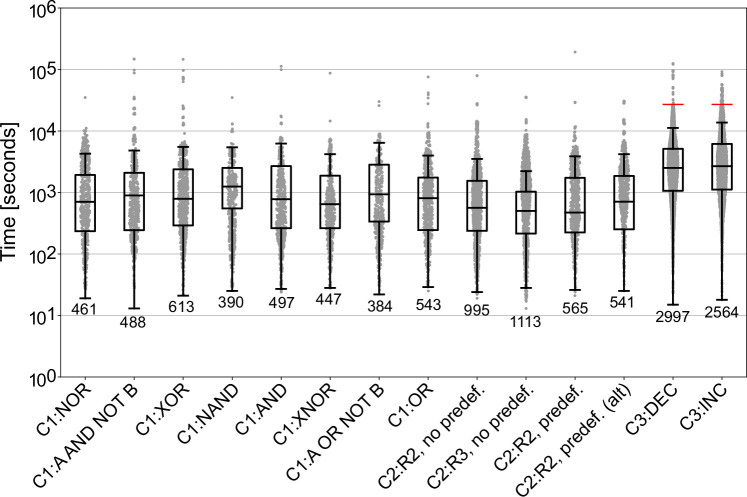
Time to submit solutions on Eterna. The distribution of the time it took for Eterna players to submit solutions from Challenge 1 to Challenge 3. C1, C2, and C3 denote Challenge 1, Challenge 2, and Challenge 3 respectively. The name after the colon indicates the puzzle the players submitted the solution to. The metric only measures the time Eterna players spent on the user interface. It does not reflect the extra time spent outside. The red horizontal line is the time it takes to run Nucleologic for Challenge 3 (7.5 h). The centerline of the box plot indicates the median; the edges of the box correspond to the first and third quartiles; the whiskers extend to 1.5 times the interquartile range.

Using Nucleologic, we computationally designed several hundred DEC and INC RNA sensors for the TB-score. The designs from Nucleologic were simulated under 1,000 conditions across different [A], [B] and [C] concentrations with NUPACK (Extended Data Fig. 6), and the top four designs based on predicted AUROC were chosen for experimental validation by flow cytometry (Methods and Extended Data Fig. 7). The best performing design, HP_MCTS_130, was further tested across 144 input conditions (Fig. 7d). Although Nucleologic took longer than players to develop designs for challenge 3—taking a median of 7.5 h compared to players’ 43 min (or upper quartile of 1.5 h) (Extended Data Fig. 8)—it scaled efficiently with additional computing resources. These time estimates only measured the time Eterna players spent on the user interface and did not reflect the extra time spent outside Eterna, as was the case for top designs such as AK2.2 or AK2.5 (Figs. 5 and 6), which involved preparation of handwritten designs by player Andrew Kae.

Although HP_MCTS_130 did not perform as well as AK2.2, it had a lower baseline fluorescence and could accurately discriminate between the on and off states when [C] was low (Fig. 7e,f). Across the 144 points in the input-space volume that were experimentally tested, HP_MCTS_130 was able to properly categorize points as on or off with a specificity of 78.5% and a sensitivity of 69.5%, and AUROC of 0.900 (95% CI 0.852–0.947). In contrast to the Eterna design AK2.2, this Nucleologic RNA sensor achieved better performance at lower input concentrations: at [A] + [B] + [C] of 70 nM or lower, HP_MCTS_130 gave an improved AUROC of 0.932 (95% CI 0.839–1.0).

To assess the generality of Nucleologic, we then tested two sensors for computing the TB-score based on DNA instead of RNA (Extended Data Fig. 7). Experimental results from flow cytometry demonstrated that one of these, MCTS_DNA_DEC, switched appropriately when A and B were varied against C with a specificity of 78.47% and sensitivity of 69.47% (Fig. 7f,g). The DNA sensor achieved an AUROC of 0.899 (95% CI 0.852–0.947; Fig. 7f).

## Discussion

RNA and DNA are ideal substrates for designing function approximators due to the ease of large-scale nucleic-acid synthesis, availability of computational modelling methods for predicting nucleic-acid structure, and increasing throughput of experimental evaluation methods. Nevertheless, the complexity of functions achieved by single-molecule nucleic-acid sensors has been limited. Here, starting from simple single-input RNA sensors, a community of Eterna citizen scientists successfully designed more complex multi-input RNA sensors, including all possible logic gate sensors, two-input ratio sensors and, in the OpenTB challenge, sensors of the three-gene TB-score [*GBP5*][*DUSP3*]/[*KLF2*]^2^ for diagnosing active TB. In all challenges, the qARs of Eterna sensors approached the limits of our experimental assays or the theoretical upper bound for the sensors. The performance of Eterna sensors was particularly striking given their compact lengths—the 85 nucleotides of the TB-score sensors presented binding sites for the 14-nt reporter as well as *GBP5*, *DUSP3* and two *KLF2* segments (20-nt each). In addition, the sensors were robust to experimental conditions, performing similarly on both repurposed Illumina sequencers (for RNA-MaP) and on oligo-coated beads for more complete characterization by flow cytometry. Driving the success of these compact, near-optimal designs were Eterna player strategies such as the kernel attractor and DMSSD heuristics. By incorporating these strategies into a Monte Carlo Tree Search, we created Nucleologic to automate the design of complex nucleic-acid sensors. Our work demonstrates the continuing utility of citizen science-based crowdsourcing integrated with iterative, high-throughput experimental evaluation to design complex RNA molecules.

These results suggest a route to developing single-strand nucleic-acid sensors for multi-gene signatures for diseases beyond active TB, including septic shock, cardiovascular disease, vaccine-induced immunity to malaria, and cancers^32–34,38,49^. However, there are limitations to the current proof of concept. Eterna and Nucleologic cannot, at present, handle sensor designs with more than six inputs due to the impractical factorial scaling of computational time with the number of interacting strands^46^. This limitation currently precludes the automated design of, for example, the ten-gene signature for sepsis^32^. Furthermore, in future efforts, it will probably remain necessary to experimentally screen multiple designs; current automated designs have generally acceptable performance (AUROC of 0.9) but are worse at extreme input gene concentrations, presumably due to inaccuracies in the available modelling packages. Importantly, the application of single-molecule sensors for low-cost diagnostics will require enzymatic amplification of gene segments and the readout of reporter binding in inexpensive platforms.

The success of single-strand nucleic-acid sensors from Eterna players and from Nucleologic demonstrates a new sensor design strategy for nucleic-acid computing. Due to the simplicity of single-strand nucleic-acid sensors, the sensors can be used in a variety of settings. Although this study explored the output of binding a fluorescent MS2 protein and a fluorescent RNA reporter, a previous study explored small-molecule input and output^14^. With the flexibility of different modalities, single-strand nucleic-acid sensors might be incorporated in different nucleic-acid computing workflows and could even use other outputs such as protein production if incorporated in an mRNA such as a toehold switch^30^. Depending on the system, different modalities would be preferred. For example, in an in vitro system, an RNA output would enable better qARs or the capability to interface with other nucleic acids in a chemical network. To engage with different proteins or ligands in vivo, aptamers or ribosome binding sites could be used instead.

There are more general limitations to this work. Most fundamentally, we only designed single-molecule sensors for up to three distinct inputs and for systems restricted to two states. The space of functions able to be approximated by a molecular sensor is much larger, comprising the space of all positive rational polynomials. This space theoretically allows for the approximation of any continuous function in the non-negative quadrant with a non-zero leading homogeneous term (Supplementary Appendix: Molecular rational function approximation). Designing systems that have more than two states and bind more than three inputs would allow for the sensing of more complex functions, with more elaborate contours such as ellipses or piecewise linear functions encoded by artificial neural networks^19^, but has not yet been carried out. Finally, for applications involving low-energy computing or embedding computation in nucleic-acid therapeutics, the thermodynamic reversibility of sensors would enable repeated and continuous use in real-world settings. Such reversibility appears feasible through the approaches described here and has been demonstrated for RNA sensors of single small molecules^14^ but remains to be demonstrated for multi-input sensors.

## Methods

### Eterna online interface

The design of RNA molecules in Eterna has been described previously^14,41^. In this Article the interface was further improved to allow for visualization, calculations and design using several strands of RNA. Multistranded folding calculations from the NUPACK folding package^46^ were integrated into Eterna, thereby providing players with computational feedback during the design process. For designs utilizing an RNA reporter output, the RNA reporter binding site was fully unconstrained in terms of binding site location, and the simulated reporter concentration was set to 100 nM (single-input sensors) or 25 nM (OpenTB). The puzzle interface provided visualization of the secondary structure of the complex with highest probability under each simulated condition. All RNAs were constrained to have no repeat of any four nucleotides and uniform lengths of 85 nucleotides to aid synthesis. Only the RNA in/MS2 out designs were 77 nucleotides. Wet-lab experimental scores were converted to numbers between 0 and 100 and were based on either qARs or, for multiple-state problems, lower-bound qARs over a subset of input conditions (qAR_LB*_) (see main text). Links to all puzzle project pages, which include in-game scores, all submitted designs and experimental summaries made available to the player community are compiled in Supplementary Table 2.

### High-throughput characterization of designs

The quantitative characterization and analysis of RNA designs through RNA-MaP was performed as previously described^13,14,42,43,62^. DNA templates for designs were purchased in oligonucleotide pools (CustomArray), amplified by PCR or emulsion PCR, and sequenced on Illumina MiSeq instruments (primers are described in ref. ^14^). The RNA was transcribed directly on the MiSeq sequencing chip in a repurposed Illumina Genome Analyzer II instrument. The sequences and protocols for preparing an array of clonal RNA clusters and for preparing fluorescently labelled MS2 coat protein were those described in ref. ^14^. Only clusters that had sequences that 100% matched a design were used. Here, several fluorescent RNA reporters were also used to measure the affinity across several input conditions. For each experiment, a full binding curve was collected for each cluster over a concentration range of 0.7–1,500 nM for the MS2 protein and 0.09–1,500 nM for fluorescent RNA oligonucleotides. Incubation times varied from 0.8–1.5 h at the lowest concentrations to 10–20 min at the highest concentrations. The RNA input and output sequences used in the experiments are shown in Supplementary Table 3. The experimental conditions tested for all challenges on RNA-MaP are shown in Supplementary Table 4. For each condition, a binding curve was fit to find the *K*_d_ for each cluster; the median across clusters with the same sequence was taken as the *K*_d_ for the sequence, and the error was estimated by the standard error on the mean. From these *K*_d_ values, the qAR was computed for each design, with errors estimated through error propagation.

### RNA-MaP upper bound on accurate qAR measurements

When testing all the designs through RNA-MaP we had a soft upper bound of qAR that could be accurately measured. For precise *K*_d_ quantification, we needed measurements that spanned fourfold above and below the concentration of the true *K*_d_. This limited the range of reliable *K*_d_ measurements to 2.8–375 nM for the MS2 protein and 0.36–375 nM for fluorescent RNA oligonucleotides. Taking the upper and lower bound, we obtained a softer upper qAR bound of 134 for the MS2 protein and 1,042 for fluorescent RNA oligonucleotides. This did not prevent us from measuring qARs above the limit but it did limit the precision of such measurements. In the figures we use a rounded value of 1,000 as the qAR limit for sensors using fluorescent RNA oligonucleotides. For fluorescent MS2, we use a rounded value of 130.

### Independent assessment of TB-score sensors using flow cytometry

Flow cytometry enabled the characterization of selected sensors across a large collection of input RNA concentrations. For each RNA sensor, DNA primer oligonucleotides for assembly were found using Primerize^63,64^ and ordered from Integrated DNA Technologies (IDT). Full-length DNA templates were assembled using the standard PCR assembly protocols available at https://primerize.stanford.edu. Briefly, 100 µl of 1× PCR mix containing Phusion DNA polymerase (Thermo Fisher Scientific) was prepared with 2 µM of first and last primers (P1 and P4 or P6 for BC_AK2.2 or HP_MCTS_130_DEC, respectively) and 40 nM of the other primers. The DNA was then amplified and transcribed to RNA as previously described^14^. For the MCTS_DNA_DEC construct, the sequence was ordered as a single-stranded DNA from IDT. The sequences are listed in Supplementary Table 3. Nucleic-acid beads were prepared as in a previous study on small-molecule sensors^14^. Nucleic acids were loaded onto the magnetic bead by first preparing 3.33 μl of bead mix solution by mixing 0.33 μl of poly-T-coated beads, 0.175 μl of RNA (250 nM) and 2.825 μl of H_2_O, followed by incubation at 37 °C for 5 min, and cooling on ice for 5 min. The buffer was removed and the beads washed three times with 100 μl of solution containing 1× Other buffer and 1× TMK buffer (10× Other buffer contained 1 mg ml^−1^ bovine serum albumin (BSA), 10 mM dithiothreitol (DTT), 0.1 mg ml^−1^ yeast tRNA, 0.1% Tween-20; 5× TMK buffer contained 500 mM Tris-HCl pH 7.5, 400 mM KCl, 20 mM MgCl_2_). After washing, the beads were resuspended in 3.33 μl of H_2_O. The bead mix was then added to 20 μl TMK buffer, 10 μl of Other buffer, 3 μl reporter (R) RNA and 43.66 μl of H_2_O, resulting in 80 μl of solution. 20 μl of solution containing different concentrations of RNA A, B and C was added. The final concentration of R was 30 nM, set slightly higher than the 25 nM simulated reporter concentration in Eterna based on empirical calibration of sensor affinities from RNA-MaP experiments. After mixing, the samples were incubated for 2 h on a shaker at room temperature. Each sample was analysed using a Sony SH800S Cell Sorter, and data for 10,000 events were collected per sample. Beads were excited using a 561-nm laser and their emitted fluorescence was measured from the 600 ± 60-nm emission channel. An example of the raw flow cytometry data collected is provided in Extended Data Fig. 8.

### Nucleologic

Nucleologic is a Monte Carlo Tree Search (MCTS) algorithm^59,60^ for designing RNA sensors, available at https://eternagame.org/about/software. Inspired by the Eterna player strategy of DMSSD, the sensor is treated as an ordered list of domains with each domain containing its own sequence. The root node of the MCTS is generated based on the user input. Possible inputs and outputs are limited to aptamers and RNA/DNA. When using aptamers in Nucleologic, the sequence, secondary structure and *K*_d_ must be specified. The input file must also specify the condition(s) that the sensor must satisfy for it to be considered a solution. Each condition is specified as on or off depending on whether the output is bound or not. The criterion of success is specified, at which point the algorithm terminates even if it has not completed the total number of iterations, *N*_iterations_. For example, the on state could have input A set to 100 nM concentration and the off state could have input A set to 0 nM concentration; and the early termination success criterion could be specified as having a predicted qAR of >50. At least one on and one off condition must be specified. Extra parameters to alter the MCTS run can also be specified such as the number of iterations, number of children generated, folding package (for example, NUPACK) and so on. The code documentation includes details and examples of input files. The MCTS then involves growing a tree whose nodes represent sequence solutions for the sensor, with scores updated through the following four-step process.

#### Step 1—selection

Starting from the root node of the tree, child nodes are successively chosen until a leaf node is reached. If a node has multiple children nodes, the child node that has the maximum value of the upper confidence bound for trees (UCT) score is chosen:

1 $${\rm{UCT}}=\frac{v}{n}+c\sqrt{\frac{\mathrm{ln}N}{n}}$$

where *N* is the total count of visits for the parent node, *n* is the total count of visits for the child node, *c* is the exploration constant, and *v* is the value of the child node’s sequence. The value *v* is defined to be sum of Boltzmann probabilities *P*_*i*_ that each state matches its target condition (on or off):

2 $$\begin{array}{c}{v}=\mathop{\sum}\limits_{i}{\mathrm{ln}}{P}_{i}\\ {P}_{i}={\rm{Prob}}({\rm{constraint}}{i})\end{array}$$

The value of each node thus reflects the probability to fulfil the desired constraints, with a higher value being closer to ideal. This formulation of the node value for MCTS is heuristic. Alternative formulations could be explored, but are not discussed here.

Every node in the tree keeps track of how many times it was visited to reach the chosen child node, which updates *n*.

#### Step 2—expansion

Once a leaf node is chosen, a child node is created by running the Metropolis–Hastings algorithm. Starting with the leaf node sequence, the sequence is randomly mutated through either a domain mutation or domain move (Fig. 7) and its corresponding value *v* is computed. The probability of accepting the sequence as the child node is computed as

$$\begin{array}{c}\Delta v={\min} ({v}_{\rm{child}}-{v}_{\rm{leaf}},\,0)\\ P={\rm{e}}^{\frac{\Delta v}{T}}\end{array}$$

where *T* is an effective temperature that scales the value of Δ*v*. If the value of the child node candidate sequence is better (higher) than the leaf node, the sequence is always accepted. This is repeated until *n* child nodes are created from the leaf node.

#### Step 3—score and backpropagation

After selecting a random child node created in step 2, the score is ‘backpropagated’ up the tree by updating the UCT score of the parent node, its parent node and so on, back up to the root node. The only change in the UCT calculation comes from the update of *N*, the total count of visits for the parent node, and *n*, the total count of visits for the child node. These updates allow subsequent stages of the search to balance exploitation of specific solutions with exploration of a broader diversity of solutions through equation (1).

Typical values for MCTS searches were *n* = 3, *c* = 1*, N*_iterations_ = 300 (corresponding to the maximum number of expansion steps of 100) and *T* = 0.61597. We chose our default *T* from *k*_B_*T*, where *k*_B_ = 1.987 kcal (mol K)^−1^ and *T* = 310 K.

The running time of Nucleologic was dominated by the NUPACK calculations. For OpenTB challenge 3, each run of Nucleologic took on average 7.5 h, with the NUPACK call per sequence being ~1.5 min.

### Nucleologic moves

In the expansion step of Nucleologic, the sequence is randomly mutated through either a domain mutation or domain move.

#### Domain move

A domain is randomly removed and a random index, excluding the original index, is chosen for reinsertion. Multiple domains can be moved at once.

#### Domain mutation

A domain is randomly chosen for mutation. There are two possible options: swap or mutation. For both moves, users can designate which domains cannot be modified, such as the MS2 hairpin domain.

A swap is when a domain is replaced with a newly generated domain. For example, if domain A′ is chosen, then a newly complementary sequence to A replaces the existing A′ sequence. If the domain chosen is not complementary to anything, it is replaced by a randomly generated sequence. The generated sequence is the same length as the existing sequence.

If mutation is chosen, there are two options. Option A is to mutate a random base in the chosen domain. Option B is to pick the base on the 5′ or 3′ end of the domain. Once chosen, the base is mutated randomly and then attached to the end of the corresponding adjacent domain. For example, if the layout of the construct is A–B–C and the 5′ end of domain B was chosen, then the nucleotide on the 5′ end of B is randomly mutated and then added to the 3′ end of domain A. This results in domain B shrinking by one nucleotide and domain A growing by one nucleotide. Either option is chosen randomly.

### Nucleologic in silico screening

Nucleologic designs for the openTB challenge were screened using NUPACK by simulating the signal of the sensor at different concentrations of inputs [A], [B] and [C] while keeping [R] at 30 nM. The concentrations consisted of ten values evenly spaced on the log scale from 1 pM to 1 μM for a total of 1,000 conditions. From these simulated conditions, a logistic regression model was fit and AUROC was computed to rank the top designs. Of the few hundred designs screened, only the top four designs, two INC and two DEC designs, were selected for experimental validation with flow cytometry (Extended Data Fig. 5). For the DNA designs only two designs were tested, one INC and DEC, respectively.

### Functions computed by RNA sensors

Equilibrium sensors can be modelled using simple equilibrium expressions involving ratios of polynomials with positive coefficients, also called positive rational polynomials, as described in the Supplementary Appendix: Molecular rational function approximation. We give four examples from each of the challenges in this study below.

#### Pilot challenge—RNA sensors for single-input oligonucleotides

With a single-input RNA A and an output reporter R, a two-state model is adequate for describing the desired sensor S. As an example, the scheme for an off sensor with high enough concentrations of A and R is described by the equilibrium A•S ↔ R•S, and the fraction of sensor with reporter bound, *f*_R_, is

$${f}_{\rm{R}}={\frac{[{\rm{R}}]}{[{\rm{R}}]+K[{\rm{A}}]}}$$

where *K* is an equilibrium constant, which is simulated in Eterna with NUPACK. At a fixed reporter concentration (100 nM simulated in Eterna), the sensor’s fluorescence switches from 1 to 0 as [A] increases from 0 to high concentrations. To precisely characterize the sensor RNA-MaP experiments, the concentration of R was titrated and its apparent dissociation constant ${K}_{\rm{d}}^{\rm{app}}$ was measured at zero and high [A]. At equilibrium, the expression above and the standard relationship defining the dissociation constant, that is, ${f}_{{\rm{R}}}={\frac{[{\rm{R}}]}{[{\rm{R}}]+{K}_{\rm{d}}}}$, guarantees that ${K}_{\rm{d}}^{\rm{app}}={K}[{\rm{A}}]$ for an accurately designed single-input sensor. Although the maximal AR for the system is unbounded in this simple two-state model, taking into account other states (for example, free sensor in the absence of A or R) leads to the maximum qAR given by the ratio of the test concentration [A] (here, 100 nM) and the intrinsic hybridization affinity of A for its complement (which can be femtomolar or smaller)^50^. This value can be very large (>10^6^), so, in practice, the maximum AR value for ‘binary’ sensors responding to oligonucleotide inputs is limited by the experimental range of ${K}_{\rm{d}}^{\rm{app}}$ measurable in RNA-MaP (~1,000). Similar expressions and considerations hold for an on sensor^50^.

#### Challenge 1—two-input logic gates

For two inputs and complex logic gates, a few-state model remains sufficient to describe the sensor. The most complex case is an XOR system that responds to two inputs A and B, which is minimally described by four states: S ↔ A•R•S ↔ B•R•S ↔ A•B•S. The output of the system is described by

$${f}_{{\rm{R}}}={\frac{{K}_{{\rm{A}}}[{\rm{A}}][{\rm{R}}]+{K}_{{\rm{B}}}[{\rm{B}}][{\rm{R}}]}{1+{K}_{{\rm{A}}}[{\rm{A}}][{\rm{R}}]+{K}_{{\rm{B}}}[{\rm{B}}][{\rm{R}}]+{K}_{{{\rm{AB}}}}[{\rm{A}}][{\rm{B}}]}},$$

where *K*_A_, *K*_B_ and *K*_AB_ are equilibrium constants that can be estimated in packages such as NUPACK. For a given [R], the expression is zero under conditions without A or B, or conditions with high concentrations of both A and B, but approaches 1 with high concentrations of just A or just B. RNA-MaP experiments varied [R] to enable more precise characterization, fitting an apparent dissociation constant, which, in the case of a well-designed sensor, conforms to the rational polynomial ${K}_{\rm{d}}^{\rm{app}}={\frac{1+{K}_{{{\rm{AB}}}}[{\rm{A}}][{\rm{B}}]}{{K}_{{\rm{A}}}[{\rm{A}}]+{K}_{{\rm{B}}}[{\rm{B}}]}}$. This should lead to a weak (large ${K}_{\rm{d}}^{\rm{app}}$) value without any inputs or with both inputs, and a tight (small ${K}_{\rm{d}}^{\rm{app}}$) value with high concentrations of any single input. For A and B that bind their complement tightly, maximal qARs can be >10^6^; as with single-RNA input gates, the maximum qARs are set here by the experimental range of ${K}_{\rm{d}}^{\rm{app}}$ measurable by RNA-MaP, about 1,000 with MS2 protein binding. See Supplementary Appendix: Molecular rational function approximation for a graph of an XOR sensor response.

#### Challenge 2—ratio sensor

A ratio sensor requires only two states to describe, A•R•S ↔ B•S. The output of the system is

$${f}_{{\rm{R}}}={\frac{[{\rm{A}}][{\rm{R}}]}{[{\rm{A}}][{\rm{R}}]+K[{\rm{B}}]}}={\frac{([{\rm{A}}]/[{\rm{B}}])}{([{\rm{A}}]/[{\rm{B}}])+r}},$$

where *K* is an equilibrium constant, and *r* = *K*/[R]. At a fixed reporter concentration [R], the system therefore varies from 0 to 1 as a monotonic function of the ratio [A]/[B], with the midpoint (‘separatrix’) set by *r*, here targeted as *r* = 1/4 for [R] set to the *K*_d_ for MS2 coat protein reporter; in Eterna, this condition is equivalent to simulating whether the MS2 hairpin is displayed or not displayed in the lowest free-energy sensor state at the different [A] and [B]. RNA-MaP experiments characterized sensors by titrating [R] and measuring the apparent dissociation constant, which, for an accurately designed sensor, is given by ${K}_{\rm{d}}^{\rm{app}}={K}[{\rm{A}}]/[{\rm{B}}]$. For a perfect sensor, a lower bound on the qAR is set by the two conditions whose [A]/[B] are closest to the separatrix *r* from above and below: qAR_LB_ = min_[A]/[B] > *r*_ ([A]/[B])/max_[A]/[B] < r_ ([A]/[B]).

#### Challenge 3—OpenTB sensor

Sensors computing the TB-score, which depend on the concentrations of three RNA segments as [A][B]/[C]^2^, can be achieved with designs that populate just two states. For a DEC sensor, the two states are A•B•S ↔ C•C•R•S, and

$${f}_{{\rm{R}}}={\frac{{[{\rm{C}}]}^{2}[{\rm{R}}]}{K[{\rm{A}}][{\rm{B}}]+{[{\rm{C}}]}^{2}[{\rm{R}}]}}={\frac{r}{[{\rm{A}}][{\rm{B}}]/{[{\rm{C}}]}^{2}+r}},$$

where *K* is an equilibrium constant, and *r* = [R]/*K*. Similar to the ratio sensor above, at a fixed reporter concentration [R], the system varies from 1 to 0 as a monotonic function of the ratio [A][B]/[C]^2^ with the midpoint (‘separatrix’) set by *r*. Here, we targeted *r* = 1/16 at [R] = 25 nM. This value for *r* was set based on a study^36^ that defined the TB-score as (*GBP5* + *DUSP3*)/2 − *KLF2*, with individual values defined as logarithm (base 2) of gene concentrations to enable convenient comparison to quantitative RT–PCR cycle threshold values, and a separatrix value of −2 based on clinical samples. A, B and C refer to *GBP5*, *DUSP3* and *KLF2*, respectively. Again, similar to the ratio sensors, RNA-MaP experiments read out ${K}_{\rm{d}}^{\rm{app}}={K}[{\rm{A}}][{\rm{B}}]/[{\rm{C}}]^2$ for a perfect TB-score sensor, and qAR_LB_ = min_[A][B]/[C][C] > *r*_ ([A][B]/[C]^2^)/max_[A][B]/[C][C] < r_ ([A][B]/[C]^2^). Similar expressions and considerations hold for an INC sensor (reporter binding at [A][B]/[C] above rather than below a threshold).

## Online content

Any methods, additional references, Nature Portfolio reporting summaries, source data, extended data, supplementary information, acknowledgements, peer review information; details of author contributions and competing interests; and statements of data and code availability are available at 10.1038/s41557-025-01907-8.

## Supplementary information

Supplementary Information Supplementary Appendix: Molecular rational function approximation and Supplementary Figs. 1–5.

Supplementary Data 1 Contains Supplementary Appendix: Eterna player resources (Eterna player resources also listed in Supplementary Table 4). Also contains the newsletters the Das lab shared with Eterna players after each design challenge.

Supplementary Tables 1–5 Supplementary Table 1 contains the consortium author list for Eterna participants. Supplementary Table 2 contains the links to all Eterna puzzle projects as well as a summary of each puzzle round. Supplementary Table 3 contains the sequences of all RNA and DNA sequences used throughout the manuscript. Supplementary Table 4 contains the Eterna player resources. Supplementary Table 5 contains the experimental conditions used to validate designs on RNA-MaP for all challenges.

## Data Availability

Experimental data for all figures, including estimated *K*_d_ values for each tested sequence, can be found in the GitHub repository at https://github.com/eternagame/paper-data-rationally-designed-RNA-sensor. Data compiled at the finer level of individual RNA-MaP sequence clusters are available at https://github.com/eternagame/EternaDataRibonet.

## Code availability

Code for generating simulated plots for visualizing response curves to different concentrations of A, B and C can be found in the Github repository: https://github.com/eternagame/conc_plots. Code for Nucleologic can be downloaded from https://eternagame.org/about/software.
