# Supplementary material for: Compact RNA sensors for increasingly complex functions of multiple inputs
Source: Nat Chem. 2025 Nov 12;17(12):1839–52. doi: 10.1038/s41557-025-01907-8 (PMC12669046; doi:10.1038/s41557-025-01907-8)
Supplement: Supplementary file 1 — Supplementary Appendix: Molecular rational function approximation and Supplementary Figs. 1–5. [file 41557_2025_1907_MOESM1_ESM.pdf]

nature chemistry

Supplementary information https://doi.org/10.1038/s41557-025-01907-8

Compact RNA sensors for increasingly complex functions of multiple inputs

In the format provided by the authors and unedited

Supplementary Appendix: Molecular rational function approximation

1 Single molecules as rational functions

Nature exhibits remarkable computational abilities mediated through the complex interactions between nucleic acids and proteins. The capacity of a molecule to bind input and output ligand(s) endows the potential to carry out computations *in-situ*. Among the vast array of biomolecules, nucleic acids, such as DNA and RNA, hold particular promise as molecular calculators due to their versatility and the ease with which they can be designed and manipulated. Although the complexity of molecular calculators can be enhanced by linking multiple calculators together, this supplement concentrates on the investigation of single-molecule calculators.

1.1 Partition function of a generalized molecular calculator at equilibrium

Given a single-molecule calculator, S, that has N unique binding sites for ligand A and M unique binding sites for a reporter ligand R we can write out all the states of S complexed with A and R. We denote $S{\cdot}A_k$ as the set of all states where A bound to the kth-unique binding site on S. We let R denote a fluorescent reporter that gives a signal of 1 when bound by S and a signal of 0 when free in solution. In practice, other output modalities can be used such as transcription activation.

$$\text{Let } S_A = \{A_i | i = 1..N\}, S_R = \{R_i | i = 1..M\}$$

$$\binom{S_i}{k} \text{ denote the set of all combinations choosing } k \text{ from } S_i$$

$$\text{E.g.} \binom{S_A}{2} = \bigcup_{i=1}^{N} \bigcup_{j=i+1}^{N} A_i{\cdot}A_j = \{A_i{\cdot}A_j | i, j = 1..N, i \neq j\}$$

We can express all the states of S complexed with A and R.

$$\begin{aligned}
\text{States } &= \{S \text{ bound to 1 copy of } A\} \cup \{S \text{ bound to 2 copy of } A\} \cup ... \\
&= \left\{ \bigcup_{j=0}^{M} \bigcup_{a \in \binom{S_A}{0}} \bigcup_{r \in \binom{S_R}{j}} S{\cdot}a{\cdot}r \right\} \cup \left\{ \bigcup_{j=0}^{M} \bigcup_{a \in \binom{S_A}{1}} \bigcup_{r \in \binom{S_R}{j}} S{\cdot}a{\cdot}r \right\} \cup ... \\
&= \bigcup_{i=0}^{N} \bigcup_{j=0}^{M} \bigcup_{a \in \binom{S_A}{i}} \bigcup_{r \in \binom{S_R}{j}} S{\cdot}a{\cdot}r \qquad (1)
\end{aligned}$$

From the set of states, we can proceed to computing the partition function Z. Assume the system is in thermodynamic equilibrium and $[R]$ is constant, we may write the partition function of the system as

$$Z = \sum_{i=0}^{N} \sum_{j=0}^{M} \sum_{a \in \binom{S_A}{i}} \sum_{r \in \binom{S_R}{j}} Z_{S \cdot a \cdot r}$$

If we know the ΔG and the dissociation constants K_A and K_R for each of the binding sites, then we can use the following substitution to simplify Z.

$$Z_{S \cdot a \cdot r} = \frac{[A]^i [R]^j}{K_A^i K_R^j} e^{-\beta \Delta G_{i,j}}$$

where $\beta = 1/k_B T$, k_B is the Boltzmann's constant, and T is temperature. K_A and K_R are the dissociation constants of A and R respectively with units $\frac{L}{mol}$

From this substitution we can get the following.

$$\begin{aligned} Z &= \sum_{i=0}^{N} \sum_{j=0}^{M} \frac{[A]^i [R]^j}{K_A^i K_R^j} e^{-\beta \Delta G_{i,j}} \\ &= \sum_{i=0}^{N} [A]^i \sum_{j=0}^{M} \frac{[R]^j}{K_A^i K_R^j} e^{-\beta \Delta G_{i,j}} \end{aligned}$$

Let $a_{i,j} = \frac{[R]^j}{k_{i,j}} e^{-\beta \Delta G_{i,j}}$ where $k_{i,j} = K_A^i K_R^j$

$$Z = \sum_{i=0}^{N} [A]^i \sum_{j=0}^{M} a_{i,j} \tag{2}$$

The term $a_{i,j}$ represents the degeneracy and energetic contributions of all states of S complexed with i copies of A and j copies of R.

We can simplify (2) further by letting $x = [A]$ and $a_i = \sum_{j=0}^{M} a_{i,j}$ resulting in the following.

$$Z = \sum_{i=0}^{N} a_i x^i \tag{3}$$

1.2 Calculating the output probability of the biomolecule

Using the partition function defined in the previous section, we can compute the probability of n copies of R bound to S which is related to the observable in an experiment.

We write for our partition function of activated states,

$$Z_{\text{out}} = \sum_{i=1}^{N} b_i x^i \tag{4}$$

Since the set of activated states is always a subset of the total states represented by a_i we have $0 \leq b_i \leq a_i$. Thus the signal, $f(x)$ of the system is represented by Z_{out}/Z.

If S can only bind up to one copy of the reporter we can write out the functional equation of a system where $M = 1$. We assume the signal of the reporter is 0 when free in solution, and 1 when bound.

$$f(x) = p(R \text{ bound}) = \frac{\sum_{i=0}^{N} b_i x^i}{\sum_{i=0}^{N} a_i x^i} \leq 1,\ 0 \leq b_i \leq a_i \tag{5}$$

If $M > 1$ our function is

$$\begin{aligned} f(x) &= \sum_{j=1}^{M} j \cdot \frac{\sum_{i=0}^{N} b_{i,j} x^i}{\sum_{i=0}^{N} a_i x^i} \\ &= \frac{\sum_{i=0}^{N} x^i \sum_{j=1}^{M} j \cdot b_{i,j}}{\sum_{i=0}^{N} a_i x^i} \\ c_i &= \sum_{j=1}^{M} j \cdot b_{i,j} \\ f(x) &= \frac{\sum_{i=0}^{N} c_i x^i}{\sum_{i=0}^{N} a_i x^i} \leq M, \end{aligned} \tag{6}$$

$$a_i, c_i \geq 0$$
$$c_i = 0 \iff a_i = 0$$

One way to interpret the expression (5) is through probability. $f(x)$ can be written as the sum of probabilities of different number of copies bound attenuated by some signaling strength factor.

$$f(x) = \sum_{k=0}^{N} A_k p(k) \tag{7}$$

A_k represents the signaling strength when k copies are bound and $p(k) = \frac{a_k x^k}{\sum_{i=0}^{N} a_i x^i}$ is the probability of k copies bound. Depending on M, $A_k \cdot a_k = b_k$ or c_k.

In theory, we can express almost any positive rational function with positive coefficients as the partition function of a molecular calculator at equilibrium (5).

One limitation is that since $c_i = 0$ if and only if $a_i = 0$ the degree of the numerator must be less than or equal to the degree of the denominator. In order to overcome this limitation one can use $\frac{1}{f(x)}$ as the output function.

1.3 Molecular functions in higher dimensions

We can expand our function of Z from (3) to higher dimensional rational functions. Let the molecule, S, have binding sites for ligand x_1, x_2, ..., x_n where each ligand x_k has N_k unique binding sites. Also, let S able to bind m copies of a reporter. We can write out the new partition function.

$$Z = \sum_{\alpha_1=0}^{N_1} \sum_{\alpha_2=0}^{N_2} \dots \sum_{\alpha_n=0}^{N_n} a_{\alpha_1,\alpha_2,\dots,\alpha_n} x_1^{\alpha_1} x_2^{\alpha_2} \dots x_n^{\alpha_n} \tag{8}$$

Using this partition function we can write the molecular calculator's output signal as a higher dimensional rational function which has the same constraints as (5).

$$f(x_1, x_2, ..., x_n) = \frac{\sum_{\alpha_1=0}^{N_1} \sum_{\alpha_2=0}^{N_2} ... \sum_{\alpha_n=0}^{N_n} c_{\alpha_1,\alpha_2,...,\alpha_n} x_1^{\alpha_1} x_2^{\alpha_2} ... x_n^{\alpha_n}}{\sum_{\alpha_1=0}^{N_1} \sum_{\alpha_2=0}^{N_2} ... \sum_{\alpha_n=0}^{N_n} a_{\alpha_1,\alpha_2,...,\alpha_n} x_1^{\alpha_1} x_2^{\alpha_2} ... x_n^{\alpha_n}} \tag{9}$$

2 Polynomial approximation

2.1 Polynomial decomposition

In addition to approximating rational functions, it is also possible for the system to approximate any positive polynomial. The Stone–Weierstrass theorem [1] states that any continuous function defined on a closed interval [a, b] can be uniformly approximated as closely as desired by a polynomial function. It is possible to create a polynomial to any desired function.

Let $f(x)$ be our polynomial where $f(x) > 0$ when $x \geq 0$. We apply these constraints on the range and domain since partition functions can only express positive values and the input in the system can never be negative. Poincaré showed that polynomials can be decomposed into linear and quadratic terms.

$$f(x) = F_1 \cdot F_2 \cdot F_3 \cdot F_4 \tag{10}$$

$$\begin{aligned} F_1 &= \prod_i x + x_{n,i} \\ F_2 &= \prod_i x^2 - a_i x + b_i \\ F_3 &= \prod_i x^2 + \alpha_i x + \beta_i \\ F_4 &= \prod_i x - x_{p,i} \\ a_i, b_i &> 0 \\ \alpha_i, \beta_i &> 0 \\ x_{n,i}, x_{p,i} &> 0 \end{aligned}$$

Since we have a positive function for $x \geq 0$ we can ignore F_4 resulting in $f(x) = F_1 \cdot F_2 \cdot F_3$. Since we know all the coefficients in F_1 and F_3 are positive if we can substitute the quadratic terms in F_2 with rational polynomials with positive coefficients, then it is possible to express any $f(x)$ as a rational polynomial with positive coefficients.

2.2 Quadratic expressed as positive rational function

Meissner [2, 3], simplified by Motzkin and Straus [4], showed that F_2 can be rewritten as a rational function with positive coefficients. Here, we briefly summarize these results.

Start with a quadratic term $x^2 - ax + b$.

$$x^2 - ax + b = \frac{Q_2(x)}{Q_1(x)} \tag{11}$$

$$Q_2(x) = \rho^2 a_0 \left(1 - \frac{\sin((m+2)\theta)}{\sin\theta} \cdot \frac{x^{m+1}}{\rho^{m+1}} + \frac{\sin((m+1)\theta)}{\sin\theta} \cdot \frac{x^{m+2}}{\rho^{m+2}}\right)$$

$$Q_1(x) = \sum_{k=0}^{m} \frac{a_k}{\rho^k} x^k$$

$$\rho^2 = b,\ a = 2\rho cos\theta$$
$$m+1 < \frac{\pi}{\theta} < \frac{\pi}{\sin\theta}$$
$$a_k = \frac{a_0}{\sin\theta}\sin((k+1)\theta),\ k = 1, ..., m$$
$$a_0 = 1$$
$$a_{-1} = a_{m+1} = 0$$
$$b_k = a_{k-1} + a_{k+1} - 2a_k\cos\theta,\ k = 0, 1, ..., m$$

Thus, any positive polynomial can be represented as a rational function with positive coefficients. In theory we can approximate any such polynomial using biomolecular calculators.

For example, using (11) we can transform $x^2 - 2x + 8$.

$$x^2 - 2x + 8 = \frac{\frac{1}{4}x^3 + \frac{1}{2}x^2 + 8}{\frac{1}{4}x + 1} \tag{12}$$

This is also true in the case of higher dimension polynomials which was proved by Meissner [2, 3]. Motzkin and Straus showed that there is an extra constraint [4]. The highest degree homogenous part of the polynomial must not have any nonnegative zeros. For example, $1 + (x - y)^2$ cannot be written as a rational function with positive coefficients since $(x - y)^2$ contains nonnegative zeros.

3 Examples functions

3.1 Sigmoid function

Let the molecule, S, be able to bind only one copy of A and one copy of R.

$$f(x) = \frac{b_0 + b_1 x}{c_0 + c_1 x} \tag{13}$$

This represents the simplest space of rational functions for the system. In addition to containing the constant functions where the output is fixed independent of the input, ON and OFF-switches are also in this space.

An example ON-switch function would be $f(x) = \frac{x}{1+x}$. If we substitute x for e^u we can transform our ON-switch function into a sigmoid where the domain is $(-\infty, \infty)$.

$$f(u) = \frac{e^u}{1 + e^u} = \frac{1}{1 + e^{-u}}$$

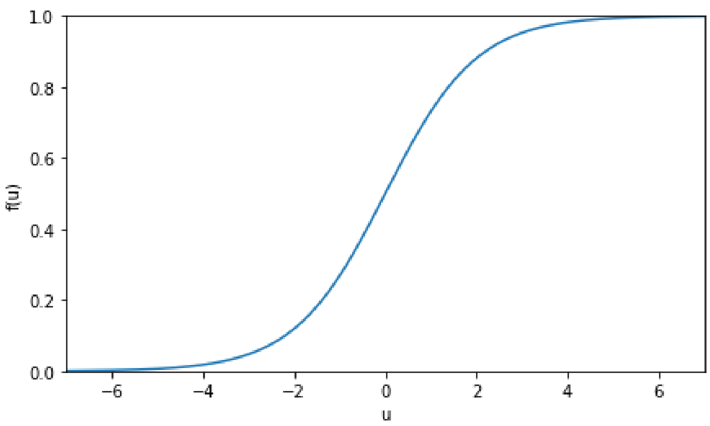

Figure 1: Example sigmoid function

If one defines an output threshold, such that responses above the threshold are labeled as "1", and outputs below the threshold are labeled as "0", then the sigmoid function above has two "islands" in its domain. One island corresponds to an output of 0 and the other island corresponds to an output of 1 (terminology from [5]). The number of islands can be viewed as a measure of complexity: a computer with more islands in its domain has the potential to perform more complex computations.

3.2 Multi-island function

By incorporating more binding sites to the input A, we can create more complex functions that have multiple islands. Let the molecule, S, be able to bind 2 copies of A. This allows the function to have up to three islands in its domain. Such a setup could be useful for detection of a ligand within a specific range of concentrations.

From this system we can create the following function:

$$f(x) = \frac{x}{1 + x + x^2} \tag{14}$$

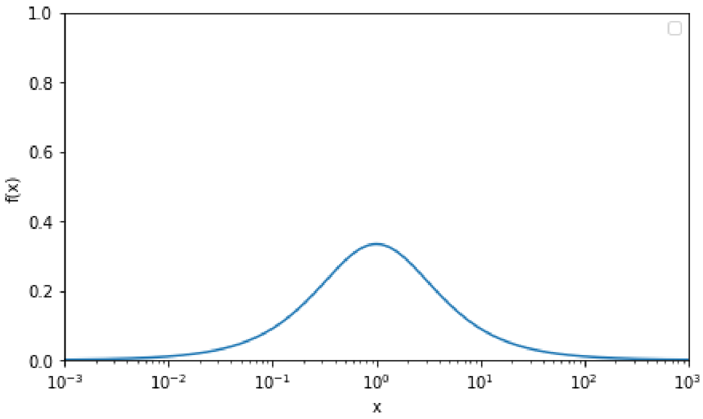

Figure 2: Example three island function

We can create more complex island functions by increasing the number of binding sites beyond 2. If the molecule, S, able to bind 4 copies of A then five islands becomes possible.

$$f(x) = \frac{x + x^3}{.01 + x + 10x^2 + x^3 + .01x^4} \tag{15}$$

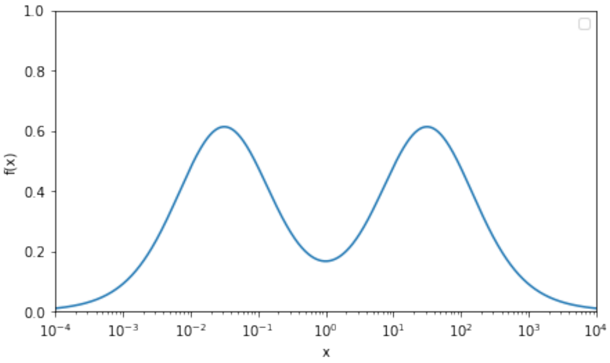

Figure 3: Example five island function

The maximum number of islands is $N + 1$ where N is the number of unique binding sites for ligand A.

3.3 Two-input logic gate

Various two-input logic gates can also be modeled by allowing for two inputs. Let x and y represent the concentration of two different input ligands. We can create a function for a XOR logic gate.

$$f(x, y) = \frac{1 + xy}{1 + x + y + xy} \tag{16}$$

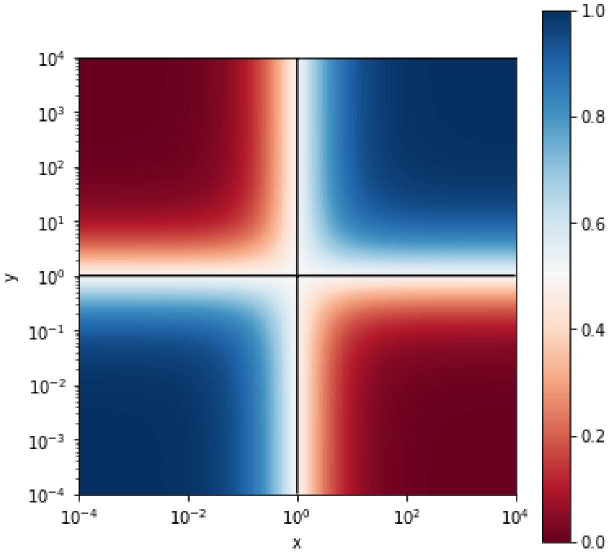

Figure 4: Example XOR logic gate function

References

[1] Marshall H. Stone. The generalized weierstrass approximation theorem. *Mathematics Magazine*, 21(5):237–254, 1948.

[2] Ernst Meissner. Über positive darstellungen von polynomen. *Mathematische Annalen*, 70:223–235, 1911.

[3] Hannah K. Wayment-Steele. A translation of "On the positive representation of polynomials" by Ernst Meissner, 2023.

[4] TS Motzkin and Ernst Straus. Divisors of polynomials and power series with positive coefficients. *Pacific Journal of Mathematics*, 29(3):641–652, 1969.

[5] David H Wolpert. The stochastic thermodynamics of computation. *Journal of Physics A: Mathematical and Theoretical*, 52(19):193001, apr 2019.

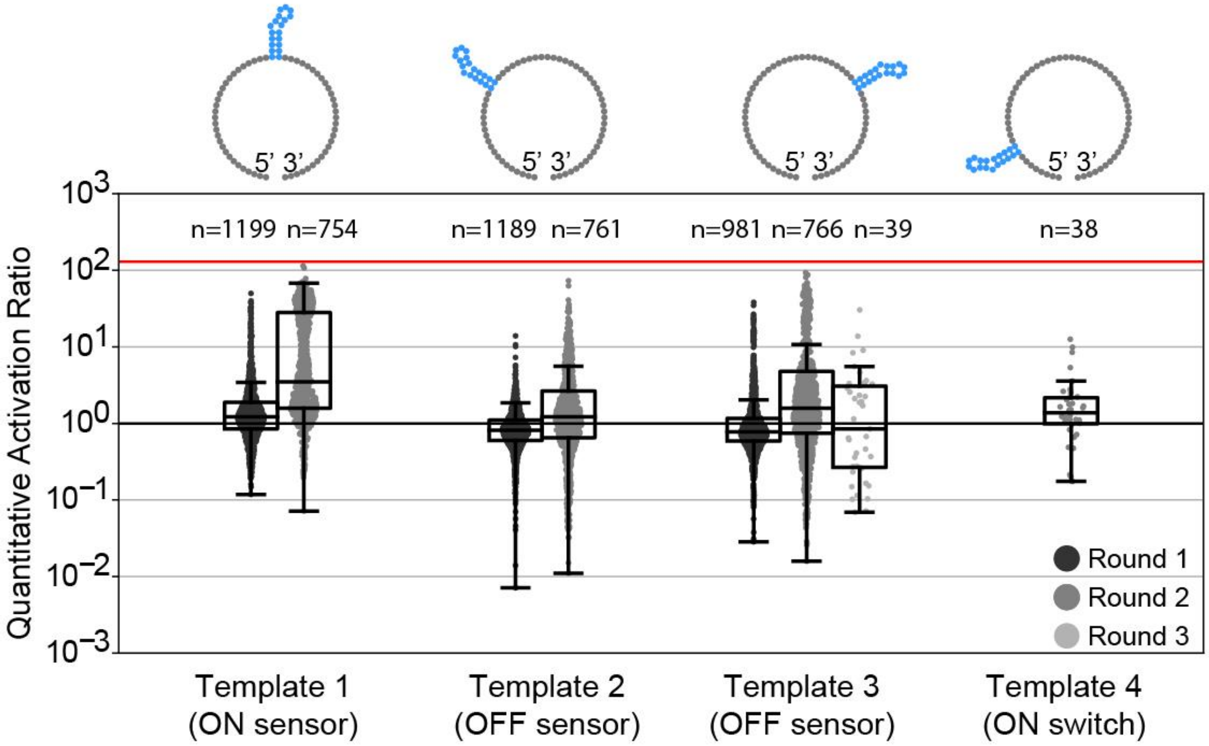

Supplemental Figure 1. All rounds of designs for Template 1 through 4 from pilot challenge. Template 3 submissions were from Eterna players in their third round of this challenge. Template 4 submissions were from an invitation-only Eterna puzzle for Stanford students of the graduate course Biochemistry/Biophysics/Structural Biology 241 (Round 2 supplemental in Supplemental Table 2). The qAR values in these separate later rounds did not exceed the qAR achieved by Eterna participants in early rounds. Players were constrained to four different templates for design with each template having a different MS2 hairpin location (top). The red horizontal line at 130 represents the soft upper limit for quantitative activation ratios that can be accurately measured on RNA-MaP for fluorescent MS2 sensors. The centerline of the box plot indicates the median; the edges of the box correspond to the first and third quartiles; the whiskers extend to 1.5 times the interquartile range.

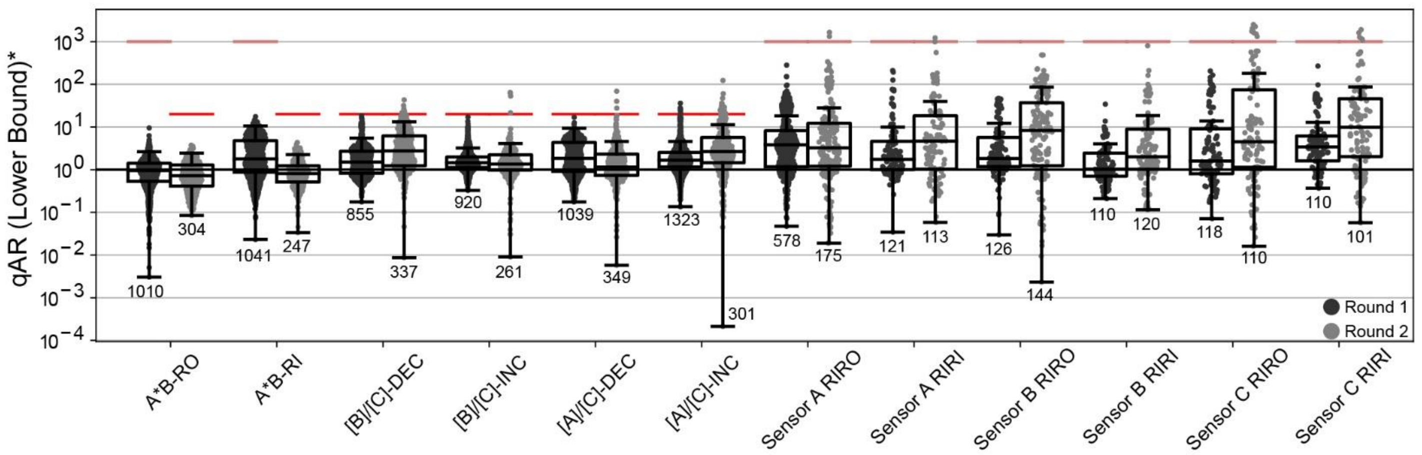

Supplemental Figure 2. Additional puzzles presented alongside Challenge 3 (OpenTB sensor). In addition to the two puzzles that correspond to designing a DEC or INC sensor for the three-gene TB-score, 12 additional puzzles were presented to players as sub-problems.The rounds correspond to the same rounds as in Figure 5 (see Supplemental Table 2 for sequences of A, B, C, and reporter R). Activation ratios (lower bound) are computed using a subset of the ON and OFF conditions related to actual conditions simulated in the Eterna puzzles (denoted as qAR_{LB*} in the main text). For A*B puzzles, in Round 1, four conditions were used for scoring (0 nM A&B, 0 nM A & 100 nM B, and 100 nM A & 0 nM B; and 100 nM A & B) and, in Round 2, three conditions were used for scoring (5 nM A&B, 100 nM A & 5 nM B; and 100 nM A & B). For the ratio sensor puzzles, two conditions were used for scoring with 5 vs. 100 nM of the ‘numerator’ oligonucleotide input, and 100 nM C in both conditions. For single-input sensor puzzles, two conditions were used for scoring, 0 nM or 100 nM for the input, and 0 nM concentrations of other oligonucleotides. See also **Supplemental Table 5**. The red horizontal line is a soft upper limit for quantitative activation ratios that can be accurately measured on RNA-MaP (1000); when the red line is below 1000 it indicates the theoretical maximum qAR_{LB*} based on the conditions for the respective puzzles. Abbreviations: INC or RI, puzzles where binding of output oligonucleotide R occurs at high values of input concentrations; DEC or RO, puzzles where binding of output occurs at low input values. The centerline of the box plot indicates the median; the edges of the box correspond to the first and third quartiles; the whiskers extend to 1.5 times the interquartile range. The number of samples in each box plot is shown.

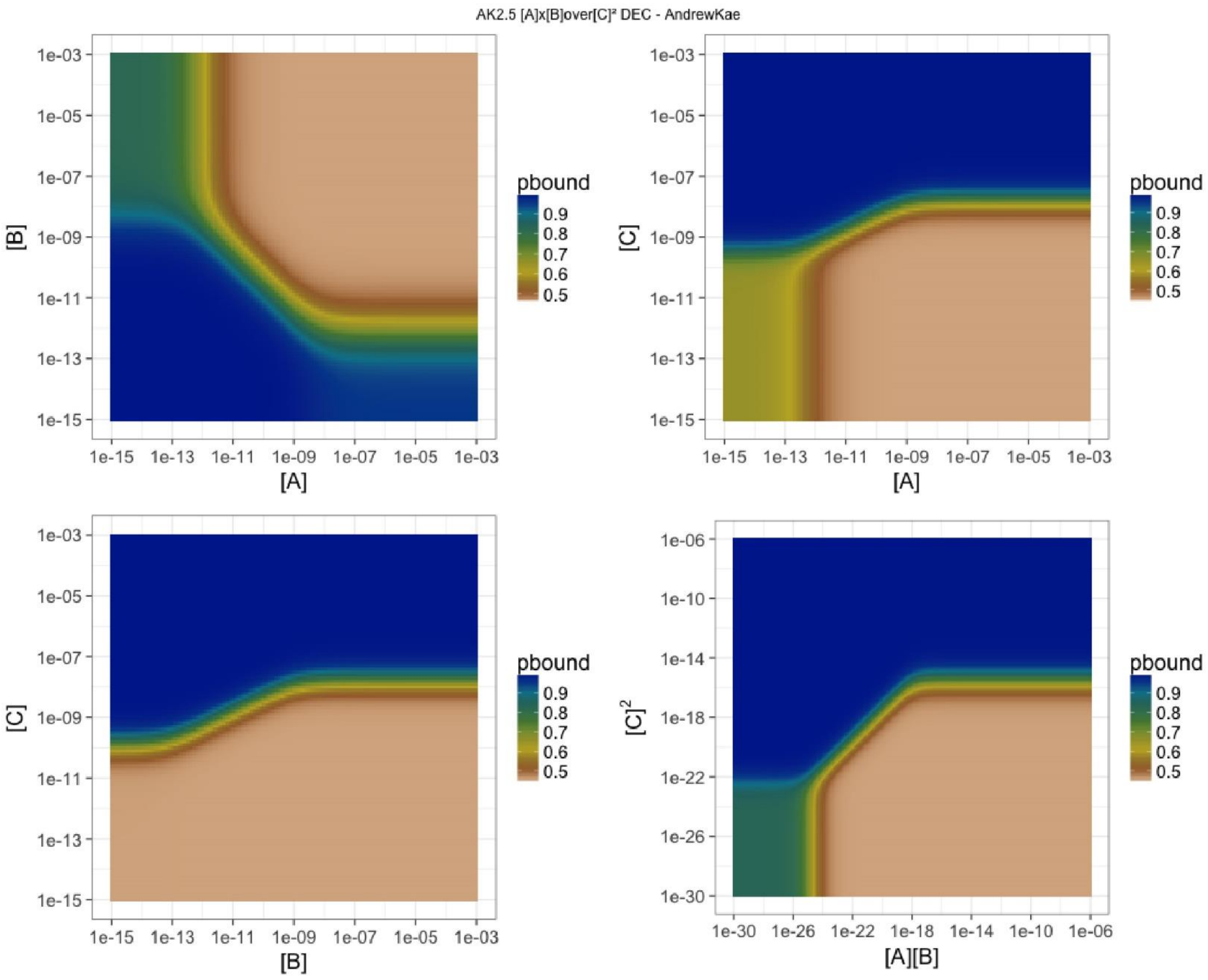

Supplemental Figure 3. On-line simulation plots for design AK2.5 submitted by AndrewKae. Simulation plots, made available to Eterna participants through a separate web server (code available at https://github.com/eternagame/conc_plots), showed the probability of binding the output reporter RNA at various concentrations of A, B, and C (given in M) as predicted by NUPACK. The simulated reporter RNA concentration was 1 nM. [C] = 1 nM in [A] vs [B]. [B] = 1 nM in [A] vs [C]. [A] = 1 nM in [B] vs [C].

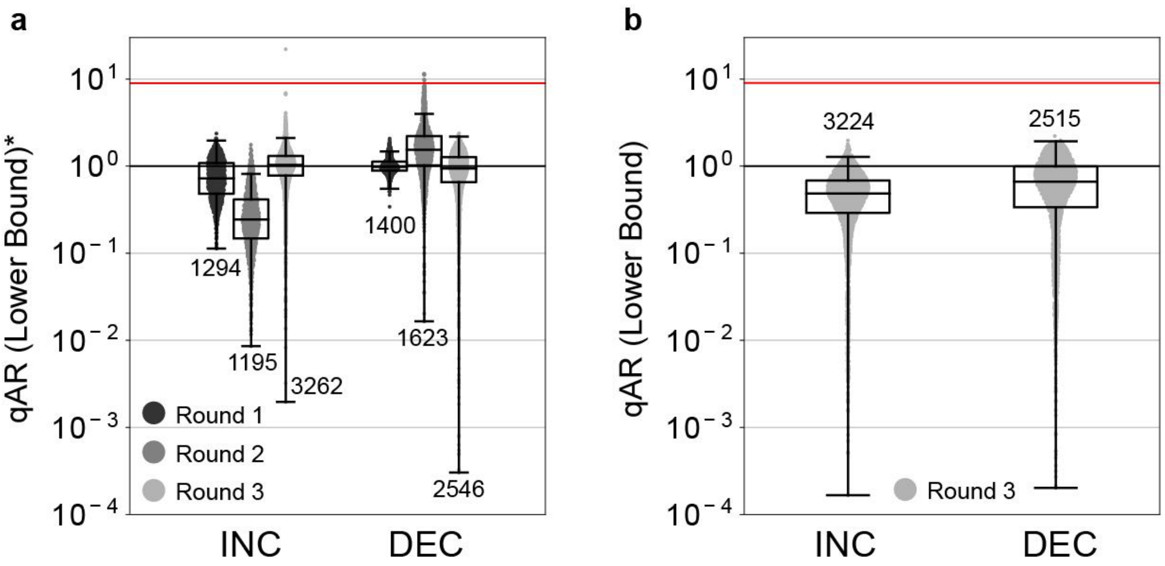

Supplement Figure 4. Quantitative activation ratios for Rounds 1 through 3 of Challenge 3: OpenTB sensors. a) Quantitative activation ratio shown is the lower bound with respect to the 4 conditions presented to players in Eterna, rather than over all conditions tested (12 or more; see **Supplemental Table 5**). To distinguish these values we use a * symbol. These qAR_{LB*} values and other experimental measurements were given to Eterna participants as feedback during the OpenTB challenge. **b)** Quantitative activation ratios for Round 3 designs with respect to the lower bound over all measured conditions (see **Supplemental Table 5**). The input and output RNA oligonucleotide sequences are the same as Round 1. Overall, the designs performed poorly, similar to Round 1. The red horizontal line is the theoretical maximum (AR_{LB*} = 9) for these four conditions. The centerline of the box plot indicates the median; the edges of the box correspond to the first and third quartiles; the whiskers extend to 1.5 times the interquartile range. The number of samples in each box plot is shown.

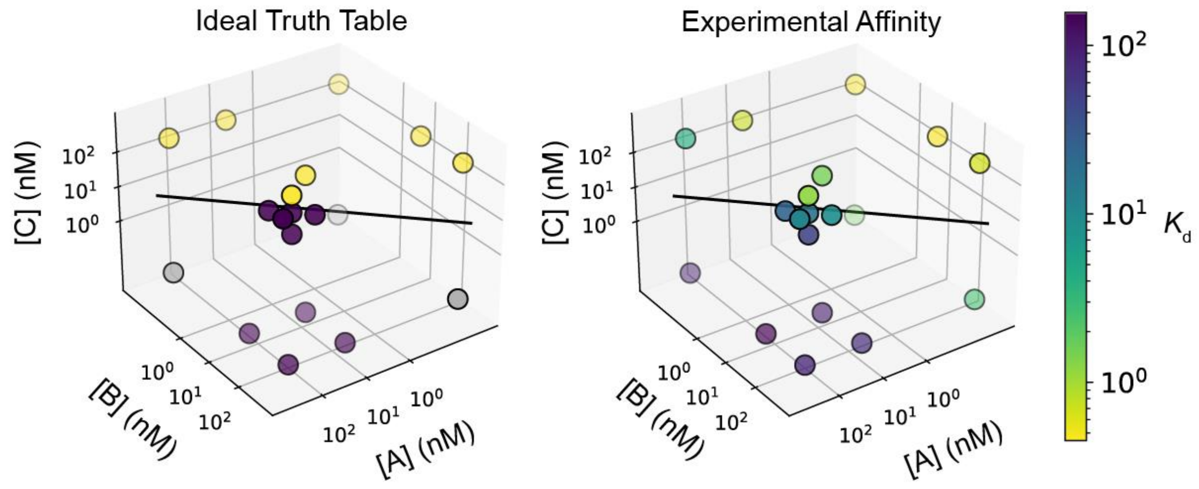

Supplemental Figure 5. 3D truth table for design AK2.2 from Round 2 of challenge 3: OpenTB calculator. 3D scatter plot of the ideal truth table (left) and experimental values (right). The separatrix plane for $[A][B]/[C]^2 = 1/16$ appears as a line due to the chosen view of the 3D plot, and gray points in ideal truth tables are undefined with respect to the three-gene TB-score.
