# Supplementary material for: Compact RNA sensors for increasingly complex functions of multiple inputs
Source: Nat Chem. 2025 Nov 12;17(12):1839–52. doi: 10.1038/s41557-025-01907-8 (PMC12669046; doi:10.1038/s41557-025-01907-8)
Supplement: Supplementary file 2 — Contains Supplementary Appendix: Eterna player resources (Eterna player resources also listed in Supplementary Table 4). Also contains the newsletters the Das lab shared with Eterna players after each design challenge. [file 41557_2025_1907_MOESM2_ESM.zip › supplemental_eterna_player_resources.pdf]

Supplemental Appendix for Eterna Player Resources for “Compact RNA sensors for increasingly complex functions of multiple inputs”

Christian A. Choe[1,a], Johan O. L. Andreasson[2,10,a], Feriel Melaine[3], Wipapat Kladwang[3], Michelle J. Wu[4,11], Fernando Portela[3,5], Roger Wellington-Oguri[3,5], John J. Nicol[3,5], Hannah K. Wayment-Steele[6], Michael Gotrik[3,12], Eterna Participants[5,b], Purvesh Khatri[7,8], William J. Greenleaf[2,c], Rhiju Das[3,4,9,c]

[1] Department of Bioengineering, Stanford University School of Medicine, Stanford, CA, USA
[2] Department of Genetics, Stanford University School of Medicine, Stanford, CA, USA
[3] Department of Biochemistry, Stanford University School of Medicine, Stanford, CA, USA
[4] Program in Biomedical Informatics, Stanford University School of Medicine, Stanford, CA, USA
[5] Eterna Massive Open Laboratory
[6] Department of Chemistry, Stanford University, Stanford, CA, USA
[7] Stanford Center for Biomedical Informatics Research, Stanford University, Stanford, CA, USA
[8] Stanford Institute for Immunity, Transplantation and Infection, Stanford University School of Medicine, Stanford, CA, USA
[9] Howard Hughes Medical Institute, Stanford University, Stanford, CA, USA
[10] Current address: Airity Technologies, Redwood City, CA, USA
[11] Current address: Verily Life Sciences, South San Francisco, CA, USA
[12] Current address: Protillion Biosciences, Burlingame, CA, USA

[a] These authors contributed equally: Christian A. Choe, Johan O. L. Andreasson.
[b] Consortium author. All contributors are listed in Supplemental Table 1.
[c] Correspondence to be addressed to wjg@stanford.edu and rhiju@stanford.edu.

Contents

1. Rational design of non-enzymatic RNA-based XOR logic gates
    - Boris Rudolphs (brourd), Fernando Portela (elnando8888)
    - Pages: 2-21

2. A Tutorial for A/B Ramp-up Puzzles
    - Roger Wellington-Oguri (omei), Eli Fisker
    - Pages: 22-86

3. Logic Gates Design Refinement Strategy
    - Jeff Anderson-Lee (jandersonlee), Eli Fisker
    - Pages: 87-118

4. Finding a balance for [A] times [B] over [C] squared designs
    - Andrew Kaechele
    - Pages: 119-140

Rational design of non-enzymatic RNA-based XOR logic gates

Boris Rudolfs[1,2,*], Fernando Portela[2,3,*],

This author order is completely tentative.

[*] Equally contributing authors.
[1] Department of Chemistry, Fresno State University
[2] Eterna massive open laboratory
[3] Department of Biochemistry, Stanford University

In preparation for submission to PLOS Comp Bio

Abstract

- Allosteric, single molecule computing has been proposed for a long time, but XOR not completed.
- Highlight introduction of ‘staple diagrams’ to aid in creation of templates.
- Massively parallel expts through eterna
- Sets stage for more complex computations

Introduction

Understanding how biological systems might be regulated through combinatorial control by multiple molecular inputs is a fundamental problem in both understanding molecular biology and in synthetic biology efforts to control living systems [cite?]. Often framed as biomolecular 'computation', design of complex logical operations has been an area of intense research in recent years. Simple Boolean logic gates like NOT, OR, AND, NAND (NOT-AND) and NOR (NOT-OR) have been successfully implemented using diverse biologically derived molecules like nucleic acids [1–3] or proteins [4]. Logic gates like the Exclusive OR (XOR) are crucial for more complex calculations such as digital addition, but due to their non-monotonic behavior, they are more difficult to implement. From a mathematical perspective, XOR logic gates can always be constructed using basic ones as "bricks" (for example, 4 interconnected NAND gates), and successful composite implementations of XOR and other complex logic gates and circuits have been reported through the cascading of multiple molecular components as varied deoxyribozymes, DNA strand displacement gates, transcriptional feedback, or genome editing [5,6].

However, this "small bricks" approach inevitably runs into various issues when it comes to building more ambitious large-scale logic circuits, like an 8-bit adder for instance (cite Foresight?). First, every logic gate introduces a delay as it accepts the inputs and propagates a result in the form of an output, and asymmetrically accumulated delays may require the additional complexity of a synchronization mechanism. Furthermore, the diffusive nature of the signals forces bio-circuit designers to attempt to reduce crosstalk (signal interference) as much as possible, and these problems become increasingly difficult to diagnose and troubleshoot as the number of signals within the solution increases with the complexity of the logic circuit. As an example, instantiation of DNA strand displacement circuits for the XOR gate require XXX strands that must be pre-mixed at exactly specified concentrations; it is difficult to see how to implement these molecular computers in real-world settings.

One solution to these problems is to integrate the computation on a single molecule rather than numerous mixed molecules. It was proposed several decades ago that allosteric interconversion between a molecule's multiple states, modulated in response to binding of external ligands, might be able to instantiate basic computations {cite Monod, 'Chance and Necessity}. Nucleic acid molecules interconverting between secondary structures offer a compelling substrate for which *in silico* modeling tools are available and have seen successful for simple AND and OR gates {Penchovsky,Breaker}. Nevertheless , limitations in these modeling methods, throughput of experiments, and unavailability of empirically tested design rules have precluded development of single-stranded, non-enzymatic, and energy-efficient nucleic acid devices with increased complexity, including XOR gates. With the advent of increased experimental throughput and attention focused on RNA design, we hypothesized that it would be possible to engineer complex logic gates like XOR and XNOR. Beyond the mere proof of existence of such solutions, we aimed at either generalizing those we could eventually find manually, or deriving a broadly applicable method from purely theoretical considerations. Through use of a 'massive open laboratory' design platform [9], we were able to model the XOR problem in silico, derive a sequence-independent heuristic method for generating candidates, and then rigorously test the success of this method with high-precision experimental results.

Material and methods

Eterna design and submission interface

The citizen-scientists members of the Eterna [10] community were challenged to submit suitable designs for a range of logic gate targets (limited to AND, OR and XOR in round 98, and a complete set of binary boolean operators in round 102). the graphical user interface allowing for free placement of the MS2 consensus sequence signal within the 85 nucleotides fixed-length design space. All logic gate puzzles in aforementioned synthesis rounds were modeled with the same two miRNA-like oligonucleotide inputs (5'-ACCCCACAAUAAAGAAUAAG-3' and 5'-CUAAGCAGUUCCCUCAUU-3', called 'oligo 1' and 'oligo 2' below) while the output was specified through the conformation of an MS2 aptamer consensus sequence (5'-ACAUGAGGAUCACCCAUGU-3'). In line with experiments testing for binding of the MS2 virus coat protein to this sequence (see below), formation of the correct hairpin secondary structure [in dot-parentheses notation: "(((((.((....)))))))"] was interpreted as "ONE" or "TRUE". Misfolding or absence of this secondary structure was interpreted as "ZERO" or "FALSE". Minimum free energy computations for multistrand targets were performed in real-time in the Eterna 'game interface' using the NuPACK folding engine [11]. (As briefly discussed below, we and other Eterna members made use of other heuristics or full-fledged partition function calculations to help discover or rank solutions, but these functionalities were not embedded within the Eterna interface.)

Sequence generation scripts

We took advantage of the built-in scripting ability of the Eterna platform to encode a simple candidate generator [12]. The implementation was straightforward, combining strict rules from the DMSSD protocol with a measure of randomness, among other things, varying the direction of application of the heuristic 5'-3' or 3'-5', and the order O-X or X-O of the oligonucleotides binding sites. A batch of generated candidate sequences were submitted unfiltered for the XOR target of round 98. Another batch of submissions for both XOR and XNOR targets of round 102 were selected by taking the 100 best performing candidates from a pool of 500 generated valid sequences, according to the following scoring formula:

where XXX and YYY are the MS2 hairpin formation probabilities as computed by the partition functions of each of the 4 different states.

Experimental tests

Experiments were carried out as part of the Eterna massive open laboratory platform, using high-throughput RNA arrays as described in [13]. Briefly, sequences submitted for XOR puzzles [14,15] were synthesized as part of 12k array-based DNA oligonucleotide libraries (CustomArray, Bothell, WA); amplified by PCR; arrayed on Illumina Miseq chips; and transcribed into RNA on-chip using E. coli RNA polymerase, with DNA oligos sequestering portions of the RNA sequence not in the designed sequence {Buenrostro,2014}. The primary data collected were dissociation constants of the MS2 coat protein with aptamer hairpins encoded by the designs (**Table 1**); these values were derived from the intensity of fluorescence generated by sequence clusters at different concentrations of labeled MS2 coat protein. Curves were repeated with and without 100 nM of input oligonucleotide sequences A and B (**Table 1**). The ratio of the lowest measured dissociation constant for the OFF condition and the highest measured dissociation constant for the ON condition – i.e., the 'worst-case' conditions – was taken as the measured fold change for the system. For further details, see {Andreasson, et al.; Supporting Information for Review}.

Results

Design Heuristics for RNA Switches

The XOR logic gate design problem, which was presented on the Eterna website as a puzzle with the following specific constraints:

- the design length was set at exactly 85 nucleobases long
- 19 of the design nucleotides had to be the MS2 consensus sequence (playing the role of an allosteric output)
- the design should respond to two short RNA strands (‘oligos’) mimicking miRNAs or mRNA segments played the role of the inputs. Four input conditions were simulated: no oligos present, 100 nM of oligo 1, 100 nM of oligo 2, 100 nM of both oligos.
- the MS2 hairpin should be folded or unfolded according to the desired logic gate in the four input conditions of the problem.
- No more than 4 A’s, C’s, G’s, or U’s in a row could be given (to help ensure synthesis).

This problem proved to be difficult to solve in silico, and several strategies were developed in order to solve the problem. Initial strategies for finding solutions focused on the duplication of the MS2 hairpin aptamer or the oligonucleotide binding sites. Both approaches were attempted continuation of work previously attempted in Eterna on simpler problems: the binding site duplication was an indirect consequence of applying the strand-displacement technique [6] used in earlier simple sensor-type Eterna experiments and similarly, the double MS2 aptamer was inspired by another earlier Eterna experiment seeking binding cooperativity for two MS2 proteins. However, these early solutions were unsatisfactory, as they failed to meet some of the generality criteria we were striving for, including independence of the strategy from fortuitous similarities or complementarities in the MS2 and input oligo sequences.

After the explorations above, we developed a strategy that we subjected to extensive testing for single-stranded XOR logic gate design. We named this strategy **D**omain **M**atching **S**econdary **S**tructure **D**esign (DM-SSD). It is a constraint-driven method using predefined domains to design secondary structures that fulfill the logic conditions of a desired ordered system. As a simplified example, Figure (X-A) showcases an RNA sequence interconverting between a structure that forms a hairpin loop, and a structure that binds the MS2 protein. With the MS2 hairpin as our desired structure, we can assign domains to this condition, which we will denote as M, and the two unstructured regions 5’ and 3’ of the aptamer, which we will denote as N1 and N2. Within the context of our single stranded system, canonical base pairing is used to control the formation of the MS2 hairpin and a subdomain within the domain 3’ of the MS2 hairpin that basepairs to the MS2 sequence, N2_subM, acts to sequester the MS2 hairpin and prevents the MS2 protein from binding.

However, more stringent controls are necessary to ensure the selective switching that occurs between these two domains. Figure (X-B) is a single input device that showcases the binding of an oligonucleotide to control this switching behavior. Two additional domains are assigned to the sequence, 3’ of the MS2 hairpin. The first domain is denoted as N2_subO, which is complementary to a

sequence 3' of the N2_subM domain, denoted as domain O. This final domain is complementary to an arbitrary oligonucleotide input, and controls the dynamic behavior of the RNA switch. In the presence of the oligonucleotide, domain O is sequestered, which prevents the canonical base pairing between domains N2_subO and O. This allows the sequence of N2_subM to base pair with domain M, controlling the binding of the MS2 coat protein to its respective hairpin. We can then apply these logical rules of complementarity and sequence exclusion through hairpin loops to more complicated systems, including an XOR logic circuit.

Strategy for Design of XOR logic gates

The approach above was applied to define domains and secondary structures that would instantiate an XOR gate. The single stranded RNA was partitioned into seven domains (**Figure 1**). Three domains O_sub1, O_sub2, and M harbored sequences complementary to oligonucleotide input 1, oligonucleotide input 2, and the MS2 hairpin aptamer domain, respectively. Two additional domains, N1 and N3, encoded separate functions involving intramolecular Watson-Crick base pairings, and were positioned between the defined regions. Additional domains denoted as N2 and N4 were explored, but were not necessary for the strategies herein and are not discussed further.

For our sequence independent method of design, the N1 and N3 domains encoded sequences complementary to the MS2 aptamer sequence to fulfill the condition of no signal in the presence or absence of both oligonucleotide inputs (Strat Schematic Fig). Domain N1 is analogous to domain N in the single-input sensor above; it directly 'locks' the MS2 sequence M unless oligo input 1 is present. Domain N3 is further divided into three subdomains which carry separate functions. Domain N3m is imperfectly complementary to domain N1 and the MS2 aptamer; it serves a dual purpose, turning on or off the MS2 aptamer region, depending on the condition. Domain N3x is imperfectly complementary to domain X and acts to lock away domains of the system that would normally respond to oligo input 2 ???. Domain N3o is imperfectly complementary to domain O and acts to guide N3m to turn off M when both oligo inputs are present ???.

In the total absence of oligonucleotide inputs, the N1 and M domains base pair to initiate the OFF condition (staple level ??? in Fig. ???). The N3x subdomain base pairs with the X domain to prevent interference of the N3m subdomain with domain N1.

In the condition where oligonucleotide input 1 alone is present, it binds to domain O, breaking the base pairing interaction between the M and N1 domains, releasing M to form its aptameric secondary structure. The RNA is ON. In this case, the N3x domain continues to base pair with domain X, sequestering the N3m domain complementary to the aptameric region.

In the condition where oligonucleotide input 2 alone is present, domain N3o base pairs with domain O, and domain N3m base pairs with domain N1, and the M domain folds into its hairpin structure.

Finally, in order to initiate the OFF condition in the presence of both oligonucleotides, both inputs form heterodimers with their respective domains. This prevents subdomain N3m from base pairing with domain N1, subdomain N3o from binding with the O domain, and subdomain N3x from base pairing

with domain X. This releases subdomain N3m to base pair with the M domain, which inhibits the formation of the MS2 hairpin.

An important feature of the above strategy is that each domain's function requires it to base pair with at most two other domains, depending on the input condition. This feature breaks the problem down to finding sequences for each domain that each need to satisfy a few constraints; their 5' to 3' arrangement then helps guarantee that if the constraints are satisfied for each domain, the overall design constraints are likely to be satisfied as well.

The strategy described above guided the straightforward programming of a simple automated sequence generator, implemented as a plugin-script within the built-in scripting interface of the Eterna platform. As an example of a specific sequence and pairings designed by the DMSSD approach, **Figure 3** shows a design BXM 28 that ended up performing accurately in *in vitro* experiments (see below). The design contains all 7 domains, highlighted in different colors in **Fig. 3**. Multistrand folding calculations in NUPACK suggested that the desired pairings amongst domains, which interconvert in the 4 conditions of the logic gate, would occur as expected in the DMSSD design strategy (compare arcs in **Fig. 3** to **Fig. 1**).

Experimental Results

Using the DMSSD strategy, 66 sequences were generated for an XOR biomolecular logic gate by a simple script (Materials and Methods) and collected for the 98th round of the massive open laboratory platform, Eterna. Comparisons to submissions by other Eterna participants show that the measured fold change of the sequences designed by the automated script were comparable to those made by humans, or better in some instances, with a measured maximum fold change of 7.02 for design BXM 28 (**Figure 3 & 4a**).

Using the strategy template a second time, a combined 200 sequences were generated for XOR and XNOR biomolecular logic gates by a script and collected for the 102nd round of Eterna's massive open laboratory. The DMSSD domain template for XNOR is given in **Supporting Figure S2**. In this second round, we tested an additional *ad hoc* strategy to select for designs derived from XXX with MS2???. The strategy generated sequences for the two logic gates were chosen for their predicted success in NUPACK (cite? What is the metric used?).

In terms of maximum fold-ratio performance, the XOR logic gate designs gave improvement over the previous round for the XOR logic gates, with a measured maximum fold change of 22.40, a 3-fold increase over the previous round **(Figure 4b)**. This improvement however was likely attributable to having more design slots (100 compared to 66); there was no clear correlation between the new metric tested and performance of the designs (Supplemental Figure S1). In comparison, players of Eterna gave a maximum fold change of 59.85 **(Figure 4b)**. While those top performing designs were not directly outputted from DMSSD, they contain the elements of the DMSSD strategy and many were modifications of DMSSD sequences from the previous round that retained the 7 domain architecture **(Figure 5 – show alignment of colored domains)**. In addition, Eterna players submitted a series of single nucleotide mutations of BXM 28, with one mutation having a measured maximum fold change of 46.62, 6.6 times the activity of the wild-type sequence **(Figure 4c)**.

DMSSD gave measured maximum fold change of 8.39 for the XNOR logic gates **(Figure 4d)**. In comparison, players of Eterna had a maximum fold change of 59.85 and 86.88 for the XOR and XNOR logic gates, respectively.

- Experimental results -- first XOR [see Brourd's results summary] figure 4 (1 panel with scores vs. author; another panel conveying the architecture of all solutions)
    - None of the XOR gates were particularly effective, and most player submitted designs were modifications of sequences made from DMSSD derived secondary structures and sequences. [need to show this]

- Experimental results XOR 2nd round and XNOR [see Brourd's results summary] figure 5

Tests of generality in silico

We tested the generality of the DMSSD method by first varying oligonucleotides inputs. The algorithm produced candidate designs after only a few iterations for all possible pairs of inputs chosen from a set of 6 miRNA-like oligonucleotides. We also tested the same pairs of possible oligonucleotides inputs in combination with the Kink turn RNA motif, which is known target motif for the L7Ae ribosomal protein. For all combinations, candidates were generated rapidly as well. **(Table X?)**

We also tested a different type of output in the form of a randomly chosen 12-mer RNA oligo. The direct application of the pattern of bindings described in Fig X consistently failed in this new context. We rapidly realized that the failure was caused by the inability of the Eterna graphical interface to handle pseudoknot-like binding patterns. However, a different DMSSD strategy was devised to provide a successful in silico solution (Supplementary Fig Z?)

Discussion

(write this last)

Using simple heuristics, we developed a method for the in silico design of simple RNA-based devices that are capable of exhibiting the behavior of XOR logic gates, which may act as elementary units of more complex computational circuits. These heuristics may also be extrapolated to even more complex systems, which may aid in the design of devices that may have diagnostic or therapeutic applications. However, the in vitro efficacy of the strategy alludes to several limitations. In particular, the strategy merely provides a blueprint of the secondary structure that an RNA sequence could adopt to fulfill the logical specifications of a design problem. The information on the optimum length of helices, the composition of base pairs, and other non-trivial aspects of designing these RNA devices is not entirely encoded within the domains of this method. Furthermore, the method ignores pseudoknot structures and long range tertiary contacts which may act to stabilize the intermediate states of these devices, and may explain to some degree the low percentage of successful designs (citation needed?).

Experimental Efficacy

DM-SSD was used to design an XOR logic gate, secondary structure architecture with nearly complete input independence **(Table X),** and the addition of expert human strategies enabled the design of sequences that were experimentally verified to act as XOR logic gates in vitro. One single nucleotide mutation of the design with the highest fold change from Eterna round 98, BXM 28, had a 6.6 times increase in fold change, and three of the SNP's had fold change measurements greater than that of the round 102 designs. Each of these . The versatility of the heuristic indicates that the in silico design of XOR logic gates using the parameters specified here could allow for the near universal design of these logical architectures, and the potential for in vitro design with more robust metrics and strategies for the design of sequences and secondary structure. In addition, given the method of the heuristic, there is an intriguing possibility for the automation of finding other XOR architectures within the same structure space. One such architecture was discovered after the experimental testing of these sequences, which involved multiple binding sites for one of the inputs and worked by displacing the formation of helices that sequester the oligonucleotide binding sites (Figure of second architecture?).

Furthermore, the concept of assigning domains to functional sequences within an RNA molecule may be applied to other structured regions of riboswitches, mRNA and long non-coding RNAs, which could provide insight into the logical design of these naturally occurring nucleic acid machinery. Extraction of these logical rules could provide a basis for the search and discovery of other RNA molecules that exhibit this structured behavior. Granted, other, simpler logic gates, such as the AND gate or OR gate, may contain far greater numbers of architectures that fulfill the logical requirements of the system, and provide some difficulty in developing a search criteria for comparison against.

• Naming of domains is strikingly similar to nomenclature used in early dissection of gene regulation (OR1, OR2, OR3, cro, N, Q, etc.; see Ptashne, “A genetic switch”).

• Identification of domains based on interactions even in designs that did not explicitly use the strategy suggests that they may be universal/general – might be able to discover XOR/XNOR's in untranslated regions of mRNAs or long non-coding RNAs by looking for domains with these arrangements & properties.

Then describe how one might get to more complex computations

Conclusion (tbd)

- potential applications
  medical: “clever” diagnostics, etc
  theoretical: biomolecular computations, half-adders and full-adders
- the technique is most likely applicable to DNA-based computations as well
- future targets in silico (?)
- Future work: composability
- a note about the power of the crowd, EteRNA and players as biomolecules designers
  (of the ingenuity of non-scientist designers, and the advantages of gamification)

Acknowledgments

References

1. Stojanovic MN, Mitchell TE, Stefanovic D. Deoxyribozyme-Based Logic Gates. J Am Chem Soc. 2002;124: 3555–3561. doi:10.1021/ja016756v

2. Gerasimova YV, Kolpashchikov DM. Connectable DNA logic gates: OR and XOR logics. Chem Asian J. 2012;7: 534–540. doi:10.1002/asia.201100664

3. Bhadra S, Ellington AD. Design and application of cotranscriptional non-enzymatic RNA circuits and signal transducers. Nucleic Acids Res. 2014;42: e58. doi:10.1093/nar/gku074

4. Baron R, Lioubashevski O, Katz E, Niazov T, Willner I. Logic gates and elementary computing by enzymes. J Phys Chem A. 2006;110: 8548–8553. doi:10.1021/jp0568327

5. Buchler NE, Gerland U, Hwa T. On schemes of combinatorial transcription logic. Proc Natl Acad Sci U S A. 2003;100: 5136–5141. doi:10.1073/pnas.0930314100

6. Qian L, Winfree E. Scaling up digital circuit computation with DNA strand displacement cascades. Science. 2011;332: 1196–1201. doi:10.1126/science.1200520

7. Miyamoto T, Razavi S, DeRose R, Inoue T. Synthesizing biomolecule-based Boolean logic gates. ACS Synth Biol. 2013;2: 72–82. doi:10.1021/sb3001112

8. Wu C, Wan S, Hou W, Zhang L, Xu J, Cui C, et al. A survey of advancements in nucleic acid-based logic gates and computing for applications in biotechnology and biomedicine. Chem Commun . 2015;51: 3723–3734. doi:10.1039/c4cc10047f

9. Lee J, Kladwang W, Lee M, Cantu D, Azizyan M, Kim H, et al. RNA design rules from a massive open laboratory. Proc Natl Acad Sci U S A. 2014;111: 2122–2127. doi:10.1073/pnas.1313039111

10. Eterna - Invent Medicine [Internet]. [cited 22 Aug 2016]. Available: http://www.eternagame.org/

11. Zadeh JN, Steenberg CD, Bois JS, Wolfe BR, Pierce MB, Khan AR, et al. NUPACK: Analysis and design of nucleic acid systems. J Comput Chem. 2011;32: 170–173. doi:10.1002/jcc.21596

12. Fernando Portela. BXMG - XOR candidate sequences generator. In: GitHub Gist [Internet]. [cited 22 Aug 2016]. Available: https://goo.gl/eh4a0n

13. Buenrostro JD, Araya CL, Chircus LM, Layton CJ, Chang HY, Snyder MP, et al. Quantitative analysis of RNA-protein interactions on a massively parallel array reveals biophysical and evolutionary landscapes. Nat Biotechnol. 2014;32: 562–568. doi:10.1038/nbt.2880

14. Eterna - The Real Logic Challenge using NuPACK (R98) - XOR Logic Gate [Internet]. [cited 22 Aug 2016]. Available: http://www.eternagame.org/game/puzzle/6096397/

15. Eterna - Logic Gates (R102) - XOR (FTTF) [Internet]. [cited 22 Aug 2016]. Available: http://www.eternagame.org/game/puzzle/6434627/

Other possible sources:

- I think there's another XOR paper from Ron Weiss's lab (in cellulo even!) -- rhiju.
- Yurke B, Turberfield AJ, Mills AP Jr, Simmel FC, Neumann JL. A DNA-fuelled molecular machine made of DNA. Nature. 2000 Aug 10;406(6796):605-8. doi:10.1038/35020524
- Paul's thesis: http://www.dna.caltech.edu/Papers/pwkr_thesis_nov15.pdf
- http://cacm.acm.org/magazines/2012/12/157881-theory-of-algorithmic-self-assembly/abstract has a cool video
- https://digamma.cs.unm.edu/wiki/bin/view/McogPublicWeb/MolecularLogicGates [link broken]
- https://biobricks.org/bpa/contributions/57/ (BIL boolean integrase logic)
- Recent very interesting paper by DePace and colleagues – totally different way to create XORs: http://biorxiv.org/content/biorxiv/early/2016/02/10/039339.full.pdf
- In-silico design of computational nucleic acids for molecular information processing https://jcheminf.springeropen.com/articles/10.1186/1758-2946-5-22

Analysis docs:
XOR puzzle solving strategy
XOR + XNOR Results Discussion

XOR R98/R102 fold change vs MS2 probabilities
XOR design topologies

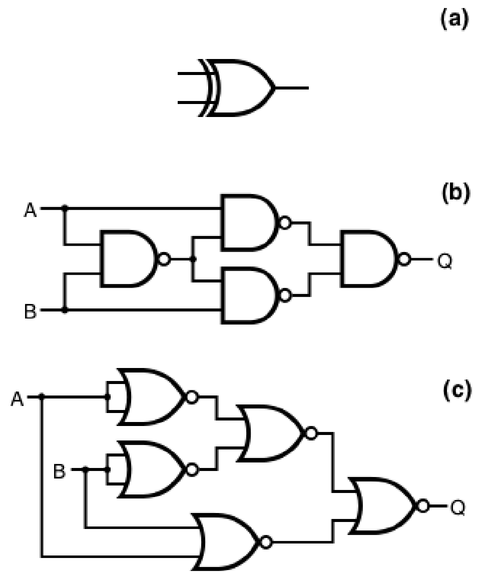

(a) ANSI symbol for the XOR logic gate. Functionally equivalent constructs made of (b) 4 NAND gates and (c) 5 NOR gates.

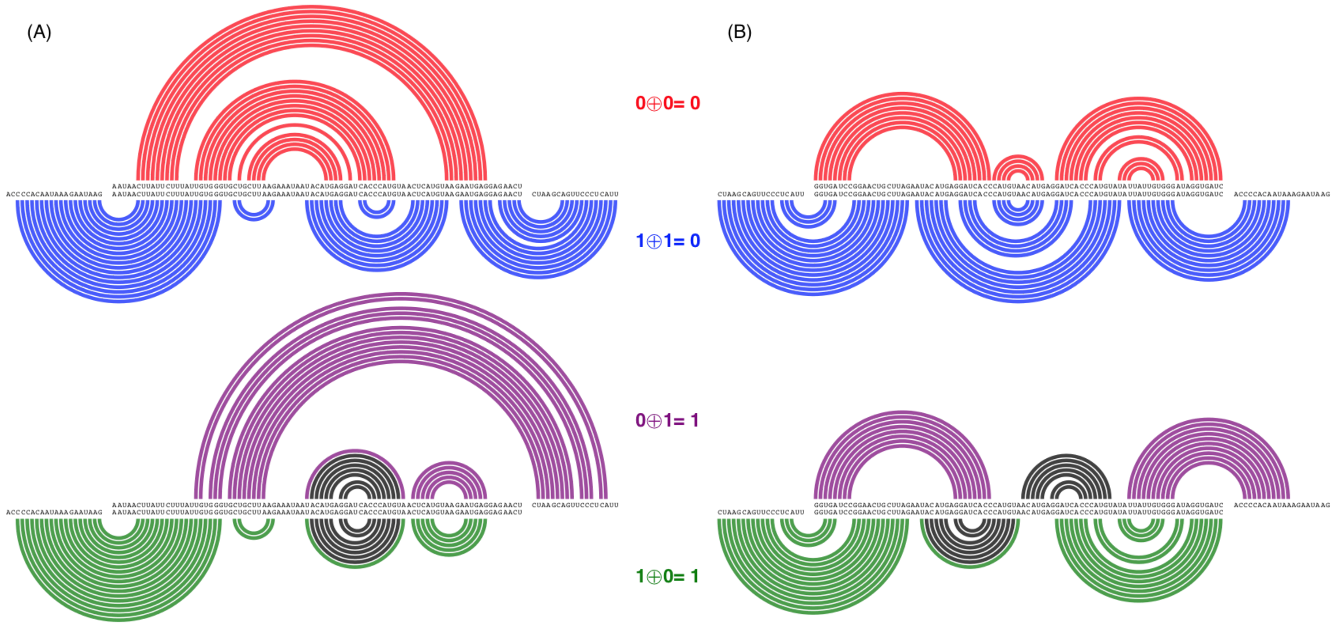

Early XOR solutions. In (A) one of the oligos has 2 possible binding sites, and of of them “collides” with the binding site of the other, inducing competition. (B) has a double copy of the MS2 sequence.

Image of the Basic Secondary Structures and the Domain Base pairing schema for XOR (needs to be revised with colors universal to Nando's images)

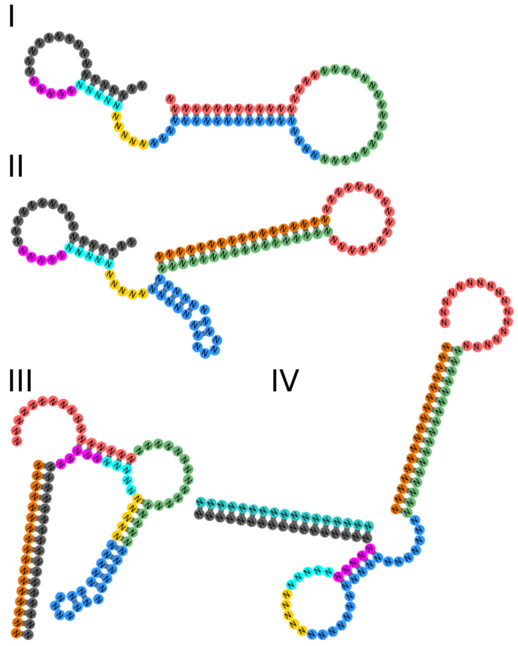

Schematic of the strategy for protein-binding output signals

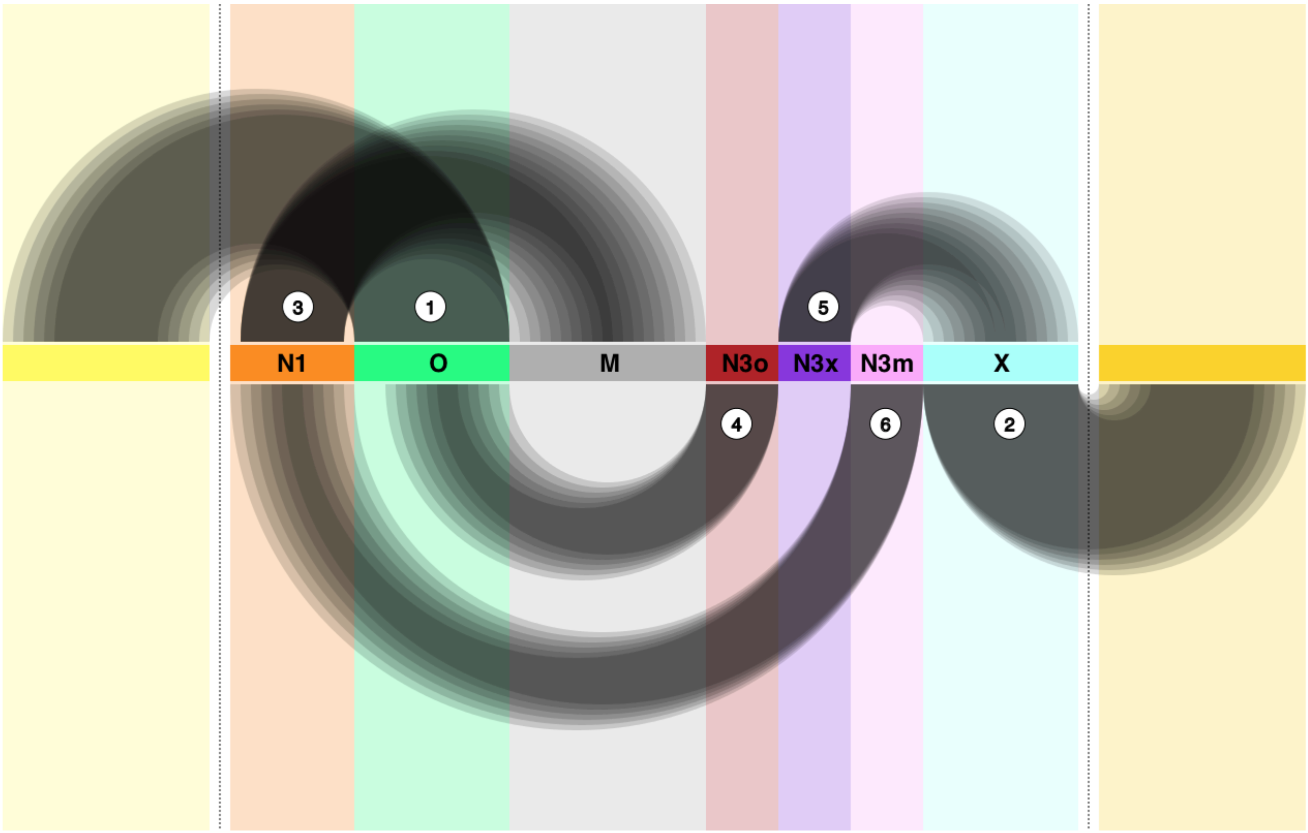

1. match (well) domain O to the first oligo
2. match (well) domain X to the other oligo
3. match (imperfectly) domain N1 to the MS2 domain
4. match (imperfectly) sub-domain N3o to the closest half of the O domain
5. match (imperfectly) sub-domain N3x to the X domain
6. match (imperfectly) sub-domain N3m to the N1 domain

XOR non-competitive binding (BXM28), 3rd iteration

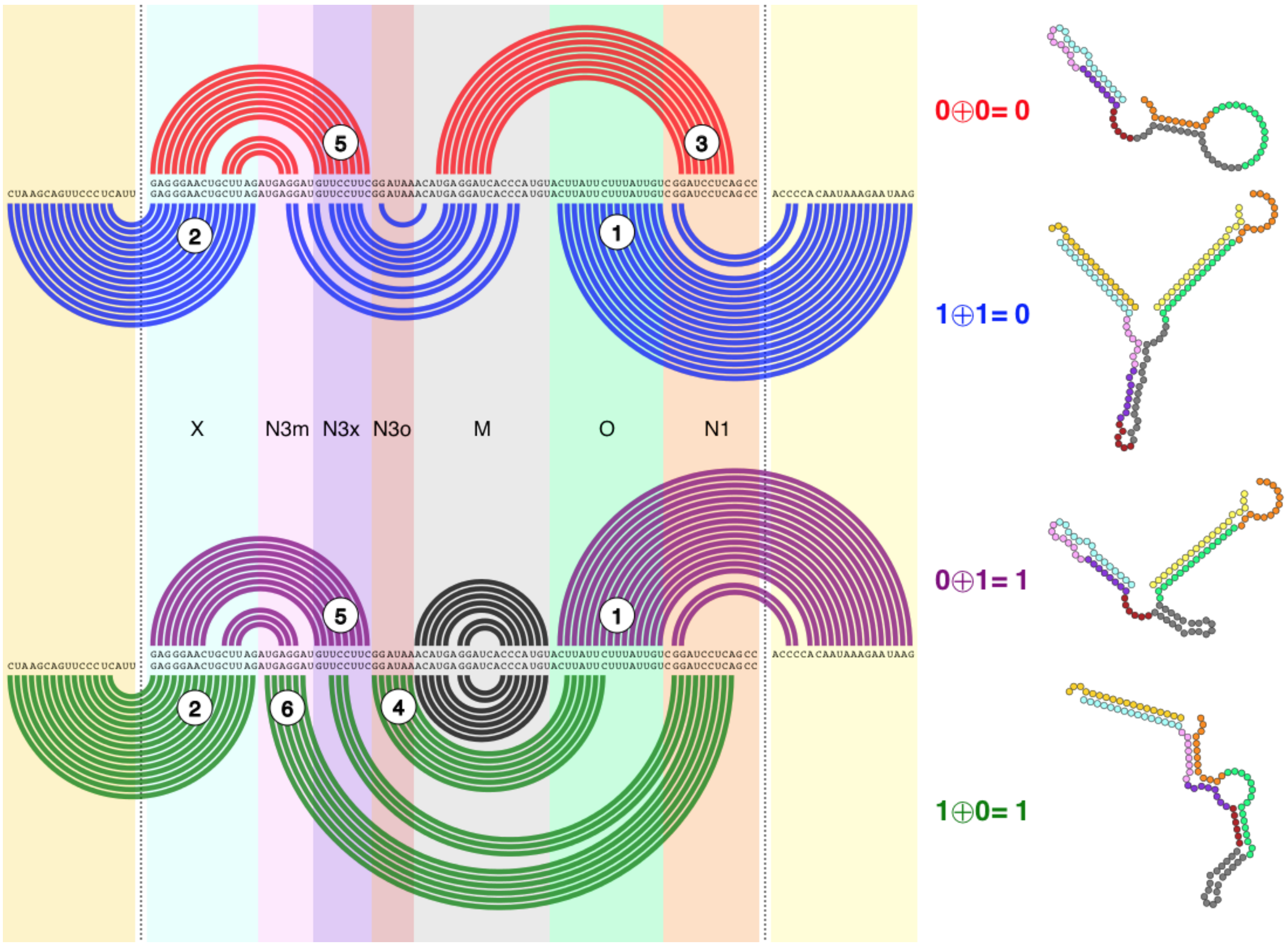

(in this case, algorithm was applied in the 3'→5' direction)

Target XOR

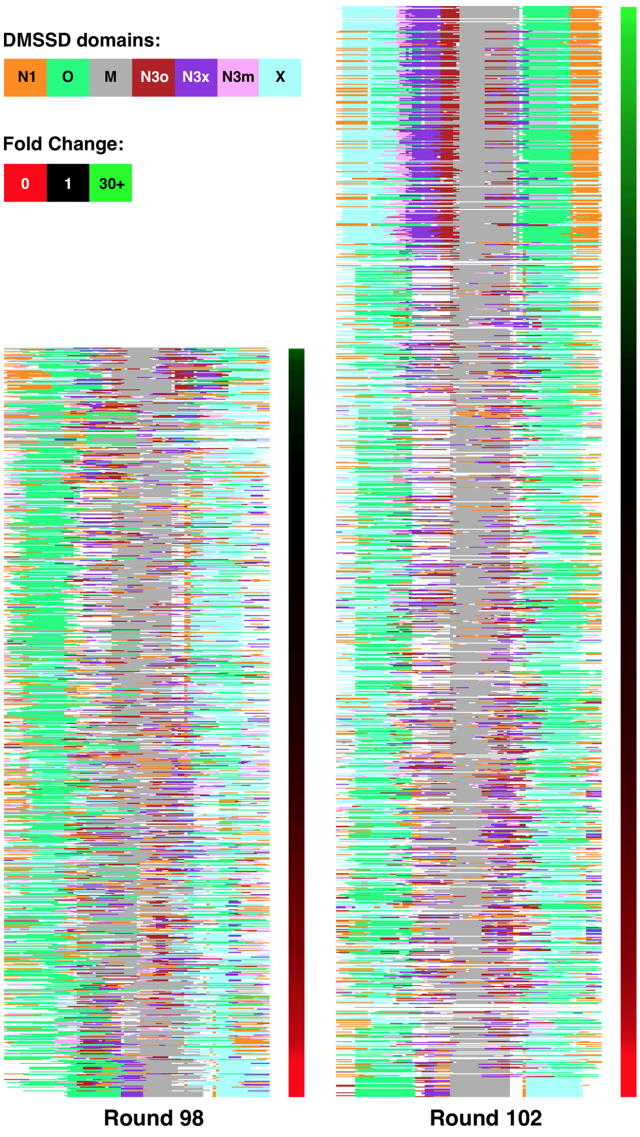

DMSSD domains:
N1
O
M
N3o
N3x
N3m
X
Fold Change:
0
1
30+
Round 98
Round 102

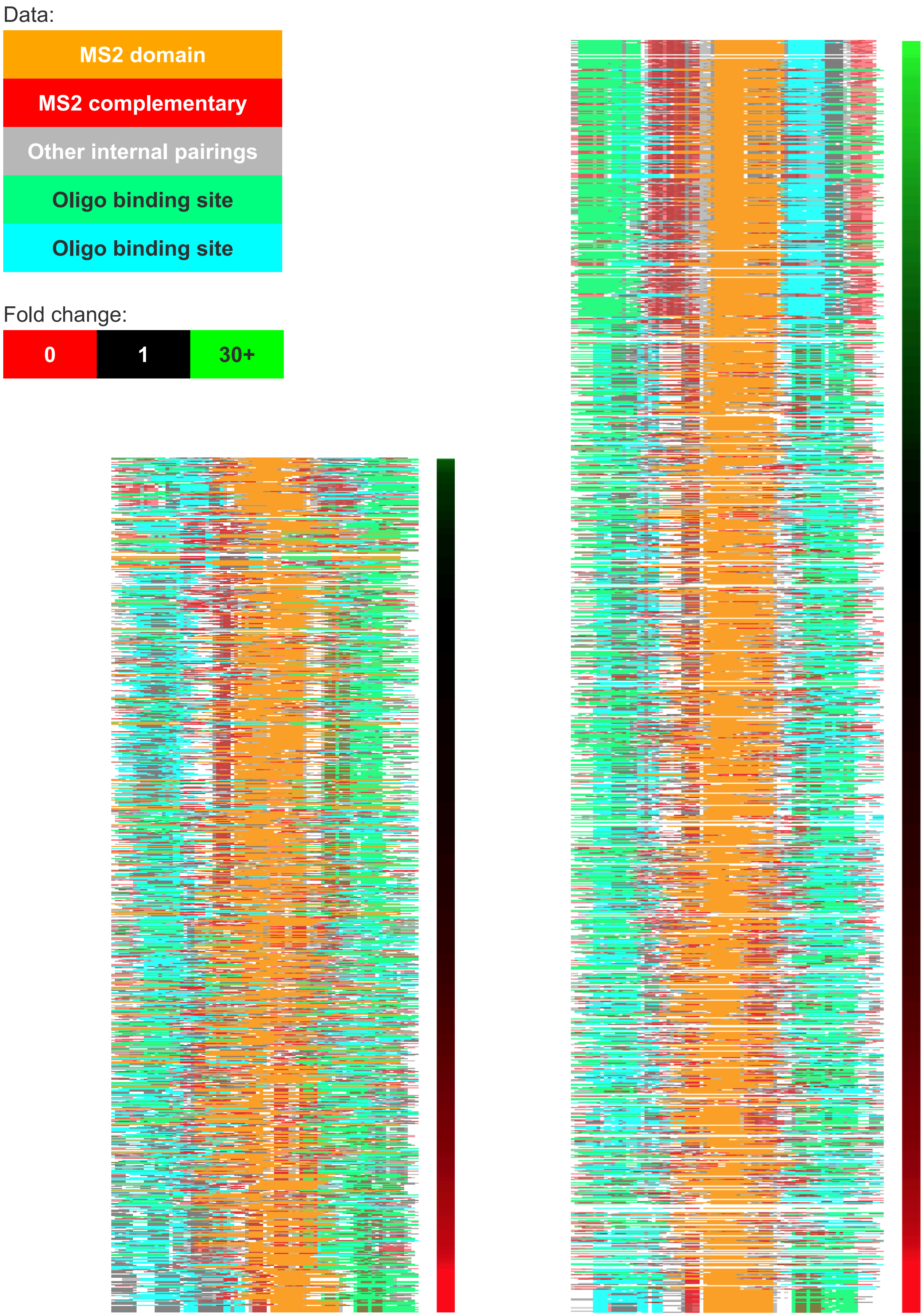
Data:
MS2 domain
MS2 complementary
Other internal pairings
Oligo binding site
Oligo binding site
Fold change:
0
1
30+

RNA motif	MS2 hairpin							Kink turn					
Inputs	A1	B1	C1	A2	B2	C2		A1	B1	C1	A2	B2	C2
A1		28	26	31	3	17			14	174	185	119	79
B1			13	28	23	20				151	39	101	151
C1				82	15	65					128	129	62
A2					3	20						42	121
B2						35							16

A1 = ACAGCUCAGCACAACAUUCC, B1 = GUUGGUGCCUUUUGUGCCAC, C1 = UUUUGGGCUACCUGGUUCGU
A2 = ACAGAUGCAGGAACAGGCUG, B2 = CCAUGGUGAUGGAUGGUUUG, C2 = GUACAUAGAGAGACAGGUGG

XOR example with reporter oligo

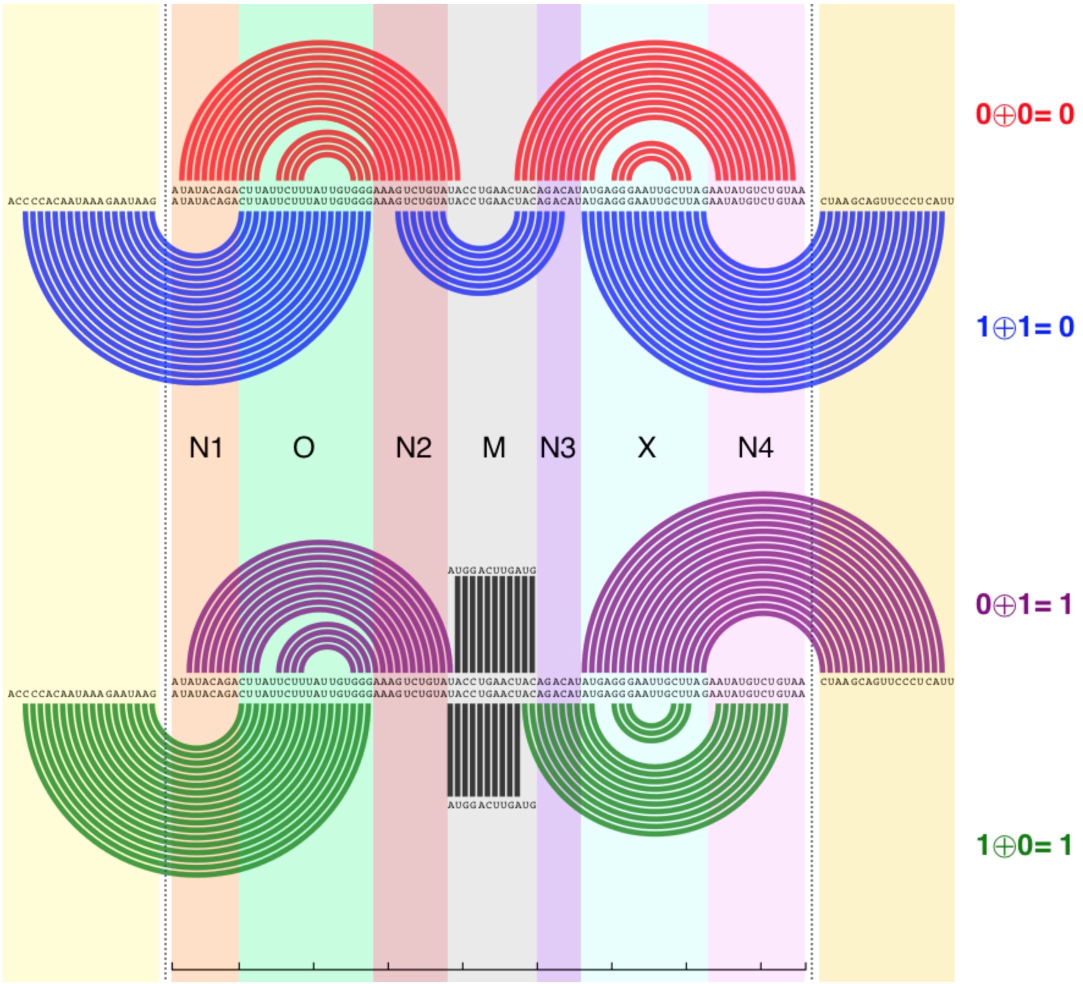

Action items (8/3 for 8/9)

- ~~Explore arc diagram and/or secstructs to illustrate 'attempt 1', 'attempt 2', and/or 'final'. [**nando**]~~
- Literature search to find all XOR's → can we get all PDFs in one Google Drive? **[boris?]**

Action Items (8/15/16)

Nando

~~• material & methods -- need some text from nando. does not have to be detailed~~

• ~~results section 1: need 1-2 sentences from nando on script though.~~

• ~~results section 'tests of generality': nando fills out. might be worth a figure. really lookin forward to reading this -- it was a great idea to test alternative inputs.~~

~~• results section 'tests of generality': test alternative output (" the same 10-mer reporter we used in a different lab?")~~

Brourd

• fill out results section 1 (DMSSD explicit strategy).

• for expt results:

there's one critical thing missing, which is to see if other designs used similar 7-domain elements as DMSSD (and whether some were mods!). OK -- would it be possible for you to take a look at, say, the top 20 from each round, and make some notes on whether they were DMSSD, DMSSD-dreived, DMSSD-like, or totally new?

Brourd: could you document that for the top 20 solves for XOR-R98, XOR-R102, and XNOR-R102?

could be very rough notes, just put in some doc somewhere. we need to do that due diligence and then summarize here does not have to be super fancy

Rhiju

• Make contact with other XNOR (R102) submitters to see if they had distinct strategy; if not, can claim here that all was DMSSD-derived. If yes, ask them if they're interested in writing separate paper, and 'punt' in this paper about DMSSD generality. Either result is OK!

What's next

Next meeting will be on Monday, ~8/22

• The goal then would be to spend 1-2 hours and really remove all this extraneous crap (comments, action items, etc.) to a near-complete draft.

• including draft figures (probably about 5 for main text), and 1-2 for an appendix or supplemental information.

you can probably see that it will take about 2 hours

• then send a note to me (rhiju), and i can try to do a full pass near the middle of next week (probably wednesday or thursday, 8/24 or 8/25)

A Tutorial for A/B Ramp-up Puzzles

Omei and Eli

This is a guide which describes a method for designing riboswitches of the type we are testing for Eterna Medicine. It is not the only way players design these switches, not every design that can be generated in this way will score well in the lab, and not all successful design can be generated using this method. However, it seems that many, if not most, of the successful switches that have been found to follow the pattern of designs that this method generates.

This document uses the labs from the project [A] / [B] Progression. The method is a simple application of some of the core roboswitch patterns we are trying to discover and describe in A Pattern Language for Riboswitch Design.

Table of Contents

- Part 1: Designing switches that can be submitted to the lab
  - MS2 Control ON
  - MS2 Control OFF
  - Sensor A MS2 ON
  - Sensor A MS2 OFF
  - General Strategy
    - Decide on a kernel attraction pattern and draw the schematic for it
    - Choose an appropriate kernel attraction sequence (or sequences)
    - Set the lab's target structure to bind the desired kernel attractions in each state
    - Extend the kernel attractor sequences into attractors that balance the energies between attractions
    - Satisfy miscellaneous attractors puzzle constraints
  - Sensor B MS2 OFF
    - Decide on a kernel attraction pattern and draw the schematic for it
  - Sensor B MS2 ON
  - [A]/[B] with predefined binding sites
  - [A]/[B] with predefined binding sites (alternative)
- Part 2: Improving your design's lab score
  - MS2 Control ON (Tie up loose ends)
    - MS2 Control ON - Lab results
  - MS2 Control OFF
    - MS2 Control OFF - Lab results
  - Sensor A MS2 ON
    - Word change game
    - MS2 gate needed for single input microRNA labs
    - Sensor A MS2 ON - Lab results
  - Sensor A MS2 OFF
    - Late or early dangling bases?

Sensor A MS2 off - Lab results
Sensor B MS2 ON
Sensor B MS2 ON - Results
Sensor B MS2 OFF
Make complementarity to what you wish to catch
The switch accelerator
Sensor B MS2 off - Results
[A]/[B] with predefined binding sites
[A]/[B] with predefined binding sites - Results
[A]/[B] with predefined binding sites (alternative)
R2 (2-states model)
Static stem in the switching area
R2 - Results
R3 (3-states model)

Part 1: Designing switches that can be submitted to the lab

MS2 Control ON

This lab puzzle is trivial to solve. Click on the MS2 stamper icon, then click on any base in the RNA. The MS2 hairpin forms automatically, satisfying the folding constraints.

It what follows, we're going to drawing little schematic diagrams to guide our design process. Here's the diagram for this first design.

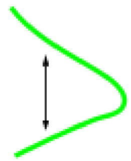

MS2 Hairpin

The green line represents the MS2 hairpin sequence. The double headed arrow indicates that the two ends of the sequence are attracted to each other, forming pairs.

In general, satisfying the folding constraints is where the real work is. But there are additional constraints on the number of bases of any one kind and how many bases are bound into pairs. These are there because without them, there's a good chance that your design wouldn't get synthesized and measured in the lab, simply because it was "unnatural" for RNA. These lab constraints vary over time, as the lab process changes, but currently they are:

- No more than 3 C's in a row
- No more than 3 G's in a row
- No more than 4 A's in a row,
- Fewer than 40% of the bases are A (fewer than 33% is advisable)
- At least 25 paired bases.

So finish your design by mutating bases as necessary to satisfy all these constraints. Once all the borders in the upper left corner have changed from red to green, you can go ahead and submit your puzzle.

(On the other hand, you might want to jump ahead and peek at Part 2. There, we've gathered advice on ways to improve your design's lab score.)

MS2 Control OFF

This is only slightly harder than the previous puzzle. In order to prevent the MS2 hairpin from forming, you'll need to lay down a sequence in the RNA that is complementary to bases in the MS2 hairpin. This sequence doesn't have to match every base in the MS2 hairpin, but it does have to match enough that the total energy of the binding between your sequence and the MS2 bases is lower than the net energy of the MS2 sequence binding into a hairpin.

Here's the schematic diagram for this design

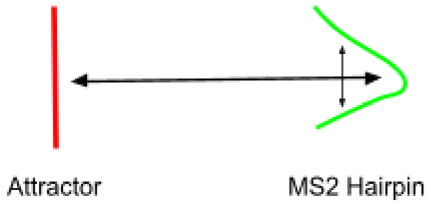

We're going to call single strands in our design that bind to either an input or an output of a switch an *attractor*. In our design, we created an attractor sequence that binds to the MS2 hairpin sequence more strongly than the two halves of the MS2 hairpin bind to each other. In the schematic, we show this by making the attraction arrow between the attractor and the MS2 hairpin thicker than the one between the two hairpin halves. But we keep both attractions in the schematic; we don't want to forget that both attractions are present.

Sensor A MS2 ON

The next lab we'll consider is Sensor A MS2 ON. In the absence of TB-A, the MS2 hairpin should not form. But if the TB-A is also present, the attractor should bind to it instead of the MS2 hairpin sequence, so the MS2 hairpin can form.

Here's the diagram that represents our strategy for solving this puzzle.

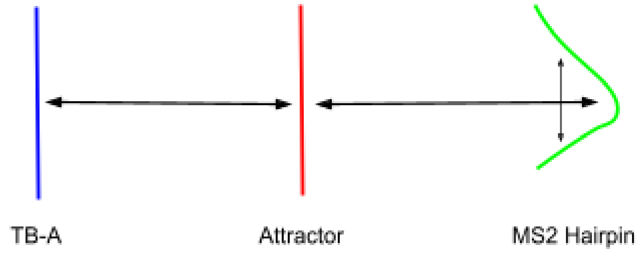

There are now three attractions, the new one being the attraction between the attractor and the TB-A miRNA. To interpret this diagram, consider what happens if the TB-A was removed from the diagram. Without the TB-A there, there can be no attraction between it and the attractor.

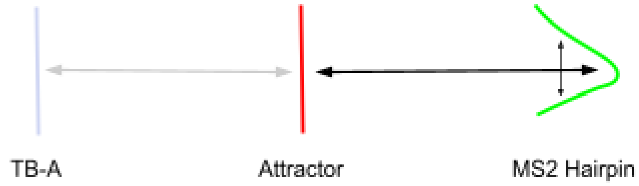

So we would be reduced to the previous diagram, where the attraction between the attractor and the hairpin would be stronger than the other attraction, and the hairpin would not form.

But when TB-A is present the combination of the attraction between TB-A and attractor, plus that between the two halves of the MS2 hairpin, is stronger than the attraction between the attractor and the hairpin. The result is that the attractor binds to TB-A instead of the MS2 sequence and the MS2 hairpin forms.

But how do we determine what attractor sequence is going to work?

It's a two-step process. First we find a short sequence that we'll call a *kernel attractor*. For this attraction pattern, we start by finding a common (or similar) 3-5 base subsequence that occurs in both TBA and the MS2 hairpin. For this design, we'll just show you one of many possibilities. Later, will go into more detail about how to find them.

```
MS2 Hairpin (5'-3'):  ACAUGAGGAUCACCCAUGU
TB-A        (5'-3'):             ACCCCACAAUAAAGAAUAAG
```

Here we have found a sequence, **ACCC**, which is common to both the MS2 hairpin and TB-A. (In this case the two sub-sequences are an exact match. This is convenient, but not necessary.)

Having found a shared subsequence, the kernel attractor is going to be the sequence that will bind most strongly to either instance of the shared subsequence. In this case, the kernel attractor is going to be **GGGU**.

*If your first thought was that it would be **UGGG** instead of **GGGU**, look at the this diagram:*

```
MS2 Hairpin (5'-3')       :  ACAUGAGGAUCACCCAUGU
Kernel Attractor (3'-5'):           ...UGGG...
TB-A(5'-3')               :            ACCCCACAAUAAAGAAUAAG
```

*When single strands bind to form a double stranded stem, they are going in opposite directions (aka anti-parallel.) So **UGGG** is not wrong, but it is the reverse of our convention that we always describe sequences in **5'-3'** order.*

To complete the construction of the attractor, open http://www.eternagame.org/game/puzzle/6296745/ and put it in target mode, with both states showing.

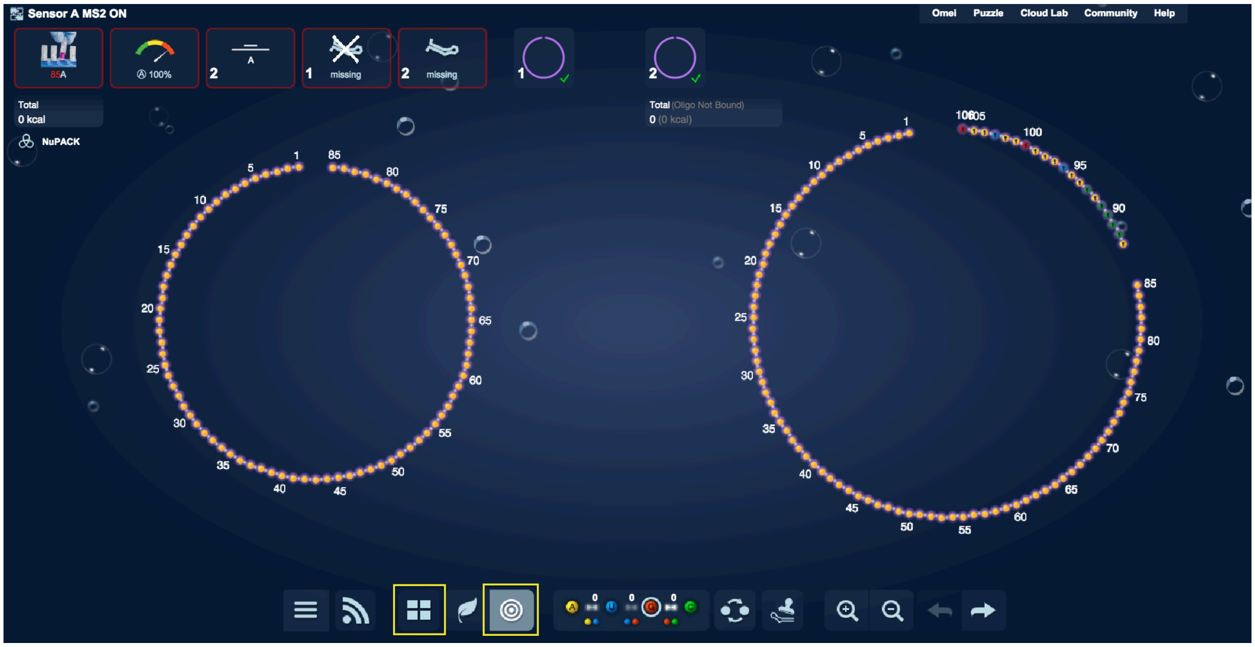

Use the MS2 stamper to place the MS2 sequence somewhere in the middle of the sequence, and use the normal base mutation controls to add in the kernel attractor sequence.

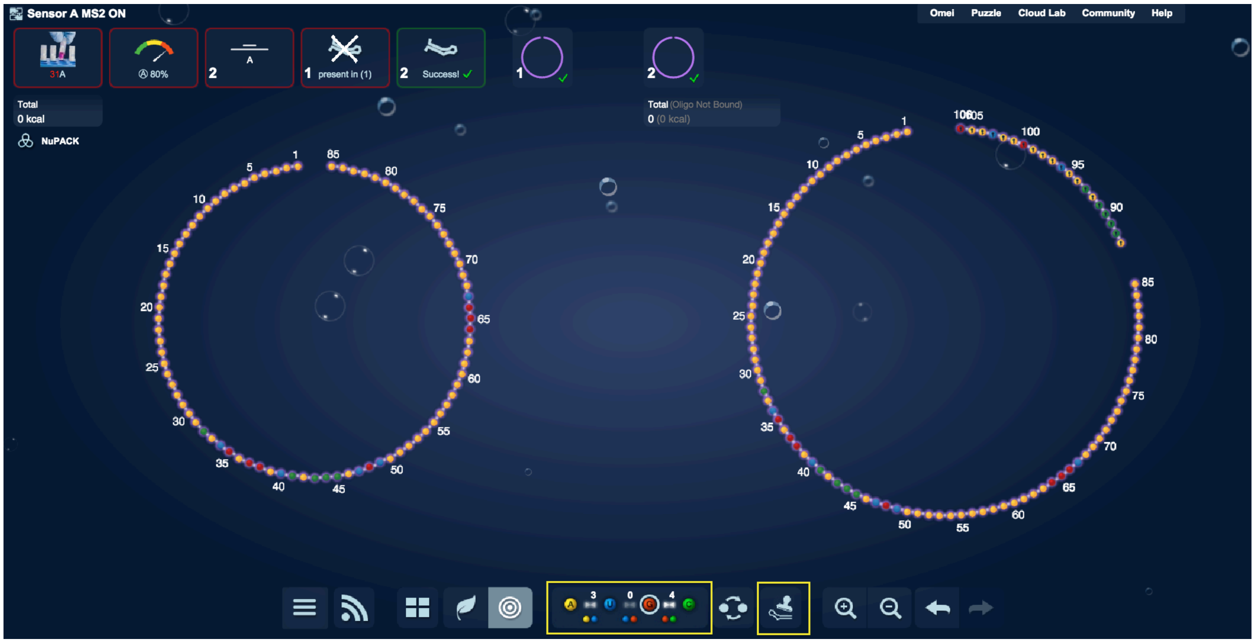

The exact locations for the sequences aren't important for solving this puzzle *in silico*; just be sure to leave some space between the mutated base segments. (In part 2, we'll address the question of how to refine the placement to get not just a valid submission, but a better lab score.)

Now use the "blue glue" tool to set the target folding to pair the kernel attractor with the MS2 hairpin in state 1. In state 2 pair the kernel attractor with TB-A and set the MS2 hairpin structure.

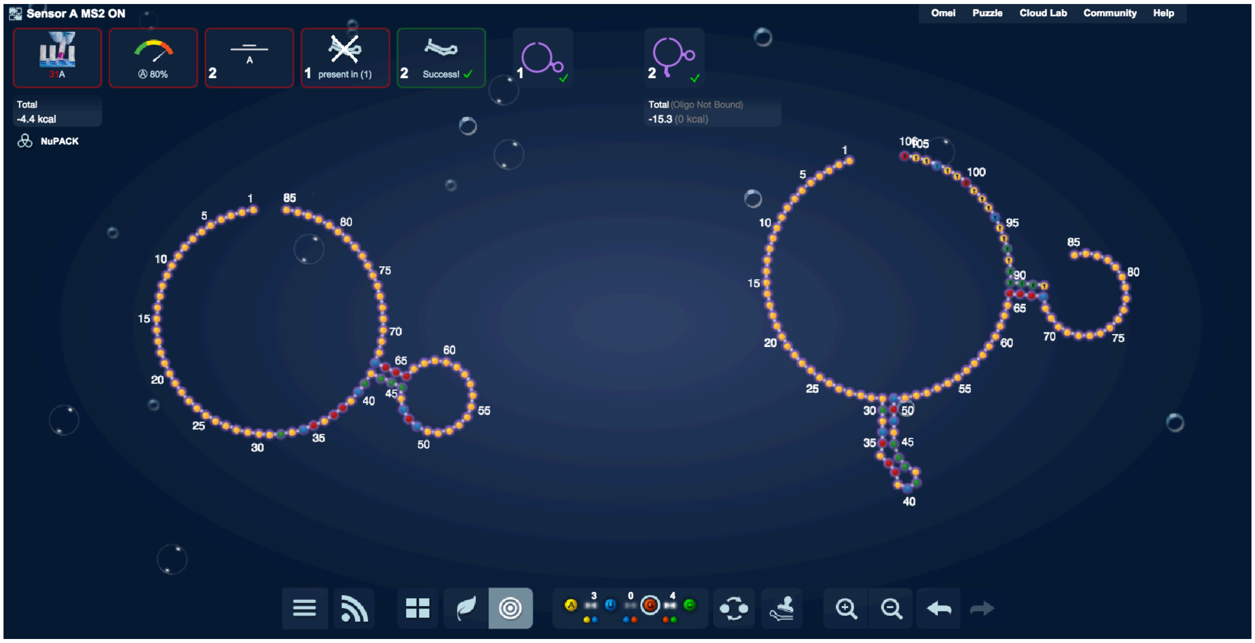

The blue glue tool doesn't have an icon associated with it. If you aren't familiar with its use, search for "blue glue" in [Switch Lab Intro](). It's currently slides 13-15, but that might change as the document is refined.

Finally, we need to extend the kernel attractor segments in each direction to balance the relative strength of the competing attractions, thus satisfying the folding constraints. Our goal for is all the binding and folding constraint boxes in the upper left corner to have a green border. As our design is now, the only satisfied constraint is that the MS2 hairpin forms in state 2. Here's the view in natural mode:

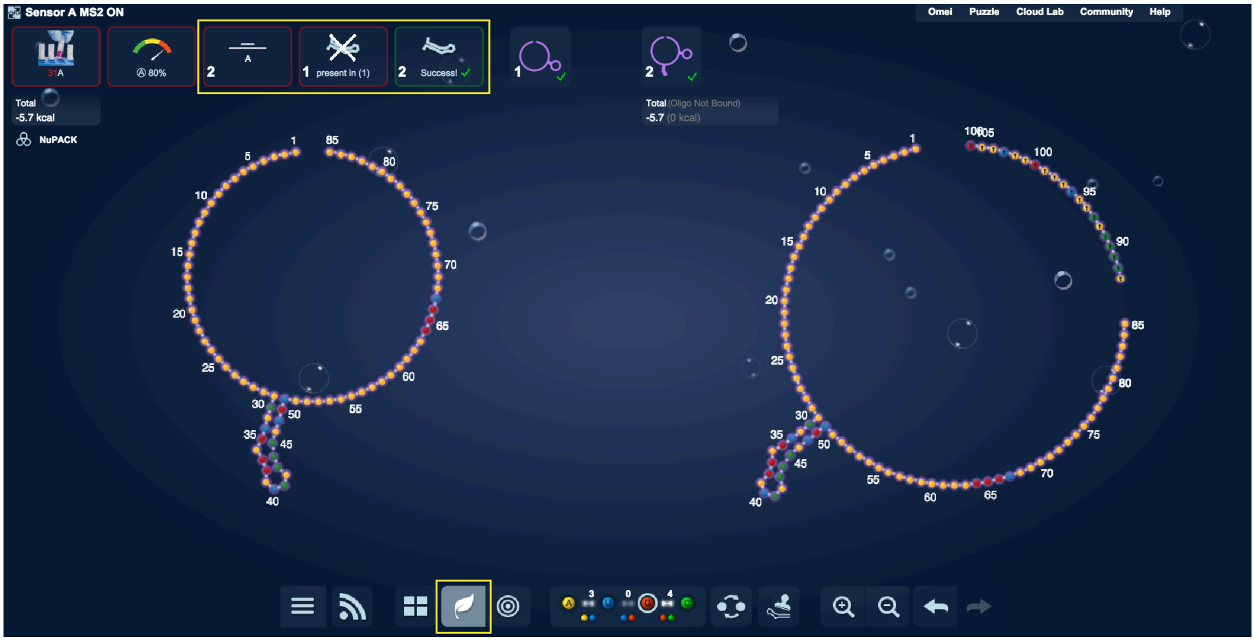

We can see from the leftmost of the highlighted constraint boxes that in state 2, our RNA should bind with TB-A. The red border surrounding that constraint indicates that it is not doing so now, so we need to strengthen that attraction. To do this, return to target mode and look at state 2. Mutate bases one at a time, starting from 65 and working lower. Mutate them so that they will form a strong attraction to TB-A. (The appropriate mutations are generally easiest to read off in target mode.)   Keep going until the border on the constraint box for state 2 turns green. This will mean that the binding between the attractor we are creating and TB-B is strong enough to cause them to pair.

Due to the simplicity of this puzzle, as soon as we satisfy this one constraint, all three constraint boxes turn green:

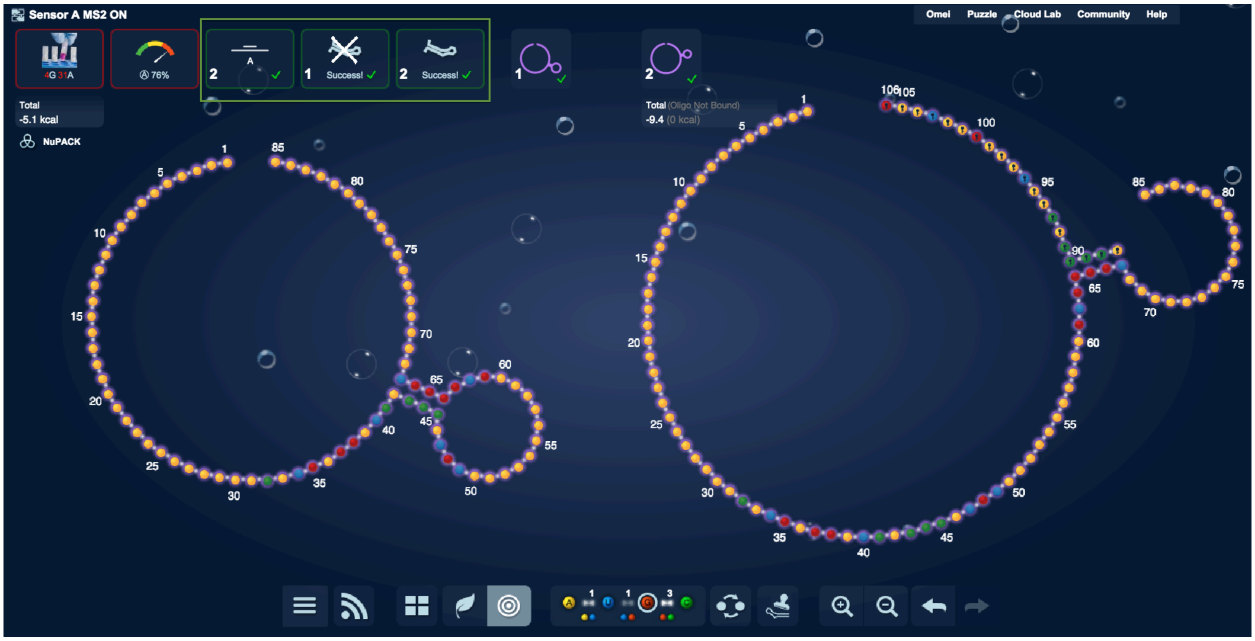

Drawn as one line

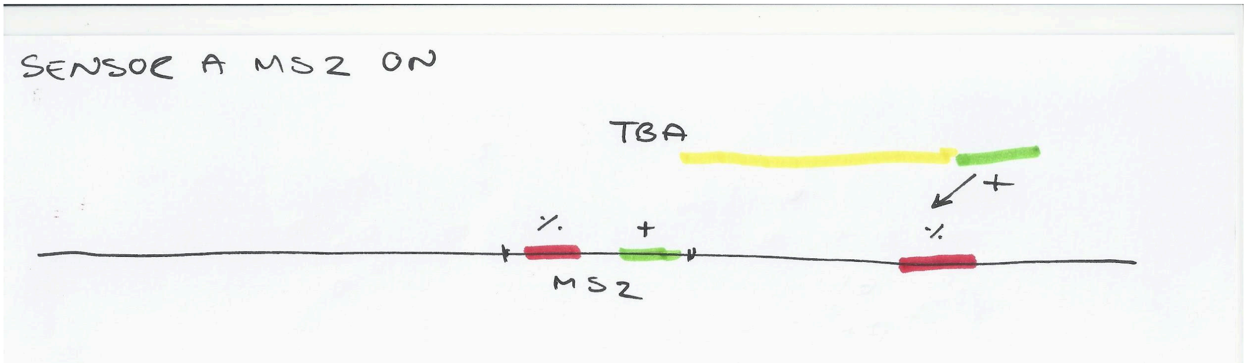

Drawn with two states as I would normally draw it. (Shown in native mode, where the puzzle image above is showing target). In first state MS2 gets turned off, by the G part of the TB A complement pairing up with the MS2 C's and thus shutting the MS2 down. In second state TB A pairs up with the TB A complement and which leaves the MS2 free to pair up with itself. I haven't drawn a blue line in to show that the RNA sequence also pairs with some of the mainly yellow part of the TB A, as I usually would. To keep it very simple here.

The trick with this style is that I simplify what the microRNA is, to its main strong bases (here C's) and to its overall A bases (yellow). Basically I brew its nature down to its main characteristics, to make it simpler to see what it can and will do.

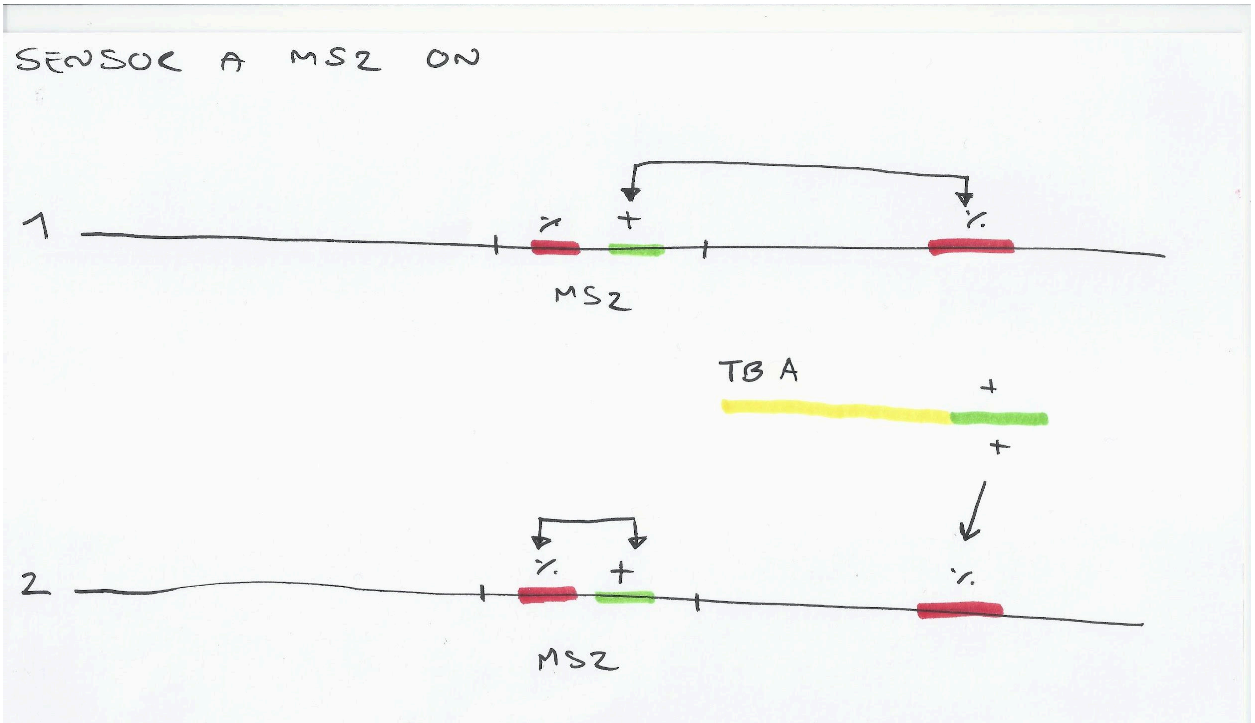

We'll see in later, more complex puzzles, that energy balancing will not be so simple.

Another way to look at what we have done is to refer back to where we chose the kernel attractor, and fill in the rest of the bases in the attractor:

```
MS2 Hairpin (5'-3')         :   ACAUGAGGAUCACCCAUGU
Completed attractor (3'-5')   :      ...UGGGGUG...
TB-A(5'-3')                 :              ACCCCACAAUAAAGAAUAAG
```

The first four bases of the attractor (UGGG in 3'-5' order) can form a strong stem with four bases in the MS2 hairpin sequence, thus preventing the hairpin from forming. Or, if TB-B is present, the last 7 bases (UGGGGUG in 3'-5' order) can form a strong stem with the TB-B, freeing the MS2 sequence to form the hairpin. But both stems can't form at the same time, so we have two distinct states.

Switching to natural mode, we can see how the NuPACK energy model predicts our switch will fold in each state:

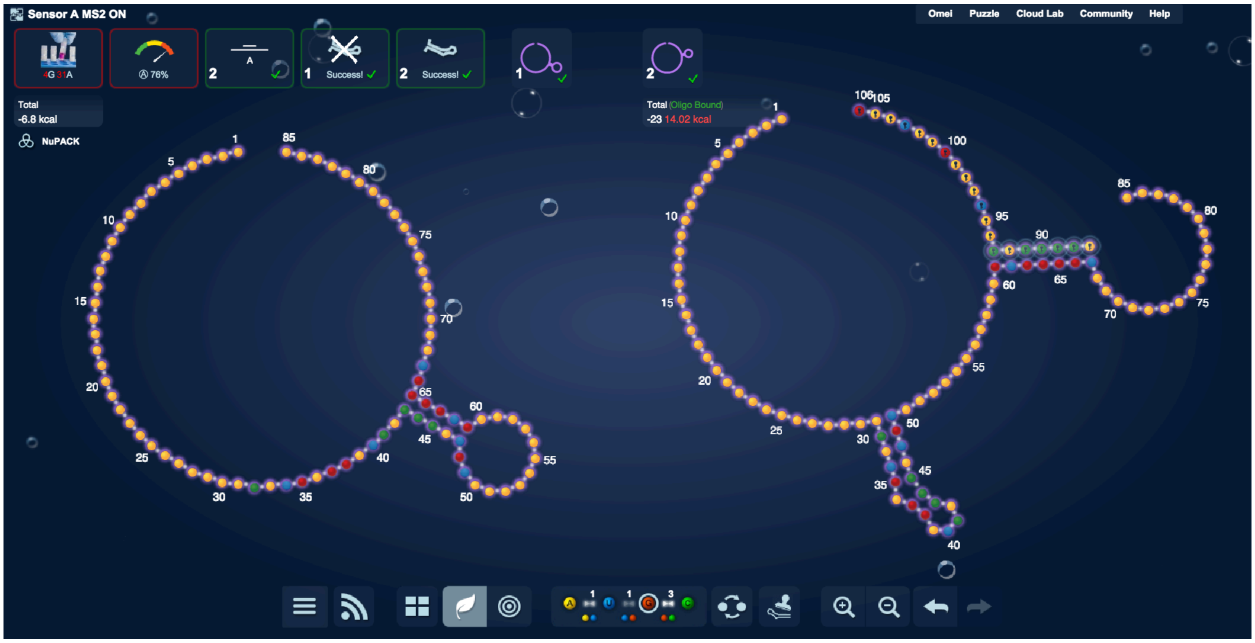

We've now completed the essence of the puzzle. In order to submit the design for synthesis, you can simply mutate the other bases as necessary to satisfy the other two constraint boxes in the upper left corner. (Or you can consult Part 2 for some hints on improving the lab score of your design before submitting it.)

Sensor A MS2 OFF

This lab is the inverse of Sensor A MS2 OFF. That is, it requires that in the absence of TB-A, the MS2 hairpin forms, and the presence of TB-A prevents its formation.

We can adapt the Sensor A MS2 OFF attractor pattern to this design by simply removing the design lane, like this:

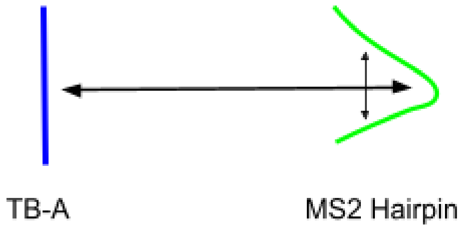

This can be verified by considering the two cases: either TB-A is present or it isn't. If TB-A is present, as above, as long as the attraction between TB-A and the hairpin is stronger than the attraction between the two stems of the hairpin, the hairpin won't form and the fluorescence

signal will be absent. If TB-A is not present, the hairpin will form and the fluorescence will be seen.

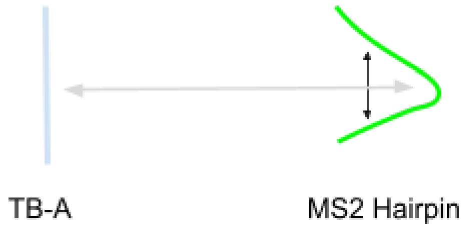

To
...

General Strategy

We're not going to go through such excruciating detail for the remainder of the labs. So in this section, we'll summarize the general strategy for creating designs using our chosen set of patterns. Then, for the rest of the designs, we'll just elaborate on certain aspects that are new in that particular puzzle.

Decide on a kernel attraction pattern and draw the schematic for it

The touchstone for creating a design in this way is to decide on a promising kernel attraction pattern that is appropriate for the puzzle and go through all the steps keeping that attraction in mind.

Here are some more examples of attraction patterns we'll be using in subsequent labs.

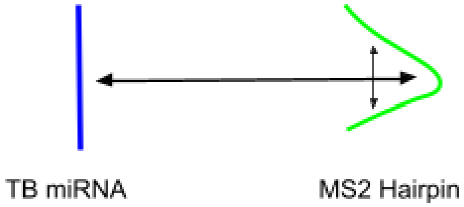

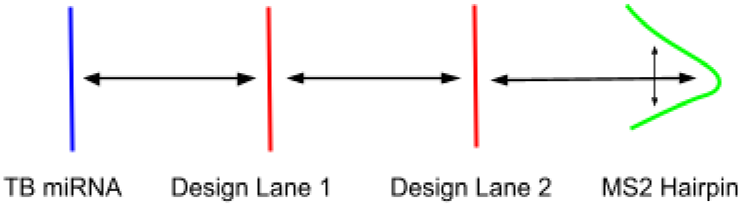

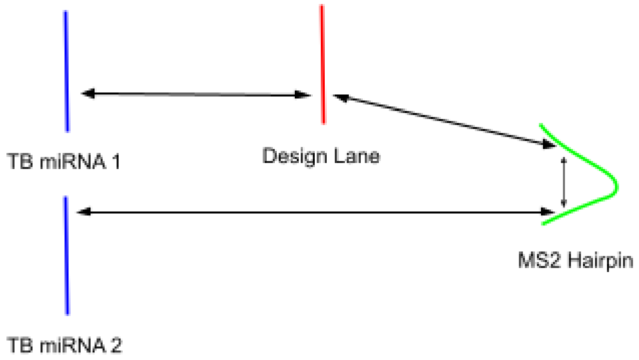

We'll wait to discuss them in more detail as we use them.

Choose an appropriate kernel attraction sequence (or sequences)

Finding good kernel attraction sequences is probably the most tedious part of the process. Fortunately, for any given combination of input/output RNAs (either separate strands of miRNA or an RNA aptamer sequence), the set of plausible kernel attraction sequences is fairly small. Furthermore, they can be determined once (either manually or with a script) and shared among all players.

In the previous section, we reached into the hat and pulled out a 4-base sequence that is shared between the MS2 hairpin and TB-A:

```
MS2 Hairpin (5'-3')      :  ACAUGAGGAUCACCCAUGU
Kernel Attractor (3'-5'):              UGGG
TB-A        (5'-3')      :              ACCCCACAAUAAAGAAUAAG
```

And from that, we derived the attraction pattern GGGU. But this is by no means the only possibility. To find others (by hand) we can systematically align the two sequences against each other in all ways, picking out common sequences by eye.

Here's another example:

```
    MS2 Hairpin (5'-3'):
ACAUGAGGAUCACCCAUGU
    Kernel Attractor (3'-5'):                      UAUUC
    TB-A        (5'-3'):          ACCCCACAAUAAAGAAUAAG
```

Notice that in this case, the third base of the 5 that are highlighted (G in the MS2 hairpin and A in the TB-A) are not identical. But because they are both purines, they both will pair with U, and the sequence CUUAU makes a perfect kernel attractor.

And another:

```
MS2 Hairpin (5'-3'):      ACAUGAGGAUCACC CAUGU
Kernel Attractor (3'-5'):          GGUGGUG
TB-A         (5'-3'):              ACC CCACAAUAAAGAAUAAG
```

Here we have come up with a long (7 bases), strong (lots of GC pairs) kernel attractor GUGGUGG by introducing a 1-bulge in each of the pairings between kernel attractor and its partners.

In general, any two RNA sequences of about these lengths or larger are almost guaranteed to have multiple possible choices for a kernel attractor sequence.

Set the lab's target structure to bind the desired kernel attractions in each state

This is the same process for every puzzle. If you find you are having trouble, just refer to the detailed instructions in the Sensor A MS2 ON discussion.

Extend the kernel attractor sequences into attractors that balance the energies between attractions

This is basically the same process for any puzzle, but the balancing can be a little more subtle than that for the Sensor A MS2 puzzle. We'll elaborate more on that in the puzzles that follow.

Satisfy miscellaneous attractors puzzle constraints

This is the same process for every puzzle. But you should know that not all ways to satisfy these constraints will work equally well in the lab. So for more discussion on this topic, see Part 2.

Sensor B MS2 OFF

Returning to the consideration of specific puzzles, we'll now consider Sensor B MS2 OFF.

Decide on a kernel attraction pattern and draw the schematic for it

Sensor B MS2 ON

[A]/[B] with predefined binding sites

[A]/[B] with predefined binding sites (alternative)

Part 2: Improving your design’s lab score

I have put up an analysis of what really works for the Reporter lab which despite not using MS2, is related this one. This may help you get a feel for what a switch lab with several inputs wants.

Now we have lab results back with a nice portion of winners in all of the A/B labs. The absolute best way to improve your lab puzzles, are to watch the lab results.

You can follow and add to the analysis here in this forum post:

[A]/[B] discussion

MS2 Control ON (Tie up loose ends)

To make good riboswitches, we don’t want to leave loose dangling ends - loose single stretches of bases that is not participating in anything. That is except if the dangle is taking part in the switching. So before submitting your design, tie up the ends into a stable structure. This could be one long stem, perhaps with an interior loop or two, or something more complex. In what follows, we’ll call this step *Tie up loose ends*.

In this lab we need to put in a MS2 hairpin. I placed the MS2 hairpin in the middle. Mostly it doesn’t like being placed too far at either end. While it is possible sometimes, it is generally a bad idea - at least not if you plan having it involved in unfolding and get turned off. The more inputs you have, the more willing it will be though. However middle position generally works well, so that's what I will go with when given a choice

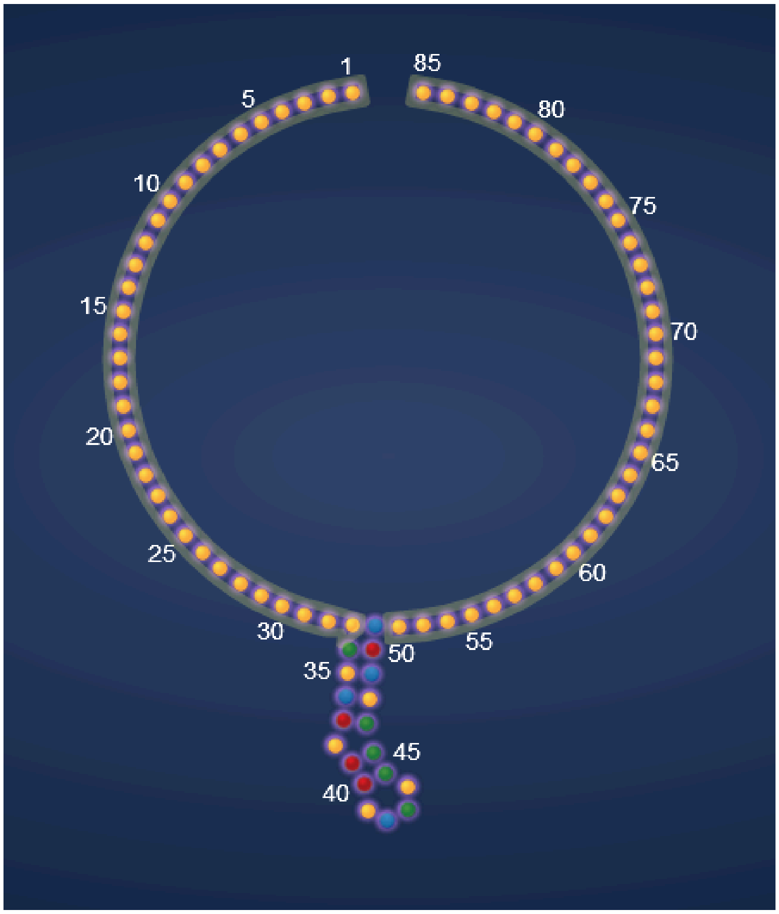

Instead of leaving heap of yellow A bases at the sides, I made two stems instead. However making stems too long without making any breaks in them, can also cause trouble.

Illustration of tying up loose ends

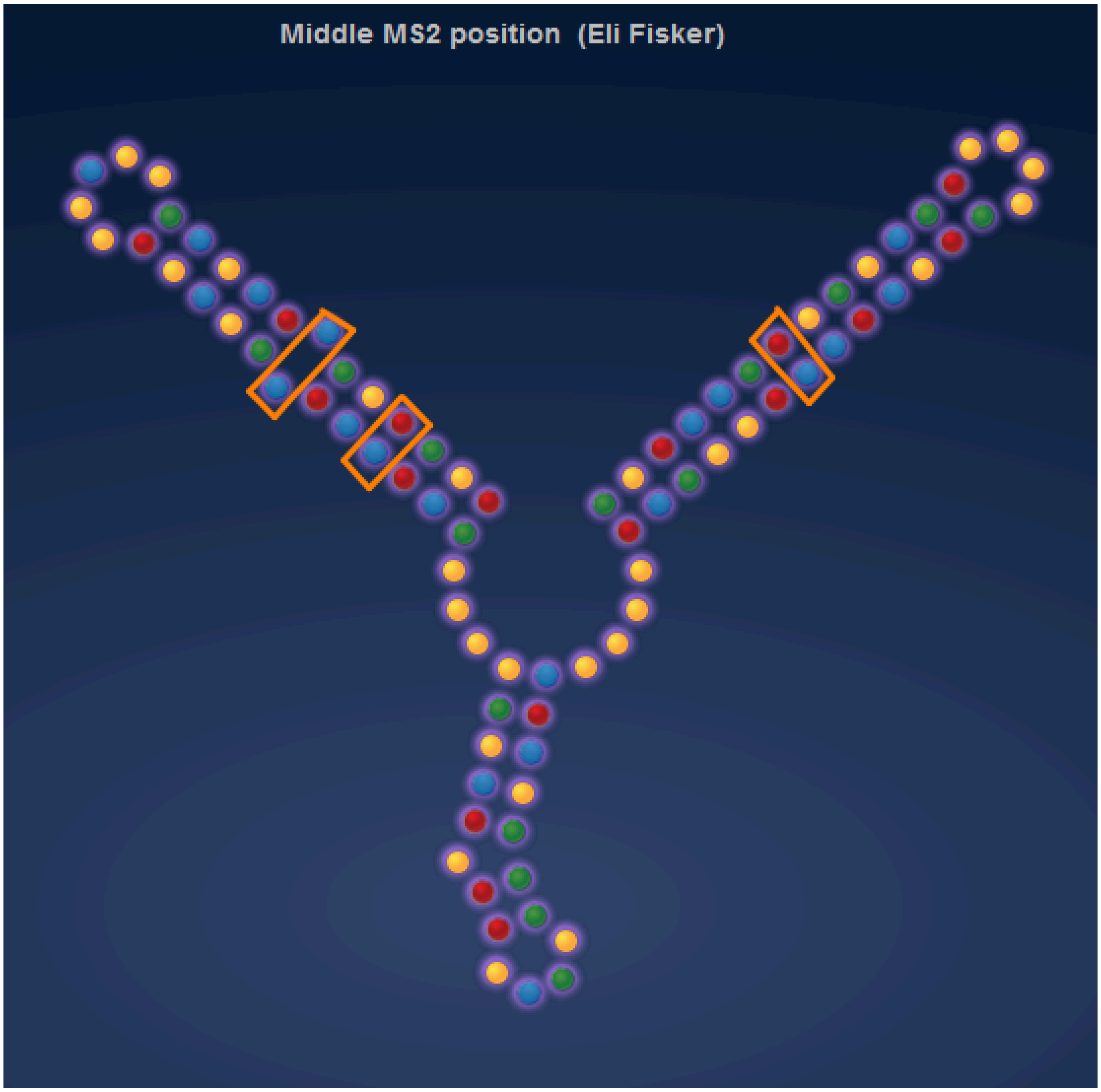

Notice the 1-1 loops and the GU's in the long static stems. Usually when a stem gets 7 base pairs and longer, it starts benefitting from getting either a 1-1 loop or a GU. Or both if really long.

This is a pattern to be used in all the labs.

MS2 Control ON - Lab results

In this lab we had many winners. It is generally not too hard to turn on MS2 especially not when it is alone. Being on and working is its favorite position. It's the turning it off that is harder work. Turnoff labs are harder in general.

The design I posted above did end up working.

Score 100%

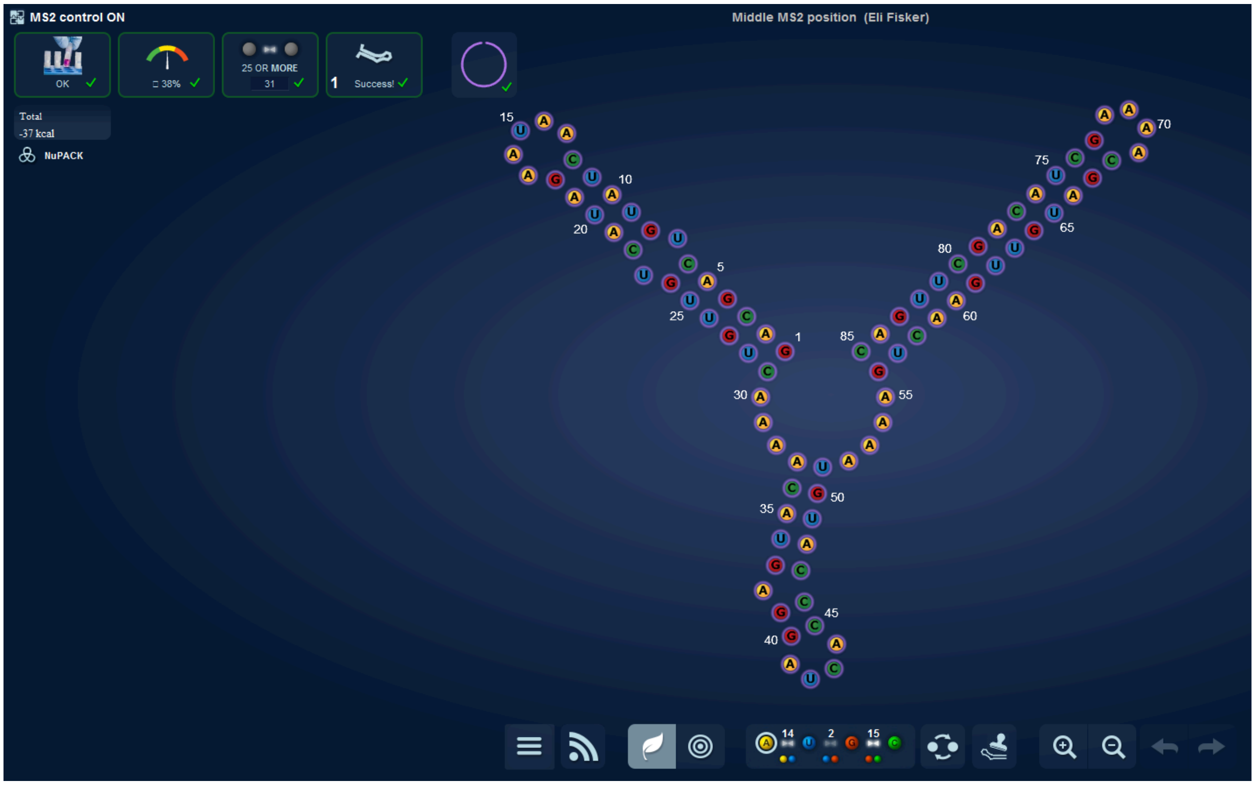

http://www.eternagame.org/game/browse/6296743/?filter1_arg1=6301192&filter1=Id&filter1_arg2=6301192

There are many other ways of achieving an MS2 on.

- Place a GC pair before the MS2 and it be less likely to move - this has been used in designs where the switching of the MS2 needed to be baked.
- Placing a stem in front of the MS2 (MS2 gate). Often it comes with a small internal loop or bulge.

The above also slows switching down and can in designs where the MS2 needs to be off also, be counter productive.

MS2 Control OFF

To turn off the MS2 sequence, you can use either side next to the MS2, to turn it off. It should be a sequence that is complementary to the MS2. It can be one or two sequences that are split up.

Illustration of where to place turnoff sequence.

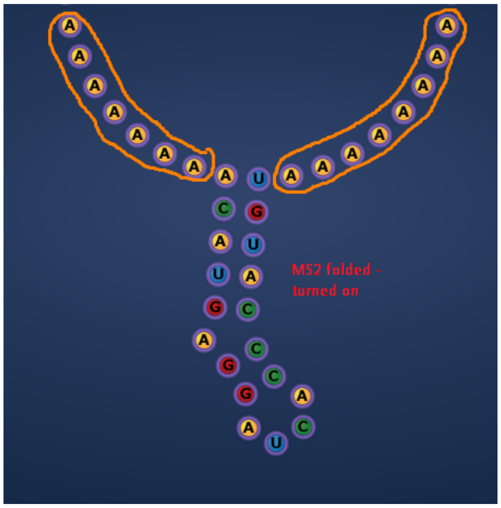

Any sequence that is complementary to the MS2 or partly complementary will do the trick *in silico* - puzzle simulation - if it is long enough. Labs are a bit more specific in their wants.

Here is a simple way to do it.

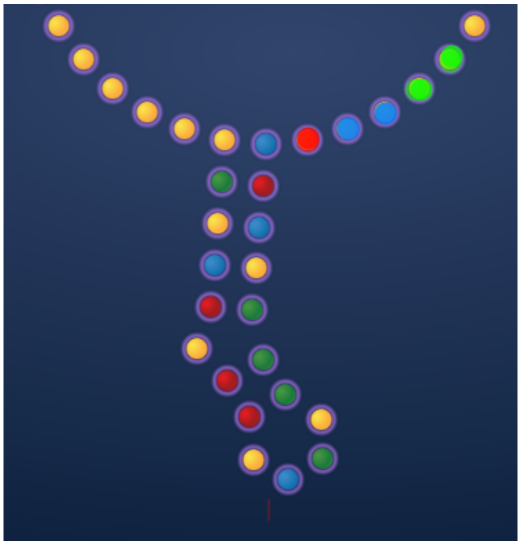

Placing these bases at the side of the MS2 will make the puzzle slide into this, when in native mode:

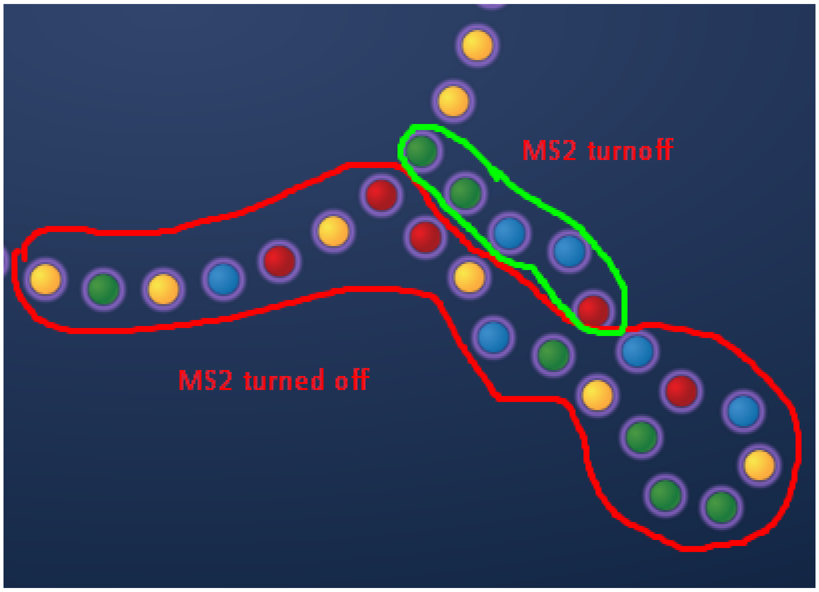

Often for many MS2 labs a stretch of pyrimidines (C’s and U’s) is favored, because a sequence like this can be complementary to the twin G’s in both MS2 and FMN. Till now it has often been one of the most effective way for a turnoff. But this is a microRNA lab and it doesn’t have FMN, are leaving a wider playground for a turnoff. So you can use different stretches of the MS2 for turnoff.

Notice the mismatch (two bases that do not bind) in the 1-1 loop. This regularly turns up in good lab solves in relation with MS2 turnoff. It can also be a small bulge forming. We believe it is to help so it is easier for the MS2 to get unstuck and turned on again. If things gets too stable, they can get harder to get moving.

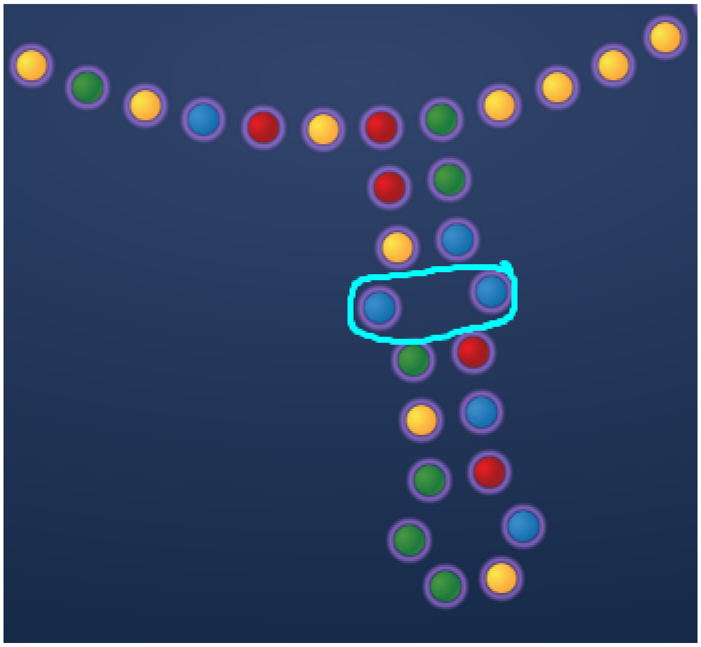

For RNA's with more than one inputs, like the [A]/[B] labs, we need to use both sides on the MS2 for turnoff sequences. (For the R3 puzzle)

One can also do [long range turnoffs]() of the MS2. For now they are generally less effective in labs with one microRNA input, but I think they may grow more useful, the more microRNA inputs we get per lab.

What to use for turnoff sequence will all depend on which microRNA's are there, in which order the microRNA's need to be turned off and how many there are. So take home lesson is that each individual lab will need something slightly different, but you can often reuse turnoffs that works well, in a similar lab.

Here is an example from [a microRNA lab](). Notice that almost all of the winners uses just a few variations of very similar MS2 turnoffs. The paler green column (left) shows the section that the turnoff targets, inside the MS2 and a bit besides.

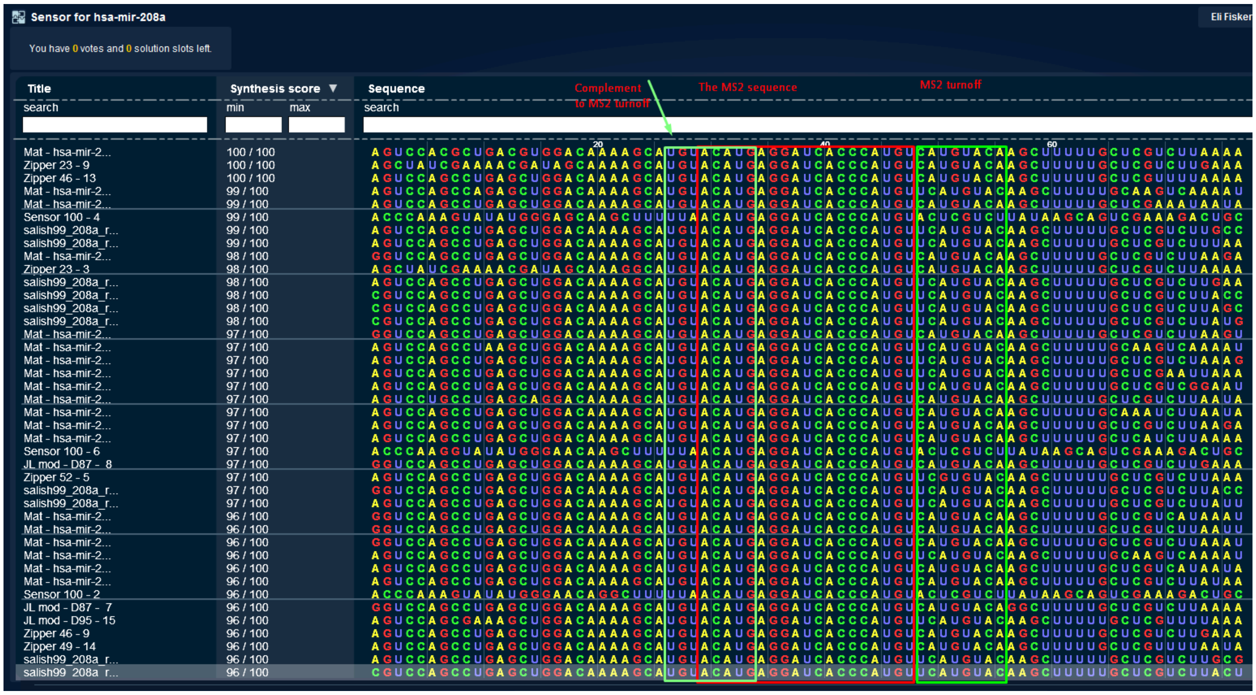

You can find [past microRNA labs]() in the archive. We only have results on the series [miRNA Switch Lab]() yet. Note last Round 3, didn't have good data. So pick [Round 2]() to see results.

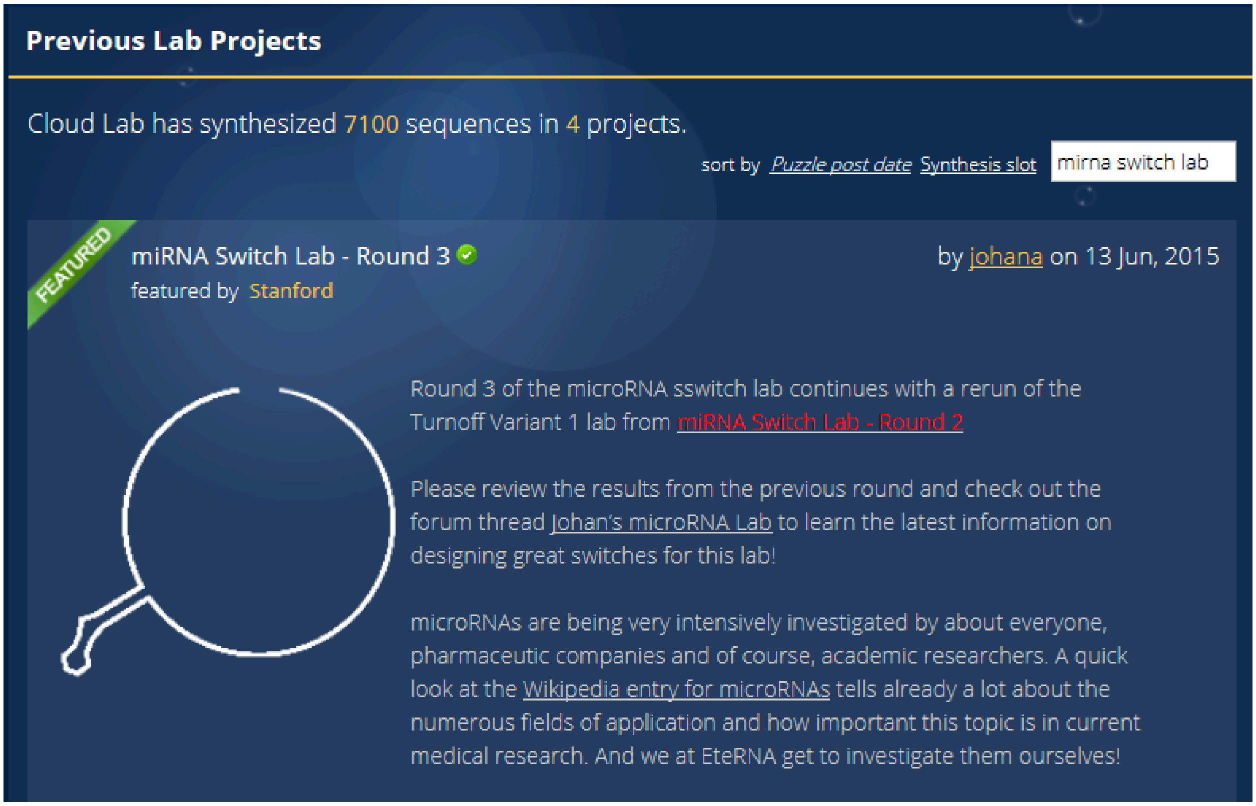

MS2 Control OFF - Lab results

In this lab there were also many winners but fewer than in the MS2 control ON lab.

The one I posted for lab was not strong enough to work on its own, but some mods of it with variations added in did. I'm so used to working with MS2 hairpins together with aptamer and MS2's that also needs to get turned on again. The aptamer adds an extra energy bonus making the a fold favorable. But this lab holds no aptamer and this MS2 turnoff was too weak on its own.

Generally a longer complementary stretch or two is a more secure way of turning off in this lab. While MS2's are not too happy to be turned off, you can generally always get them turned off, if you just have a long enough stretch of it targeted.

Score 0%

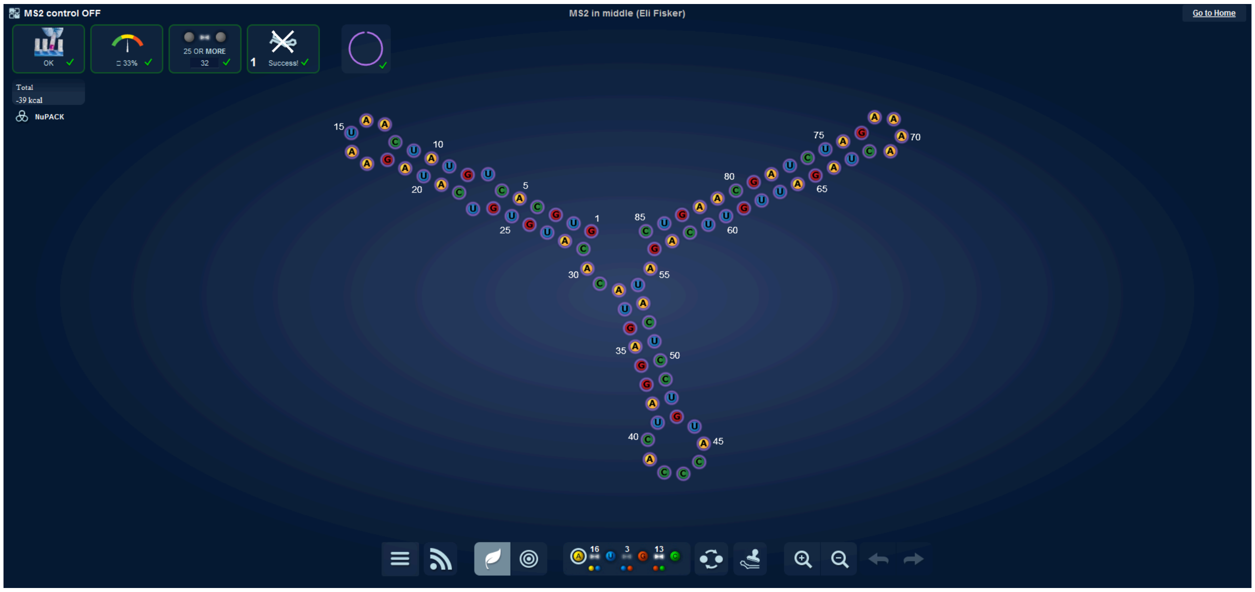

http://www.eternagame.org/game/browse/6296744/?filter1=Id&filter1_arg2=6301260&filter1_arg1=6301260

Here is one that did work.

Salish, score 100%

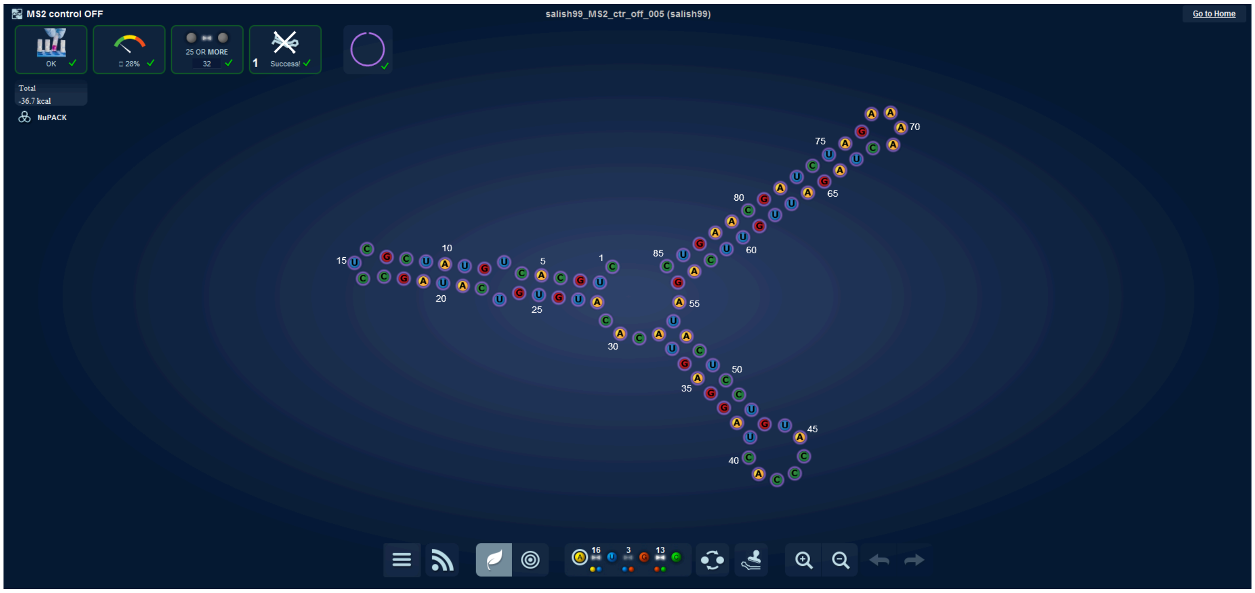

http://www.eternagame.org/game/browse/6296744/?filter1=Id&filter1_arg2=6313825&filter1_arg1=6313825

There were many alternative ways of achieving a MS2 turnoff. Here is one of the more fun ones.

Double strand turnoff - a strategy that was also used in the Riboswitch on a chip Exclusion labs.

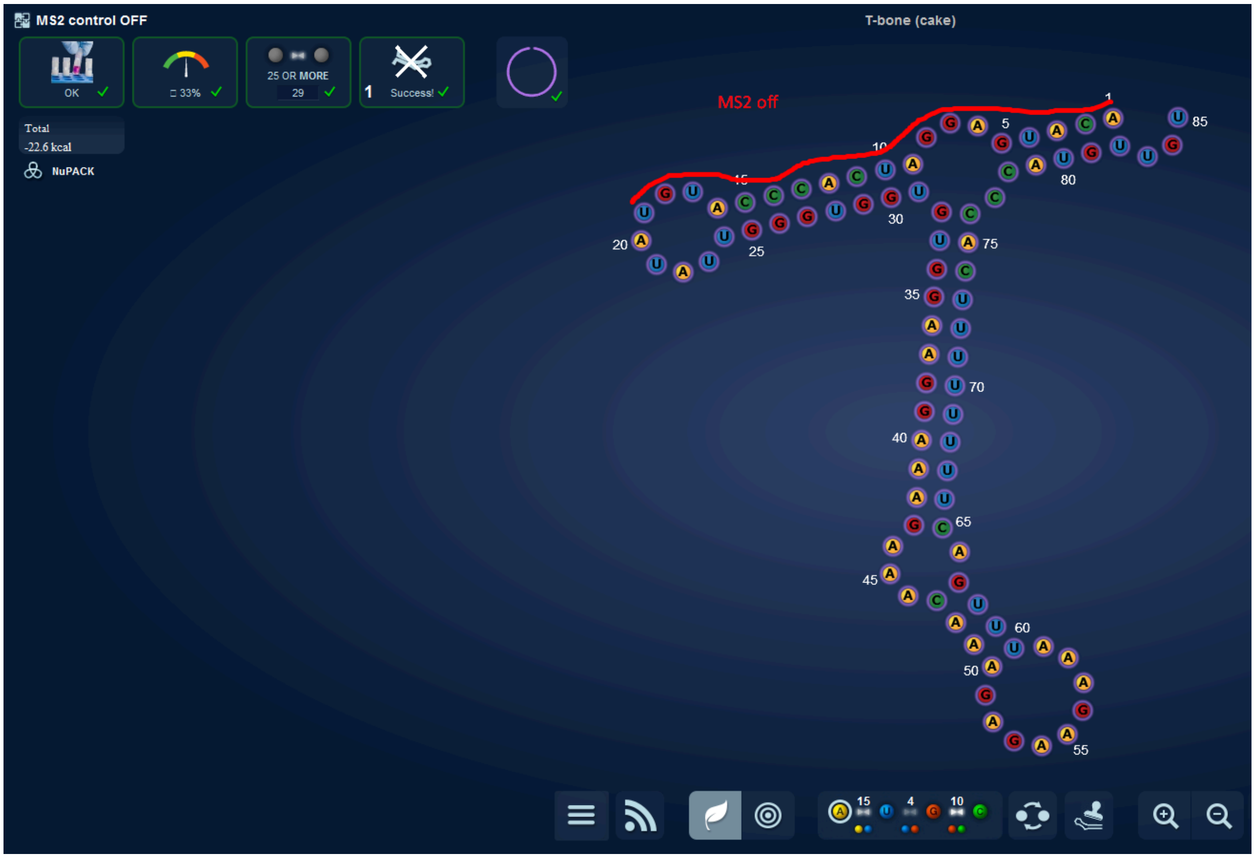

http://www.eternagame.org/game/browse/6296744/?filter1=Id&filter1_arg2=6349929&filter1_arg1=6349929

Usually MS2 don't like to get to the furthest end of the RNA design. Only exceptions so far tends to turn up in some two input labs. MS2 generally needs to be held from both sides for a turnoff as they are very strong. But when they are in company with two equally as long RNA inputs, they protest less about an end position.

Sensor A MS2 ON

Now to make the puzzle style solve into a good lab solve also, it helps moving the microRNA complement to TB A (Base 59-67) further to the end of the sequence.

The MS2 turnon lab with single input microRNA labs have shown a strong preference for having the microRNA complement at the end of the RNA.

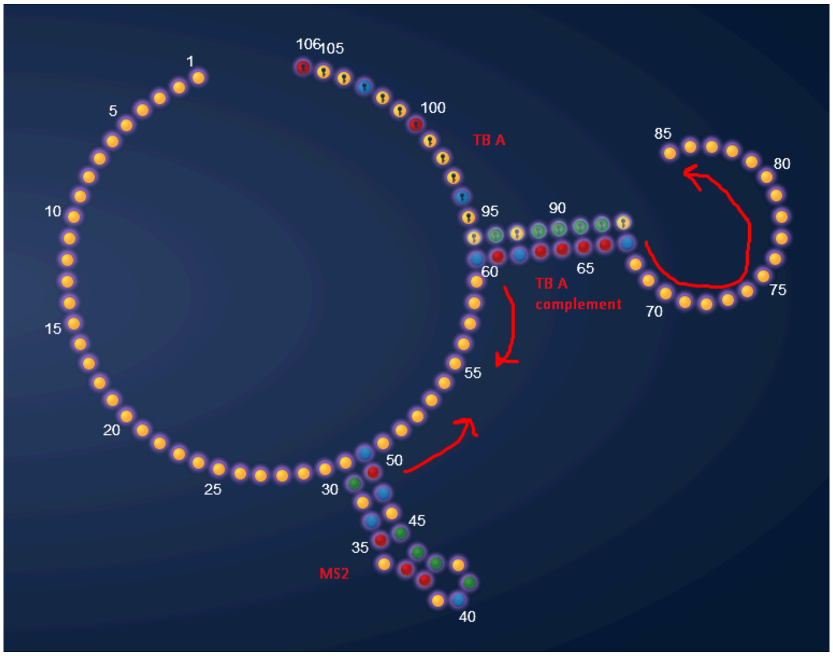

Basically a MS2 turnoff sequence is most effective if it is right next to the MS2 sequence or just short away. The further apart the turnoff sequence gets from the MS2, the less likely it is that the microRNA complement is actually going to turn off the MS2.

But first I need to ensure that the microRNA complement is fairly stable before I start sliding the sequence. Then I'm a little less likely to encounter problems with the puzzle going unstable from the move. (The puzzle will often go unstable during the move, but don't let it stop you from trying to move it. First when you have moved everything you will know if moving it has been successful.) So I add some few more bases so the design can tolerate me removing the one too many G's in line, so the puzzle constraints are fulfilled.

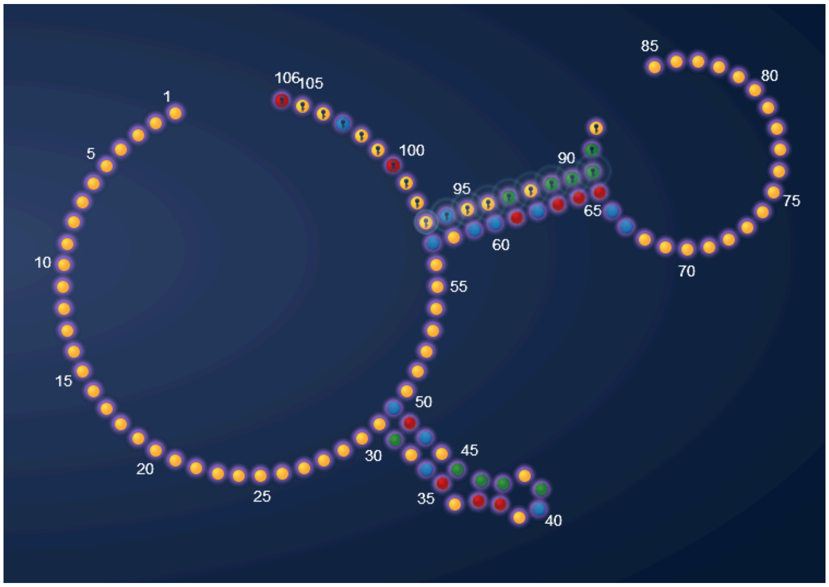

Then I moved the TB A complement in one go, by simply painting the bases at later in the sequence and deleting the ones before. I used the MS2 stamper tool again to move the MS2 closer to the TB A compliment and I moved it a bit closer than it was before.

NB: There are now a very easy way to slide whole stretches of bases back and forth, instead of moving one base at a time. For more on how to do this, check the introduction to the [Sequence Shifter tool](#).

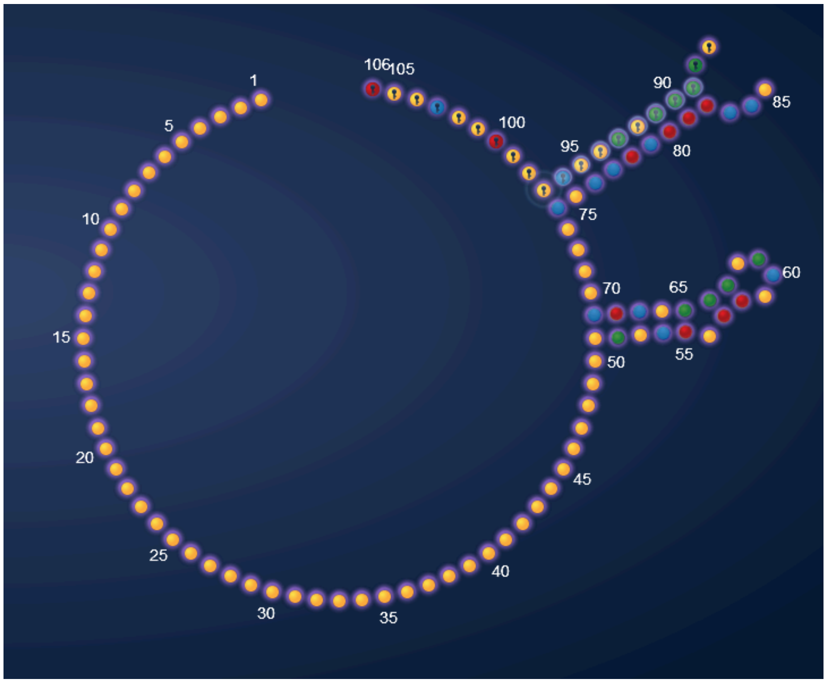

Notice that the TB A complement is moved almost but not fully to the end of the RNA sequence. The results we have seen to now indicates that leaving a few bases unbound at ends, when a microRNA binds up with a microRNA complement, may be beneficial. Here is 3 bases. 1 or 2 should generally do. Does this happen in certain labs more than others? You yourself can watch out for new trends emerging.

Now the loose ends needs to get tied up for all the loose beginning bases. Place the static stem some 3-4 bases away from the MS2. This will help the elements stay separate.

Here is a slightly varied full solve.

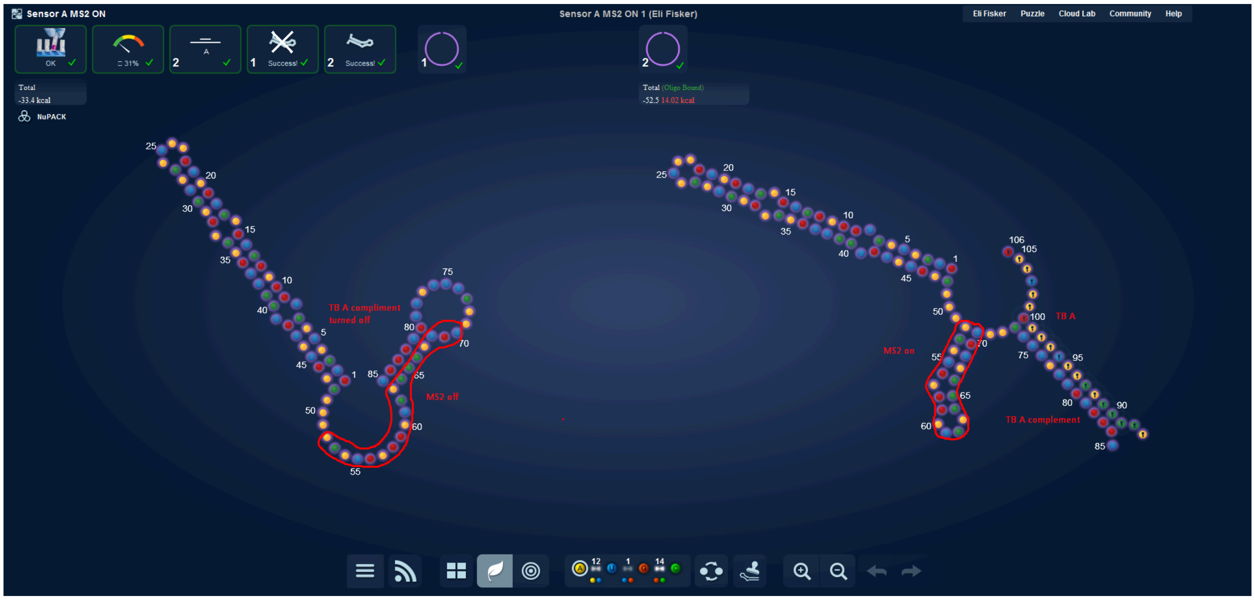

Now I'm not fully happy with the dangle I get from the MS2 in first state (left) and especially that there is no dangle for the TB A complement in second state (right) - unless the one in the end loop counts. End dangles are generally a great tool for [catching and hooking] your design up with the RNA input. Something that [jandersonlee demonstrated] with his winning design in the first lab we had with single input microRNA.

Generally it seems harder for the microRNA to bind up bases in end loops, internal loops and multiloop rings, compared to gap bases and best of all tail bases. Preferable the microRNA wishes to land in the late end of the RNA sequence, but if not available or possible, early tail bases will do too.

I decided to look at the microRNA winners from a similar lab, that we got data from and solve in a similar manner.

While I would far have preferred seeing a pyrimidine (C and U's) dangling stretch, to a purine (G and A's) dangling stretch, as this is what have worked till now, I have also seen that it is most beneficial placing the microRNA complement late in the RNA sequence. So I think a G's dangle is worth trying out. At least this is what I will be betting a part of my lab slots on.

Word change game

I have ended up realizing that this strategy is the most central, when it comes to microRNA labs with single inputs. I have written a short intro to [why].

One way to get to a solve is to have 5 strands that are complementary with each other. Of these 5, two are already predetermined, as the MS2 sequence and the MicroRNA sequence are

locked. So what we want is 3 strands that makes complementarity between the locked stretches. Illustration of the switch mechanism from the microRNA lab we have had most winners in. (MIR = can be any microRNA)

Notice that both states have a 1-1 loop in the switching area. We believe this helps with the breaking open of the switching area so it doesn't get too stuck and unwilling to switch.

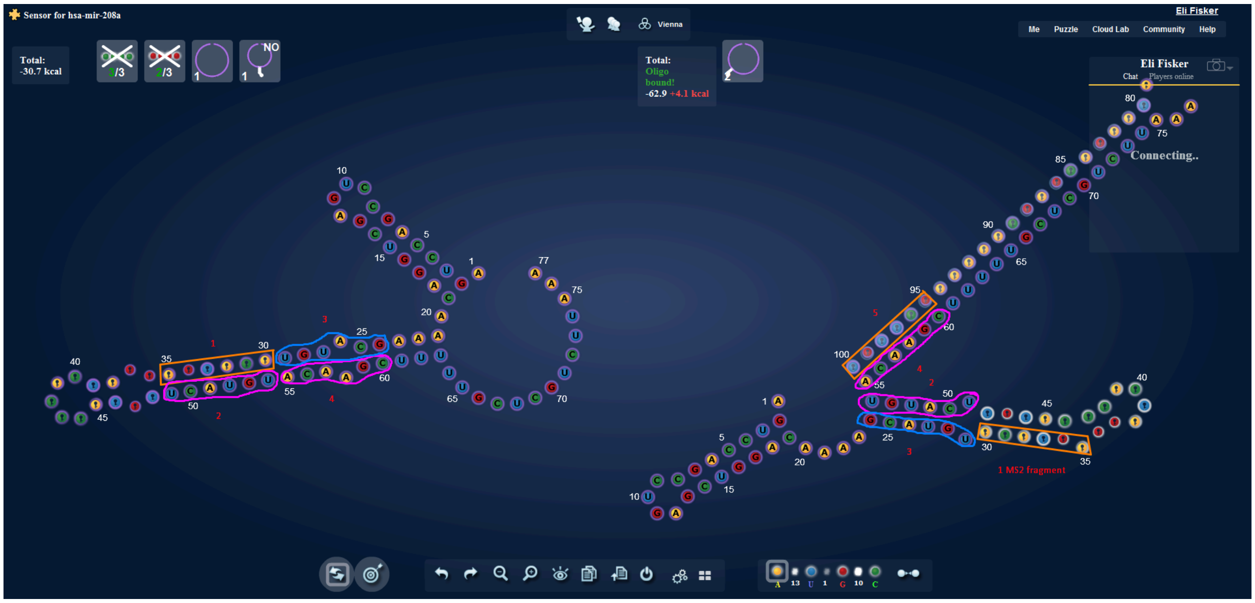

Design example with complementary word gaming. Both the MS2 and the microRNA input is locked in sequence. So the trick is to make the sequences that goes in between these, be complimentary enough with each other to make the individual parts fit, so they can take turns turning each other on and off. They don't need to be 100% complementary - just the main part of them have to be. Usually a 1-1 loop or sometimes a 1 nucleotide size bulge will be beneficial.

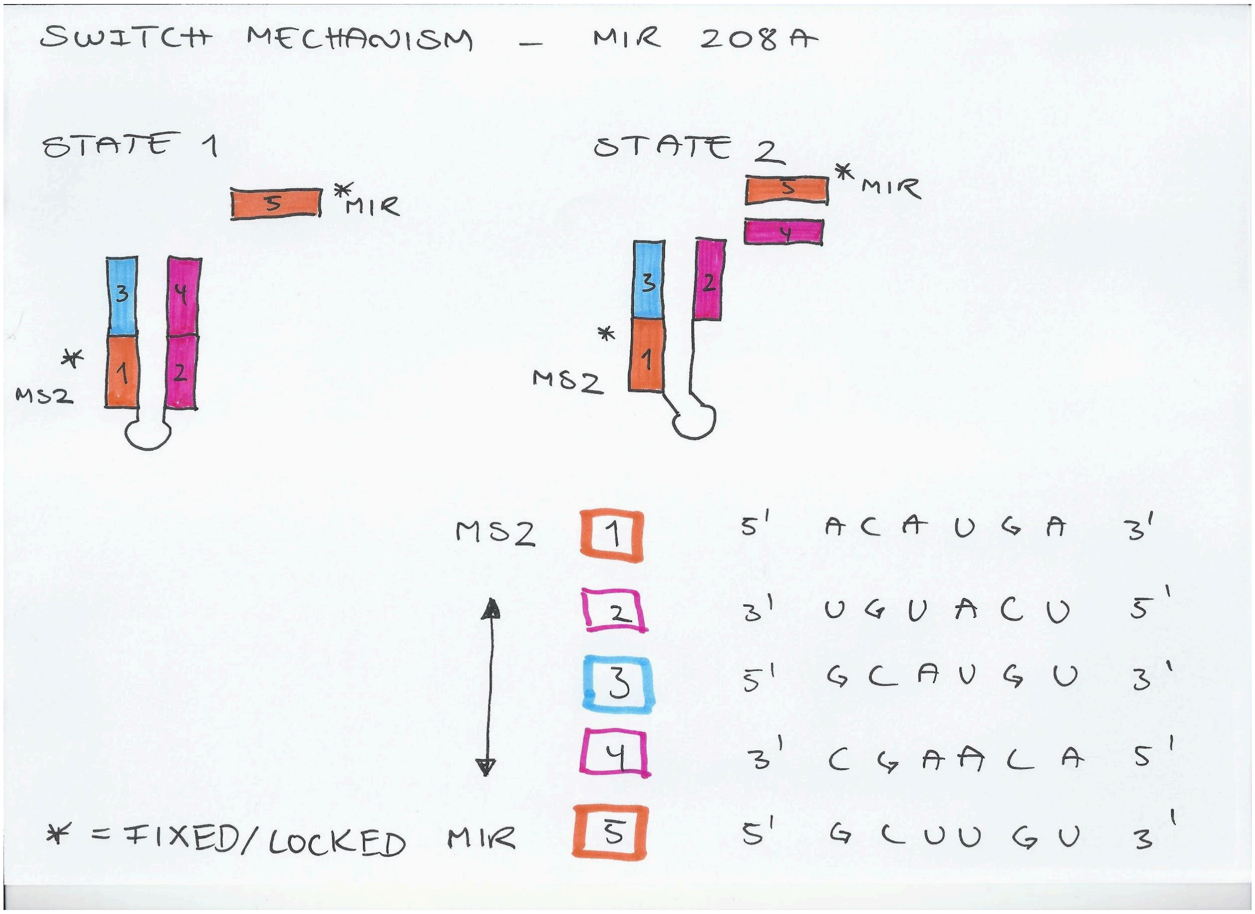

I have described how the design follows a particular word change game. You can read more about it here.

Here is a solve for the current lab that is inspired from the word game solving strategy.

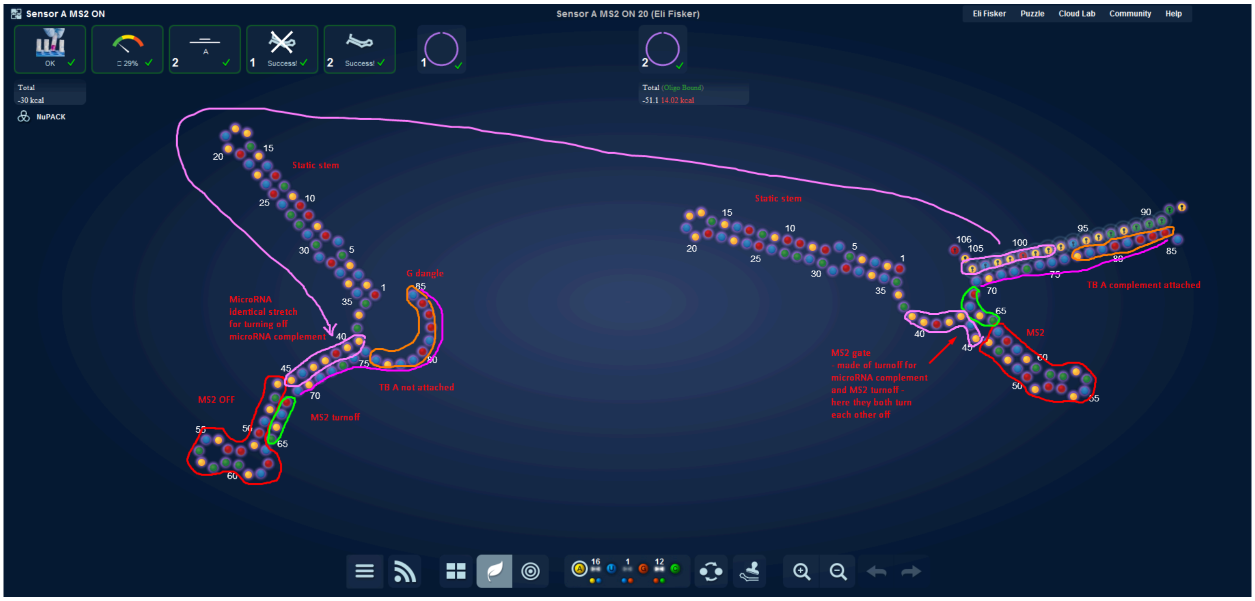

I have made a drawing of the schematics of the folding.

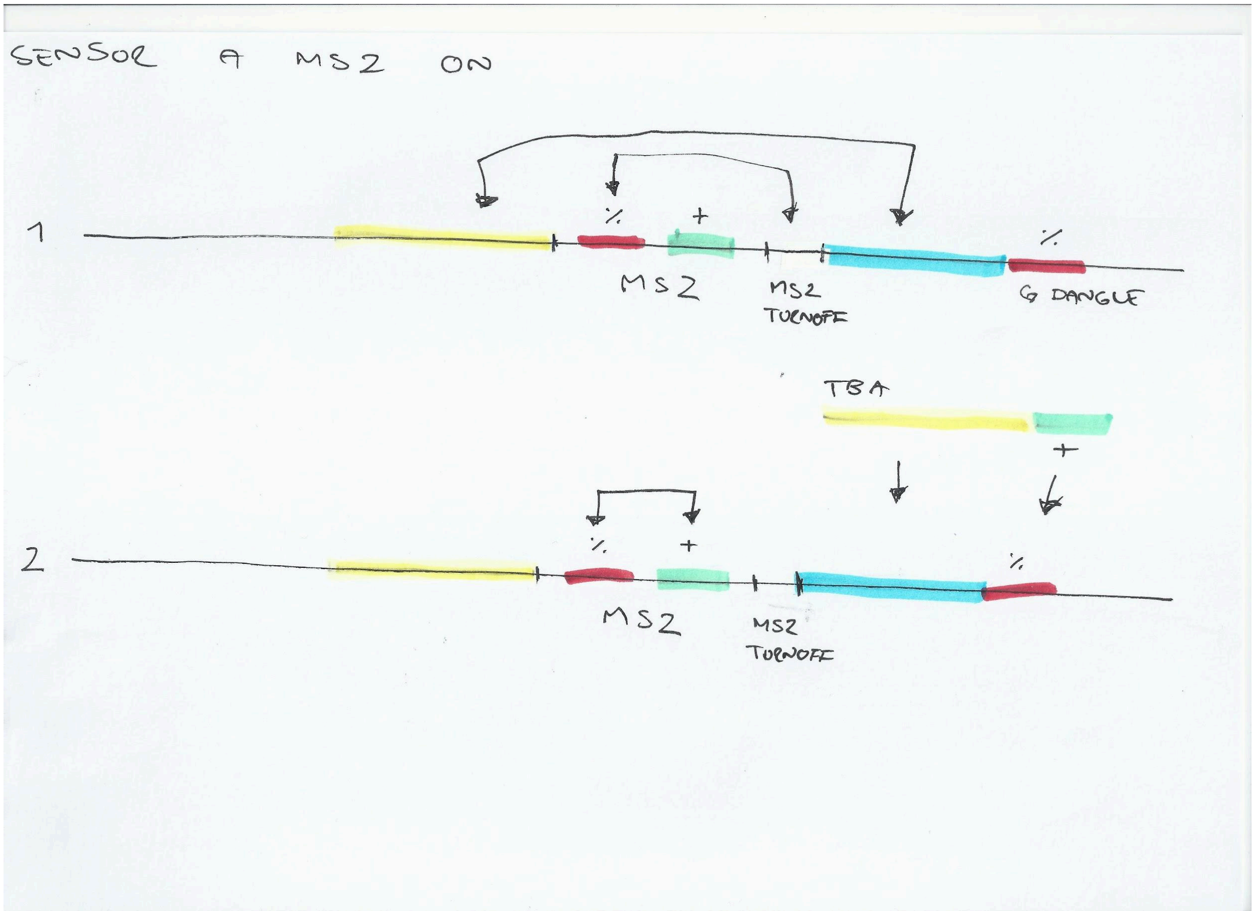

Basically the idea is to have like 5-6 bases after the MS2 for a MS2 turnoff. I'm using the same MS2 turnoff sequence that hit strong through in the winning designs for [Sensor for hsa-mir 208a](#) lab. **CAUGU**. Here is an example with one of the winners carrying this pattern. (Based on jandersonlee's first round high scorer)

Example below

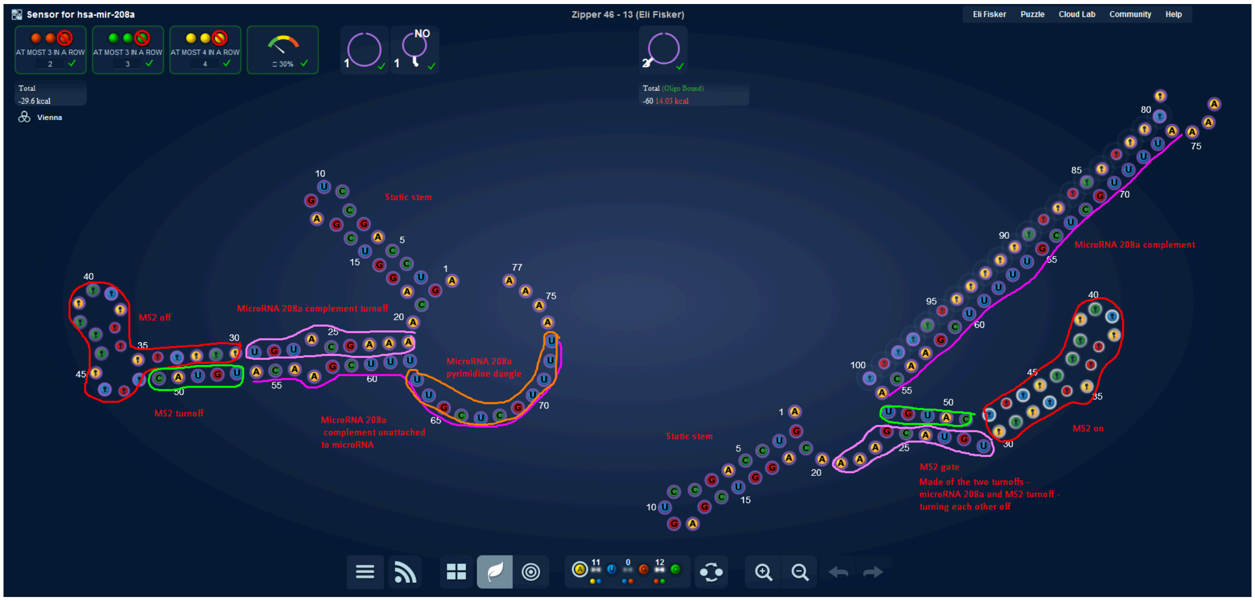

http://www.eternagame.org/game/browse/5750136/?filter1_arg1=5801071&filter1_arg2=5801071&filter1=Id

Then before the microRNA, put in a sequence that is identical to the sequence in the end of the microRNA. I put in bases that mimics the 5-6 bases 99-104. That way I ensure they are complementary to the microRNA and I can thus use this stretch for shutting down the microRNA complement, to hide it a bit away, when the microRNA isn't around.

I have come to think as word change gaming and MS2 gates as central to single microRNA input designs.

MS2 gate needed for single input microRNA labs

I think I now understand why our past microRNA labs have such a strong want for longer MS2 gates. Search for the section:

[MS2 gates in the microRNA labs](#)

Short sum up: My latest advice for single microRNA input labs is to make MS2 gates. The pattern jandersonlee made for first microRNA round, I think I know why it is needed. The long MS2 gate is simply a way to secure that the MS2 is force held from both sides, which means a

better chance for turning it off and on. The word change gaming complementarity of letters between the switching stretches, is just a way to get it glide also.

Sensor A MS2 ON - Lab results

I got so lucky that the design I used for demonstration above ended up as a winner.

Score 100%

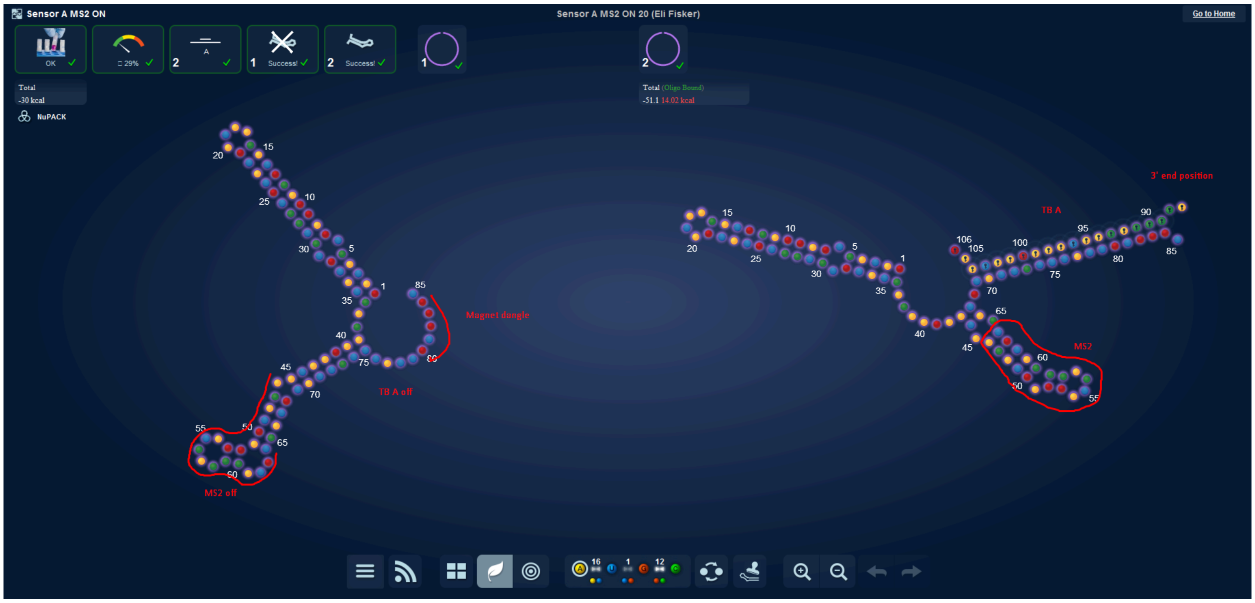

http://www.eternagame.org/game/browse/6296745/?filter1_arg2=6325389&filter1=Id&filter1_arg1=6325389

Overall lab trends: Design complex placed at 3’ end of the RNA sequence. The Sensor B MS2 ON allows the same trend.

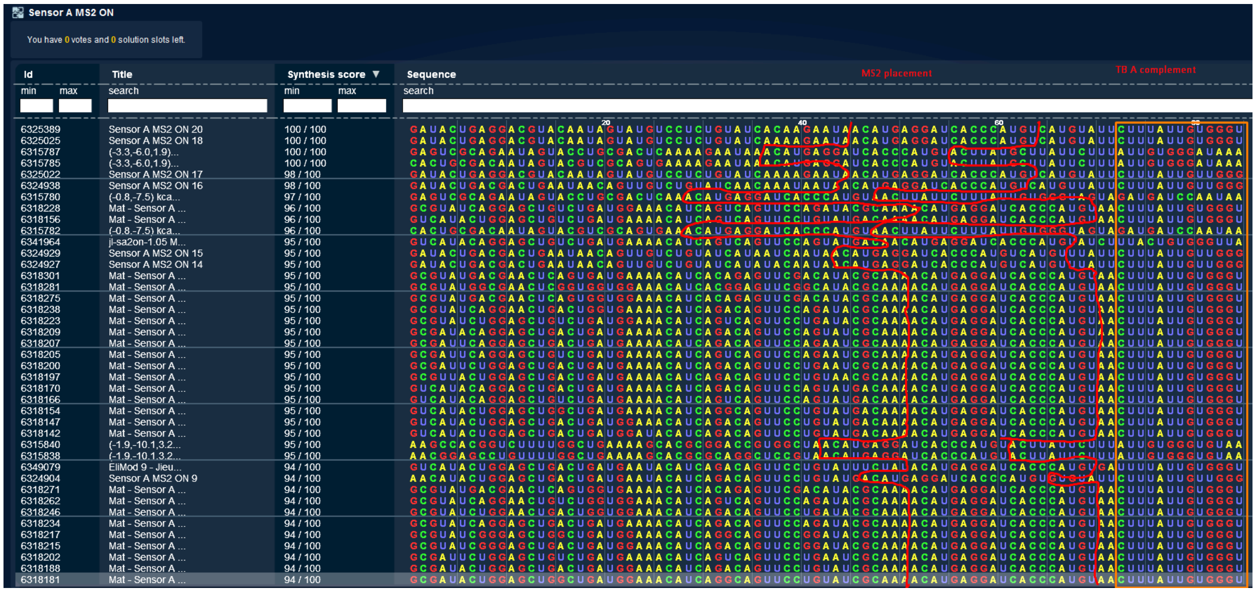

For more analysis see the section, Sensor A MS2 ON, I wrote on this lab.

Sensor A MS2 OFF

Late or early dangling bases?

I put the TB A complement at ends of the RNA. Then I put a pyrimidine stretch in, that is complementary to both a part of MS2 but also slightly to the TB A complementary part. What I wished to achieve was to turn off both MS2 and TB A, but taking turns. That way I get the microRNA complement after the MS2, as it seems to prefer, and the MS2 a somewhat middle position as it also prefers.

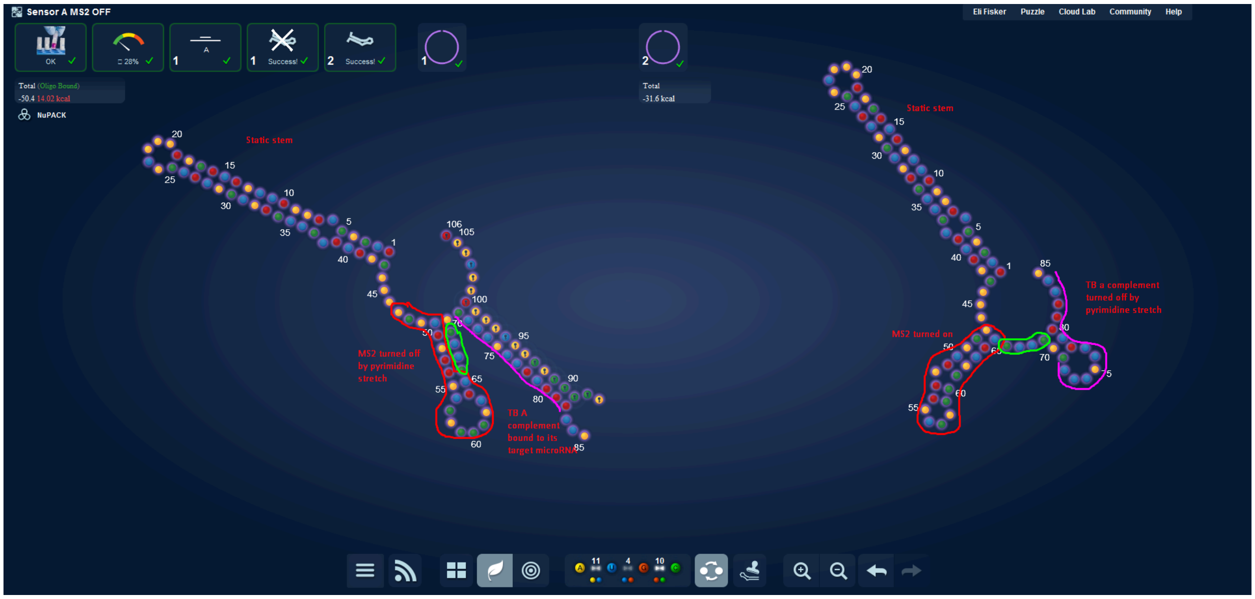

However we have no lab results from labs where the microRNA is to bind up in the first state. So I consider what Omei did with his lab designs in this lab, a good bet at what could actually work. I especially like that there is a pyrimidine dangling stretch of bases early in the design. Which is second best if one can't make a late end dangle.

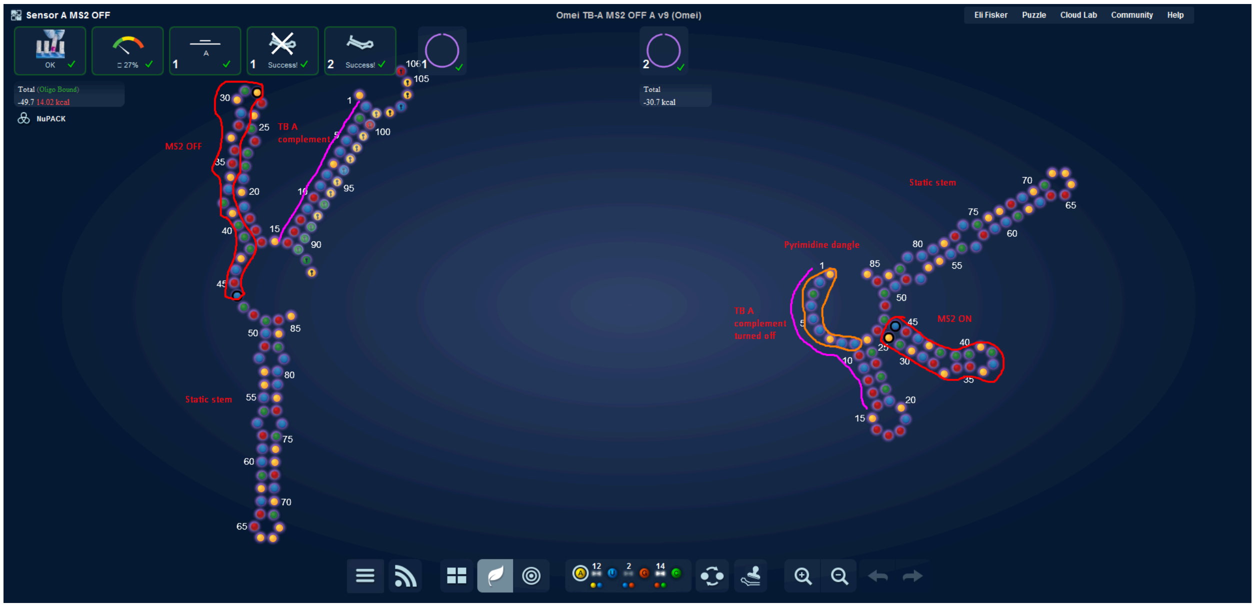

Why I think an early dangle of C's and U's could just be worth it for this lab, is since the microRNA needs to be bound in 1 state, we need to kick it off for the second state. And then it is probably a good idea placing the microRNA at its less favored spot which should make it easier kicking it out.

Another thing worth noticing are the sets of magnets that fits with both the microRNA and the MS2. The turnoff is basically mediated by a hairpin in the middle that has a set of C's and a set of G's that are pairing with each other in state 2 but pairing with the microRNA and turning off the MS2 in state 1.

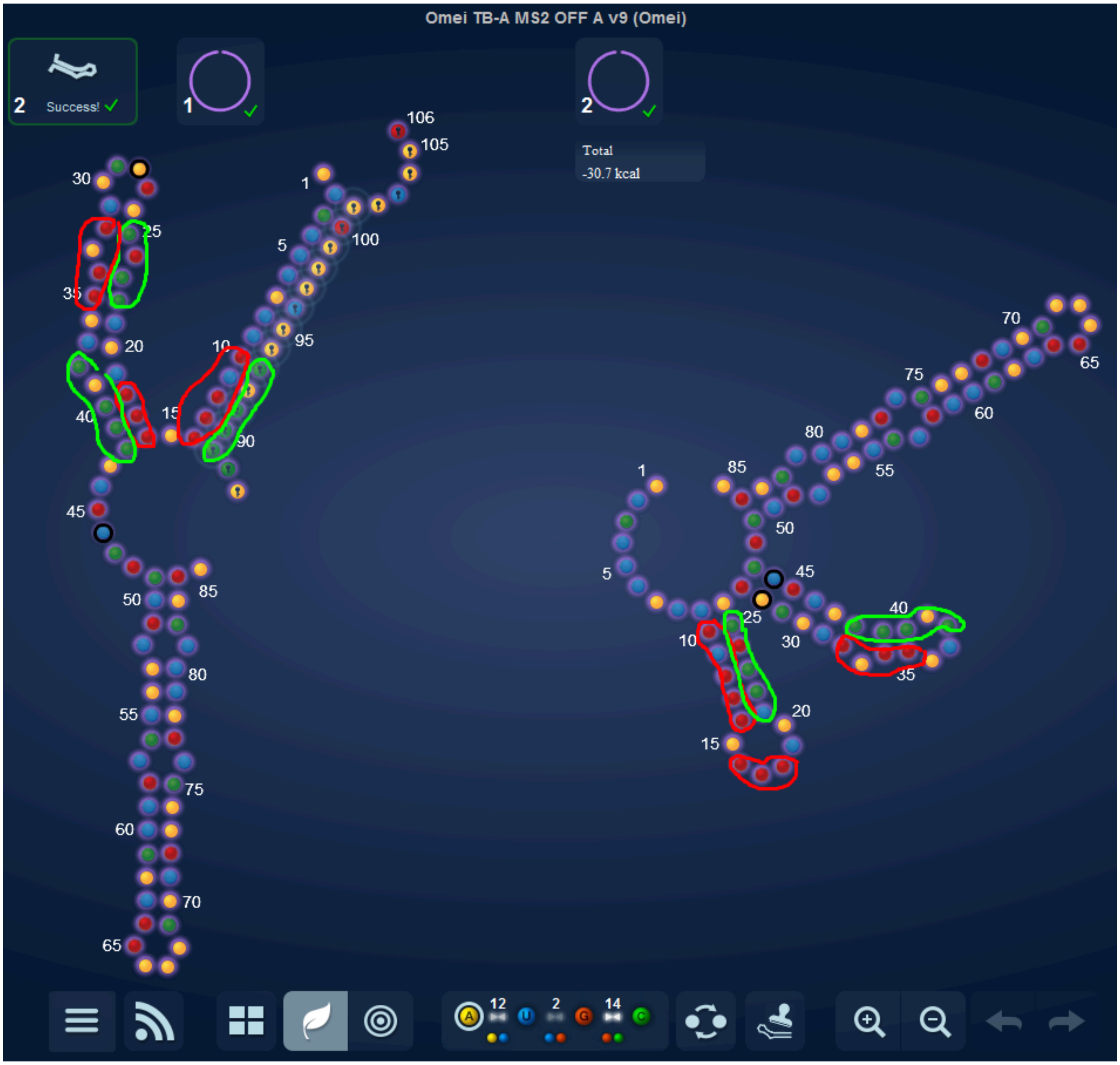

If the turnoff hairpin gets too long and have more GC pairs, it runs a risk of being too stable to want to get to switching.

Similar, if the MS2 turnoff is very long, it is likely to get stuck with the MS2 and not letting it go. And of cause if it is too short too or too weak, then it can not hold the MS2. Which is why the MS2 almost always takes a few C's in its turnoff sequence or sometimes also a few G's. As C and G bases are those that makes the strongest connections of the basepairs. Most of the time

a pyrimidine stretch will do the trick for turnoff. More rarely will a G turnoff work. But we will need to figure out when to use it, as the lab puzzle is not always making it optimal to use a pyrimidine stretch.

In many of the FMN and MS2 labs, it typically made double sense with using a pyrimidine turnoff sequence to target the same purine stretch in the FMN and MS2. Now we don't have a FMN around anymore, so this may open up for other turnoff sequences being needed, as their main criteria for use is that they can target a section in the MS2 and make it turnoff.

Here is a lab solve the word change game way. Notice the small stem folding in front of the MS2 in state 2 (Right).

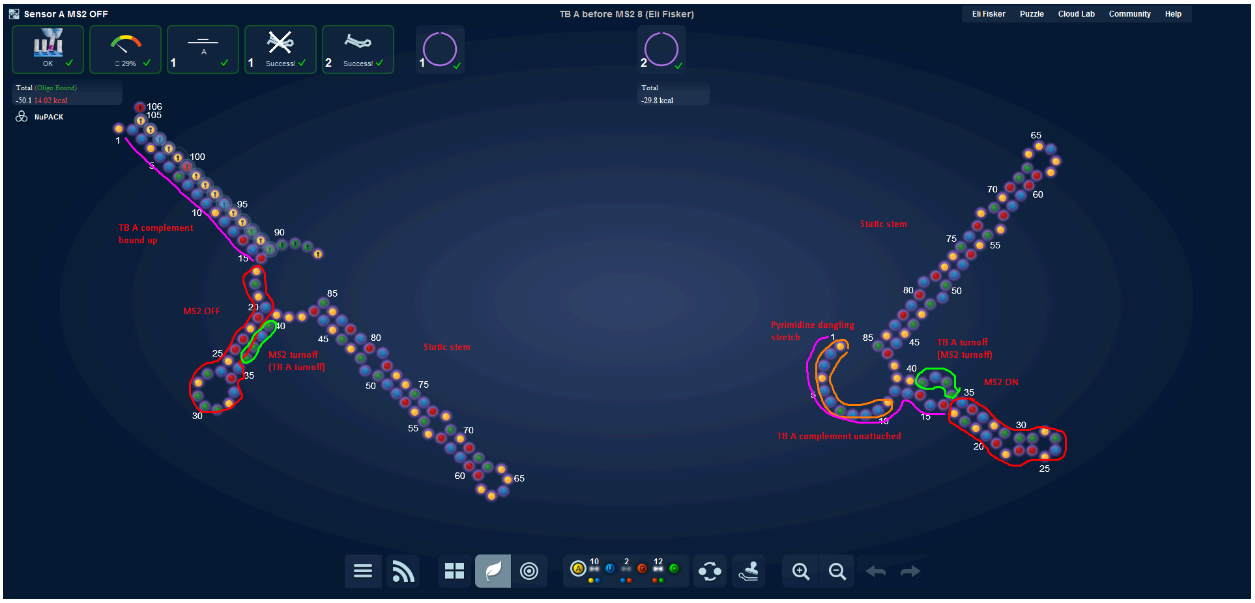

Sensor A MS2 off - Lab results

Also here I got extremely lucky and one of the designs I demonstrated ended as a winner. I was using one of Omei's design as inspiration.

Basically the microRNA solving strategy with word change game is working well for both ON and OFF single input puzzles.

Score 100%

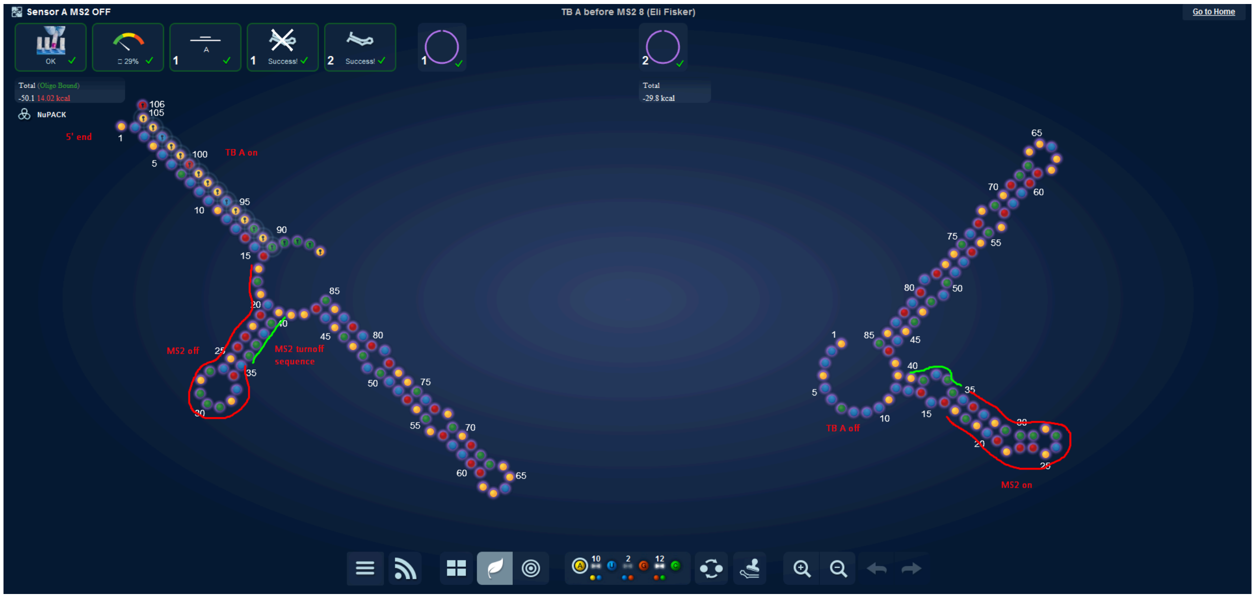

http://www.eternagame.org/game/browse/6296746/?filter1_arg1=6330006&filter1_arg2=6330006&filter1=Id

While there were few winners, the starting trend for this turnoff lab, is to have the TB A complement placed first at the 5' end at the worst spot, so it is easier to kick off.

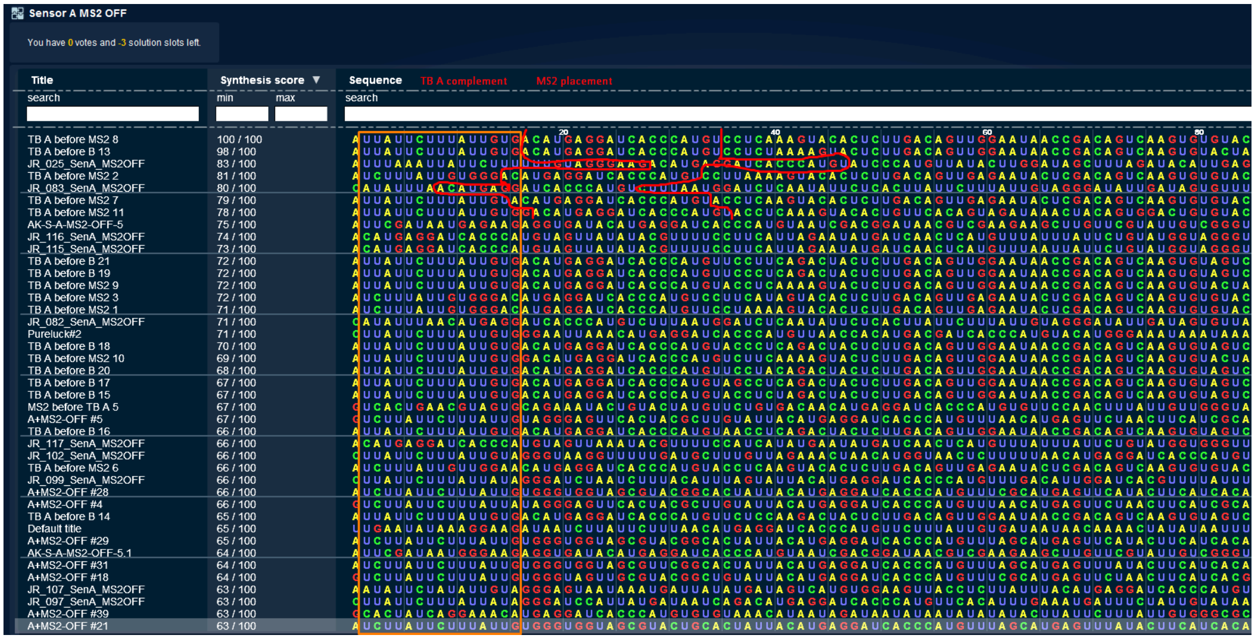

Both the sensor A and Sensor B off puzzle, seems to prefer an early (5') position in the design. And not as I kind of expected from the earlier mir labs, at the late end.

Just as in the original mir 208a lab, these designs show a want for a dangling end. And dangles work in both ends, but seems easier to get to work at the late end, just as in the mir lab.

For more on this lab, read the analysis I put up in the A/B lab discussion, search for Sensor A MS2 off.

Sensor B MS2 ON

In this lab I’m going to demonstrate the steps I take towards getting to a solve and try explain why I do what I do.

State 1

TB B off
MS2 off

State 2

TB B on
MS2 on

Ok, since TB B and MS2 both needs to be gone in state 1, a potentially quick and dirty way to solve is to check for direct complementary between TB B and MS2 and make them both turn each other off.

This will get the strongest overlap. This may be too much, as there will be little single bases on either the MS2 and the TB B to get them detaching from each other.

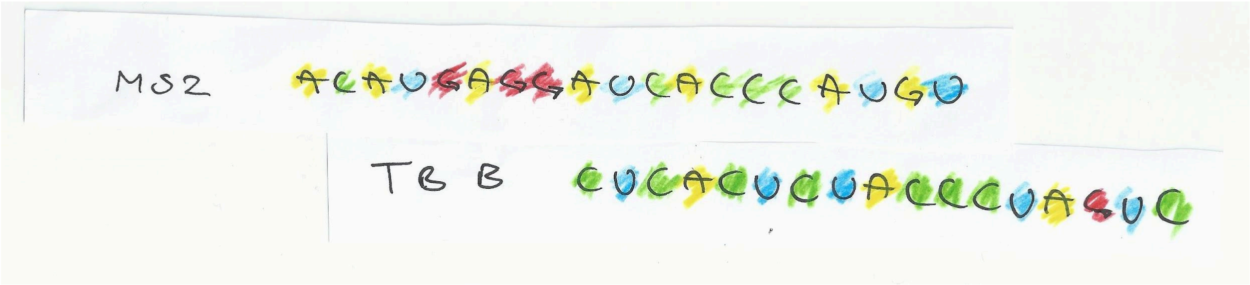

Which is why this weaker bind may be more interesting.

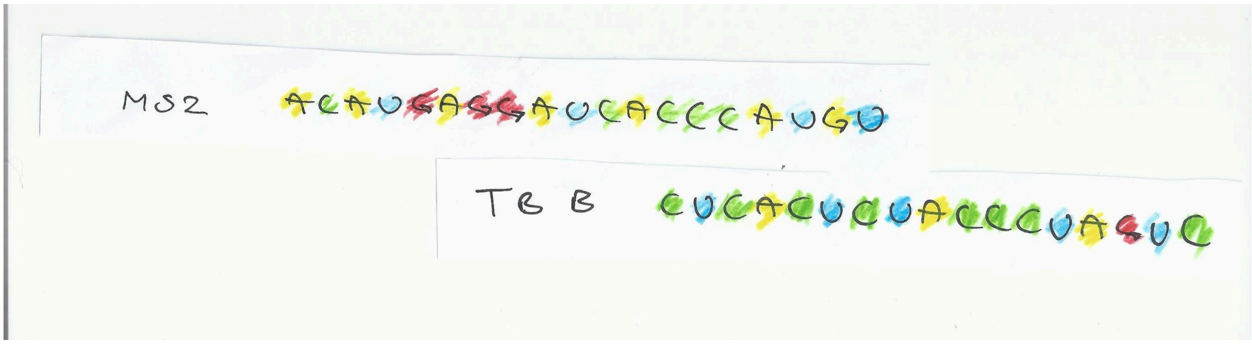

This is too weak to work. Ok, quick and dirty style will not do for this lab.

Next strategy is to put MS2 in the middle, the TB B complement at the end of the RNA design and then try make a MS2 turnoff/TB B complement turnoff sequence somewhere in between them. And when I have a solve, I will move things closer together for it to become a good solve. The MS2 usually want it's turnoff sequence as next door neighbour for it to work - sometimes short distance will work too. Long distance MS2 turnoffs can work in rare cases but I suspect they are going to be less effective and of more use when we have more microRNA inputs. [Yup, that seems to be the case. In labs with two inputs, further distance between MS2 and MS2 turnoff has been seen and far distance MS2 turnoffs come of more use. The MS2 turnoff also tends towards becoming longer.]

I hardly got the microRNA complement in before there was a solve. So as you can see, this can be a quick way to get to a puzzle solve.

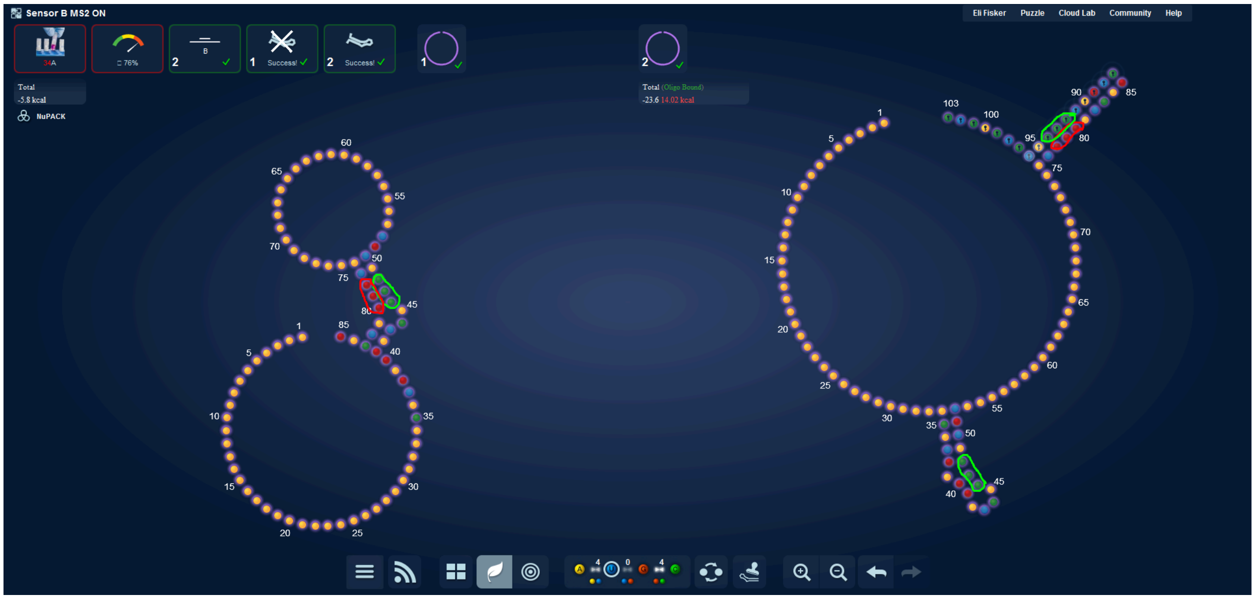

As can be seen the microRNA complement has 3 G's which functions as core attractor between the MS2 and the microRNA.

Sum up: Place the MS2 in the middle, start from absolute end of the RNA sequence and put in part of the microRNA complement. Works for two microRNA inputs too. Just put in one of microRNAs complement at absolute start and end of the RNA sequence. And it will often get a solve. Afterwards you can always move things closer together.

Now this is not a good lab solve yet. The microRNA complement and the MS2 is way too far apart. Plus in single input labs MicroRNA generally prefer being mostly paired up with the RNA design - sometimes except a bit of the ends of the microRNA. It is first double input labs, that starts to need breaks in the pairing between RNA complement and input.

I will use the MS2 stamper to move MS2 closer to the microRNA complement and I will likely need to make most of the microRNA pair up. But I will move the MS2 first and then figure how much of the microRNA that I can get pairing up. Sometimes if I pair up too much of the microRNA, it gets in the way of solving.

But first I tie up all the loose bases to the left, by forming a hairpin with long stretches of C and G bases. I prefer doing so over using the glue tool. As I will need to change things later anyway.

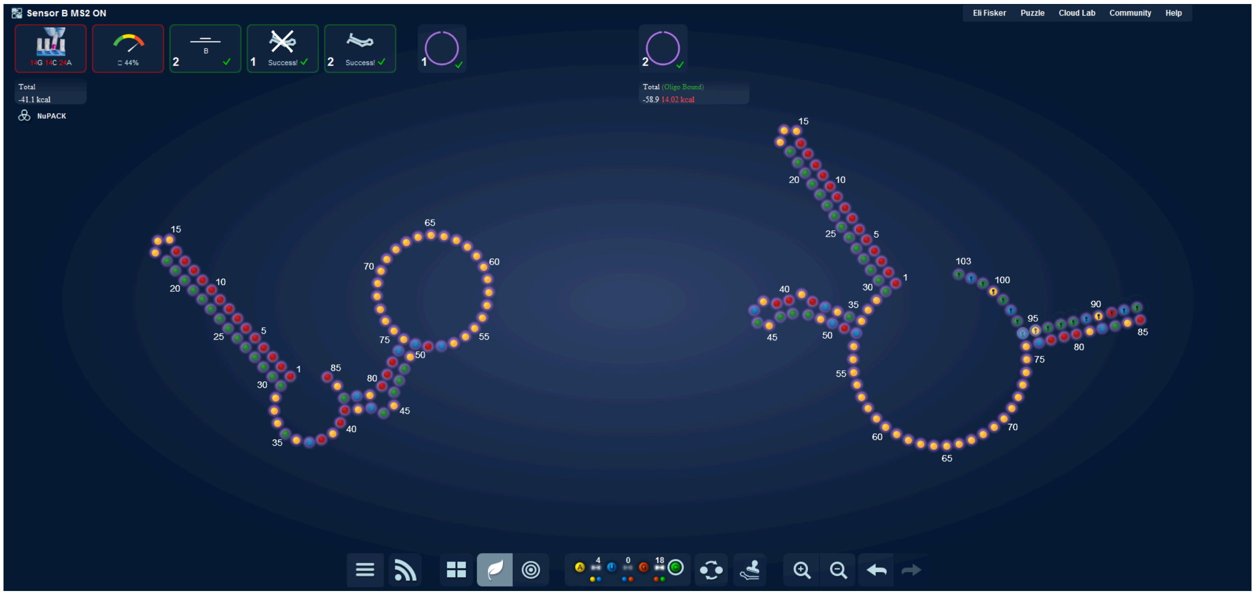

I move the MS2 much closer to the microRNA complement.

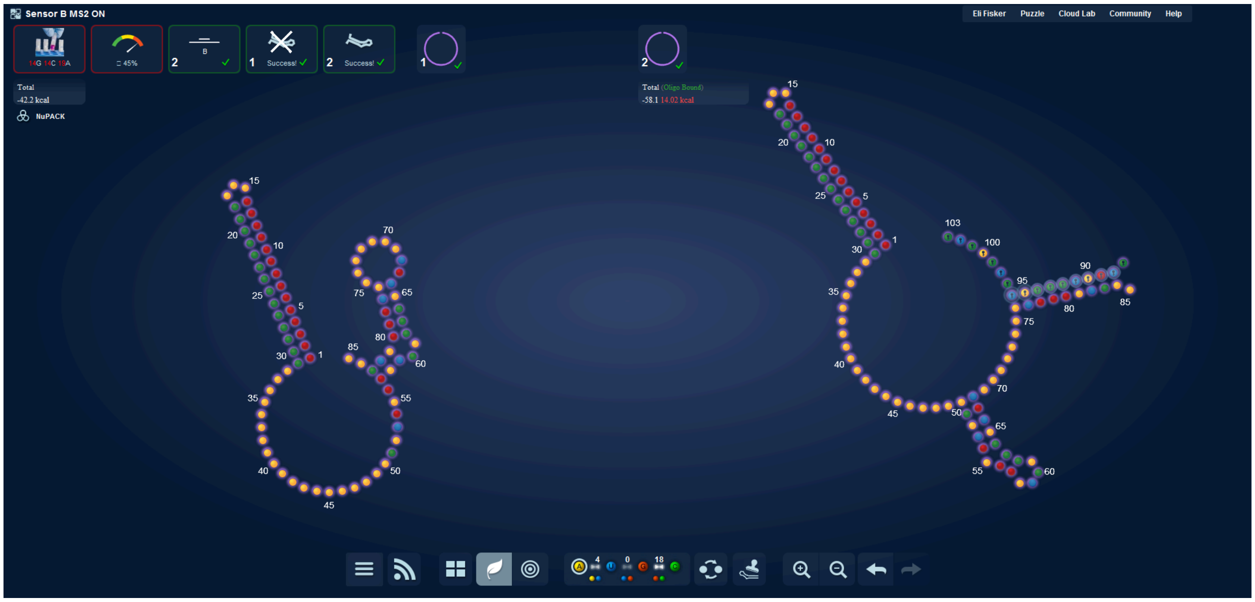

I try put in some more of the MicroRNA complement bases. The puzzle protests and the microRNA can't get turned off. The puzzle got unstable - notice that the MS2 is present in both states and it should only be present in state 2.

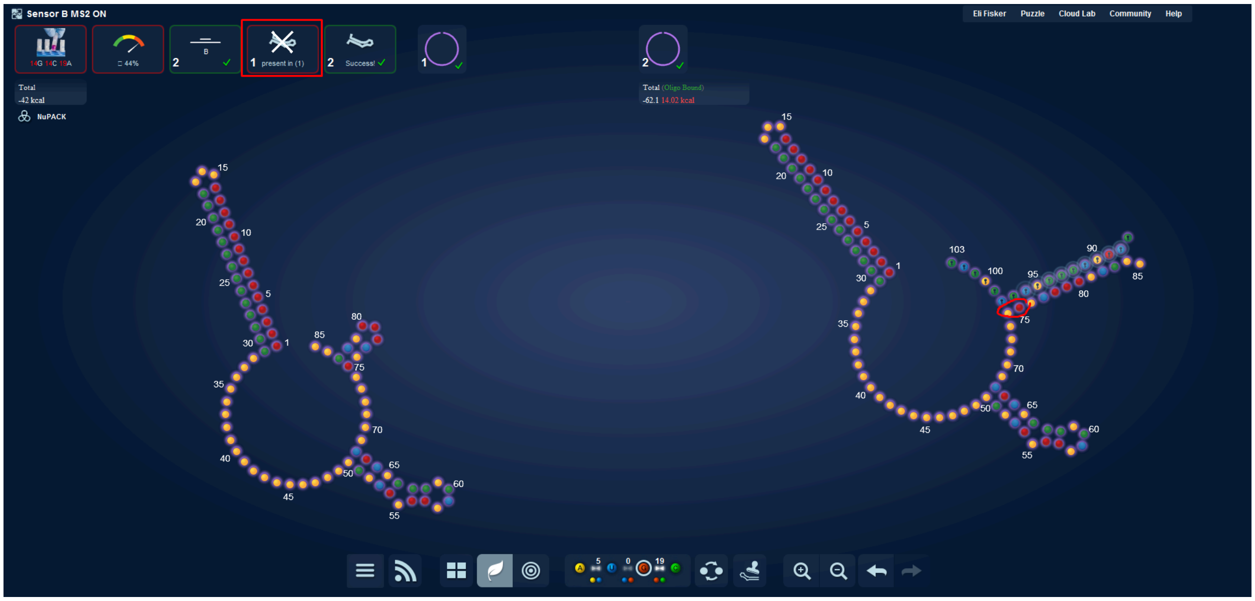

So to add a bit more balance between the strengths of the elements - I do want both the MS2 turned off but also more of the microRNA paired up - I add a MS2 turnoff sequence, as to help the microRNA.

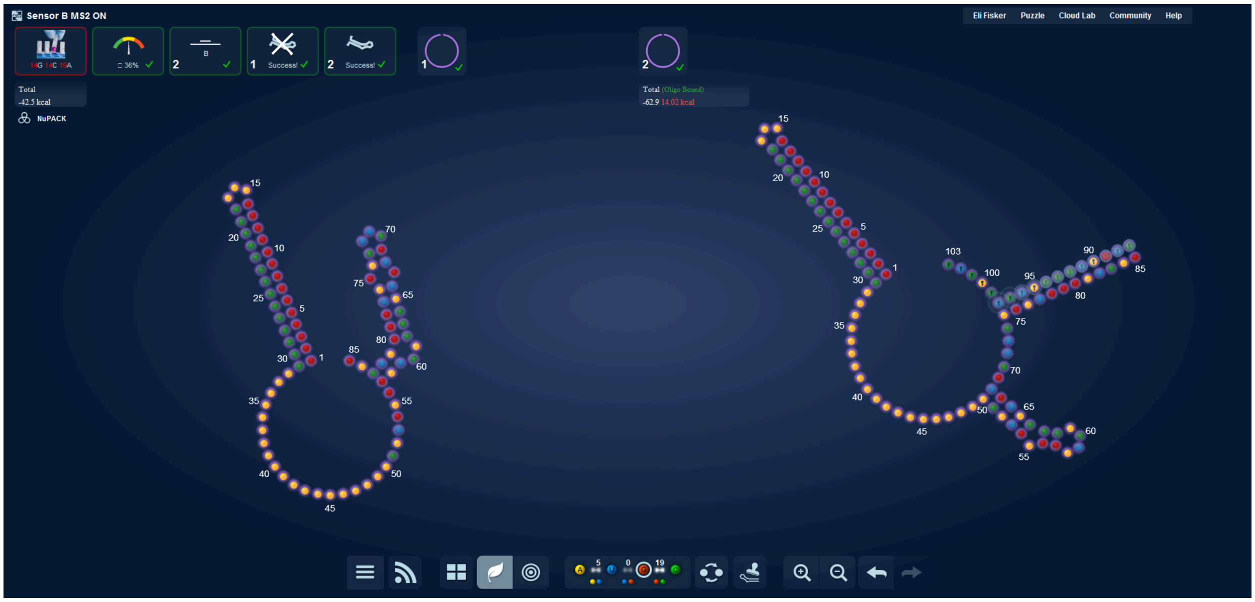

I keep moving the MS2 backwards and move the MS2 turnoff to see how many complementary bases I can make to the microRNA.

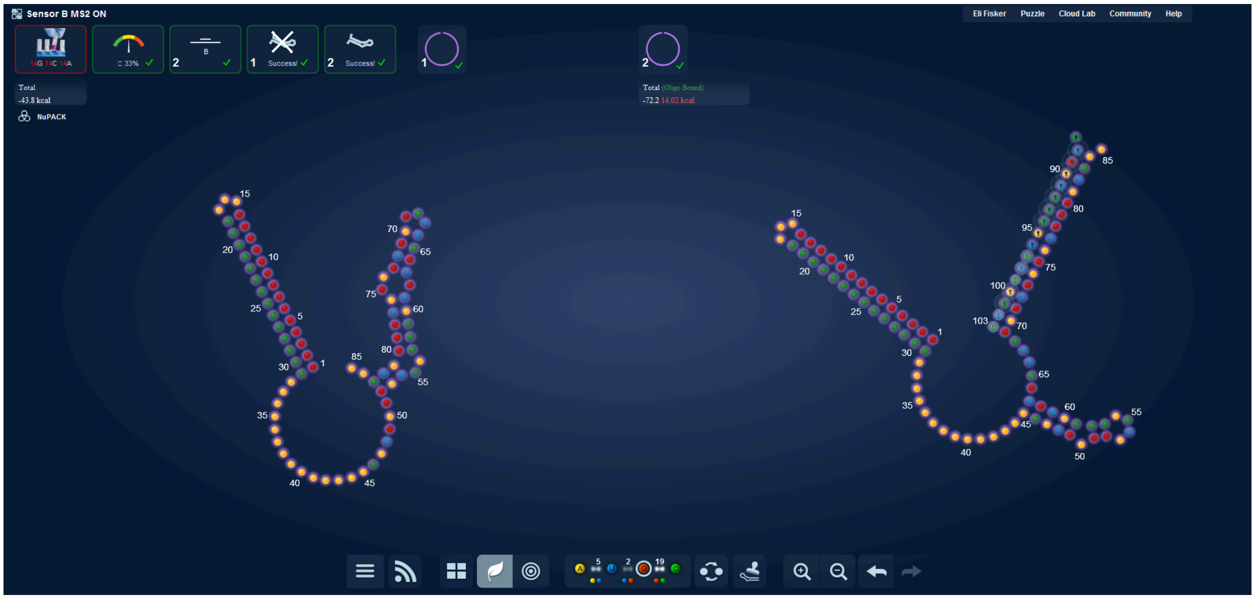

I now have most of the microRNA bases paired up and the puzzle stable. If I can’t get the last ones paired up that is okay. And it is regular useful with a few loose bases at either end. However I still have no dangling stretch of bases in State 1, as an invitation to catch the microRNA.

Actually now I try and see if I can move the MS2 right next to the microRNA complement and I can. The microRNA complement works as a direct turnoff.

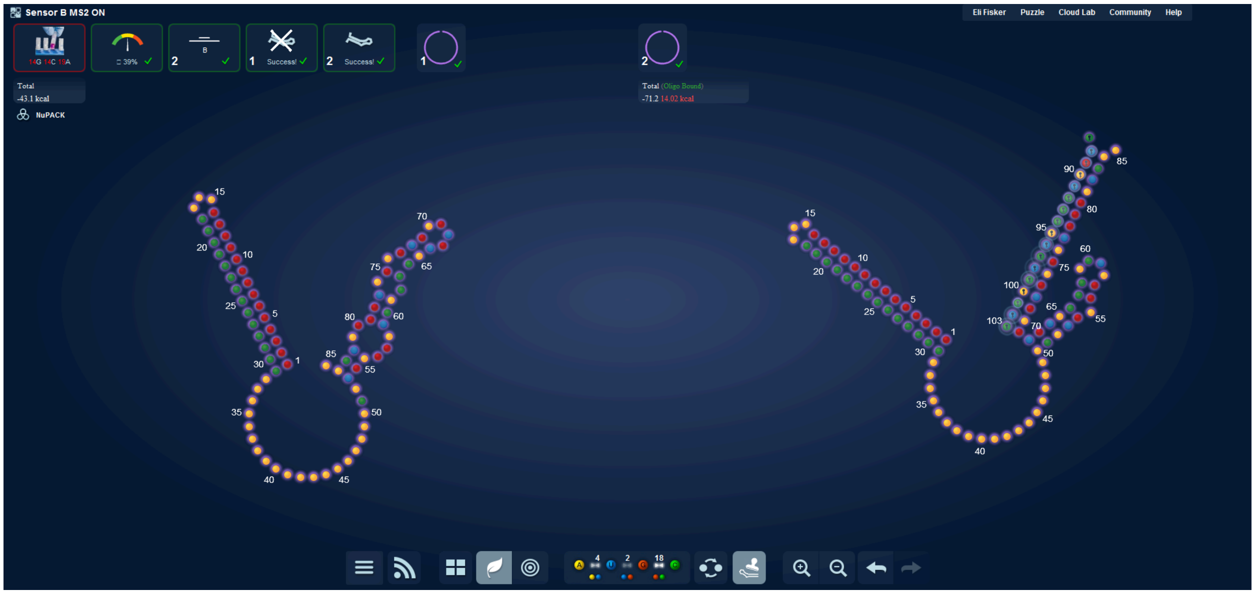

However there are still no end dangles in state 1. A honey trap of single stranded microRNA complementary bases is generally needed in the state that do not hold the microRNA. So what I need to do to get it is to put in an microRNA identical stretch before the MS2, to turn the microRNA complement off. Notice that I left one extra A base before the MS2. This creates a bulge and makes it easier to get the switch to move because the longer a stem gets, the harder it gets to switch. A 1-1 loop will also often do.

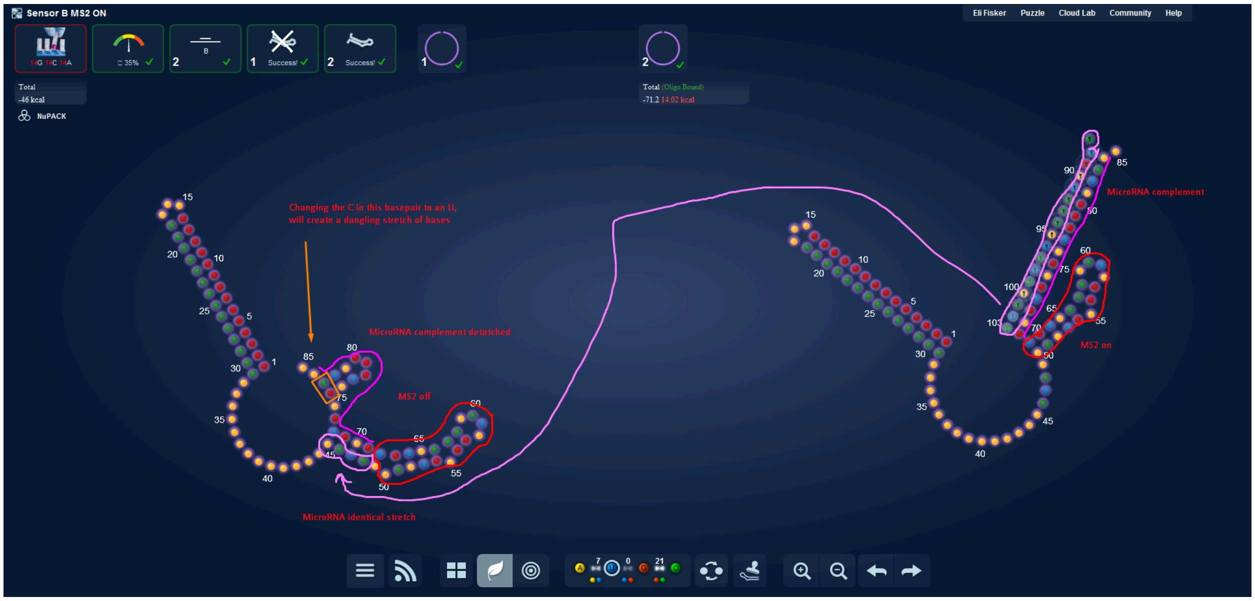

At ends of the RNA sequence, the dangle is not freed up as it forms a stem with itself. I change a C base in the stem to an U and voila - end tail dangle!

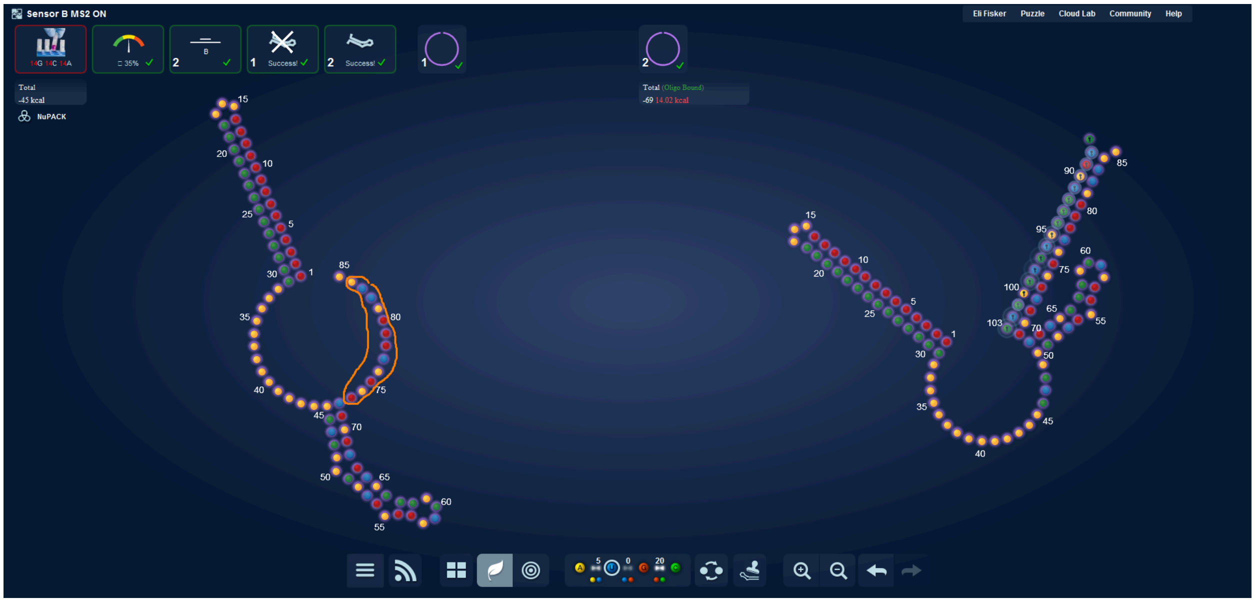

Now there is a dangle (orange highlight above). But it is rather long. So I might still need that MS2 turnoff sequence that I removed. Also as the stems are getting so long that the MS2 would need some help getting turned off, as rather here what might be happening is rather a split of two bases at the end of the MS2, which is rarely enough to get the MS2 to get turned off. So I'm putting the MS2 turnoff stretch of 5 bases back in again.

A 1-1 loop mismatch is good to have close to the MS2 that is turned off. Like the A bases marked in the winning microRNA example below:

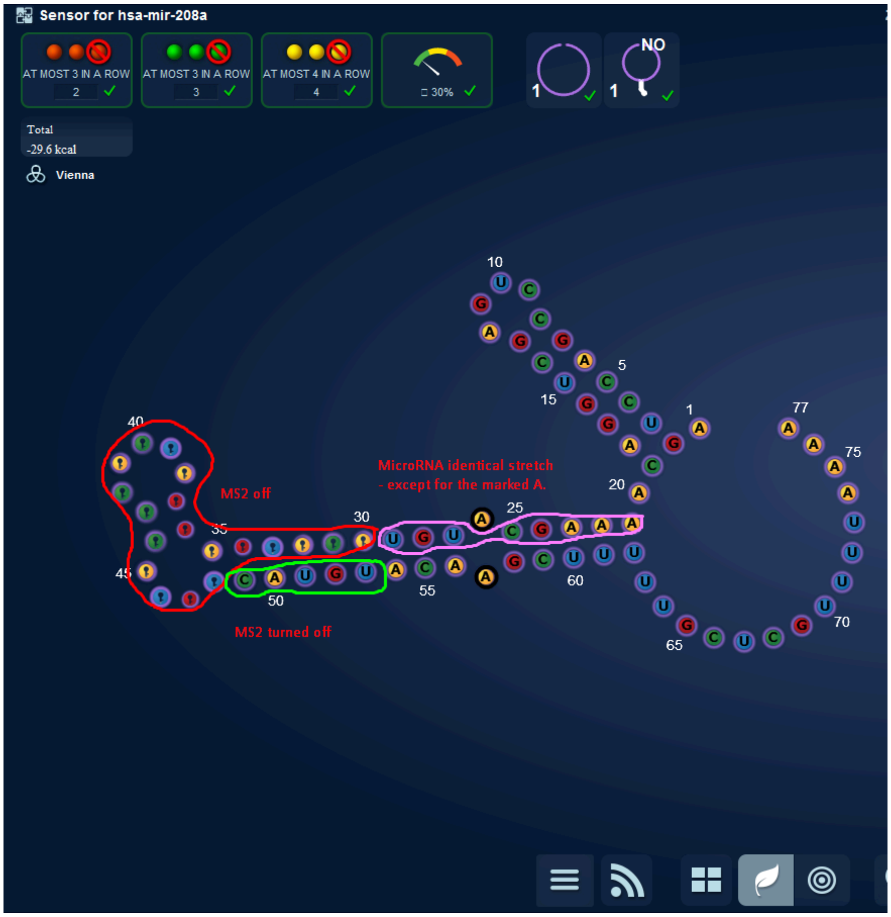

Fourth base (red arrow) in the microRNA identical stretch I make weaker by making a GU pair, as I couldn't make a 1-1 loop mismatch without getting the stem in front of the MS2 splitting.

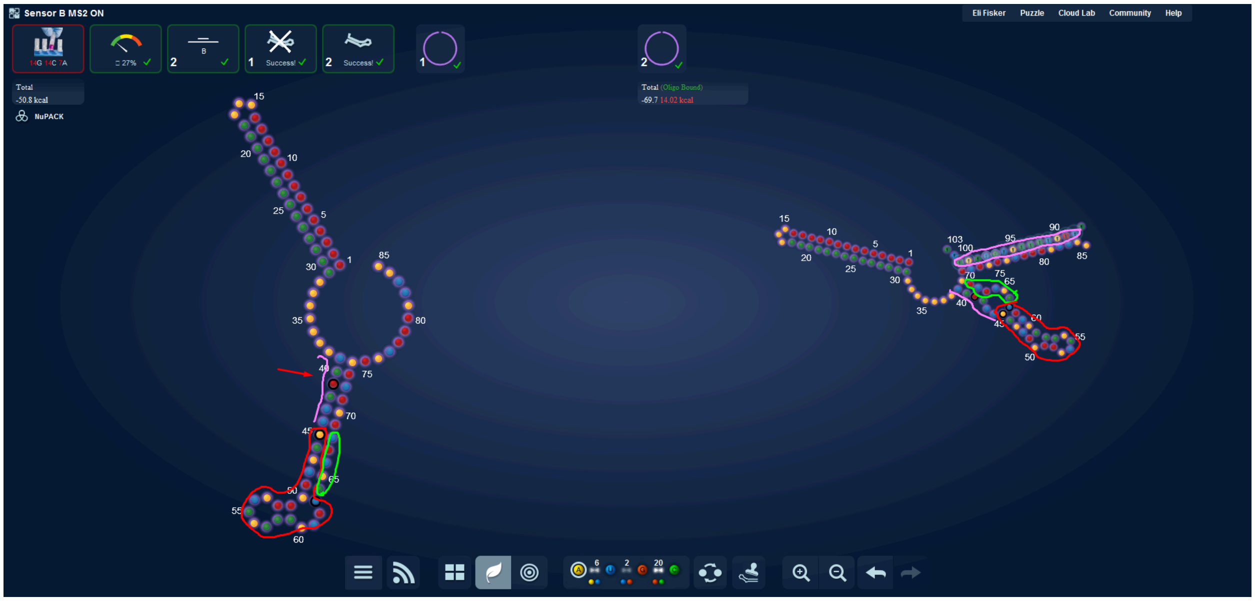

Also notice that I don’t use all the C’s for the microRNA identical stretch as are in the original microRNA. If I make too strong a stem before the MS2, then it won’t get moving. And if no move - no switch. Even though the microRNA labs have shown a far greater tolerance to having a rather long MS2 gate in front of the MS2.

Ok, I’m about happy with the solve of the main part of the design for now. Now I need to make a good sequence for the static stem and give it around 3-4 A bases between the static stem and the active switching design. These bases I call a Salish hinge. [Salish noticed] that they seemed to be beneficial at a specific part of switch designs.

For now I think I see them typically turn up between static and switching part of the design. I think this works as a spacer area between different elements and as such help them stay separate which is helpful when we don’t want them to interfere.

Notice that the switching elements for a good deal like to stay really close together - perhaps this helps them get moving and switching. The Same State labs are different though - there switching can happen over a distance - although not too long either. But Same State labs have also got the energy input from both the MS2 and the FMN aptamer to help it get folded, whereas the Exclusion labs have only the FMN to help them get folded and the MS2 pulling in the backwards direction.

This is what I ended up with and now this is what I’m going to make my lab solutions over with some mutations and slight moves of elements.

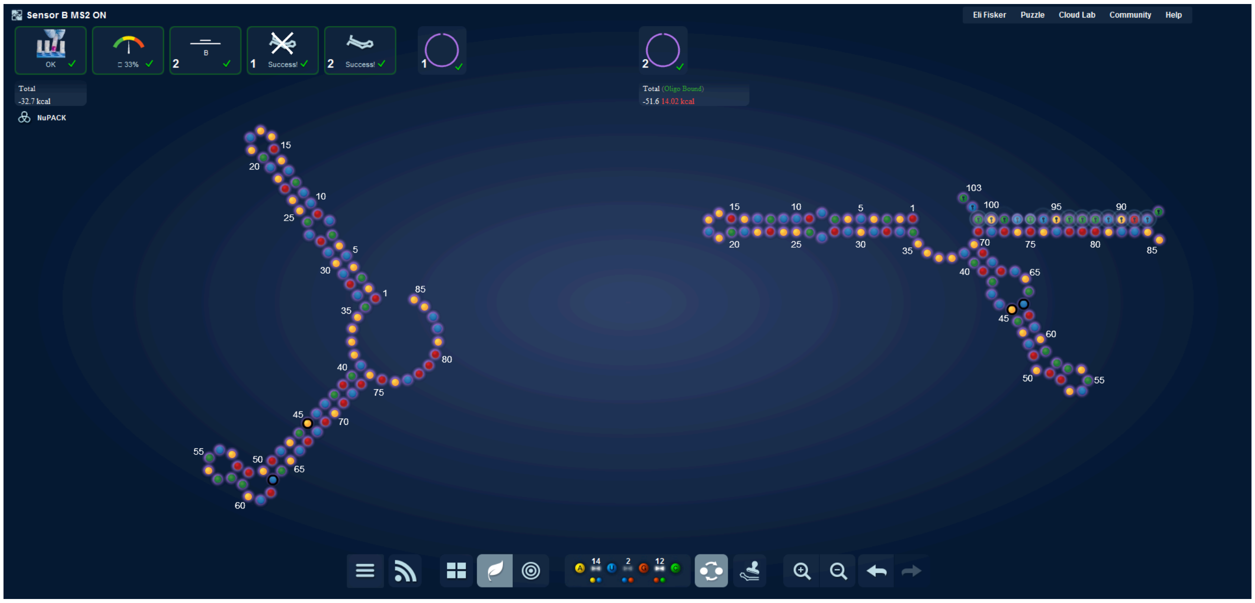

Sensor B MS2 ON - Results

What I demonstrated above, did work. This solve is very close to the one I showed.

Score 96%

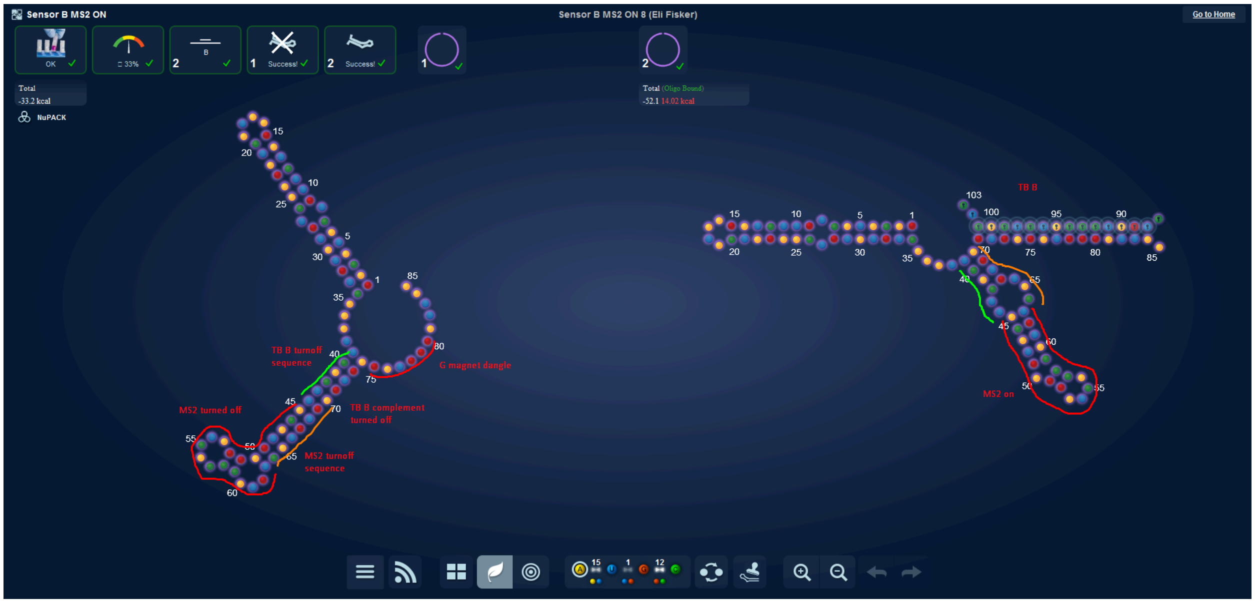

This design follows exact same patterns as the winners from the Sensor A MS2 ON lab.

But there are several other positions of the TB B along the RNA design sequence, that gets a winning score too. I think because the TB B sequence is so strong, it is allowed a wider range of options. It simply takes more to make it unhappy and the lab design to fail.

For more on this type lab, search for the section Sensor B MS2 ON.

Sum up MS2 ON

As can be seen both Sensor A MS2 ON and Sensor B MS2 ON can be solved in very similar manner. Notice that even the same MS2 off sequence (black rings) can be used between the labs. One I took from jandersonlee's microRNA winner in the 208a microRNA lab, that also is a single input and turn on lab. These solutions are even positioned the same way at the 3' end.

Design description

Mimiciking the microRNA winners from the 208a lab

Here my mod of jandersonlee's design as example:
http://www.eternagame.org/game/browse/5750136/?filter1_arg1=5801071&filter1_arg2=5801071&filter1=Id

100%

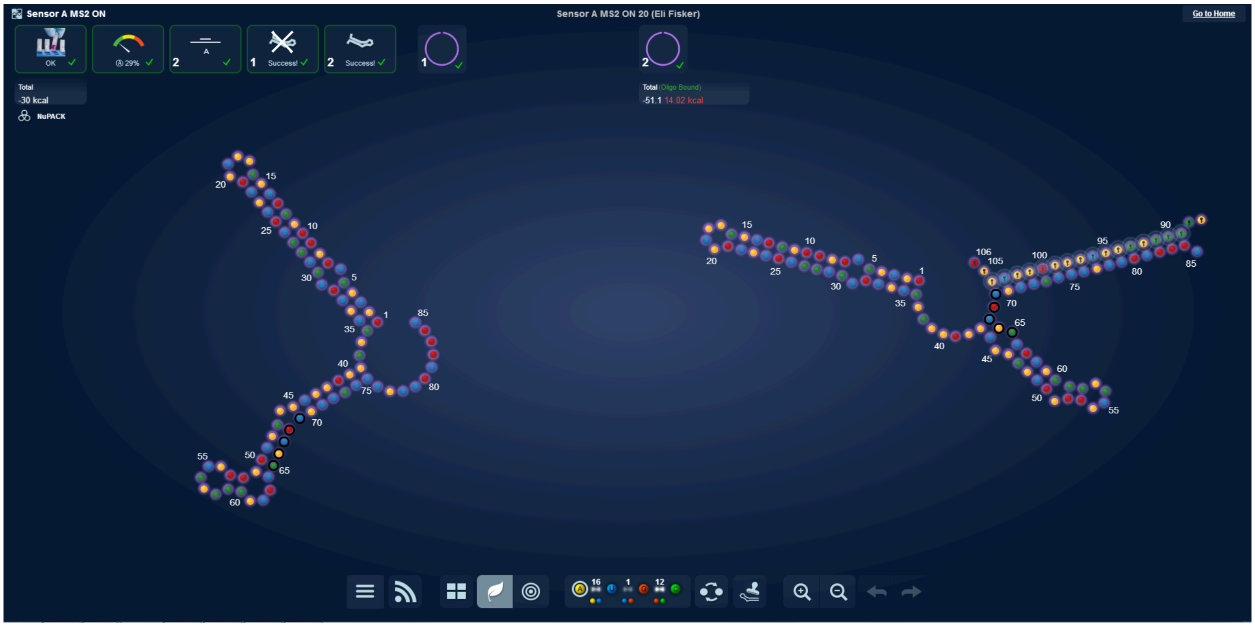

http://www.eternagame.org/game/browse/6296745/?filter1=Id&filter1_arg1=6325389&filter1_arg2=6325389

96%

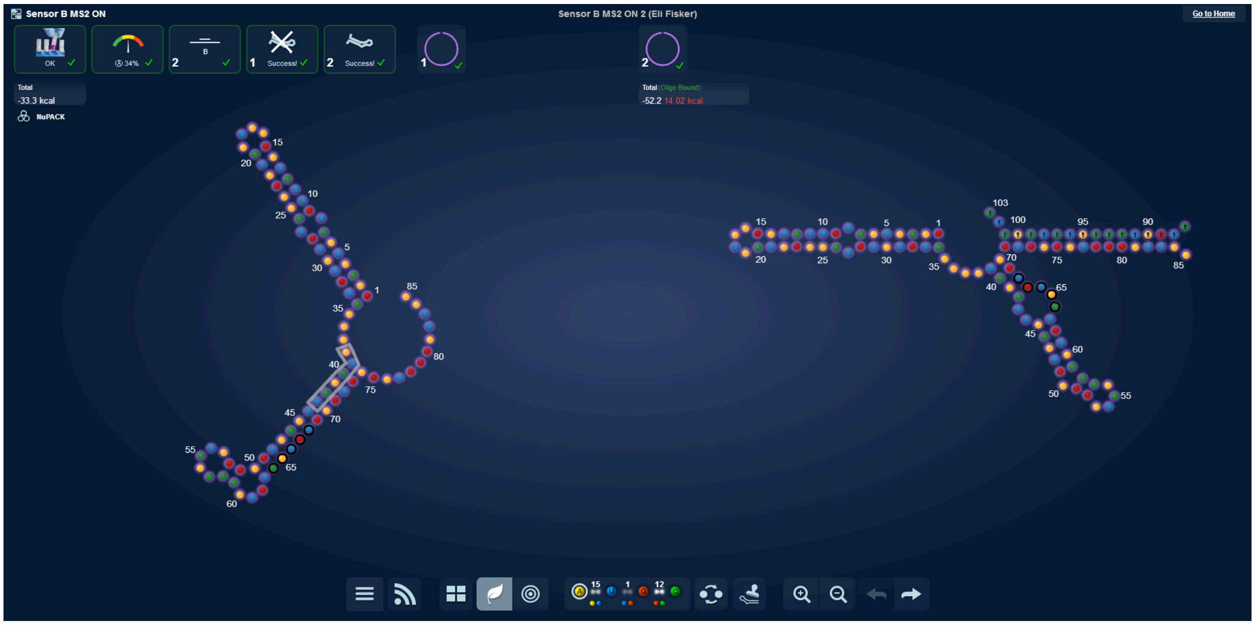

http://www.eternagame.org/game/browse/6296750/?filter1=Id&filter1_arg1=6335015&filter1_arg2=6335015

Sensor B MS2 OFF

[After I have seen the results I have different recommendations for how to make a solve for this lab, than those below. I have added them in the Sensor B MS2 Off - Results section]

Here is a different route to a solve.

Give MS2 a middle placement

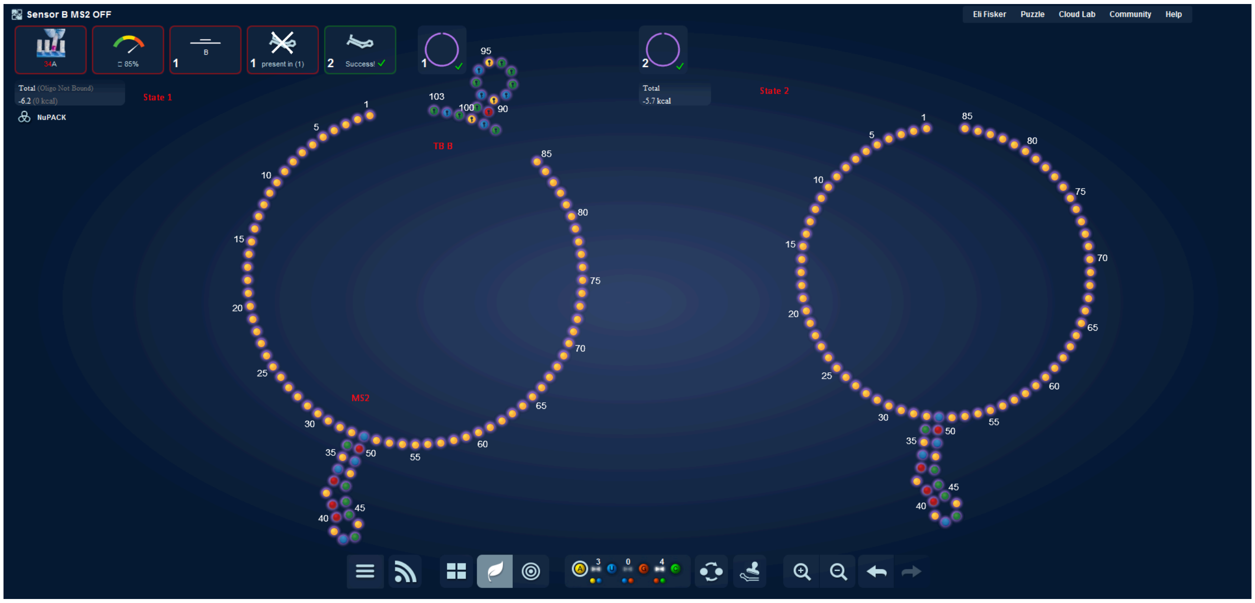

TB B should be on in State 1, when MS2 should be turned off. And MS2 should be on in State 2, when TB B should be off. This means if you can find direct complementarity between MS2 and TB B, then you can use the one to turn the other off, and revers.

There is an overlap while not a strong one, XCXXGXG, between MS2 and TB B.

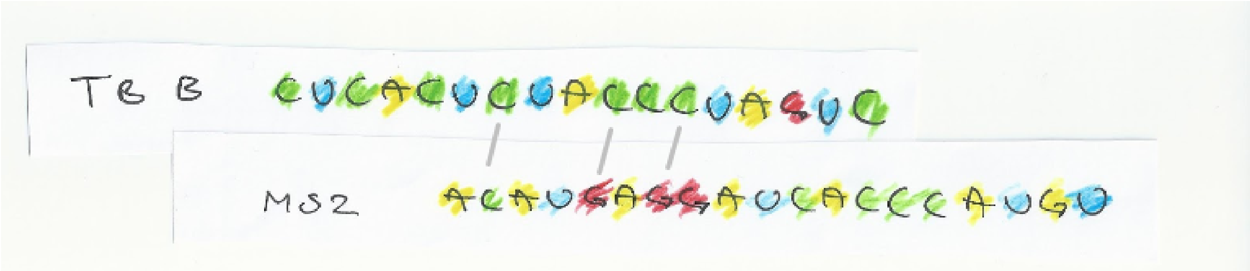

Which means you can solve by continuing the complement to the TB B microRNA, before the MS2 sequence in the RNA design.

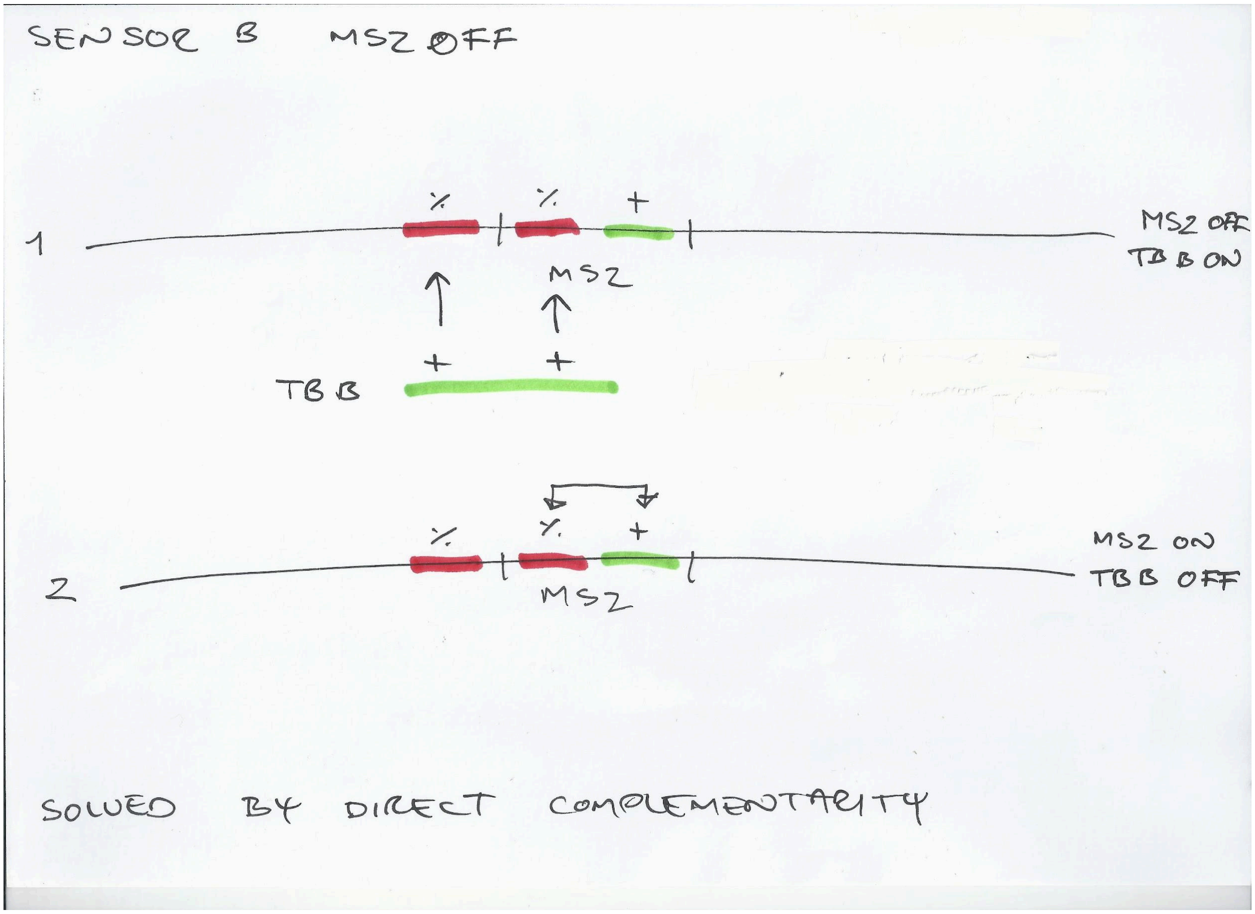

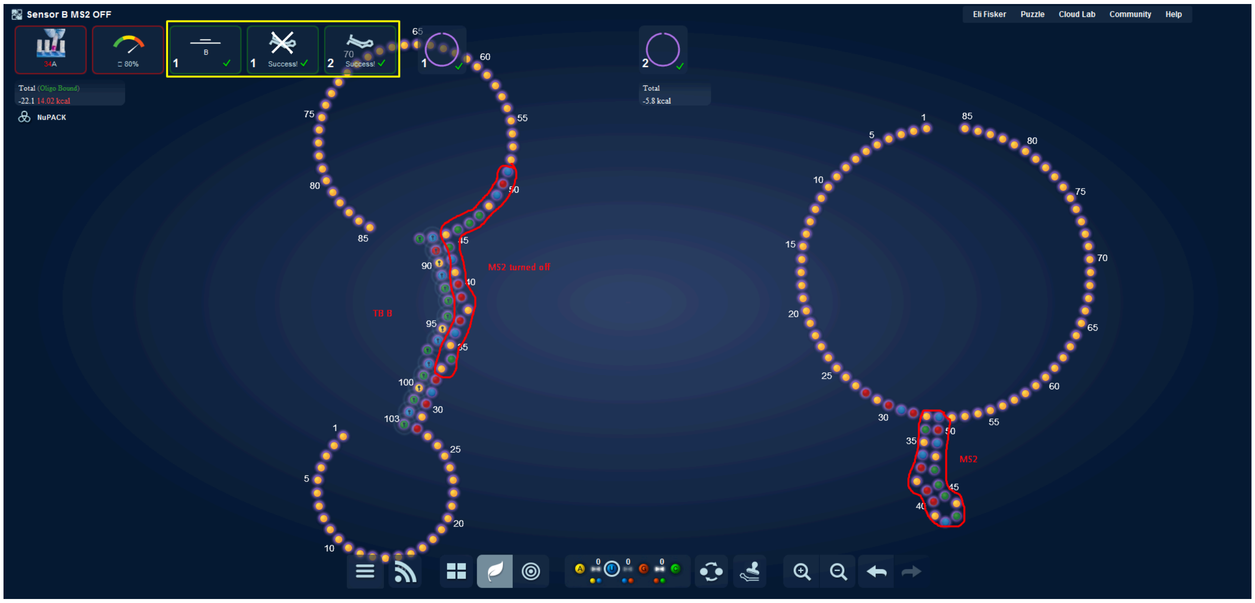

And with ends tied up.

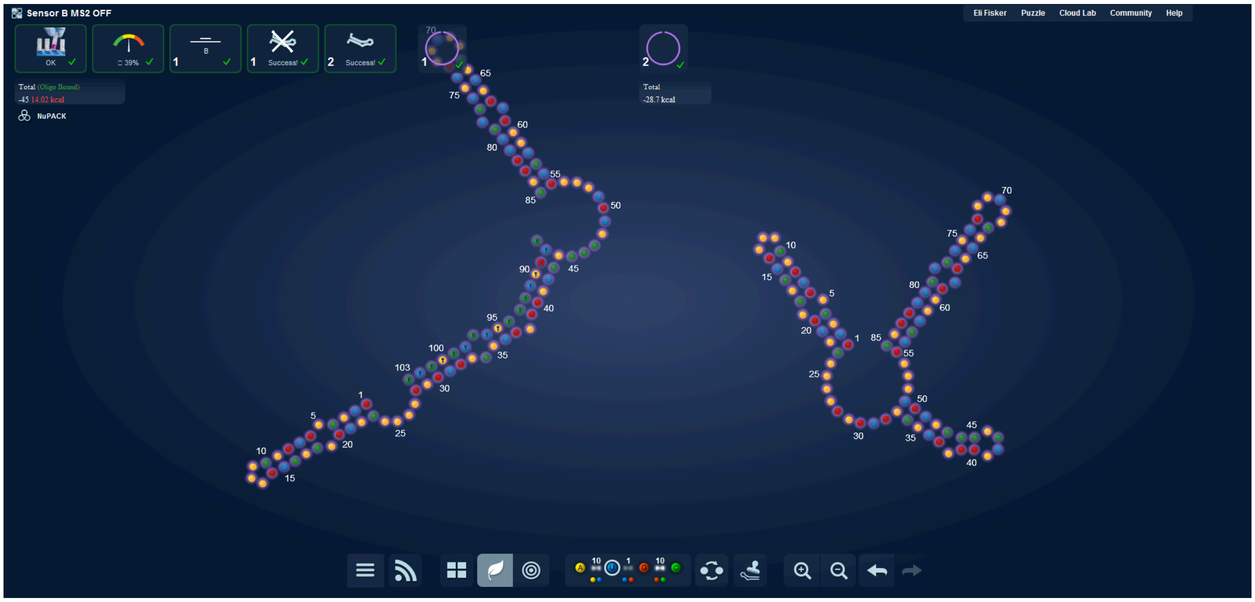

Now this solve isn't necessarily the best. As the microRNA tends for MS2 in turn on labs, to like being after the MS2, instead as here, after the MS2. (Turn on labs - labs where the MS2 gets turned on in the later state or most of the states)

Make complementarity to what you wish to catch

Here is another way to get to a solve, that I have regularly seen work well in a past MicroRNA lab and which is rather simple. I think this way is actually better than what I showed just before. Both as it gets the microRNA complement to the more effective end of the RNA design and after the MS2, but also because it lays out a sticky trap for the microRNA we wish to catch.

It goes like this:

Make a pair up between the end of the RNA sequence to most of the microRNA. Then place MS2 short space before, and put something like 5 bases between MS2 and the microRNA sequence, that should aim for turning off the MS2.

Labs that catches 1 microRNA generally likes to pair with most of the microRNA.

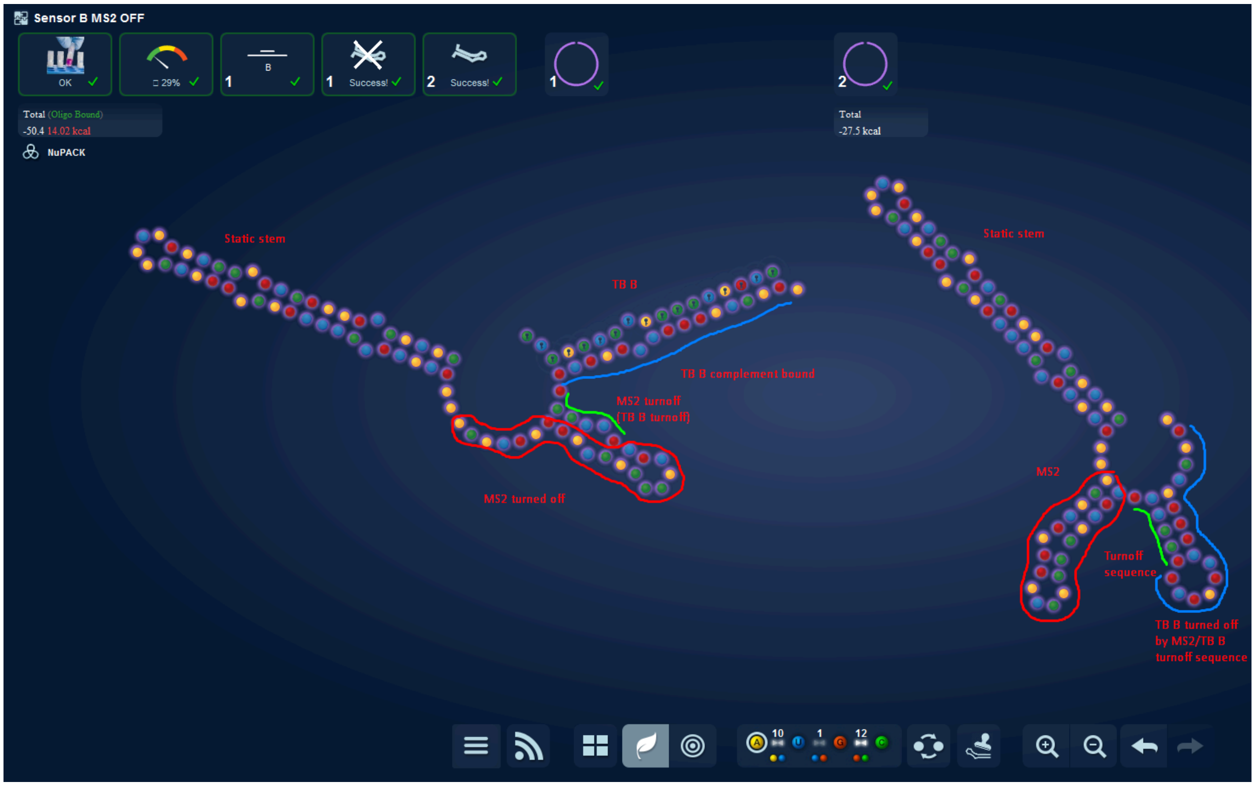

Notice that the MS2 turnoff (GUUCC) does the same as the GGGU kernel attractor that Omei has mentioned earlier. It acts as middle man between the G's in the TB B complement and the G's in the MS2 sequence.

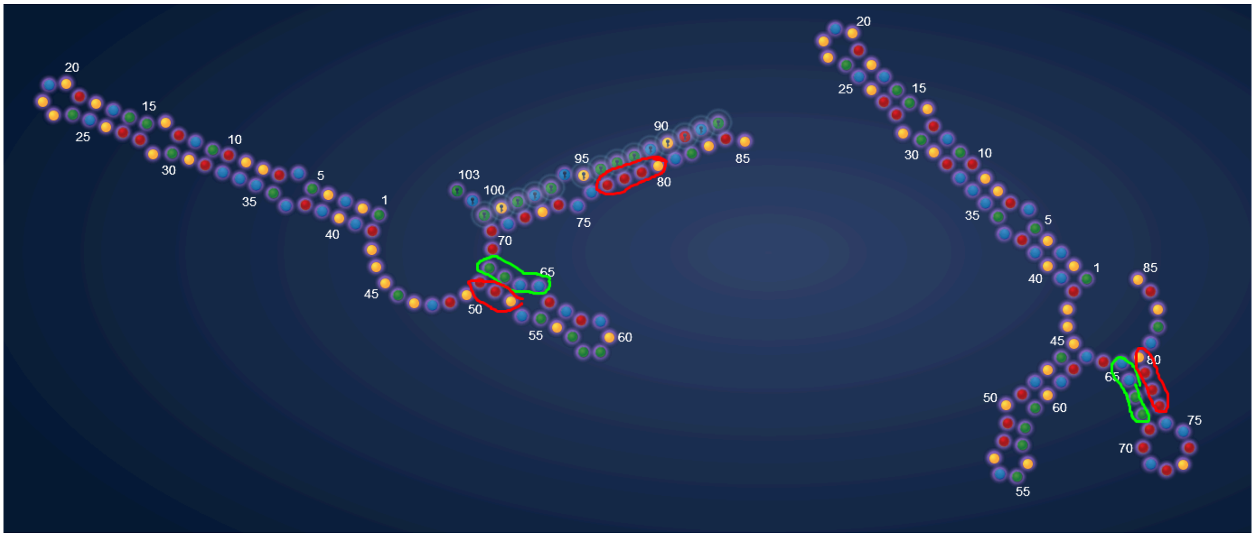

The switch accelerator

Also there is something special worth noticing here. The dangling stretch of bases in each state.

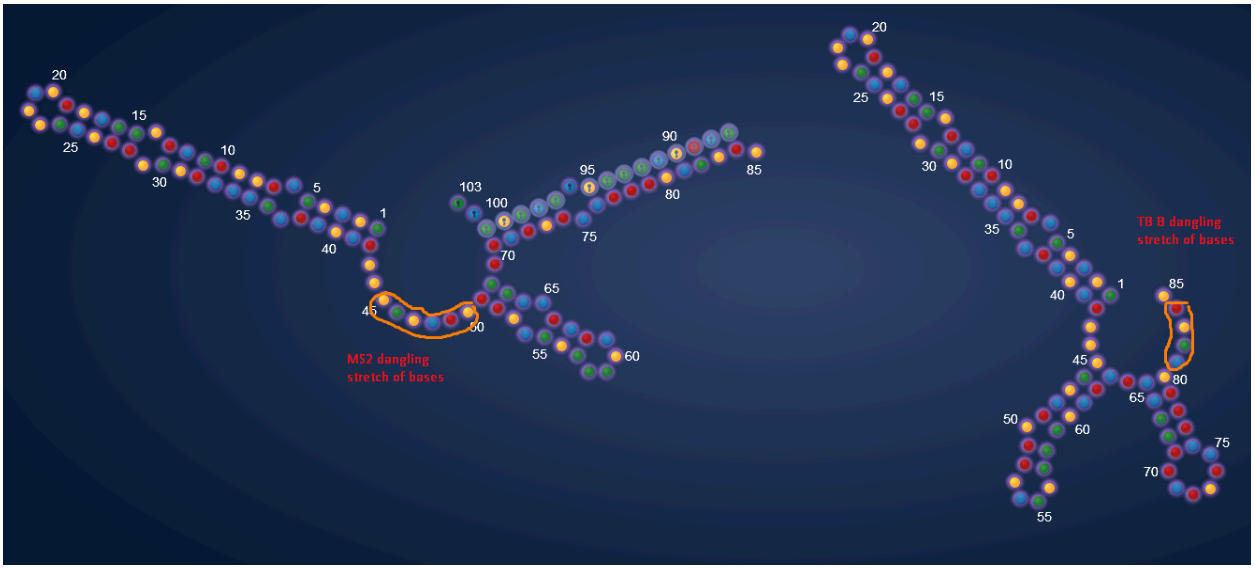

To get a microRNA switch between different states, it has turned out to be very helpful to have a stretch of unpaired bases ready for activating the other state. Something jandersonlee showed in our first microRNA lab. Simply by laying out a complementary stretch of single bases to “trap” the microRNA he wished to catch, he won 2 out of 3 labs in the first go. This strategy showed to work in the nr. 3 lab also.

Also notice that the MS2 also have dangling bases. For now it seems helpful if each state of a microRNA, has a dangling stretch of bases that can help switch the RNA to that state. So this design having a dangling stretch in each state, is good. It should help the RNA get unstuck and be able to move easier between states. In static lab designs such a strategy would be counterproductive and make the design unstable, where we don’t want it to switch.

You can read about it here.
[MicroRNA welcoming tail]
[A landing spot for the MicroRNA]

Just having having any stretch of single unpaired bases won’t help. The bases doing the dangles should be part of the switching elements. Like a MS2 or a TB B or A complement. Actually having too many unpaired bases have proved to be counterproductive, which is why there is a constraint on a minimum number of base pairs for the lab puzzles.

Sensor B MS2 off - Results

For this lab I got my predictions all wrong. Basically this lab needs its design complex put at the 5’ end similar to what was working for the Sensor A MS2 off. I put it at the 3’ end. I have

generally favored the 3’ end since I saw this work really well in our first microRNA labs. However there seems to be other rules at play also.

There is a trend for single input labs, that needs to be turned off, to have their oligo placed at the beginning with the MS2 after and the labs that needs to be turned on, have their oligo placed late, and the MS2 before. Or put simpler like this:

- The late bit in the RNA sequence (3’ end) is best for whatever one wants turned on.
- The early bit in the RNA sequence (5’ end) is best for whatever one wants turned off.

Read more about this at: [The grammar of RNA] and [RNA as Teeter Tooter].

Read more about this labs results, search for [Sensor B MS2 OFF]

Also just as with the other B lab, this lab with strong input, shows less care about where the input is caught. It seems like it is able to handle more different solutions compared to the A labs. However the A lab is pretty easy making happy in comparison, if just the input is put fairly right.

What worked, as demonstrated by this JR puzzle. Notice the TB B complement at beginning of the design.

Score 95%

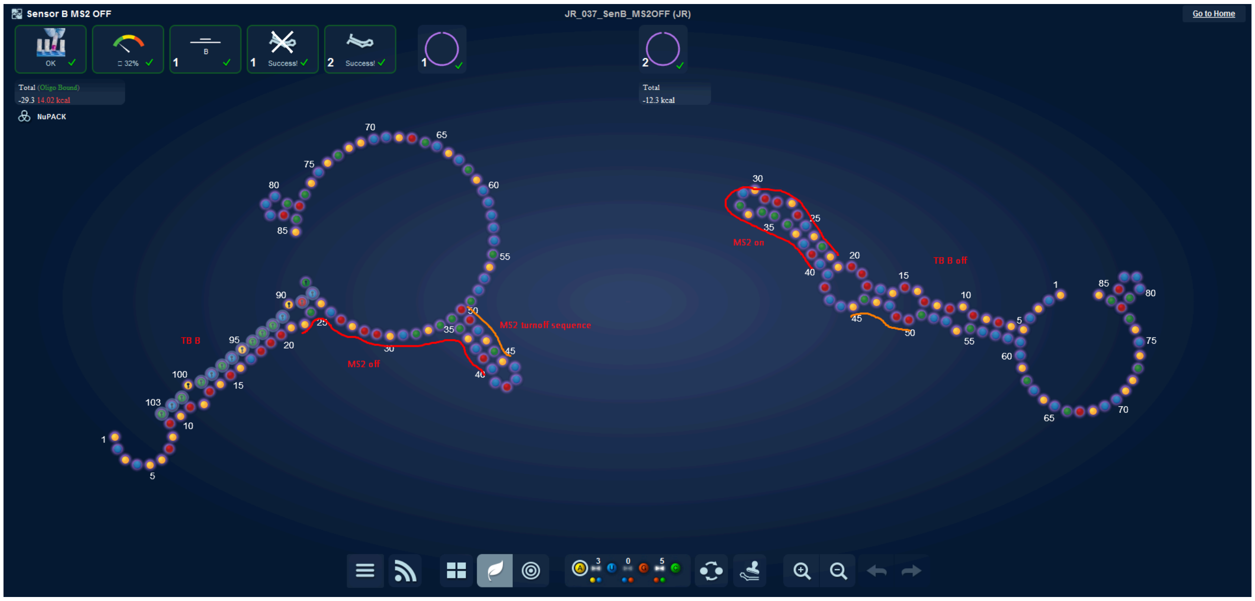

http://www.eternagame.org/game/browse/6296751/?filter1_arg2=6318647&filter1=Id&filter1_arg1=6318647

Similar Mat’s JL mod did well too. This uses an approach of switch element sequence sharing. MS2 shares lane with TB B

94%

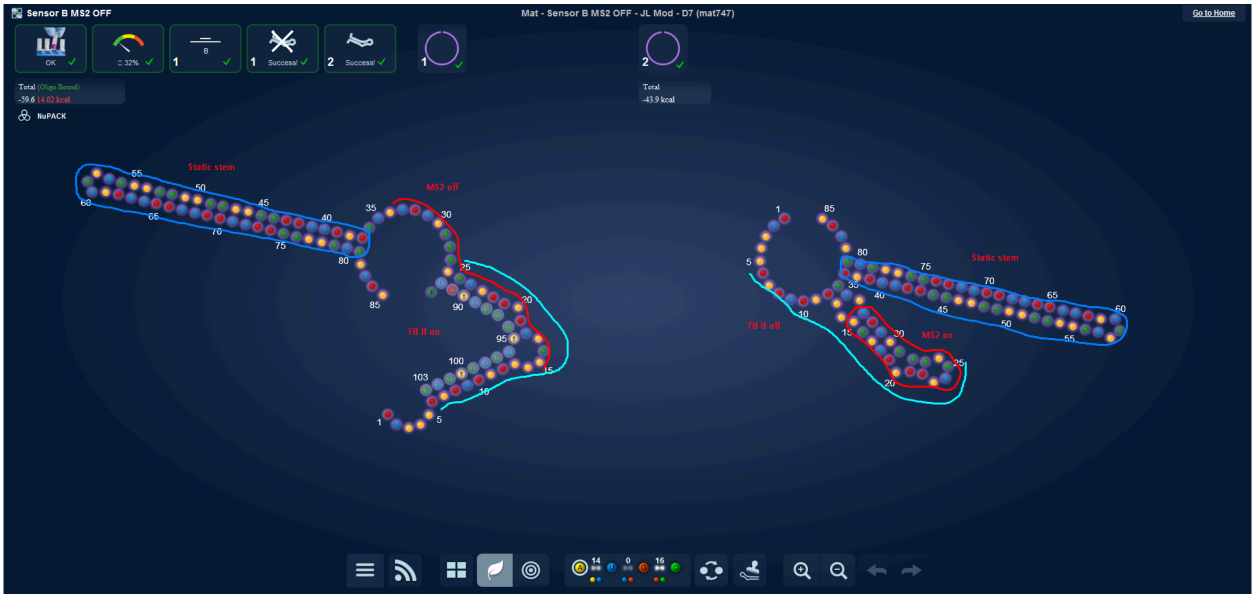

http://www.eternagame.org/game/browse/6296751/?filter1_arg2=6318647&filter1=Id&filter1_arg1=6318647

[A]/[B] with predefined binding sites

I recommend looking at the other players designs for this lab. Since there is not much you can move around to improve the lab solve. I will recommend placing the MS2 somewhere in the middle between the microRNA complements.

Step 1:

Attach one of the microRNA's by making complements to the

I start with TB A as I know from earlier that it is the harder one attaching. I make direct = as strong as possible complementarity and leave in the 4 G's in a row which is forbidden for submission. I deal with them later.

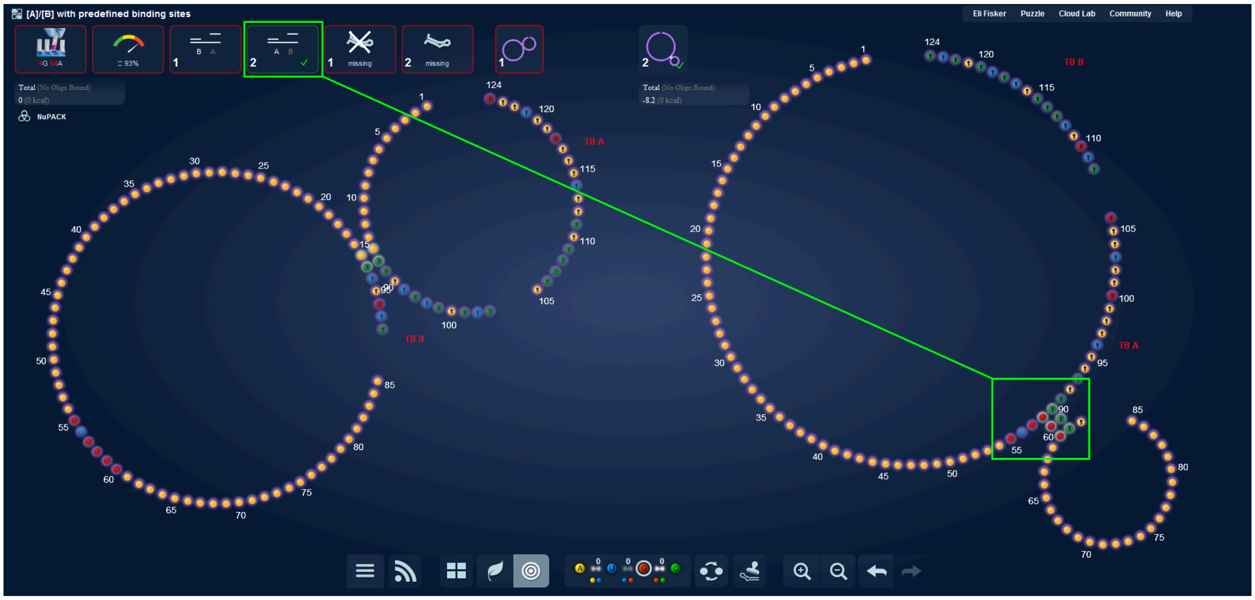

Step 2:

I attach the TB B. Now they are both attached. Notice that the A and B attachment box goes red. This is because they don't take turn for being attached.

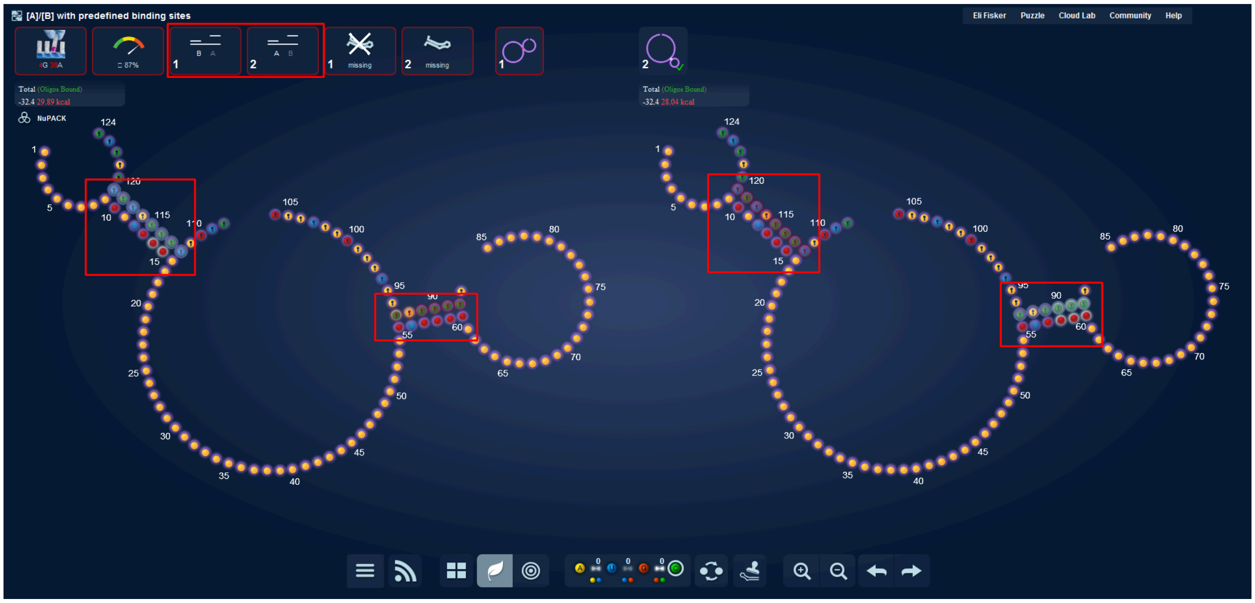

Step 3:

Put in MS2 with the stamper. I put in the MS2 so it is next to the TB B complement, if it was full paired up.

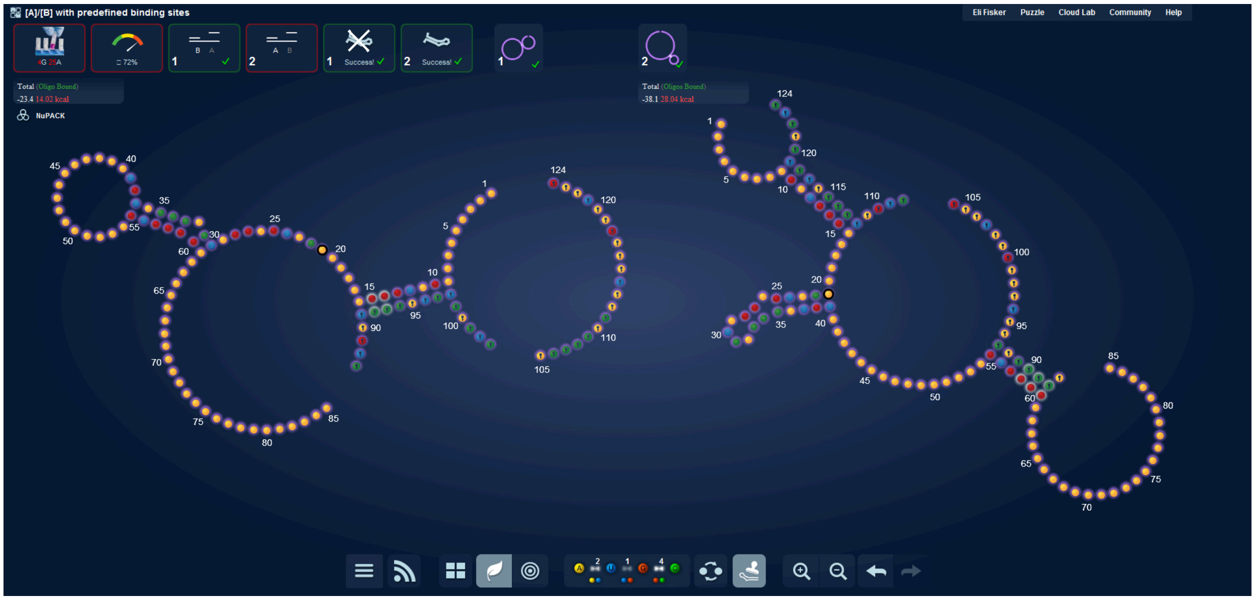

Now is a lot of space between the elements. Generally microRNA likes to be full or almost full paired up with the design. So I put in as many complementary bases as possible. Since the TB B complement is bound up, I start by prolonging that one.

[A]/[B] with predefined binding sites (alternative)

I recommend looking at the other players designs for this lab. Since there is not much you can move around to improve the lab solve.

R2 (2-states model)

Examples of designing for the 2-states model lab. Both are of the kind where the microRNA inputs share attractor in the RNA design. The reason for this choice is that it is easier kicking one RNA out, when part of its attractor is needed for a full bind of the other microRNA.

I have made a drawing with colors and symbols for attraction of the R2 labs for one of the things that I think could work well. I colored C stretches green and gave them a + sign for showing attraction and similar I colored G stretches red and gave them a - to show their attraction.

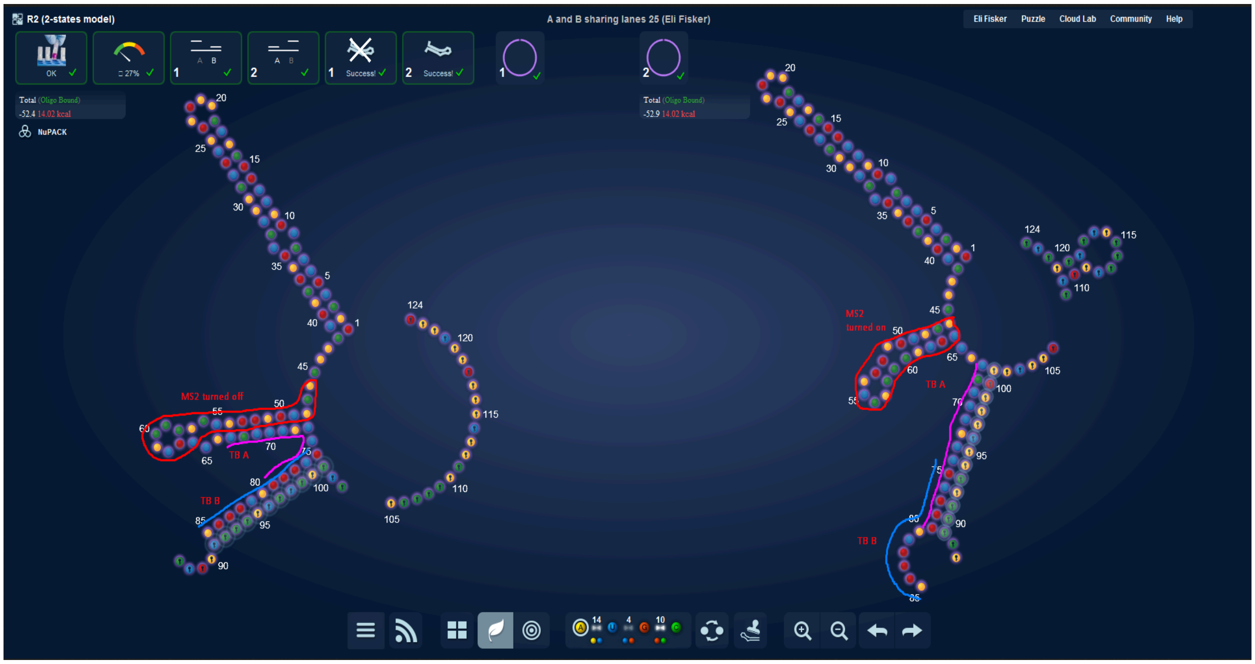

I did this both for the microRNA inputs and for the design. I think this could be useful for showing what a specific microRNA would be inclined to prefer.

Eg if one microRNA has a C stretch and the other microRNA has a G stretch - then placing them right opposite each other on either side of the MS2 is going to be hard, as then they would naturally want to pair with each other and not let the MS2 go. So you can use their nature to say something about what they will prefer and need.

In this lab where TB A needs to kick out TB B, I take advantage of that MS2 and TB A needs to be gone in the same state (1). I asked if they had complementary stretches between them that I could use for turning them both off at once, by making them pair with each other and used the TB A complement to help turn off the MS2.

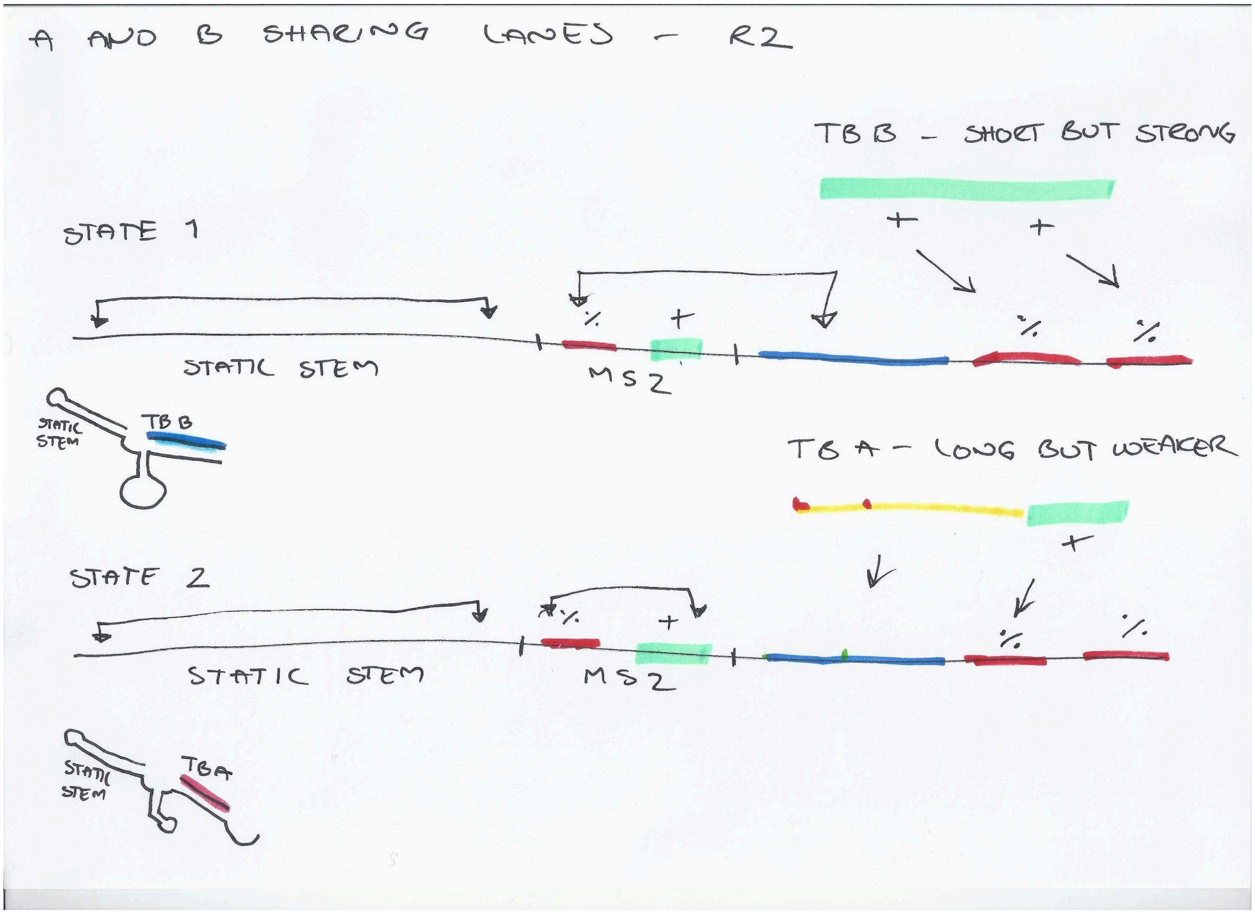
A AND B SHARING LANES - R2
TB B - SHORT BUT STRONG
STATE 1
STATIC STEM
MS2
STATIC STEM
TB B
TB A - LONG BUT WEAKER
STATE 2
STATIC STEM
MS2
STATIC STEM
TBA

Example:

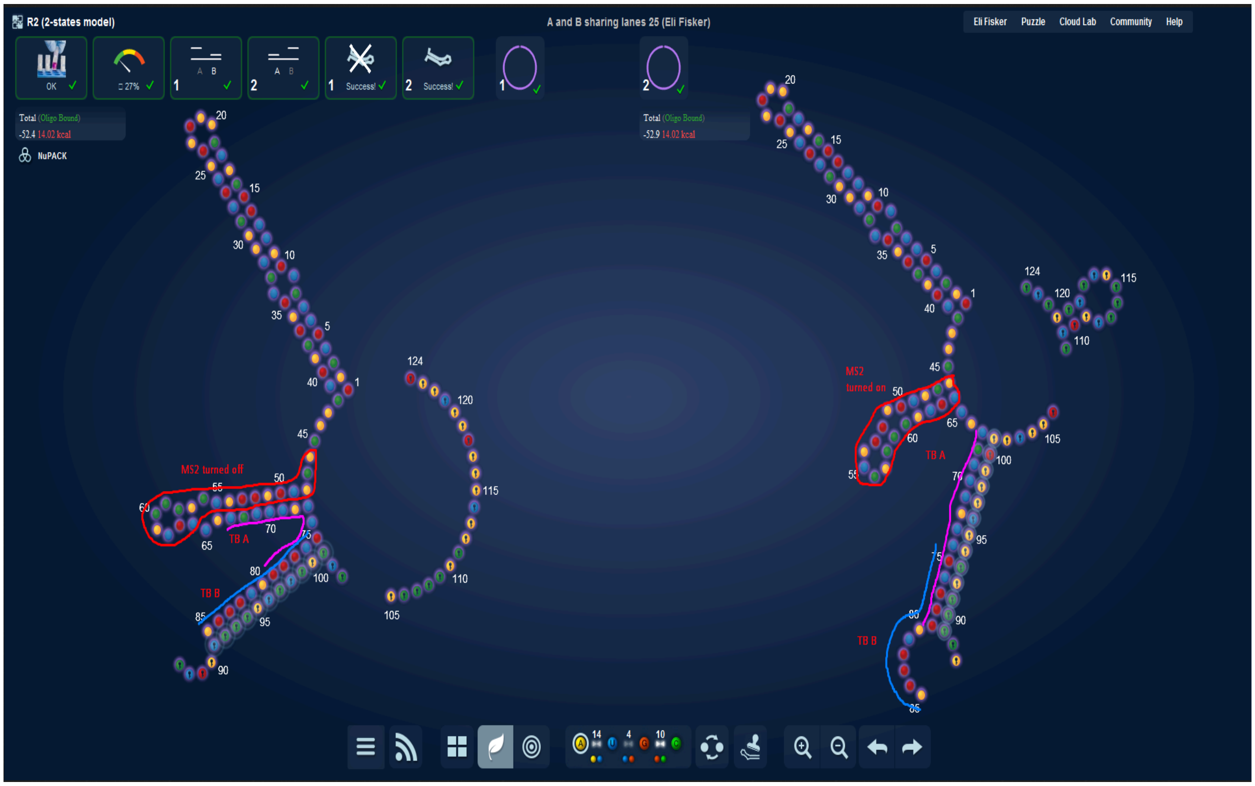

Here is an illustration of another route to get to a solve. I have called this design route B before A, as the TB B complement comes before TB A in in the RNA sequence. Here I take advantage of that MS2 and TB B are directly complementary and use it for MS2 turnoff in state 1, where both needs to be gone.

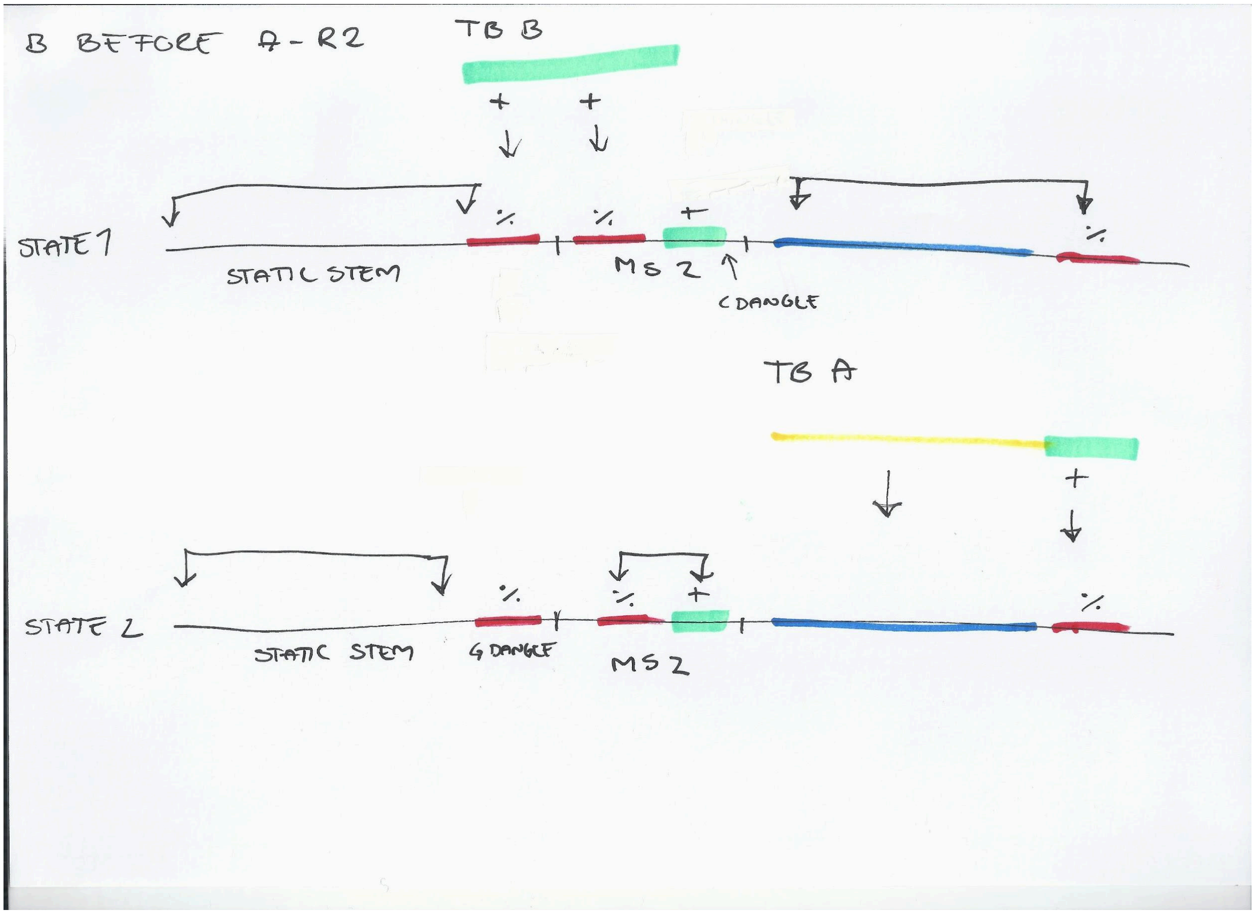
B BEFORE A - R2
TB B
STATE 1
STATIC STEM
MS 2
C DANGLE
TB A
STATE 2
STATIC STEM
G DANGLE
MS 2

Example:

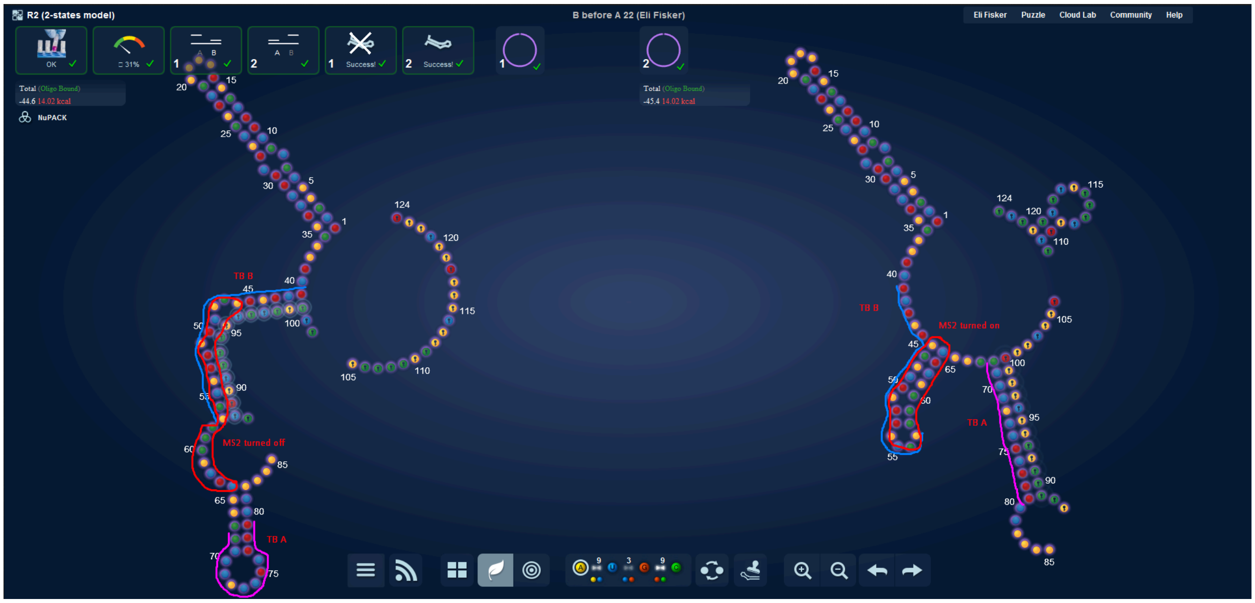
R2 (2-states model)
B before A 22 (Eli Fisker)
Eli Fisker
Puzzle
Cloud Lab
Community
Help
TB B
MS2 turned off
TB A
MS2 turned on
NuPACK

Notice that I in both illustrations have put the static stem first in the RNA sequence. Our earlier microRNA lab data with 1 microRNA input has shown that the microRNA mainly prefer to land in the late end of the RNA sequence (3') in turnon labs, which R2 is.

Turnon and turnoff is taking the perspective of the MS2. Here MS2 is needed to get turn on in the later state = hence turnon lab.

Static stem in the switching area

I have decided that I do believe that a static stem can have a function in a microRNA lab, despite my earlier dismissal of the static stem having a function in single input microRNA puzzles. Especially I think static stems start to play a bigger role in microRNA's with multiple inputs. I think it has an additional function.

I think it could be worth a shot putting in a static stem in the switching area, despite we have no data on this yet. But then on the other hand we don't have any data yet on two microRNA input labs either.

Static stems and the microRNA labs

R2 - Results

For designs with two inputs and that are not using the full space of the RNA sequence - like mine above - this round showed to prefer a early positioning of the design complex (5') where I had put it late (3') as this was what I have earlier seen single input labs prefer. However this has now seemed to change. Both R2 and R3 seems to prefer having their design complex early at 5' end over 3' end. So my first recommendations didn't work. Instead I have slided some of my designs to the other end of the RNA sequence to see if I can get them work this round.

I ended up having most luck with the Static stem in the switching area strategy, that I earlier described here. I have commented on the results in that same post.

R3 (3-states model)

It is also possible solving the R3 lab with a static stem in the switching area just as I mentioned for the R2 lab just above.

Here is an example that I judge have a shot. TB B should be present in all states and TB A only in two. I think that it is so strong that it may have a chance to bind up to half its own complement in stat 1 and 2. And in state 3 it should win out since it is higher in concentration. Again, I can't know if it works, but I think this style with a static stem in the switching area is worth a shot.

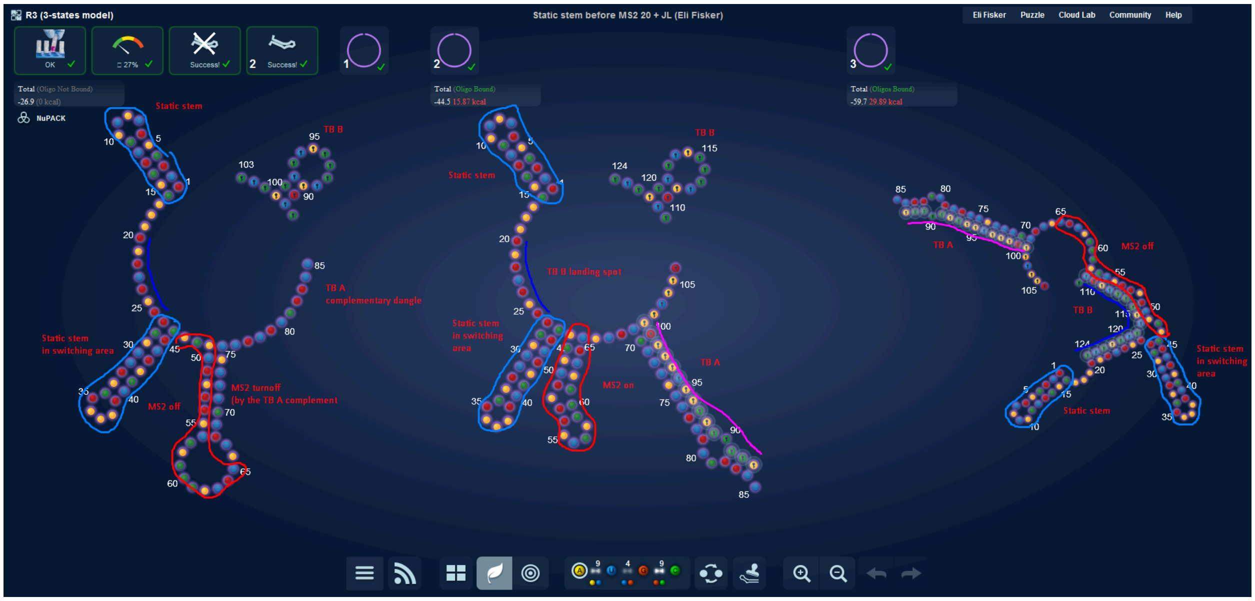

I have put up a bunch of designs, where I do some description. You can see Omei's lab solves here, my lab solves are here. Not all of mine are Kosher. Those named experiment and unstable are experiments. I point towards what I think could work, but I'm learning just as you. If there are a lot of one kind, it is usually one type I believe in.

R3 - Results

I put up my analysis of the R3 lab in the forum.

For more lab advice for the [A]/[B] labs, see Doing Math with RNA.

Save at least the diagrams for re-use later:

… or add a second one in series, like this:

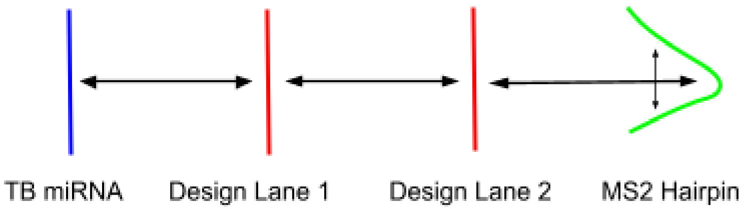

To verify that this attraction pattern does what we want for this lab, consider the two cases - TB-A is either present or not. If TB-A is not present, the attraction between the two design lines and the internal attraction of the MS2 hairpin can overpower the the attraction between attractor 2 and the hairpin

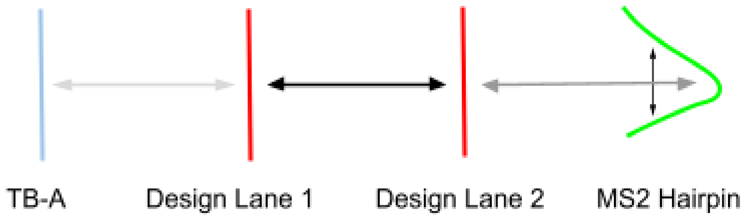

But if TB-A is present, the attraction between TB-A and attractor 1, plus that between attractor 2 and the MS2 hairpin will overpower the other two.

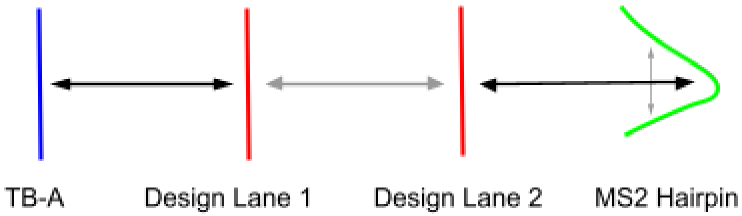

~~This version is a little less flexible, because it can be hard to extend the kernel attraction into a strong attractor.~~
~~For this to actually work requires a special condition -- the input miRNA must complement the MS2 hairpin so well that the binding between them as stronger than the internal binding of the MS2 Hairpin. But somewhat surprisingly, TB-A does in fact do that. So we can use the second option here.~~

~~But keep in mind that this strong attraction between an miRNA and the MS2 hairpin is an exception, not the rule. For the Sensor B MS2 OFF lab, we'll need to use the attraction pattern with two attractors.~~

Logic Gates Design Refinement Strategy

Jeff Anderson-Lee, Eli Fisker
Dec 2022

Abstract

The Eterna on-line citizen-science game held two lab rounds aimed at designing 2-input logic function sensors for RNA input oligos using a fluorescent MS2 molecule for output signaling. Players were tasked with designing an RNA strand that would "switch" its shape depending on external conditions. The game was modified to use the NuPACK algorithm to model the experimental conditions and show players the MFE shape for each state. The first lab round put forward three sub-labs that represented OR, AND, and XOR conditions of two input molecules. The second round added five additional logic functions (A AND NOT B, A OR NOT B, NAND, NOR, and XNOR). Three Eterna players used an off-line NuPACK analysis to evaluate and select designs for the second round. The pairing-probability of the closing base-pair of the MS2 aptamer was used to include some ensemble effects in the analysis. The sequences from the first lab round were used to estimate constraints for selecting potentially better performing designs. Selected sequences were then "mutated" and re-analyzed to create submissions for the second lab round, including for logic functions not present in the first lab. This strategy created winning sensors in all eight sub-labs in the second lab-round. In 75% of the sub-labs (all except AND and NAND) this strategy produced a top-ranked design (i.e. as good as or better than a player-generated design); in half of the sub-labs, it produced a design that scored 100.

Introduction

Rounds R98: The Real Logic Challenge using NuPACK, and R102: Logic Gates of the on-line citizen-science game Eterna (eternagame.org) [1], were two rounds aimed at designing RNA sensor molecules that could detect the presence or absence of two "input RNAs" and signal the result of a two-input logical boolean operation by interacting with the inputs in a way that changed the the ability of the sensor to bind with a fluorescent MS2 signaling molecule [2]. The use of the MS2 signaling molecule built upon the prior experience of Eterna players in designing single input sensor labs that used the same MS2 signaling molecule.

This document describes one of the design refinement strategies used by three Eterna players Eli Fisker, jandersonlee, and mat747 in designing winning sensors for round R102. This strategy assumed that there was an existing sensor design that was expected to perform somewhat in the manner desired but which required some fine-tuning via "mutations" to produce a more optimally performing sensor. This document does not cover the means by which the original designs were made.

Note that it is a common strategy in Eterna to take a design made in an earlier round or earlier in the same round and "modify" (or "mod") it in an attempt to improve it. In the simplest case these modifications may be substitutions of a single base or base-pair which is sometimes termed a "mutation". More complex design alteration strategies often include shifting portions of the design towards the 5' or 3' end of the sequence or bundling unused portions of a design sequence into a "static stem" that does not change from one state to another; these sorts of modifications were not used in the mutation/evaluation design refinement strategy described here but were used successfully by many players in some of these sub-labs.

Basic Switch Design

In the parlance of Eterna, an RNA sensor molecule that folds differently dependent on external conditions is called a *switch*. The type of switches designed in these labs used an MS2 protein as a signaling molecule (aka *reporter* or *R*). A region of the sensor strand holds an RNA subsequence that can form a target aptamer binding site for the MS2 protein. If the sensor molecule forms a particular shaped aptamer region (Figure 1a), the MS2 molecule can bind and the resulting combination will fluoresce under the appropriate illumination. However, when the aptamer region is partially bound up by other regions of the sensor RNA (figure 1b) or with other input oligos, it does not form the target aptamer shape and the fluorescent MS2 reporter will not bind to the sensor. When the RNA sensor molecule folds so as to form the aptamer region such that the reporter binds, the sensor is said to be in an *ON* state. When the aptamer shape does not form, the sensor is said to be in an *OFF* state. The task of switch design is therefore to create an RNA molecule that will be ON or OFF (forming the aptamer region or not) by folding differently under the various target states or conditions.

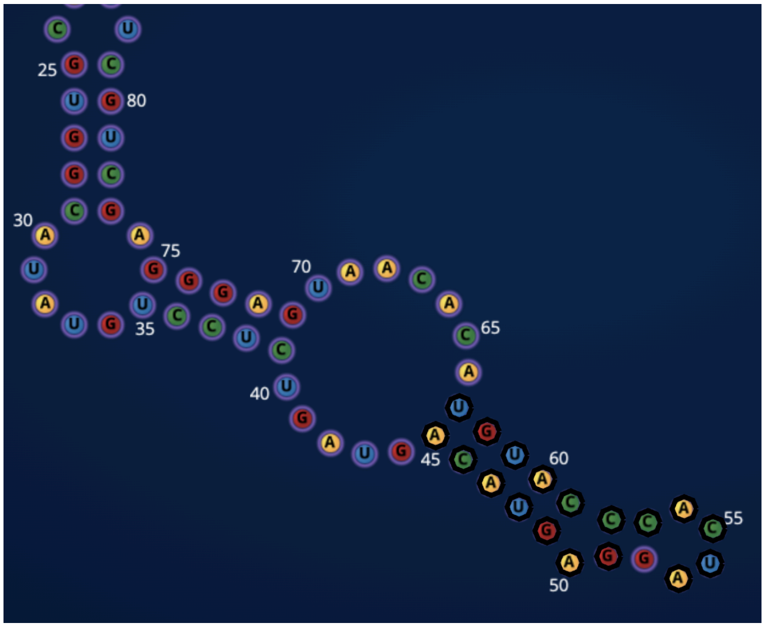

Figure 1a. An MS2 target aptamer site (bases 45 to 63) folded for MS2 receptivity

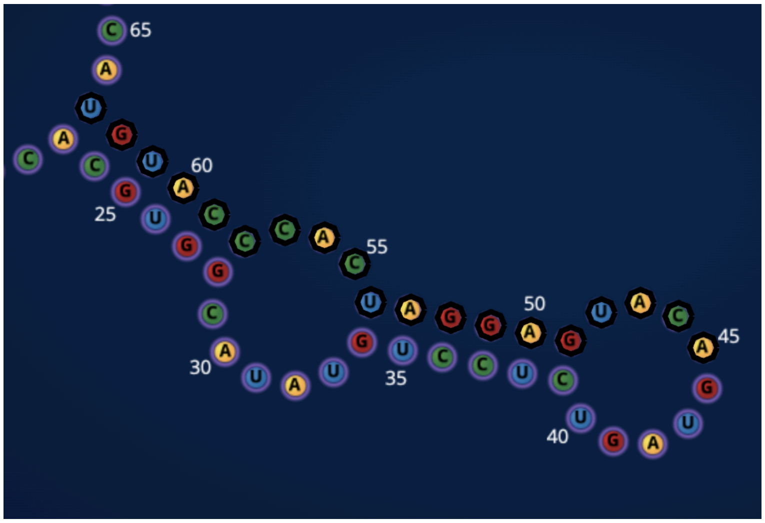

Figure 1b. An MS2 binding site (bases 45 to 63) blocked from forming the target aptamer shape

Each sensor is designed to have additional target regions that have some affinity with portions of the input oligos, such that when an input oligo is present in a sufficient concentration it will tend to bind with that region of the sensor molecule. For input oglio A, the target binding region is sometimes referred to as *A'*. This in turn prevents that region (or nearby regions) of the sensor from binding with other regions of itself (sometimes called *A"*). Similarly, the MS2 reporter molecule (*R*) has an target aptamer region sometimes referred to as *R'*, and the area(s) it binds to when OFF can be referenced as *R"*. Sometimes some of these regions overlap. By carefully arranging these regions and balancing their affinities with the input oligos and other regions of the sensor it is possible to design sensor molecules that only form the MS2 signaling aptamer region in certain desired states.

The exact techniques used to design and arrange these various regions to form the sensor molecule are beyond the scope of this document. What is important however is that having designed a rough plan for the layout of the sensor we may need to estimate its performance and fine tune its action by giving certain regions and oligos stronger or weaker affinities with each other.

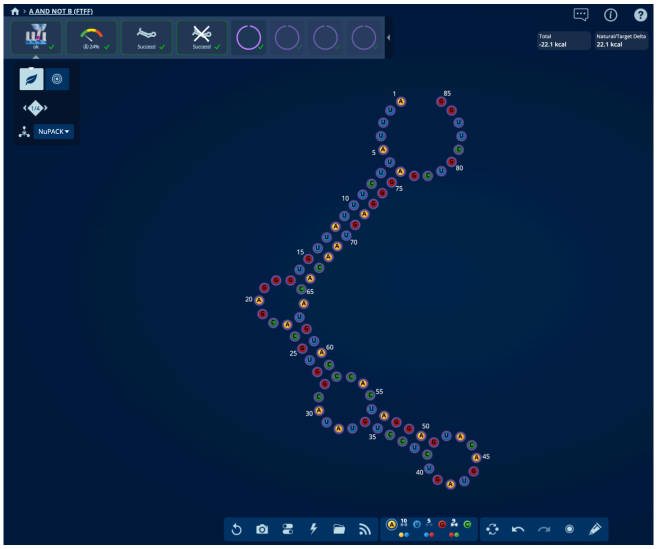

Fig 2a: Design [6492827](6492827) State 1 - No input oligos

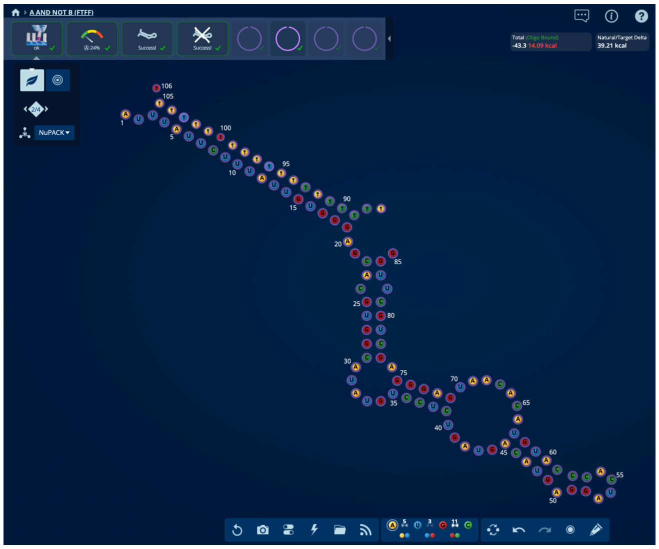

Fig 2b: Design 6492827 State 2 - oligo A

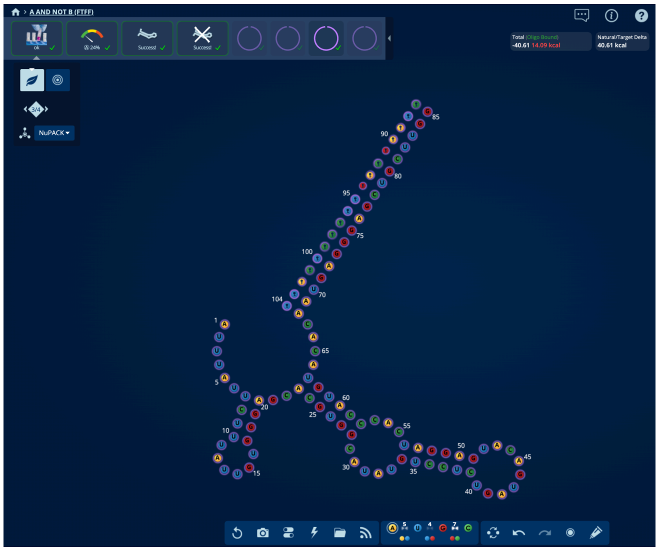

Fig 2c: Design 6492827 State 3 - oligo B

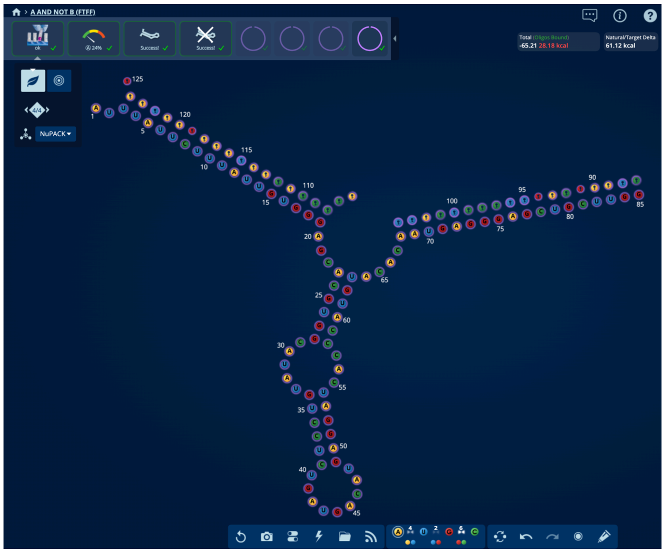

Fig 2d: Design 6492827 State 4 - Oligos A and B

Figure 2 shows four states of a successful sensor molecule designed to perform the logic function "A AND NOT B" also known as "FTFF" since it is only ON (or True) in state 2 when oligo A is present at

100 nm concentration and oligo B is not present. In the three OFF states, base 23 to 63 tend to form a structure with two helical stacks, an internal loop and a hairpin that occupy many of the bases (45-63) that would otherwise form the MS2 aptamer shape. In the ON state (state 2), some of the bases from the oligo B target region (bases 68-85) pair with bases in the range 22-38 that would otherwise normally pair with bases from the MS2 aptamer range (*R'*) to block the formation of the aptamer shape. This leads to the sensor being ON only in state 2.

NuPACK and State Modeling

As well as folding into a lowest energy configuration (Minimum Free Energy or *MFE*), an RNA strand can fold into a variety of similar energy level shapes with lower probability. The collection of these shapes is called the *ensemble*. As an RNA strand folds it takes on one of the ensemble of shapes, each forming with differing probabilities. Over time a given strand may fold and refold holding each shape for some possibly brief interval of time and at any time have a certain probability of being folded into any given shape. However given enough of the same molecules we can estimate the probability that a given fraction of the molecules will have a certain shape or in this instance that the target aptamer shape will form or not form.

As a result, an RNA sensor is rarely said to be 100% ON or OFF. That is, while each individual strand may be ON or OFF, given a cluster of similar RNA strands, some fraction of them may be folded into ensemble shapes that include the MS2 aptamer while others are folded into shapes that do not.

An algorithm such as NuPACK [3] can compute the modeled ensemble for a given set of conditions involving various concentrations of multiple RNA oligos, and in fact Eterna was modified to use NuPACK to do just that for these labs. However the Eterna game interface only shows players a single most probable shape (the MFE shape) from the ensemble for each state.

The NuPACK algorithm, like most RNA folding schemes, tries to predict the free energy of the various most probable shapes in the ensemble. The ensemble itself is combinatorially huge but many of the possible shapes are so unlikely that eliminating them from consideration makes little difference in the result. From the combination of the shape predictions and their free energy predictions, NuPACK is able to estimate the probabilities of any given shape appearing in the ensemble as well as the probability of any given base-pair bond forming (the pairing-probability) for the ensemble. However the predictions of the free energies and pairing-probabilities are only estimates based on modeling of various base-base interactions and other considerations.

While the model is accurate enough to assist in RNA folding prediction and hence RNA sensor design is not a 100% match to nature, so ultimately the RNA sequences must be tested in the lab to confirm how they fold. This is one of the key strong-points of Eterna – it combines citizen-science powered in-silico aided RNA sequence design with in-vitro laboratory testing.

R98: The Real Logic Challenge using NuPACK

The R98 Eterna lab round was the first to use two RNA oligo input sequences taken from tuberculosis signature molecules [4] for use in a two-input, four-state RNA sensor design challenge. This lab round put forward three sub-labs that represented OR, AND, and XOR conditions of two input molecules

dubbed “A” and “B” (or alternatively “oligo1" and “oligo2”)[1]. In each case the sensor molecules were both modeled in-silico and tested in-vitro in 4 different states/conditions representing different concentrations of the input molecules: (state 1 aka FF) with no input oligos, (state 2 aka TF) with 100 nm of oligo A, (state 3 aka FT) with 100 nm of oligo B, and (state 4 aka TT) with 100 nm each of oligo A and oligo B. The Eterna game used the NuPACK [3] algorithms to model the folding and interaction of the RNA molecules at the same concentrations and conditions that were later tested in the lab.

For the AND sub-lab, a sensor was to be designed that would bind to the fluorescent MS2 reporter only when both input oligos were present at 100 nm concentration. For the OR sub-lab, a sensor was to be designed that would bind to the fluorescent MS2 reporter only when either or both input oligos were present at 100 nm concentration. For the XOR sub-lab, a sensor was to be designed that would bind to the fluorescent MS2 reporter only when one or the other of the input oligos were present at 100 nm concentration, but not when neither or both input oligos were present.

R98 Scoring and Metrics

The general outline for scoring of single-state Eterna Switch labs is laid out in a document entitled “Scoring of riboswitches in EteRNA” by Johan Andreasson. An additional forum thread on “Switch Scored for EteRNA Switch Puzzles” further outlines the scoring. One essential component of the scoring is “the dissociation constant, Kd, is the concentration [of MS2] where half of the RNA binds MS2” as measured in the lab.

For a one-input ON switch lab the switch_score or *fold change* portion of the overall score is based on the ratio of KDoff/KDon since the OFF state should require higher concentrations of MS2 to fluoresce. With two input labs the fold change is measured as the min(KDoff)/max(KDon) where min(KDoff) is the minimum Kd for all OFF states and max(KDon) is the maximum Kd for all ON states. For these labs a fold change of 75 or more would yield a “perfect” switch score of 40.

Three Eterna players chose to try a strategy of examining round R98 submissions using an off-line analysis looking for somewhat better performing or promising starter sequences and trying various mutations to see if they could improve the functionality of the designs. For the player’s off-line design purposes it was important to have some means to evaluate a given RNA sequence. Accurate modeling of the KDon and KDoff was presumed to be too complex, so an alternative metric was chosen.

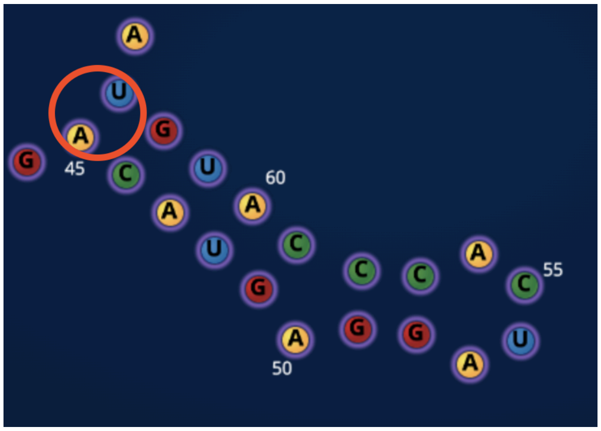

Figure 3: Closing Pair of an MS2 Aptamer

[1] R102 later added five additional 2-input logic challenges for a total of eight.

In order for a sensor to fluoresce, a significant fraction of the ensemble must form the MS2 aptamer site. The MS2 aptamer includes a stem with a bulge and a harpin end. When the full stem forms it must include the pairing of the closing base-pair (Figure 3) for the stem. The ensemble pairing-probabilities data can be computed by NuPACK. The pairing-probability estimation of the aptamer closing-pair was chosen as a proxy for the probability of the MS2 aptamer being formed within the ensemble.

Round R98 RNA design sequences were analyzed using off-line NuPACK evaluation to determine the probability of the MS2 aptamer site forming in the four different states. The sequences and results from the R98 lab round were examined in comparison to the modeled pairing-probabilities to try to determine constraints on the pairing-probabilities that might be indicative of a more successful design.

R98 FFFT (AND) Ranking and Analysis

This section covers the analysis of the R98 AND designs. It referenced the spreadsheet R98-FFFT-Ranking -- a spreadsheet that lists the NuPACK expected probabilities for MS2 arm formation at the experimental titrations. It also references the R98 AND Results, a Google Sheet with the results for the R98 AND sub-lab.

The Google Sheet R98-FFFT-Ranking holds the results of a NuPACK analysis of the 286 AND sub-lab designs from R98 that had an Eterna Score of better than 60.0 which means they had at least some tendency to switch. In the following, FF=State 1 (no additional oligos), TF=State 2 (oligo A at 100 nm), FT=State 3 (oligo B at 100 nm), TT=State 4 (both oligo A and B at 100 nm).

The column labeled FF holds a proxy for the expected probability (in the NuPACK model) of the MS2 arm forming absent both oligoA and oligoB (FF: A=False, B=False); it appeared that this should be a small value (e.g. <0.05 or 5%) for a design to work well as an “AND” detector.

The column labeled TF holds a proxy for the expected probability (in the NuPACK model) of the MS2 arm forming with oligoA at 100nM and oligoB at 0nM (TF: A=True, B=False); it appeared that this should also be a small value (e.g. <0.05 or 5%) for a design to work well as an “AND” detector.

The column labeled FT holds a proxy for the expected probability (in the NuPACK model) of the MS2 arm forming with oligoA at 0nM and oligoB at 100nM (FT: A=False, B=True); it appeared that this should also be a small value (e.g. <0.05 or 5%) for a design to work well as an “AND” detector.

The column labeled TT holds a proxy for the expected probability (in the NuPACK model) of the MS2 arm forming with oligoA at 100nM and oligoB at 100nM (TT: A=True, B=True); it appeared that this should be a large value (>0.8 or 80%) for a design to work well as an “AND” detector.

The column labeled “rating” holds a design rating value derived as follows:

rating = sqrt(TT) * TT / max(FF, FT, TF)

The expectation was that the bioluminescence signal would be roughly proportional to the percentage of sequences that fold with the MS2 arm in place in each experimental condition. It was expected that a design would do better as an AND detector if the ON signal (TT, for A=True and B=True) was much stronger than the brightest OFF signal (the max of max(FF, FT, TF)) at the same concentration. The sqrt(TT) factor was to give some extra preference for designs with a brighter ON signal.

The columns ms2start and ms2end hold the base numbers of the start and end of the subsequence that represents the MS2 arm to use in estimating the closing-pair pairing-probabilities. The estimated probability of the ms2start:ms2end pair bond forming in the NuPACK energy model (at the experimental molar concentrations) was used as the proxy for the expected probability of formation of the MS2 arm.

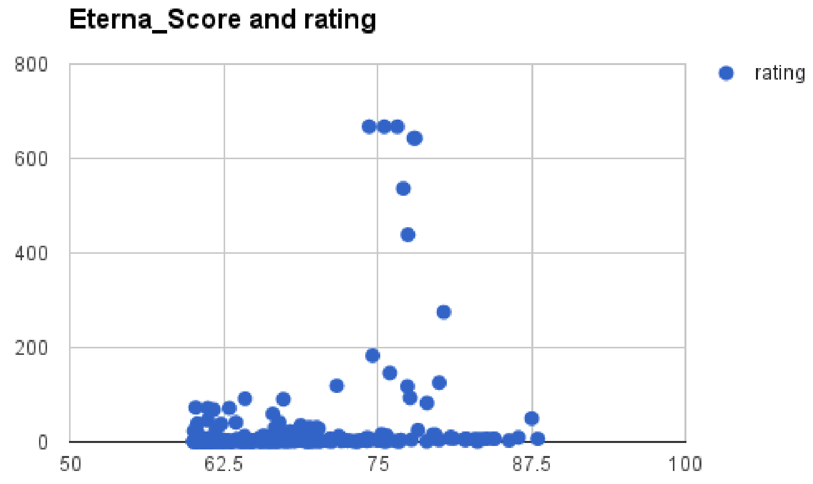

Figure 4: Eterna Score versus Rating for R98 AND Sub-lab

Looking at the rating defined above in relation to the measured Eterna Score in the R98 lab results, there was not any sort of linear relationship of high rating to high score. In fact, the highest scoring designs (with one exception) tended to have ratings of under 11 and go as low as 1.14. (See Figure 5)

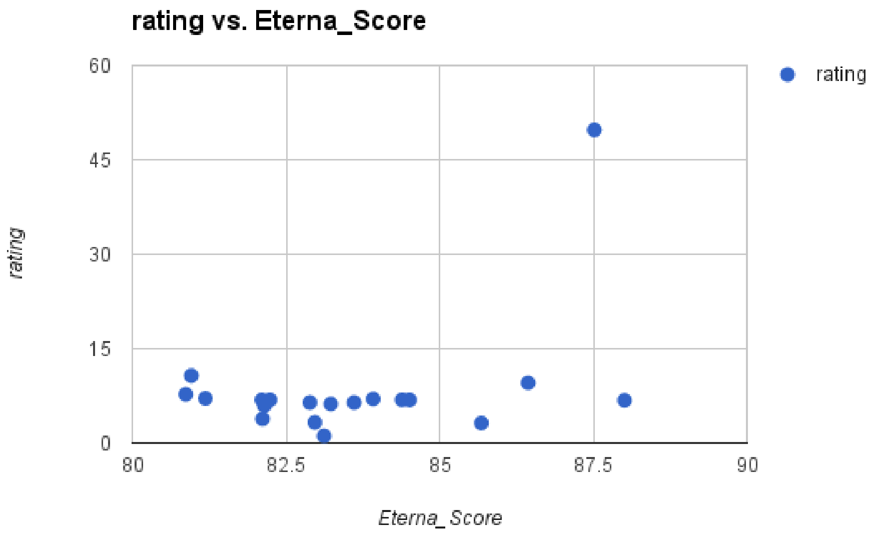

Figure 5: Eterna Score versus Rating for High Scoring R98 AND Sub-lab Designs

id	Eterna_Score	rating	FF	TF	FT	TT	ms2start	ms2end
6115791	88.01	6.79	0.0000	0.0037	0.1210	0.8775	34	52
6122197	87.52	49.75	0.0015	0.0085	0.0042	0.5613	33	51
6145586	86.44	9.57	0.0000	0.0697	0.0092	0.7634	33	51
6118446	85.68	3.17	0.0296	0.0637	0.2822	0.9287	31	49
6154110	84.51	6.86	0.0542	0.1190	0.0528	0.8740	34	52
6154179	84.39	6.87	0.0528	0.1189	0.0448	0.8740	34	52
6154229	83.92	7.01	0.1166	0.0953	0.0448	0.8740	34	52
6154170	83.61	6.44	0.0567	0.1268	0.0348	0.8740	34	52
6154183	83.23	6.22	0.0595	0.1314	0.0448	0.8740	34	52
6106428	83.12	1.14	0.0011	0.2480	0.0012	0.4308	30	48
6104390	82.97	3.28	0.1226	0.1386	0.2523	0.8822	34	52
6154172	82.89	6.44	0.0518	0.1269	0.0528	0.8740	34	52
6104396	82.24	6.87	0.0585	0.1189	0.0348	0.8740	34	52
6154221	82.15	5.97	0.0614	0.1368	0.0448	0.8740	34	52
6118440	82.12	3.84	0.0319	0.0815	0.2326	0.9278	31	49
6154177	82.11	6.86	0.0669	0.1191	0.0341	0.8740	34	52
6154181	81.19	7.09	0.0508	0.1153	0.0448	0.8740	34	52
6145574	80.96	10.71	0.0000	0.0545	0.0142	0.6988	33	51
6154227	80.87	7.74	0.1056	0.0940	0.0448	0.8740	34	52
6115796	80.38	274.97	0.0000	0.0000	0.0017	0.5939	34	52
6115804	80.01	125.43	0.0000	0.0000	0.0054	0.7711	34	52
6118444	80.00	3.99	0.0382	0.1327	0.2229	0.9256	31	49
6152978	79.69	15.55	0.0000	0.0035	0.0528	0.8768	31	49
6154225	79.68	7.57	0.0470	0.1080	0.0448	0.8740	34	52
6152982	79.50	15.53	0.0000	0.0012	0.0456	0.7946	31	49
6115809	79.02	82.71	0.0000	0.0000	0.0101	0.8884	34	52
6118498	79.00	1.21	0.0094	0.5221	0.0085	0.7375	30	48
6105985	78.28	25.07	0.0000	0.0182	0.0190	0.6106	30	48
6116005	78.07	643.06	0.0000	0.0000	0.0015	0.9635	34	52
6116007	77.94	643.06	0.0000	0.0000	0.0015	0.9635	34	52

Table 1: MS2 Arm Probability Predictions for High Scoring R98 AND Designs

Table 1 lists the detailed NuPACK probability estimates for the MS2 arm formation under the various experimental conditions for most of the top scoring designs. Highlighted here are some of the areas that may be problematic for each design. If an OFF state has probability 5%<p<=10% or an ON state has probability 60%<p<=80% it is highlighted in yellow. If an OFF state is probability >10% or an ON state has probability <=60% it is highlighted in pink. If an OFF state has probability <0.01% it is highlighted in purple. Green is thought to be good: 0.1%<OFF<5% and 80%<ON.

Beginning at the bottom of Table 1, there are some designs (6116005 Figure 6, and 6116007) with very high ratings (643.06 and 643.06) that score less than 80 in the in-vitro lab. They both have a very high ON response (96%) but also a very strong OFF response (0.00% for B=False). What this suggests is that it may be problematic for a design to switch OFF too effectively. If the MS2 arm is not present in the ensemble to at least some small extent in the no oligoB states, then the sequence may be folding *too strongly* into the OFF state to allow the oligoB to bind. Perhaps if these designs could be “loosened up” so as to not switch OFF as strongly (in States 1 and 2), their scoring would improve.

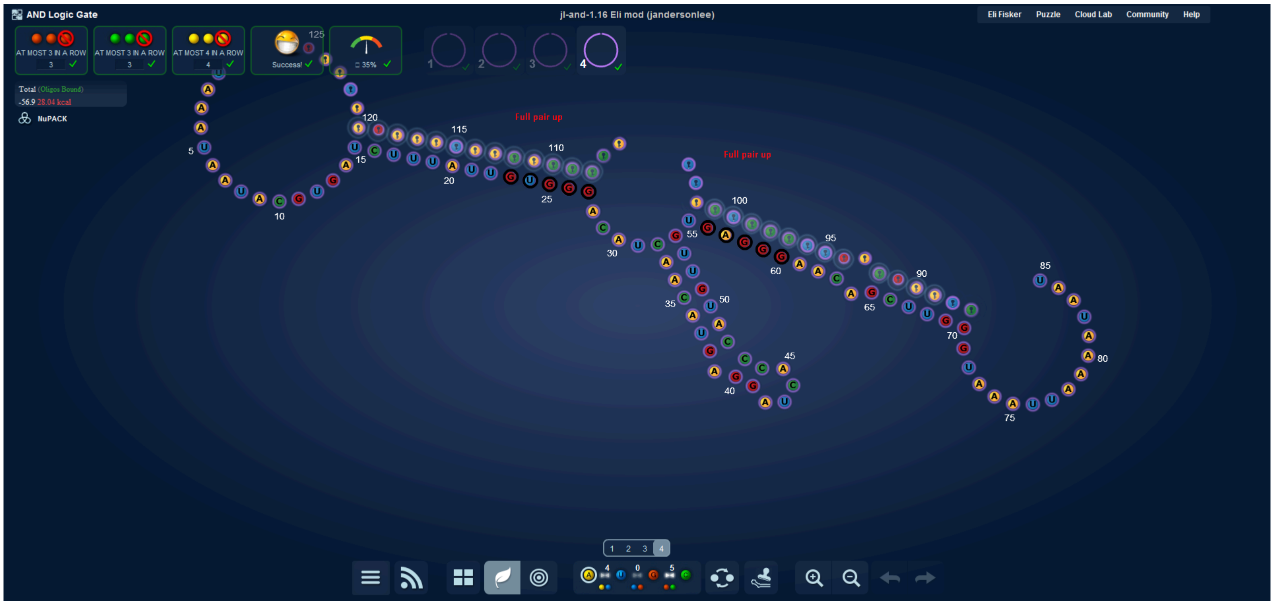

Figure 6: State 4 (TT) of 6116005 -- “jl-and-1.16 Eli mod” by jandersonlee (score 78.07)

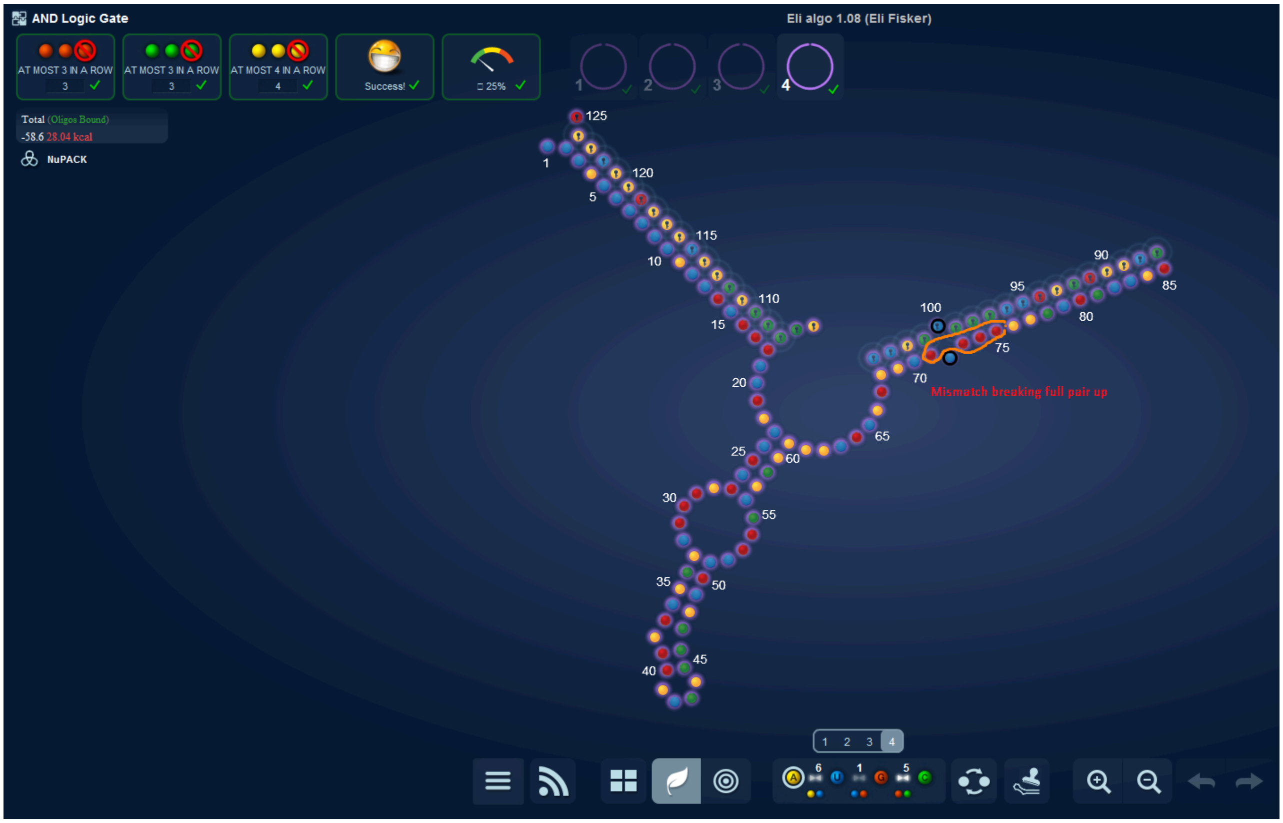

Figure 7: State 4 (TT) of 6122197-- “Eli algo 1.08” by Eli Fisker (score 87.52)

Near the top of Table 1 there is a design (6122197, Figure 7) where each of the three OFF states is weak, but non-zero. However the ON state is also weaker than desired (56.13%). Perhaps if the ON state could be strengthened, the score could be improved.

All of the designs in Table 1 have at least one state that is marked as pink or purple which may give clues as to which parts/states of the design to tweak.

R102: Logic Gates

R102 was the follow-on two-input logic function lab which re-ran the original 3 logic sub-labs (AND, OR, XOR) with mostly new candidate sequences and added five additional logic functions (A AND NOT B, A OR NOT B, NAND, NOR, and XNOR). The input oligo strands and test conditions were the same as for the original R98 lab, which allowed sequences tested in the prior lab to be (re)used as a design starting point for the new round.

Data Mining

All R98 sub-lab designs were tested in the same 4 states as R102 which allowed for previously scored R98 designs to be “rescored” for any of the eight R102 sub-labs. This allowed design sequences that failed at the targeted sub-lab to be checked for use in other modes. When this was done, some designs were found which proved to have a tendency to behave better for another logic function than the one for which they were originally designed. In particular, a few of the XOR (FTTF) designs were found to function well as an A AND NOT B (FTFF) sensor and one sequence functioned moderately as a NAND (TTTF) sensor.

In addition designs submitted by other players for R102 were also available for modification (a key strategy in Eterna labs). Both R98 and early R102 submissions were analyzed off-line for their MS2 aptamer formation probability estimates in the 4 experimental conditions using the NuPACK software with the same modeling parameters as used for the Eterna game which matched the expected lab conditions. Several designs created by other players for round R102 as well as some designs from round R98 were chosen for subsequent “mutation” rounds.

In some cases, the NuPACK evaluation determined that the MFE implied behavior of a design in one or more states was incorrect and that the ensemble behavior implied by the NuPACK pairing-probabilities data suggested a different functionality for the design. Such was the case for a R98 XOR (FTTF) submission designed by player Brourd which was determined to be a partially effective NAND (TTTF) sensor. This sequence is discussed further in the R102 NAND Case Study section.

R102 Design Mutation Strategy

From the analysis of the R98 results it seemed that designs needed to fit into a “sweet-spot” in some regards. If the MS2 aptamer formed too weakly or too strongly in an OFF state or not strongly enough in an ON state, then the design might not function well. If there was 0% probability for the design to form the MS2 aptamer in one or more OFF states, it might never switch ON as conditions changed.

The off-line NuPACK based estimations of the pairing probabilities also gave hints as to which parts of the selected designs might need to be modified.

If an OFF state had a pairing-probability less than 0.5% it might be too strongly off to ever switch on. For a sensor that was to be OFF in the presence of an oligo that might mean modifying the region of the design that is typically bound to the oligo (e.g. A’) to make the attraction weaker, or to make the attraction of A’ and A’’ stronger. For a sensor that was to be OFF in the absence of a particular oligo, that might mean weakening the bonding between the target region (e.g. A’) and other parts of the design that tended to bond in the absence of the oligo (e.g. A’’).

Conversely, if an OFF state had a probability greater than 5% it might be too weakly OFF to produce a strong fold-change response. For a sensor that was to be OFF in the presence of an oligo, that might mean modifying the design to make the attraction of the oglio a target region (e.g. A’) stronger or to weaken the attraction of that segment to other regions (e.g. A’’). For a sensor that was to be OFF in the absence of a particular oligo, that might mean strengthening the bonding between the target region (A’) and other parts of the design which tend to bond in the absence of the oligo (e.g. A’’).

If an ON state had a probability less than 80% it might be too weakly ON to generate a strong fold-change score. For a sensor that was to be ON in the presence of an oligo that might mean modifying the target region of the design that is typically bound to the oligo (e.g. A’) to make the attraction to the oglio stronger or to weaken the attraction of that region to other parts of the design (e.g. A’’) in the OFF states. For a sensor that was to be ON in the absence of a particular oligo, that might mean strengthening the bonding between the oglio target region (e.g. A’) and other parts of the design that tended to bond in the absence of the oligo (e.g. A’’).

If the MS2 aptamer (sometimes termed R’) never formed well in any ON states then it may be too strongly bonded to the segment that turned it off (R’’). Similarly if the MS2 aptamer (R’) formed too strongly in OFF states then it may need to bind more strongly to the region that turns it OFF (R’’).

For strengthening or weakening bonding, it is often possible to modify one base of a pair to strengthen or weaken a pairing. For example, a C to U mutation could weaken a GC pair to a GU to weaken an affinity; alternatively changing a U to a C might either create a stronger GC pair or weaken an AU pair to a UC mismatch. Similarly an A to G mutation could weaken an AU pairing to a weaker GU pair while a G to A mutation might convert a GU pair to a stronger AU pair. If a segment needed larger changes in affinity then creating or removing mismatched bases allows for even greater variation of energies.

When time and computational resources allow, running a full single-base mutation round on a sequence and then evaluating and selecting some mutated sequences can also be effective. This can generate some serendipitous results, especially when the ensemble includes many alternative foldings. In this case, changing just one base can be enough to change the MFE energy and shape.

Sometimes a single round of mutation would not produce enough of an improvement in the pairing-probability for the MS2 closing base pair in all 4 states to meet the preferred criteria: 0.5%<OFF<5% and 80%<ON. In such a case the variants were checked for sequences that showed some improvement in the desired direction for the states of greatest concern. One or more sequences that showed at least some improvement were then selected for another round of mutation and evaluation. Up to four rounds of mutation were tried for a few sub-labs until sufficient improvement was seen in the pairing-probabilities and some top scoring designs were generated in this way. (Table 2)

› XNOR (TFFT)

You have **1200 votes** and **0 solution slots** left.

Id	Title	Synthesis Score	Designer	Description
min max	ONE	min max	Search	Search
6500846	6499042_G82 #MUTATE_ONE #...	60 / 100	Eli Fisker	mutate G82 based on this round design 64...
6500795	6499049_C32 #MUTATE_ONE #...	60 / 100	Eli Fisker	mutate C32 based on this round design 64...
6499608	6499042_C16 #MUTATE_ONE #...	60 / 100	jandersonlee	mutate C16 based on this round design 64...
6499601	6499042_U32 #MUTATE_ONE #...	60 / 100	jandersonlee	mutate U32 based on this round design 64...
6499596	6499049_U8 #MUTATE_ONE #N...	60 / 100	jandersonlee	mutate U8 based on this round design 649...
6499549	6499042_A20 #MUTATE_ONE #...	60 / 100	jandersonlee	mutate A20 based on this round design 64...
6499542	6499042_C32 #MUTATE_ONE #...	60 / 100	jandersonlee	mutate C32 based on this round design 64...
6499567	6499042_U8 #MUTATE_ONE #N...	59 / 100	jandersonlee	mutate U8 based on this round design 649...
6500865	6499042_U59 #MUTATE_ONE #...	58 / 100	Eli Fisker	mutate U59 based on this round design 64...
6500859	6499049_U32 #MUTATE_ONE #...	58 / 100	Eli Fisker	mutate U32 based on this round design 64...
6500826	6499042_U79 #MUTATE_ONE #...	58 / 100	Eli Fisker	mutate U79 based on this round design 64...
6499554	6499042_C17 #MUTATE_ONE #...	58 / 100	jandersonlee	mutate C17 based on this round design 64...
6500845	6499049_U24 #MUTATE_ONE #...	52 / 100	Eli Fisker	mutate U24 based on this round design 64...
6499562	6499042_U4 #MUTATE_ONE #N...	49 / 100	jandersonlee	mutate U4 based on this round design 649...
6500715	6499042_G17 #MUTATE_ONE #...	47 / 100	Eli Fisker	mutate G17 based on this round design 64...
6499587	6499042_G2 #MUTATE_ONE #N...	46 / 100	jandersonlee	mutate G2 based on this round design 649...
6499537	6499042_A19 #MUTATE_ONE #...	46 / 100	jandersonlee	mutate A19 based on this round design 64...
6499557	6499042_C4 #MUTATE_ONE #N...	44 / 100	jandersonlee	mutate C4 based on this round design 649...
6499538	6499049_A19 #MUTATE_ONE #...	44 / 100	jandersonlee	mutate A19 based on this round design 64...
6499561	6499042_G31 #MUTATE_ONE #...	43 / 100	jandersonlee	mutate G31 based on this round design 64...

Table 2a: One Mutation Designs for XNOR - max score 60

› XNOR (TFFT)

You have **1200 votes** and **0 solution slots** left.

Id	Title	Synthesis Score	Designer	Description
min max	TWO	min max	Search	Search
6501320	6491758_G56_C76 title_suf...	76 / 100	Eli Fisker	mutate G56 mutate C76 NuPACK_FC=41.08 FF...
6501944	6491758_G82_G2 #MUTATE_TW...	74 / 100	mat747	mutate G82 mutate G2 NuPACK_FC=63.91 FF=...
6501357	6491758_U77_G2 title_suff...	74 / 100	Eli Fisker	mutate U77 mutate G2 NuPACK_FC=28.24 FF=...
6501134	6491758_G59_G2 #MUTATE_TW...	74 / 100	jandersonlee	mutate G59 mutate G2 NuPACK_FC=31.04 FF=...
6501111	6491758_G10_G82 #MUTATE_T...	72 / 100	jandersonlee	mutate G10 mutate G82 NuPACK_FC=75.7 FF=...
6501128	6491758_G82_G55 #MUTATE_T...	71 / 100	jandersonlee	mutate G82 mutate G55 NuPACK_FC=31.99 FF...
6501976	6491758_G56_A85 #MUTATE_T...	70 / 100	mat747	mutate G56 mutate A85 NuPACK_FC=27.16 FF...
6501969	6491758_G56_G76 #MUTATE_T...	70 / 100	mat747	mutate G56 mutate G76 NuPACK_FC=29.71 FF...
6501110	6471242_G26_U60 #MUTATE_T...	70 / 100	jandersonlee	mutate G26 mutate U60 NuPACK_FC=79.76 FF...
6501947	6491758_G49_G56 #MUTATE_T...	69 / 100	mat747	mutate G49 mutate G56 NuPACK_FC=47.06 FF...
6501946	6491758_G56_G77 #MUTATE_T...	69 / 100	mat747	mutate G56 mutate G77 NuPACK_FC=42.43 FF...
6501112	6491758_G56_U77 #MUTATE_T...	68 / 100	jandersonlee	mutate G56 mutate U77 NuPACK_FC=44.77 FF...
6502073	6499042_C32_G78 #MUTATE_T...	67 / 100	mat747	mutate C32 mutate G78 NuPACK_FC=94.79 FF...
6501351	6491758_U77_C24 title_suf...	67 / 100	Eli Fisker	mutate U77 mutate C24 NuPACK_FC=26.99 FF...
6501148	6491758_U77_U5 #MUTATE_TW...	67 / 100	jandersonlee	mutate U77 mutate U5 NuPACK_FC=29.56 FF=...
6501125	6491758_G5_G82 #MUTATE_TW...	67 / 100	jandersonlee	mutate G5 mutate G82 NuPACK_FC=33.61 FF=...
6502026	6499042_A19_A72 #MUTATE_T...	66 / 100	mat747	mutate A19 mutate A72 NuPACK_FC=866.69 F...
6501959	6491758_G5_U77 #MUTATE_TW...	66 / 100	mat747	mutate G5 mutate U77 NuPACK_FC=31.93 FF=...
6501952	6491758_U77_A57 #MUTATE_T...	66 / 100	mat747	mutate U77 mutate A57 NuPACK_FC=35.82 FF...
6501157	6491758_G5_A83 #MUTATE_TW...	66 / 100	jandersonlee	mutate G5 mutate A83 NuPACK_FC=26.64 FF=...

Table 2b: Two Mutation designs for XNOR - max score 76

› XNOR (TFFT)

You have **1200 votes** and **0 solution slots** left.

Id	Title	Synthesis Score	Designer	Description
min max	FOUR	min max	Search	Search
6501007	6133692 #MUTATE_FOUR #MIX...	100 / 100	Eli Fisker	6133692_G2_U62_G45_C61 based on a R98 AN...
6500952	6133692 #MUTATE_FOUR #MIX...	99 / 100	jandersonlee	6133692_G2_U62_G45_U85 based on a R98 AN...
6501003	6133692 #MUTATE_FOUR #MIX...	98 / 100	Eli Fisker	6133692_G2_U62_G45_G46 based on a R98 AN...
6500951	6133692 #MUTATE_FOUR #MIX...	98 / 100	jandersonlee	6133692_G2_U62_G45_A65 based on a R98 AN...
6501009	6133692 #MUTATE_FOUR #MIX...	97 / 100	Eli Fisker	6133692_G2_U62_G45_U65 based on a R98 AN...
6500961	6133692 #MUTATE_FOUR #MIX...	97 / 100	jandersonlee	6133692_G2_A66_G45_C83 based on a R98 AN...
6500950	6133692 #MUTATE_FOUR #MIX...	97 / 100	jandersonlee	6133692_G2_U62_G45_A83 based on a R98 AN...
6501006	6133692 #MUTATE_FOUR #MIX...	96 / 100	Eli Fisker	6133692_G2_U62_G45_C84 based on a R98 AN...
6500954	6133692 #MUTATE_FOUR #MIX...	96 / 100	jandersonlee	6133692_G2_U62_G45_G66 based on a R98 AN...
6500949	6133692 #MUTATE_FOUR #MIX...	96 / 100	jandersonlee	6133692_G2_U62_G45_U66 based on a R98 AN...
6501018	6133692 #MUTATE_FOUR #MIX...	95 / 100	Eli Fisker	6133692_G2_U62_G45_C65 based on a R98 AN...
6501008	6133692 #MUTATE_FOUR #MIX...	95 / 100	Eli Fisker	6133692_G2_U62_G45_U67 based on a R98 AN...
6500953	6133692 #MUTATE_FOUR #MIX...	95 / 100	jandersonlee	6133692_G2_U62_G45_A84 based on a R98 AN...
6501019	6133692 #MUTATE_FOUR #MIX...	94 / 100	Eli Fisker	6133692_G2_A66_G45_C67 based on a R98 AN...
6501014	6133692 #MUTATE_FOUR #MIX...	94 / 100	Eli Fisker	6133692_G2_A66_G45_G83 based on a R98 AN...
6501002	6133692 #MUTATE_FOUR #MIX...	93 / 100	Eli Fisker	6133692_G2_U62_G45_G85 based on a R98 AN...
6500965	6133692 #MUTATE_FOUR #MIX...	93 / 100	jandersonlee	6133692_G2_U62_U1_G45 based on a R98 AND
6500962	6133692 #MUTATE_FOUR #MIX...	93 / 100	jandersonlee	6133692_G2_G85_G45_G66 based on a R98 AN...
6500964	6133692 #MUTATE_FOUR #MIX...	91 / 100	jandersonlee	6133692_G2_A66_A82_G45 based on a R98 AN...

Table 2c: Four Mutation designs for XNOR - max score 100

R102 Submissions

The three Eterna players taking part in this strategy each used some or all of their lab submission slots for designs created and selected via this process. A player-generated on-line "EternaScript" (javascript incorporated into the browser-based Eterna game platform) was used to automate the submission of the selected candidate sequences. For some sub-labs, more candidates were generated than there were open submission slots and so submissions were somewhat randomly selected from the available options with a range of "rating" scores.

R102 Results

The R102 results are summarized in a Google Sheet called R102_results_logic. The original copy was released by the Eterna lab administrators. In this version, the sub-lab results have been separated into separate tabs for easier reference and sorted by score. What follows is a brief discussion of the performance of the data-mine/mutation/evaluation strategy for each sub-lab.

FFFT - A AND B

The top design scoring 98.97 was by Eli Fisker but was not one of the designs created using the automated mutation/evaluation runs described here. It was a modification of a prior round design that had scored 88 in R98.

Of the 16 winning designs (Eterna score of 94 or better[2]), 12 were mods of the same prior round design and four were one or two base mutations of the sort that are described here, however they were not evaluated and selected via the ranking scheme described above.

In all 13 of the top 45 designs were designed using the mutation/evaluation strategy and scored between 90 and 94. One of these designs scored 93.63 which the Eterna game rounded up to 94 and hence was considered a winning design for the round.

This was the least successful sub-lab for this strategy.

FTFF - A AND NOT B

This sub-lab was a big win for data-mining. At least 8 designs scoring 100 were simply reruns of failed XOR (FTTF) designs from R98 that worked as FTFF designs. Another 45 designs that scored 100 were based on mutations of well-scoring prior lab designs. In all 139 winning designs scored 94 or better and most of these were mutations of data-mined designs.

Even though many of the data-mined designs already scored 100, the mutation/evaluation strategy was still able to improve on some of them. The R98:XOR designs 6120320 and 6117637 scored 100 when rerun as A AND NOT B designs meaning they had a FoldChange value of at least 75. However the mutate/evaluate strategy found 4 designs with a higher folding score for 6120320 and 3 designs with a higher folding score for 6117637 meaning they switch more strongly than the originals, as well as several other designs that still scored 100. (Table 3)

[2] Actually 93.500 or better since Eterna reports scores as integers with rounding.

DesignID	Design	Eterna_Score	FoldChange	KDOFF	KDON	Puzzle_Name
6492835	6120320 #MUTATE_TWO #NUPACK_SCORING	100.00	132.14	813.16	6.15	A AND NOT B (FTFF)
6479056	6120320_A40 #MUTATE_ONE	100.00	114.07	784.98	6.88	A AND NOT B (FTFF)
6574511	6117637_C27_U79 #MUTATE_TWO #MIXED_SCORING	100.00	113.20	720.29	6.36	A AND NOT B (FTFF)
6492827	6120320 #MUTATE_TWO #NUPACK_SCORING	100.00	109.27	612.96	5.61	A AND NOT B (FTFF)
6479102	6117637_C27 #MUTATE_ONE	100.00	109.21	697.80	6.39	A AND NOT B (FTFF)
6557696	6117637_U27_A3 #MUTATE_TWO #MIXED_SCORING	100.00	107.80	767.04	7.12	A AND NOT B (FTFF)
6479103	6117637_rerun #MUTATE_ONE	100.00	102.04	645.98	6.33	A AND NOT B (FTFF)
6479121	6117637_C20 #MUTATE_ONE	100.00	99.58	574.25	5.77	A AND NOT B (FTFF)
6479126	6117637_A19 #MUTATE_ONE	100.00	98.27	607.11	6.18	A AND NOT B (FTFF)
6557688	6117637_U27_G32 #MUTATE_TWO #MIXED_SCORING	100.00	97.73	591.56	6.05	A AND NOT B (FTFF)
6479088	6120320_C30 #MUTATE_ONE	100.00	95.56	697.51	7.30	A AND NOT B (FTFF)
6557684	6117637_G30_G31 #MUTATE_TWO #MIXED_SCORING	100.00	91.08	514.54	5.65	A AND NOT B (FTFF)
6557685	6117637_G32_C5 #MUTATE_TWO #MIXED_SCORING	100.00	90.39	583.25	6.45	A AND NOT B (FTFF)
6479117	6117637_U27 #MUTATE_ONE	100.00	89.05	540.61	6.07	A AND NOT B (FTFF)
6479057	6120320_rerun #MUTATE_ONE	100.00	88.08	630.53	7.16	A AND NOT B (FTFF)
6479097	6120320_G4 #MUTATE_ONE	100.00	83.56	572.10	6.85	A AND NOT B (FTFF)
6479114	6117637_G31 #MUTATE_ONE	100.00	81.41	548.78	6.74	A AND NOT B (FTFF)
6557694	6117637_G31_C30 #MUTATE_TWO #MIXED_SCORING	100.00	78.79	527.31	6.69	A AND NOT B (FTFF)
6479083	6120320_G2 #MUTATE_ONE	100.00	77.88	517.55	6.65	A AND NOT B (FTFF)
6557682	6117637_G32_C3 #MUTATE_TWO #MIXED_SCORING	100.00	76.91	598.20	7.78	A AND NOT B (FTFF)

Table 3: Improving FoldChange on High Scoring A AND NOT B Designs

FTTF - XOR

In this sub-lab, no designs scored 100 but there were eight winning designs that scored 94 or better and seven of these were designed with the mutation/evaluation strategy including the top two designs. Another six designs scored at least 90.0 and five of these were designed with the mutation/evaluation strategy. On the whole the mutation/evaluation strategy produced more winners and more top-ranked designs than other player strategies for this sub-lab.

FTTT - OR

The twenty-four winning designs included six that scored 100. Of these, 15 were designed with the mutation/evaluation strategy (three that scored 100 and 12 more that scored at least 93.5). Of the nine remaining winning designs, all but one were designed by Eli Fisker.

TFFF - NOR

This was seemingly a harder challenge. No designs scored 100. The four winning designs were all designed with the mutation/evaluation strategy. Of the five other designs that scored 90.0 or better, all but one were designed with the mutation/evaluation strategy.

TFFT - XNOR

The twenty winning designs for this sub-lab were all mutation/evaluation designs; one of these scored 100. The highest ranked non mutation/evaluation design was 28th and scored 77.49. All of the top designs were 4 mutations from a R98 AND design that was selected via data-mining for its XNOR behavior leanings. The best score for a mutation/evaluation design with only two mutations was 76.39. The best score for a mutation/evaluation design with just one mutation was 60.38. Unfortunately the original design was not rerun to see how it would have scored as an XNOR without modification.

TTFT - A OR NOT B

Of the 32 winning designs for this sub-lab, all but two were mutation/evaluation designs; five of these scored 100. One design of particular interest was R98 OR:6112196 which the NuPACK MFE shapes suggested would be an OR sensor. Looking at the R98 lab Kd data for all 4 states, it was selected as an TTFT candidate instead and scored 87 when rerun unchanged in the A OR NOT B sub-lab; it had only scored 30 as an OR sensor. Many different single-base mutation variants of this sequence were run as A OR NOT B and four of them scored a perfect 100 (a 13 point improvement). In all, 26 single-base variants of this design were winners. The TTFT sub-lab winners also included four other two-base variants of three other starter sequences (one of which also scored 100) showing that this was not an isolated case.

TTTF - NAND

There were nine winning designs for the NAND sub-lab. The top scoring (100) design was designed by the player worseize. The other eight winning designs were mutation/evaluation designs including one that scored 96.83. Of the 42 designs that score 90.0 or better, all but 6 were mutation/evaluation designs.

R102 NAND Case Study

In the case of the NAND sub-lab, one data-mined XOR design from R98 (design 6130947, "X1-OligoB-X3-OligoA: Submission 2" by Brourd) was deemed to be a promising NAND starter sequence for this strategy. When the R98 results were rescored for NAND it was estimated that this one design would have had a relatively strong NAND fold-change value of 16.78. Furthermore, its MS2 pairing values were promising (FF=0.6818, TF=0.9977, FT=0.9408, TT=0.0233) such that if the FF value could be strengthened and the TT value weakened it could score even better as a NAND sensor. This was in spite of the fact that the state 1 (FF) MFE shape suggested that the design should work as an XOR sensor; both the NuPACK pairing data and the R98 lab results suggested otherwise. Ultimately a selection of single and double mutation designs were submitted. The pre-submission evaluation data have been combined with the lab results for these designs in a single Google Sheet.

A full in-silico single-mutation run of this initial sequence was performed with all 3 possible substitutions made for all bases except bases 25 to 44 which formed the MS2 aptamer and the FF, TF, FT, and TT MS2 pairing-probability values were calculated for each of these mutated sequences. In addition, a scaled estKDno, estKDA, estKDB, and estKDAB were computed by assuming that a proportional increase in FF post-mutation would result in a proportional decrease in KDno, and similarly for TF/KDA, FT/KDB, and TT/KDAB. That is assuming that if the probability of the MS2 aptamer forming was doubled, the Kd concentration for half illumination should be halved, and vice versa.

All of these single-mutation sequences were submitted in R102 for the NAND sub-lab. In addition, several of what seemed to be promising single-base mutations were selected for an additional round of single-base mutation: G23, G45, C47, U55, and U67.

Most of the 187 single-mutation sequences did not improve the scoring of the design; however, given that all possible single-base mutations were tested, this is not surprising. Of the 187 mutations tried, 30 (16%) improved the EternaScore by up to 6.5 points (avg 1.6, stdev 1.6) or left it unchanged, while 157 (84%) degraded the EternaScore by up to 56.8 points (avg -16.2, stdev 14.7).

If the single-base mutations were selected for with some considerations that were applied to other sub-labs, the situation improved somewhat. For ON states (FF, TF, and FT) the MS2 probability was restricted to 60% or greater while the OFF state (TT) was constrained to 0.5%<OFF<10%. These constraints eliminated 107 of the single-base mutations, only two of which were slight improvements to the scoring of the original sequence. Of the 80 remaining sequences, 28 (35%) were now improvements (avg +1.7, stdev 1.6) and 52 (65%) degraded the performance (avg -8.7, stdev 10.5).

The assumption that a scaled estKDno, estKDA, estKDB, and estKDAB could be computed based on the proportional increase/decrease in predicted MS2 percentage ultimately proved to be false, with no correlation seen between estKDON and measured KDON and R^2=0.082 for estKDOFF and measured KDOFF for the tested combined single-base and two-base mutation results. Of the 5 single mutation designs that were selected for additional mutation, the two that had degraded responses were also the most suspect in the NuPACK estimates, with 6130947_G23 having FF=73.1% (<80%) and 6130947_U55 having TT=0.2% (<0.5%) although both had decent estFC estimates derived from estKDOFF/estKDON. If it was known at the outset that the estKDON, estKDOFF, and estFC were not to be trusted, these two sequences might not have been selected for further mutations. The other three single mutations (G45, C47, and U67) proved to be beneficial as were many of the dual-mutation designs derived from these.

When selecting from those tested sequences where the ON states (FF, TF, and FT) MS2 percentage was restricted to 60%<ON and the OFF state (TT) was constrained to 0.5%<OFF<10%, the results were relatively good. Of these 190 cases, just over 48% improved the EternaScore of the modified design relative to the starting sequence. The highest scoring design 6130947_U67_A4 scored 96.83, which was enough to make it one of the only two winning designs for the sub-lab.

When selecting those designs where the ON states (FF, TF, and FT) MS2 percentage was restricted to 80%<ON and the OFF state (TT) was constrained to 0.5%<OFF<2%, the specificity for improved designs was better, but the selectivity was less. With the tighter constraints 59 of 89 designs selected or just over 66% improved the EternaScore of the modified design relative to the starting sequence. However an additional 33 designs that would have been selected at the 60%<ON and 0.5%<OFF<10% level were rejected. As long as the objective was to find *some* designs that performed well rather than detect *all* mutations that could improve a design, the tighter constraints might be worthwhile as a higher percentage of successful designs would have been found in testing a smaller sample of mutants.

While the best single-base mutation (U67) improved the EternaScore from that of the starting sequence at 86.81 to 93.31 (+6.50), the addition of a second mutation (U67, A4) further improved the EternaScore to a winning value of 96.83 – just over 10 points of improvement with two mutations.

Overall, the NAND sub-lab case results suggest that the technique of modeling the pairing probability of the MS2 aptamer closing pair and selecting designs based on its ON versus OFF pairing-probabilities can be a useful way to trim the search space as well as a useful way to evaluate and data-mine sequences off-line. Likewise, selecting one or more promising mutated sequences and running additional rounds of simulated mutations seems a useful way to further improve the results. This validated some of the expectations based on the evaluations of the R98 AND sub-lab results.

Conclusions

While the Eterna game presents players with the MFE shape predicted for an RNA sequence under different experimental conditions, that is not always predictive of the true functioning of the design. Adding the pairing-probability data from a single carefully selected base-pair was helpful to characterize the RNA sequences for the task of designing a multi-state sensor using the MS2 signaling molecule.

The measured Kd ratios of OFF and ON states were used in scoring design sequences in the in-vitro lab. A hypothesis that proportional changes in the MS2 pairing probabilities could be used to estimate changes in the Kd behavior of the sequences turned out to be false as no significant correlation was found between predicted and measured Kd values.

In spite of this, players were able to leverage the experimental data and initial sequences from the initial lab round to develop a constraint-based evaluation scheme that generated more successful sequences in the second lab round. Some unsuccessful designs from the first lab round were data-mined to find successful starters or sequences for the same or other logic functions in the second round. In addition, analysis of early submissions of untested sequences designed for the second round produced some additional starters for “mutation” leading to winning designs.

The data-mining/mutation/evaluation strategy produced winning designs (scores of 94 or better[3]) in all sub-labs. In 75% of the sub-labs (all except AND and NAND) this strategy produced a top-ranked design (i.e. as good as or better than a player-generated design); in half of the sub-labs, it produced a design that scored 100.

[3] The AND lab mutation/evaluation design scoring “94” in the game actually scored 93.63 which is “rounded up” in the game interface to be reported as 94, so technically a winning design.

Addenda

Strategy VARY_MS2

I have found one more strategy we tried heavily out in the OR lab. We didn't use it elsewhere. Wasn't there a time where we were allowed to cycle through different MS2 in the lab? I don't recall which lab it came up in. I recall it worked quite fine. I could abuse it to make better pairings in the aptamer switching regions. I also think this is the best potential use of the strategy. It takes some fitting to the region it has to pair with. So if one mutates in the MS2, one also has to mutate in the MS2 turnoff site, to make full use of it.

We transferred designs from the R98 lab as usual. But as an add on we also played with mutating in MS2 itself, instead of mutating in the rest. VARY_MS2. There are also cases where this improved the design score.

First and best case

R98 original score 87
https://eternagame.org/game/browse/6096395/?filter1=Id&filter1_arg1=6115071&filter1_arg2=6115071

R102 score 93
https://eternagame.org/game/browse/6434654/?filter1=Id&filter1_arg1=6509187&filter1_arg2=6509187

Mutate ONE

Change of a single GU to a GC made the design too stable to properly switch.

This design by Malcolm was moved from XOR with an original score of 30% to an immediate score of 100%. A round of MUTATE_ONE was run. One of the mutations of a GU pair to a GC pair probably made the switching region in state 1 and 3 too stable.

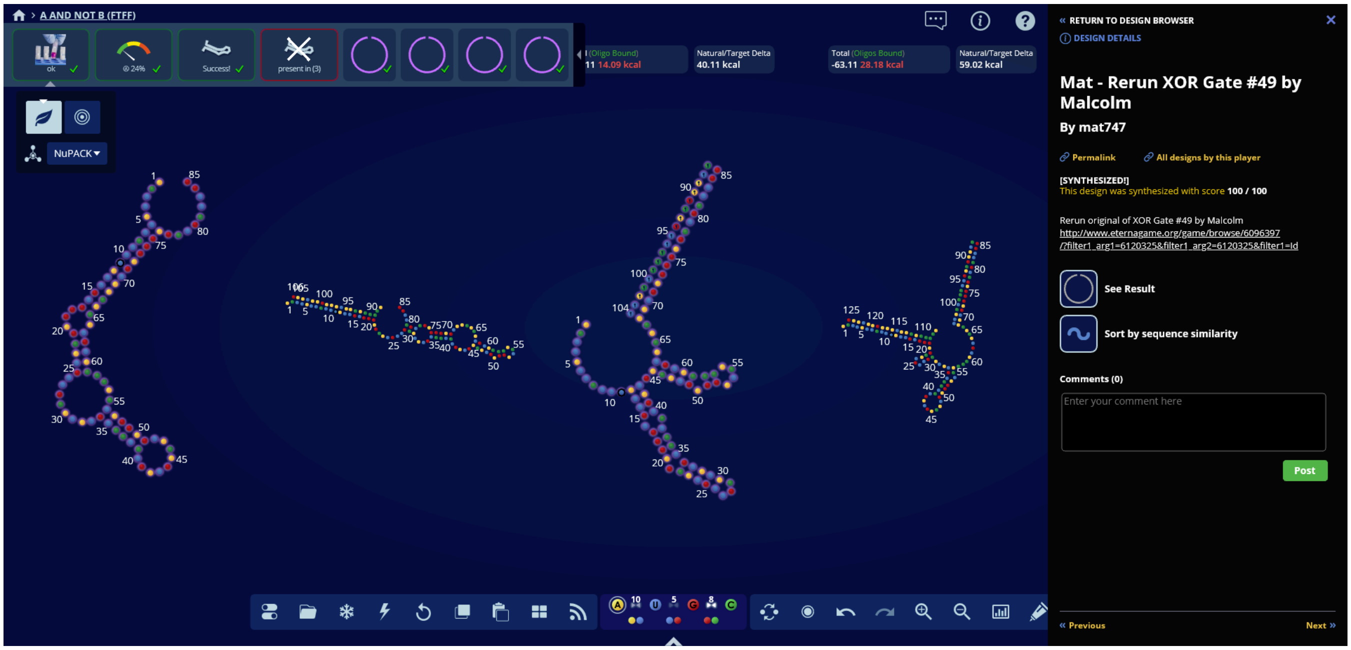

https://eternagame.org/game/browse/6434626/?filter1=Id&filter1_arg1=6479271&filter1_arg2=6479271

The C11 mutation from U11 resulted in a score drop to 64%

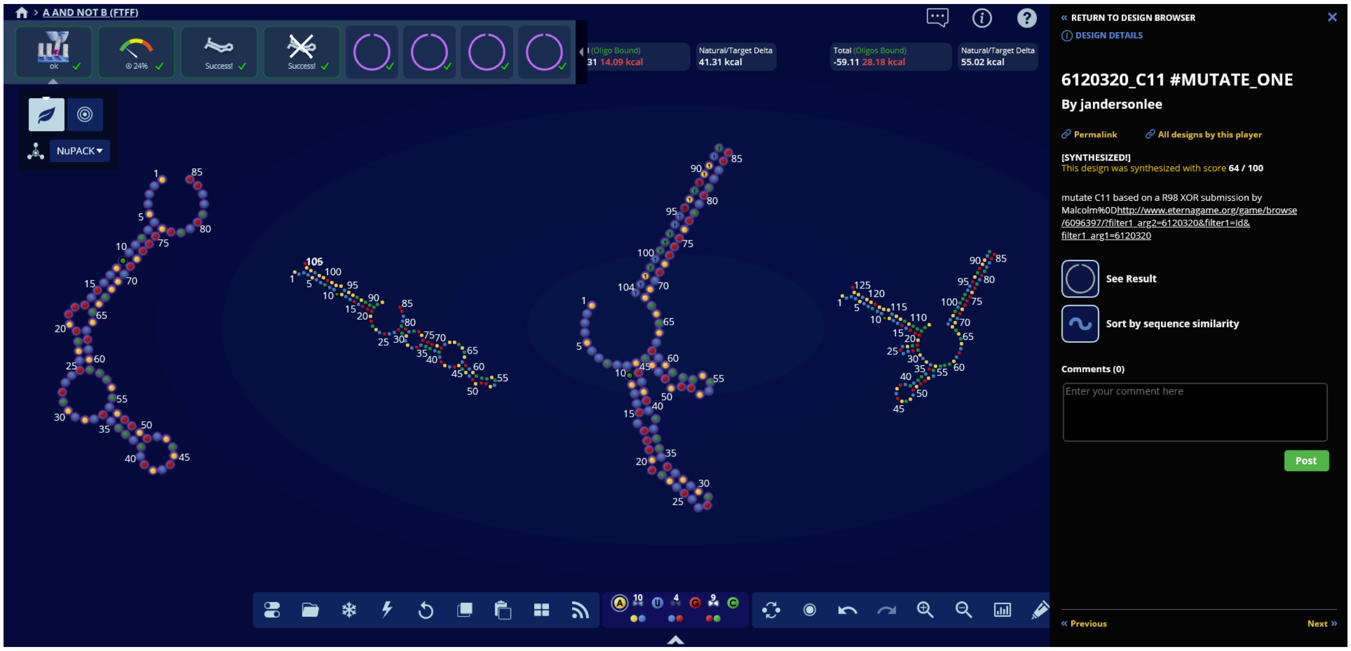

https://eternagame.org/game/browse/6434626/?filter1=Id&filter1_arg1=6479089&filter1_arg2=6479089

Task to find examples for: *For example, a C to U mutation could weaken a GC pair to a GU to weaken an affinity; alternatively changing a U to a C might either create a stronger GC pair or weaken an AU pair to a UC mismatch. Similarly an A to G mutation could weaken an AU pairing to a weaker GU pair while a G to A mutation might convert a GU pair to a stronger AU pair. If a segment needed larger changes in affinity then creating or removing mismatched bases allows for even greater variation of energies.*

Single base change from a G to an A - score jump from 87 to 96. Resulting in a GU pair in state 1 and 2 becoming a stronger AU pair. Strengthening a stem region that already has two weak GU pairs in state

1 and one in state 2. Also resulting in a GC pair in state 3 and 4 in a really long oligo match being an A-C mismatch that is weakening the bind and likely aiding the oligo in detaching.

Score 87

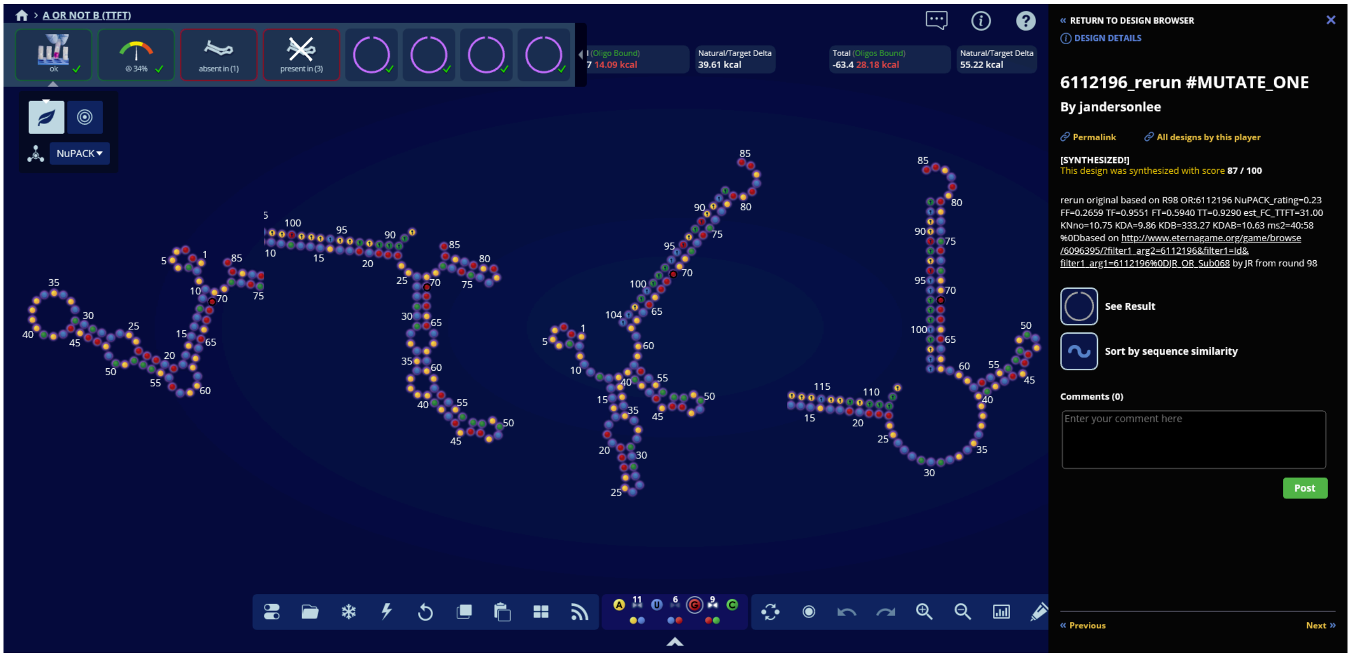

https://eternagame.org/game/browse/6434649/?filter1=Id&filter1_arg1=6485329&filter1_arg2=6485329

Score 96

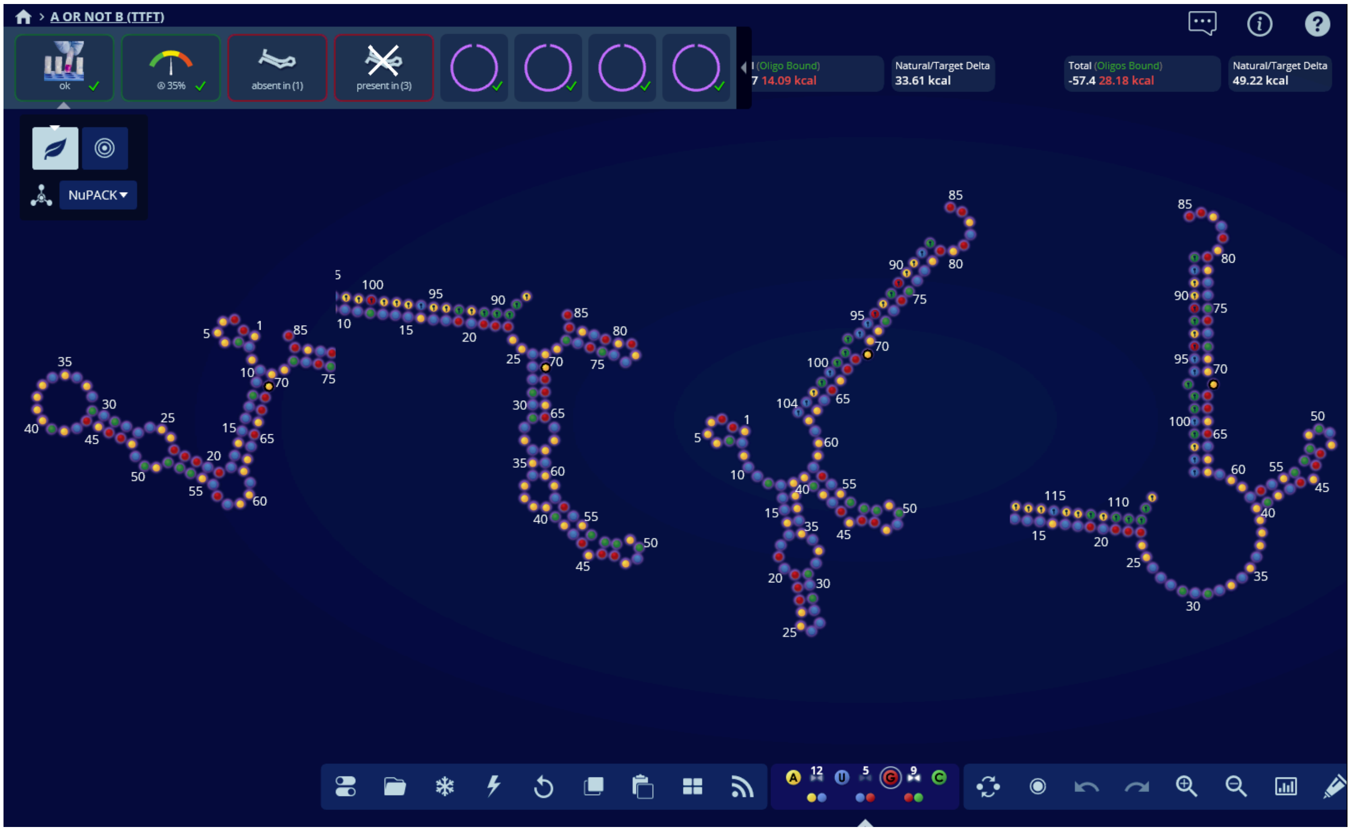

https://eternagame.org/game/browse/6434649/?filter1=Id&filter1_arg1=6481560&filter1_arg2=6481560

A AND NOT B notes

Based on Mutate One designs with single mutation.
GC to GU in state 3

Score 69, C28 to U28

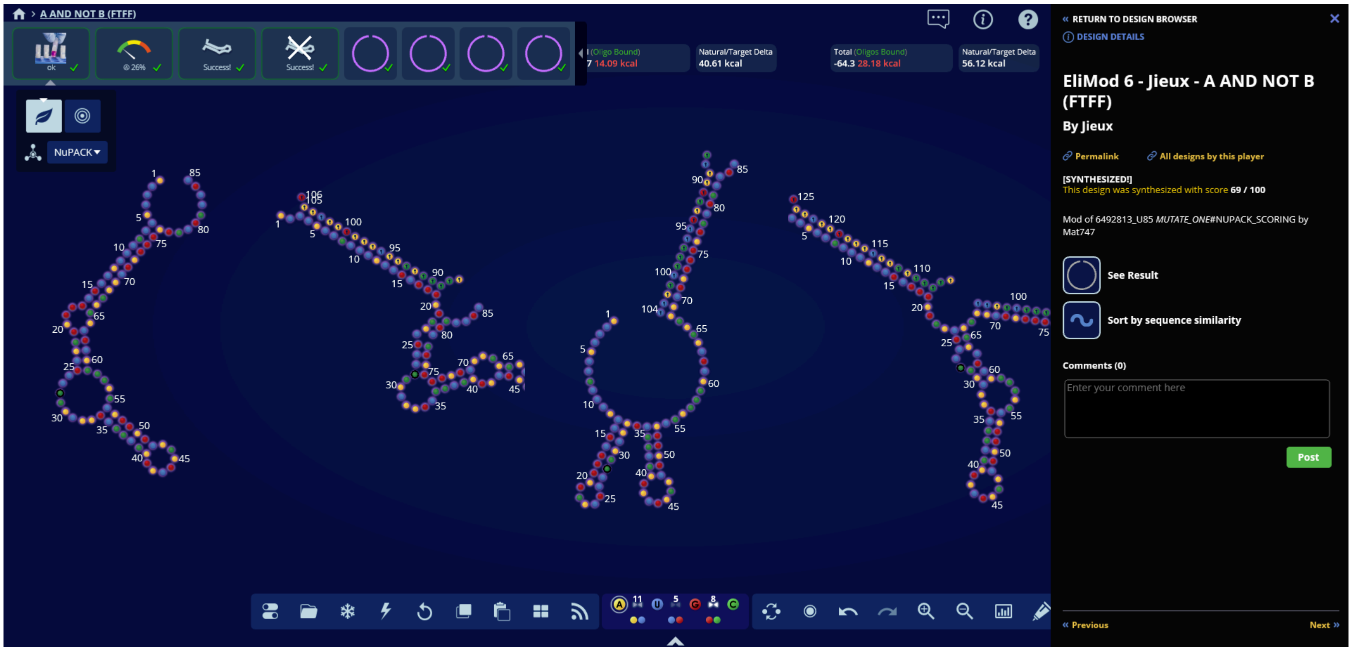

https://eternagame.org/game/browse/6434626/?filter1=Id&filter1_arg1=6653279&filter1_arg2=6653279

Score 100

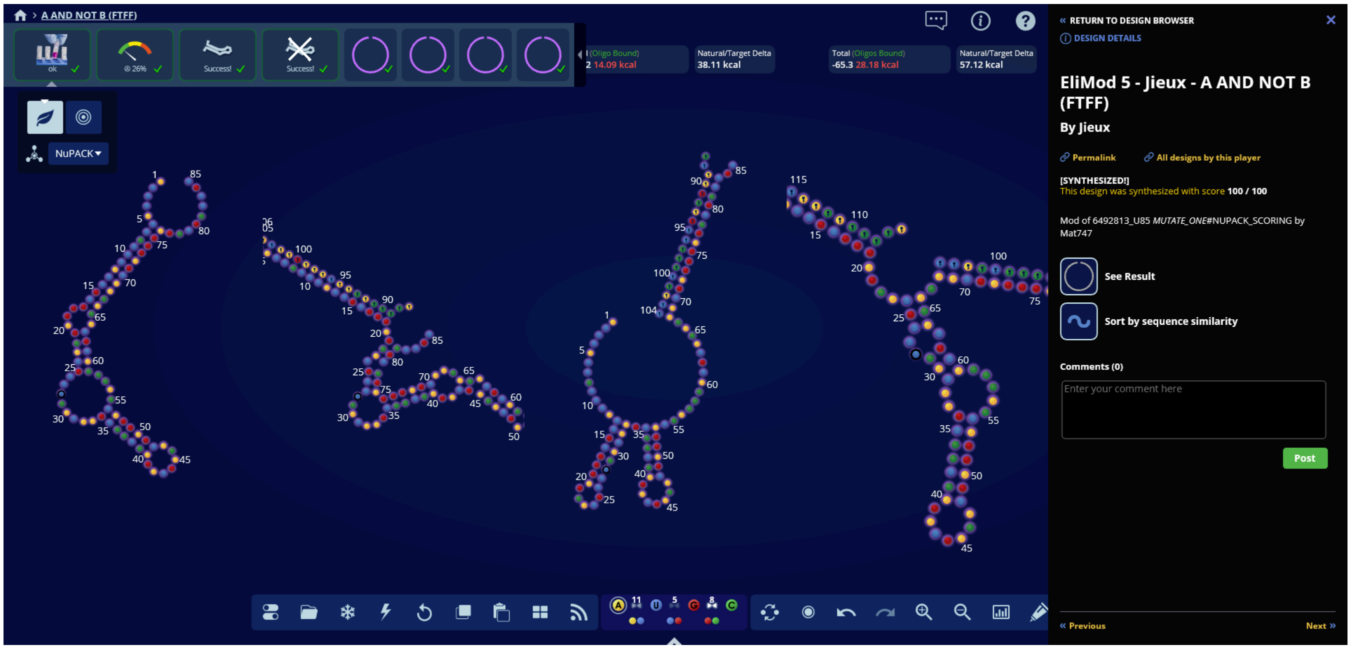

https://eternagame.org/game/browse/6434626/?filter1=Id&filter1_arg1=6653274&filter1_arg2=6653274

From mutate Two to mutate One

I found a case where you ran a mutate one round on top of a mutate two round (with only a single design submitted from that run). So the strategy didn't just go from mutate one to mutate two and if still

not improved enough, to mutate four. It could also go like mutate two and then mutate one. In this case the starting point was already a winner (99), but doing the extra mutate one round yielded five 100 and a good bunch of other winners. You must have picked just one of the starter mutate two and then run a mutate one on this one. Because it is two bases different from the originator design by Malcolm which scored 30 as an XOR design. I recall that you sometimes didn't get many designs as output from a mutate round, so I'm guessing you just picked the most promising one and were trying to expand the pool of promising offspring. In this case the original design from XOR was rerun and got an immediate 100.

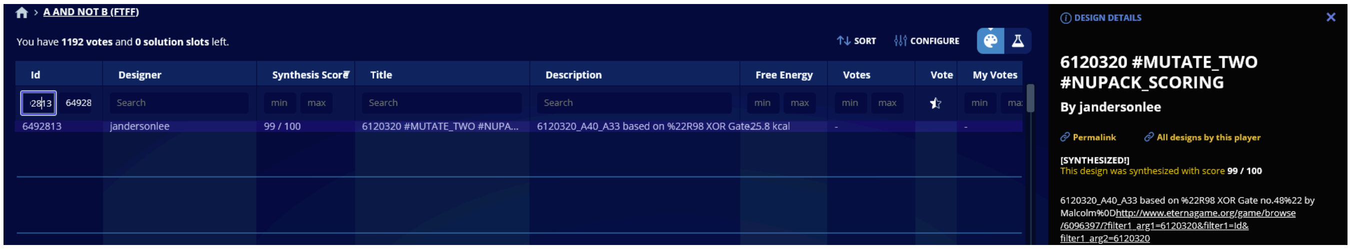

https://eternagame.org/game/browse/6434626/?filter1=Id&filter1_arg1=6492813&filter1_arg2=6492813

6492813

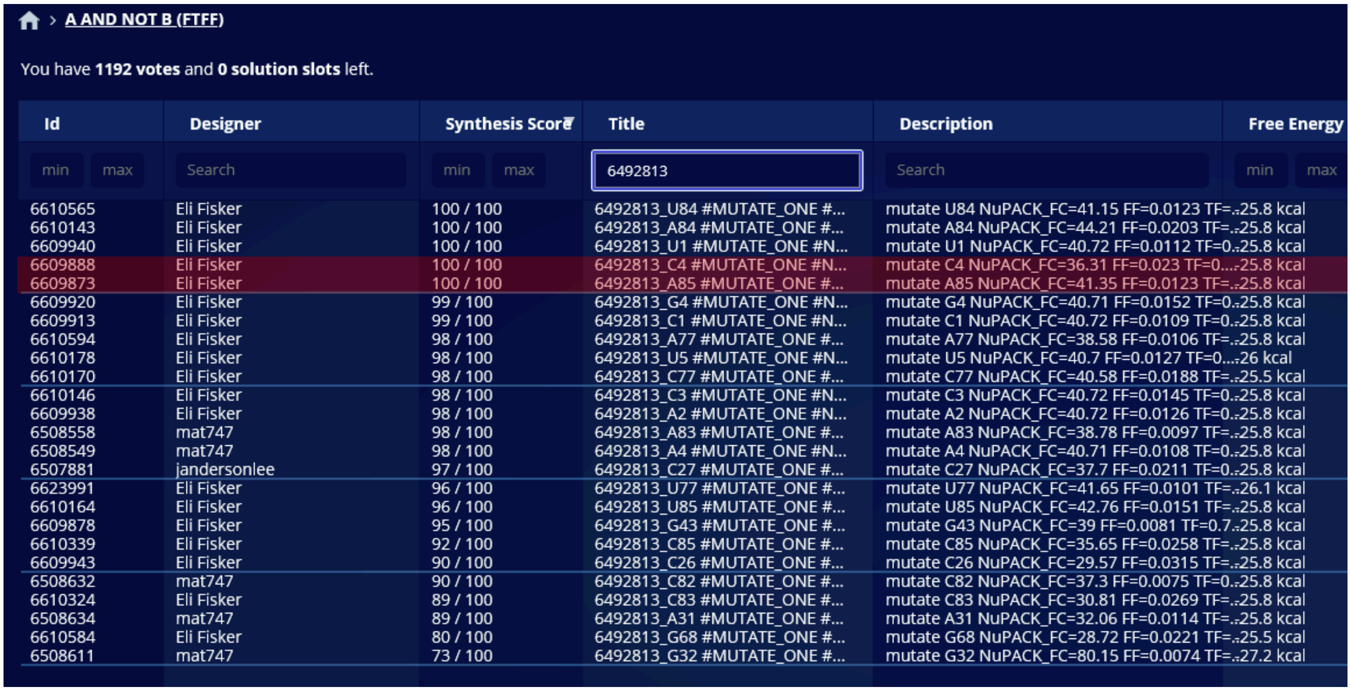
A AND NOT B (FTFF)

You have **1192 votes** and **0 solution slots** left.

Id	Designer	Synthesis Score	Title	Description	Free Energy
6610565	Eli Fisker	100 / 100	6492813_U84 #MUTATE_ONE #...	mutate U84 NuPACK_FC=41.15 FF=0.0123 TF=...	25.8 kcal
6610143	Eli Fisker	100 / 100	6492813_A84 #MUTATE_ONE #...	mutate A84 NuPACK_FC=44.21 FF=0.0203 TF=...	25.8 kcal
6609940	Eli Fisker	100 / 100	6492813_U1 #MUTATE_ONE #N...	mutate U1 NuPACK_FC=40.72 FF=0.0112 TF=0...	25.8 kcal
6609888	Eli Fisker	100 / 100	6492813_C4 #MUTATE_ONE #N...	mutate C4 NuPACK_FC=36.31 FF=0.023 TF=0....	25.8 kcal
6609873	Eli Fisker	100 / 100	6492813_A85 #MUTATE_ONE #...	mutate A85 NuPACK_FC=41.35 FF=0.0123 TF=...	25.8 kcal
6609920	Eli Fisker	99 / 100	6492813_G4 #MUTATE_ONE #N...	mutate G4 NuPACK_FC=40.71 FF=0.0152 TF=0...	25.8 kcal
6609913	Eli Fisker	99 / 100	6492813_C1 #MUTATE_ONE #N...	mutate C1 NuPACK_FC=40.72 FF=0.0109 TF=0...	25.8 kcal
6610594	Eli Fisker	98 / 100	6492813_A77 #MUTATE_ONE #...	mutate A77 NuPACK_FC=38.58 FF=0.0106 TF=...	25.8 kcal
6610178	Eli Fisker	98 / 100	6492813_U5 #MUTATE_ONE #N...	mutate U5 NuPACK_FC=40.7 FF=0.0127 TF=0....	26 kcal
6610170	Eli Fisker	98 / 100	6492813_C77 #MUTATE_ONE #...	mutate C77 NuPACK_FC=40.58 FF=0.0188 TF=...	25.5 kcal
6610146	Eli Fisker	98 / 100	6492813_C3 #MUTATE_ONE #N...	mutate C3 NuPACK_FC=40.72 FF=0.0145 TF=0...	25.8 kcal
6609938	Eli Fisker	98 / 100	6492813_A2 #MUTATE_ONE #N...	mutate A2 NuPACK_FC=40.72 FF=0.0126 TF=0...	25.8 kcal
6508558	mat747	98 / 100	6492813_A83 #MUTATE_ONE #...	mutate A83 NuPACK_FC=38.78 FF=0.0097 TF=...	25.8 kcal
6508549	mat747	98 / 100	6492813_A4 #MUTATE_ONE #N...	mutate A4 NuPACK_FC=40.71 FF=0.0108 TF=0...	25.8 kcal
6507881	jandersonlee	97 / 100	6492813_C27 #MUTATE_ONE #...	mutate C27 NuPACK_FC=37.7 FF=0.0211 TF=0...	25.8 kcal
6623991	Eli Fisker	96 / 100	6492813_U77 #MUTATE_ONE #...	mutate U77 NuPACK_FC=41.65 FF=0.0101 TF=...	26.1 kcal
6610164	Eli Fisker	96 / 100	6492813_U85 #MUTATE_ONE #...	mutate U85 NuPACK_FC=42.76 FF=0.0151 TF=...	25.8 kcal
6609878	Eli Fisker	95 / 100	6492813_G43 #MUTATE_ONE #...	mutate G43 NuPACK_FC=39 FF=0.0081 TF=0.7...	25.8 kcal
6610339	Eli Fisker	92 / 100	6492813_C85 #MUTATE_ONE #...	mutate C85 NuPACK_FC=35.65 FF=0.0258 TF=...	25.8 kcal
6609943	Eli Fisker	90 / 100	6492813_C26 #MUTATE_ONE #...	mutate C26 NuPACK_FC=29.57 FF=0.0315 TF=...	25.8 kcal
6508632	mat747	90 / 100	6492813_C82 #MUTATE_ONE #...	mutate C82 NuPACK_FC=37.3 FF=0.0075 TF=0...	25.8 kcal
6610324	Eli Fisker	89 / 100	6492813_C83 #MUTATE_ONE #...	mutate C83 NuPACK_FC=30.81 FF=0.0269 TF=...	25.8 kcal
6508634	mat747	89 / 100	6492813_A31 #MUTATE_ONE #...	mutate A31 NuPACK_FC=32.06 FF=0.0114 TF=...	25.8 kcal
6610584	Eli Fisker	80 / 100	6492813_G68 #MUTATE_ONE #...	mutate G68 NuPACK_FC=28.72 FF=0.0221 TF=...	25.5 kcal
6508611	mat747	73 / 100	6492813_G32 #MUTATE_ONE #...	mutate G32 NuPACK_FC=80.15 FF=0.0074 TF=...	27.2 kcal

Perhaps the expansion round was due to the rerun showing up as not fulfilling conditions in the lab:

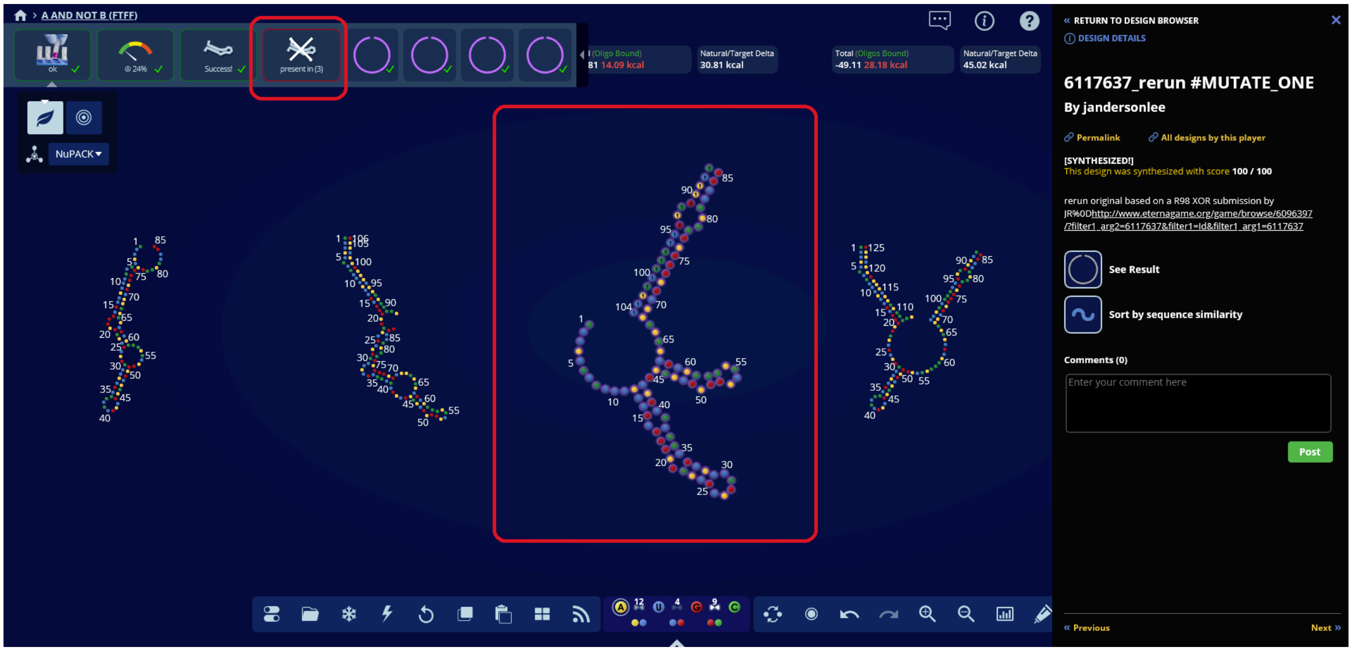

In this lab, there are several different series that originate from 6120320. (There may be more than the ones I have mentioned below)

These series: 6492813, 6492835, 6120320 (mutate one and two)

Things to consider:
-Sliding parts of the design towards or from each other - what is changed here is regularly the distance of MS2 in relation to the oligo/s or the oligo's position in relation to each other.
- Bonus of static stems beyond getting rid of potential unused bases, is coaxial stacking with bound oligo or the tether.

One of the things I find most impressive is how few of the mutate/evaluation designs that score really low. They start getting good really fast, even in a de novo run.

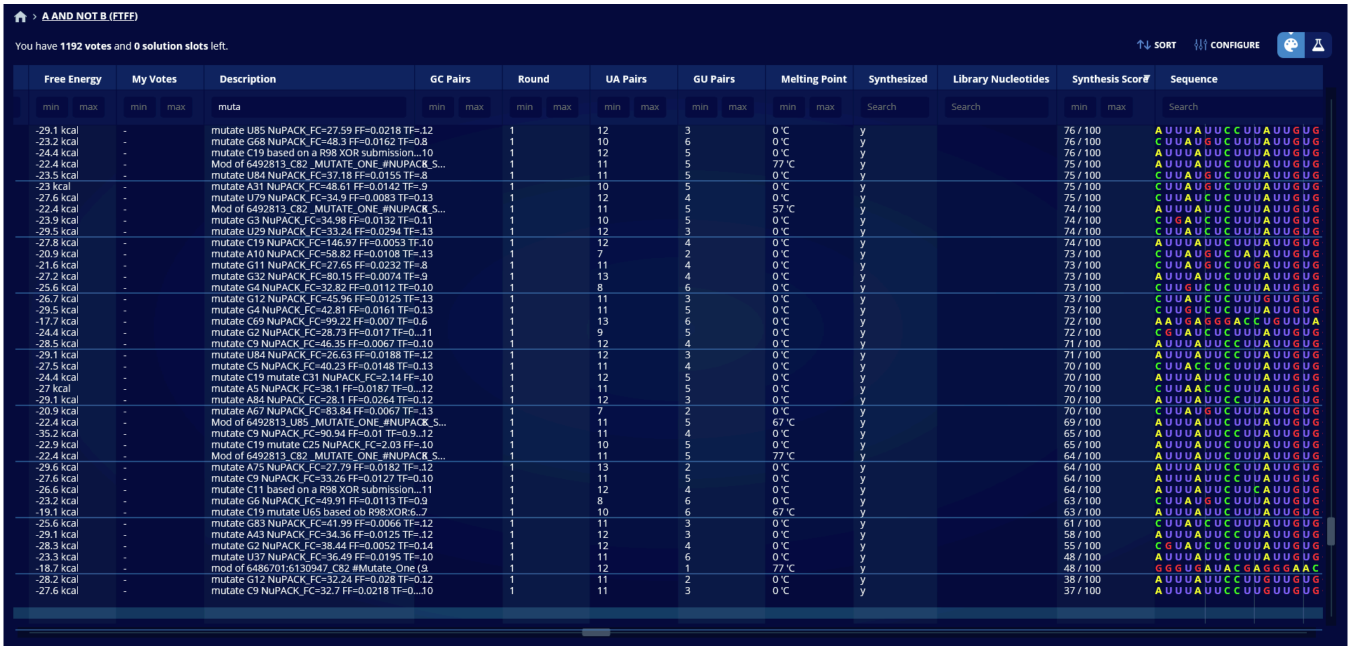

A AND NOT B (FTFF)

You have **1192 votes** and **0 solution slots** left.

SORT CONFIGURE

Free Energy	My Votes	Description	GC Pairs	Round	UA Pairs	GU Pairs	Melting Point	Synthesized	Library Nucleotides	Synthesis Score	Sequence
min max	min max	muta	min max	min max	min max	min max	min max	Search	Search	min max	Search
-29.1 kcal	-	mutate U85 NuPACK_FC=27.59 FF=0.0218 TF=.	12	1	12	3	0 'C	y		76 / 100	AUUUAUUCCUUAUUGUG
-23.2 kcal	-	mutate G68 NuPACK_FC=48.3 FF=0.0162 TF=0.	8	1	10	6	0 'C	y		76 / 100	CUUAUGUCUUUAUUGUG
-24.4 kcal	-	mutate C19 based on a R98 XOR submission...	10	1	12	5	0 'C	y		76 / 100	AUUUAUUCUUUAUUGUG
-22.4 kcal	-	Mod of 6492813_C82 _MUTATE_ONE_#NUPACK_S...	8	1	11	5	77 'C	y		75 / 100	AUUUAUUCUUUAUUGUG
-23.5 kcal	-	mutate U84 NuPACK_FC=37.18 FF=0.0155 TF=.	8	1	11	5	0 'C	y		75 / 100	CUUAUGUCUUUAUUGUG
-23 kcal	-	mutate A31 NuPACK_FC=48.61 FF=0.0142 TF=.	9	1	10	5	0 'C	y		75 / 100	CUUAUGUCUUUAUUGUG
-27.6 kcal	-	mutate U79 NuPACK_FC=34.9 FF=0.0083 TF=0.	13	1	12	4	0 'C	y		75 / 100	CUUAUCUCUUUAUUGUG
-22.4 kcal	-	Mod of 6492813_C82 _MUTATE_ONE_#NUPACK_S...	8	1	11	5	57 'C	y		74 / 100	AUUUAUUCUUUAUUGUG
-23.9 kcal	-	mutate G3 NuPACK_FC=34.98 FF=0.0132 TF=0.	11	1	10	5	0 'C	y		74 / 100	CUGAUCUCUUUAUUGUG
-29.5 kcal	-	mutate U29 NuPACK_FC=33.24 FF=0.0294 TF=.	13	1	12	3	0 'C	y		74 / 100	CUUAUCUCUUUAUUGUG
-27.8 kcal	-	mutate C19 NuPACK_FC=146.97 FF=0.0053 TF.	10	1	12	4	0 'C	y		74 / 100	AUUUAUUCUUUAUUGUG
-20.9 kcal	-	mutate A10 NuPACK_FC=58.82 FF=0.0108 TF=.	13	1	7	2	0 'C	y		73 / 100	CUUAUGUCUAUAUUGUG
-21.6 kcal	-	mutate G11 NuPACK_FC=27.65 FF=0.0232 TF=.	8	1	11	4	0 'C	y		73 / 100	CUUAUGUCUUGAUUGUG
-27.2 kcal	-	mutate G32 NuPACK_FC=80.15 FF=0.0074 TF=.	9	1	13	4	0 'C	y		73 / 100	AUUUAUUCUUUAUUGUG
-25.6 kcal	-	mutate G4 NuPACK_FC=32.82 FF=0.0112 TF=0.	10	1	8	6	0 'C	y		73 / 100	CUUGUCUCUUUAUUGUG
-26.7 kcal	-	mutate G12 NuPACK_FC=45.96 FF=0.0125 TF=.	13	1	11	3	0 'C	y		73 / 100	CUUAUCUCUUUGUUGUG
-29.5 kcal	-	mutate G4 NuPACK_FC=42.81 FF=0.0161 TF=0.	13	1	11	5	0 'C	y		73 / 100	CUUGUCUCUUUAUUGUG
-17.7 kcal	-	mutate C69 NuPACK_FC=99.22 FF=0.007 TF=0.	6	1	13	6	0 'C	y		72 / 100	AAUGAGGGACCUGUUUA
-24.4 kcal	-	mutate G2 NuPACK_FC=28.73 FF=0.017 TF=0...	11	1	9	5	0 'C	y		72 / 100	CGUAUCUCUUUAUUGUG
-28.5 kcal	-	mutate C9 NuPACK_FC=46.35 FF=0.0067 TF=0.	10	1	12	4	0 'C	y		71 / 100	AUUUAUUCCUUAUUGUG
-29.1 kcal	-	mutate U84 NuPACK_FC=26.63 FF=0.0188 TF=.	12	1	12	3	0 'C	y		71 / 100	AUUUAUUCCUUAUUGUG
-27.5 kcal	-	mutate C5 NuPACK_FC=40.23 FF=0.0148 TF=0.	13	1	11	4	0 'C	y		70 / 100	CUUACCUCUUUAUUGUG
-24.4 kcal	-	mutate C19 mutate C31 NuPACK_FC=2.14 FF=.	10	1	12	5	0 'C	y		70 / 100	AUUUAUUCUUUAUUGUG
-27 kcal	-	mutate A5 NuPACK_FC=38.1 FF=0.0187 TF=0...	12	1	11	5	0 'C	y		70 / 100	CUUAACUCUUUAUUGUG
-29.1 kcal	-	mutate A84 NuPACK_FC=28.1 FF=0.0264 TF=0.	12	1	12	3	0 'C	y		70 / 100	AUUUAUUCCUUAUUGUG
-20.9 kcal	-	mutate A67 NuPACK_FC=83.84 FF=0.0067 TF=.	13	1	7	2	0 'C	y		70 / 100	CUUAUGUCUUUAUUGUG
-22.4 kcal	-	Mod of 6492813_U85 _MUTATE_ONE_#NUPACK_S...	8	1	11	5	67 'C	y		69 / 100	AUUUAUUCUUUAUUGUG
-35.2 kcal	-	mutate C9 NuPACK_FC=90.94 FF=0.01 TF=0.9...	12	1	11	4	0 'C	y		65 / 100	AUUUAUUCCUUAUUGUG
-22.9 kcal	-	mutate C19 mutate C25 NuPACK_FC=2.03 FF=.	10	1	10	5	0 'C	y		65 / 100	AUUUAUUCUUUAUUGUG
-22.4 kcal	-	Mod of 6492813_C82 _MUTATE_ONE_#NUPACK_S...	8	1	11	5	77 'C	y		64 / 100	AUUUAUUCUUUAUUGUG
-29.6 kcal	-	mutate A75 NuPACK_FC=27.79 FF=0.0182 TF=.	12	1	13	2	0 'C	y		64 / 100	AUUUAUUCCUUAUUGUG
-27.6 kcal	-	mutate C9 NuPACK_FC=33.26 FF=0.0127 TF=0.	10	1	11	5	0 'C	y		64 / 100	AUUUAUUCCUUAUUGUG
-26.6 kcal	-	mutate C11 based on a R98 XOR submission...	11	1	12	4	0 'C	y		64 / 100	AUUUAUUCUUCAUUGUG
-23.2 kcal	-	mutate G6 NuPACK_FC=49.91 FF=0.0113 TF=0.	9	1	8	6	0 'C	y		63 / 100	CUUAUGUCUUUAUUGUG
-19.1 kcal	-	mutate C19 mutate U65 based ob R98:XOR:6..	7	1	10	6	67 'C	y		63 / 100	AUUUAUUCUUUAUUGUG
-25.6 kcal	-	mutate G83 NuPACK_FC=41.99 FF=0.0066 TF=.	12	1	11	3	0 'C	y		61 / 100	CUUAUCUCUUUAUUGUG
-29.1 kcal	-	mutate A43 NuPACK_FC=34.36 FF=0.0125 TF=.	12	1	12	3	0 'C	y		58 / 100	AUUUAUUCCUUAUUGUG
-28.3 kcal	-	mutate G2 NuPACK_FC=38.44 FF=0.0052 TF=0.	14	1	12	4	0 'C	y		55 / 100	CGUAUCUCUUUAUUGUG
-23.3 kcal	-	mutate U37 NuPACK_FC=36.49 FF=0.0195 TF=.	10	1	11	6	0 'C	y		48 / 100	AUUUAUUCUUUAUUGUG
-18.7 kcal	-	mod of 6486701;6130947_C82 #Mutate_One (	9	1	12	1	77 'C	y		48 / 100	GGGUGAUACGAGGGAAC
-28.2 kcal	-	mutate G12 NuPACK_FC=32.24 FF=0.028 TF=0.	12	1	11	2	0 'C	y		38 / 100	AUUUAUUCCUUGUUGUG
-27.6 kcal	-	mutate C9 NuPACK_FC=32.7 FF=0.0218 TF=0...	10	1	11	3	0 'C	y		37 / 100	AUUUAUUCCUUGUUGUG

Sorted after score and with the term mutate in description.

That even go for the pesky AND lab (rerun)

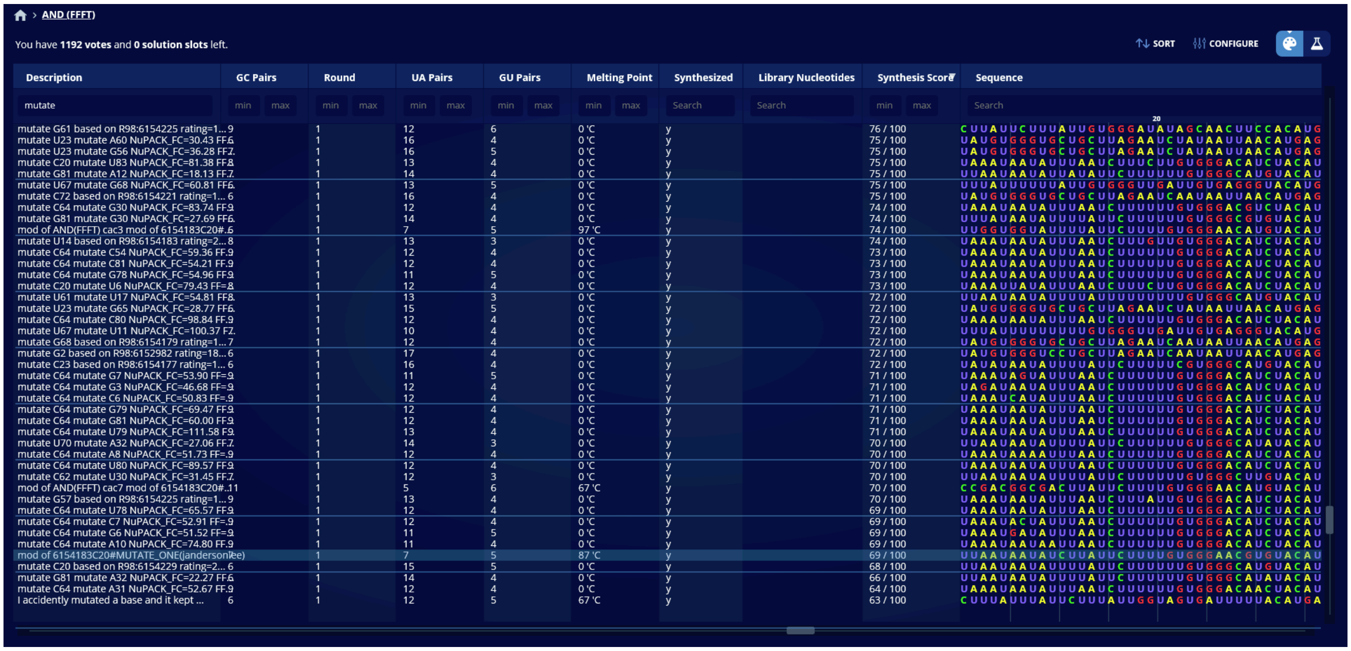

AND (FFFT)

You have **1192 votes** and **0 solution slots** left.

SORT CONFIGURE

Description	GC Pairs	Round	UA Pairs	GU Pairs	Melting Point	Synthesized	Library Nucleotides	Synthesis Score	Sequence
mutate	min max	min max	min max	min max	min max	Search	Search	min max	Search
mutate G61 based on R98:6154225 rating=1...	9	1	12	6	0 'C	y		76 / 100	CUUAUUCUUUAUUGUGGGAUAUAGCAACUUCCACAUG
mutate U23 mutate A60 NuPACK_FC=30.43 FF.	6	1	16	4	0 'C	y		75 / 100	UAUGUGGGUGCUGCUUAGAAUCUAUAAUUAACAUGAG
mutate U23 mutate G56 NuPACK_FC=36.28 FF.	7	1	16	5	0 'C	y		75 / 100	UAUGUGGGUGCUGCUUAGAAUCUAUAAUUAACAUGAG
mutate C20 mutate U83 NuPACK_FC=81.38 FF.	8	1	13	4	0 'C	y		75 / 100	UAAAUAAUAUUUAAUCUUUCUUGUGGGACAUCUACAU
mutate G81 mutate A12 NuPACK_FC=18.13 FF.	7	1	14	4	0 'C	y		75 / 100	UUAAUAAUAUUAUAUUCUUUUUUGUGGGCAUGUACAU
mutate U67 mutate G68 NuPACK_FC=60.81 FF.	6	1	13	5	0 'C	y		75 / 100	UUUAUUUUUUAUUGUGGGUUGAUUGUGAGGGUACAUG
mutate C72 based on R98:6154221 rating=1...	6	1	16	4	0 'C	y		75 / 100	UAUGUGGGUGCUGCUUAGAAUCAAUAAUUAACAUGAG
mutate C64 mutate G30 NuPACK_FC=83.74 FF.	9	1	12	4	0 'C	y		74 / 100	UAAAUAAUAUUUAAUCUUUUUUGUGGGACGUCUACAU
mutate G81 mutate G30 NuPACK_FC=27.69 FF.	6	1	14	4	0 'C	y		74 / 100	UUUAUAAUAUUUUAUUCUUUUUUGUGGGCGUGUACAU
mod of AND(FFFT) cac3 mod of 6154183C20#.	6	1	7	5	97 'C	y		74 / 100	UUGGUGGUAUUUUAUUCUUUUGUGGGAACAUGUACAU
mutate U14 based on R98:6154183 rating=2...	8	1	13	3	0 'C	y		74 / 100	UAAAUAAUAUUUAAUCUUUGUUGUGGGACAUCUACAU
mutate C64 mutate C54 NuPACK_FC=59.36 FF.	9	1	12	4	0 'C	y		73 / 100	UAAAUAAUAUUUAAUCUUUUUUGUGGGACAUCUACAU
mutate C64 mutate C81 NuPACK_FC=54.21 FF.	9	1	12	4	0 'C	y		73 / 100	UAAAUAAUAUUUAAUCUUUUUUGUGGGACAUCUACAU
mutate C64 mutate G78 NuPACK_FC=54.96 FF.	9	1	11	5	0 'C	y		73 / 100	UAAAUAAUAUUUAAUCUUUUUUGUGGGACAUCUACAU
mutate C20 mutate U6 NuPACK_FC=79.43 FF=.	8	1	12	4	0 'C	y		73 / 100	UAAAUUAUAUUUAAUCUUUCUUGUGGGACAUCUACAU
mutate U61 mutate U17 NuPACK_FC=54.81 FF.	8	1	13	3	0 'C	y		72 / 100	UUAAUAAUAUUUUAUUUUUUUUUGUGGGCAUGUACAU
mutate U23 mutate G65 NuPACK_FC=28.77 FF.	6	1	15	5	0 'C	y		72 / 100	UAUGUGGGUGCUGCUUAGAAUCUAUAAUUAACAUGAG
mutate C64 mutate C80 NuPACK_FC=98.84 FF.	9	1	12	4	0 'C	y		72 / 100	UAAAUAAUAUUUAAUCUUUUUUGUGGGACAUCUACAU
mutate U67 mutate U11 NuPACK_FC=100.37 F.	7	1	10	4	0 'C	y		72 / 100	UUUAUUUUUUUUUGUGGGUUGAUUGUGAGGGUACAUG
mutate G68 based on R98:6154179 rating=1...	7	1	12	4	0 'C	y		72 / 100	UAUGUGGGUGCUGCUUAGAAUCAAUAAUUAACAUGAG
mutate G2 based on R98:6152982 rating=18...	6	1	17	4	0 'C	y		72 / 100	UAUGUGGGUCCUGCUUAGAAUCAAUAAUUAACAUGAG
mutate C23 based on R98:6154177 rating=1...	6	1	16	4	0 'C	y		72 / 100	UAUAUAAUAUUUUAUUCUUUUUCGUGGGCAUGUACAU
mutate C64 mutate G7 NuPACK_FC=53.90 FF=.	9	1	11	5	0 'C	y		71 / 100	UAAAUAGUAUUUAAUCUUUUUUGUGGGACAUCUACAU
mutate C64 mutate G3 NuPACK_FC=46.68 FF=.	9	1	12	4	0 'C	y		71 / 100	UAGAUAAUAUUUAAUCUUUUUUGUGGGACAUCUACAU
mutate C64 mutate C6 NuPACK_FC=50.83 FF=.	9	1	12	4	0 'C	y		71 / 100	UAAAUCAUAUUUAAUCUUUUUUGUGGGACAUCUACAU
mutate C64 mutate G79 NuPACK_FC=69.47 FF.	9	1	12	4	0 'C	y		71 / 100	UAAAUAAUAUUUAAUCUUUUUUGUGGGACAUCUACAU
mutate C64 mutate G81 NuPACK_FC=60.00 FF.	9	1	12	4	0 'C	y		71 / 100	UAAAUAAUAUUUAAUCUUUUUUGUGGGACAUCUACAU
mutate C64 mutate U79 NuPACK_FC=111.58 F.	9	1	13	4	0 'C	y		71 / 100	UAAAUAAUAUUUAAUCUUUUUUGUGGGACAUCUACAU
mutate U70 mutate A32 NuPACK_FC=27.06 FF.	7	1	14	3	0 'C	y		70 / 100	UUAAUAAUAUUUUAUUCUUUUUUGUGGGCAUAUACAU
mutate C64 mutate A8 NuPACK_FC=51.73 FF=.	9	1	12	4	0 'C	y		70 / 100	UAAAUAAAAUUUAAUCUUUUUUGUGGGACAUCUACAU
mutate C64 mutate U80 NuPACK_FC=89.57 FF.	9	1	12	4	0 'C	y		70 / 100	UAAAUAAUAUUUAAUCUUUUUUGUGGGACAUCUACAU
mutate C62 mutate U30 NuPACK_FC=31.45 FF.	7	1	12	3	0 'C	y		70 / 100	UUAAUAAUAUUUUAUUCUUUUUUGUGGGCUUGUACAU
mod of AND(FFFT) cac7 mod of 6154183C20#.	11	1	5	6	67 'C	y		70 / 100	CCGACGGCGACUUAUUCUUUUGUGGGAACAUGUACAU
mutate G57 based on R98:6154225 rating=1...	9	1	13	4	0 'C	y		70 / 100	UAAAUAAUAUUUAAUCUUUAUUGUGGGACAUCUACAU
mutate C64 mutate U78 NuPACK_FC=65.57 FF.	9	1	12	4	0 'C	y		69 / 100	UAAAUAAUAUUUAAUCUUUUUUGUGGGACAUCUACAU
mutate C64 mutate C7 NuPACK_FC=52.91 FF=.	9	1	12	4	0 'C	y		69 / 100	UAAAUACUAUUUAAUCUUUUUUGUGGGACAUCUACAU
mutate C64 mutate G6 NuPACK_FC=51.52 FF=.	9	1	11	5	0 'C	y		69 / 100	UAAAUGAUAUUUAAUCUUUUUUGUGGGACAUCUACAU
mutate C64 mutate A10 NuPACK_FC=74.80 FF.	9	1	11	4	0 'C	y		69 / 100	UAAAUAAUAAUUAAUCUUUUUUGUGGGACAUCUACAU
mod of 6154183C20#MUTATE_ONE(jandersonee)	7	1	7	5	87 'C	y		69 / 100	UUAAUAAUAUCUUAUUCUUUUGUGGGAACGUGUACAU
mutate C20 based on R98:6154229 rating=2...	6	1	15	5	0 'C	y		68 / 100	UUAAUAAUAUUUUAUUCUUUUUUGUGGGCAUGUACAU
mutate G81 mutate A32 NuPACK_FC=22.27 FF.	6	1	14	4	0 'C	y		66 / 100	UUAAUAAUAUUUUAUUCUUUUUUGUGGGCAUAUACAU
mutate C64 mutate A31 NuPACK_FC=52.67 FF.	9	1	12	4	0 'C	y		64 / 100	UAAAUAAUAUUUAAUCUUUUUUGUGGGACAACUACAU
I accidently mutated a base and it kept ...	6	1	12	5	67 'C	y		63 / 100	CUUUAUUUAUUCUUUAUUGGUAGUGAUUUUUACAUGA

I noticed something else that I think is related. Here an example from the OR lab. I took note that two of the higher scoring designs weren't using the strongest possible pairing with the oligo. The other designs extend with full 3 G's.

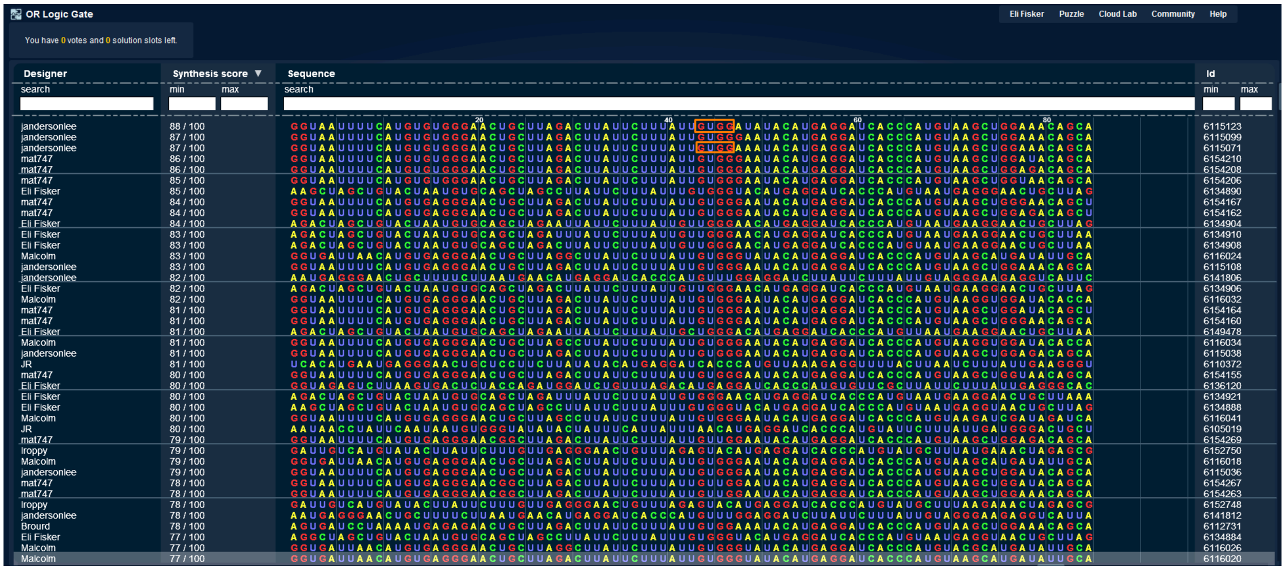

Figure 4: OR Lab Results

By the way, I found Johan's PDF for the 102 round which is a fine visual report of the lab data.

[Results for Eterna R102: Logic Gates]()

TTTF - NAND - 1/2 50%	Mutate one & two
TFFF - NOR - 3/3 100%	Mutate one & two
FTTF - XOR - 3/4 (rerun) 75%	Mutate one, two & four
FFFT - A AND B - 4/12 (rerun) 33%	Mutate one & two
FTTT - OR - 9/18 (rerun) 50%	Mutate one & two
TFFT - XNOR - 18/18 100%	Mutate one, two & four
TTFT - A OR NOT B - 21/23 91%	Mutate one & two
FTFF - A AND NOT B - most/120	Mutate one & two

I was attempting if I could see a pattern in which labs were the hard ones.

FFFT - A AND B - 4/12 (rerun) 33%
TTTF - NAND - 1/2 50%
FTTT - OR - 9/18 (rerun) 50%
FTTF - XOR - 3/4 (rerun) 75%
TTFT - A OR NOT B - 21/23 91%
FTFF - A AND NOT B - most/120
TFFF - NOR - 3/3 100%
TFFT - XNOR - 18/18 100%

Labs, where we have the original to the highest scorer

NOR
98% Mutate 2
https://eternagame.org/game/browse/6434625/?filter1=Id&filter1_arg1=6496405&filter1_arg2=6496405
Original: 76%
https://eternagame.org/game/browse/6434625/?filter1=Id&filter1_arg1=6474721&filter1_arg2=6474721

Strategies

Mismatches or GU's between oligos and sequence to aid a long oligo detach from the sequence are both strategies that will naturally get made with the mutation/evaluation strategy.

Mismatches between oligos and switch - allowing oligos to detach

One thing I found particularly interesting when I was reviewing my designs, was that in one case, just one base difference, resulted in a 10% score change.

No 1-1 loop between last oligo and RNA design, score 60%

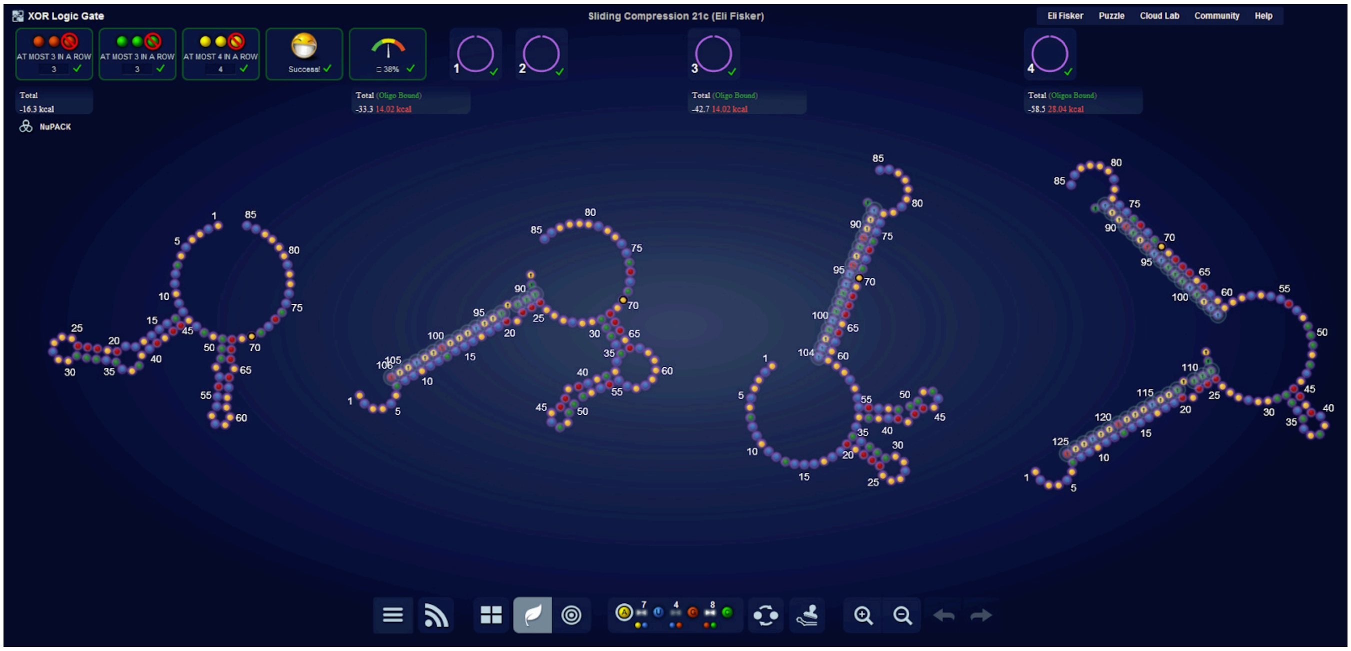

http://www.eternagame.org/game/browse/6096397/?filter1_arg1=6149320&filter1_arg2=6149320&filter1=Id

A 1-1 loop between last oligo and RNA design, score 70%

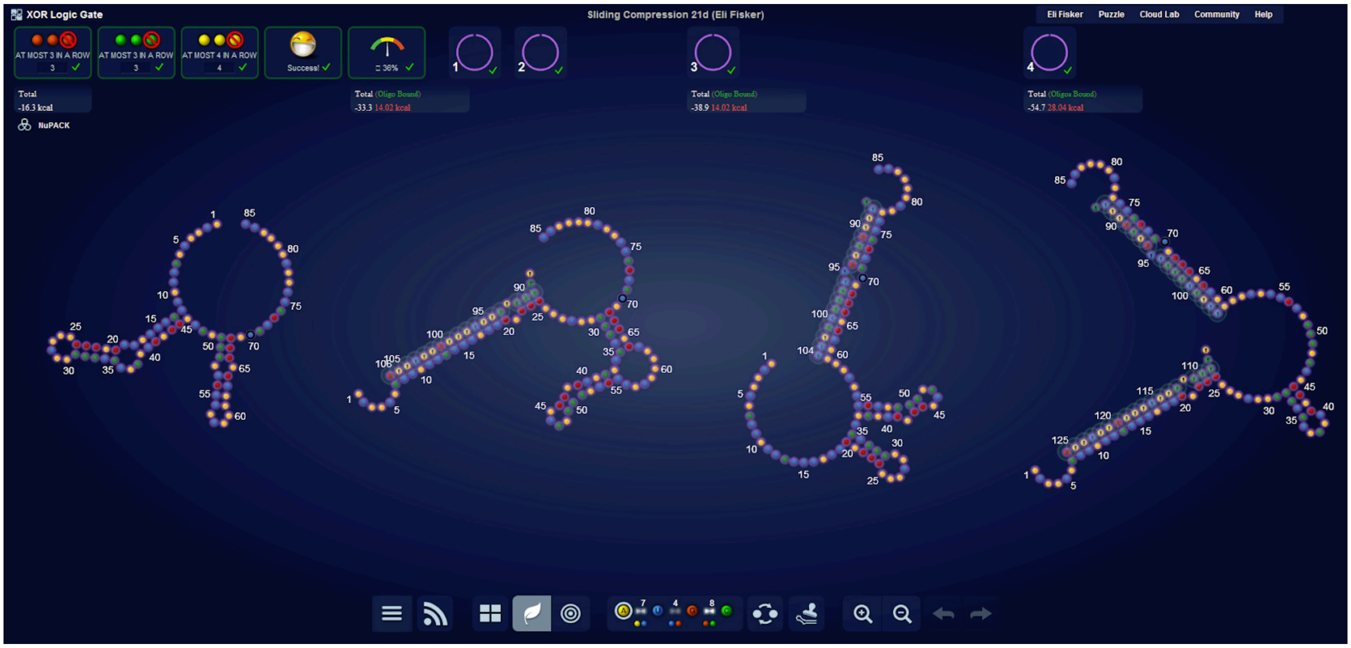

http://www.eternagame.org/game/browse/6096397/?filter1_arg1=6149327&filter1_arg2=6149327&filter1=Id

One or a few mismatches between a long oligo and sequence pairing have been most useful for aiding the switch between states.

R102 XOR:6468808 - 6136140_U79 #MUTATE_ONE

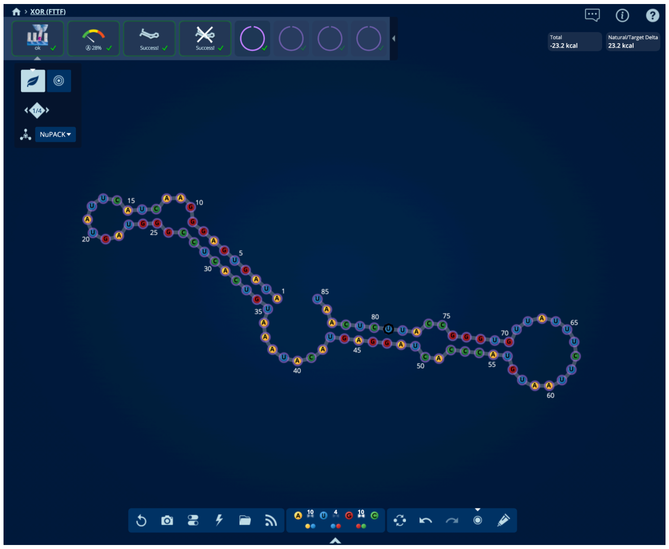

6136140_U79_FF

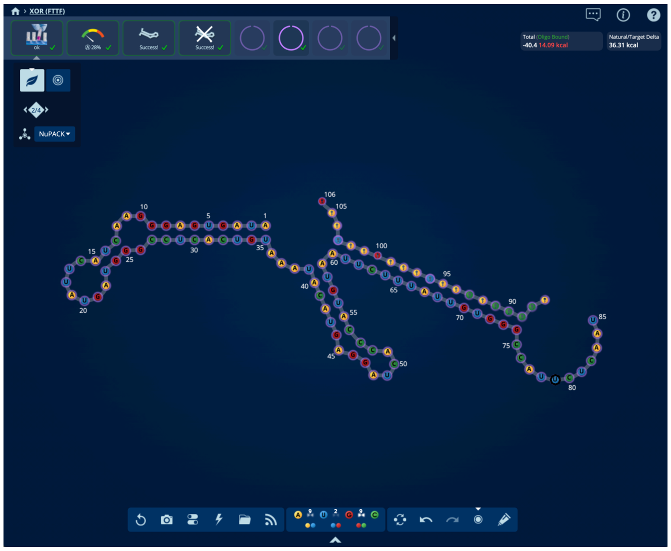

6136140_U79_TF

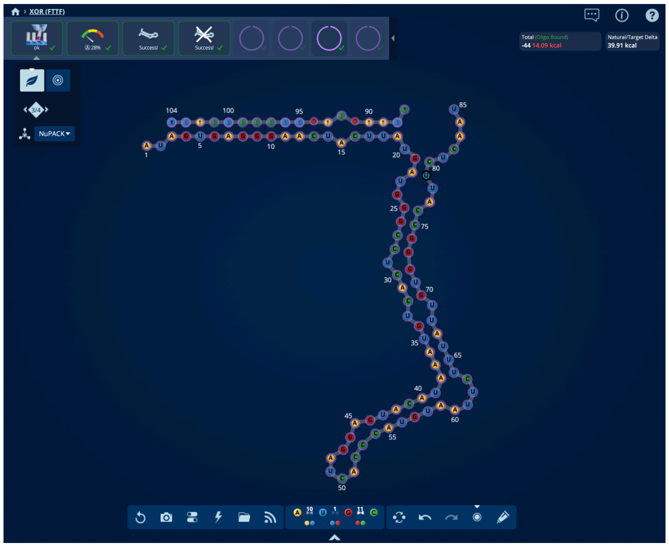

6136140_U79_FT

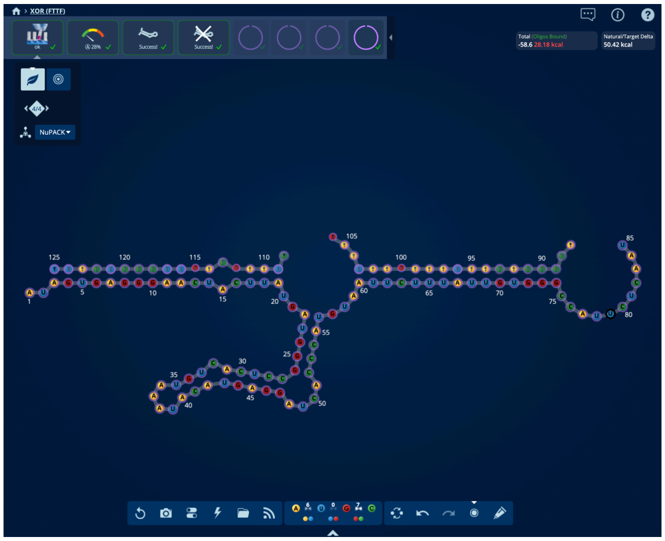

6136140_U79_TT

mutate U79 based on R98:6136140 FF=0.2430 TF=0.9608 FT=0.9946 TT=0.2817

This design mutated C79 to U79. In terms of the MFE shapes, this converted a GC pair to a GU in state 1 and changed the predicted free energy from -25.9 to -23.2, weakening the turnoff of the oglio A and MS2 binding sites in state 1. It also shifted the MFE structure in state 3, changing the free energy from -42.6 to -44.0 kcal thus strengthening the turn on of the MS2 aptamer in state 3. In state 1, both arms have open loops, increasing the ease for the stems to unwind and the shape to shift.

In terms of the pairing probabilities, the profile has the right general shape but although the ON states are strong at 96% and 99%, the OFF states are still high at 24% and 28%. The lab score was 91.

R102 XOR:6471802 - 6136140_U79_U76 #MUTATE_TWO

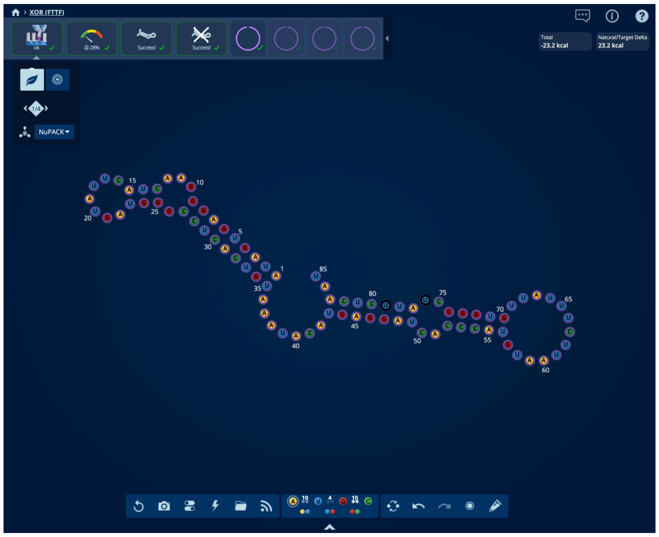

6136140_U79_U76_FF

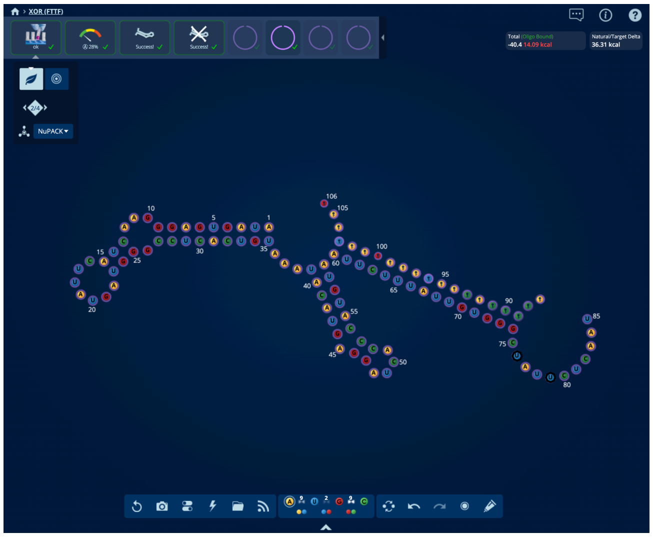

6136140_U79_U76_TF

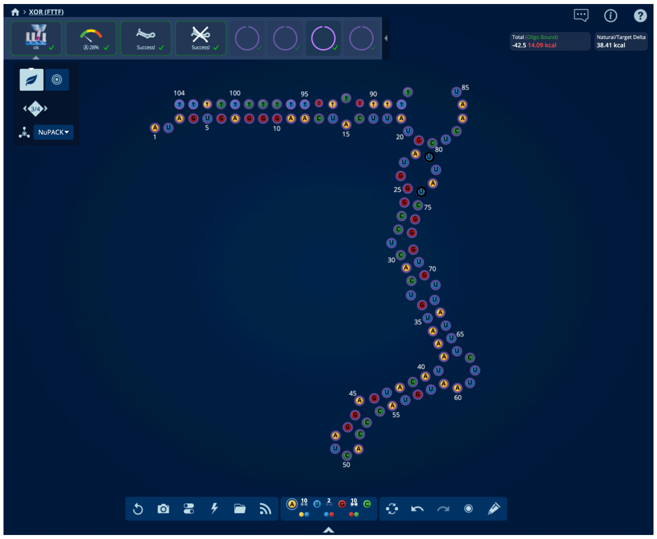

6136140_U79_U76_FT

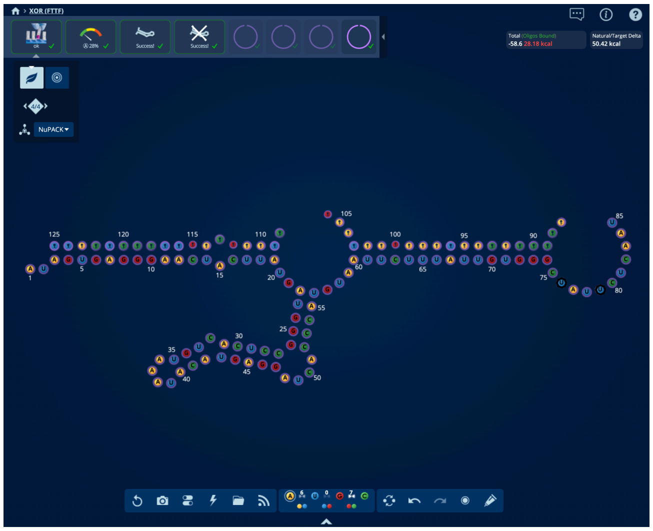

6136140_U79_U76_TT

mutate U79 mutate U76 based on R98:6136140 FF=0.0285 TF=0.9626 FT=0.9873 TT=0.0377

This design mutated C79 to U79 and a C76 to U76. This converted a GC pair to a GU in state 1 and changed the predicted free energy from -25.9 to -23.2, weakening the turnoff of the oglio A and MS2 binding sites in state 1. It also shifted the MFE structure and converted a GC to GU in state 3, changing the free energy from -42.6 to -42.5 kcal thus reverting the change to the MS2 aptamer turn on caused by the single C79 to U79 change in the previous design. In state 1, both arms have open loops, increasing the ease for the stems to unwind and the shape to shift.

In terms of the pairing probabilities, the profile has the right shape and a good profile with the ON states strong at 96% and 99% and the OFF states are low at 3% and 4%. The lab score was 98.

Forum posts:
[1 base difference - 10% score difference](),
[Mismatches between oligo and complement]()

Crossing GU's - allowing oligos to detach

A strategy similar to mismatches, also worked well. Crossed GU's would help weaken the bond between oligo and RNA sequence. If a pairing between an oligo and the RNA sequence becomes really long, due to hydrogen bonding it gets harder for the oligo to also fall off. This strategy showed its full strength in the OpenTB lab.

Score 98%, fc (fold change) 58, fc error (fold change error) 1.26

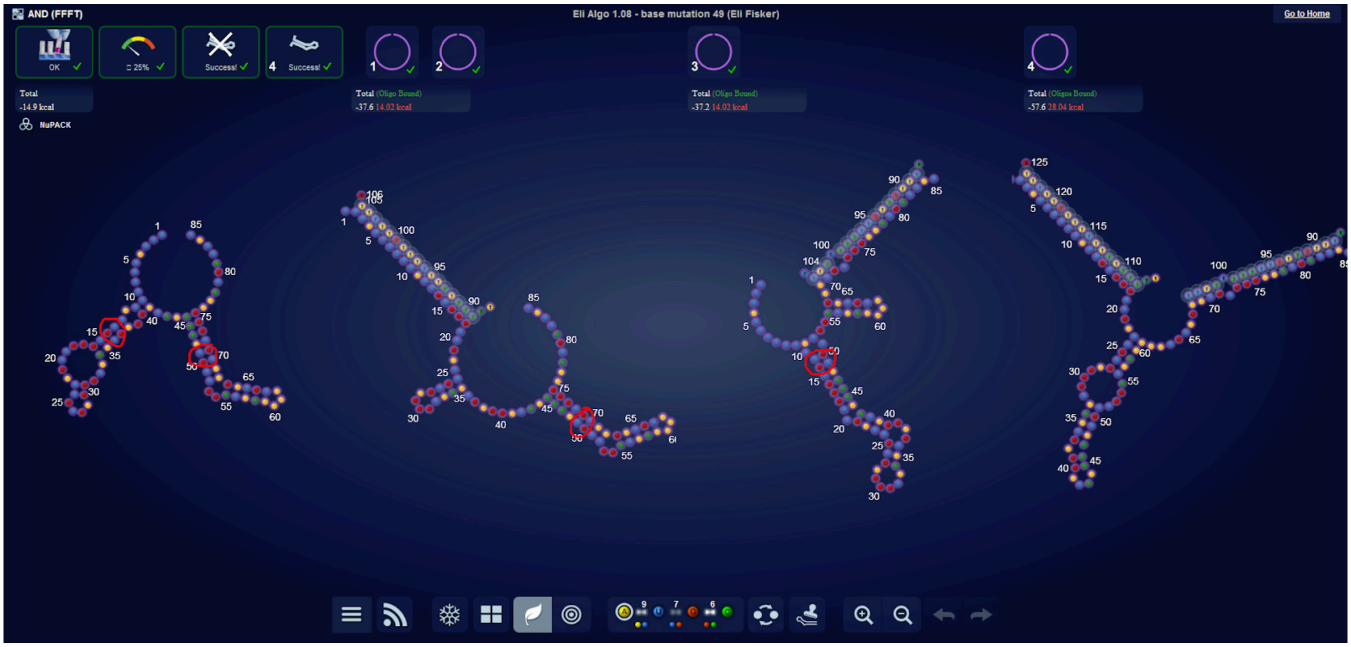

https://eternagame.org/game/browse/6434647/?filter1=Id&filter1_arg1=6466266&filter1_arg2=6466266

Forum posts:
Crossed GU's are now legit,
Use of GU in two input labs

Difference in sequence length use for ON and OFF switches

ON switches tend to not need the full sequence, but often allow throwing unused bases into a static stem in one or both ends of the design. While OFF switches are notorious for using most or all of the bases. All our Logic gates and the main part of the OpenTB lab fits this pattern. I bet our hardest OpenTB OFF switch could use some more sequence space.

Here are two partner lab examples:

XNOR - ON switch - Long static stem for hiding away bases

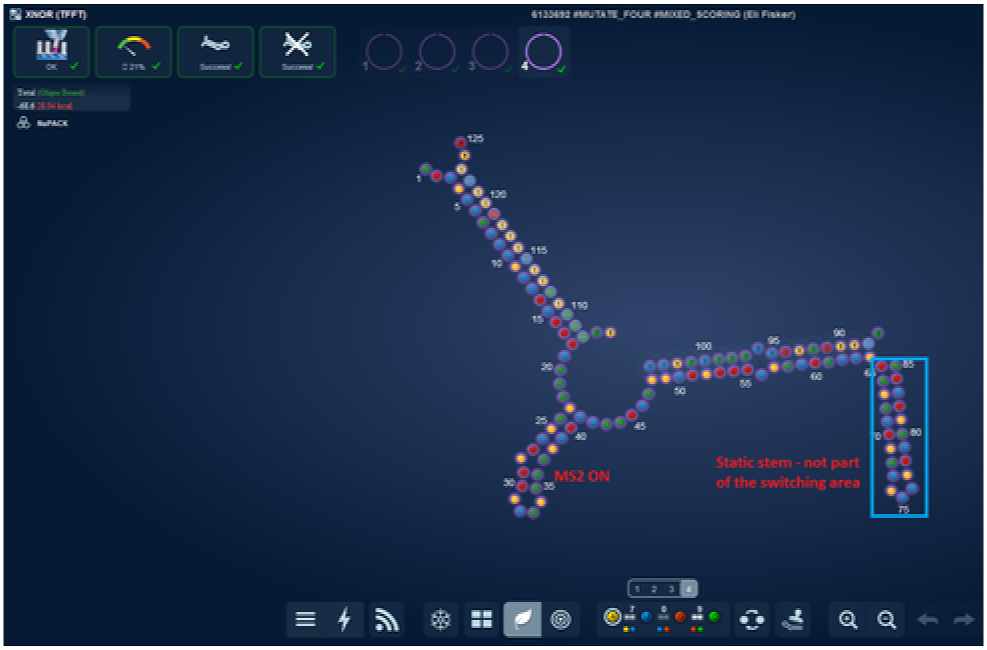

https://eternagame.org/game/browse/6434648/?filter1=Id&filter1_arg1=6501007&filter1_arg2=6501007

XOR - OFF switch - use of full sequence

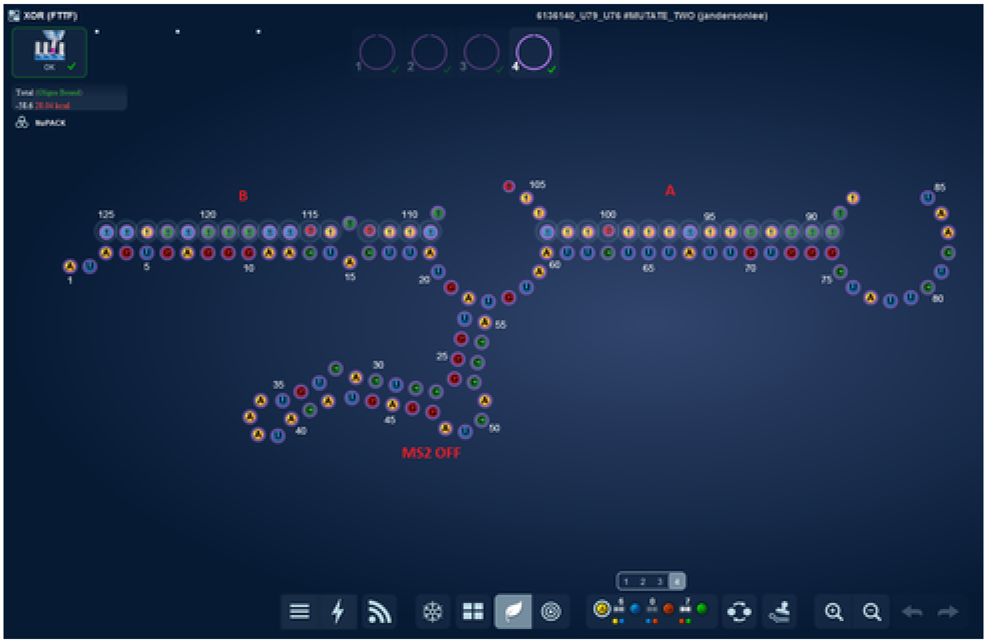

https://eternagame.org/game/browse/6434627/?filter1=Id&filter1_arg1=6471802&filter1_arg2=6471802

ON switches being shorter than OFF switches, in particular happens in puzzles with two or more inputs.

Forum post:
Difference in length for ON and OFF switches

Reversal of strong and weak oligo input for ON and OFF switches

To be summarized yet

Quantum entanglement in partner labs

Mirror entanglement still happening

Input order - depends on if it is an ON switch or an OFF switch

Strength of input affect how distanced the inputs will land in the RNA design

References

[1] Lee et al., 2014, J. Lee, W. Kladwang, et al., “RNA design rules from a massive open laboratory”, Proc. Natl. Acad. Sci. (2014), 10.1073/pnas.1313039111

[2] Emmanuelle Querido, Pascal Chartrand, “Using fluorescent proteins to study mRNA trafficking in living cells”, PMID: 18155467, DOI: 10.1016/S0091-679X(08)85012-1

[3] Joseph N. Zadeh, Conrad D. Steenberg, Justin S. Bois, Brian R. Wolfe, Marshall B. Pierce, Asif R. Khan, Robert M. Dirks, Niles A. Pierce, “NUPACK: Analysis and design of nucleic acid systems”, 17 November 2010, https://doi.org/10.1002/jcc.21596

[4] Timothy E Sweeney, Lindsay Braviak, Cristina M Tato, Purvesh Khatri, “Genome-wide expression for diagnosis of pulmonary tuberculosis: a multicohort analysis”, Lancet Respir Med. 2016 Mar;4(3):213-24. doi: 10.1016/S2213-2600(16)00048-5. Epub 2016 Feb 20, PMID: 26907218, PMCID: PMC4838193, DOI: 10.1016/S2213-2600(16)00048-5

Finding a balance for [A] times [B] over [C] squared designs.

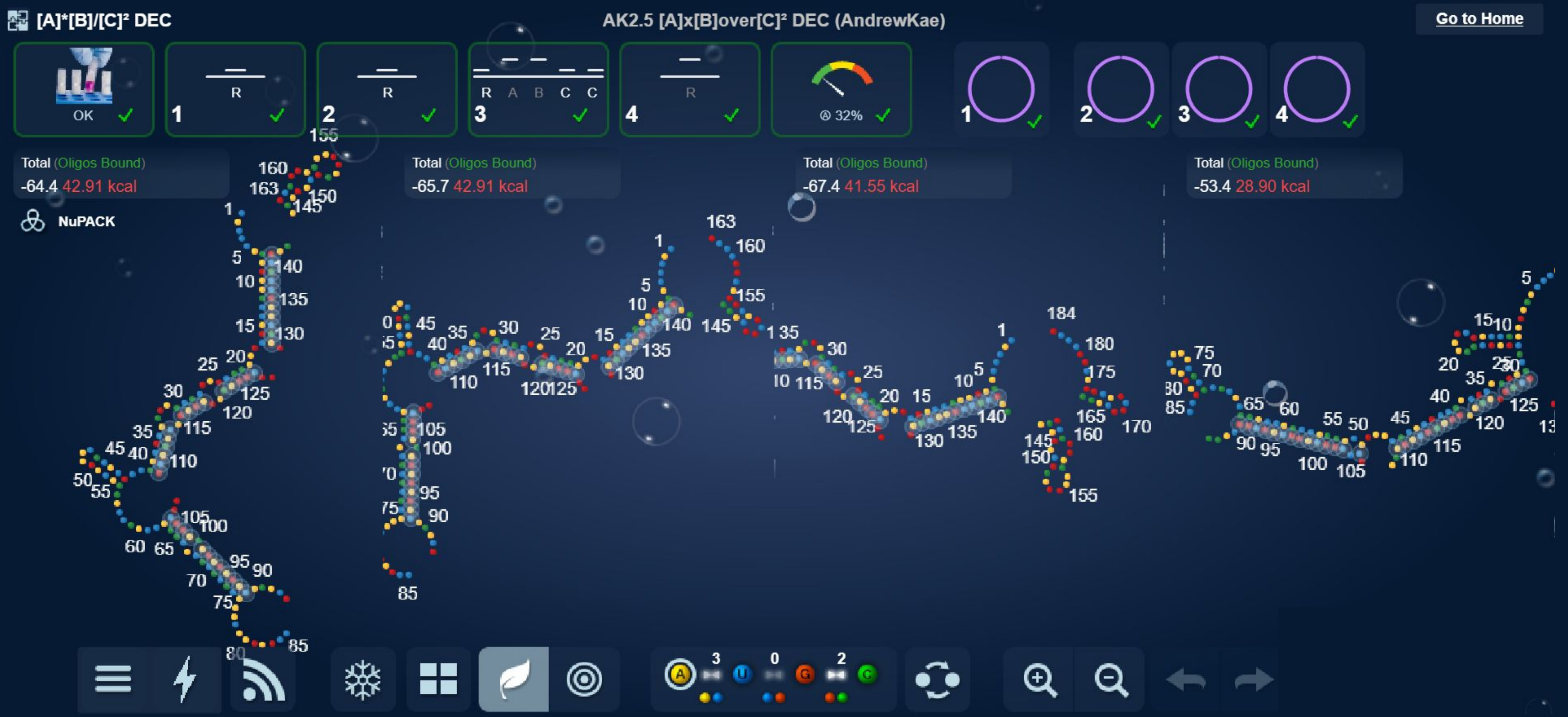

Andrew Kaechele

OpenTB Round 2 - [A]x[B]/[C]^2 - AK2.5

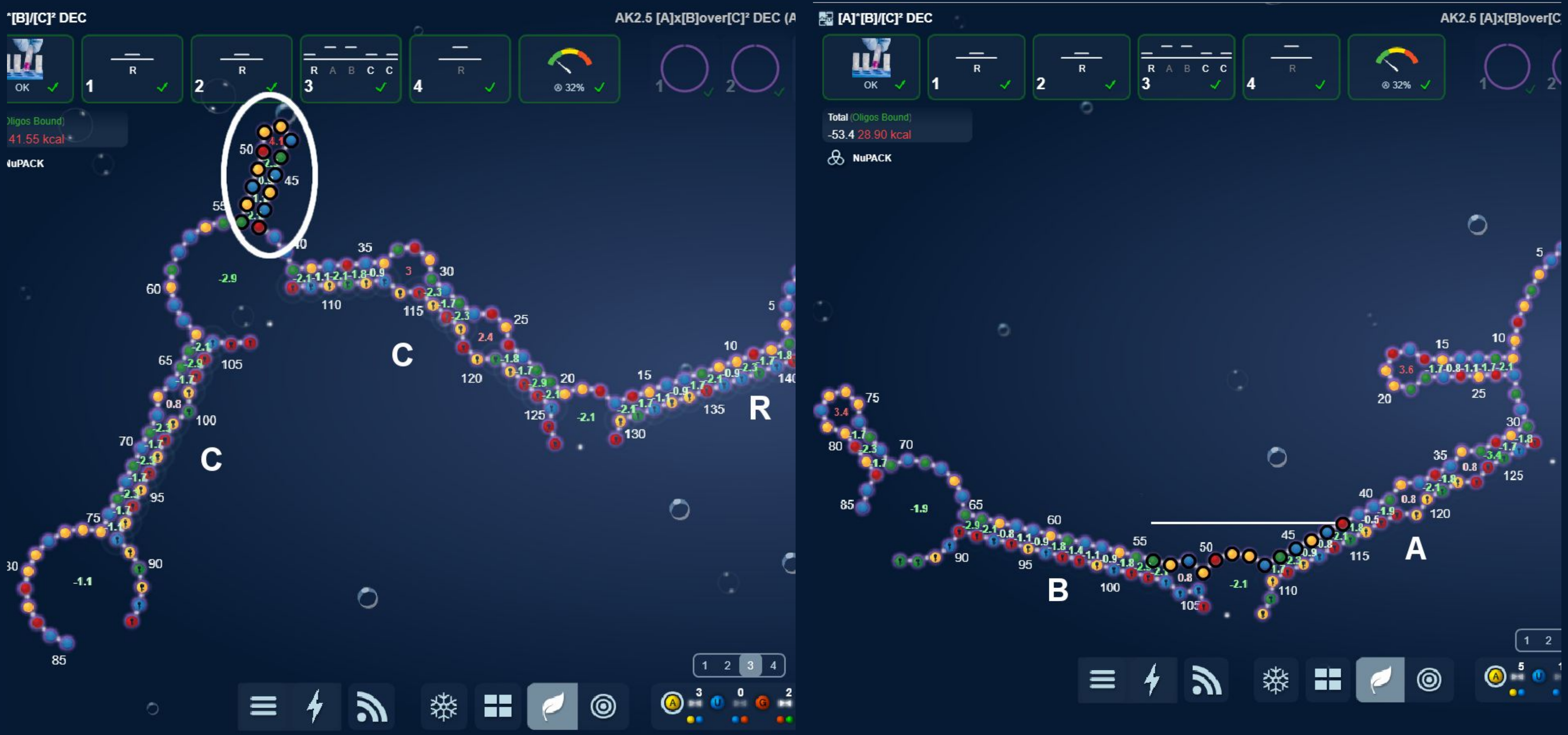

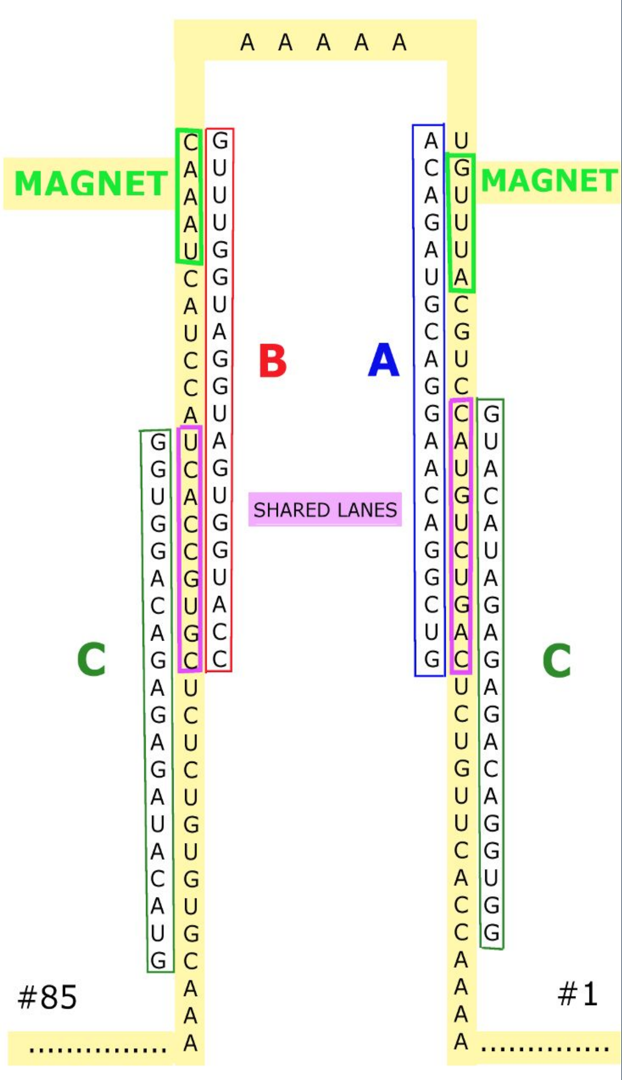
A A A A A
MAGNET
MAGNET
B
A
SHARED LANES
C
C
#85
#1
CAAAUCAUCCAUCACCGUGCUCUCUGUGUGCAAA
GUUUGGUAGGUAGUGGUACC
GGUGGACAGAGAGAUACAUG
ACAGAUGCAGGAACAGGCUG
UGUUUACGUCCAUGUCUGACUCUGUUCACCAAAA
GUACAUAGAGAGACAGGUGG

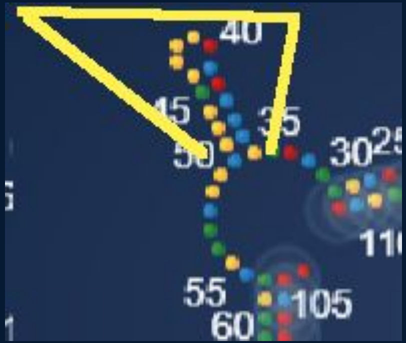
40
45
35
30
25
50
55
105
60

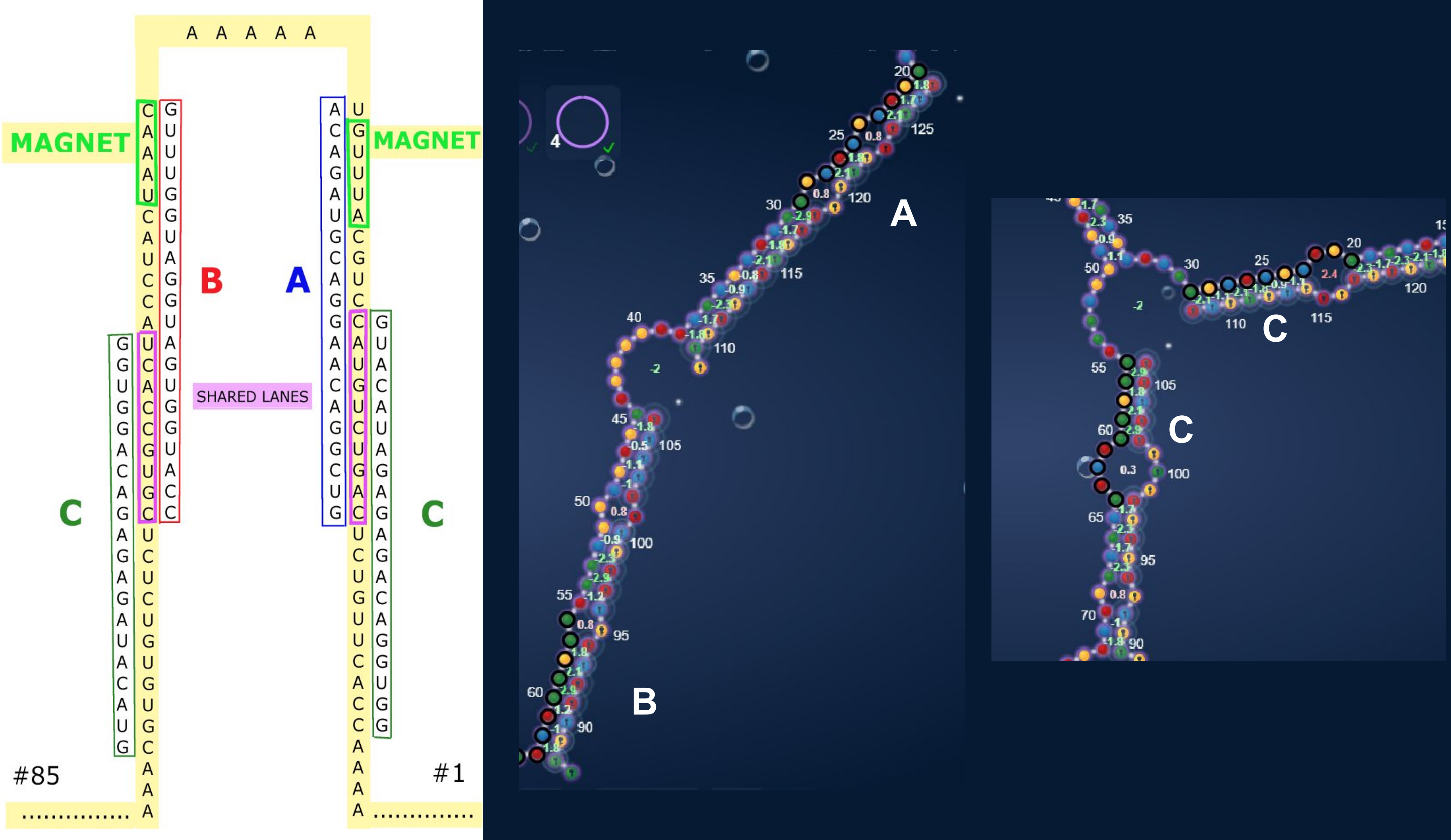

A A A A A
MAGNET
MAGNET
B
A
SHARED LANES
C
C
#85
#1
A
B
C
C

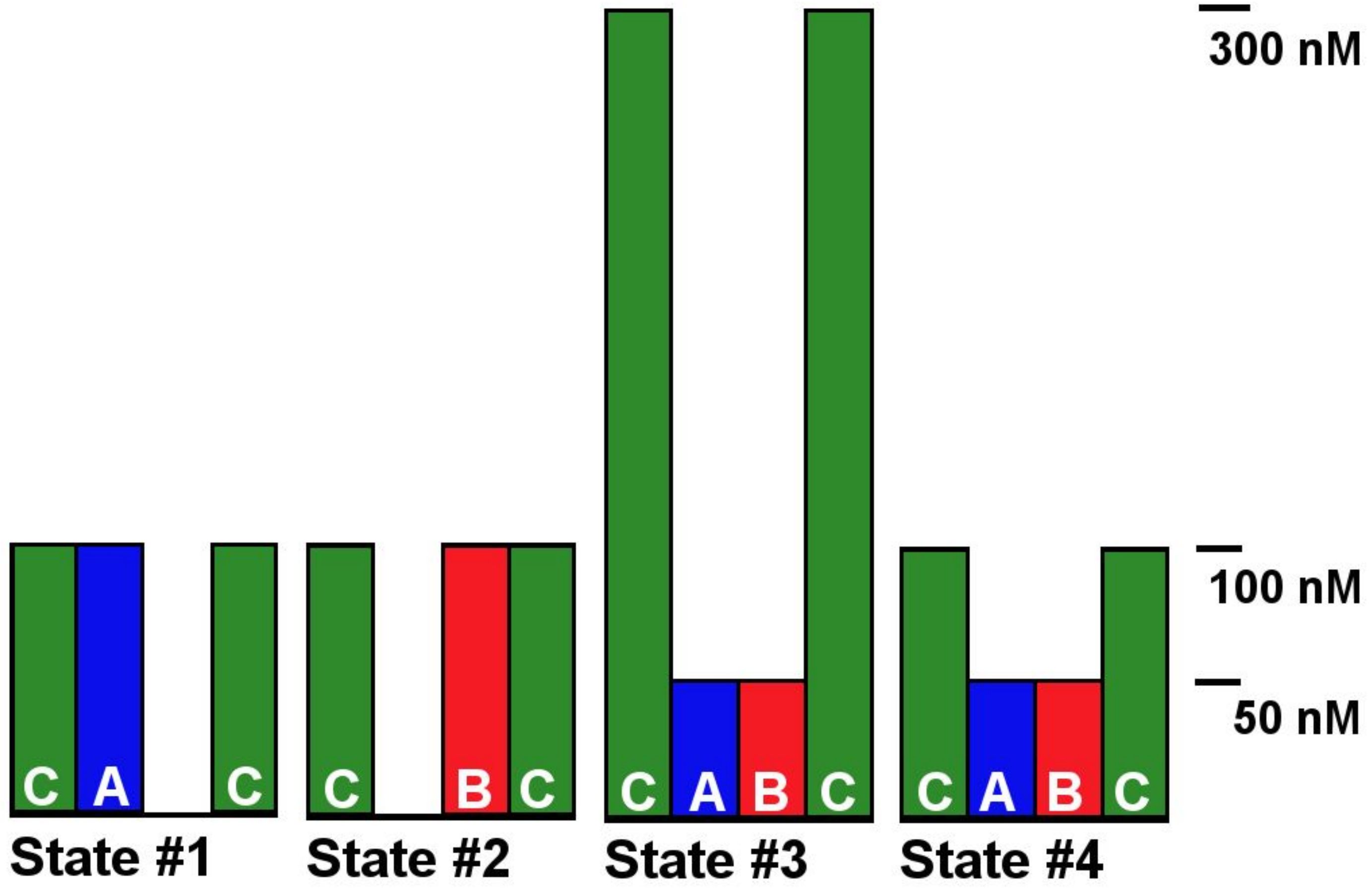
300 nM
100 nM
50 nM
C
A
C
State #1
C
B
C
State #2
C
A
B
C
State #3
C
A
B
C
State #4

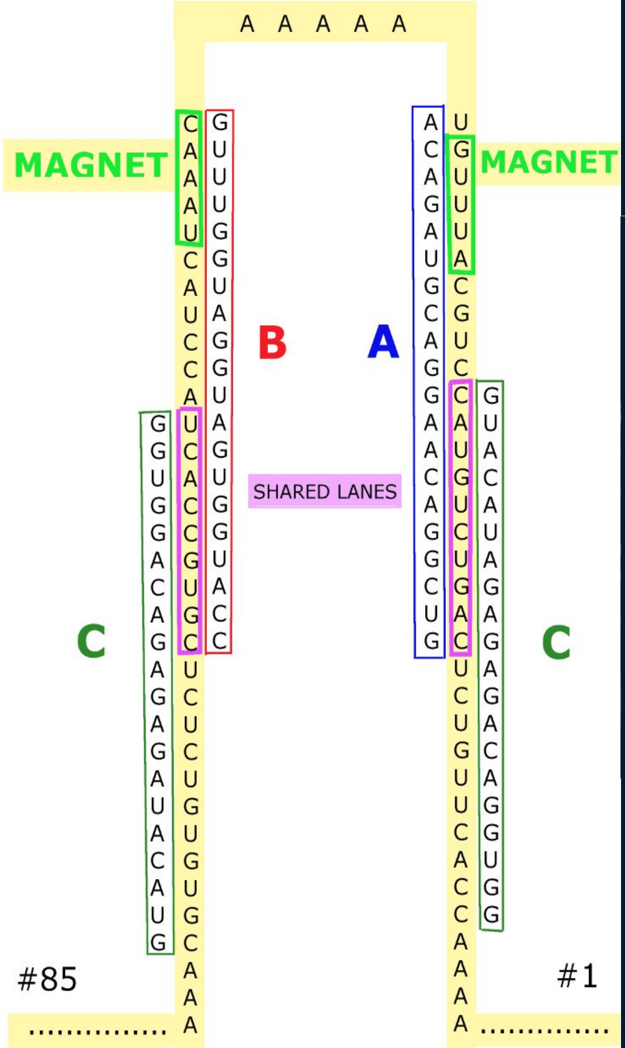
A A A A A
MAGNET
MAGNET
B
A
SHARED LANES
C
C
#85
#1

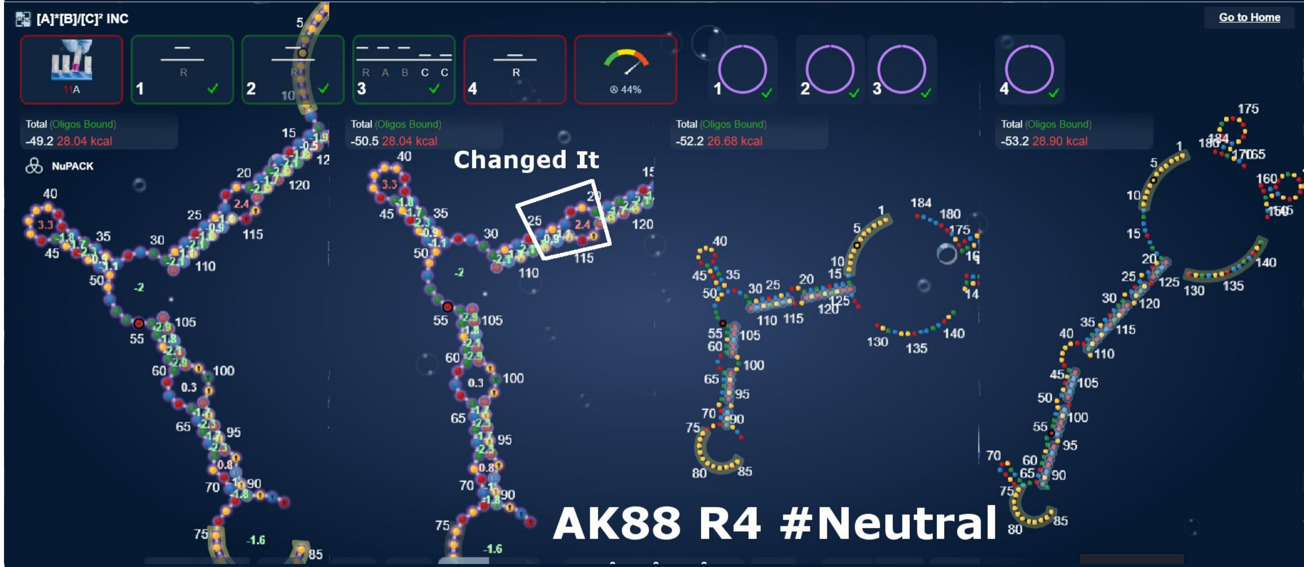
[A]*[B]/[C]² INC
Go to Home
Total (Oligos Bound)
-49.2 28.04 kcal
-50.5 28.04 kcal
-52.2 26.68 kcal
-53.2 28.90 kcal
NuPACK
Changed It
AK88 R4 #Neutral

AAAAAAAAACCACUUGUCUCAGUCUGUACCUGCAUUUGUAAAAACAAAUCAUCCAUCACCGUGCUCU
CUGUGUGCAAAAAAAAAA

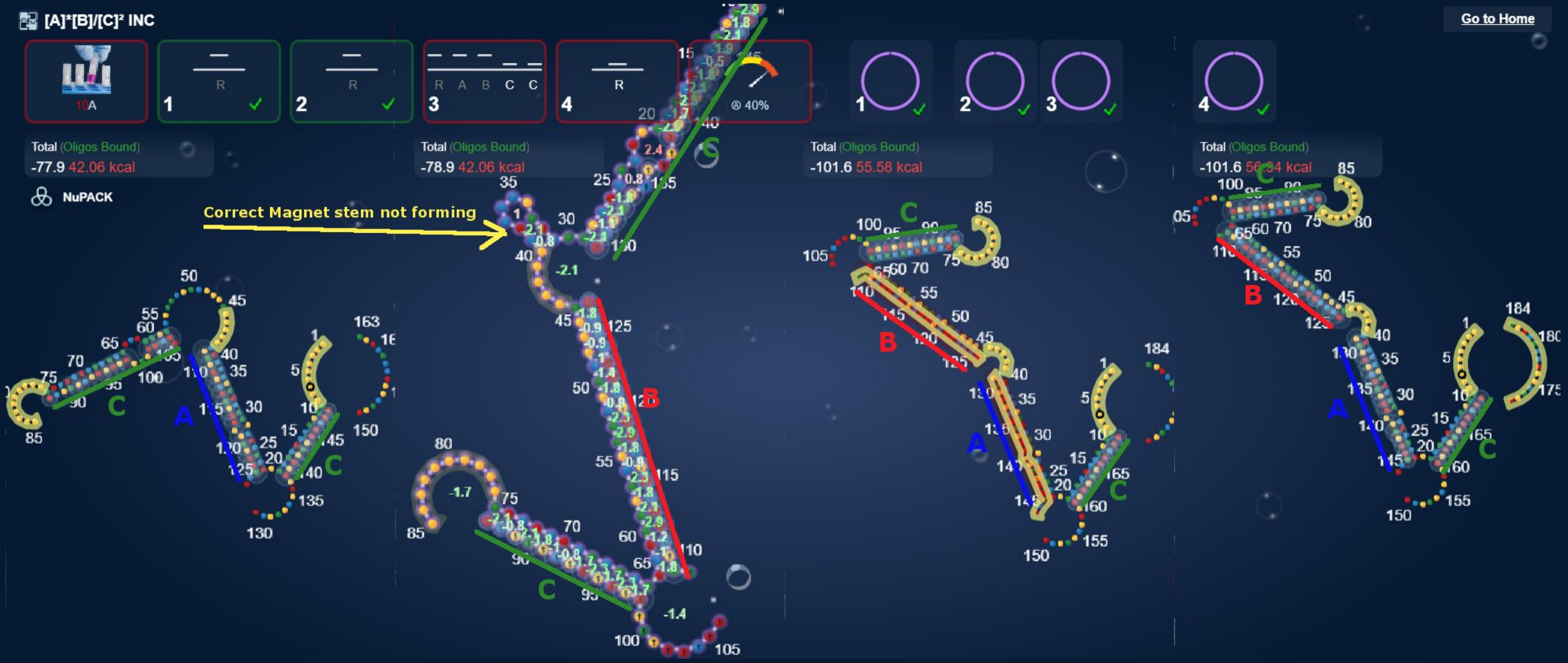

AAAAAAAAACCACUUGUCUCAGUCUGUACCUGCAUUUGUGAAAACAAAUAAUCCAUCACCGUGCUCU
CUGUGUGCAAAAAAAAAA

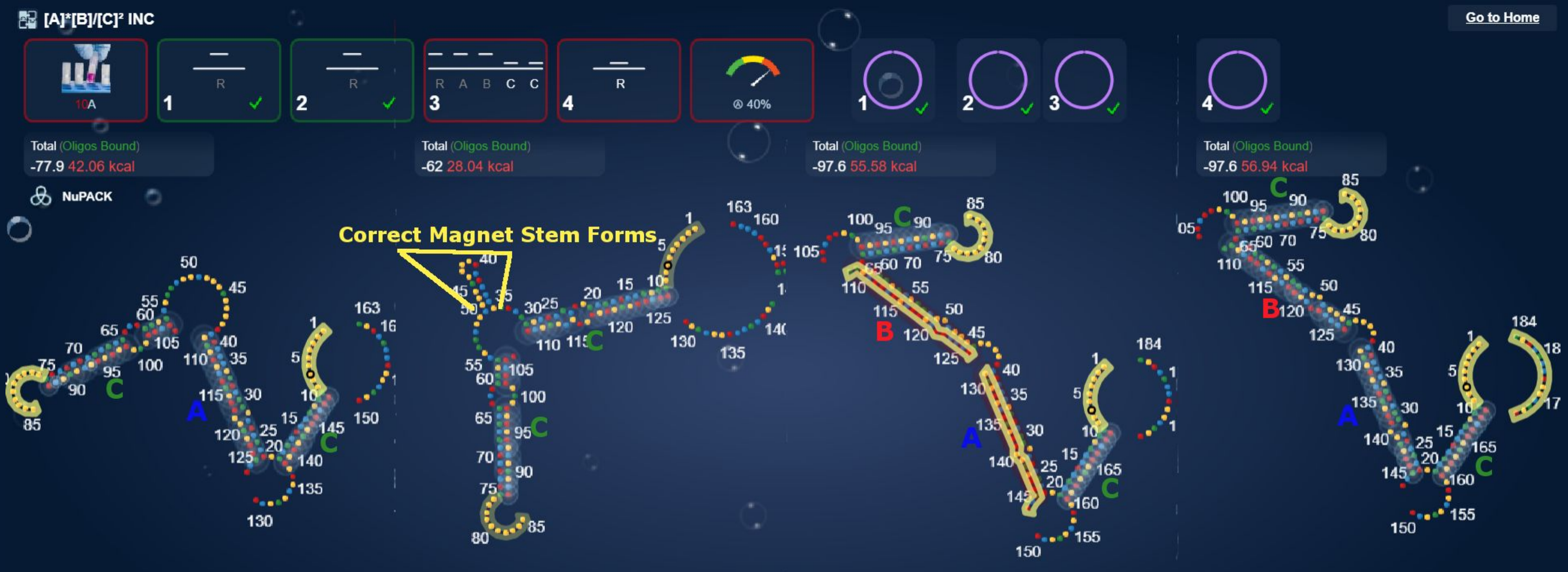

AAAAAAAAACCACUUGUCUCAGUCUGUACCUGUAUUUGUGAAAACAAAUAAUCCAUCACCGUGCUCUCUGUGUGCAAAAAAAAAA

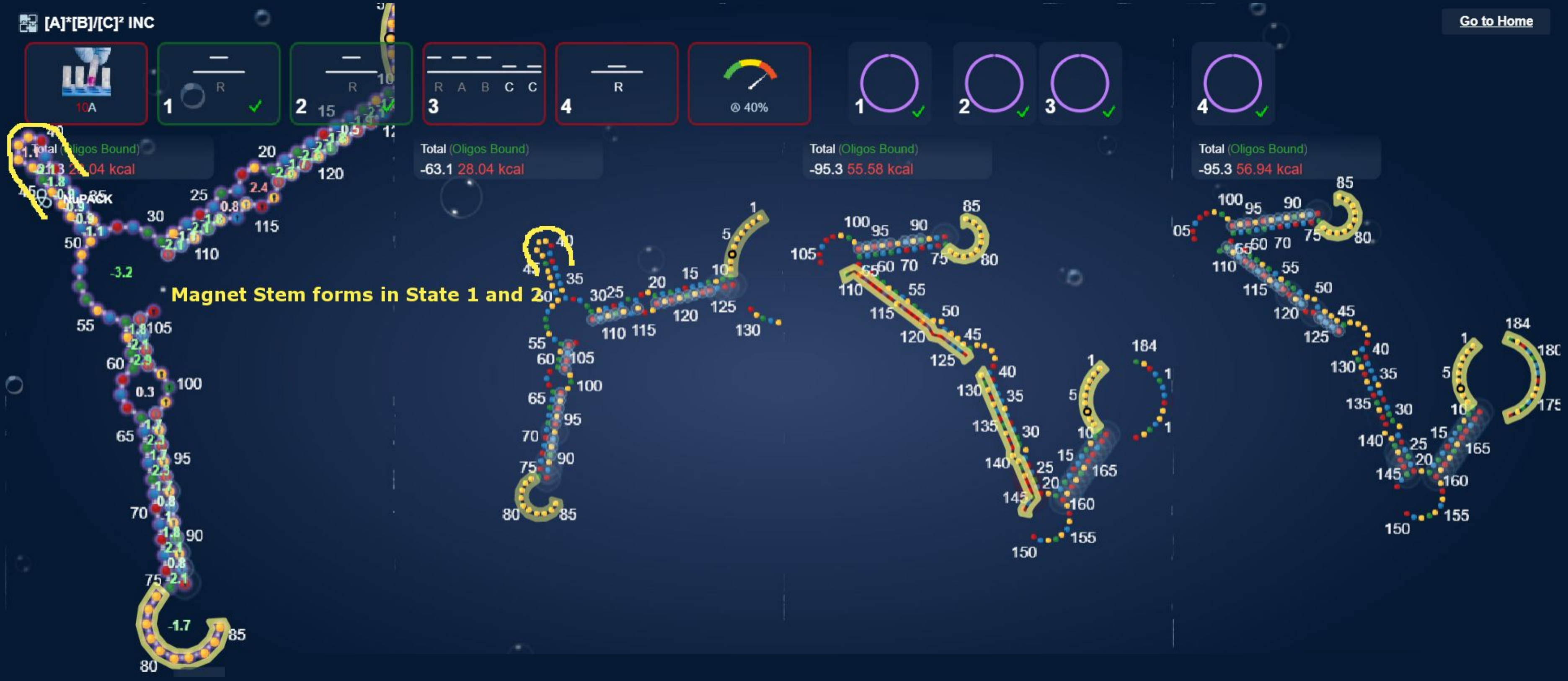

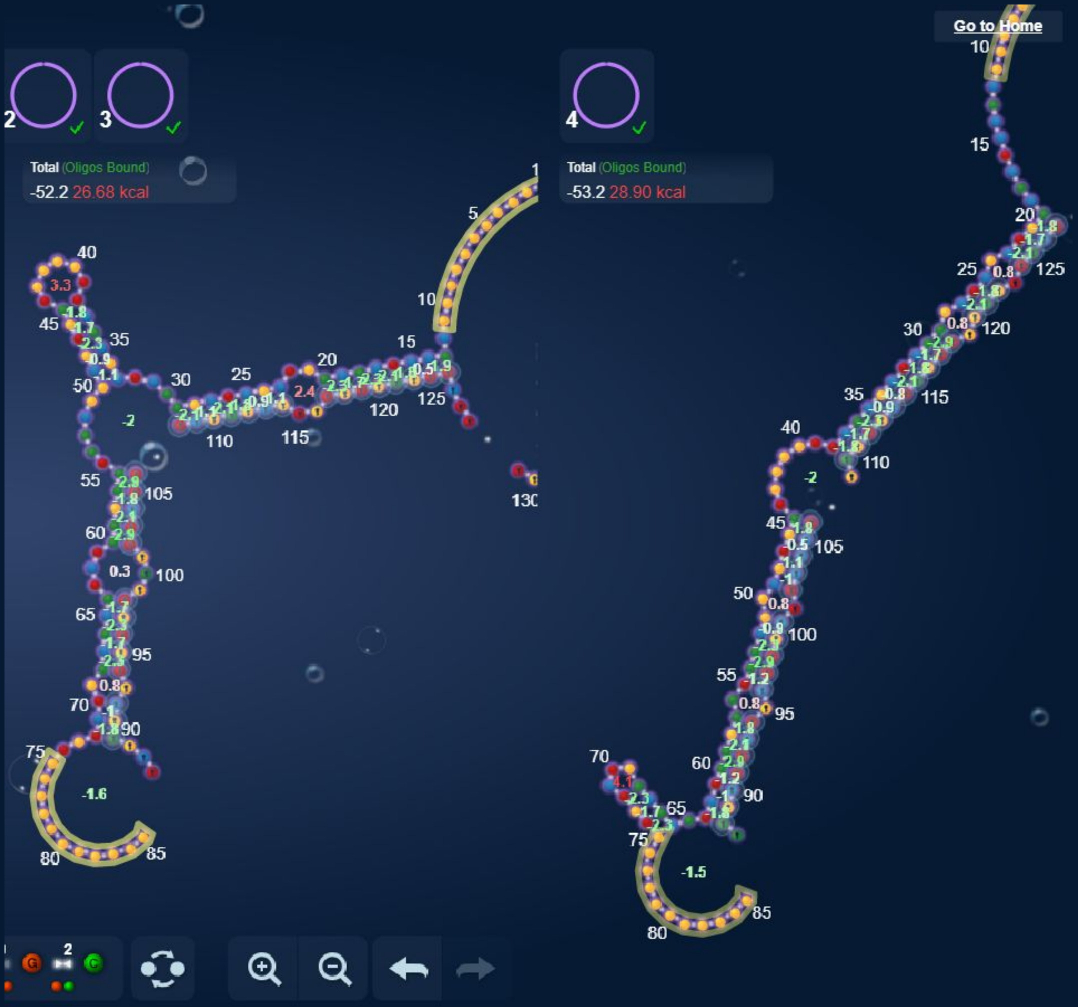

Total (Oligos Bound)
-52.2 26.68 kcal
Go to Home
Total (Oligos Bound)
-53.2 28.90 kcal

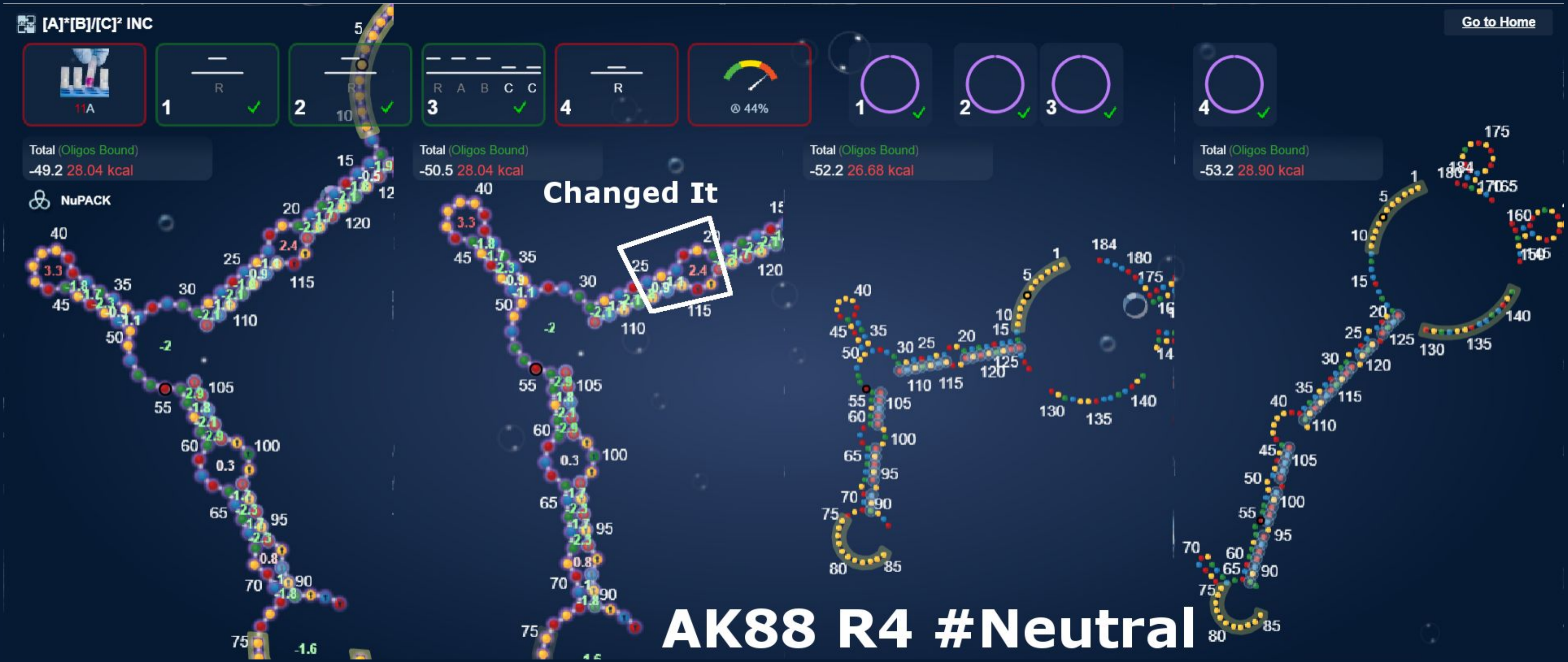
[A]*[B]/[C]² INC
Go to Home
11A
1
2
3
4
R
R A B C C
44%
Total (Oligos Bound)
-49.2 28.04 kcal
NuPACK
Total (Oligos Bound)
-50.5 28.04 kcal
Changed It
Total (Oligos Bound)
-52.2 26.68 kcal
Total (Oligos Bound)
-53.2 28.90 kcal
AK88 R4 #Neutral

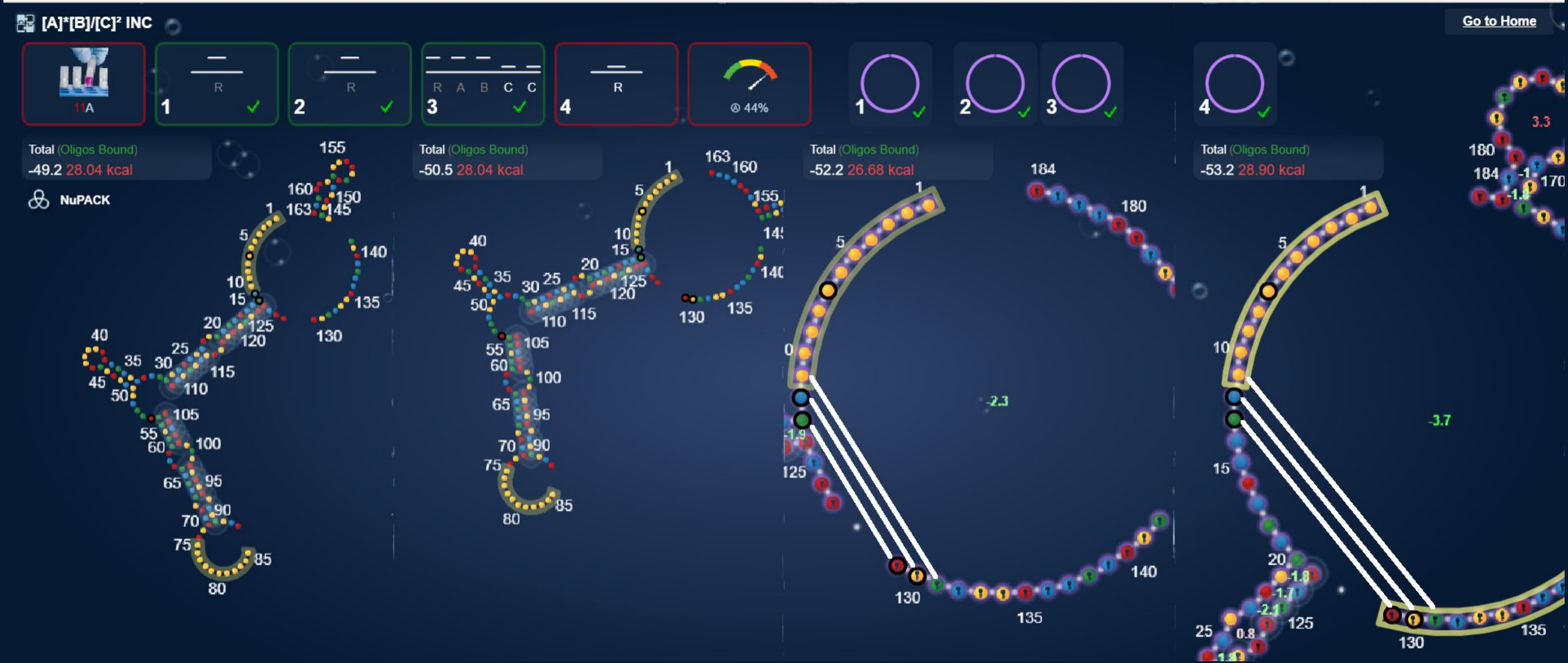
[A]*[B]/[C]² INC
Go to Home
11A
R
R
R A B C C
R
44%
Total (Oligos Bound)
-49.2 28.04 kcal
NuPACK
Total (Oligos Bound)
-50.5 28.04 kcal
Total (Oligos Bound)
-52.2 26.68 kcal
Total (Oligos Bound)
-53.2 28.90 kcal

AAAAAAAAGUCAGAACUUAGUCUUGUCUCAGUAUGUACCUGUAUCUGGAAAAGCAGAUAAUCCGCCA
CCGUGCUCUCAGUGAGAA

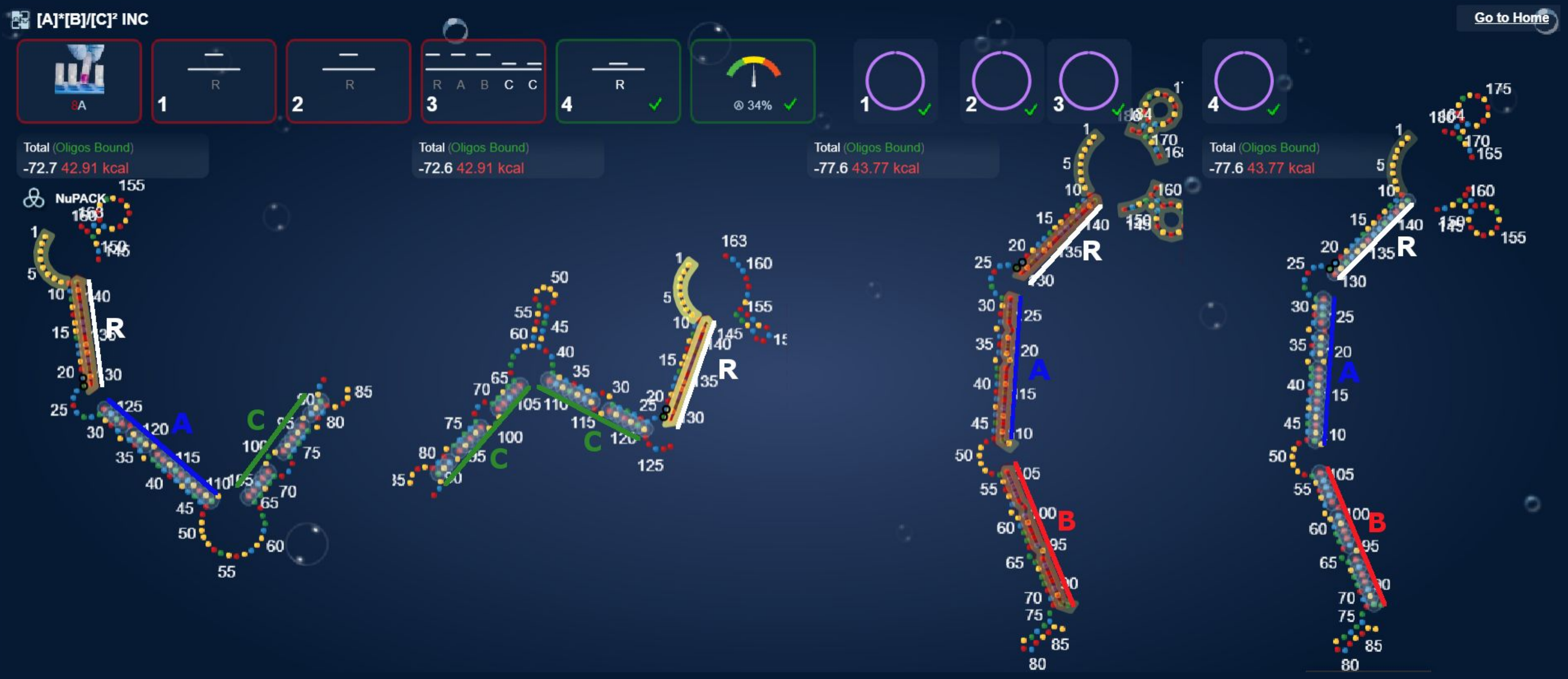

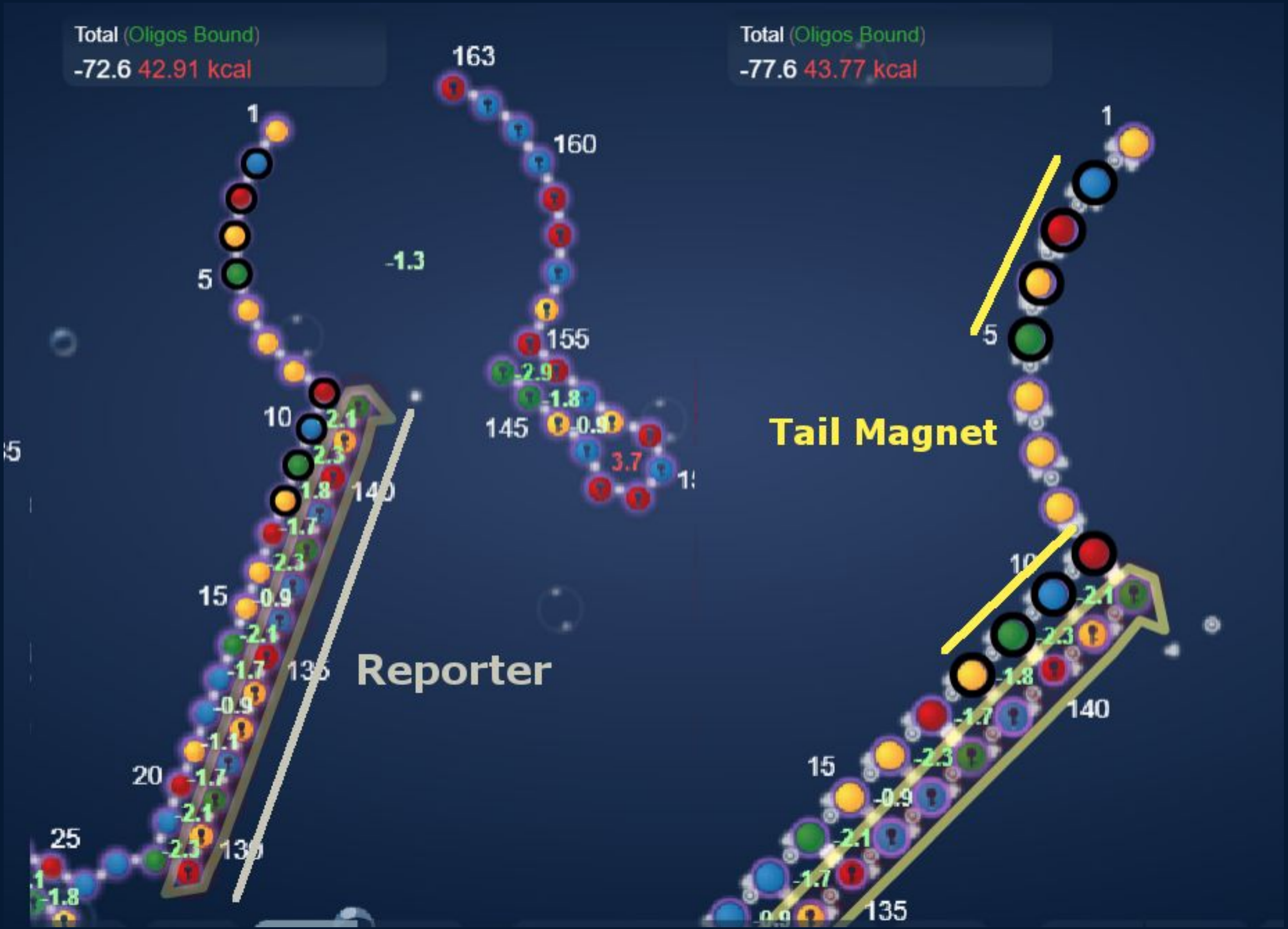
Total (Oligos Bound)
-72.6 42.91 kcal
Reporter
Total (Oligos Bound)
-77.6 43.77 kcal
Tail Magnet

AUGACAAAGUCAGUAUUUAGUCUUGUCUCAGUAUGUACCUGUAUCUGGAAAAGCAGAUAAUCCGCCAC
CGUGCUCUCAGUGAGAA

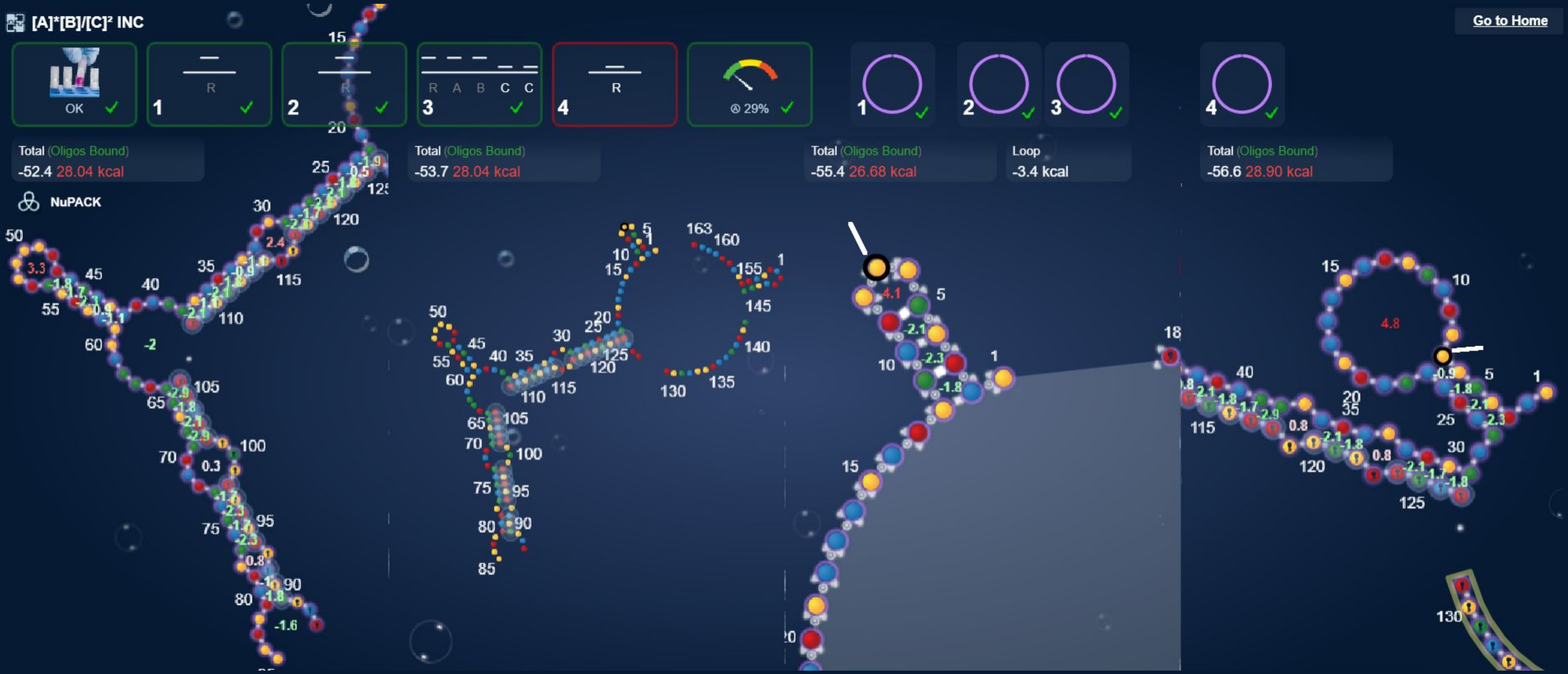

AUGACAUAGUCAGUAUUUAGUCUUGUCUCAGUAUGUACCUGUAUCUGGAAAAGCAGAUAAUCCGCC
ACCGUGCUCUCAGUGAGAA

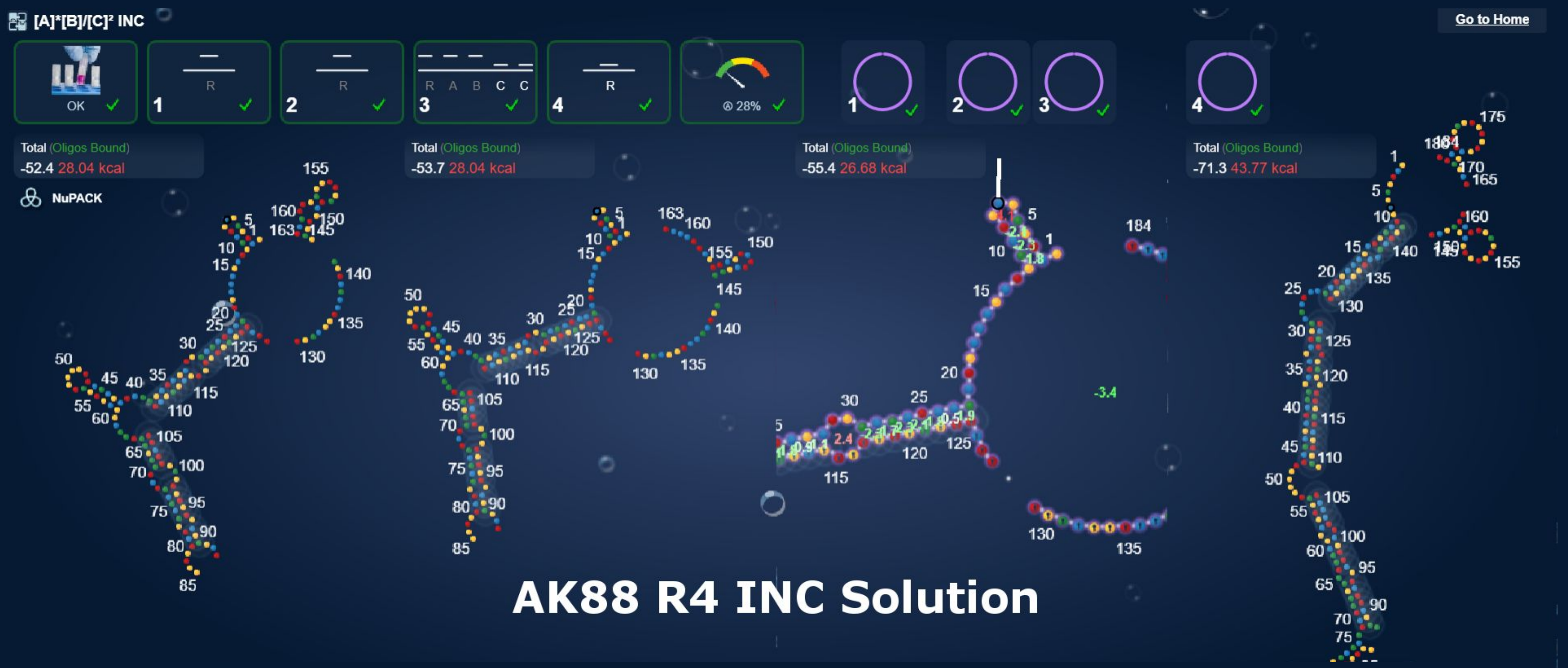

AK88 R4 INC Solution

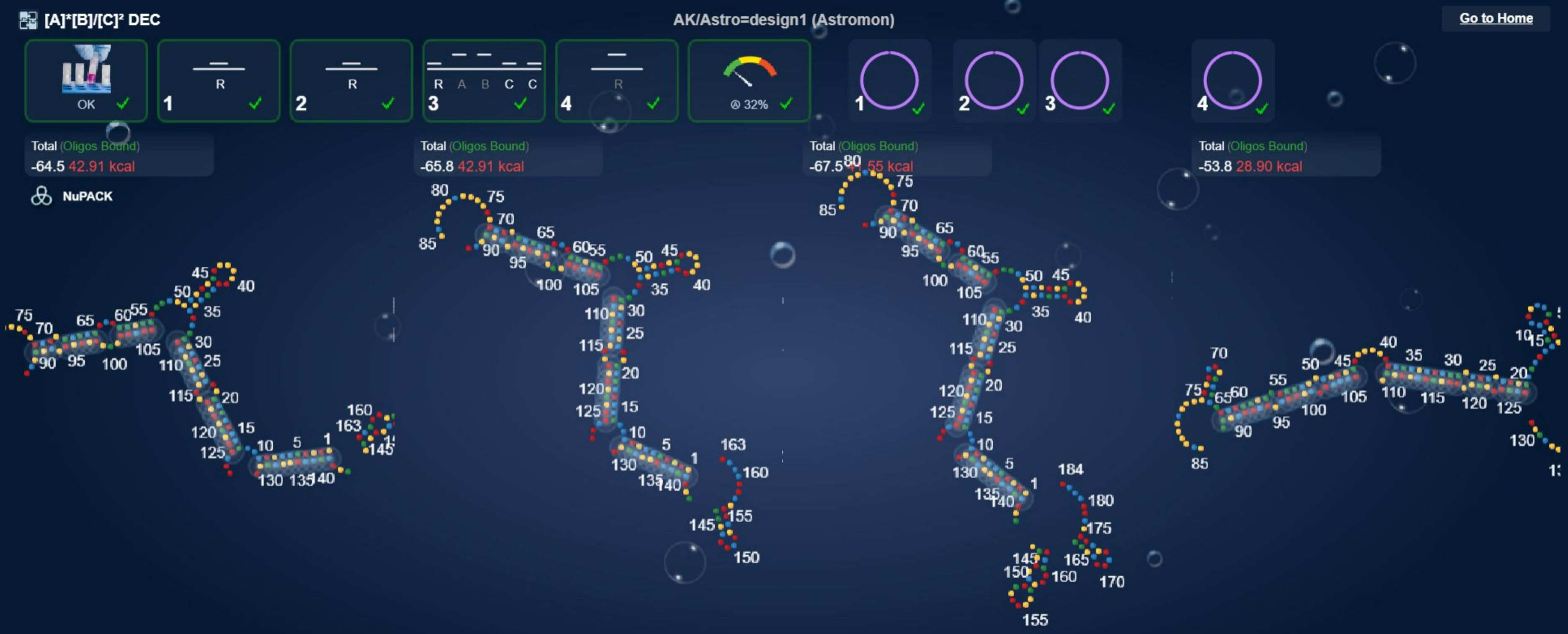

[A]*[B]/[C]² DEC
AK/Astro=design1 (Astromon)
Go to Home
OK
1 R
2 R
3 R A B C C
4 R
32%
1
2
3
4
Total (Oligos Bound)
-64.5 42.91 kcal
NuPACK
Total (Oligos Bound)
-65.8 42.91 kcal
Total (Oligos Bound)
-67.5 41.55 kcal
Total (Oligos Bound)
-53.8 28.90 kcal

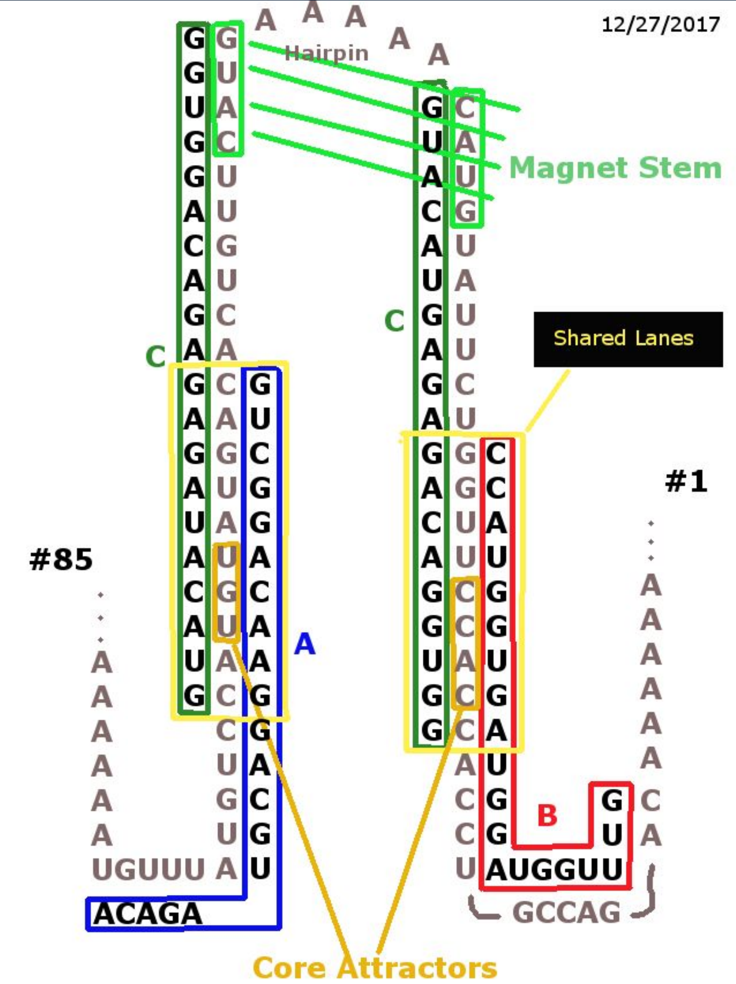

Rough Design for AK99 from #neutral for DEC and INC

State 1 - 3

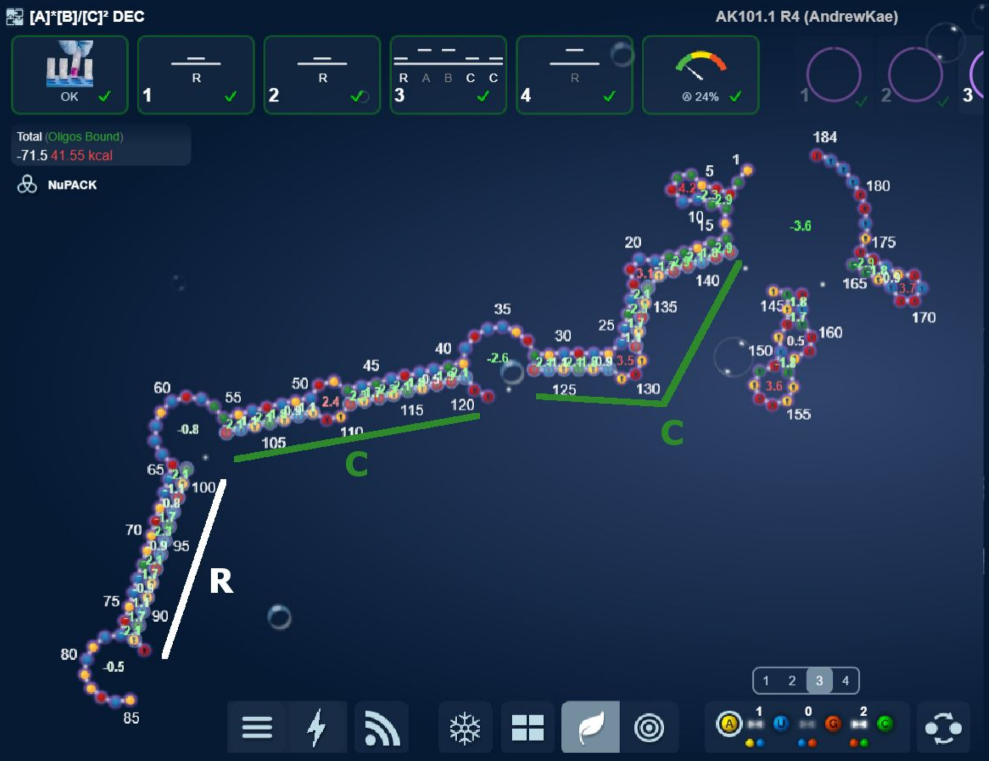

State 4

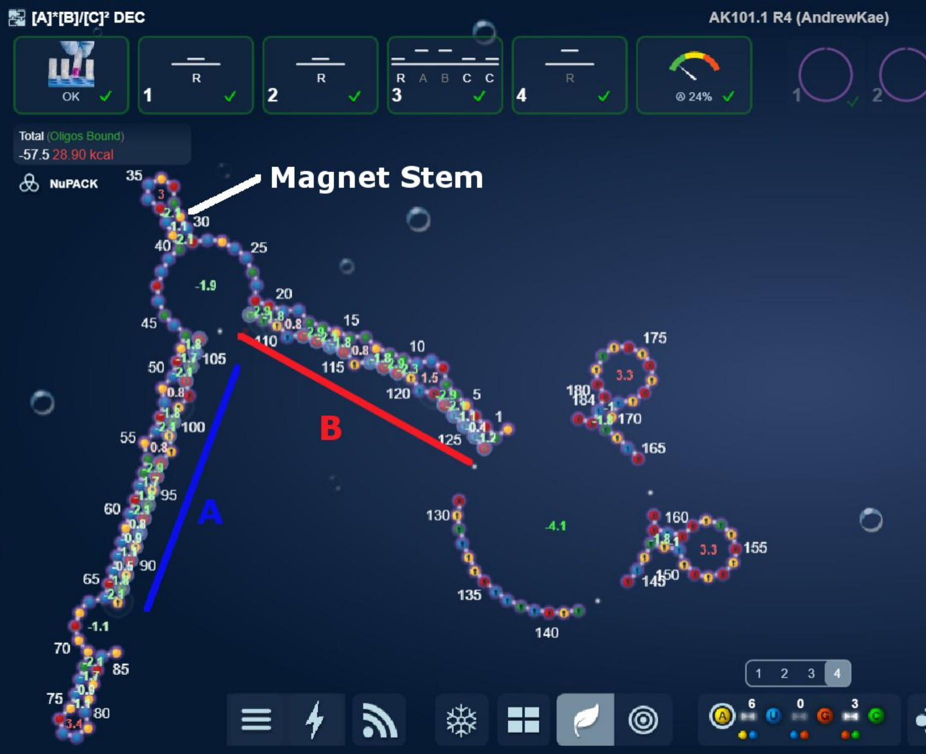

Thank You

You can view this presentation in article form, with links to solutions: here
