# Supplementary material for: Compact RNA sensors for increasingly complex functions of multiple inputs
Source: Nat Chem. 2025 Nov 12;17(12):1839–52. doi: 10.1038/s41557-025-01907-8 (PMC12669046; doi:10.1038/s41557-025-01907-8)

Results for Eterna R102

Logic Gates

http://www.eternagame.org/web/lab/6434655/

Johan Andreasson, 4/7/2016

Introduction

The ideal solution to these puzzles is an RNA design that performs a logical operation, i.e., a logic gate (https://en.wikipedia.org/wiki/Logic_gate). We first introduced The Real Logic Challenge using NuPACK (www.eternagame.org/web/lab/6096398/) in Round 98 and user designed many successful solutions. An introduction to the logic gates is found in the summary PDF of the Round 98 results.

In the current round we have expanded the number of targeted gates to include all the interesting two-input logic gates:

Operator	Symbol	Conditions			
		No ligand	Oligo A	Oligo B	Oligos A & B
NOR	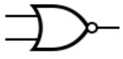	T (ON)	F (OFF)	F (OFF)	F (OFF)
A AND NOT B	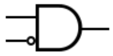	F (OFF)	T (ON)	F (OFF)	F (OFF)
XOR	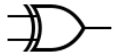	F (OFF)	T (ON)	T (ON)	F (OFF)
NAND	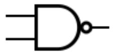	T (ON)	T (ON)	T (ON)	F (OFF)
AND	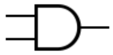	F (OFF)	F (OFF)	F (OFF)	T (ON)
XNOR	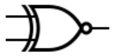	T (ON)	F (OFF)	F (OFF)	T (ON)
A OR NOT B	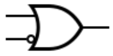	T (ON)	T (ON)	F (OFF)	T (ON)
OR	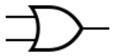	F (OFF)	T (ON)	T (ON)	T (ON)

For our experiments, the ON- and OFF-states are characterized by strong and weak MS2 binding, respectively. Here is an example for the AND gate:

		Oligo A	
		0 nM	100 nM
Oligo B	0 nM	High K_D	High K_D
	100 nM	High K_D	Low K_D

Scoring of logic gate puzzles

An **Eterna score** (0–100) is used for scoring the RNA logic gates. Similar to the riboswitch puzzles, it is the sum of a Folding subscore (0–30), a Baseline subscore (0–30) and a Switch subscore (0–40). For details about the riboswitch scoring, please see Scoring of riboswitches in Eterna. In Round 102 all the K_D values were normalized to their respective MS2 control K_D values which varied from 5.4 to 7.0 nM.

The **Folding subscore** is determined by the maximum (best) intensity (F_{max}) of the ON states. For an F_{max} above 0.7 the score is 30, below 0.3 the score is 0, and in between the score increases linearly with the F_{max}.

The **Baseline subscore** is based on the highest (worst) K_D of the ON states. If the value is within a factor of two of the K_D measured on MS2 hairpin controls (~ 6 nM in Round 102) the score is 30. Above that point, the score decreases linearly with the measured K_D until the K_D is more than six-fold higher than the control (i.e., >36 nM). For all higher K_D values the score is 0.

The **Switch score** is based on the highest (worst) K_D of the ON states and the lowest (also worst) K_D of the OFF states. The score is proportional to the logarithm of the fold change in K_D between the ON and OFF states, i.e. $subscore \sim \log\left(\frac{K_{D,OFF}}{K_{D,ON}}\right)$. The subscore is 0 if the ratio is less than 1 and the maximum score of 40 is reached when the ratio is 75. The score is very similar to the riboswitch scores but a high score is harder to achieve since all four states have to be correct. The score is based on the K_D values that minimize the separation between the ON and OFF states.

Scores for all logic gate puzzles

The second highest peak in the Eterna score histogram mostly corresponds to designs with Switch score = 0. The largest peak includes designs that also have Baseline subscore = 0. As always, the most promising designs fall in the tail with high scores.

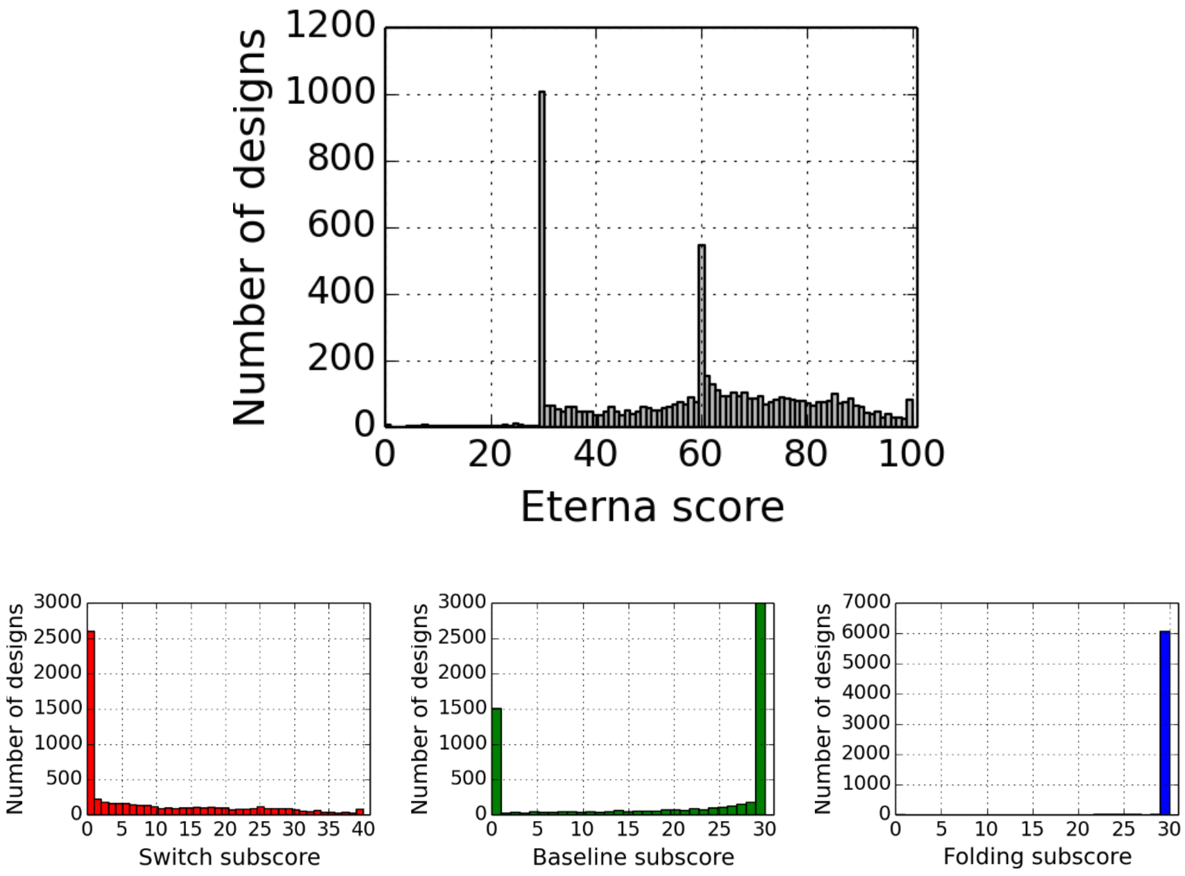

NOR (TFFF)

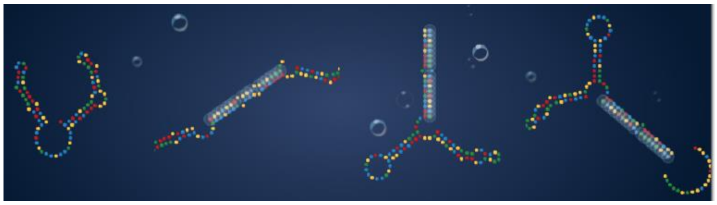

mod Astromon 6474721_C9_U29 #MUTATE_TWO #NUPACK_SCORING
by Eli Fisker, Eterna score 98

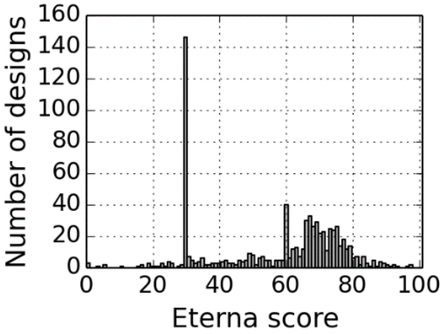

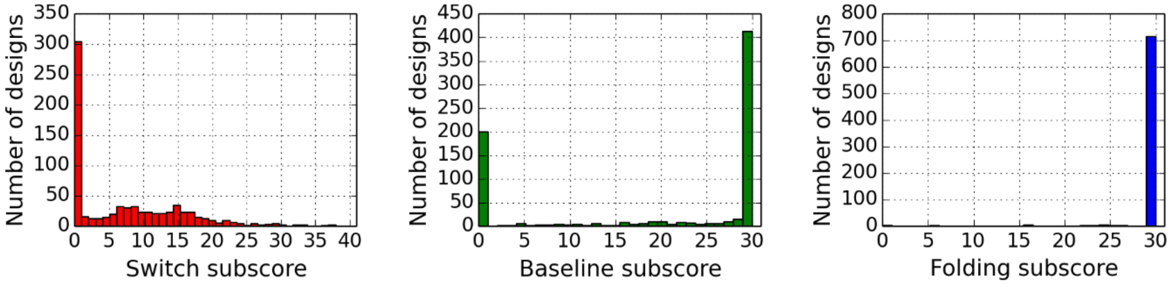

Dissociation constants (K_D)

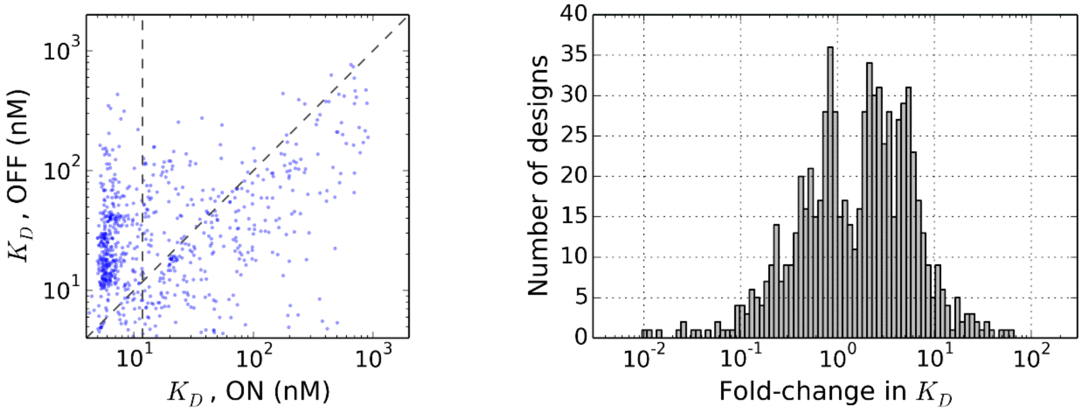

A AND NOT B (FTFF)

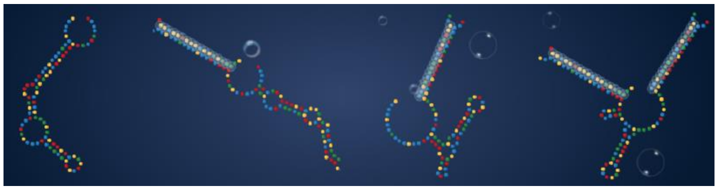

6492799_U84 #MUTATE_ONE #NUPACK_SCORING by mat747,
Eterna score 100

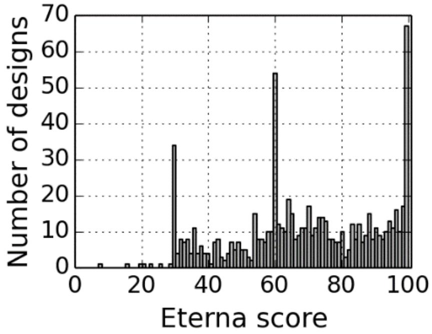

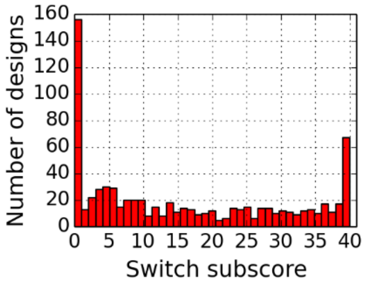

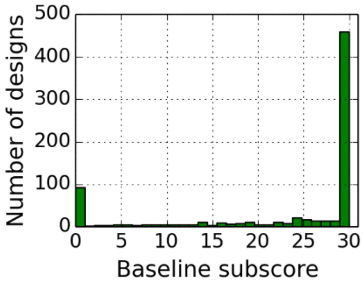

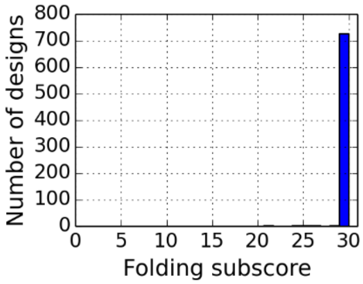

Dissociation constants (K_D)

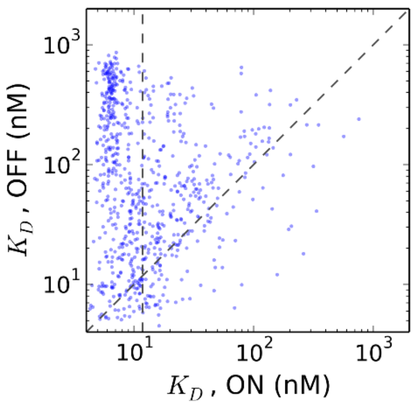

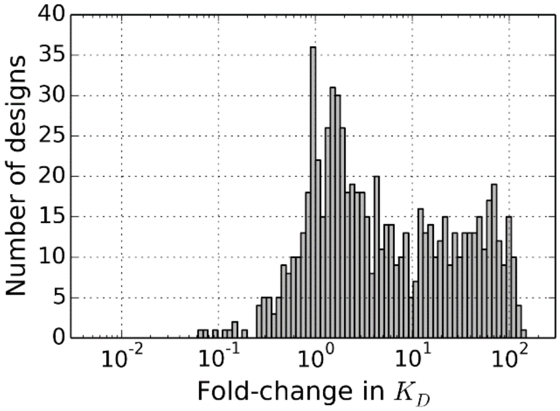

XOR (FTTF)

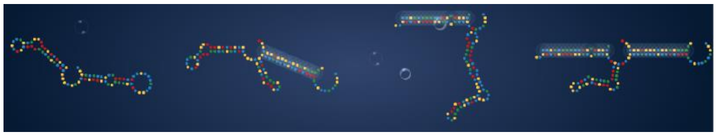

6136140_U79_U76 #MUTATE_TWO by jandersonlee, Eterna score 98

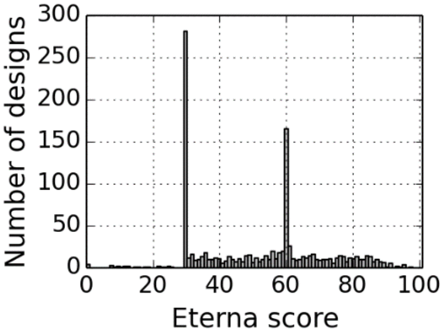

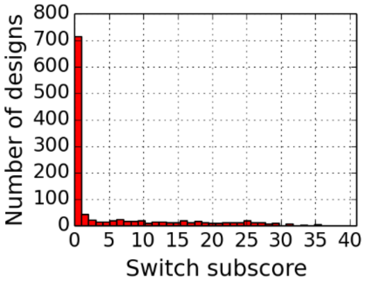

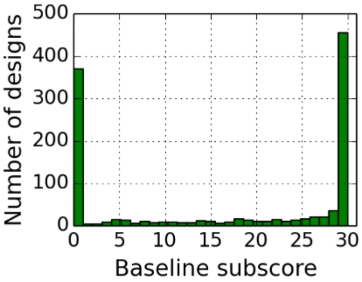

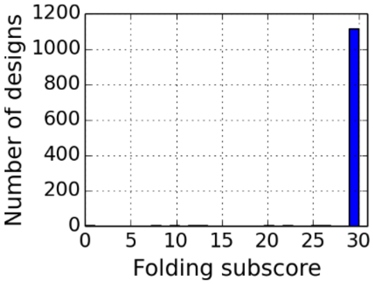

Dissociation constants (K_D)

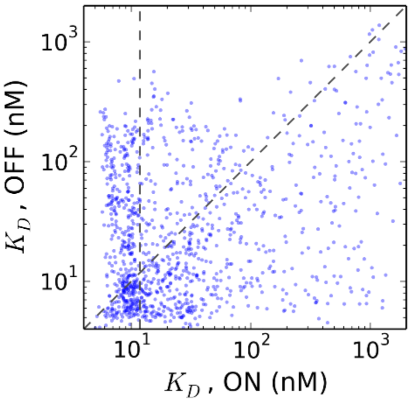

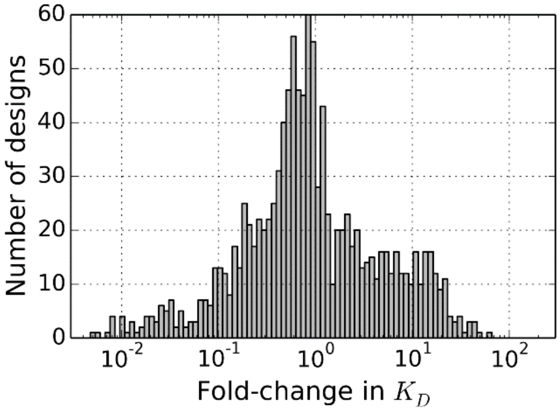

NAND (TTTF)

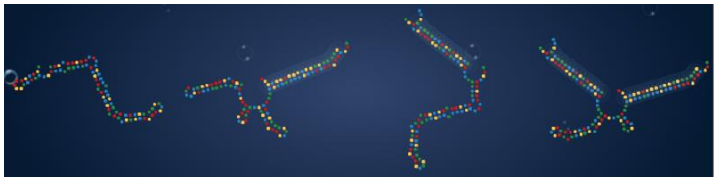

NAND (TTTF) by worseize, Eterna score 100

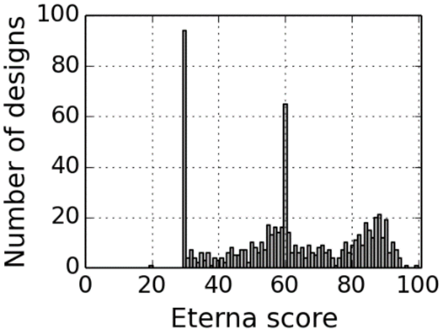

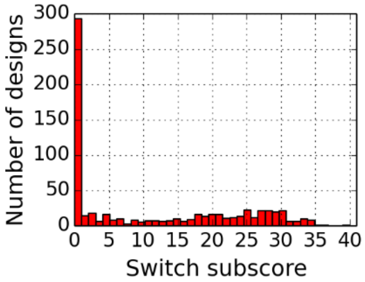

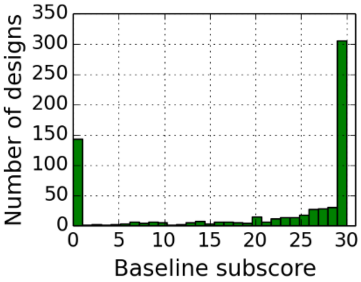

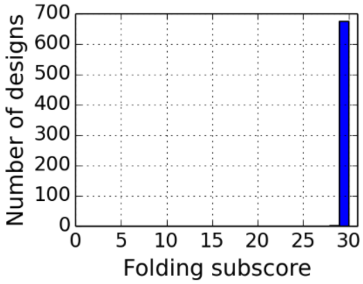

Dissociation constants (K_D)

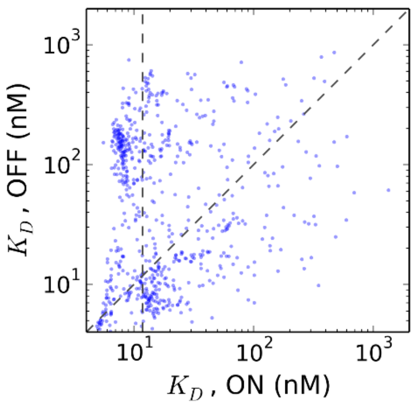

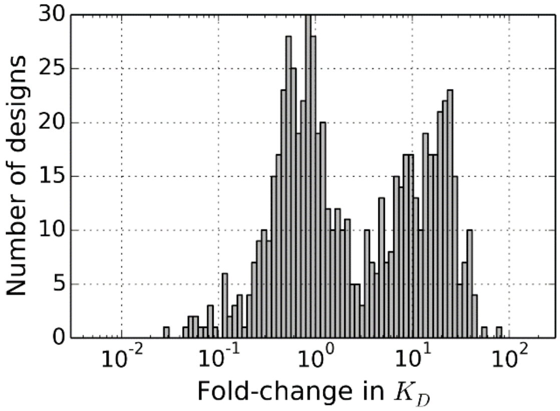

AND (FFFT)

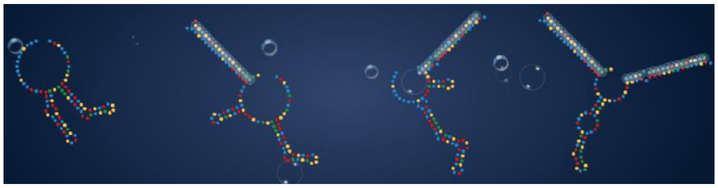

Eli Algo 1.08 - Mutation storm by Eli Fisker, Eterna score 99

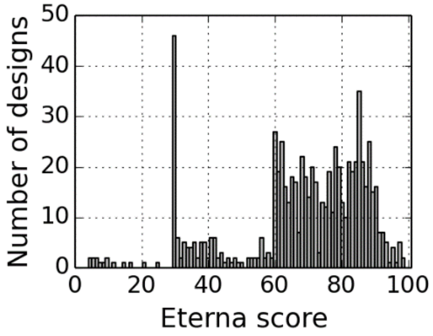

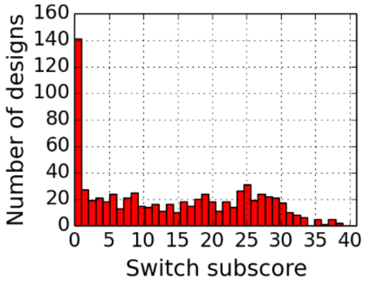

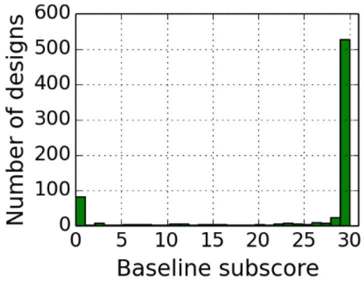

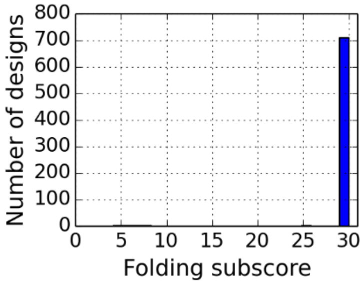

Dissociation constants (K_D)

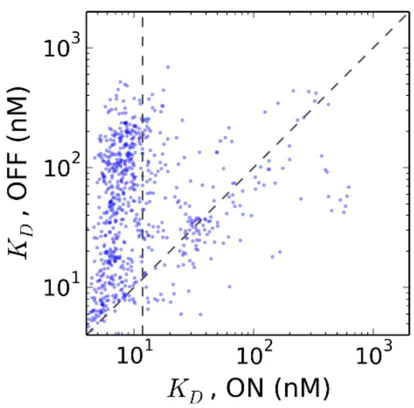

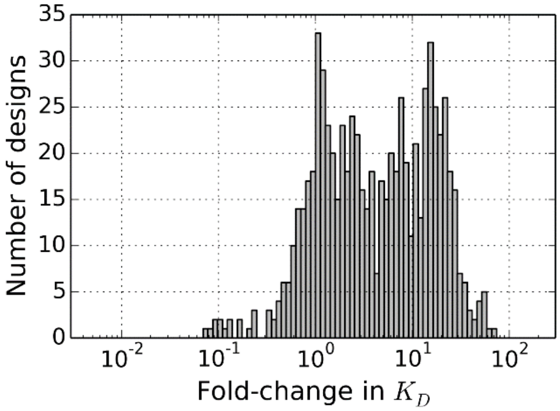

XNOR (TFFT)

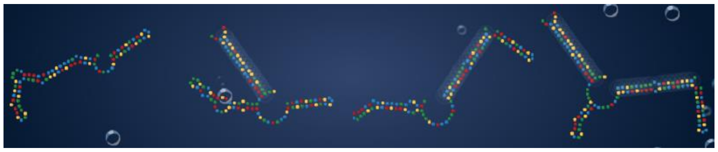

6133692 #MUTATE_FOUR #MIXED_SCORING by Eli Fisker,
Eterna score 100

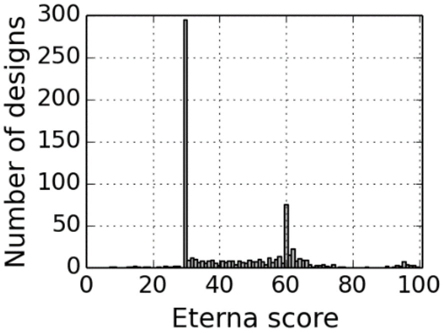

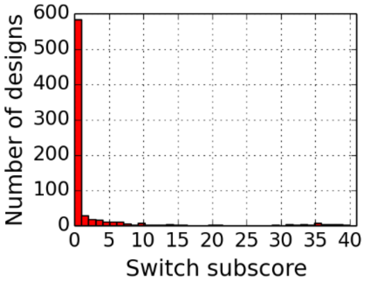

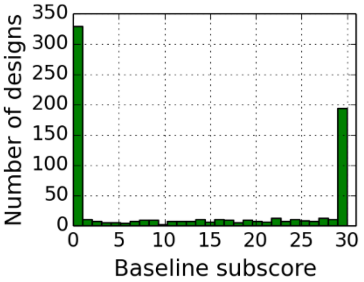

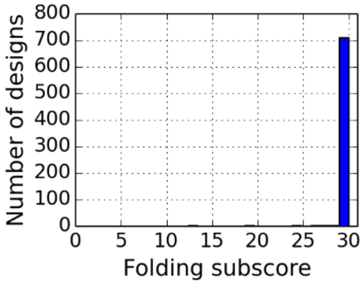

Dissociation constants (K_D)

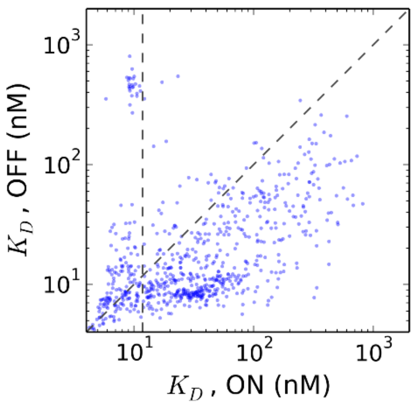

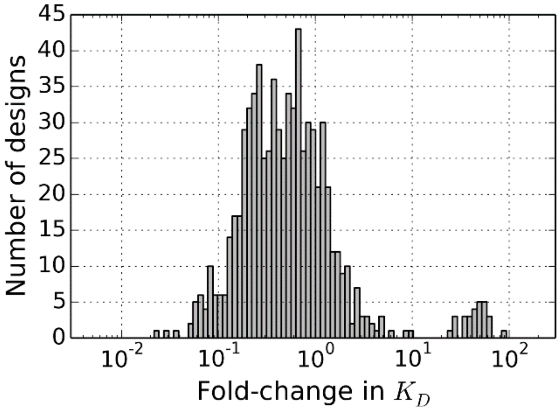

A OR NOT B (TTFT)

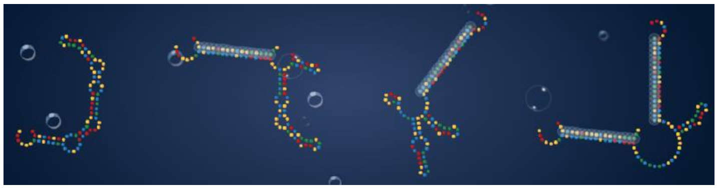

#MUTATE_ONE 6112196_C27 by Eli Fisker, mat747 score 100

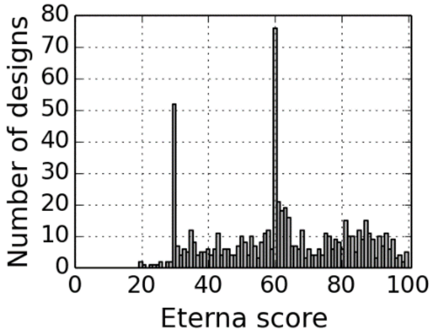

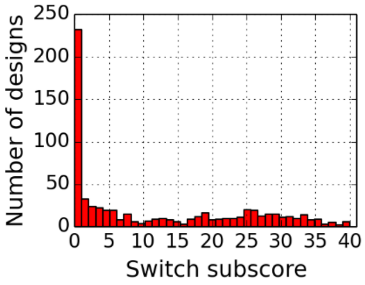

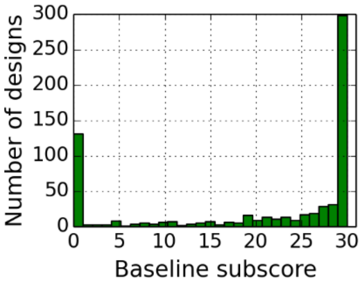

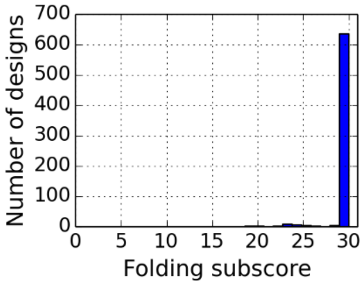

Dissociation constants (K_D)

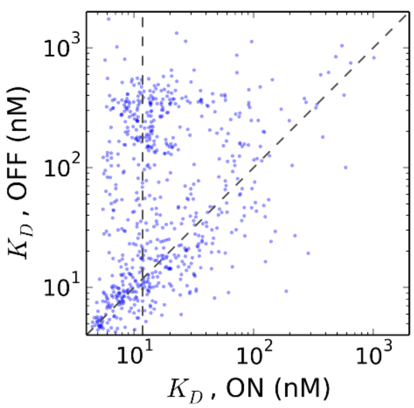

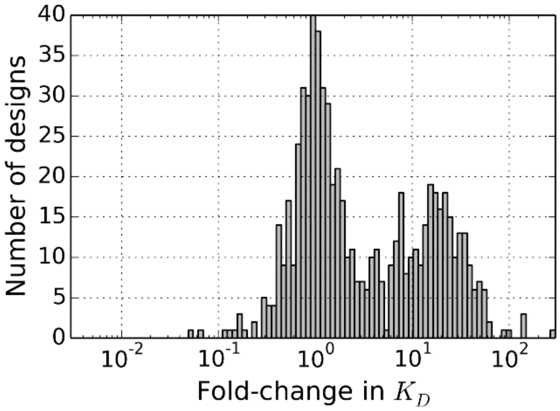

OR (FTTT)

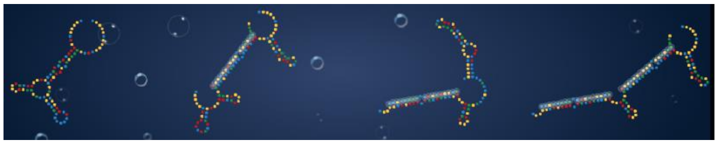

Static middle stem by Eli Fisker, Eterna score 100

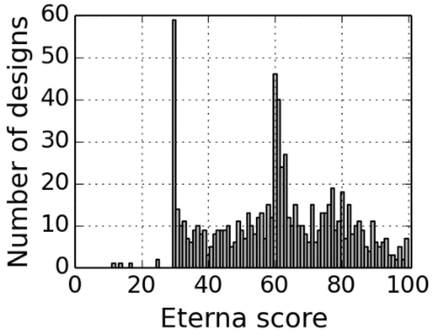

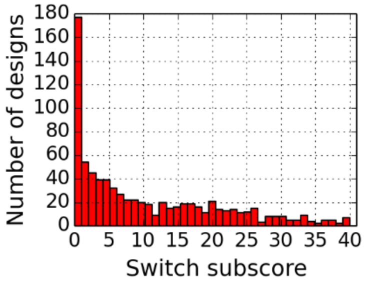

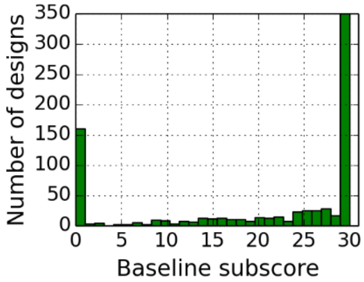

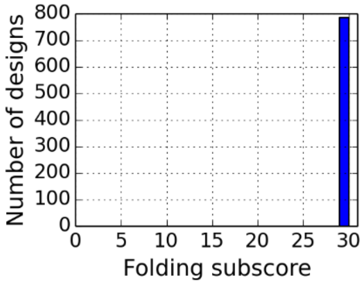

Dissociation constants (K_D)

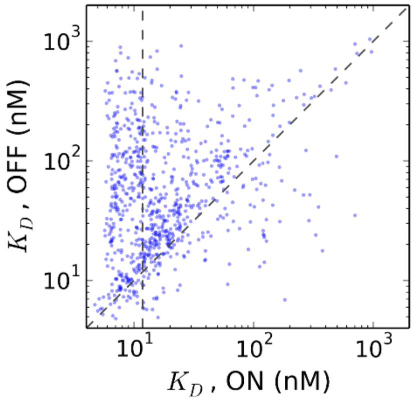

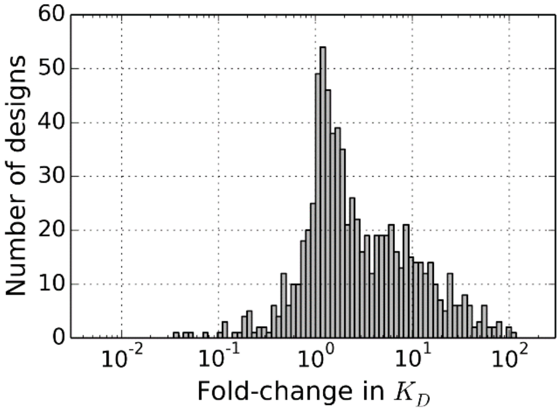

Supplement: Supplementary file 2 — Contains Supplementary Appendix: Eterna player resources (Eterna player resources also listed in Supplementary Table 4). Also contains the newsletters the Das lab shared with Eterna players after each design challenge. [file 41557_2025_1907_MOESM2_ESM.zip › supplemental_newsletter.zip!/supplemental_newsletter/Challenge_1_R102_Logic_gates_results.pdf]
