# Supplementary material for: Compact RNA sensors for increasingly complex functions of multiple inputs
Source: Nat Chem. 2025 Nov 12;17(12):1839–52. doi: 10.1038/s41557-025-01907-8 (PMC12669046; doi:10.1038/s41557-025-01907-8)
Supplement: Supplementary file 2 — Contains Supplementary Appendix: Eterna player resources (Eterna player resources also listed in Supplementary Table 4). Also contains the newsletters the Das lab shared with Eterna players after each design challenge. [file 41557_2025_1907_MOESM2_ESM.zip › supplemental_newsletter.zip!/supplemental_newsletter/Challenge_1_R98_Logic_gates_results.pdf]

Results for Eterna R98

The Real Logic Challenge using NuPACK

http://www.eternagame.org/web/lab/6096398/

Johan Andreasson, 01/05/2016 (updated 1/21/2016)

Introduction

In Eterna Round 98 we deployed logic gate puzzles of the first time. An ideal solution is an RNA design that performs a logical operation, i.e., a logic gate (https://en.wikipedia.org/wiki/Logic_gate).

The design of biomolecules that perform Boolean logic is an important challenge in synthetic biology and several different implementations have been demonstrated[1]. The RNA logic gates in Eterna are cell-free systems that are unique in that they use two short RNA oligos as inputs but a fluorescent protein, MS2, as the output. In theory, the computations are also reversible unlike many ribozyme based logic devices that cleave the RNA. Using few components, players can in principle design any of the sixteen binary logical operators (en.wikipedia.org/wiki/Truth_table#Truth_table_for_all_binary_logical_operators).

We targeted three common Boolean operations:

1. OR Logic Gate (www.eternagame.org/game/puzzle/6096395/)
2. AND Logic Gate (www.eternagame.org/game/puzzle/6096396/)
3. XOR Logic Gate (www.eternagame.org/game/puzzle/6096397/)

The output for ideal gates can be represented by their truth tables

AND gate

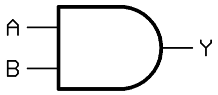

INPUT		OUTPUT
A	B	A AND B
0	0	0
0	1	0
1	0	0
1	1	1

OR gate

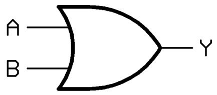

INPUT		OUTPUT
A	B	A OR B
0	0	0
0	1	1
1	0	1
1	1	1

XOR gate

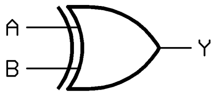

INPUT		OUTPUT
A	B	A XOR B
0	0	0
0	1	1
1	0	1
1	1	0

Both inputs and outputs are binary for ideal gates. For our Eterna experiments, on the other hand, states are determined by reversible binding of both oligos and MS2 which means that the signals are often somewhere between “0” and “1” (or high and low K_D, respectively). The grand challenge is to create designs that maximize the difference in binding between the ON states (1 or TRUE) and the OFF states (0 or FALSE).

The corresponding truth table for an RNA based AND gate becomes

AND gate<br>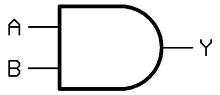		
**INPUT**		**OUTPUT**
**Oligo A**	**Oligo B**	**MS2 binding**
0 nM	0 nM	Weak binding, High K_D
0 nM	100 nM	Weak binding, High K_D
100 nM	0 nM	Weak binding, High K_D
100 nM	100 nM	Strong binding, Low K_D

or, equivalently

		**Oligo A**	
		0 nM	100 nM
**Oligo B**	0 nM	High K_D	High K_D
	100 nM	High K_D	Low K_D

We measure full binding curves for each design and these are characterized by the dissociation constant, K_D, which is the concentration of MS2 at which the binding signal reaches its half-maximum. The ON state is defined as a low K_D, which means strong binding. As a reference, a low K_D in our experiments is 20 nM and a high K_D is 500 nM. The lower limit is by the binding affinity of MS2 to its native hairpin (~19 nM). The upper limit is set by background fluorescence in our microscope that precludes data collection beyond the low micromolar range.

Below is an example of binding curves for a good AND gate in Eterna. The K_D for the A AND B state (green) is low and the K_D for the other three states are high.

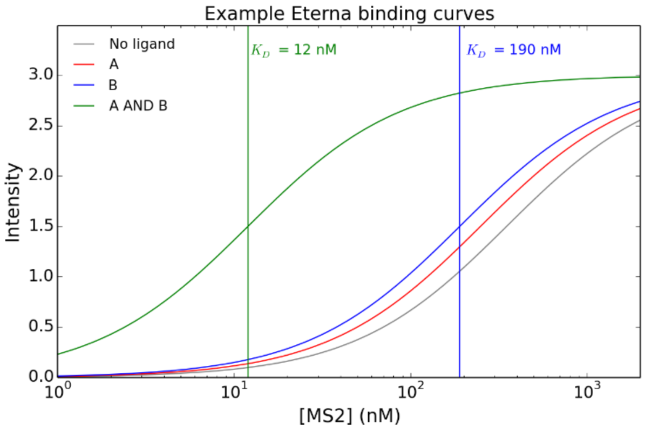

Scoring of logic gate puzzles

An **Eterna score** (0–100) is used for scoring the RNA logic gates. Similar to the riboswitch puzzles, it is the sum of a Folding subscore (0–30), a Baseline subscore (0–30) and a Switch subscore (0–40). For details about the riboswitch scoring, please see Scoring of riboswitches in Eterna.

The **Folding subscore** is determined by the maximum (best) intensity (F_{max}) of the ON states. For an F_{max} above 0.7 the score is 30, below 0.3 the score is 0, and in between the score increases linearly with the F_{max}.

The **Baseline subscore** is based on the highest (worst) K_D of the ON states. If the value is within a factor of two of the K_D measured on MS2 hairpin controls (~ 19 nM in Round 98) the score is 30. Above that point, the score decreases linearly with the measured K_D until the K_D is more than six-fold higher than the control (i.e., >114 nM). For all higher K_D values the score is 0.

The **Switch score** is based on the highest (worst) K_D of the ON states and the lowest (also worst) K_D of the OFF states. The score is proportional to the logarithm of the fold change in K_D between the ON and OFF states, i.e. $subscore \sim \log\left(\frac{K_{D,OFF}}{K_{D,ON}}\right)$. The subscore is 0 if the ratio is less than 1 and the maximum score of 40 is reached when the ratio is 75. The score is very similar to the riboswitch scores but a high score is harder to achieve since all four states have to be correct. The score is based on the K_D values that minimize the separation between the ON and OFF states, i.e. the distance between the green and blue vertical lines in the figure above.

Scores for all logic gate puzzles

The second highest peak in the Eterna score histogram corresponds to designs with Switch score = 0. The largest peak includes designs that also have Baseline subscore = 0. As always, the most promising designs fall in the tail with high scores.

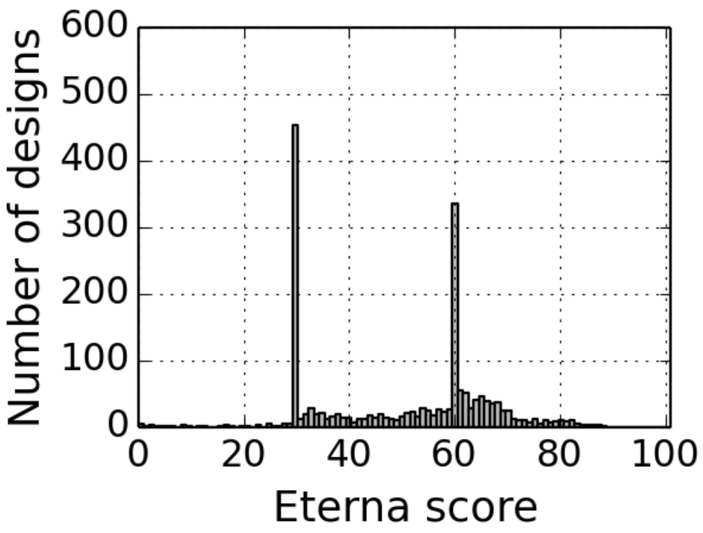

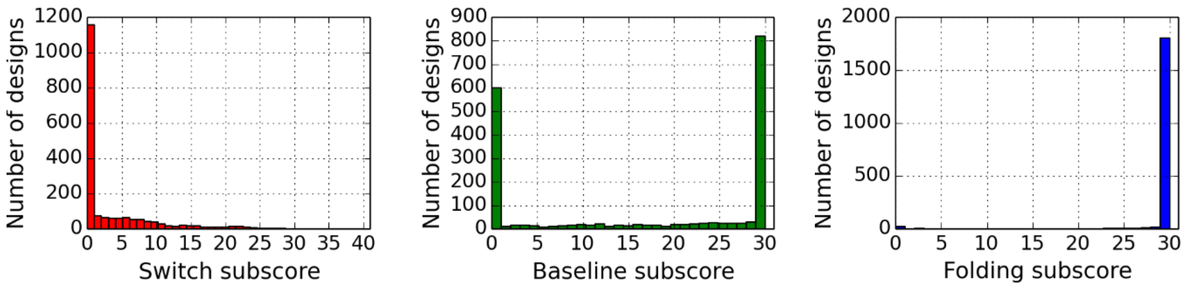

All puzzles (same as above), different *y*-axis scales

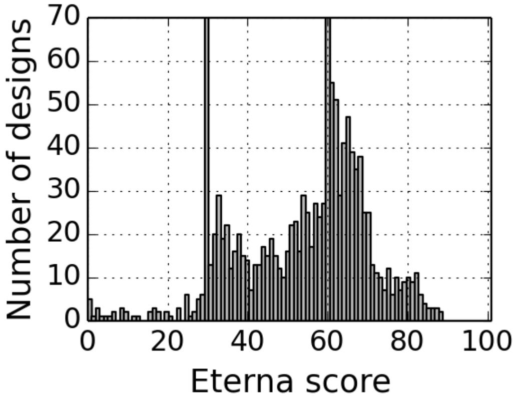

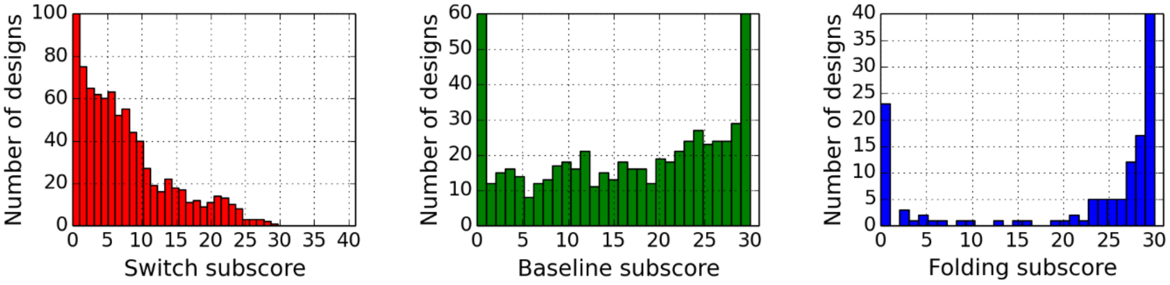

OR Logic Gate

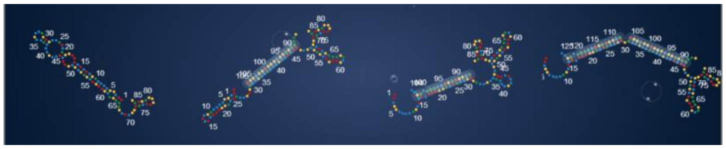

jl-olg-1.06 Brourd mod by jandersonlee, Eterna score 88

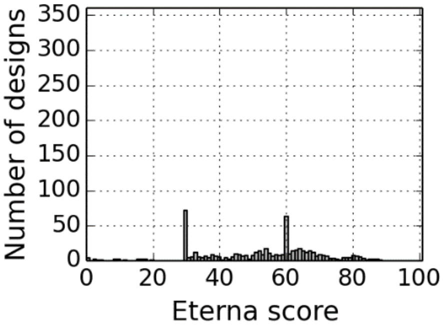

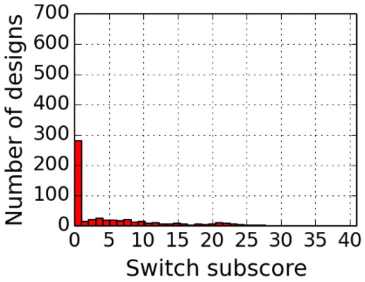

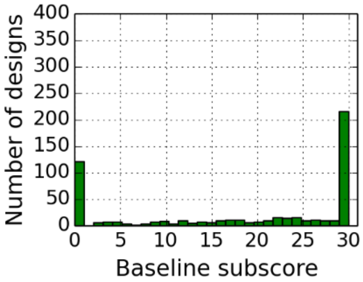

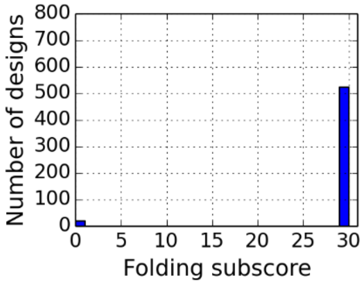

Different *y*-scale

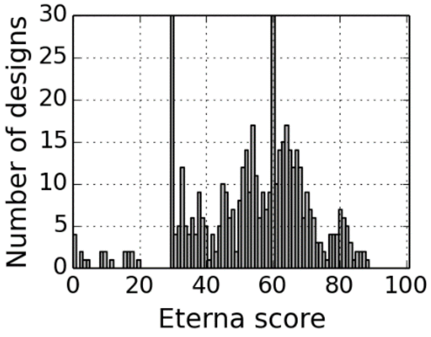

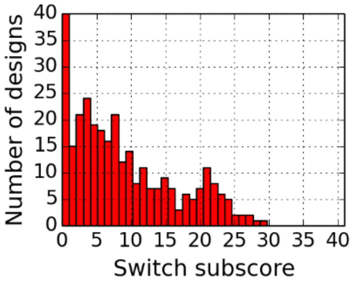

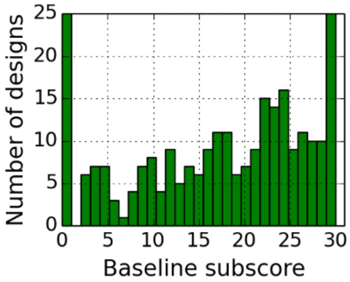

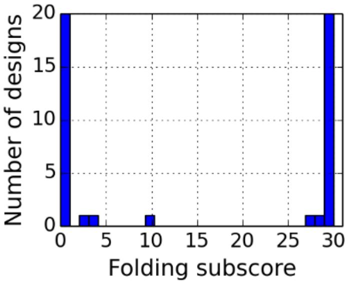

AND Logic Gate

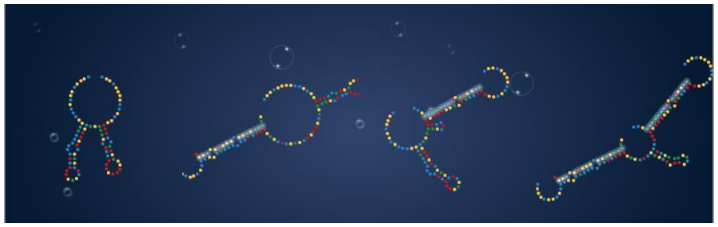

jl-and-1.10 Eli mod by jandersonlee, Eterna score 88

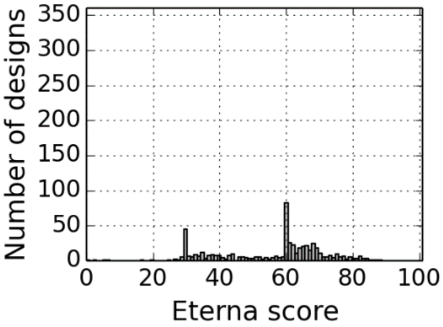

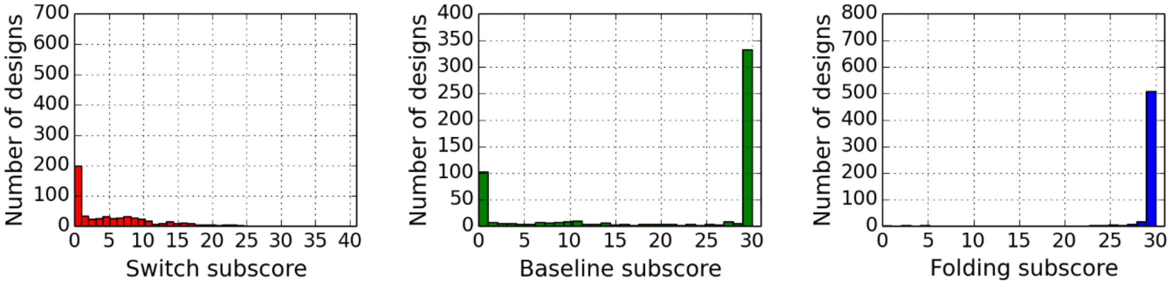

Different *y*-scale

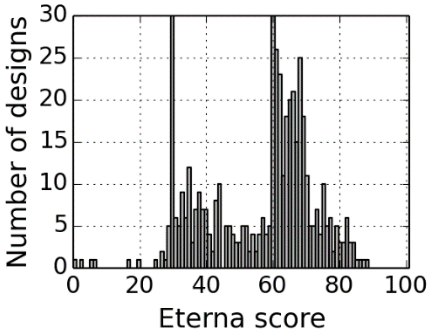

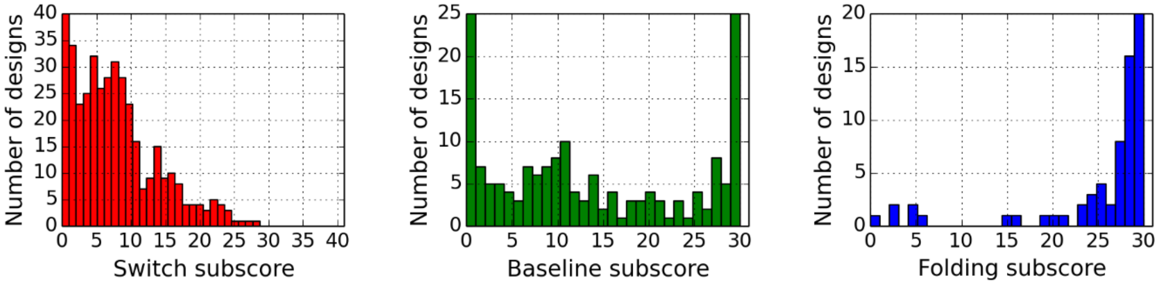

XOR Logic Gate

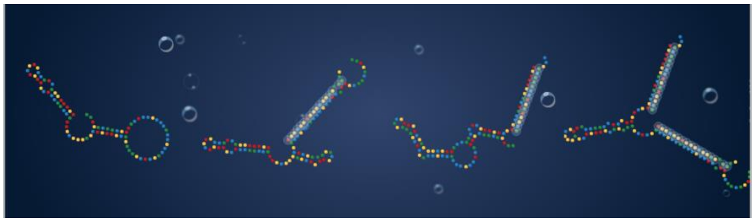

BXM 28 by ElNando888, Eterna score 78

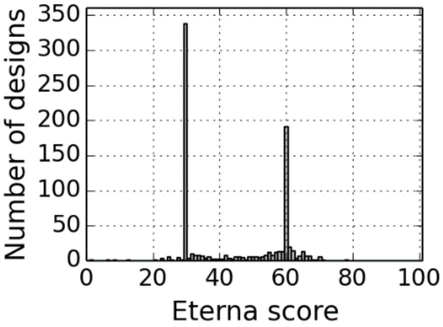

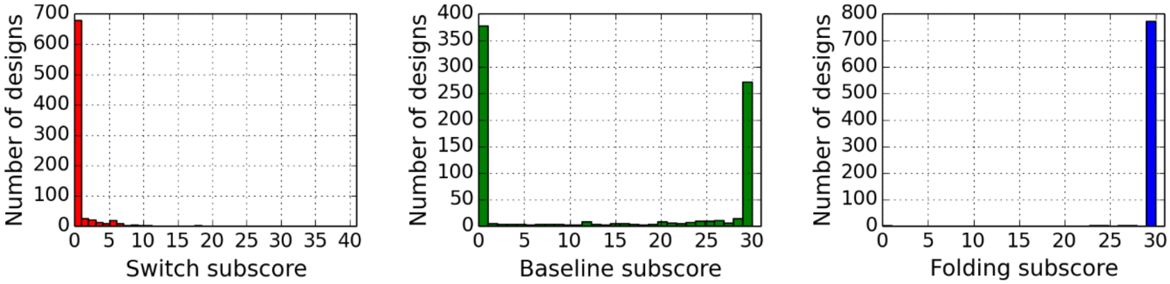

Different *y*-scale

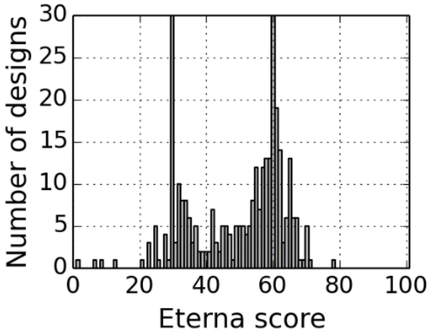

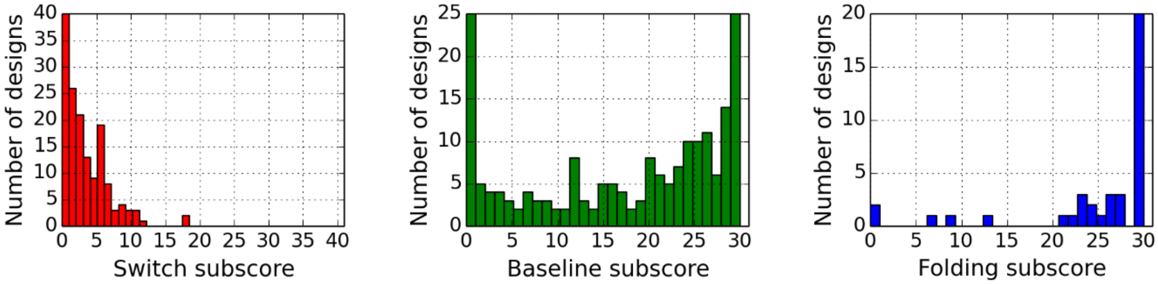

Fold change in K_D for all puzzles

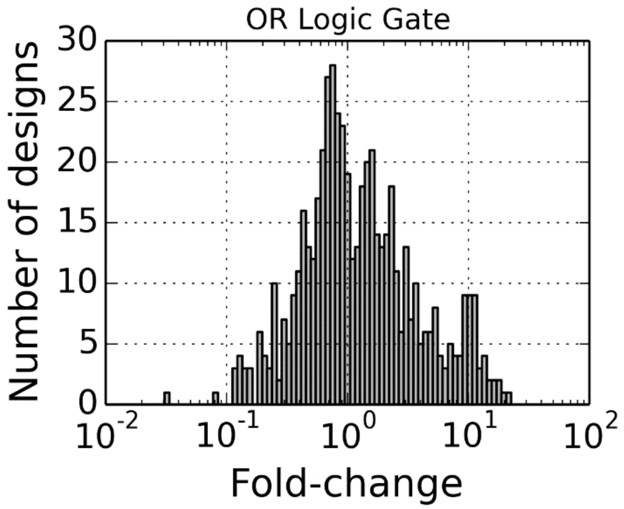

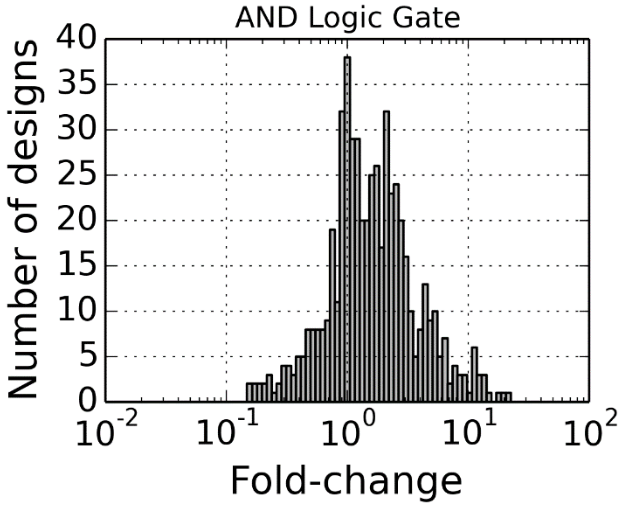

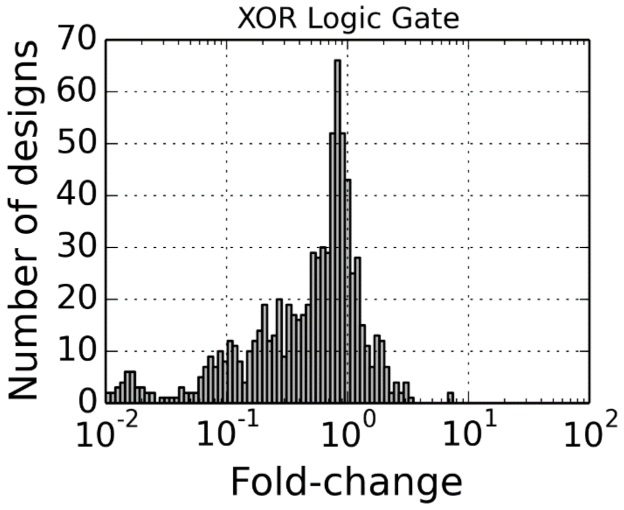

References

1 Miyamoto, T., Razavi, S., DeRose, R. & Inoue, T. Synthesizing biomolecule-based Boolean logic gates. *ACS Synth Biol* **2**, 72-82 (2013).
