# Supplementary material for: Compact RNA sensors for increasingly complex functions of multiple inputs
Source: Nat Chem. 2025 Nov 12;17(12):1839–52. doi: 10.1038/s41557-025-01907-8 (PMC12669046; doi:10.1038/s41557-025-01907-8)

Results for Eterna R103

[A]/[B] puzzles

http://www.eternagame.org/web/lab/6645697/

Johan Andreasson, 07/26/2016

Introduction

Expressions including several concentrations of molecules may be used to determine disease status in medical diagnostics. This was the second time we designed riboswitches to test such an expression: a ratio between two RNA concentrations. The final switches should be ON if the ratio [A]/[B] is larger than 1/4, where A and B are two different input RNA oligonucleotides.

Tutorial puzzles

As in Round 100, several puzzles were presented to introduce the problem. We started by designing molecules that bind MS2 or not (**MS2 control ON** and **MS2 control OFF**). The next step was to create switches that either turn on or off in the presence of an RNA input at a concentration of 100 nM. Two puzzles (**Sensor A MS2 ON** and **Sensor A MS2 OFF**) used a molecule that we call TB-A (in anticipation of a tuberculosis signature) or simply A. Two other puzzles (**Sensor B MS2 ON** and **Sensor B MS2 OFF**) used a second RNA molecule (TB-B or B).

[A]/[B] puzzles

Both of the RNA inputs (A and B) were combined in three two-state puzzles where the difference between the states was the concentration of A. Good switches are ON, or bind MS2, when [A] = [B] = 100 nM. The ratio is [A]/[B] = 1 which is higher than the 1/4 cutoff. The switches should be OFF, or not bind MS2, when [A] = 5 nM and [B] = 100, i.e., the ratio [A]/[B]=1/20 is less than 1/4.

Two puzzles (**[A]/[B] with predefined binding sites** and **[A]/[B] with predefined binding sites (alternative)** provided some guidance and **R2 (2-states model)** allowed the players to design from scratch.

Finally, in **R3 (3-states model)** we tried to design a switch that is correct in three states, all with different concentrations of A and B and with different ratios that fall on either side of the cutoff. The three states were

1) [A]=0 nM, [B]=5 nM
2) [A]=[B]=5 nM
3) [A]=5 nM, [B]=100 nM.

The corresponding ratios were [A]/[B]=0, 5, and 1/20, respectively.

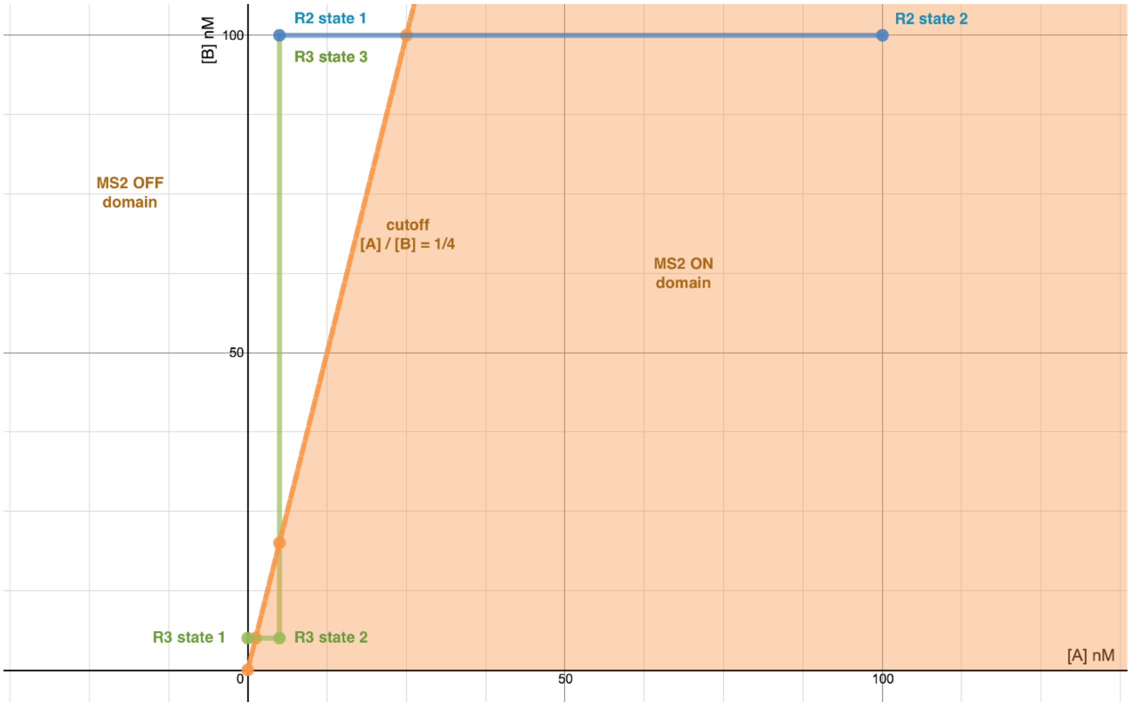

Scoring of RNA in –RNA out puzzles

As for most puzzles, the **fold-change in K_D** is the best single metric for good switching. We also use the Eterna score to ensure that there is reasonable binding and that the ON-state has a high affinity (low K_D).

The designs were scored based on the states present in each puzzle. In each case the maximum Eterna score is 100 and it is a combination of the useable subscores for each puzzle.

MS2 control OFF

This control puzzle only has one state that should not bind MS2. The Eterna score therefore only has one component, the Baseline subscore. Here it is defined to be 0 for K_D < 80 nM (20 times the K_D for the MS2 hairpin control, $K_{D,\,MS2}$ = 4 nM, in the no-ligand condition for this round) and 100 for K_D > 160 nM. It increases linearly in between those two concentrations

MS2 control ON

This puzzle is also a single-state control. It should bind MS2 and therefore exhibit a K_D close to $K_{D,\,MS2}$. Here we define the Baseline subscore as in previous MS2 switch puzzles. It is 30 for K_D < 8 nM (2 x $K_{D,\,MS2}$), 0 for K_D = 24 nM (6 x $K_{D,\,MS2}$), and decreases linearly in between. We also use a Folding subscore as before, determined by the intensity (F_{max}). For an F_{max} above 0.7 the score is 30, below 0.3 the score is 0, and in between the score increases linearly with the F_{max}

Sensor A MS2 ON, Sensor A MS2 OFF, Sensor B MS2 ON, and Sensor B MS2 OFF

These switch puzzles are scored identically to previous miRNA puzzles. The **Eterna score** (0–100) is the sum of a Folding subscore (0–30), a Baseline subscore (0–30) and a Switch subscore (0–40). For details

about the riboswitch scoring, see Scoring of riboswitches in Eterna. In each puzzle, the average dissociation constant for the MS2 hairpin control from all conditions was used: $K_{D,\,MS2}$ = 4 nM.

The **Folding subscore** is determined by the intensity (F_{max}) of the ON state. For an F_{max} above 0.7 the score is 30, below 0.3 the score is 0, and in between the score increases linearly with the F_{max}.

The **Baseline subscore** is based on the K_D of the ON-state. The score is 100 for for K_D < 8 nM (2 x $K_{D,\,MS2}$), 0 for K_D > 24 nM (6 x $K_{D,\,MS2}$), and decreases linearly in between.

The **Switch score** is based on the K_D of the ON-state and the OFF-state. The score is proportional to the logarithm of the fold-change in K_D between the ON- and OFF-states, i.e. $subscore \sim \log\left(\frac{K_{D,OFF}}{K_{D,ON}}\right)$. The subscore is 0 if the ratio is less than 1 and the maximum score of 40 is reached when the ratio is above 75.

([A]/[B] with predefined binding sites, **[A]/[B] with predefined binding sites (alternative), and R2 (2-states model)**

These puzzles are scored similarly to the Sensor puzzles above. The only difference is that the maximum Switch subscore is reached for a fold ratio of 15. The ratio in concentration for the input RNA in the ON- and OFF-states is 100 nM/5 nM = 20 which sets an upper theoretical limit on the switching range. We use 75% of this value (15) as a cutoff for achieving the maximum switch score.

R3 (3-states model)

The three-state puzzle is scored identically to the two-state puzzles above. For all subscores the values in the ON-state was selected from the condition with the highest K_D value, i.e., the worst of the two ON-states.

MS2 control ON

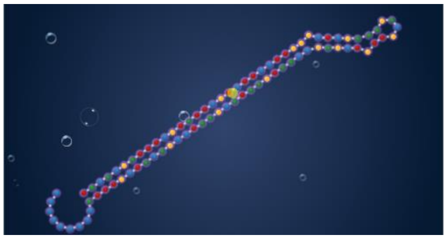

1 1 by dl2007, Eterna score 100

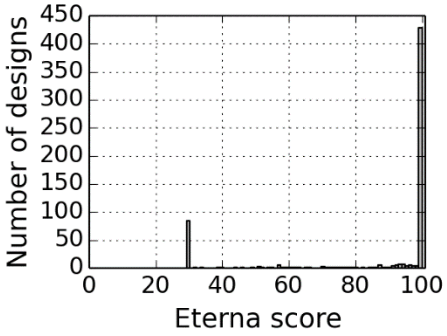

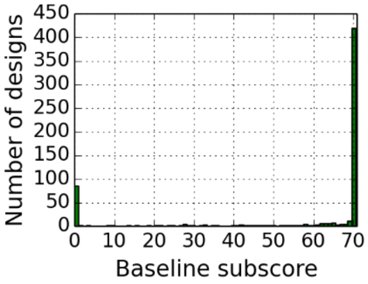

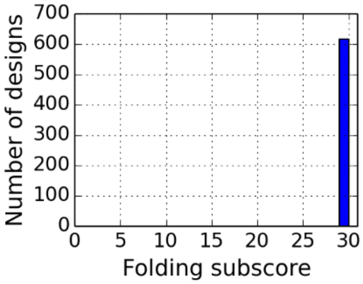

Dissociation constants (K_D)

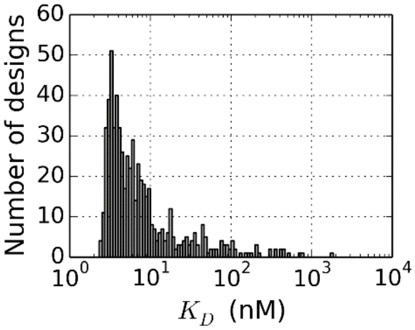

MS2 control OFF

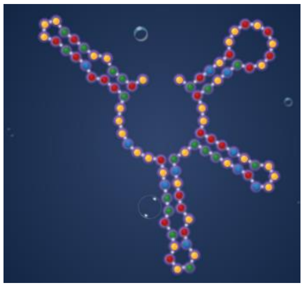

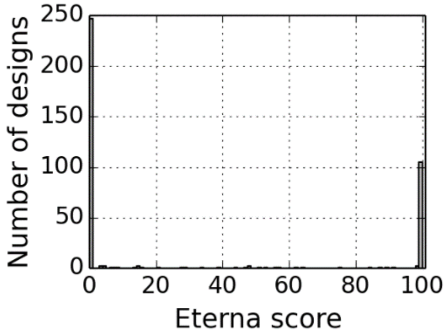

Goat Goat v2.001 by Eized, Eterna score 100

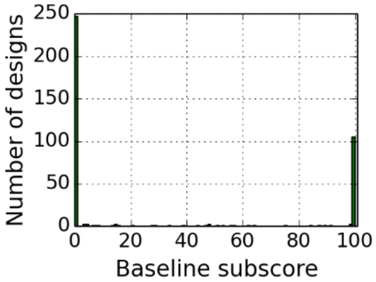

Dissociation constants (K_D)

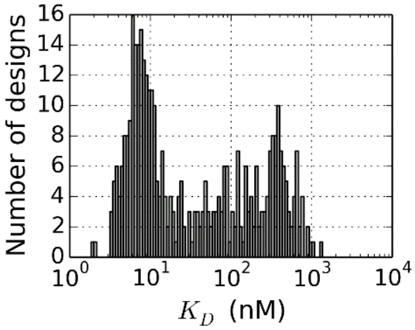

Sensor A MS2 ON

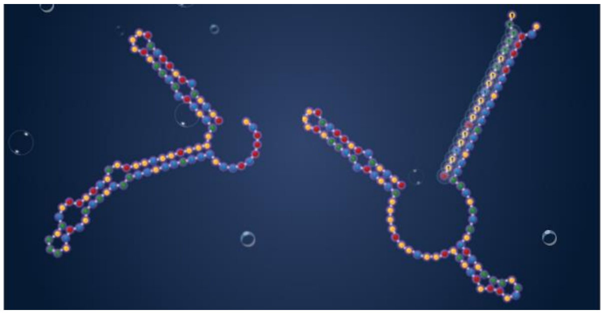

Sensor A MS2 ON 20 - word change game strategy 5 by Eli Fisker,
Eterna score 100

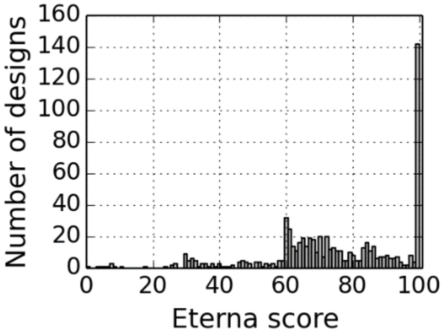

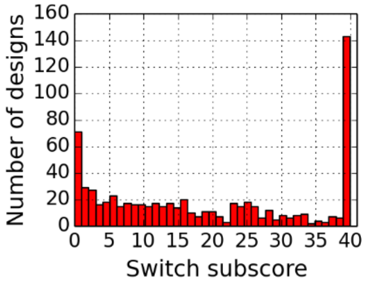

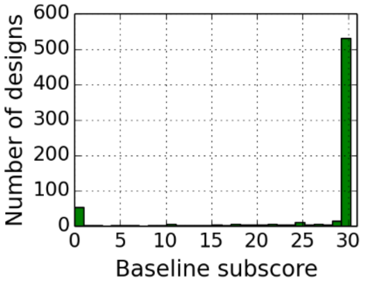

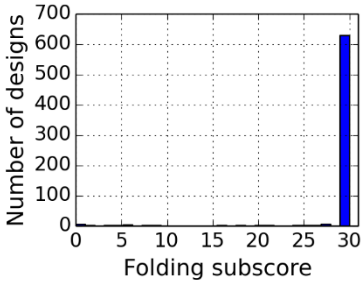

Dissociation constants (K_D)

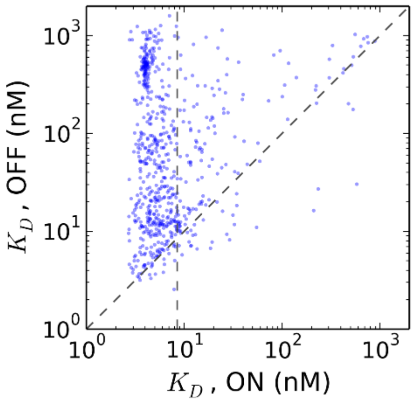

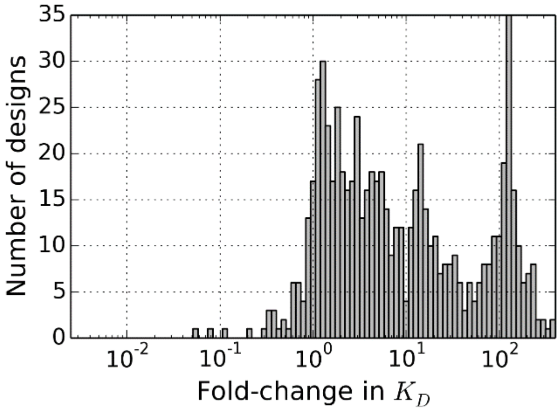

Sensor A MS2 OFF

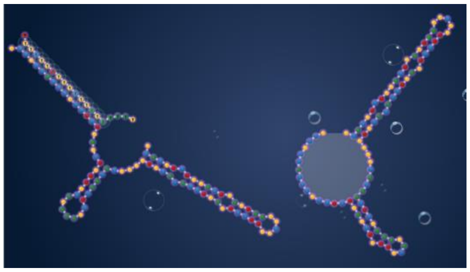

TB A before B 13 - Mod 12 by mat747, Eterna score 100

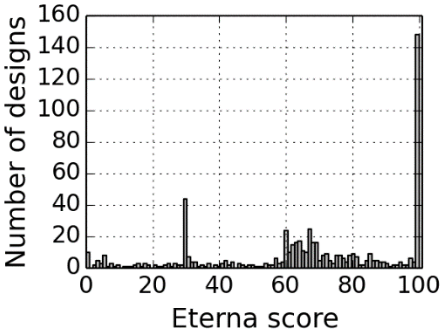

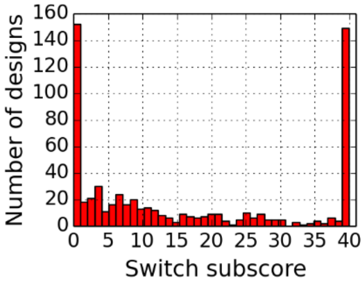

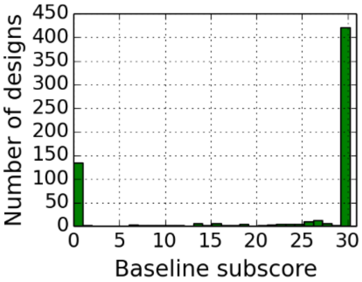

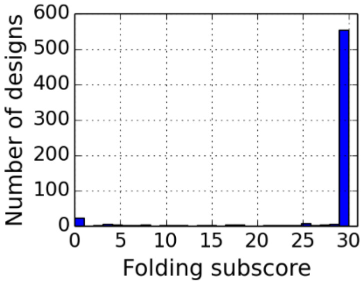

Dissociation constants (K_D)

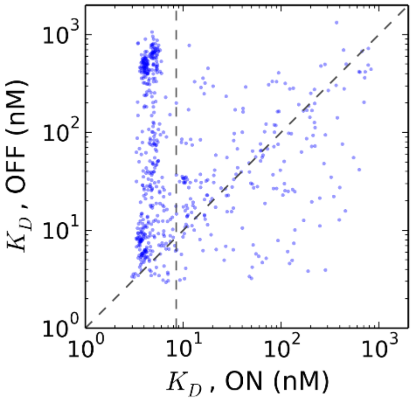

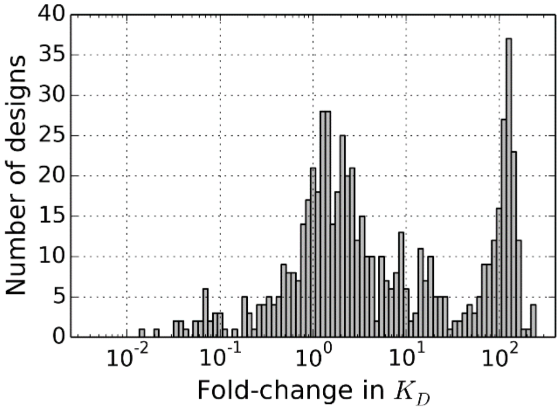

Sensor B MS2 ON

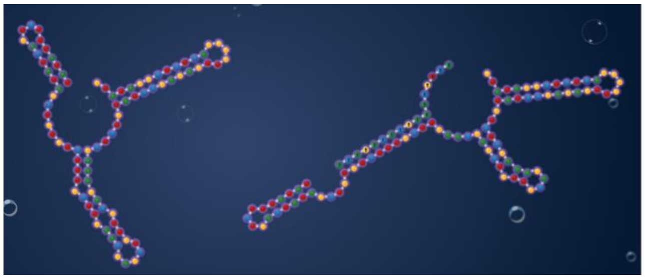

6345921 G30 Jieux mod #MUTATE_ONE by jandersonlee,
Eterna score 100

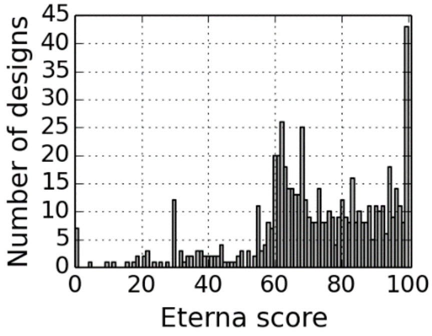

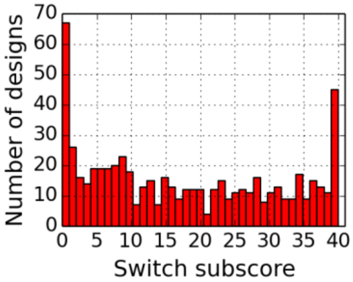

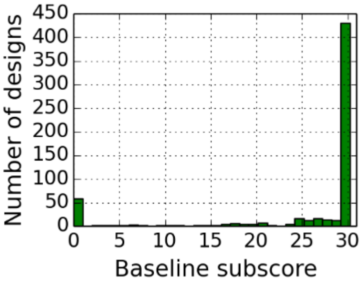

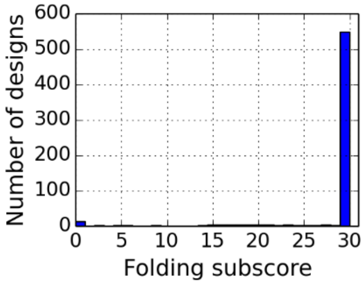

Dissociation constants (K_D)

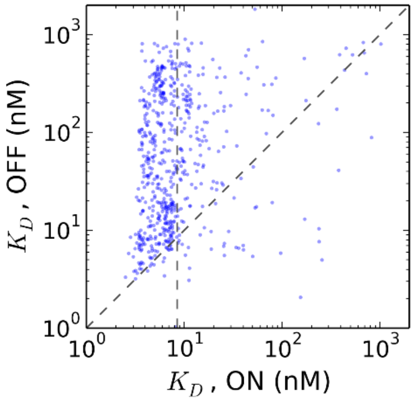

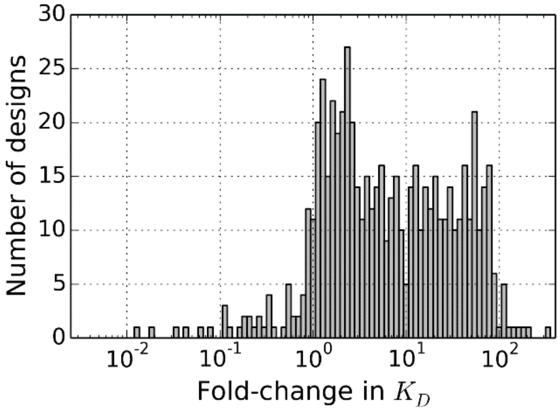

Sensor B MS2 OFF

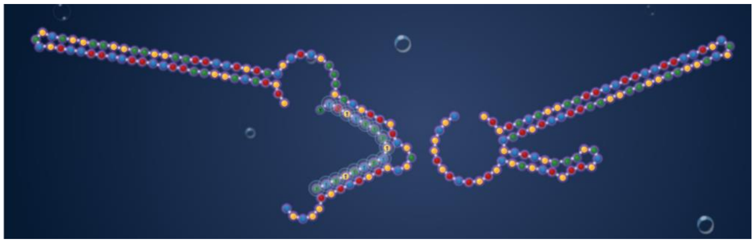

JL whbob mod - Variant 1 by Eli Fisker, Eterna score 100

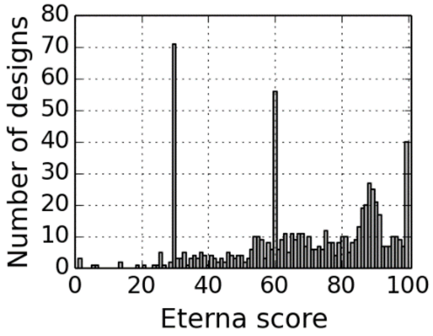

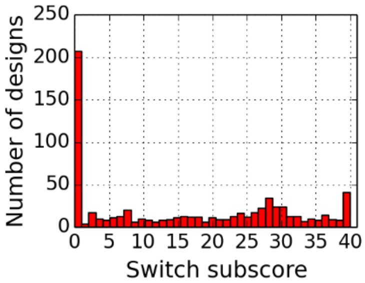

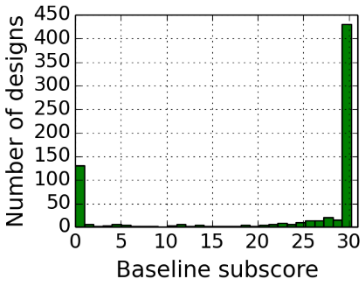

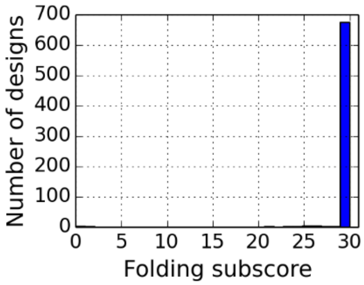

Dissociation constants (K_D)

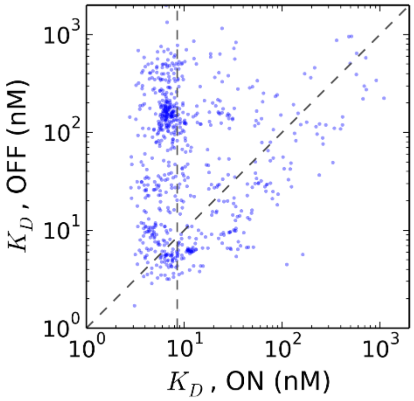

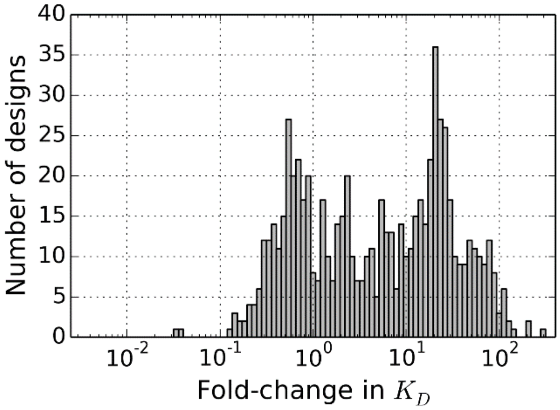

[A]/[B] with predefined binding sites

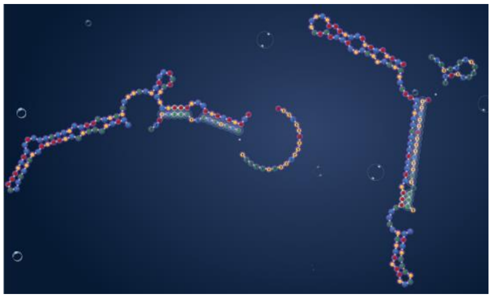

[A]/[B] wpbs malcolm mod by wateronthemoon, Eterna score 100

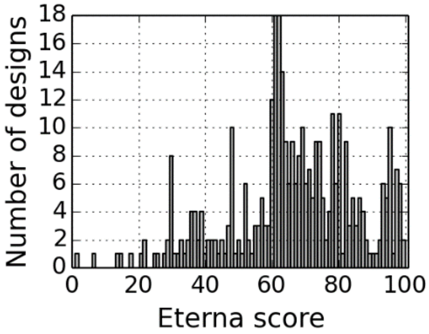

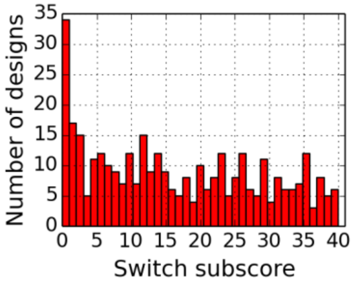

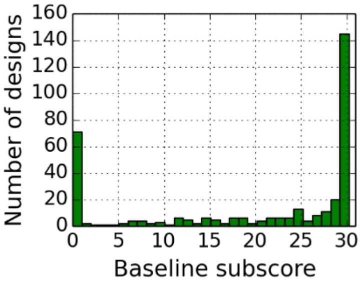

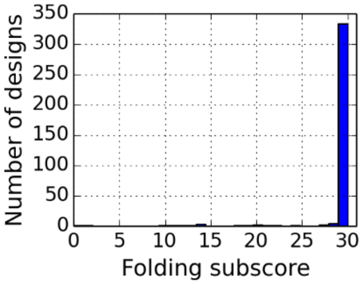

Dissociation constants (K_D)

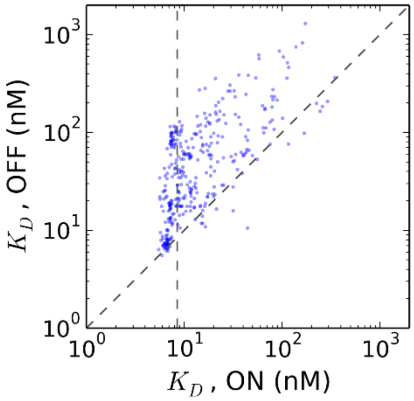

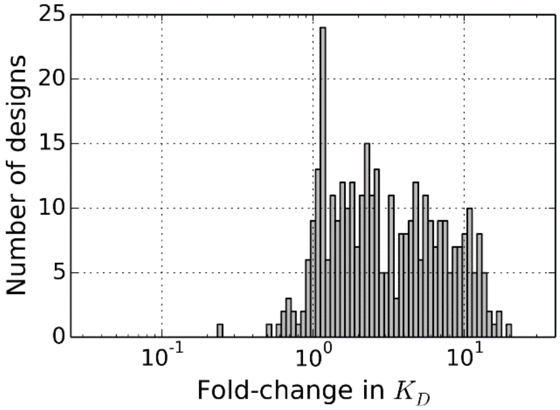

[A]/[B] with predefined binding sites (alternative)

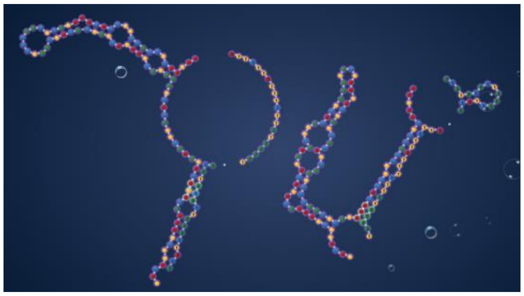

Salish JR Mod - D13 by mat747, Eterna score 99

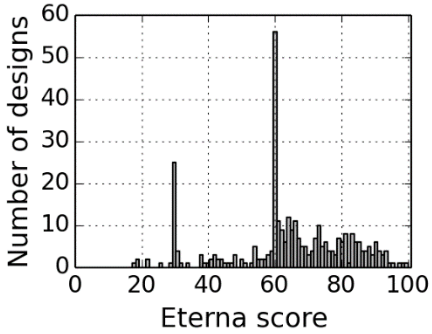

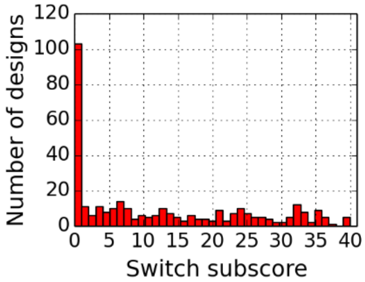

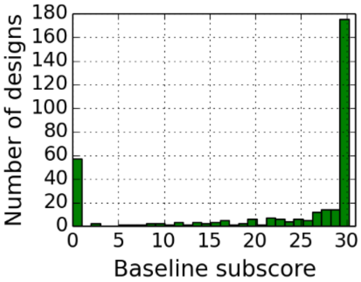

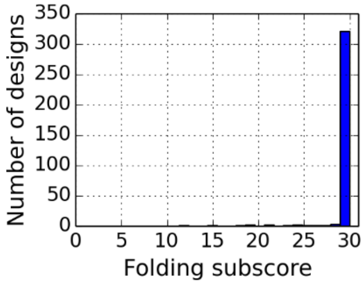

Dissociation constants (K_D)

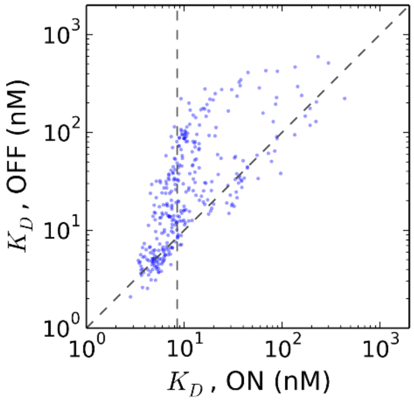

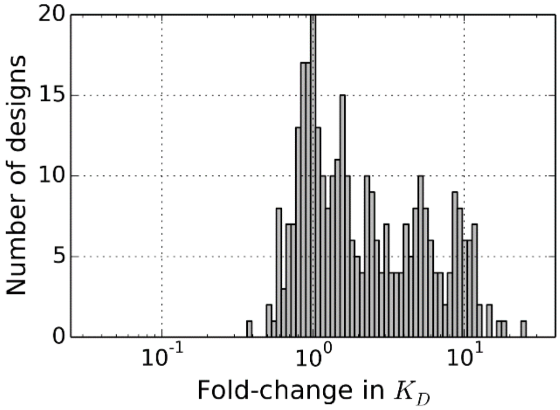

R2 (2-states model)

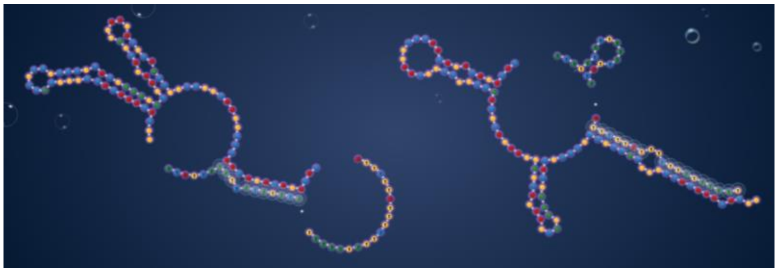

mod "6245377 G38 U24 JR mod#MUTATE_TWO" - Eli Fisker by sellena,
Eterna score 100

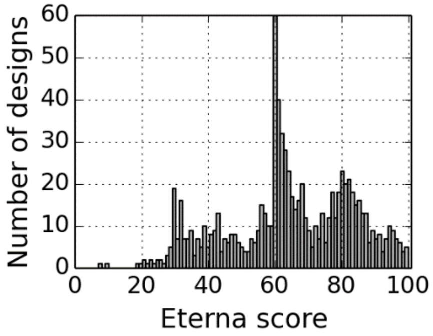

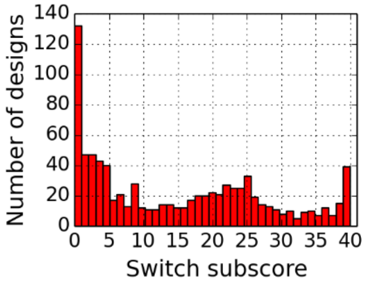

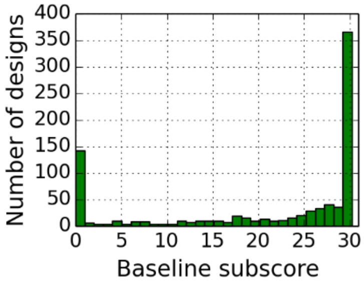

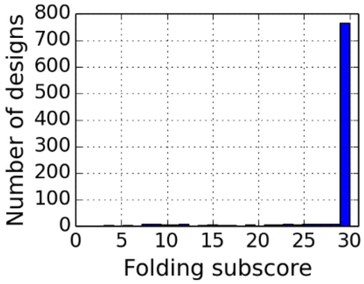

Dissociation constants (K_D)

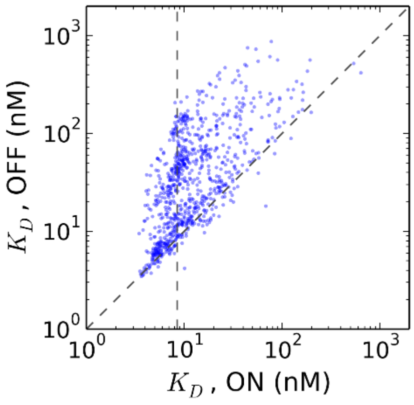

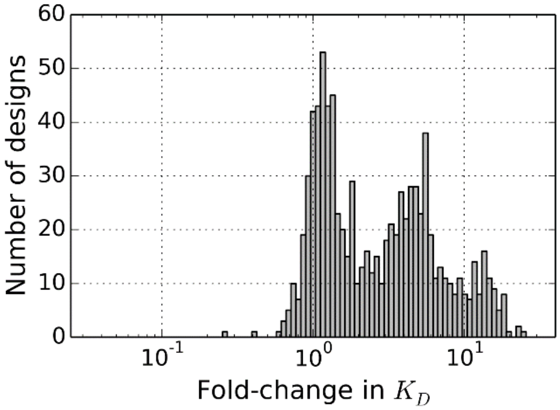

R3 (3-states model)

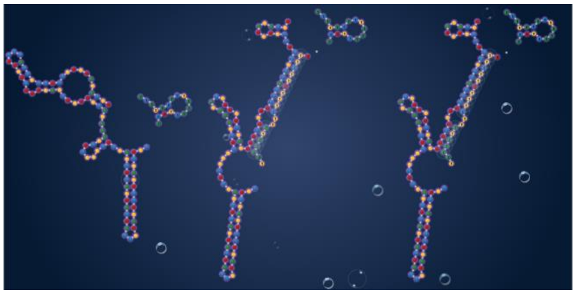

6248647 U11 jandersonlee mod #MUTATE_ONE by jandersonlee,
Eterna score 100

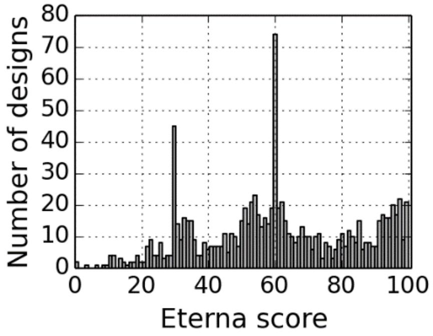

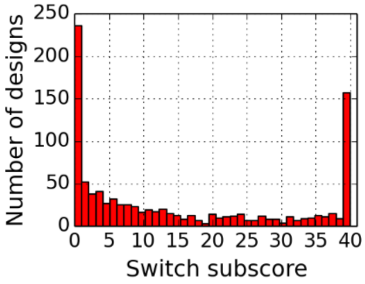

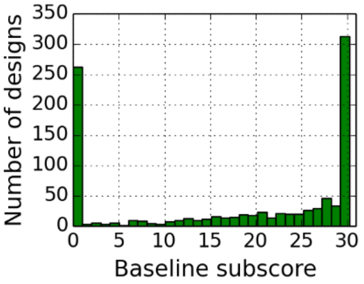

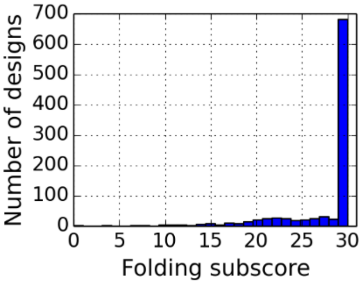

Dissociation constants (K_D)

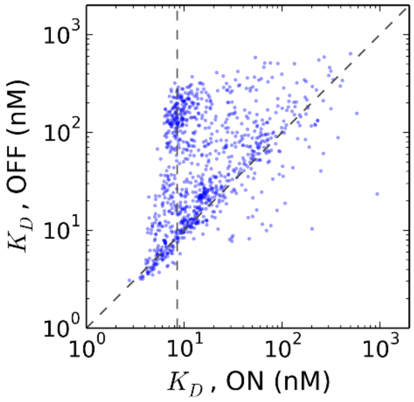

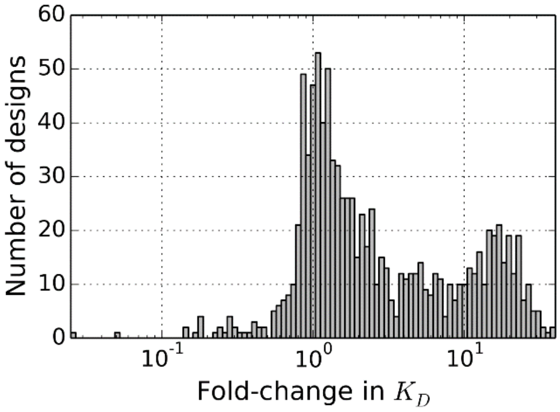

Supplement: Supplementary file 2 — Contains Supplementary Appendix: Eterna player resources (Eterna player resources also listed in Supplementary Table 4). Also contains the newsletters the Das lab shared with Eterna players after each design challenge. [file 41557_2025_1907_MOESM2_ESM.zip › supplemental_newsletter.zip!/supplemental_newsletter/Challenge_2_R103_ratio_results.pdf]
