# Supplementary material for: Compact RNA sensors for increasingly complex functions of multiple inputs
Source: Nat Chem. 2025 Nov 12;17(12):1839–52. doi: 10.1038/s41557-025-01907-8 (PMC12669046; doi:10.1038/s41557-025-01907-8)

Results for Eterna

OpenTB puzzles (first round, R104)

http://www.eternagame.org/web/lab/6892349/
http://www.eternagame.org/web/lab/6892318/

Johan Andreasson, 09/07/2016

Introduction

In the OpenTB labs, our goal is to design RNA molecules that can report on a disease signature by calculating a ratio of RNA concentrations. The input RNA molecules, here referred to as A, B, and C, were identified to be a predictor for tuberculosis (http://khatrilab.stanford.edu/tb). Tuberculosis (TB) is present if the ratio $[A][B]/[C]^2$ is ¼ or higher, where [A], [B] and [C] are our RNA input concentrations.

Getting started puzzles: Let's get started!

As in previous Eterna rounds, several puzzles were presented to introduce the problem. In the 'Getting started' puzzles, we design switches that act as sensors of the three different input sequences by changing the binding to a short 10-nt RNA molecule (the Reporter). For switches that turn on in the presence of input RNA molecules (**Sensor A (GBP5) – RIRI**, **Sensor B (DUSP3) – RIRI**, and **Sensor C (KLF2) – RIRI**), the Reporter should bind when the input is present at a concentration of 100 nM but not at 0 nM. This 'RNA in/RNA in' mechanism is abbreviated RIRI in the puzzle names. Conversely, for switches that turn off (**Sensor A (GBP5) – RIRO**, **Sensor B (DUSP3) – RIRO**, and **Sensor C (KLF2) – RIRO**), the reporter should bind at an input oligo concentration of 0 nM, but not at 100 nM. We refer to these designs as 'RNA in/RNA out', or RIRO. In practice, we measure binding of the reporter across a large concentration range and quantify the quality of the switch through the change in the apparent dissociation constant, K_D, between the two conditions, just as we did in previous rounds with the MS2 or RNA in/RNA out switches.

OpenTB puzzles: This is it!

To break down the $[A][B]/[C]^2$ expression into smaller modules, both for practice and for finding strategies that can be extended to the full problem, we introduced several more puzzles. The **A (AND) B** puzzles senses the presence of two input oligos, just like the AND gates in our previous Logic Gates puzzles. Only when both the inputs are present at a concentration of 100 nM should the designs either bind the fluorescent Reporter (**A (AND) B – RI**) or not bind a Reporter (**A (AND) B – RO**).

In contrast to the AND gates, where the inputs are either present at high concentrations or completely absent, the next puzzles aimed at reporting a ratio of two concentrations. These puzzles are similar to those in the [A] / [B] challenge but here we use the fluorescent Reporter RNA as a readout instead of an MS2 protein. In total, four puzzles were introduced. In two of these (**[A]/[C] – INC** and **[B]/[C] – INC**), the reporter RNA should bind if the ratio is high (e.g. [[A]/[C] >1/4). In practice, we check the binding for two concentration ratios. In the first, [A]=[C]=100 nM (e.g., ratio [A]/[C] = 1) and in the second, [A]=5 nM and

[C]=100 nM (ratio [A]/[C] = 1/20). The other two puzzles (**[A]/[C] – DEC** and **[B]/[C] – DEC**), are identical, except that they should instead bind for low ratios.

The full TB signature is addressed in the two final puzzles: **[A]*[B]/[C]² INC** and **[A]*[B]/[C]² DEC**. In the first puzzle (**[A]*[B]/[C]² INC**, the designs should bind the 10-nt Reporter RNA oligo only when the ratio $[A][B]/[C]^2 \geq 1/4$. The second puzzle (**[A]*[B]/[C]² DEC**) is identical, except that the Reporter should not bind when the ratio is high, instead it should bind when the ratio is low ($[A][B]/[C]^2 \leq 1/4$. The ratio is a continuous variable but in the Eterna game we limit ourselves to show four different states:

Condition	A (nM)	B (nM)	C (nM)	$[A][B]/[C]^2$	State
**1**	100	0	100	0	OFF
**2**	0	100	100	0	OFF
**3**	50	50	300	1/36	OFF
**4**	50	50	100	1/4	ON

Scoring of puzzles

Fold-change in K_D

As for our previous switch puzzles, the **fold-change in K_D** is probably the best single metric for good switching (see also Scoring of riboswitches in Eterna). For a simple binding curve, the fluorescent signal, *F*, from a reporter, *R*, binding to a design is $F[R] = \frac{[R]}{[R]+K_D}$, where the apparent dissociation constant, K_D, is the concentration at which half of the maximum signal is observed. This number is an indicator of how strong the apparent interaction is between the reporter and the design. For switches, the goal is to modulate this strength using the input oligos. The larger the change is in K_D between the ON and OFF-states the better the design is working. As we like to think of K_D in log-space, and to normalize for the binding in the ON-state, we use the ratio of the K_D in the OFF and ON-states (i.e., $K_{D,\,OFF}$ / $K_{D,\,ON}$) as a metric for the change.

For simple switches (**Sensor A (GBP5) – RIRI**, **Sensor B (DUSP3) – RIRI**, **Sensor C (KLF2) – RIRI**, **Sensor A (GBP5) – RIRO**, **Sensor B (DUSP3) – RIRO**, and **Sensor C (KLF2) – RIRO**), there is only one ON state and one OFF state (0 and 100 nM input oligo). However, for the other puzzles there are multiple state in either the ON or OFF states, or both. To account for this, we report the fold-change between the worst results for each state, i.e., the highest K_D in the ON state and the lowest K_D in the OFF state.

Note: Because to the large number of conditions and experimental variations, all K_D values have been normalized to the average for a control constructs consisting of the perfect complement to the Reporter oligo. The average was 7.5 nM for the controls (min 4.5 nM for 100 nM A, max 12 nM for 100 nM A and C). The numbers in the figure for each design represent these normalized values but the raw experimental data are plotted in the graph.

Global fold-change in K_D

Each puzzle is represented by a few states in the game, but a ratio such as $[A][B]/[C]^2$ is a continuous variable. Because we test many different puzzles with the same experimental pipeline, we also have the opportunity to map the behavior of the player designs for more conditions than the ones specified in the game. The global fold-change in K_D that we also report in the spreadsheets and in the figures (switch graphs) refers to the full $[A][B]/[C]^2$ ratio across all 9 well-defined conditions for the first round of the openTB challenge. A positive Global fold-change means that the design is good and the higher the ratio the better. This metric allows us to both better evaluate good results from each **[A]*[B]/[C]²** puzzles and to spot designs for other puzzles that happen to work well for the full $[A][B]/[C]^2$ signature.

Below is the complete list of conditions tested in this round. Of the twelve conditions, only the last nine have a well defined $[A][B]/[C]^2$ ratio.

Condition	A (nM)	B (nM)	C (nM)	$[A][B]/[C]^2$	State
**1**	0	0	0	undefined	-
**2**	100	0	0	undefined	-
**3**	0	100	0	undefined	-
**4**	0	0	100	0	OFF
**5**	100	100	0	∞	ON
**6**	5	0	100	0	OFF
**7**	100	0	100	0	OFF
**8**	0	5	100	0	OFF
**9**	0	100	100	0	OFF
**10**	50	50	300	1/36	OFF
**11**	50	50	100	1/4	ON
**12**	200	200	300	4/9	ON

Eterna score

As for all previous puzzles, the designs were also given an Eterna score is between 0 and 100 that is a combination of three subscores for each puzzle (see also Scoring of riboswitches in Eterna).

The **Folding subscore** is determined by the intensity (F_{max}) of the ON state. For an F_{max} above 0.7 the score is 30, below 0.3 the score is 0, and in between the score increases linearly with the F_{max}.

The **Baseline subscore** is based on the K_D of the ON-state. The score is 100 for for K_D < 15 nM (2 x $K_{D,\, reporter}$), 0 for K_D > 45 nM (6 x $K_{D,\, MS2}$), and decreases linearly in between.

The **Switch score** is based on the K_D of the ON-states and the OFF-states. The score is proportional to the logarithm of the fold-change in K_D between the ON- and OFF-states, i.e. $subscore \sim \log\left(\frac{K_{D,OFF}}{K_{D,ON}}\right)$. The subscore is 0 if the ratio is less than 1 and the maximum score of 40 is reached when the ratio is above

- 75 (**Sensor A (GBP5) – RIRI**, **Sensor B (DUSP3) – RIRI**, **Sensor C (KLF2) – RIRI**, **Sensor A (GBP5) – RIRO**, **Sensor B (DUSP3) – RIRO**, **Sensor C (KLF2) – RIRO, A (AND) B – RI**, and **A (AND) B – RO**)
- 15 (**[A]/[C] – INC**, **[B]/[C] – INC**, **[A]/[C] – DEC**, and **[B]/[C] – DEC**)
- 9 (**[A]*[B]/[C]² INC** and **[A]*[B]/[C]² DEC.**

Sensor A (GBP5) - RIRI

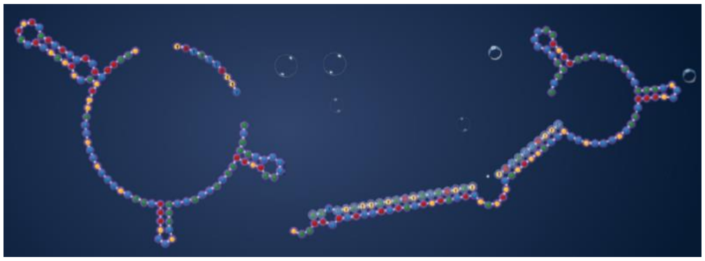

Sensor A (GBP5)-RIRI 3rd wateronthemoon, Eterna score 100

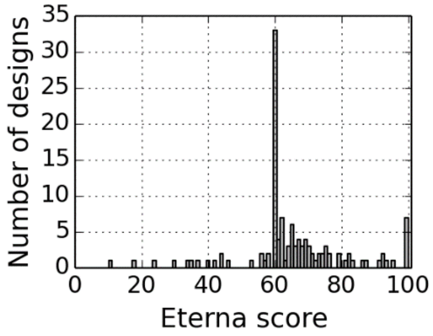

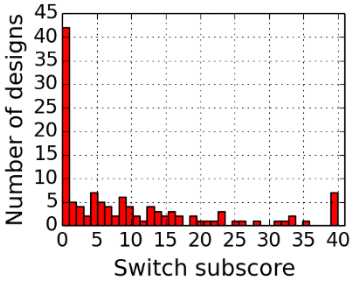

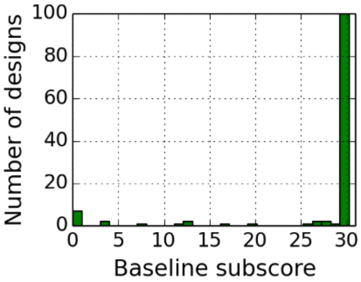

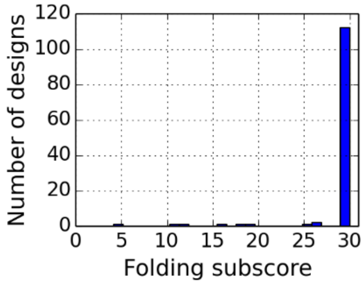

Dissociation constants (K_D)

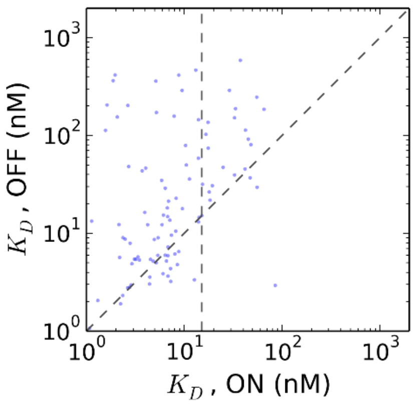

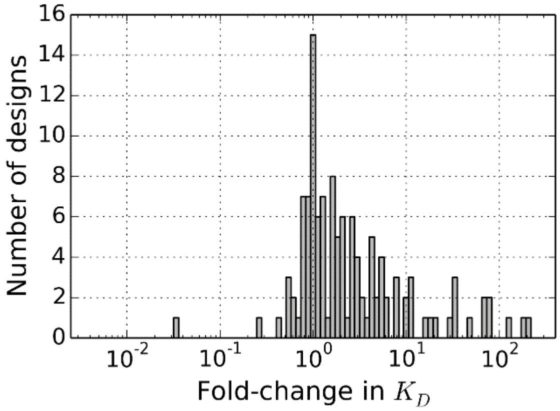

Sensor A (GBP5) - RIRO

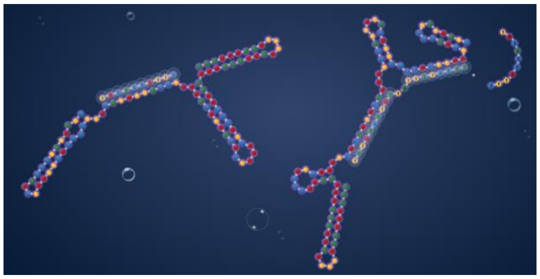

SockTatersMod - Jieux - Sensor A (GBP5) - RIRO by Jieux, Eterna score 100

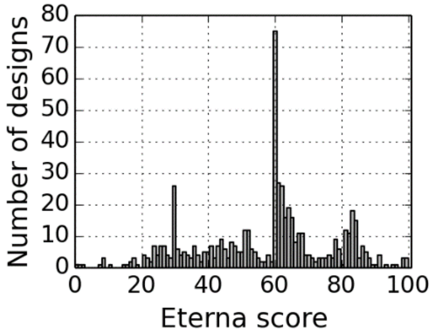

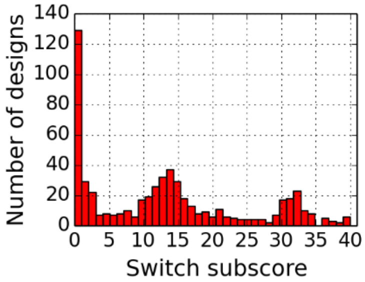

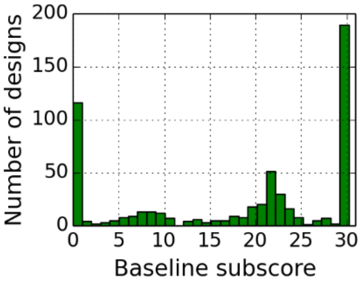

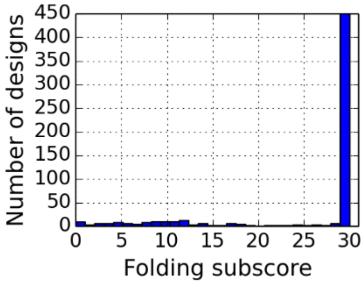

Dissociation constants (K_D)

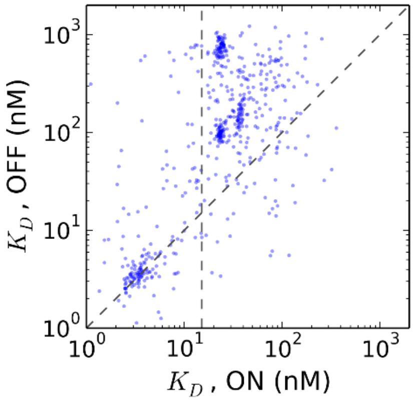

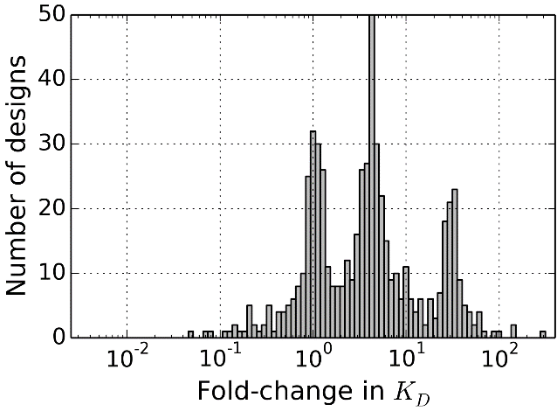

Sensor B (DUSP3) - RIRI

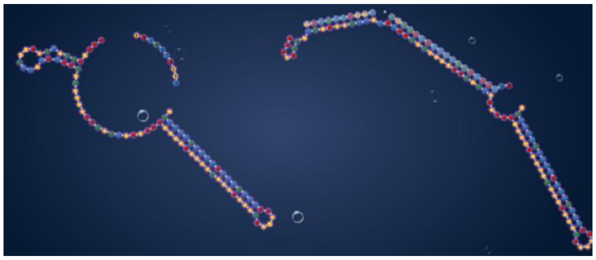

Whbob mod 1 by Eli Fisker,
Eterna score 81

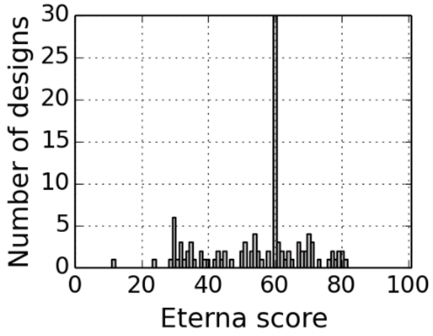

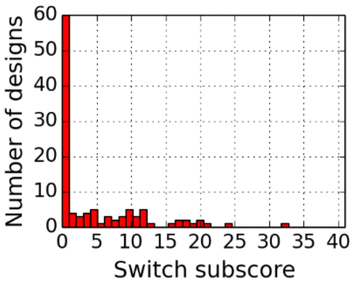

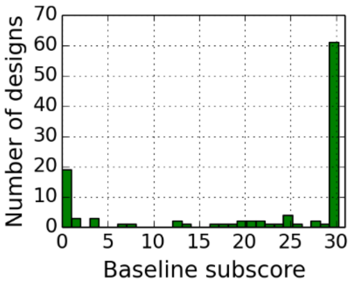

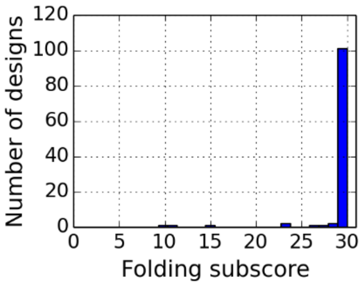

Dissociation constants (K_D)

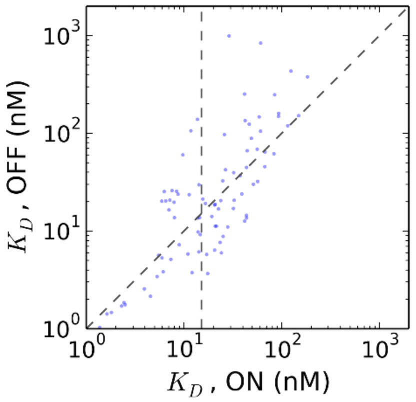

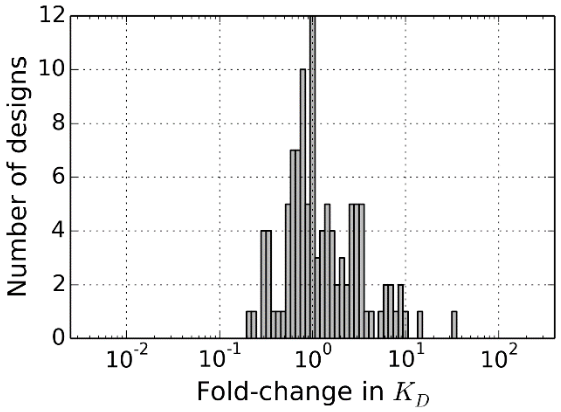

Sensor B (DUSP3) - RIRO

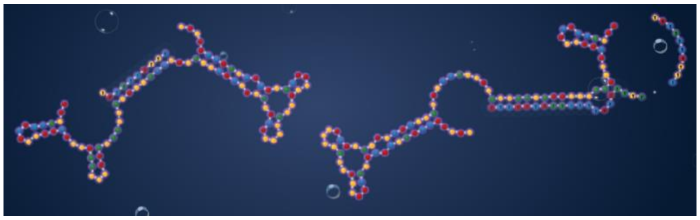

Mac's II by MacSousa, Eterna score 96

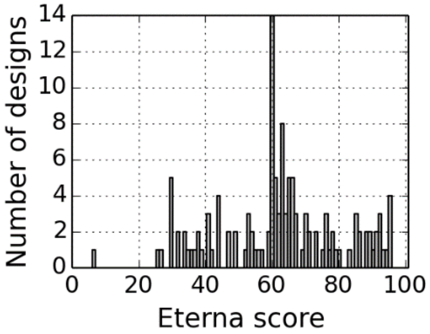

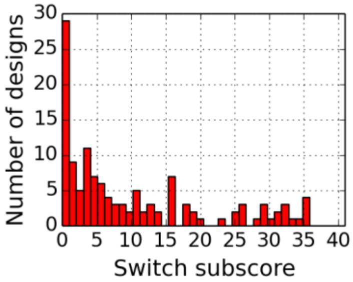

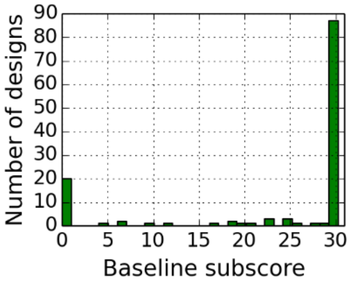

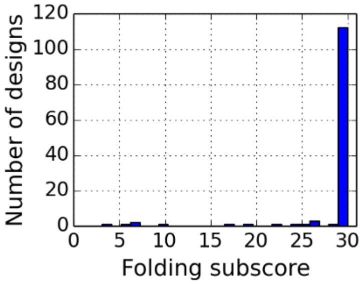

Dissociation constants (K_D)

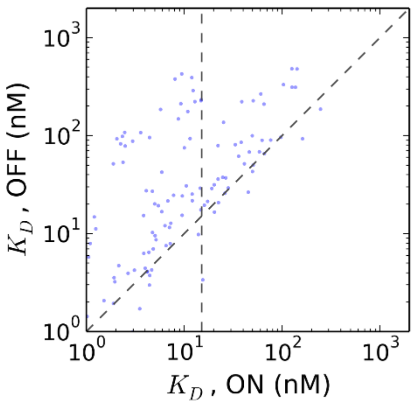

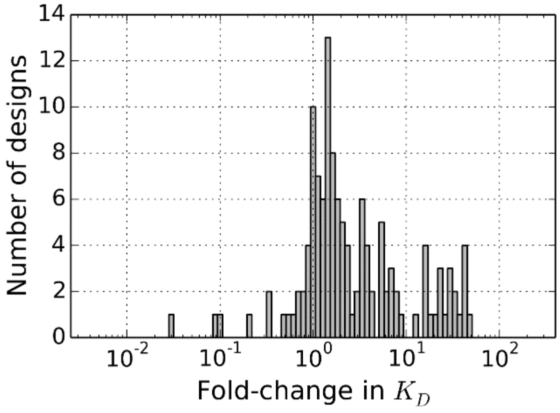

Sensor C (KLF2) - RIRI

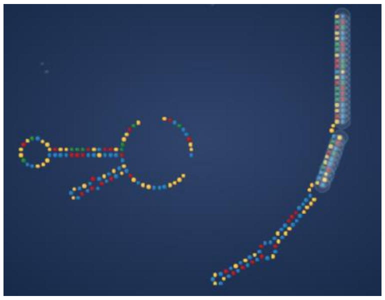

Zipper by ererexiue, Eterna score 100

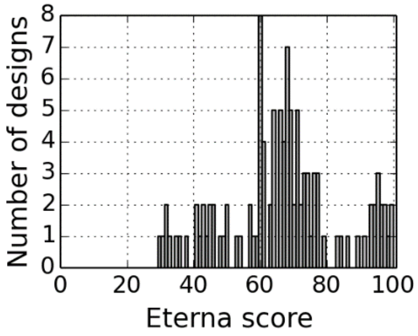

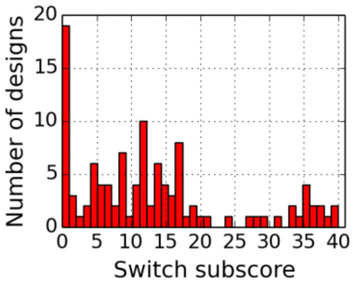

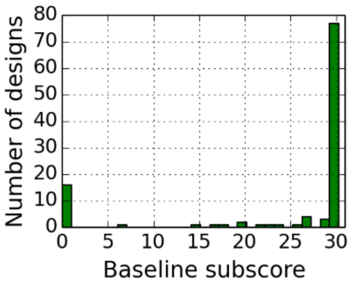

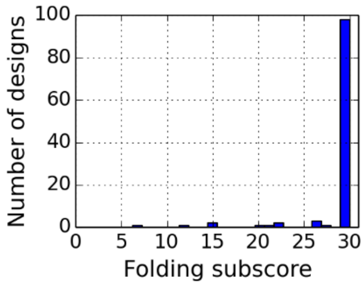

Dissociation constants (K_D)

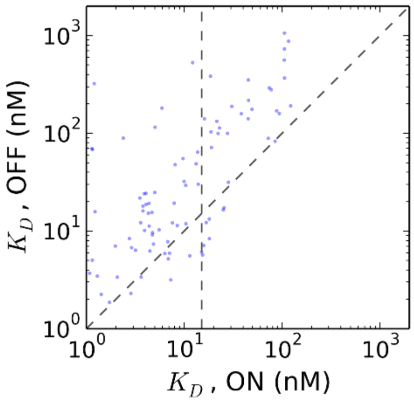

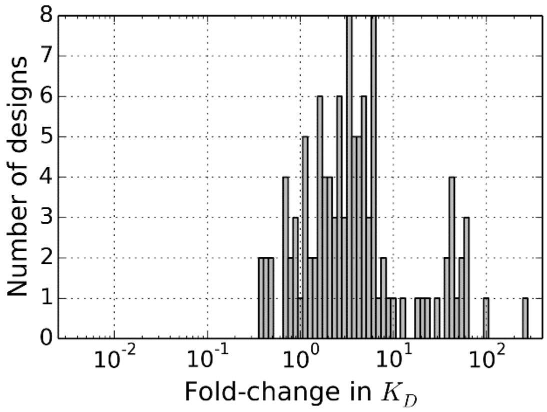

Sensor C (KLF2) - RIRO

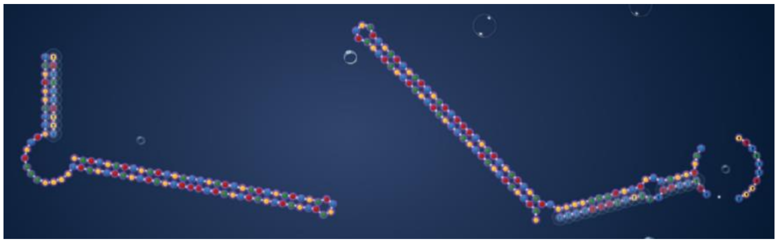

Sensor C #1 by Gracier, Eterna score 100

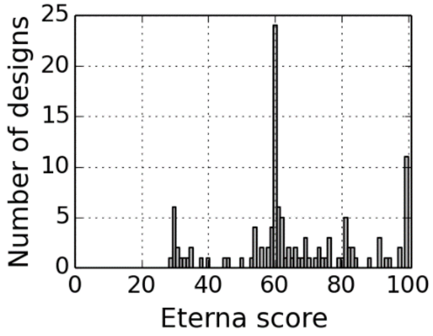

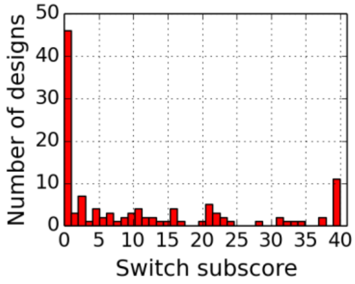

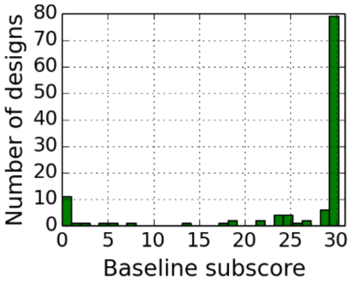

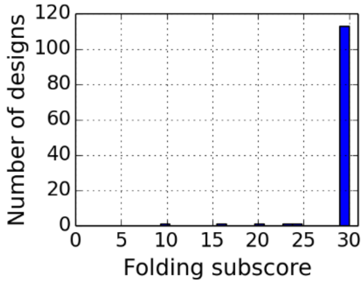

Dissociation constants (K_D)

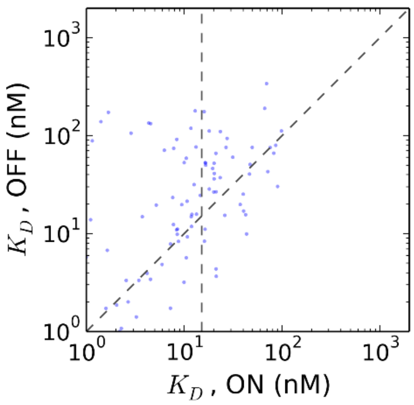

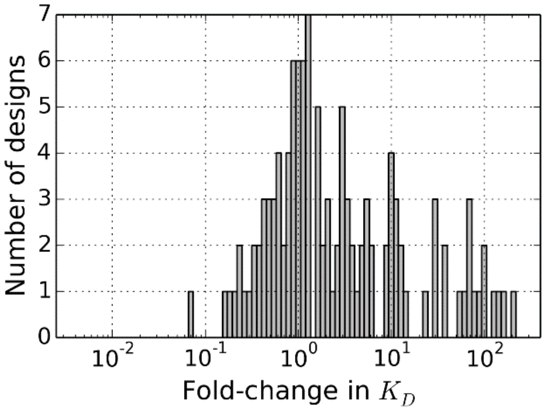

A (AND) B - RI

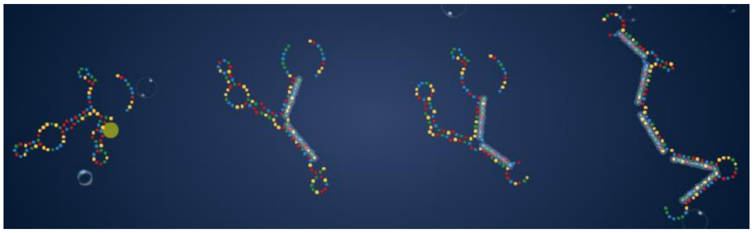

Outer site lock + C suffix mod 2 by atanas.atanasov, Eterna score 87

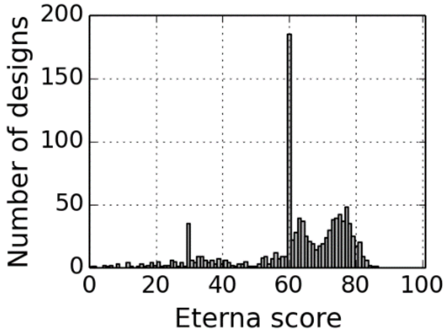

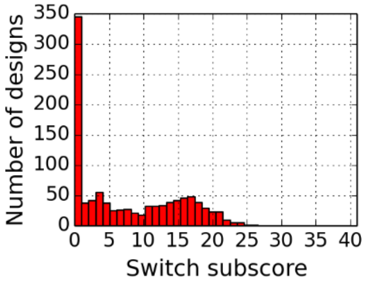

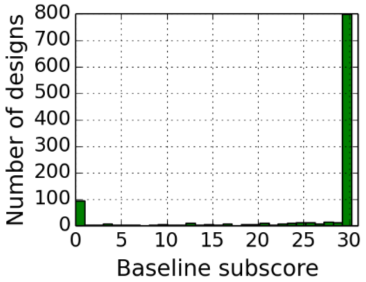

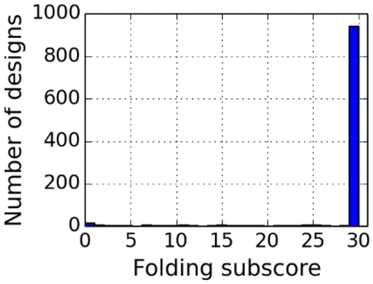

Dissociation constants (K_D)

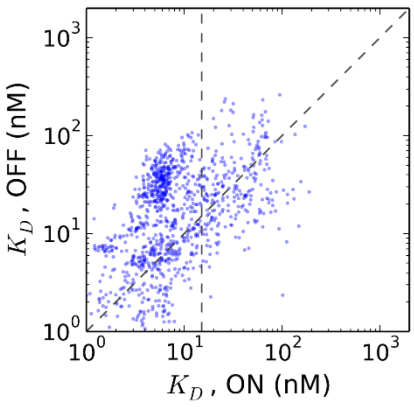

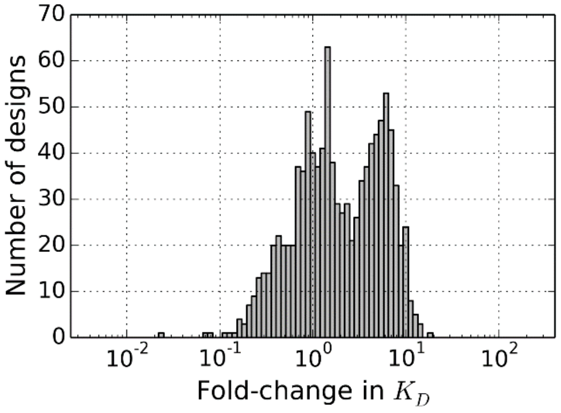

A (AND) B - RO

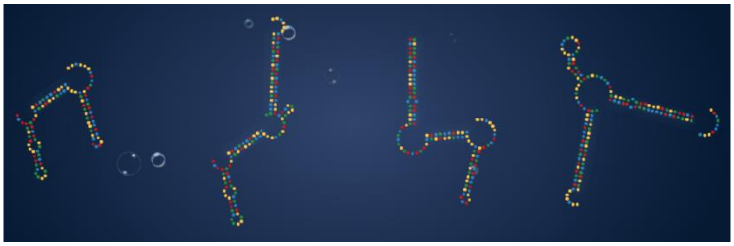

Reversed Worzeise style 9 by Eli Fisker, Eterna score 81

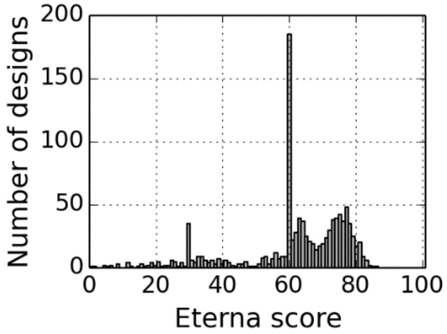

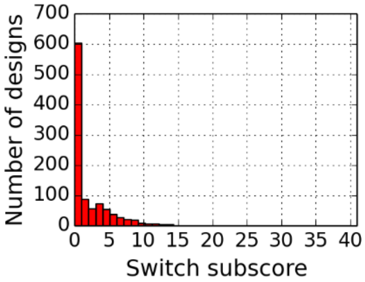

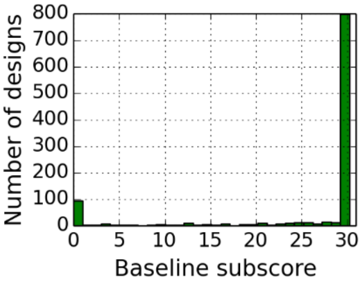

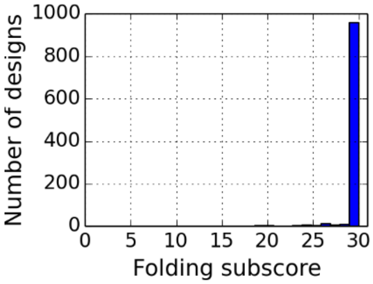

Dissociation constants (K_D)

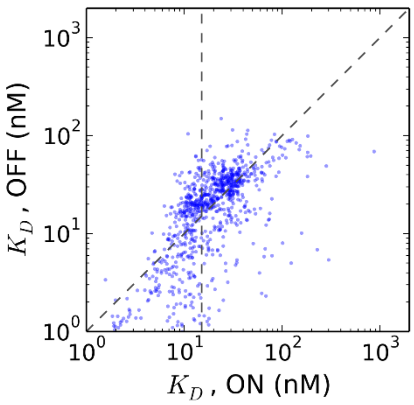

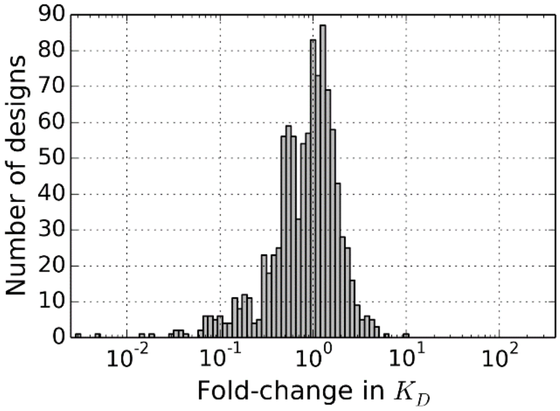

[A]/[C] - INC

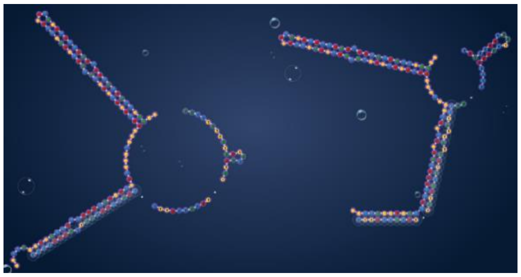

HarryS mod 7 mutants #MUTATE_ONE 0 U55 anon mod
by Eli Fisker, Eterna score 100

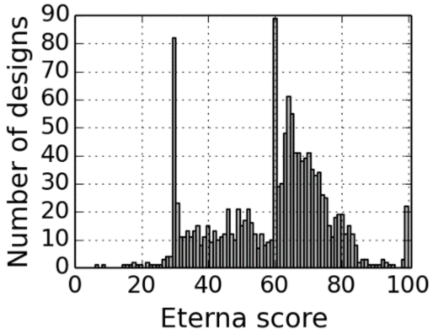

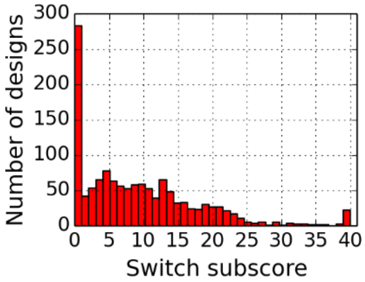

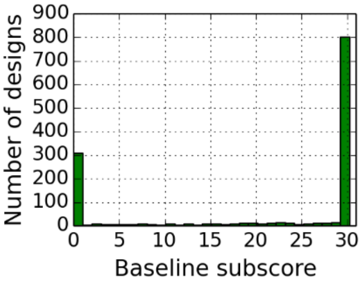

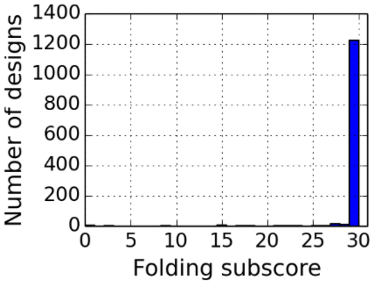

Dissociation constants (K_D)

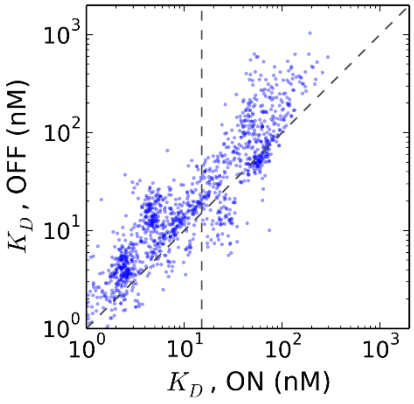

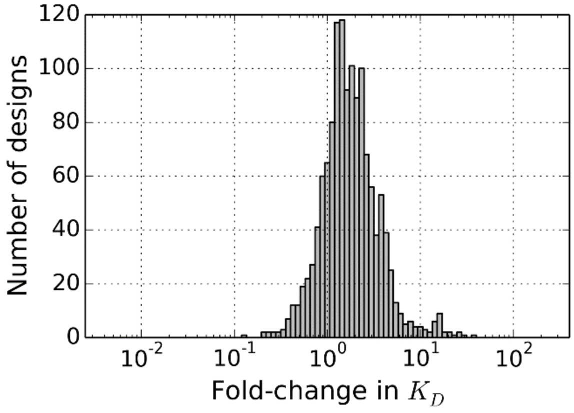

[A]/[C] - DEC

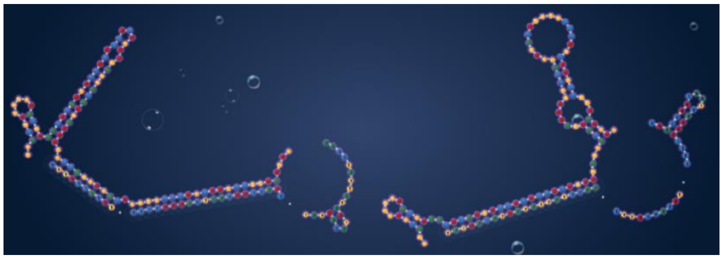

Rocketdog 42 mod 3 by Eli Fisker, Eterna score 100

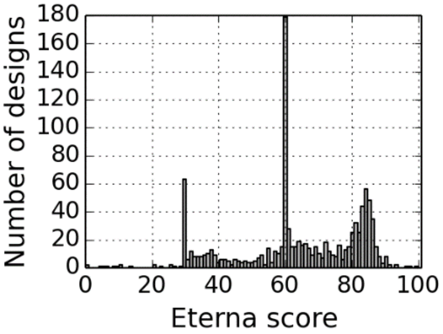

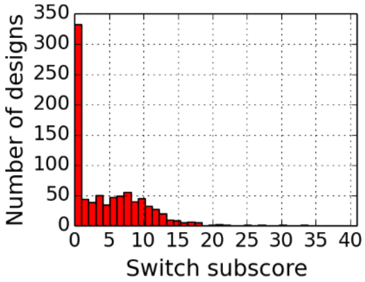

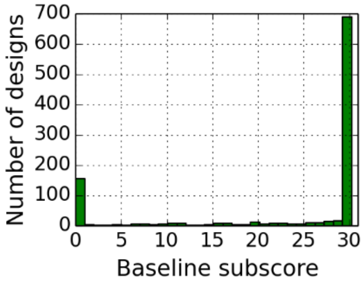

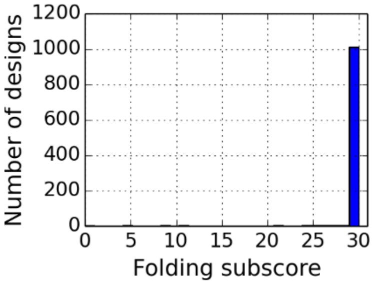

Dissociation constants (K_D)

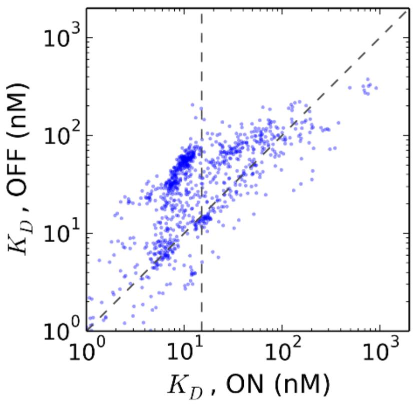

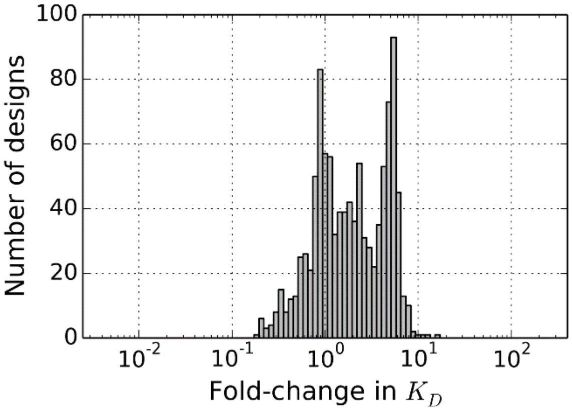

[B]/[C] - INC

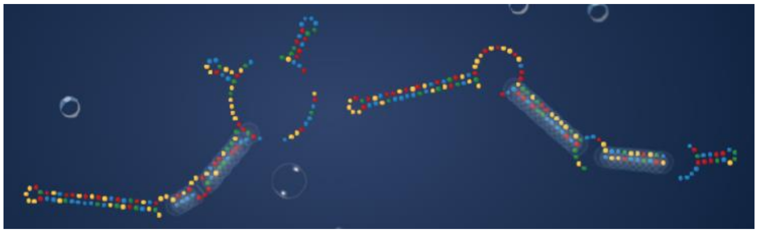

B/C INC J1 by joy45, Eterna score 100

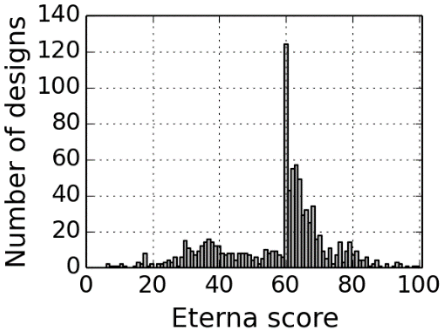

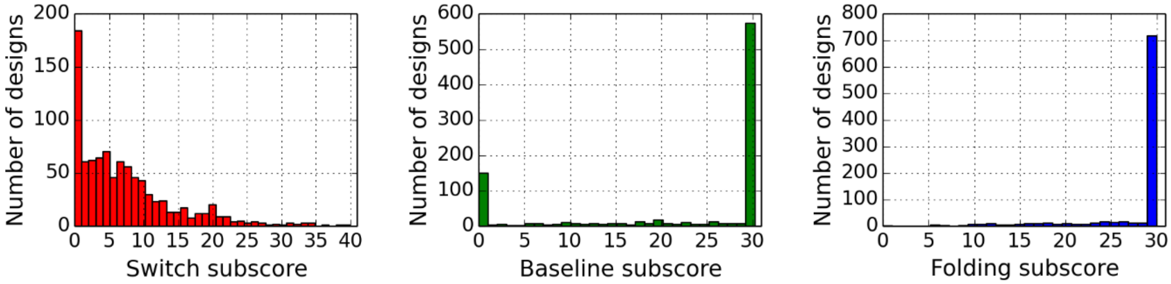

Dissociation constants (K_D)

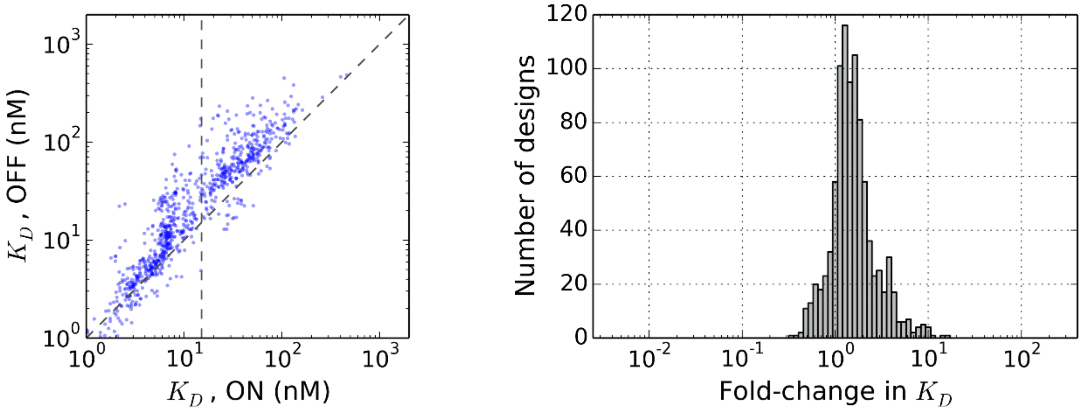

[B]/[C] - DEC

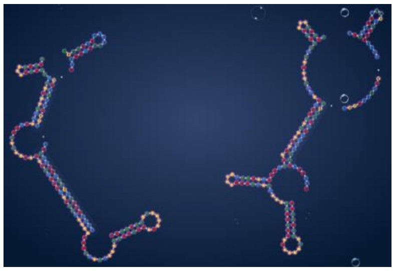

Key for B/C by Menosfree, Eterna score 86

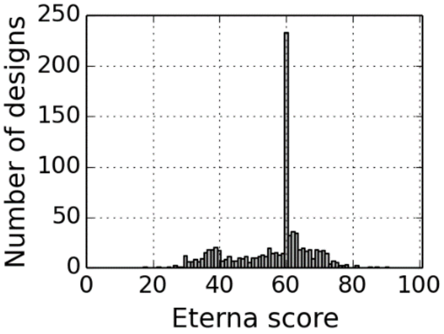

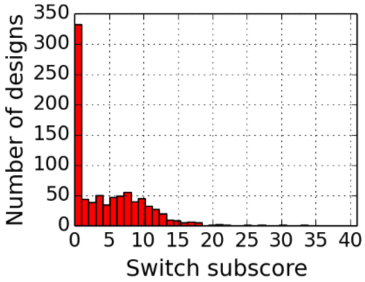

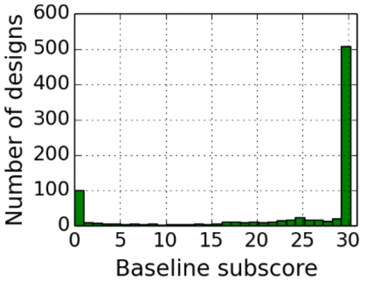

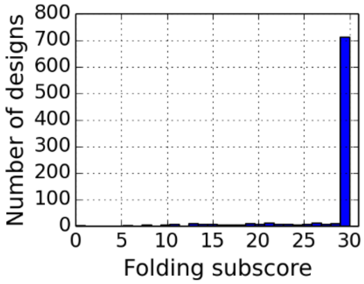

Dissociation constants (K_D)

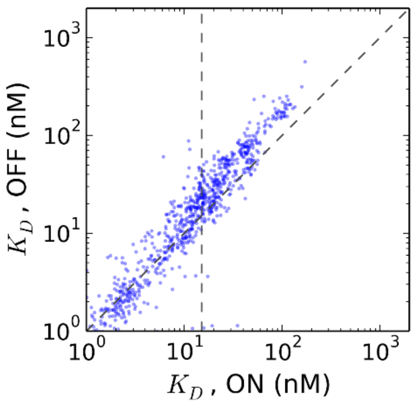

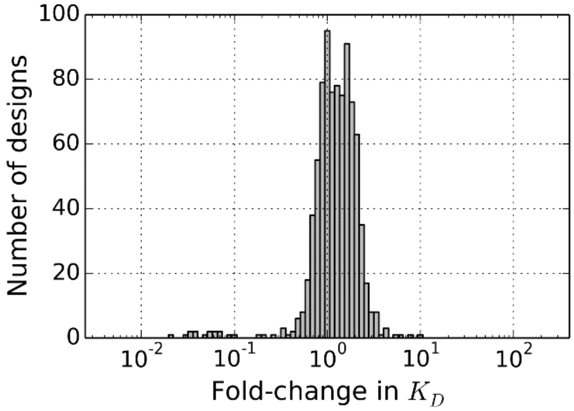

[A]*[B]/[C]² INC

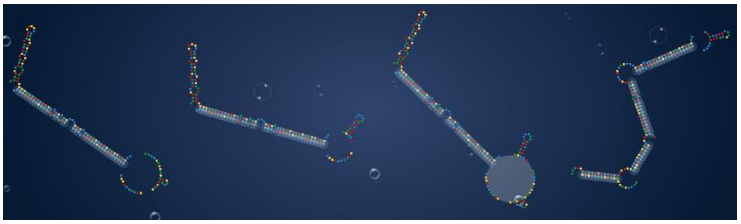

A*B/C^2 INC 53 by dl2007, Eterna score 72

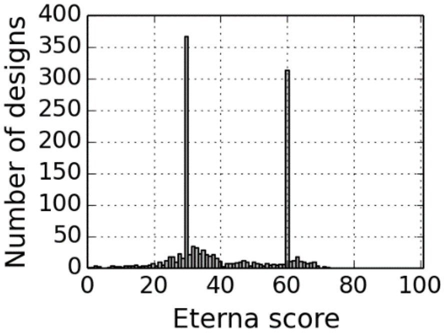

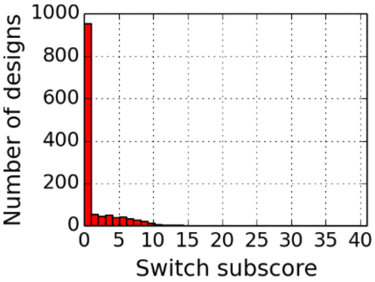

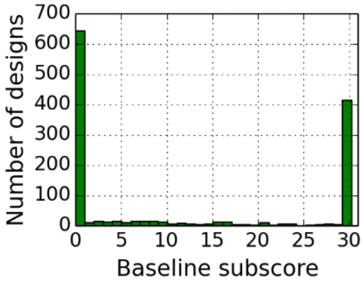

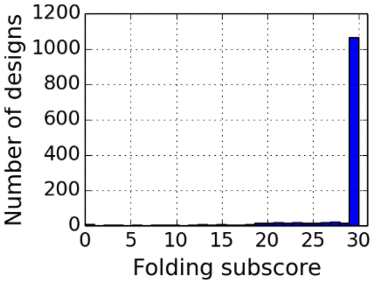

Dissociation constants (K_D)

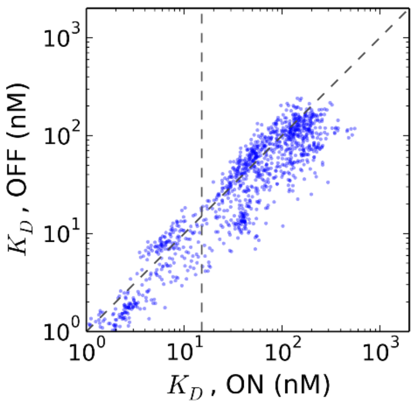

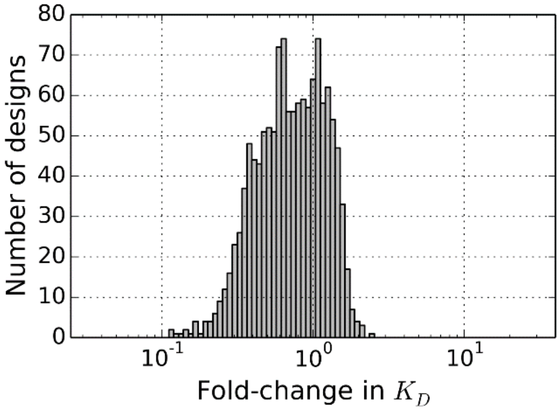

[A]*[B]/[C]² DEC

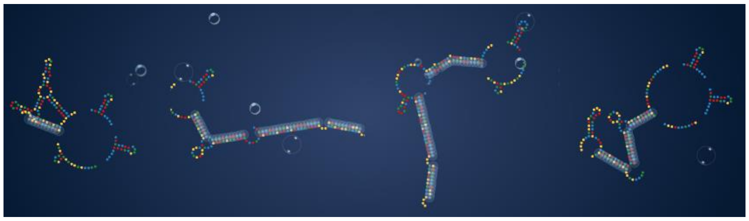

Fusion style 1.41 by Eli Fisker, Eterna score 73

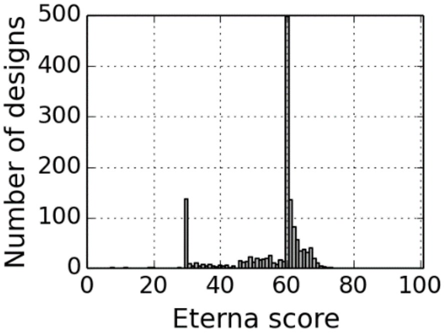

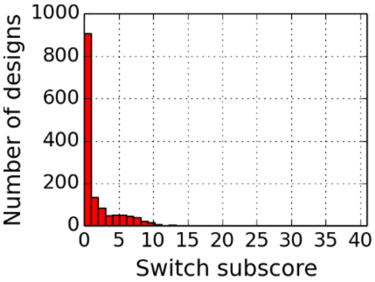

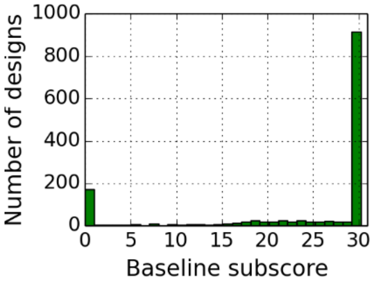

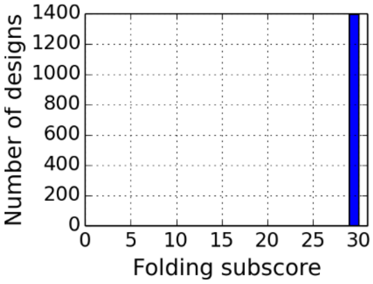

Dissociation constants (K_D)

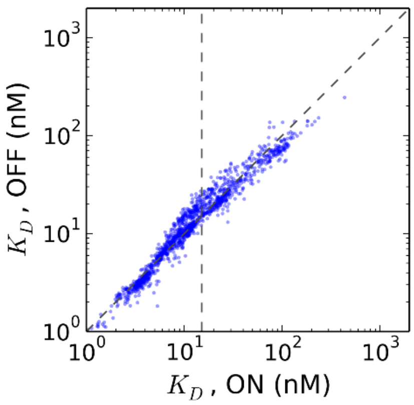

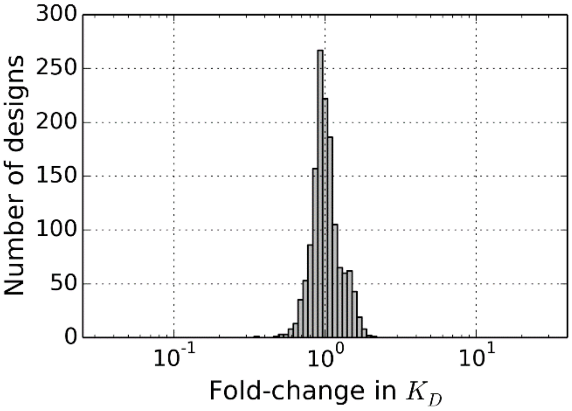

Supplement: Supplementary file 2 — Contains Supplementary Appendix: Eterna player resources (Eterna player resources also listed in Supplementary Table 4). Also contains the newsletters the Das lab shared with Eterna players after each design challenge. [file 41557_2025_1907_MOESM2_ESM.zip › supplemental_newsletter.zip!/supplemental_newsletter/Challenge_3_R104_openTB_results.pdf]
