# Supplementary material for: Compact RNA sensors for increasingly complex functions of multiple inputs
Source: Nat Chem. 2025 Nov 12;17(12):1839–52. doi: 10.1038/s41557-025-01907-8 (PMC12669046; doi:10.1038/s41557-025-01907-8)

Results for Eterna

OpenTB puzzles (2nd round, R105)

http://www.eternagame.org/web/lab/7113338/
http://www.eternagame.org/web/lab/7113227/

Johan Andreasson, 12/07/2016

Introduction

In the OpenTB labs, our goal is to design RNA molecules that can report on a disease signature by calculating a ratio of RNA concentrations. The input RNA molecules, here referred to as A, B, and C, can be used to diagnose tuberculosis (http://khatrilab.stanford.edu/tb). Tuberculosis (TB) is present if the ratio $[A][B]/[C]^2$ is 1/16 or higher, where [A], [B] and [C] are our RNA input concentrations.

The results for the second round were very positive! Players designed several solutions that were correct across all sixteen tested conditions.

The sequences in this round (R105) were chosen from a different segment of the short RNA signature than those in the previous round so the sequences in the game were different. Based on player suggestions, we also used a longer fluorescent RNA oligo reporter (14 nt instead of 10 nt) that also had a different sequence composition. The explanation of the puzzles below are largely identical to those in the summary of results for R104, with an added section on how to interpret the switch graphs.

Getting started puzzles: Let's get started!

As in previous Eterna rounds, several puzzles were presented to introduce the problem. In the 'Getting started' puzzles, we design switches that act as sensors of the three different input sequences by changing the binding to a short 14-nt RNA molecule (the Reporter). For switches that turn on in the presence of input RNA molecules (**Sensor A (GBP5) – RIRI**, **Sensor B (DUSP3) – RIRI**, and **Sensor C (KLF2) – RIRI**), the Reporter should bind when the input is present at a concentration of 100 nM but not at 0 nM. This 'RNA in/RNA in' mechanism is abbreviated RIRI in the puzzle names. Conversely, for switches that turn off (**Sensor A (GBP5) – RIRO**, **Sensor B (DUSP3) – RIRO**, and **Sensor C (KLF2) – RIRO**), the reporter should bind at an input oligo concentration of 0 nM, but not at 100 nM. We refer to these designs as 'RNA in/RNA out', or RIRO. In practice, we measure binding of the reporter across a large concentration range and quantify the quality of the switch through the change in the apparent dissociation constant, K_D, between the two conditions, just as we did in previous rounds with the MS2 or RNA in/RNA out switches.

OpenTB puzzles: This is it!

To break down the $[A][B]/[C]^2$ expression into smaller modules, both for practice and for finding strategies that can be extended to the full problem, we introduced several more puzzles. The **A * B** puzzles senses the concentration of two input oligos. They are similar to the AND gates in the previous round but instead of sensing the presence or absence of inputs, the designs should respond to different

concentrations: 5 nM or 100 nM. Only when both the inputs are present at a concentration of 100 nM should the designs either bind the fluorescent Reporter (**A * B – RI**) or not bind a Reporter (**A * B – RO**).

The next puzzles aimed at reporting a ratio of two concentrations. In total, four puzzles were introduced. In two of these (**[A]/[C] – INC** and **[B]/[C] – INC**), the reporter RNA should bind if the ratio is high (e.g. [[A]/[C] >1/4). In practice, we check the binding for two concentration ratios. In the first, [A]=[C]=100 nM (e.g., ratio [A]/[C] = 1) and in the second, [A]=5 nM and [C]=100 nM (ratio [A]/[C] = 1/20). The other two puzzles (**[A]/[C] – DEC** and **[B]/[C] – DEC**), are identical, except that they should instead bind for low ratios.

The full TB signature is addressed in the two importantfinal puzzles: **[A]*[B]/[C]² INC** and **[A]*[B]/[C]² DEC**. In the first puzzle (**[A]*[B]/[C]² INC**, the designs should bind the 14-nt Reporter RNA oligo only when the ratio $[A][B]/[C]^2 > 1/16$. The second puzzle (**[A]*[B]/[C]² DEC**) is identical, except that the Reporter should not bind when the ratio is high, instead it should bind when the ratio is low ($[A][B]/[C]^2 < 1/16$. The ratio is a continuous variable but in the Eterna game we limit ourselves to show four different states:

					**State**	
**Condition**	**A (nM)**	**B (nM)**	**C (nM)**	**[A][B]/[C]²**	**[A]*[B]/[C]² INC**	**[A]*[B]/[C]² DEC**
**1**	100	0	100	0	OFF	ON
**2**	0	100	100	0	OFF	ON
**3**	50	50	300	1/36	OFF	ON
**4**	50	50	100	1/4	ON	OFF

Scoring of puzzles

Fold-change in K_D

As for our previous switch puzzles, the **fold-change in K_D** is probably the best single metric for good switching (see also Scoring of riboswitches in Eterna). For a simple binding curve, the fluorescent signal, *F*, from a reporter, *R*, binding to a design is $F[R] = \frac{[R]}{[R]+K_D}$, where the apparent dissociation constant, K_D, is the concentration at which half of the maximum signal is observed. This number is an indicator of how strong the apparent interaction is between the reporter and the design. For switches, the goal is to modulate this strength using the input oligos. The larger the change is in K_D between the ON and OFF-states the better the design is working. As we like to think of K_D in log-space, and to normalize for the binding in the ON-state, we use the ratio of the K_D in the OFF and ON-states (i.e., $K_{D,\,OFF}$ / $K_{D,\,ON}$) as a metric for the change.

For simple switches (**Sensor A (GBP5) – RIRI**, **Sensor B (DUSP3) – RIRI**, **Sensor C (KLF2) – RIRI**, **Sensor A (GBP5) – RIRO**, **Sensor B (DUSP3) – RIRO**, and **Sensor C (KLF2) – RIRO**), there is only one ON state and one OFF state (0 and 100 nM input oligo). However, for the other puzzles there are multiple states in either the ON or OFF states, or both. To account for this, we report the fold-change between the worst results for each state, i.e., the highest K_D from the ON states and the lowest K_D in the OFF states.

Global fold-change in K_D

Each puzzle is represented by a few states in the game, but a ratio such as $[A][B]/[C]^2$ is a continuous variable. Because we test many different puzzles with the same experimental pipeline, we also have the opportunity to map the behavior of the player designs for more conditions than the ones specified in the game. The global fold-change in K_D that we also report in the spreadsheets and in the figures (switch graphs) refers to the full $[A][B]/[C]^2$ ratio across all 16 well-defined conditions for the second round of the OpenTB challenge. A positive Global fold-change means that the design is good and the higher the ratio the better. This metric allows us to both better evaluate good results from each **$[A]*[B]/[C]^2$** puzzle and to spot designs for other puzzles that happen to work well for the full $[A][B]/[C]^2$ signature.

Below is the complete list of conditions tested in this round. Of the nineteen conditions, only the first sixteen have a well defined $[A][B]/[C]^2$ ratio.

					State	
**Condition**	**A (nM)**	**B (nM)**	**C (nM)**	**$[A][B]/[C]^2$**	**'INC' puzzles**	**'DEC' puzzles**
**1**	0	0	100	0	OFF	ON
**2**	100	0	100	0	OFF	ON
**3**	0	100	100	0	OFF	ON
**4**	5	0	100	0	OFF	ON
**5**	0	5	100	0	OFF	ON
**6**	5	5	100	0.0025 (1/400)	OFF	ON
**7**	50	50	300	0.028 (1/36)	OFF	ON
**8**	50	50	100	0.25 (1/4)	ON	OFF
**9**	25	100	100	0.25 (1/4)	ON	OFF
**10**	100	25	100	0.25 (1/4)	ON	OFF
**11**	200	200	300	0.44 (4/9)	ON	OFF
**12**	50	50	25	4	ON	OFF
**13**	100	100	0	∞	ON	OFF
**14**	5	5	0	∞	ON	OFF
**15**	100	5	0	∞	ON	OFF
**16**	5	100	0	∞	ON	OFF
**17**	0	0	0	undefined		
**18**	100	0	0	undefined		
**19**	0	100	0	undefined		

Eterna score

As for all previous puzzles, the designs were also given an Eterna score is between 0 and 100 that is a combination of three subscores for each puzzle (see also Scoring of riboswitches in Eterna).

The **Folding subscore** is determined by the intensity (F_{max}) of the ON state. For an F_{max} above 0.7 the score is 30, below 0.3 the score is 0, and in between the score increases linearly with the F_{max}.

The **Baseline subscore** is based on the K_D of the ON-state. The score is 100 for for K_D < 10 nM, 0 for K_D > 30 nM, and decreases linearly in between. There was no data for an oligo control this round so the 10 nM value was chosen arbitrarily.

The **Switch score** is based on the K_D of the ON-states and the OFF-states. The score is proportional to the logarithm of the fold-change in K_D between the ON- and OFF-states, i.e. $subscore \sim \log(K_{D,OFF}/K_{D,ON})$. The subscore is 0 if the ratio is less than 1 and the maximum score of 40 is reached when the ratio is above

- 75 (**Sensor A (GBP5) – RIRI**, **Sensor B (DUSP3) – RIRI**, **Sensor C (KLF2) – RIRI**, **Sensor A (GBP5) – RIRO**, **Sensor B (DUSP3) – RIRO**, and **Sensor C (KLF2) – RIRO,**)
- 15 (**A (AND) B – RI, A (AND) B – RO , [A]/[C] – INC**, **[B]/[C] – INC**, **[A]/[C] – DEC**, and **[B]/[C] – DEC**)
- 9 (**[A]*[B]/[C]² INC** and **[A]*[B]/[C]² DEC.**

Interpreting the plots

In addition to analyzing the raw numbers in the Eterna Data Browser, Google sheets, or Excel files, the "Switch graph" in the Data Browser displays the result in a visual format (see next page). It also summarizes the most important information such as the player, the design name and number, the scores and the fold change.

For each combination of input oligos (A, B, and C), millions of complete apparent binding curves were collected on our high-throughput array. Each design is represented by many (~100) clusters on a surface. Starting on the bottom left of the Switch graph, we display the data for all clusters in the small graphs, one per condition as indicated by the title. We fit data from each cluster individually and then extract the median K_D and F_{max} as indicated by the red horizontal and vertical lines. The distributions of K_D values is shown above the curves and the distribution of F_{max} values is shown to the right. Although we don't fit the median values at each 14-nt RNA oligo reporter concentration, these median values are also plotted (large dots) to help distinguish whether the binding exhibits non-typical binding, such as biphasic behavior that is indicative of more than one binding site.

Each condition is given a unique color and the fit values are listed on the top right of the Switch graph. Also listed is the value of the $[A][B]/[C]^2$ ratio. The conditions are ranked from the lowest ratio to the highest. A good solution for the full $[A][B]/[C]^2$ expression maximizes the separation in K_D (the fold change) between all the low-ratio conditions (all in red or orange) and all the high-ratio conditions (green or blue).

The idealized fit curves are also plotted together in the top middle part of the switch graph. The separation in K_D manifests itself as a left-right separation between the red/orange and green/blue curves, in particular their middle points. Depending on the direction of the switch, the red/orange curves should be either on the left (high low vs high ratios) or the right (high vs low ratios). This combined graph should make it easier to spot all the conditions that give the wrong signal (K_D too low or too high).

Another tool to analyze the results for all conditions is the 3D plot to the right. It is similar to the classic Eterna heatmaps but now with one axis for each of the three input oligo concentrations. The conditions are represented by dots and the perspective is set such that the plane for $[A][B]/[C]^2=1/16$ separates the low-ratio and high-ratio conditions The location of each dot is dictated by the concentrations for that condition and the color indicates the measured K_D. On the bottom is the target heatmap, with all points above the plane in yellow and all the point below in blue, or vice versa depending on the anticipated direction of the switching. Conditions with an undefined ratio (0 divided by 0) are indicated in grey. The goal of the OpenTB challenge is to design an RNA sequence that matches the target pattern as closely as possible. The example on the next page is the best solution from the current round (R105). The shades in the 3D plot largely matches the target. As you will find, many of the other solutions have yellow dots that should be blue and blue dots that are yellow. These are the conditions we have to fix by tweaking the sequence or architecture of the design.

Best design overall

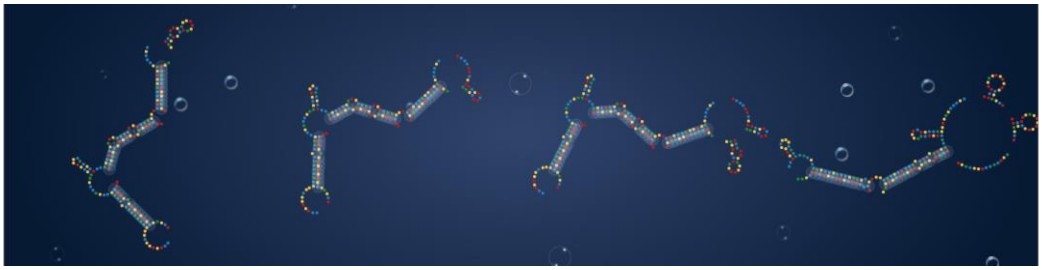

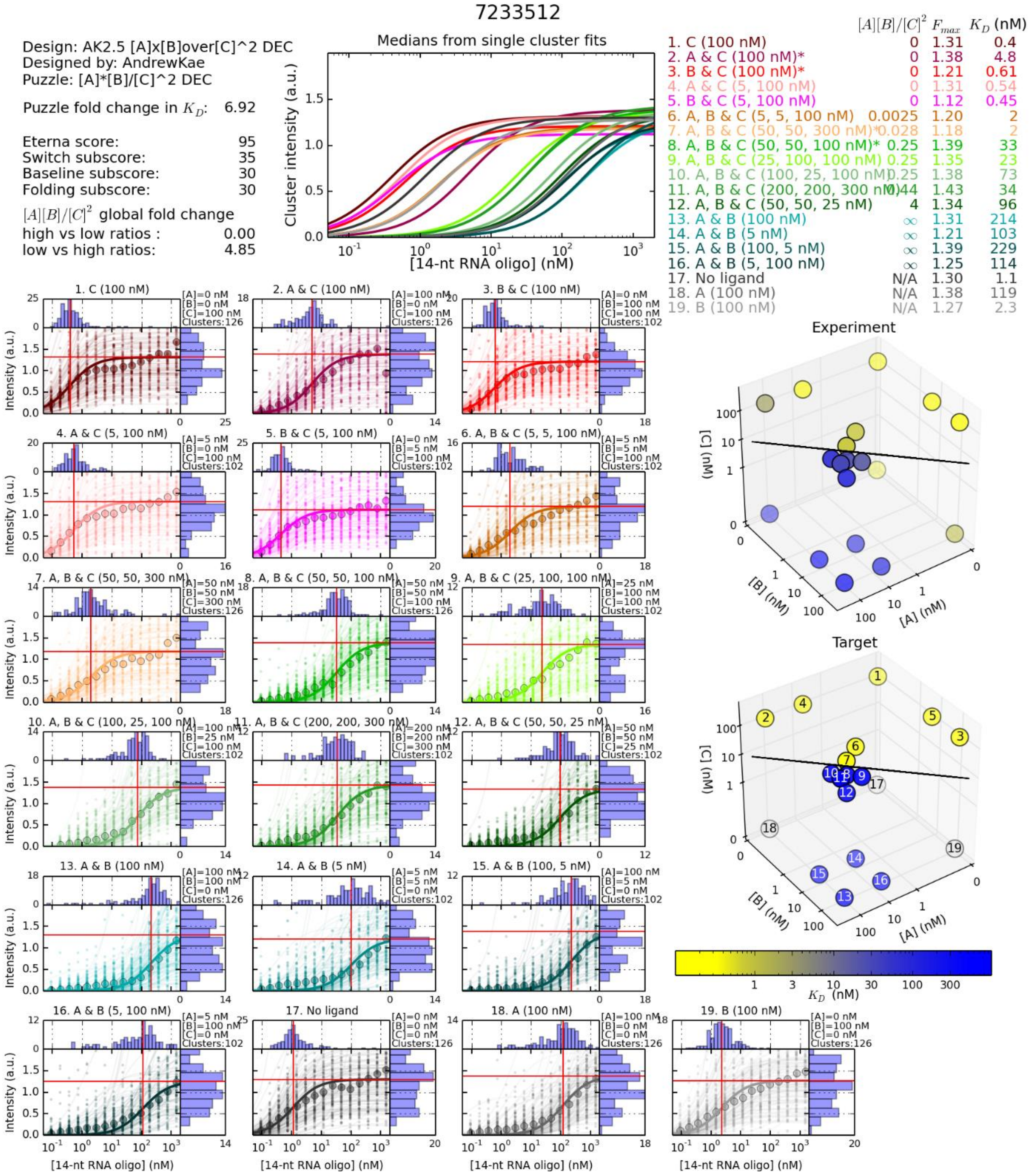

Sensor A (GBP5) - RIRI

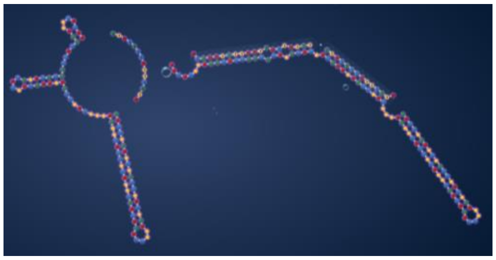

7140078, AK Sen A GBP5-RIRI 2.3, by AndrewKae , Eterna score 100

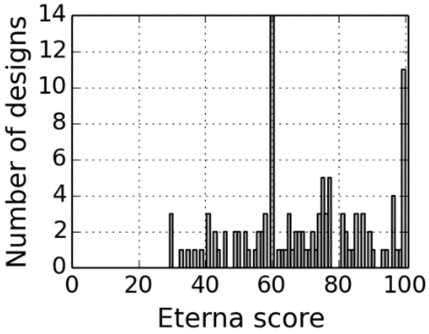

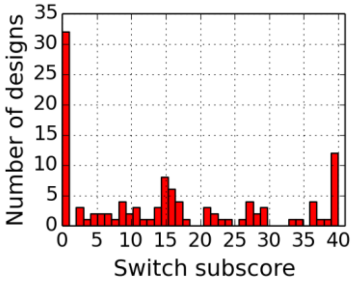

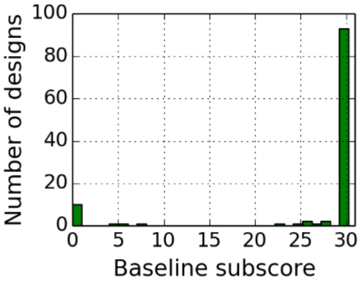

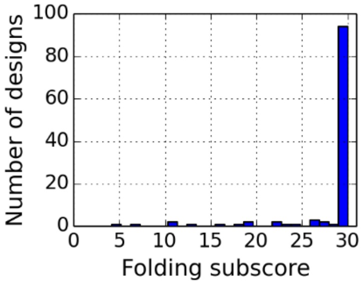

Dissociation constants (K_D)

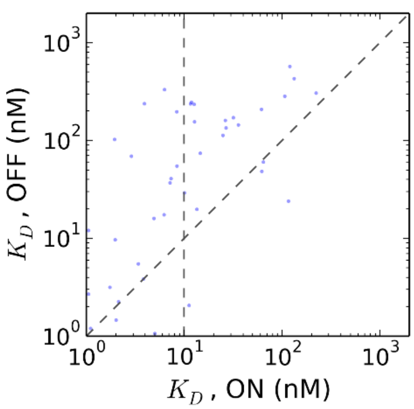

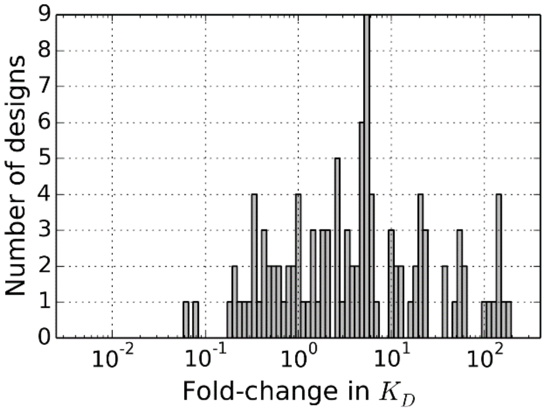

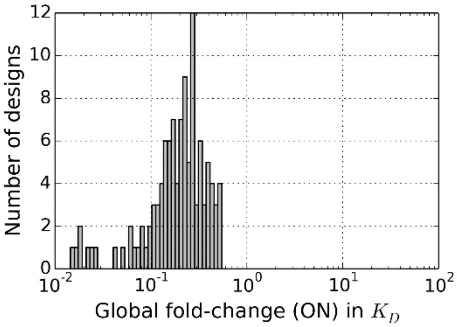

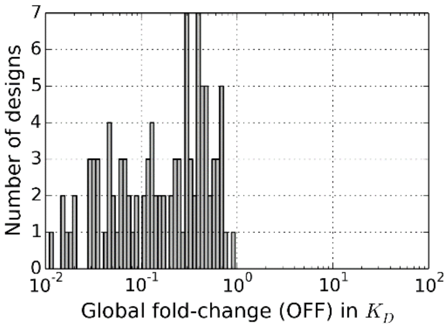

Sensor A (GBP5) - RIRO

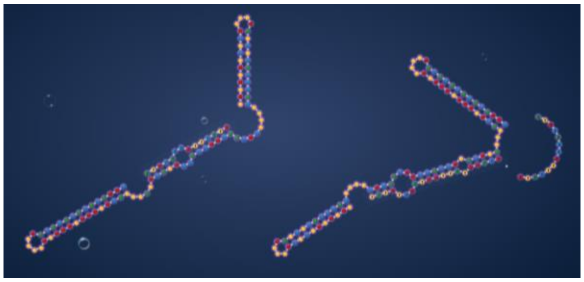

7250937, good energy, by sn69
Eterna score 100

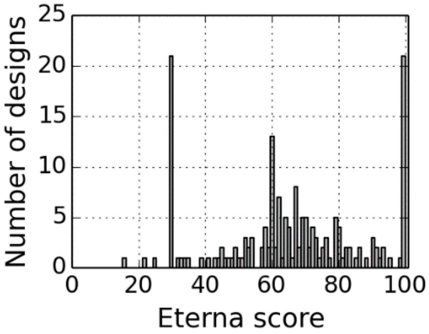

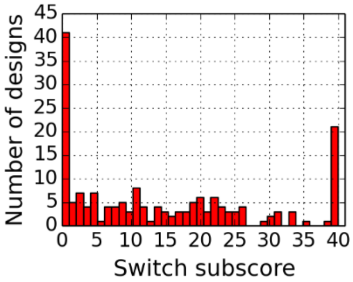

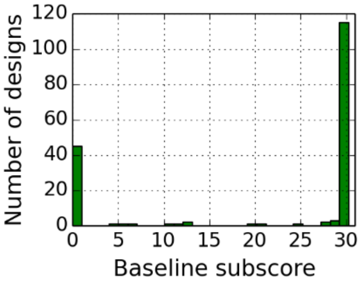

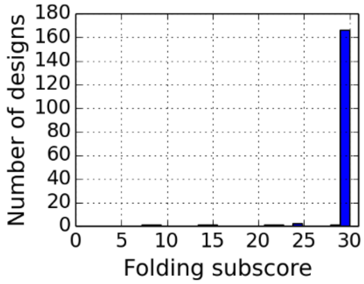

Dissociation constants (K_D)

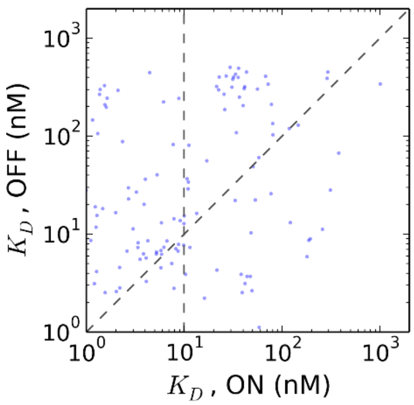

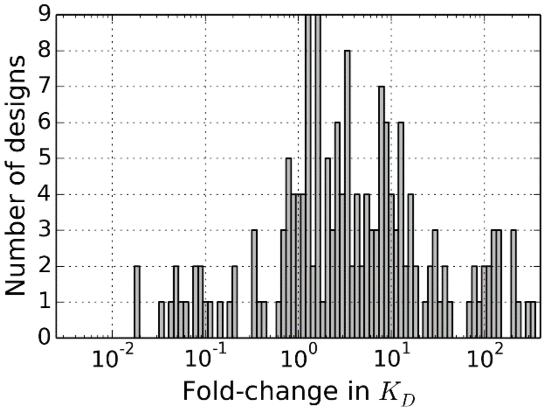

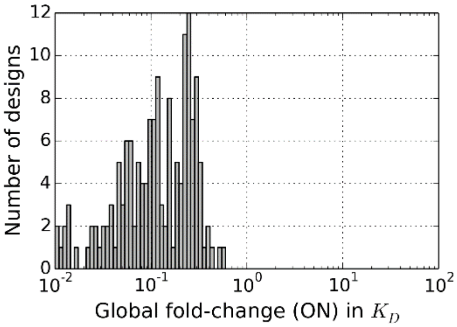

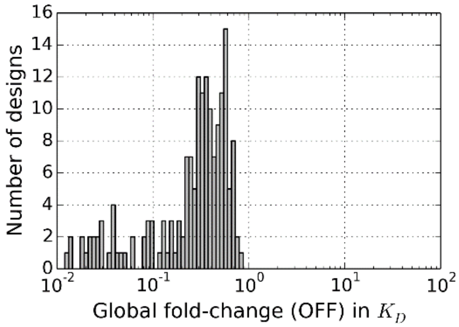

Sensor B (DUSP3) - RIRI

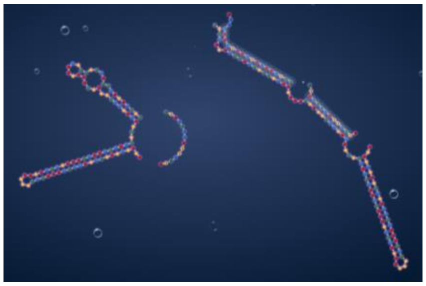

7124796, Lots of room to change things by Snowpup
Eterna score 100

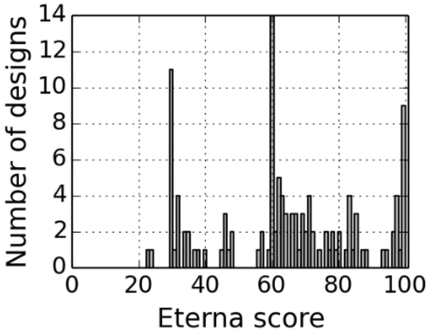

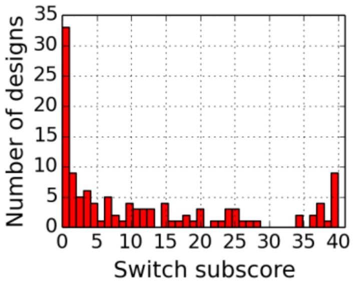

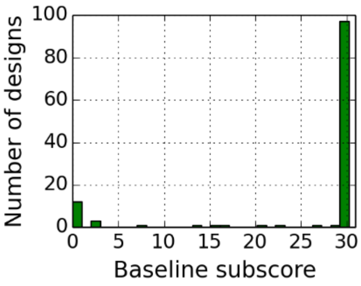

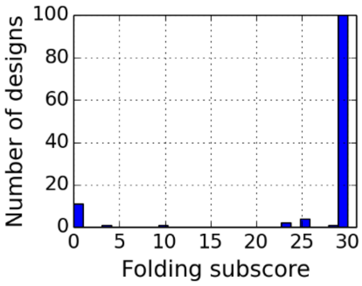

Dissociation constants (K_D)

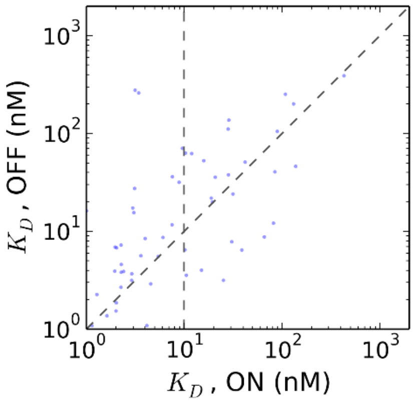

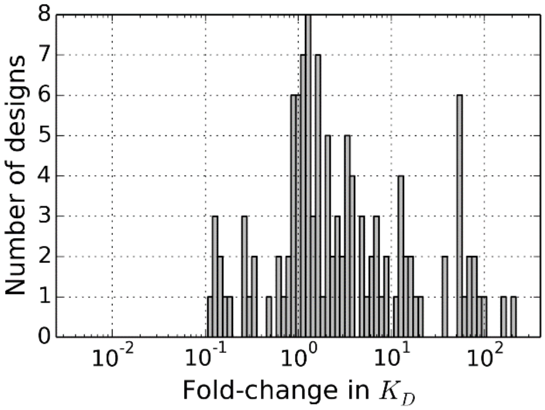

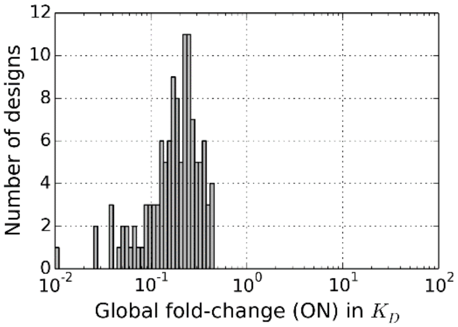

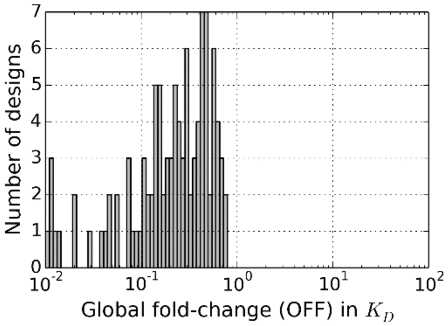

Sensor B (DUSP3) - RIRO

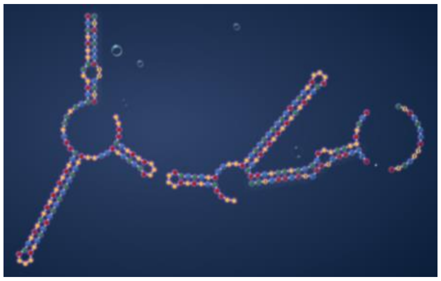

7232649, Solution 2 by IceBolt, Eterna score 100

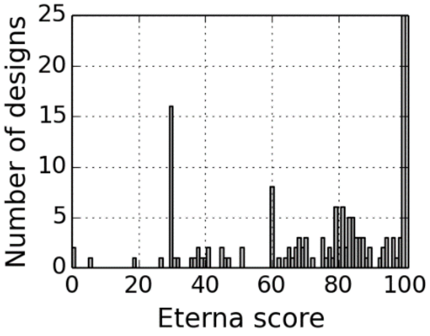

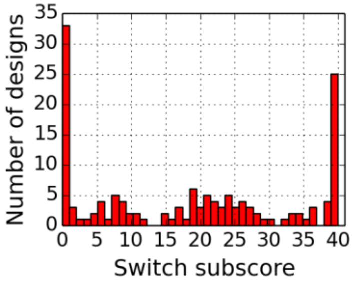

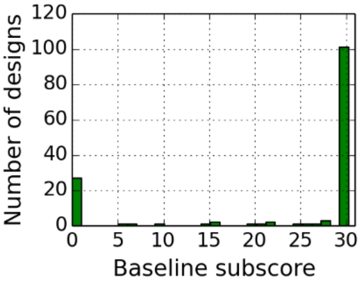

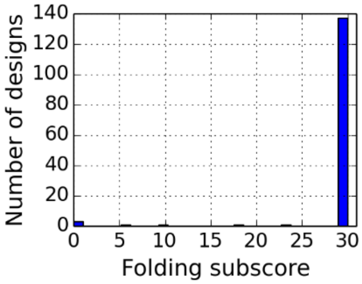

Dissociation constants (K_D)

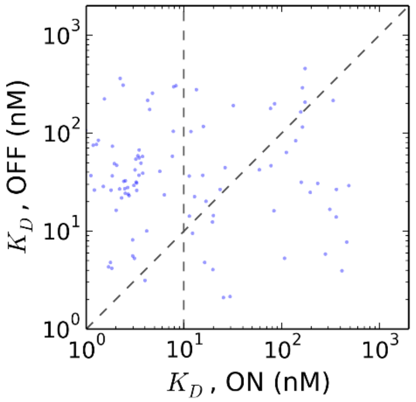

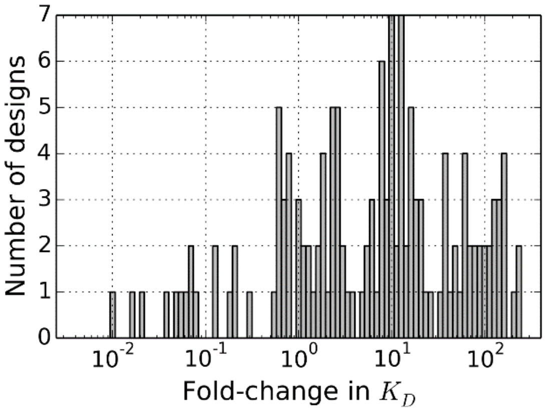

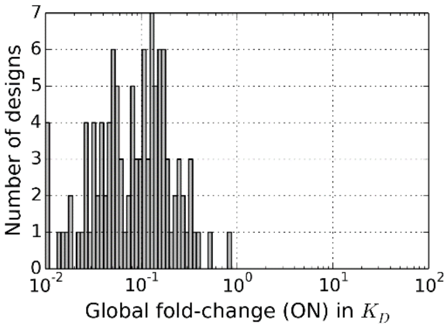

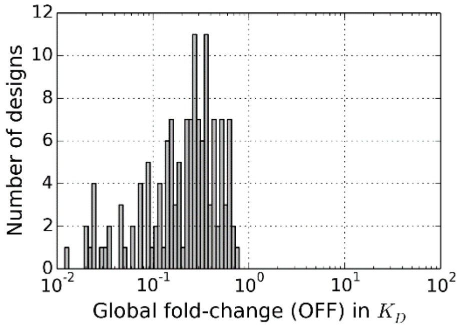

Sensor C (KLF2) - RIRI

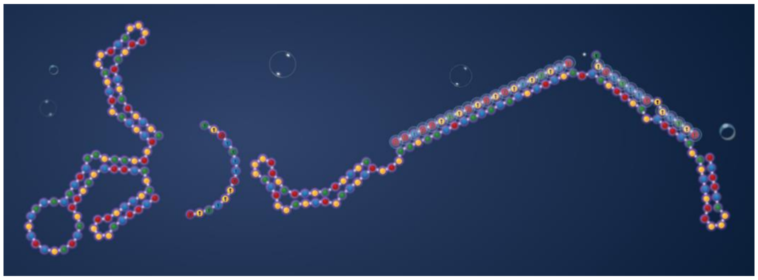

7238209, 9/12 #2 by Snowpup, Eterna score 100

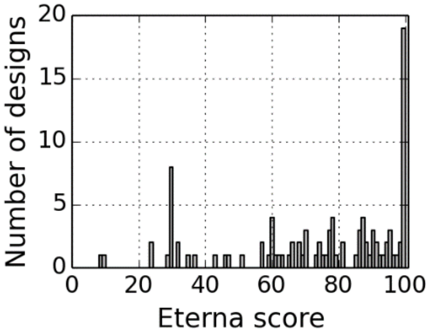

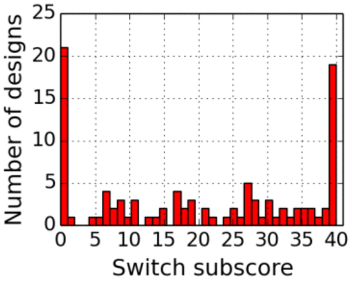

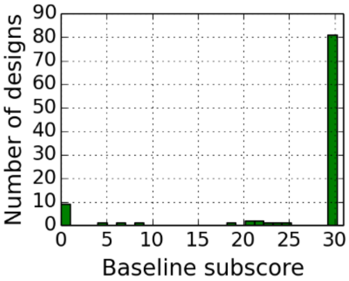

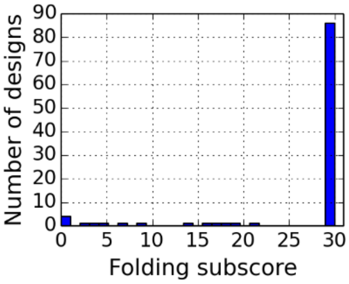

Dissociation constants (K_D)

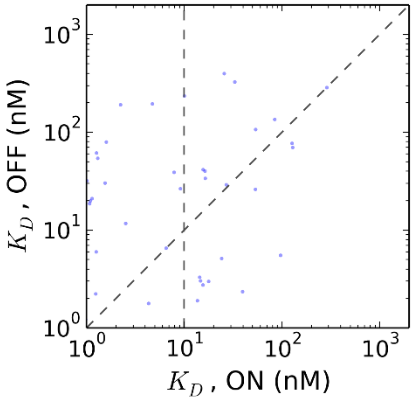

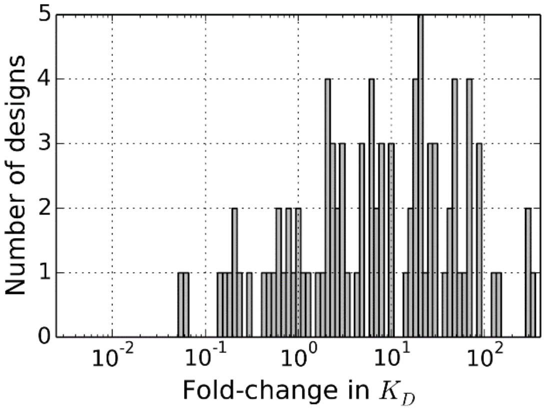

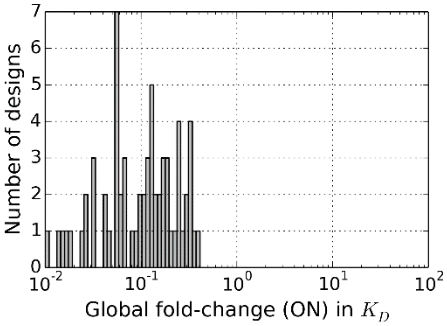

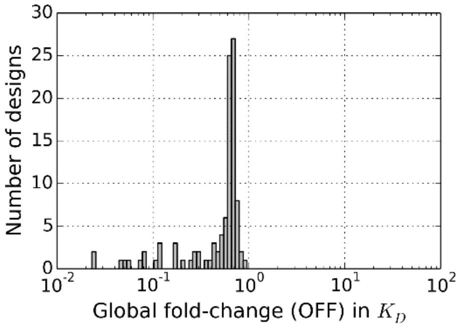

Sensor C (KLF2) - RIRO

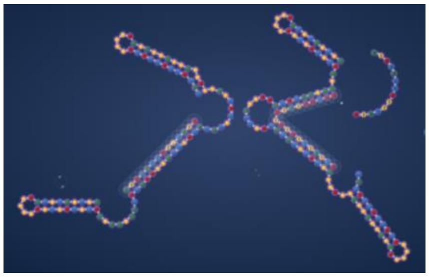

7241383, Malcolmmod3 by biggestlegoheroicafanever, Eterna score 100

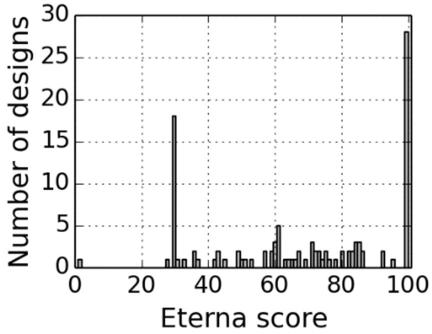

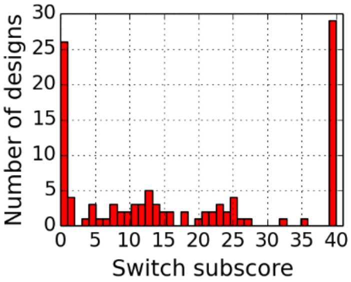

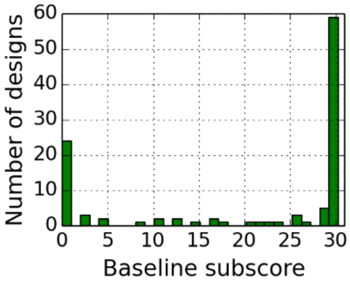

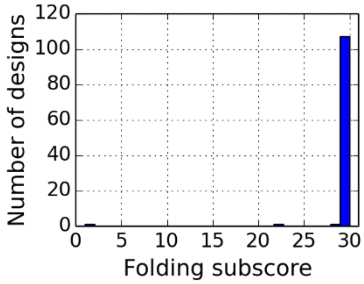

Dissociation constants (K_D)

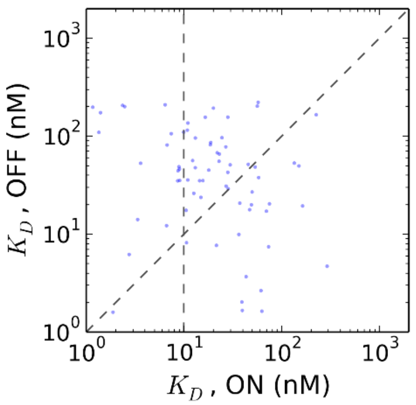

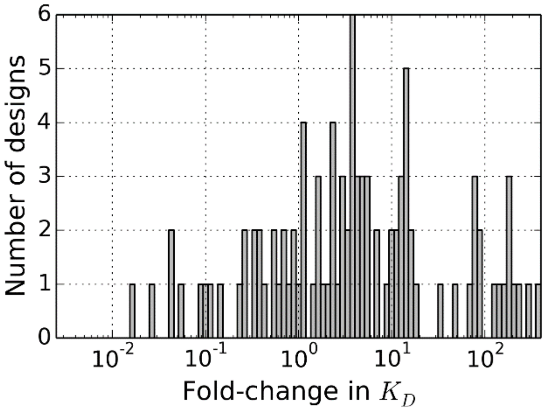

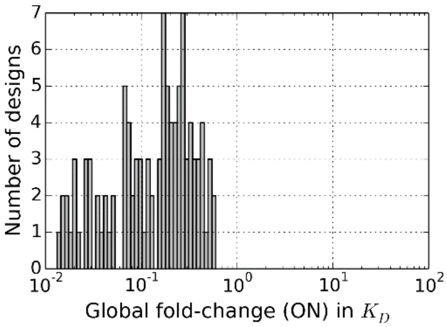

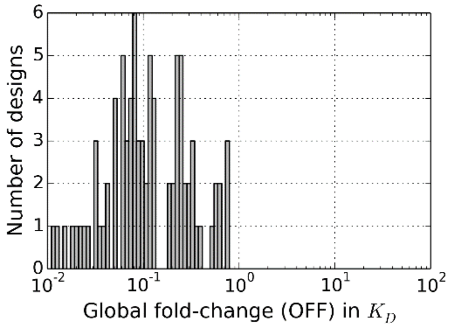

A * B - RI

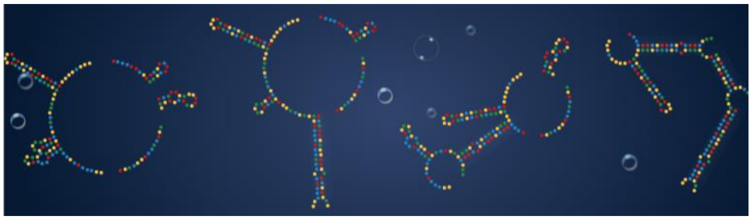

7253246, A * B - RI - Mat - Mod Eli _D8 by mat747, Eterna score 82

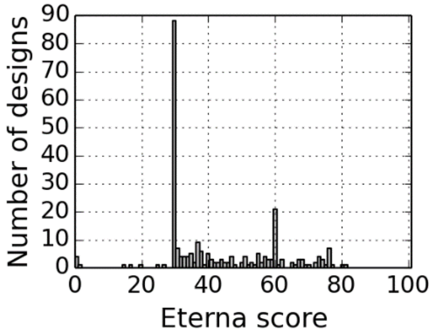

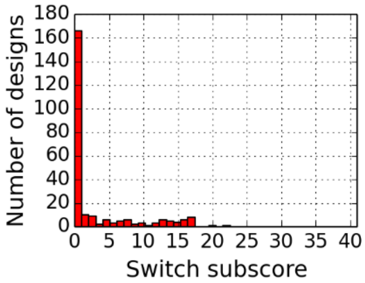

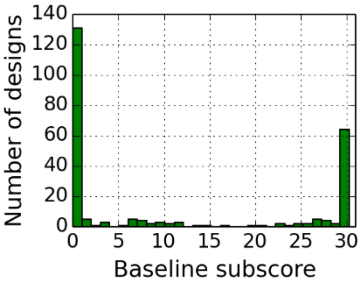

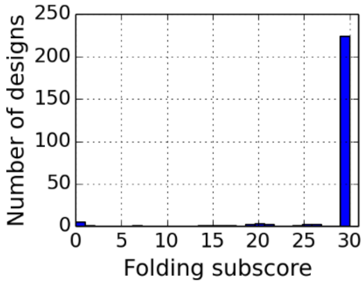

Dissociation constants (K_D)

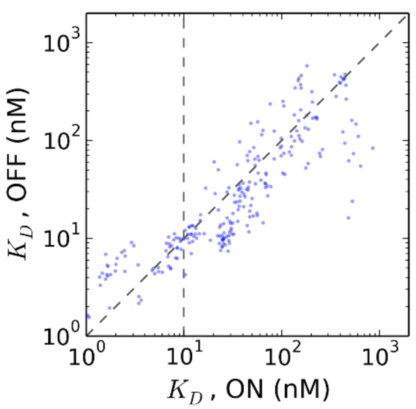

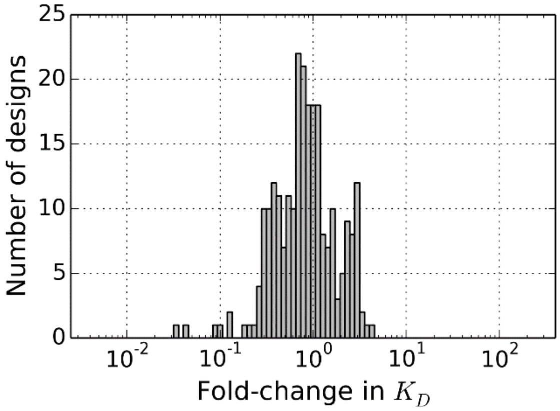

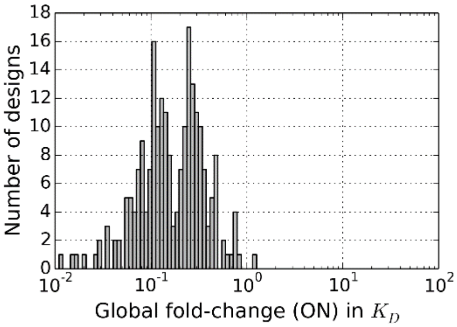

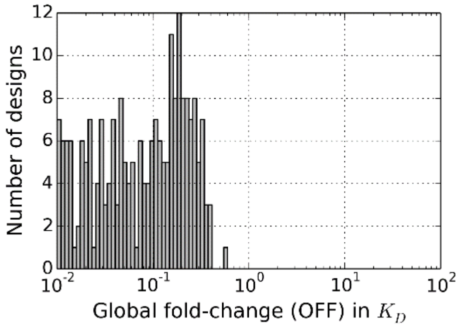

A * B - RO

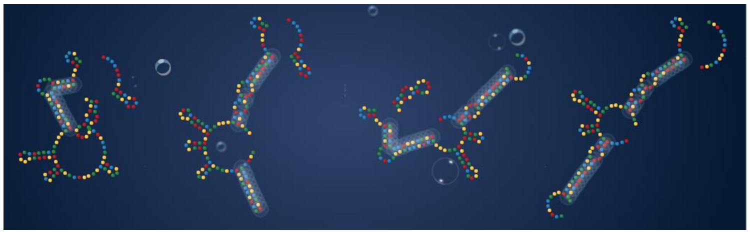

7243141, Oestric1 mod 7 - 7181215 by Eli Fisker, Eterna score 80

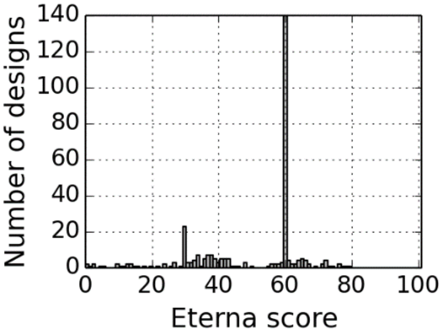

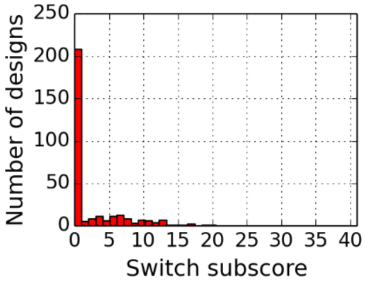

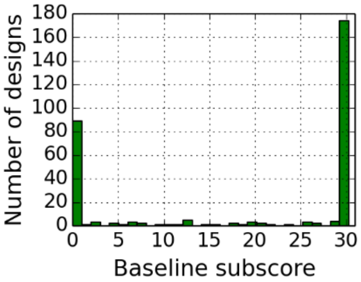

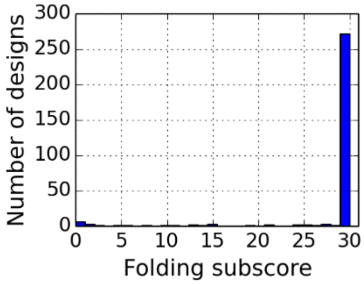

Dissociation constants (K_D)

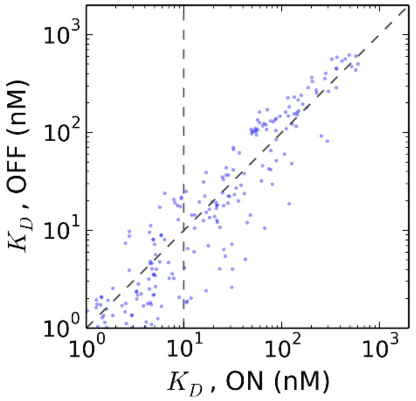

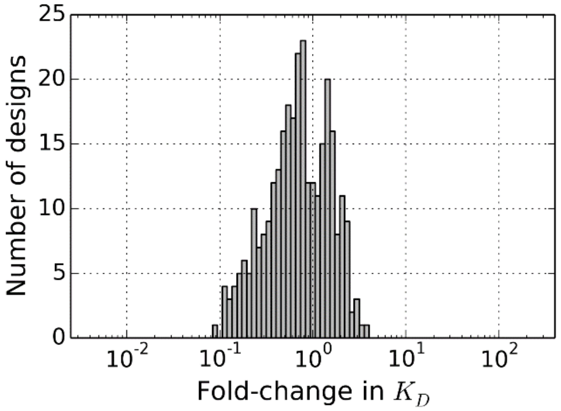

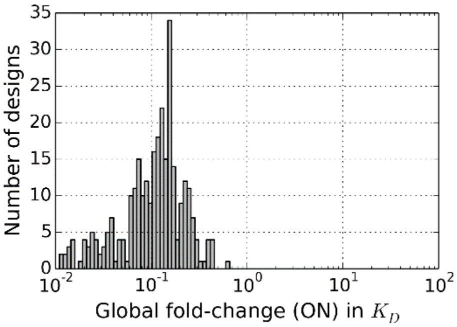

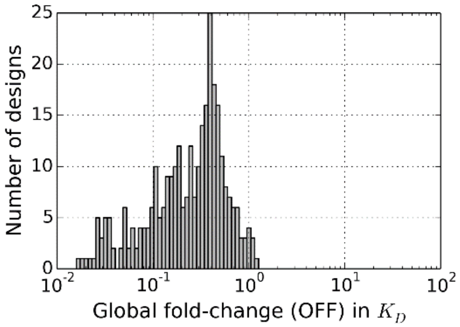

[A]/[C] - INC

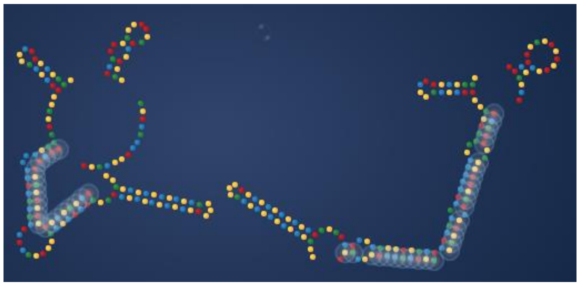

7221474, [A]/[C]-INC #5 by Malcolm, Eterna score 100

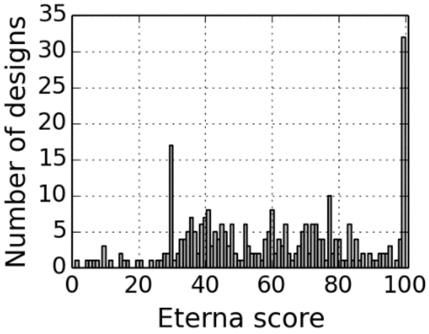

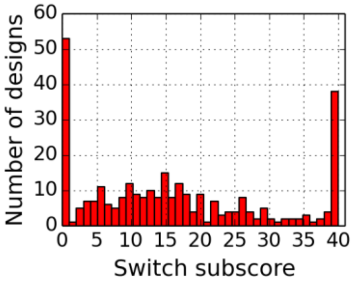

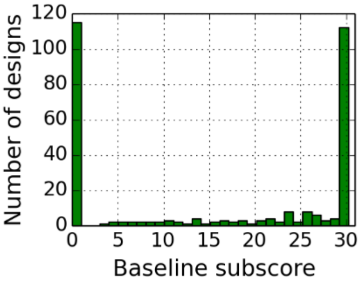

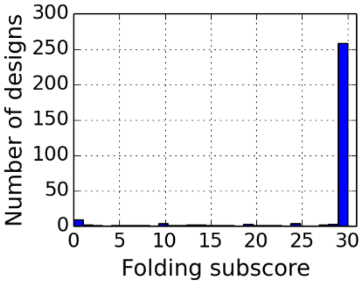

Dissociation constants (K_D)

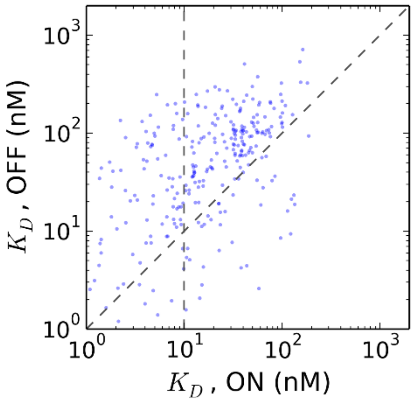

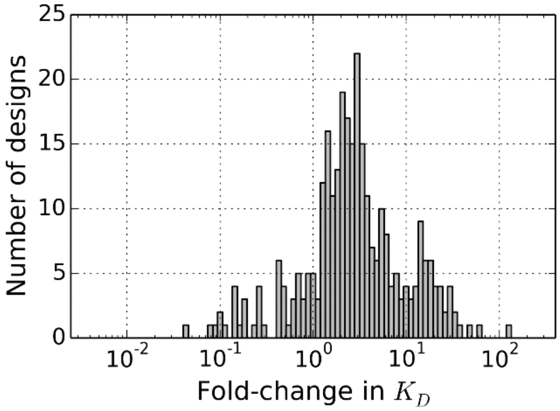

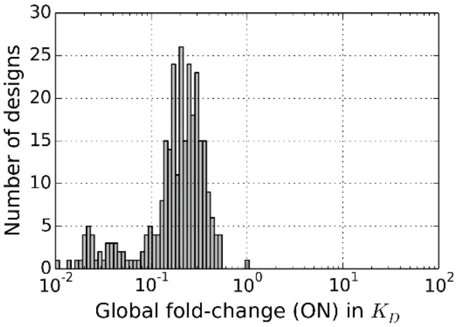

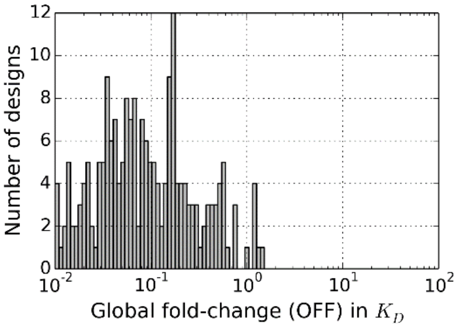

[A]/[C] - DEC

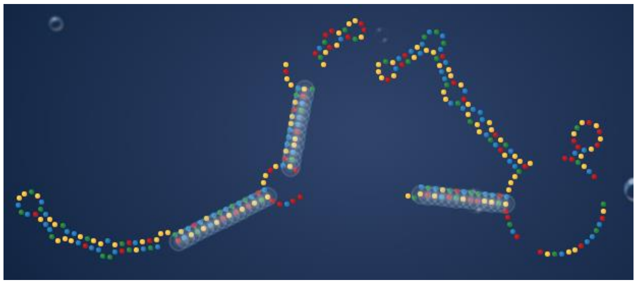

7152626, A/C - DEC - 6-4 by Brourd, Eterna score 100

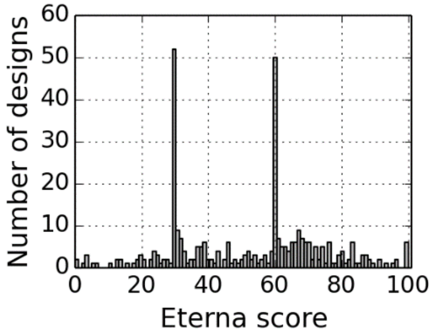

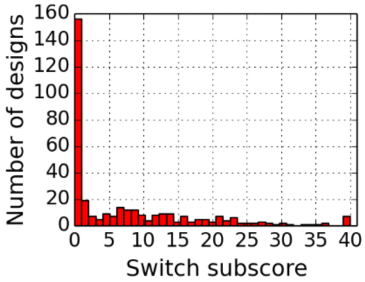

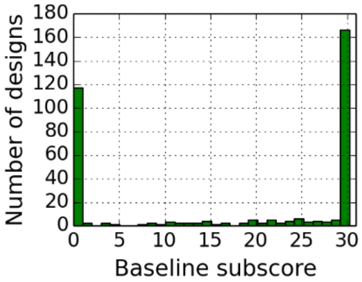

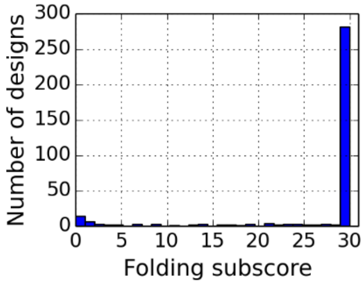

Dissociation constants (K_D)

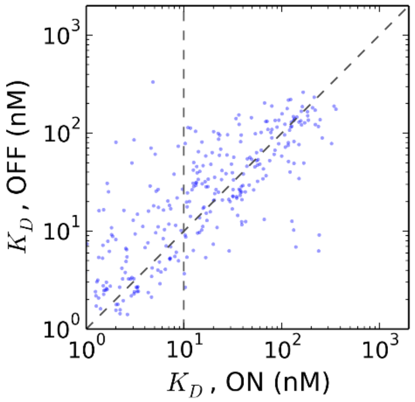

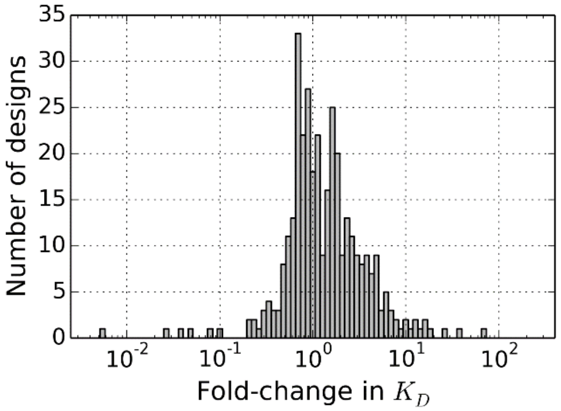

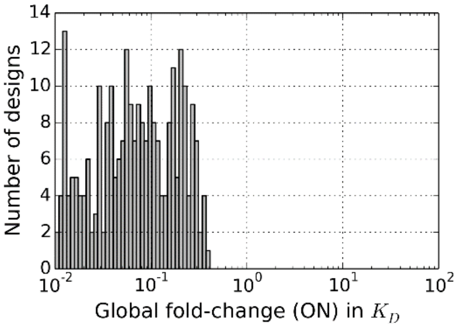

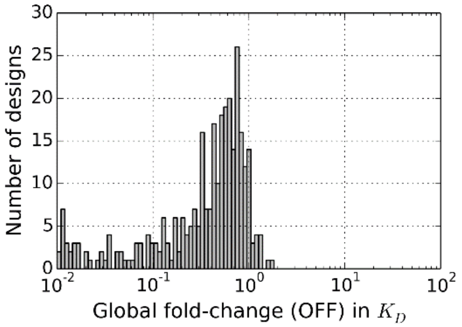

[B]/[C] - INC

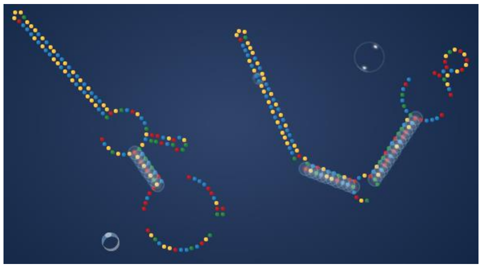

7230825, [B]/[C]-INC #13 by Malcolm, Eterna score 100

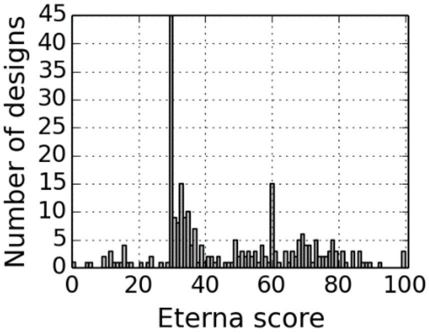

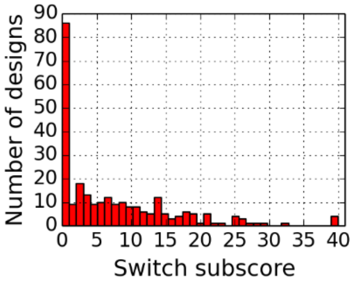

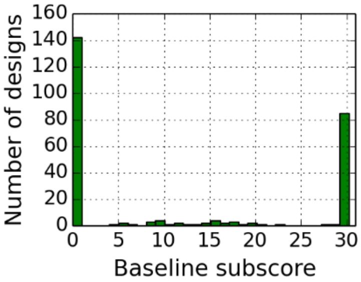

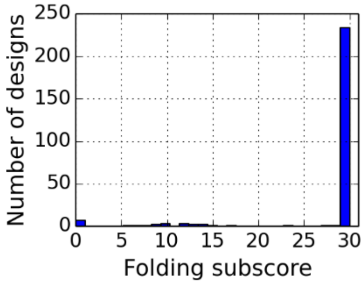

Dissociation constants (K_D)

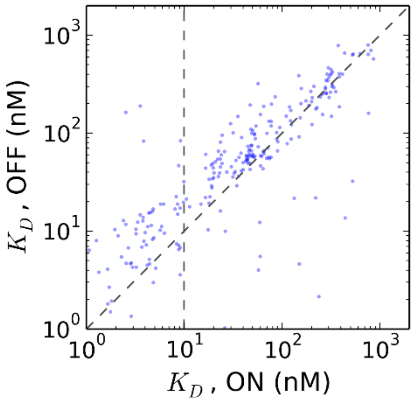

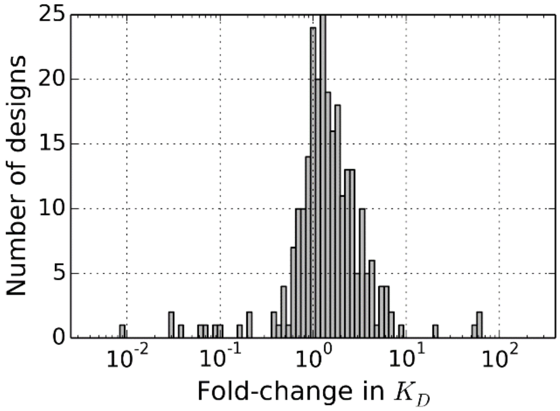

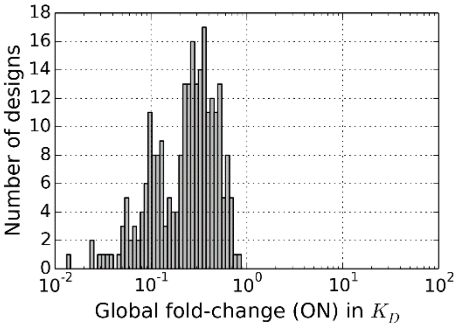

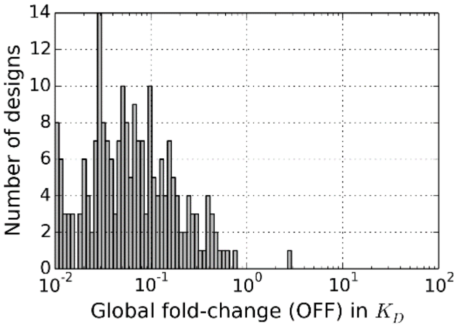

[B]/[C] - DEC

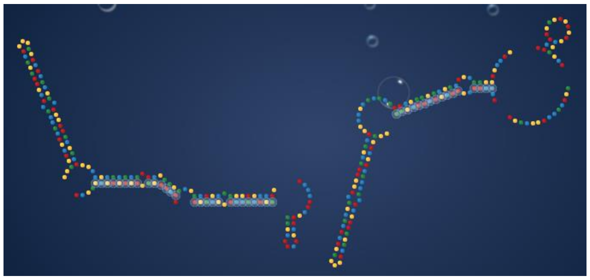

7124366, rBc 2 by Eli Fisker, Eterna score 100

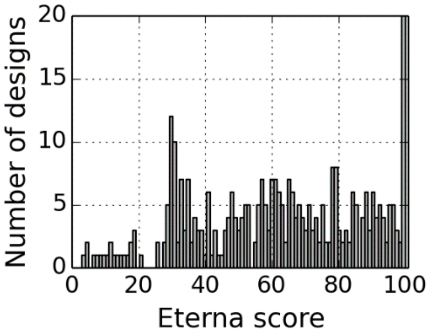

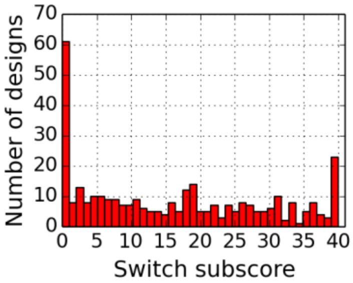

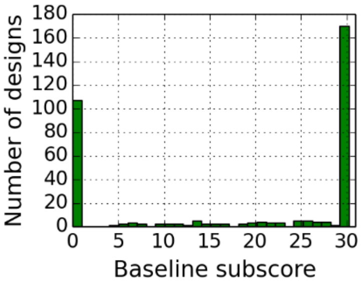

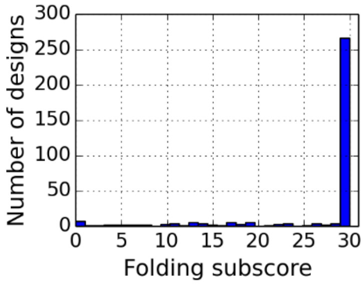

Dissociation constants (K_D)

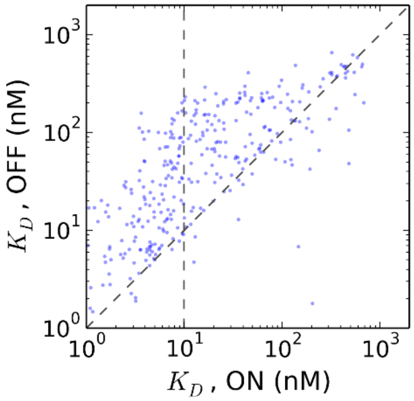

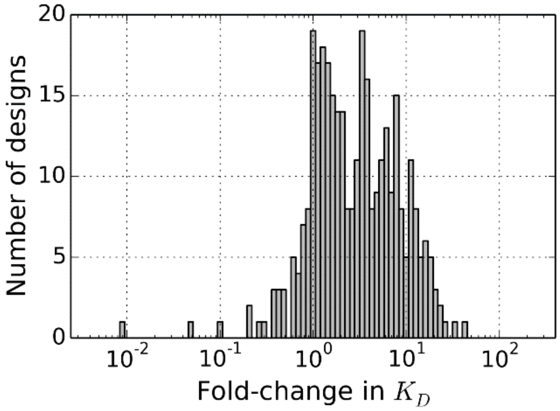

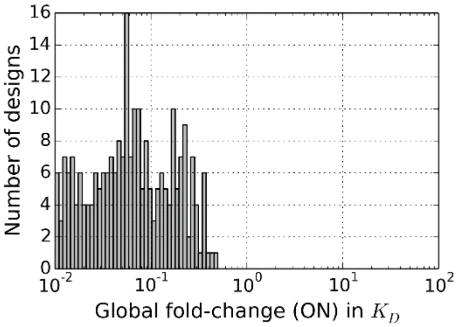

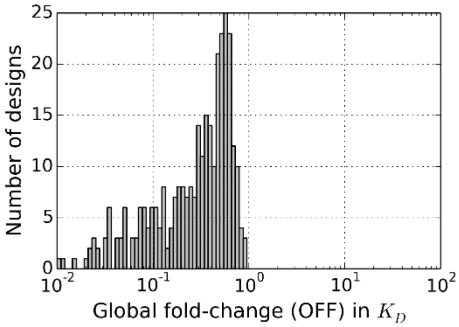

[A]*[B]/[C]^2 INC

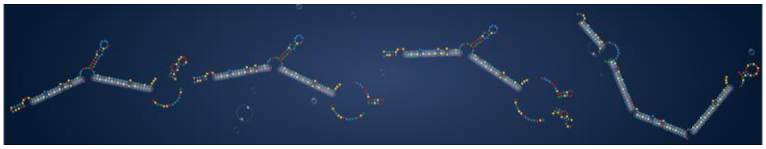

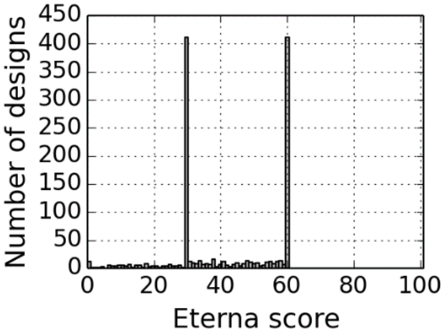

7219467, A*B/C^2 INC 13 3 by dl2007, Eterna score 60

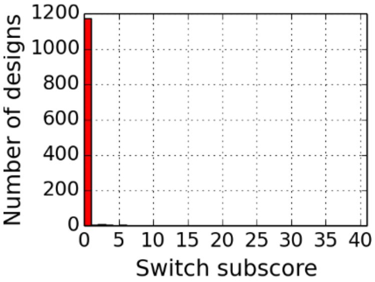

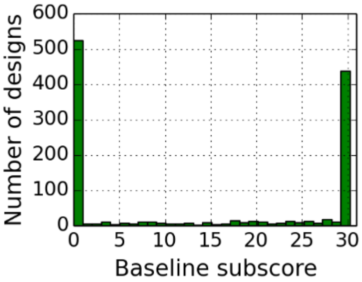

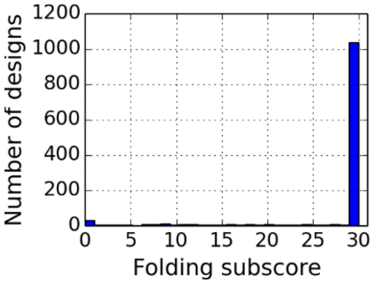

Dissociation constants (K_D)

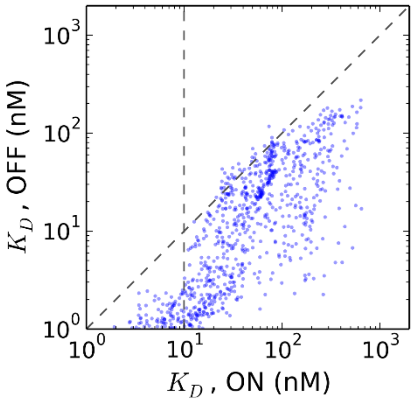

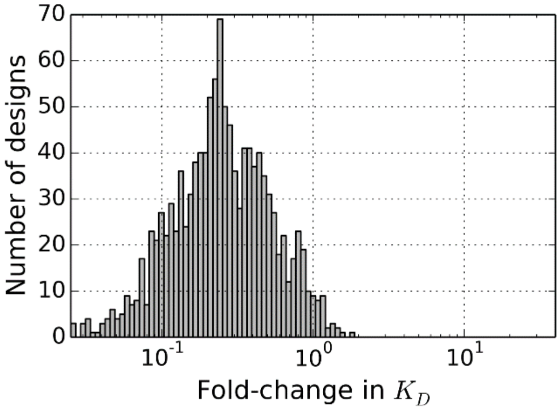

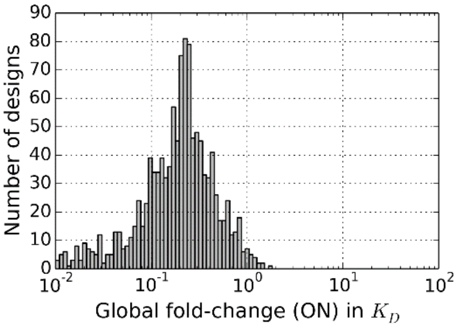

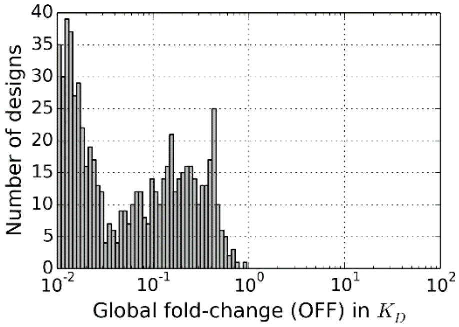

[A]*[B]/[C]² DEC

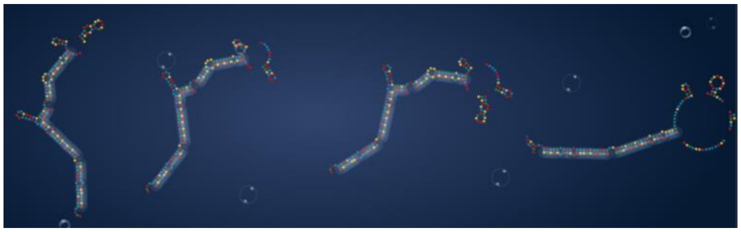

7172797, A*B/C^2 DEC 8 9 by dl2007, Eterna score 100

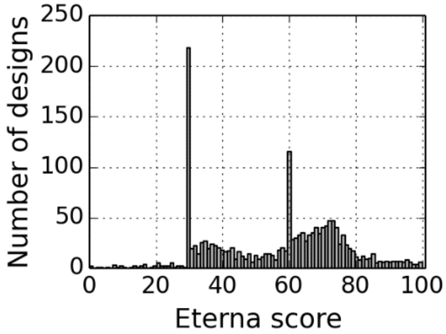

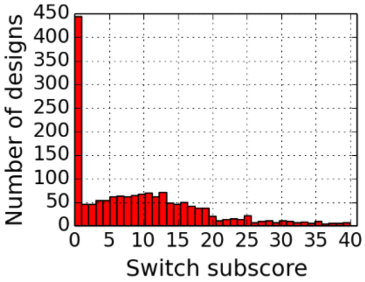

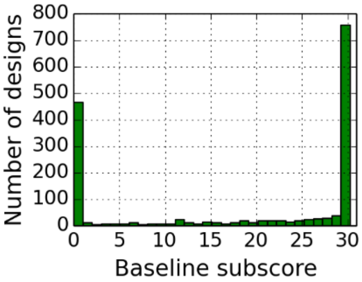

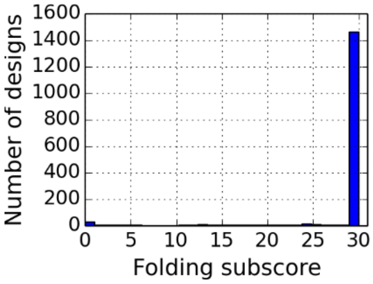

Dissociation constants (K_D)

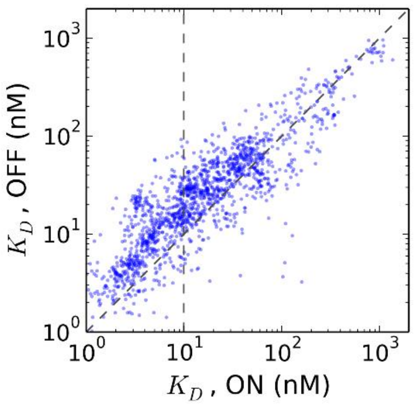

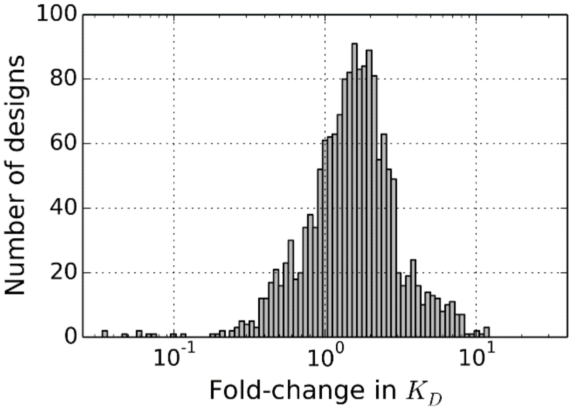

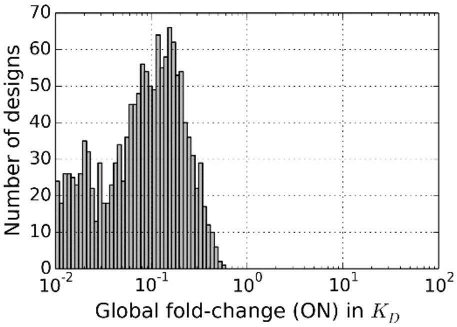

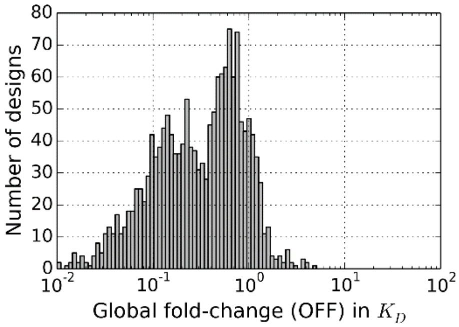

Supplement: Supplementary file 2 — Contains Supplementary Appendix: Eterna player resources (Eterna player resources also listed in Supplementary Table 4). Also contains the newsletters the Das lab shared with Eterna players after each design challenge. [file 41557_2025_1907_MOESM2_ESM.zip › supplemental_newsletter.zip!/supplemental_newsletter/Challenge_3_R105_openTB_results.pdf]
